# Unlocking Lactonase Enzymes as Biocatalysts for the Deracemisation of Chiral γ‐Thiolactones

**DOI:** 10.1002/anie.202505032

**Published:** 2025-05-29

**Authors:** Jingyue Wu, Michele Crotti, Ivan Bassanini, Mahdi Hassankalhori, Erica Elisa Ferrandi, Ferran Sancho, Daniela Monti, Daniele Castagnolo

**Affiliations:** ^1^ Department of Chemistry University College London Marshgate Building, Manufacturing Futures Lab, 7 Sidings Street London E20 2AE UK; ^2^ Istituto di Scienze e Tecnologie Chimiche “Giulio Natta” Consiglio Nazionale delle Ricerche Via Mario Bianco 9 Milano 20131 Italy; ^3^ Zymvol Biomodeling C/ Pau Claris, 94, 3B Barcelona 08010 Spain

**Keywords:** Biocatalysis, Kinetic resolution, Lactonase, Sulphur compounds, Thiolactones

## Abstract

Lactonases, a class of metalloenzymes that exhibit catalytic promiscuity, have been extensively studied from a biological perspective, yet their application as biocatalysts remains underexplored. In this study, we disclose the biocatalytic activity of lactonase enzymes in the hydrolysis and deracemisation of chiral C3‐substituted‐γ‐thiolactones and the asymmetric synthesis of γ‐thio‐α‐substituted‐carboxylic acids. The thiolactonase activity of lactonases from different protein superfamilies was investigated. The biocatalyst GcL, from the metallo‐β‐lactamase‐like lactonase family, catalysed the enzymatic kinetic resolution (EKR) of homocysteine (Hcy) thiolactones with excellent enantioselectivity (*E*‐value up to 136), yielding enantioenriched Hcy thiolactones and γ‐thio‐α‐amino‐carboxylic acids with high ees. Additionally, the biocatalyst N9 Y71G, a rationally engineered variant of the reconstructed ancestral paraoxonase enzyme N9, catalysed the dynamic kinetic resolution (DKR) of C3‐thio‐γ‐thiolactones, yielding γ‐thio‐α‐thio‐carboxylic acids in enantioselective manner with high ees (up to >99%) and yields (up to >99%). Insights on the mechanism and the stereoselectivity of the lactonase biocatalysts were gained through computational and site‐directed mutagenesis studies.

## Graphical abstract

The application of lactonase enzymes as biocatalysts still remains underexplored. This study discloses the biocatalytic activity of two lactonases in the deracemisation of chiral C3‐substituted‐γ‐thiolactones. The biocatalyst GcL catalyses the enzymatic kinetic resolution (EKR) of C3‐amide‐thiolactones, while the rationally engineered biocatalyst N9 Y71G catalyses the dynamic kinetic resolution (DKR) of C3‐thio‐thiolactones.

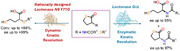

## Introduction

Quorum‐quenching (QQ) lactonases are a diverse class of metalloenzymes that catalyse the degradation of *N*‐acyl homoserine lactones (AHLs), which are key signalling molecules involved in bacterial quorum sensing (QS). These enzymes have been identified across a wide range of organisms, including archaea, bacteria, fungi, and mammals.^[^ ^1^, ^2^, ^3^, ^4^, ^5^, ^6^, ^7^, ^8^ ^]^ They are classified into three major protein superfamilies: metallo‐β‐lactamase‐like lactonases (MLLs), phosphotriesterase‐like lactonases (PLLs), and paraoxonases (PONs).^[^ ^9^ ^]^ MLLs feature a characteristic αβ/βα sandwich fold and a conserved “HXHXDH” motif, which is involved in the formation of a bimetallic active site.^[^ ^10^, ^11^, ^12^, ^13^, ^14^, ^15^, ^16^ ^]^ These enzymes typically exhibit broad substrate specificity, effectively hydrolysing AHLs and 3‐oxo‐AHLs,^[^ ^9^ ^]^ and, in some cases, other lactones, such as γ‐, δ‐, ϵ‐, and whiskey lactones.^[^ ^15^, ^16^ ^]^ PLLs are lactonases showing a promiscuous phosphotriesterase activity^[^ ^3^ ^]^ and are divided into two subclasses (PLL‐A and PLL‐B) based on structural and enzymatic properties. PLL‐As, such as SsoPox,^[^ ^17^, ^18^ ^]^ SisLac,^[^ ^19^ ^]^ PPH,^[^ ^20^ ^]^ and MCP,^[^ ^21^ ^]^ hydrolyse AHLs and other γ‐ or δ‐lactones, contributing to QS regulation. In contrast, PLL‐Bs, including Dr0930,^[^ ^22^, ^23^ ^]^ Gsp,^[^ ^24^ ^]^ and GKL,^[^ ^25^ ^]^ primarily degrade γ‐ or δ‐lactones while showing minimal or no activity towards AHLs. PONs, predominantly found in mammals,^[^ ^2^, ^3^, ^26^ ^]^ are also present in some vertebrates and nematodes.^[^ ^27^, ^28^ ^]^ This family includes three members, PON1, PON2, and PON3, which share 65% sequence identity^[^ ^29^ ^]^ and adopt a six‐bladed β‐propeller fold with a central tunnel containing one structural and one catalytic calcium cation.^[^ ^30^, ^31^, ^32^ ^]^ PON1 is notable for its ability to hydrolyse toxic organophosphates, including pesticide metabolites (parathion, diazinon, and chlorpyrifos) and nerve agents (sarin, soman).^[^ ^33^ ^]^ Additionally, PON1 breaks down aromatic esters^[^ ^34^ ^]^ and various δ‐ and γ‐lactones with lipophilic side chains.^[^ ^35^, ^36^, ^37^, ^38^ ^]^ PON3 has lower arylesterase and minimal paraoxonase activity but shares lipo‐lactone substrates with PON1,^[^ ^39^, ^40^, ^41^ ^]^ whereas PON2 lacks paraoxonase, aryl‐lactonase, or lipo‐lactonase activity but specifically hydrolyses the QS molecule 3OC12‐HSL.^[^ ^42^, ^43^ ^]^ Overall, PONs exhibit a broad range of substrate specificity beyond lactone hydrolysis, extending to organophosphates, aryl esters, carbonates^[^ ^36^ ^]^ and, to a limited extent, thiolactones.^[^ ^44^ ^]^

While lactonases have been biologically studied in an extensive manner, their use as biocatalysts in organic synthesis remains limited. Only a few lactonases, including those from *Fusarium* strains, *Agrobacterium tumefaciens* Lu681, and *Thielavia* sp. zmu20201, have been reported to selectively hydrolyse 3‐hydroxy‐γ‐butyrolactone substrates.^[^ ^45^, ^46^, ^47^, ^48^, ^49^, ^50^ ^]^ These enzymes exhibit good stereoselectivity but are restricted to lactones with a hydroxyl group at the C3 position of the lactone ring. Other than lactonases, only a few hydrolase enzymes have been reported to hydrolyse macrocyclic lactones, such as the zearalenone mycotoxin.^[^ ^51^, ^52^, ^53^ ^]^ Building on our previous research on the stereoselective biocatalytic synthesis of chiral sulphur compounds,^[^ ^54^, ^55^, ^56^, ^57^, ^58^, ^59^ ^]^ we became interested in leveraging lactonases for the deracemisation of C3‐substituted‐γ‐thiolactones and the synthesis of chiral γ‐thio‐α‐substituted‐carboxylic acids.

**Figure 1:**
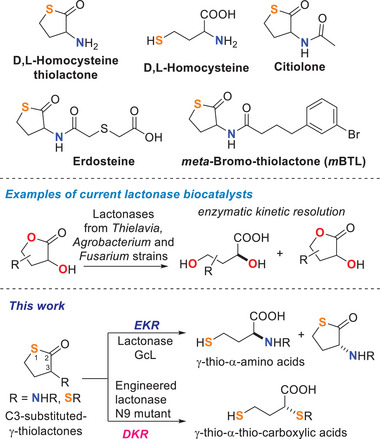
Previous works on lactonase biocatalysts and aims of the present work.

Chiral γ‐thiolactones, such as homocysteine (Hcy) thiolactone and its derivatives, i.e., the antioxidant erdosteine, the drug citiolone used in liver therapy, or the quorum‐sensing inhibitor *meta*‐bromo‐thiolactone (*m*BTL), find application in organic, medicinal, and materials chemistry (Figure 1). Similarly, γ‐thiocarboxylic acids, derived from γ‐thiolactone hydrolysis, such as homocysteine, serve as key building blocks in the synthesis of drugs and peptides,^[^ ^60^, ^61^, ^62^ ^]^ as well as in materials science^[^ ^63^, ^64^, ^65^, ^66^ ^]^ and as chiral ligands in organometallic chemistry.^[^ ^67^, ^68^ ^]^ Current methods for synthesising chiral thiolactones and thiocarboxylic acids typically rely on chiral pool synthesis using naturally derived precursors like amino acids^[^ ^69^, ^70^, ^71^, ^72^ ^]^ and hydroxycarboxylic acids.^[^ ^73^, ^74^ ^]^ While effective for stereoselective outcomes, these approaches often require harsh reaction conditions, with the stereocontrol dictated only by the starting materials' inherent configuration.

Herein, we describe the identification of two lactonase enzymes, namely GcL from *Parageobacillus caldoxylosilyticus* NBRC 107762 (MLL family)^[^ ^16^ ^]^ and the mammalian PONs ancestor N9,^[^ ^7^ ^]^ and their application as biocatalysts in the deracemisation of chiral C3‐substituted‐γ‐thiolactones. We demonstrated that the two lactonases present a high activity towards substituted γ‐thiolactone substrates and may be employed in the stereoselective synthesis of chiral γ‐thio‐α‐amino acids (GcL) and γ‐thio‐α‐thiocarboxylic acids (N9). Wild‐type GcL proved to be an excellent biocatalyst in the enzymatic kinetic resolution (EKR) of 3‐acylamide‐γ‐thiolactones, while N9 was rationally bioengineered to give N9 variants able to catalyse the dynamic kinetic resolution (DKR) of 3‐alkyl/arylthio‐γ‐thiolactones with excellent stereoselectivities and yields (Figure 1). To the best of our knowledge, this is the first methodology disclosing the potential of lactonase enzymes as biocatalysts in the deracemisation of γ‐thiolactones and the synthesis of enantioenriched chiral Hcy analogues.

## Results and Discussion

### Initial Screening of Lactonase Enzymes on γ‐Thiolactones

Three lactonases, each from one of the three major lactonase protein superfamilies, VmutPLL from *Vulcanisaeta moutnovskia* (PLL family),^[^ ^75^ ^]^ GcL from *Parageobacillus caldoxylosilyticus* (MLL family),^[^ ^16^ ^]^ and N9, a reconstructed ancestral enzyme from the PON family,^[^ ^7^ ^]^ were selected for this study based on available literature data. This choice was made considering both the phylogenetic distance of these three enzymes with distinct structural and functional characteristics and their well‐known broad substrate specificity. Moreover, thanks to their thermophilic or ancestral origin, these enzymes exhibit intrinsic stability, making them excellent candidates for biocatalytic synthetic applications.

The three enzymes were first screened as biocatalysts on the thiolactones **1a** and **1b**, bearing respectively a hexanoyl and a benzoyl amide group at the C3 position of the thiolactone ring, on the lactone **3a**, and on the thiolactone **5a** bearing a phenylthio group at position C3 (Table 1). The biocatalytic reactions were carried out in Tris‐HCl buffer, pH 8.0, at 37 °C for variable times. According to the currently accepted catalytic mechanism,^[^ ^8^, ^30^, ^75^ ^]^ lactonase enzymes require the presence of a metal in the catalytic binding pocket to promote the hydrolysis of lactone substrates. Thus, different and appropriate metal additives, namely CoCl_2_ for GcL, MnCl_2_ for VmutPLL, and CaCl_2_ for N9, were added at 1 mM concentration to the reaction mixtures.

**Table 1:** Screening of lactonase enzymes on γ‐thiolactones **1a‐b** and **5a**, and on lactone **3a**.

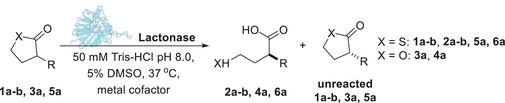						
Entry	Substrate	Enzyme	Time	Conv. (%)^a)^	Unreacted thiolactone ee (%)^a)^	Acid ee (%)^a)^
1	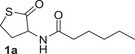	GcL	1 h	64^b)^	>99	57
2		GcL	10 min	51^b)^	88	84
3		VmutPLL	1 h	n.d.^c)^	Racemic	n.d.^c)^
4		N9	1 h	n.d.^c)^	Racemic	n.d.^c)^
5		No enzyme	1 h	<1^b)^	Racemic	n.d.^c)^
6	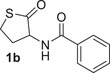	GcL	1 h	46	73	86
7		VmutPLL	1 h	<1	Racemic	n.d.^c)^
8		N9	1 h	<1	Racemic	n.d.^c)^
9		No enzyme	1 h	<1	Racemic	n.d.^c)^
10	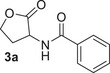	GcL	15 h	93	58	11
11		VmutPLL	15 h	78	Racemic	Racemic
12		N9	15 h	78	Racemic	Racemic
13		No enzyme	15 h	78	Racemic	Racemic
14	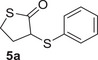	GcL	15 min	>99	n.d.^c)^	Racemic
15		N9	24 h	36	Racemic	47
16		No enzyme	18 h	<1%	Racemic	n.d.^c)^

All the reactions were conducted at 1 mL scale with 30 mM thiolactone substrate, 1 mM metal cofactor (CoCl_2_ for GcL, MnCl_2_ for VmutPLL, CaCl_2_ for N9) in 50 mM Tris‐HCl buffer, pH 8.0, at 37 °C. After addition of purified lactonase (GcL or VmutPPL, 0.2 mg) or cell lysate (N9, 10% v/v), conversions and enantiomeric excesses were monitored at scheduled times.

^a)^ Determined by chiral HPLC using Chiralpak IC or IG column, monitored at 240 nm.

^b)^ Determined by ^1^H‐NMR.

^c)^ Not determined.

The MLL enzyme GcL catalysed the enzymatic kinetic resolution of the thiolactone **1a** with good conversion (64%) after 1 h and excellent and good enantioselectivity for the unreacted thiolactone (>99%) and the acid product **2a** (57%), respectively (Table 1, entry 1). Since the observed conversion (64%) is higher than that expected for an enzymatic kinetic resolution (50% max), it is reasonable to assume that in 1 h GcL hydrolysed both enantiomers of **1a**. Thus, the same reaction was carried out for reduced time. After 10 min, a 51% conversion was observed and the lactone **1a** and the acid **2a** were formed with 88% ee and 84% ee, respectively (Table 1, entry 2). When **1a** was reacted with VmutPLL and N9 lactonases, no conversion was observed and the lactone **1a** was recovered after 1 h as racemate (Table 1, entries 3 and 4). Similarly, GcL was the only lactonase able to deracemise the thiolactone **1b**, providing the acid **2b** with 46% conversion and 86% ee, and the unreacted **1b** with 73% ee (Table 1, entry 6). Blank experiments were carried out with both **1a** and **1b**, showing no conversion when the reactions were carried out in the absence of the enzymes (Table 1, entries 5 and 9). On the other hand, when the lactone **3a**, analogue of **1b**, was treated with the three different enzymes, spontaneous hydrolysis and no enantioselectivity were observed (Table 1, entries 10–13). Only GcL was able to convert almost all the lactone **3a** into the corresponding acid, but **4a** was obtained with low 11% ee (Table 1, entry 10). Clearly, the thiolactone substrate **1b** is less susceptible than its lactone counterpart **3a** to spontaneous hydrolysis under the biocatalytic reaction conditions (Tris‐HCl buffer pH 8.0), thus making the thiolactones better substrates for lactonase biocatalysed deracemisation reactions. Finally, the thiolactone **5a**, bearing a thio‐substituent at C3, was treated with both GcL and N9 enzymes. Lactonase GcL fully converted **5a** into **6a** in only 15 min with no enantioselectivity, while a moderate conversion (36%) and 47% ee of **6a** were observed with N9 after 24 h (Table 1, entries 14 and 15). Importantly, no spontaneous hydrolysis of **5a** under the reaction conditions was observed (Table 1, entry 16). Based on the preliminary screening results, GcL was selected as the preferred enzyme for further biocatalytic deracemisation studies on thiolactone substrates **1** bearing an acyl‐amide group at C3, while the lactonase N9 was further investigated for the deracemisation of thiolactones **5** bearing a thio‐substituent at C3 position.

### Enzymatic Kinetic Resolution of 3‐*N*‐Amide‐γ‐thiolactones 1

The biocatalytic conditions for the enzymatic kinetic resolution of the thiolactone **1a** with GcL enzyme were first optimised. All the reactions were initially carried out for 10 min in 1 mL Tris‐HCl buffer with 30 mM of **1a**, and different parameters, such as pH, temperature, metal cofactor, and biocatalyst loading, were varied. GcL enzyme presents a hetero‐bimetallic active site containing Fe^2+^ and Co^2+^ ions.^[^ ^16^ ^]^ While Fe^2+^ ions were provided by the autoinduction media (see preparation procedure in Supporting Information), the external addition of Co^2+^ ions, in the form of CoCl_2_, was investigated. Initial experiments were carried out by adding CoCl_2_ (1 mM) to the biocatalytic reaction. The results are reported in Table 2. At pH 8.0, 51% conversion of thiolactone **1a** was achieved along with a good *E*‐value of 33 (Table 2, entry 1), while, when the biocatalytic reaction was carried out at lower pH (6.0 and 7.0), only a minimal conversion was observed (Table 2, entries 2 and 3). When the pH was increased from 8 to 9, the *E*‐value decreased from 33 to 24 (Table 2, entry 4), even if the stereoselectivity of the reaction was not affected, and the acid (*S*)‐**2a** was obtained with comparable conversions and ees than those observed at pH 8.0 (52% conv. and 80% ee). The absolute configuration of the thiolactone (*R*)‐**1a** and the corresponding acid (*S*)‐**2a** were determined by comparison of their [*α*]_D_ values with those reported in the literature.^[^ ^76^ ^]^ The decrease of the reaction temperature also did not affect the outcome and the selectivity of the biotransformation (Table 2, entry 5). The enzyme loading was then investigated. A slight decrease or increase of the enzyme loading did not affect substantially the selectivity of the reaction; thus, the ideal GcL loading was found at 21.6 mU mL^−1^ with an *E*‐value of 43 (Table 2, entry 7). Finally, the use of different metal additives was also investigated. Since different lactonase enzymes require different metal cofactors in their active site, we decided to investigate the effect that metal ions, other than Co^2+^, could have on the biocatalytic activity of GcL. The replacement of CoCl_2_ with CaCl_2_ led to a slight improvement of the reaction selectivity providing the acid **2a** with 91% ee and *E*‐value of 59 (Table 2, entry 10), while in the presence of Mn^2+^ a drop in the *E*‐value to 22 and a lower ee for **2a** were observed (Table 2, entry 11). Interestingly, when the biocatalytic reaction was carried out without the addition of any metal additive, the acid **2a** was obtained with excellent 92% ee and 47% conversion, while the unreacted thiolactone **1a** was recovered with 83% ee (Table 2, entry 9). An *E*‐value of 62 was calculated under these reaction conditions. Given that the crystal structure of GcL revealed a hetero‐binuclear iron/cobalt active site,^[^ ^34^ ^]^ it is plausible that the addition of more Co^2+^ to the reaction medium may facilitate the replacement of Fe^2+^, resulting in a more active, but less enantioselective, dicobalt‐substituted enzyme. Metal displacement is widely recognised for its profound impact on the catalytic properties of various metalloenzymes, including lactonases from the PLL^[^ ^75^ ^]^ and metallo‐β‐lactamase superfamilies.^[^ ^12^ ^]^ Finally, a control experiment using an empty pET28a vector *Escherichia coli* BL21(DE3)‐pGro7 cell lysate confirmed that the observed catalytic activity was due to GcL enzyme (Table 2, entry 12).

**Table 2:** Optimisation of reaction conditions of the GcL biocatalysed enzymatic kinetic resolution of 3‐*N*‐hexanoyl‐thiolactone **1a**.

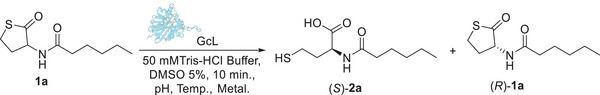								
Entry	pH	Temp. (°C)	Metal cofactor (1 mM)	Enzyme loading (mU mL^−1^)^a)^	Thiolactone (*R*)‐**1a** ee (%)^b)^	Acid (*S*)‐**2a** ee (%)^b)^	Conv. (%)^c)^	*E*‐value^d)^
1	**8**	**37**	**CoCl_2_**	**18**	**88**	**84**	**51**	**33**
2	6	37	CoCl_2_	18	n.d.^e)^	n.d.^e)^	8	n.d.^e)^
3	7	37	CoCl_2_	18	n.d.^e)^	Traces	12	n.d.^e)^
4	9	37	CoCl_2_	18	88	80	52	24
5	8	30	CoCl_2_	18	85	83	50	29
6	8	37	CoCl_2_	16.2	67	88	43	31
7	8	37	CoCl_2_	21.6	81	89	48	43
8	8	37	CoCl_2_	27	90	85	51	38
**9**	**8**	**37**	–	**21.6**	**83**	**92**	**47**	**62**
10	8	37	CaCl_2_	21.6	86	91	49	59
11	8	37	MnCl_2_	21.6	97	69	58	22
12^f)^	8	37	–	–	<1	n.d.^e)^	<1	n.d.^e)^

All reactions were performed in duplicate, with the average values reported (deviation of conversions and ees < 5%).

^a)^ GcL was used as purified enzyme; GcL activity = 0.54 U mL^−1^.

^b)^ The ee were calculated by chiral HPLC using Chiralpak IC or IG column, monitored at 240 nm.

^c)^ The conversions were calculated from the ee values using the following formula: Conversion = ee_s_/(ee_p_ + ee_s_).^[^ ^77^ ^]^

^d)^ The *E*‐value was calculated according to literature using the following formula: *E*‐value = ln[ee_p_(1 − ee_s_)/(ee_p_ + ee_s_)]/ln[ee_p_(1 + ee_s_)/(ee_p_ + ee_s_)].^[^ ^77^ ^]^

^e)^ Not determined.

^f)^ Reaction conducted using the empty pET28a vector *E. coli* BL21(DE3)‐pGro7 cell lysate.

According to the screening and optimisation results (Tables 1 and 2), the GcL biocatalysed hydrolysis of **1a** is fast and the enantioselectivity seems to be strictly dependent on the reaction time. Thus, to gain further insights into this biotransformation, the enzymatic hydrolysis of **1a** was monitored over 120 min by ^1^H‐NMR. As shown in Table 3 and Figure S1, the thiolactone is quickly hydrolysed by GcL and the formation of the acid (*S*)‐**2a** is readily visible by ^1^H‐NMR after a few minutes, increasing over time. The ee of (*S*)‐**2a**, calculated by chiral HPLC, is maximum after 6 min (Table 3, entry 1) and then it decreases over time, while the ee of the unreacted lactone (*R*)‐**1a** gradually increases as the reaction progresses, ultimately reaching 99% after 1 h (Table 3, entry 5). This observation is consistent with a typical enzymatic kinetic resolution (EKR) reaction, which, in this specific case, reaches the ideal conditions (47% conv., 83% ee for (*R*)‐**1a**, 92% ee for (*S*)‐**2a**) after 10 min (Table 3, entry 2). The conversions of the biotransformation were calculated at each time point using the kinetic resolution equation that relates the enantioselectivity to the reaction conversion.^[^ ^78^ ^]^ To confirm the data obtained through the kinetic resolution equation, the conversions were also calculated by ^1^H‐NMR integration of the crude reaction mixtures (Table 3) and they proved to be in perfect agreement with the calculated ones. Additional experiments confirmed that both (*R*)‐**1a** and (*S*)‐**2a** are configurationally stable under the reaction conditions (Scheme S1).

**Table 3:** Time reaction optimisation of the GcL biocatalysed deracemisation of 3‐*N*‐hexanoyl‐thiolactone **1a**.

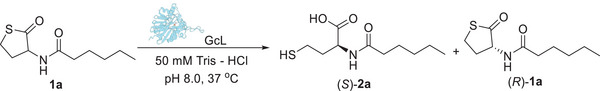					
Entry	Time (min)	Thiolactone (*R*)‐**1a** ee (%)^a)^	Acid (*S*)‐**2a** ee (%)^a)^	Calculated conv. (%)^b)^	^1^H‐NMR conv. (%)^c)^
1	6	57	94	38	36
**2**	**10**	**83**	**92**	**47**	**47**
3	20	96	86	53	52
4	30	97	80	55	55
5	60	99	64	61	62
6	120	99	45	70	74

All reactions were performed in duplicate, with the average values reported (deviation of conversions and ees < 5%).

^a)^ The ee were calculated by chiral HPLC using Chiralpak IC or IG column, monitored at 240 nm.

^b)^ The conversions were calculated from the ee values using the following formula: Conversion = ee_s_/(ee_p_ + ee_s_).^[^ ^77^ ^]^

^c)^ The conversions were calculated by ^1^H‐NMR integration of the crude mixture.

With the optimised conditions established, the substrate scope of the EKR of thiolactone substrates **1a–m** was then explored (Table 4). The conversion and the rate of the biocatalytic transformation were affected by the amide substituent structure and bulkiness. While the thiolactone **1a** bearing a *n*‐hexanoylamide group at C3 is selectively hydrolysed by GcL biocatalyst in only 10 min (Table 4, entry 1), the thiolactones **1c–g** bearing bulkier and cyclic aliphatic amide groups require longer reaction time to reach the optimal 50% conversion of an EKR reaction (Table 4, entries 3–7).

**Table 4:** Substrate scope of the GcL biocatalysed EKR of 3‐*N*‐amide‐thiolactones **1a–m**.

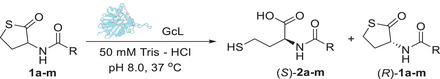							
Entry	Cmpd.	*R*	Time	Thiolactone (*R*)‐**1** ee (%)^a)^	Acid (*S*)‐**2** ee (%)^a)^	Conv. (*S*)‐**2** ee (%)^b)^	*E*‐Value^c)^
1	**1a**	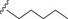	10 min	83	92	47	62
2	**1b**	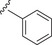	1 h	92	90	51	74
3	**1c**	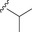	1 h	94	90	51	67
4	**1d**	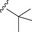	3 h	97	94	51	136
5	**1e**	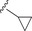	5 h	64	65	50	9
6	**1f**	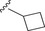	3 h	76	68	53	12
7	**1g**	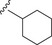	5 h	78	96	45	117
8	**1h**	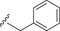	20 min	94	79	54^d)^	30
9	**1i**	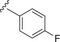	2 h	90	94	49	100
10	**1j**	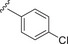	24 h	71	95	43^d)^	83
11	**1k**	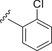	24 h	88	94	48^d)^	94
12	**1l**	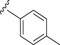	24 h	64	93	41	53
13	**1m**	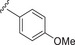	24 h	19	70	21	7

All the experiments were carried out on 0.03 mmol of thiolactone substrate **1**; GcL was used as purified enzyme (GcL activity = 0.54 U mL^−1^). All reactions were performed in duplicate, with the average values reported (deviation of conversions and ees < 5%).

^a)^ The ee were calculated by chiral HPLC using Chiralpak IC or IG column, monitored at 240 nm.

^b)^ The conversions were calculated from the ee values using the following formula: Conversion = ee_s_/(ee_p_ + ee_s_).^[^ ^77^ ^]^

^c)^ The *E*‐values were calculated according to literature using the following formula: *E*‐value = ln[ee_p_(1 − ee_s_)/(ee_p_ + ee_s_)]/ln[ee_p_(1 + ee_s_)/(ee_p_ + ee_s_)].^[^ ^77^ ^]^

^d)^ The conversions were calculated by HPLC using Chiralpak IC or IG column, monitored at 240 nm.

The thiolactone **1c** bearing a *i*‐propanamide group at C3 is deracemised in 1 h, providing the acid (*S*)‐**2c** and the unreacted lactone (*R*)‐**1c** with high ees (90% and 94%, respectively, Table 4, entry 3). The bulkier *tert‐*butylamide derivative **1d** was deracemised in 3 h, giving the acid (*S*)‐**2d** in 94% ee and the unreacted (*R*)‐**1d** with excellent 97% ee and an *E*‐value of 136 (Table 4, entry 4), while the cyclopropylamide thiolactone **1e** required longer reaction time (5 h) and the enantiomers (*S*)‐**2e** and (*R*)‐**1e** were obtained only in moderate ees and a low *E*‐value of 9 (Table 4, entry 5). Similarly, the substrate **1f** bearing a cyclobutane amide moiety reached 53% conversion after 3 h and (*S*)‐**2f** and (*R*)‐**1f** were obtained with moderate ees and *E*‐value of 12 (Table 4, entry 6). Interestingly, a linear correlation between the increase of the carbocyclic ring size from cyclopropyl in **1e** to cyclohexane in **1g** was observed. In particular, the thiolactone **1g** was deracemised in 5 h providing the acid (*S*)‐**2g** with an excellent 96% ee (*E*‐value = 117, Table 4, entry 7). The thiolactone **1h** bearing a benzylamide group at C3 was hydrolysed by GcL in only 20 min. However, while the unreacted (*R*)‐**1h** was recovered with high 94% ee, a lower ee (79%) was observed for the acid (*S*)‐**2h**. The thiolactones **1b** and **1i–m**, bearing an arylamide substituent at C3 generally required long reaction times (up to 24 h) to achieve ∼50% conversion (Table 4, entries 2 and 9–13). The unsubstituted benzoylamide thiolactone **1b** reached 51% conversion in 1 h, affording (*R*)‐**1b** and (*S*)‐**2b** with excellent ees of 92% and 90% respectively, along with an excellent *E*‐value of 74 (Table 4, entry 2). On the other hand, the presence of different substituents on the arylamide moiety affected the rate and selectivity of the EKR. The fluorine derivative **1i** was deracemised in 2 h providing (*S*)‐**2i** with 94% ee and (*R*)‐**1i** with 90% ee, and a high *E*‐value of 100 (Table 4, entry 9). Longer reaction times (24 h) were required for the deracemisation of **1j** and **1k** bearing a chlorine substituent on the aryl ring. In both cases, the acids (*S*)‐**2j** and (*S*)‐**2k** were obtained with excellent ees (95% and 94%, respectively), while slightly lower ees were recorded for the unreacted lactones (Table 4, entries 10 and 11). Finally, the replacement of the halogen substituents with a methyl or a methoxy group in the thiolactones **1l** and **1m** affected the GcL biocatalytic activity and selectivity. The acid (*S*)‐**2l** was obtained after 24 h with an excellent 93% ee, but the biotransformation showed only an *E*‐value of 53, due to the lower optical purity of (*R*)‐**1l** (Table 4, entry 12). Poor conversion and low ees were observed for **1m** (Table 4, entry 13), suggesting that both steric and electronic factors may influence the hydrolytic activity and selectivity of GcL biocatalyst.

**Figure 2:**
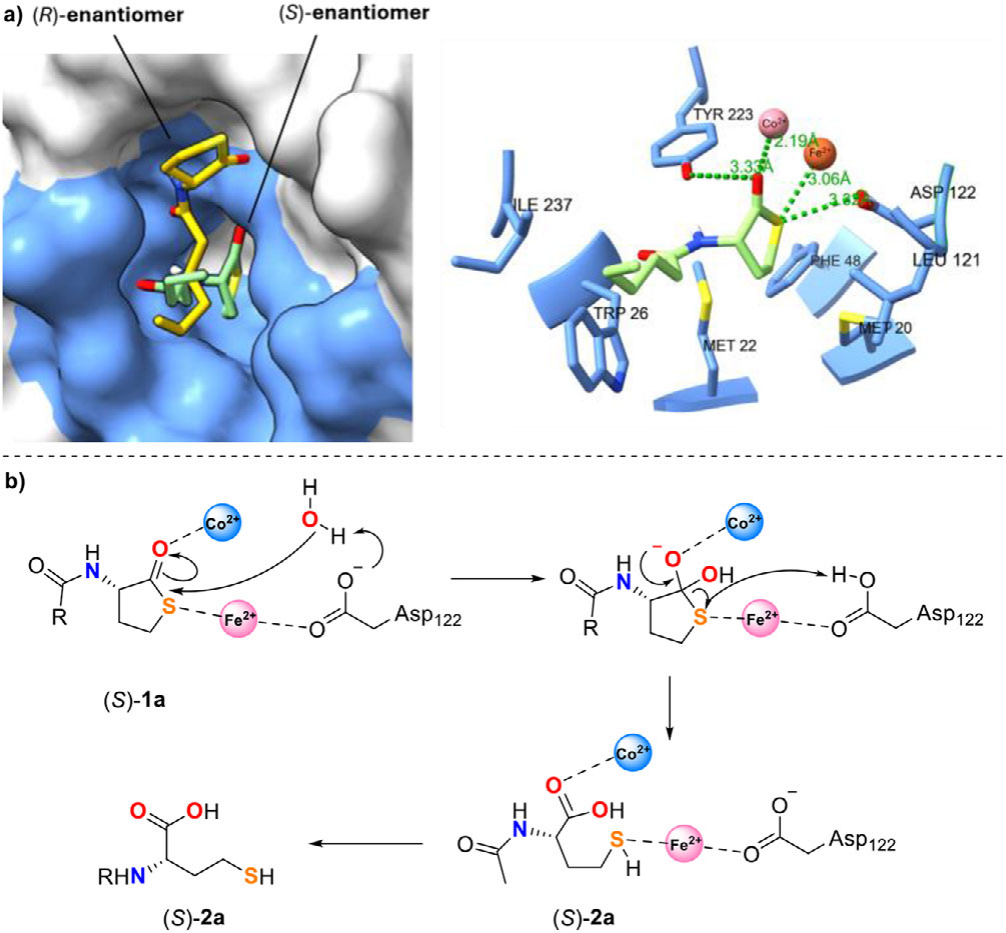
a) Docking of (*R*)‐**1a** (yellow) and (*S*)‐**1a** (green) enantiomers in the GcL catalytic site and interaction of (*S*)‐**1a** with metal cofactors and amino acid residues; b) proposed mechanism for the GcL biocatalysed EKR of thiolactones **1**.^[^ ^16^, ^79^ ^]^ Additional information on docking analysis is provided in Table S10.

In silico docking studies were carried out to rationalise the observed enantioselectivity of GcL towards the thiolactone **1a** enantiomers. As shown in Figure 2a, the active site of GcL (PDB: 6N9Q) is organised around two metal cations, Co^2+^ and Fe^2+^, which bind, respectively, the carbonyl group and the sulphur atom of the thiolactone (*S*)‐**1a**. Further coordination to the sulphur atom is provided by the Asp_122_ residue, while the Tyr_223_ provides additional binding to the carbonyl group and to the amide NH moiety. A proposed mechanism for the GcL biocatalysed hydrolysis of the thiolactone **1a** is shown in Figure 2b.^[^ ^16^, ^79^ ^]^ The Co^2+^ and Fe^2+^ ion activate the thiolactone ring through coordination with the sulphur atom and the carbonyl group of **1a**, in turn promoting the attack of a molecule of H_2_O and the following thiolactone hydrolysis. Docking studies revealed that the enantiomer (*R*)‐**1a** was not able to bind to the metal cofactors and to the Asp_122_ and Tyr_223_ residues in a similar pose than (*S*)‐**1a**, thus explaining the observed enantiopreference and selectivity of GcL towards the hydrolysis of the (*S*)‐**1** enantiomers.

### Engineering N9 Enzyme: Dynamic Kinetic Resolution of 3‐Alkyl/Arylthio‐γ‐thiolactones 5

**Figure 3:**
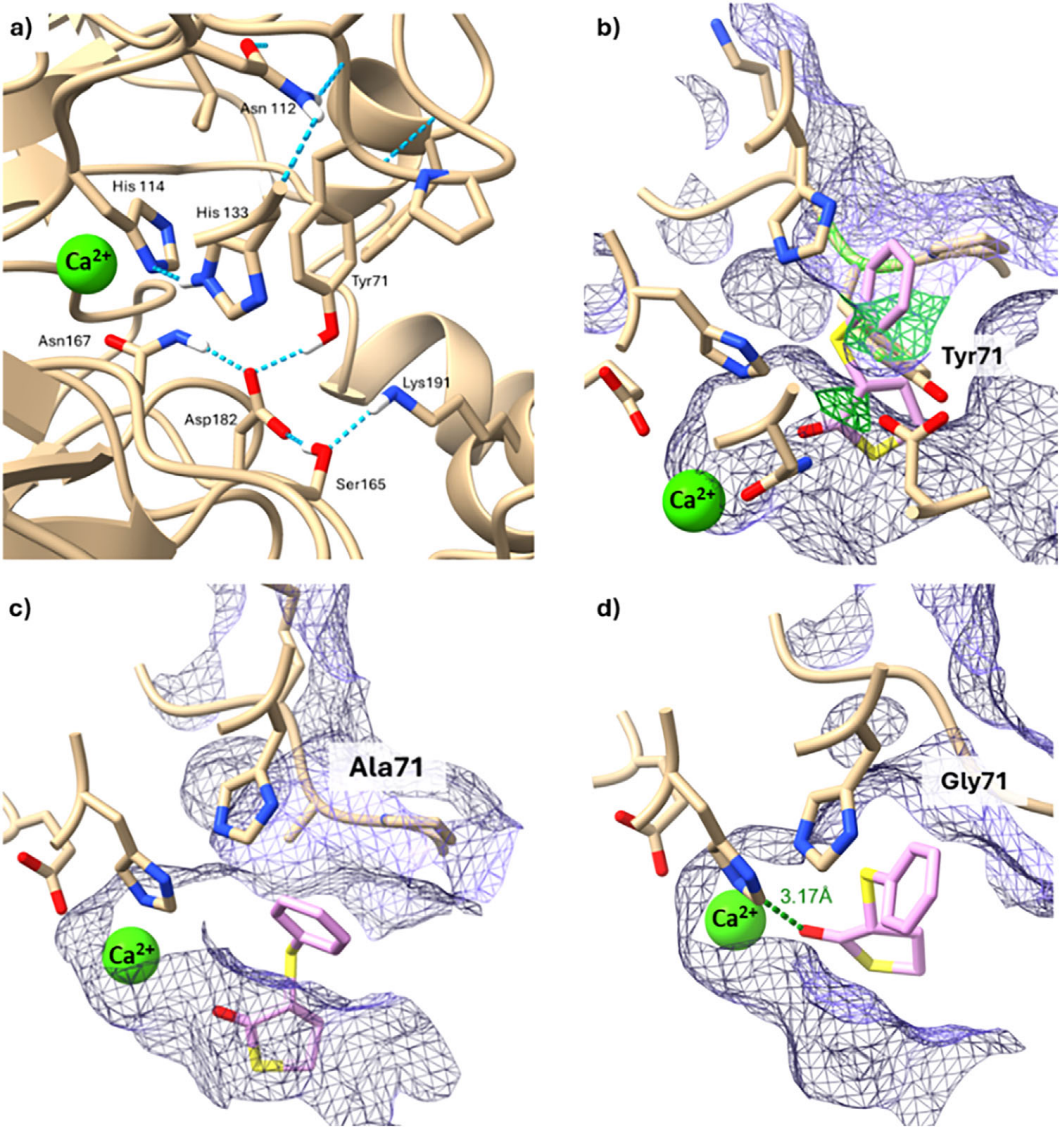
a) Catalytic pocket of N9. b) Docking of thiolactone (*R*)‐**5a** (pink) into N9 catalytic pocket showing the steric hindrance of the residue Tyr_71_ (Y71). The surface area of Tyr_71_ is highlighted in green. c) Docking of thiolactone (*R*)‐**5a** (pink) into N9 Y71A mutant catalytic pocket. d) Docking of thiolactone (*R*)‐**5a** (pink) into N9 Y71G mutant catalytic pocket.

In preliminary screening, enzyme N9 was found to catalyse the selective hydrolysis of **5a** affording the γ‐thio‐α‐thiocarboxylic acid (*R*)‐**6a** in low conversion (36%) and moderate ee (47%). The unreacted thiolactone **5a** was recovered as racemate from the reaction mixture (Table 1, entry 15). Due to promising preliminary data, we decided to further investigate the enzyme N9 and to carry out mutagenesis studies to improve its selectivity and biocatalytic activity. The 3D structure of N9 was predicted using AlphaFold2 starting from its protein sequence (see Supporting Information). Consistent with the known structure of PON1,^[^ ^8^ ^]^ the active site of N9 includes a catalytic calcium ion and a hydrophobic tunnel formed by various amino acid residues involved in a hydrogen‐bonding network, as shown in Figure 3a. While the residues His_114_ and His_133_ seem to play an active role in the lactonase catalytic mechanism,^[^ ^32^, ^80^, ^81^, ^82^ ^]^ the residues Tyr_71_, Asn_167_, and Asp_182_ are present at the entrance of the tunnel, but they do not have any direct involvement in the thiolactone hydrolysis. Thus, Tyr_71_, Asn_167_, and Asp_182_ were selected for alanine scanning mutagenesis experiments.

Firstly, the N9 variants Y71A, N167A, and D182A were prepared as cell lysates and tested as biocatalysts for the hydrolysis of thiolactone **5a**. The N167A and D182A mutants displayed reduced activity compared to N9, affording the acid (*R*)‐**6a** in lower conversions (9% and 7%) and ees (12% and 33%) (Table 5, entries 2 and 3). The absolute configuration of acid product **6a** was assigned through comparison of the [*α*]_D_ value with literature data^[^ ^83^ ^]^ (see Supporting Information and Table S9). Interestingly, during the biocatalytic reactions, the disulphide **7** was formed in variable amounts. However, the addition of dithiobutylamine (DTBA) to the reaction mixture during the workup procedure fully converted the dimer **7** back to (*R*)‐**6a** in situ. No traces of **7** were detected in the crude reaction mixture after work‐up. Remarkably, the Y71A mutant yielded the acid (*R*)‐**6a** with high conversion (74%) and good enantioselectivity (ee 88%) (Table 5, entry 4). This result suggests that Tyr_71_, while not directly involved in the biocatalytic mechanism, plays a significant role in influencing both the enantioselectivity and the overall enzyme activity due to its steric hindrance (Figure 3b,c). Optimisation of the reaction conditions, namely increasing the CaCl_2_ concentration to 4 mM, increasing the pH to 9.0 and reducing the temperature to 30 °C, led to a remarkable improvement of the conversion (>99%) while the ee of (*R*)‐**6a** remained almost constant (89%) (Table 5, entries 5–7).

**Table 5:** Screening of N9 mutants on the thiolactone **5a**.

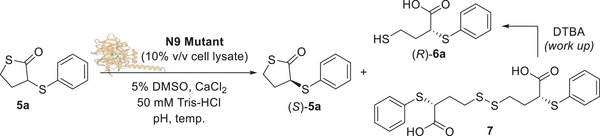								
Entry	Variant	Time (h)	pH	CaCl_2_ (mM)	Temp. (°C)	Conv. (*R*)‐**6a** (%)^a)^	Unreacted (*S*)‐**5a** ee (%)^b)^	Acid (*R*)‐**6a** ee (%)^b)^
1	N9	48	8.0	1	37	36	Racemic	45
2	D182A	24	8.0	1	37	7	Racemic	33
3	N167A	24	8.0	1	37	9	Racemic	12
4	Y71A	24	8.0	1	37	74	Racemic	88
5	Y71A	24	8.0	4	37	83	Racemic	88
6	Y71A	24	9.0	4	37	92	Racemic	88
7	Y71A	24	9.0	4	30	>99	Racemic	89
8	**Y71G**	**24**	9.0	4	30	**>99**	**Racemic**	**97**
9	Y71L	24	9.0	4	30	53	Racemic	78
10	Y71I	24	9.0	4	30	5	Racemic	<1
11	Y71M	24	9.0	4	30	67	Racemic	75
12	Y71W	24	9.0	4	30	9	Racemic	45

All the reactions were carried out with 0.01 mmol of thiolactone **5a** substrate. All reactions were performed in duplicate, with the average values reported (deviation of conversions and ees < 5%).

^a)^ The conversions were calculated by chiral HPLC using Chiralpak IC or IG column, monitored at 240 nm. The conversions were calculated using the following equation: conv% = [HPLC area of the acid product/(HPLC areas of acid product + unreacted lactone)] x 100.

^b)^ The ees were calculated by chiral HPLC using Chiralpak IC or IG column, monitored at 240 nm.

**Scheme 1:**
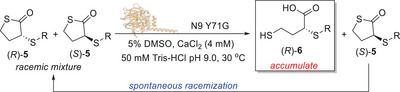
Mechanism of the DKR of 3‐thiosubstituted thiolactones **5**.

With the aim of further improving the selectivity of the biocatalytic reaction, a series of additional N9 mutants with different residues at position 71 were then prepared and tested on thiolactone **5a**. The general trend observed was that a decrease in the van der Waals volume of the residue at position 71 led to an increase in both (*R*)‐**6a** ee and conversion. The bulky mutant Y71W gave the product (*R*)‐**6a** with only 9% conversion and 45% ee (Table 5, entry 12), while the mutants Y71L and Y71M, bearing smaller residues at position 71 provided (*R*)‐**6a** with higher conversions (53% and 67%) and ee values (78% and 75%), respectively (Table 5, entries 9 and 11). The mutant Y71I with a branched isoleucine residue at position 71 yielded (*R*)‐**6a** with only 5% conversion and negligible ee (Table 5, entry 10), while, remarkably, the mutant Y71G bearing a small glycine residue at position 71 (Figure 3d) provided the acid (*R*)‐**6a** with >99% conversion and an excellent 97% ee (Table 5, entry 8), clearly indicating a strict correlation between the bulkiness of the residue 71 and the stereoselectivity and activity of N9 biocatalyst. Interestingly, all the biocatalytic transformations carried out with either N9 or any of the above‐mentioned mutants always led to the recovery of the thiolactone **5a** as a racemate, even if in variable amounts. Moreover, the best mutant N9 Y71G led to enantioenriched (*R*)‐**6a** with excellent ee (97%) and full conversion (>99%). Such data are not compatible with a standard EKR reaction, while they point towards a DKR of **5a** as shown in Scheme 1. Previous studies from our group showed that stereocentres bearing a C─S bond at the α position to an electron withdrawing group may undergo rapid racemisation under basic conditions.^[^ ^54^, ^55^, ^56^, ^57^, ^58^, ^59^ ^]^ It was thus assumed that under the biocatalytic reaction conditions (pH 9.0), the N9 Y71G mutant selectively hydrolyses the (*R*)‐**5a** enantiomer into the acid (*R*)‐**6a** while the unreacted (*S*)‐**5a** enantiomer undergoes rapid racemisation leading back to racemic **5a**. The latter is then further hydrolysed by N9 Y71G in a DKR process, eventually leading to the accumulation of enantioenriched acid (*R*)‐**6a** (Scheme 1).

**Scheme 2:**
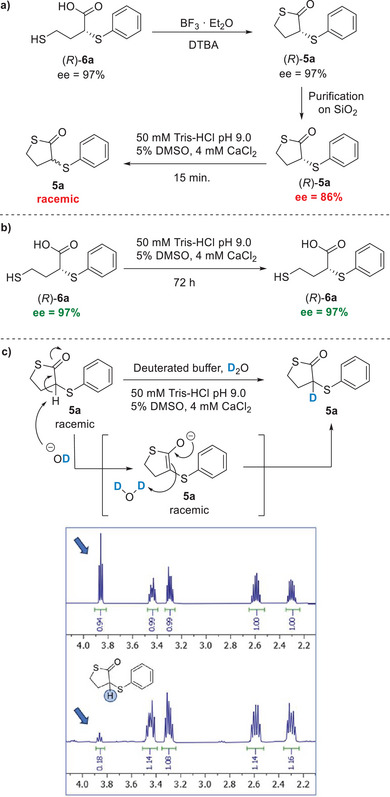
a) Racemisation experiment of enantioenriched thiolactone (*R*)‐**5a** in Tris‐HCl buffer, pH 9.0. b) Racemisation experiments of enantioenriched acid (*R*)‐**6a** in Tris‐HCl buffer, pH 9.0. c) Deuterium labelling experiment on thiolactone (*R*)‐**5a**. ^1^H‐NMR spectra of the thiolactone (*R*)‐**5a** before (above) and after (below) suspension in deuterated buffer.

In order to confirm this hypothesis, a set of racemisation and labelling experiments was carried out (Scheme 2). The enantioenriched (*R*)‐**6a** (ee 97%) was converted to the thiolactone (*R*)‐**5a** with BF_3_·Et_2_O and in the presence of DTBA, to avoid the formation of disulphide byproduct **7** (Scheme 2a). The purified thiolactone (*R*)‐**5a** was obtained with 86% ee since a partial epimerisation occurred during the purification process on silica gel. Then, (*R*)‐**5a** was suspended in the biocatalytic reaction media, namely Tris‐HCl buffer, pH 9.0, containing 5% DMSO and 4 mM CaCl_2_. After only 15 min, **5a** was recovered as a racemate, thus confirming that a spontaneous racemisation of the unreacted **5a** occurs during the biotransformation allowing the DKR of the chiral thiolactone (Scheme 2a). On the other hand, when the enantioenriched acid (*R*)‐**6a** was suspended in buffer at pH 9.0, no racemisation was observed and (*R*)‐**6a** was recovered with 97% ee after 72 h (Scheme 2b). Finally, a labelling experiment was carried out by suspending racemic **5a** in deuterated buffer at pH 9.0. After 15 min, the thiolactone **5a** was recovered partially deuterated at C3, as shown by ^1^H‐NMR analysis (Scheme 2c), suggesting that its racemisation may be due to a keto‐enol equilibrium favoured by the basic conditions of the biotransformation medium.

Once the DKR mechanism was confirmed, the reaction conditions of the biocatalytic deracemisation of **5a** with N9 Y71G mutant were further optimised (Table 6). While the reduction of the enzyme (cell lysate) loading from 10% to 5% v/v did not affect the ee and conversion of (*R*)‐**6a** (97% and >99%, respectively, Table 6, entries 2 and 3), a further reduction to 1% v/v led to a decrease of the ee and conversion to 94% and 94%, respectively (Table 6, entry 1). At higher enzyme loading (20% v/v), the conversion of (*R*)‐**6a** remained >99%, but the ee decreased to 95% (Table 6, entry 4). Therefore, the enzyme loading was fixed at 5% v/v for subsequent experiments. When the CaCl_2_ concentration was reduced from 4 to 2 mM, the ee of (*R*)‐**6a** slightly decreased to 96% (Table 6, entry 5). Conversely, increasing CaCl_2_ to 8 mM also resulted in a similar ee reduction to 96%, but a slightly lower conversion of 98% was observed (Table 6, entry 6). Finally, lowering the reaction pH from 9.0 to 8.0 caused the reduction of the ee of (*R*)‐**6a** to 95% (Table 6, entry 7) as well as when the biotransformation was carried out at pH 7.5 the acid (*R*)‐**6a** was obtained with a conversion of 97% and an ee of 95% (Table 6, entry 8). Under more acidic conditions (pH 5.5), the reaction conversion significantly dropped to 31%, though the ee of (*R*)‐**6a** remained high at 97% (Table 6, entry 9). Finally, a negative control experiment using cell lysate of *E. coli* Rosetta (DE3) cells carrying an empty pET28a vector was carried out under the optimised conditions (enzyme 5% v/v, 4 mM CaCl_2_, pH 9.0). No reaction was observed (Table 6, entry 10), confirming that the stereoselective hydrolysis of **5a** was catalysed by N9 Y71G.

**Table 6:** Optimisation of the reaction conditions of the N9 Y71G catalysed DKR of **5a**.

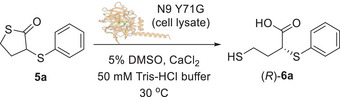					
Entry	N9 Y71G (% v/v)	CaCl_2_ (mM)	pH	Conv. (%)^a)^	Acid ee (%)^b)^
1	1	4	9	94	94
**2**	**5**	**4**	**9**	**>99**	**97**
3	10	4	9	>99	97
4	20	4	9	>99	95
5	5	2	9	>99	96
6	5	8	9	98	96
7	5	4	8	>99	95
8	5	4	7.5	97	95
9	5	4	5.5	31	97
10^c)^	5	4	9	<1	<1

All the reactions were carried out with 0.01 mmol of thiolactone **5a** substrate. All reactions were performed in duplicate, with the average values reported (deviation of conversions and ees < 5%).

^a)^ The conversions were calculated by chiral HPLC using Chiralpak IG column, monitored at 240 nm. The conversions were calculated using the following equation: conv% = [HPLC area of the acid product/(HPLC areas of acid product + unreacted lactone)] x 100.

^b)^ The ees were calculated by chiral HPLC using Chiralpak IG column, monitored at 240 nm.

^c)^ Empty pET28a vector *E. coli* Rosetta (DE3) cell lysate was used.

With the optimised reaction conditions in hand, the substrate scope of N9 Y71G in the DKR of 3‐thiosubstituted‐γ‐thiolactones **5a‐p** was finally explored (Table 7). In general, all the thiocarboxylic acids (*R*)‐**6a‐j** bearing an arylthiol substituent at C3 were obtained with excellent conversions (up to >99%) and ee (up to >99%) (Table 7, entries 1–10). The acids (*R*)‐**6c‐e** bearing arylthiol substituents at *para*‐position were obtained with excellent enantioselectivities (97% ee, Table 7, entries 3–5), with the only exception of the *p*‐Cl‐Ph derivative (*R*)‐**6b** which, however, was still formed with a remarkable 95% ee (Table 7, entry 2). However, the bulkier substrate **5c** bearing a *p*‐Br‐Ph‐thiol moiety required a longer reaction time (72 h) to achieve a high conversion (94%, Table 7, entry 3). The need for extended reaction time suggests that the steric hindrance of the bromo substituent slows down the biocatalytic hydrolysis, however without compromising the enantioselectivity. Interestingly, *ortho*‐halogen‐substituted thiophenyl groups exhibited a clear trend in enantioselectivity. The fluorine‐substituted acid (*R*)‐**6f** was obtained with excellent ee (>99%) and conversion (>99%) (Table 7, entry 6), while a decrease in enantioselectivity was observed with the bulkier and less electronegative chlorine derivative (*R*)‐**6g** (95% ee, Table 7, entry 7) and the bromine derivative (*R*)‐**6h** (92% ee, entry 8).

**Table 7:** Substrate scope of the N9 Y71G biocatalysed DKR of 3‐SR‐thiolactones **5a‐p**.

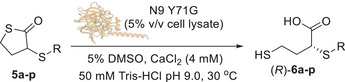					
Entry	Cmpd.	*R*	Time (h)	Conv. (yield) (%)a),b)	(*R*)‐**6** ee (%)^c)^
1	**5a**	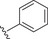	24	>99 (90)^d)^	97^d)^
2	**5b**	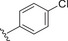	24	>99 (92)	95
3	**5c**	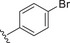	72	94 (83)	97
4	**5d**	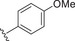	24	99 (91)	97
5	**5e**	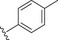	24	>99 (89)	97
6	**5f**	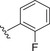	24	>99 (94)	>99
7	**5g**	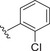	24	>99 (92)	95
8	**5h**	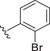	48	90 (81)	92
9	**5i**	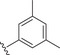	48	95 (86)	97
10	**5j**	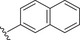	72	95 (85)	93
11	**5k**	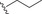	24	84 (72)	87
12	**5l**	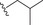	24	87 (80)	90
13	**5m**	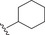	48	93 (82)	87
14	**5n**	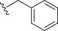	48	97 (89)	90
15	**5o**	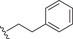	72	36 (32)	90
16	**5p**	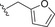	24	99 (89)	80

All the reactions were carried out with 0.05 mmol of thiolactone **5** substrates. N9 Y71G was used as cell lysate (N9 Y71G lysate activity = 2 U mL^−1^). All reactions were performed in duplicate, with the average values reported (deviation of conversions and ees < 5%).

^a)^ The conversions were calculated by chiral HPLC using Chiralpak IG or Chiralcel OJ‐H columns, monitored at 240 nm. The conversions were calculated using the following equation: conv% = [HPLC area of the acid product/(HPLC areas of acid product + unreacted lactone)] x 100.

^b)^ Isolated yields are reported in brackets.

^c)^ The ees were calculated by chiral HPLC using Chiralpak IG or Chiralcel OJ‐H columns, monitored at 240 nm.

^d)^ When the reaction was carried out at preparative scale (1 mmol of **5a**), the acid (*R*)‐**6a** was obtained with 86% isolated yield and 97% ee.

Whilst (*R*)‐**6g** was still obtained with excellent conversion (>99%), a longer reaction time (48 h) was required for (*R*)‐**6h** to achieve 90% conversion, suggesting a more pronounced steric effect of the bromine group on both the selectivity and the reaction rate. High enantioselectivity was observed for the dimethyl‐substituted substrate **5i**, which was hydrolysed into (*R*)‐**6i** with 97% ee and 95% conversion after 48 h (Table 7, entry 9). Similarly, the bulky 2‐naphthyl‐substituted thiolactone **5j** was converted to the corresponding acid (*R*)‐**6j** with 93% ee and 95% conversion after 72 h (Table 7, entry 10). Such results highlight the versatility of N9 Y71G in accommodating and hydrolysing thiolactone substrates bearing bulky aromatic systems. Thiolactones **5k**–**p** bearing an alkylthio‐substituent at C3 were then investigated, showing, as a general trend, to be good substrates of N9 Y71G. The propyl‐substituted acid (*R*)‐**6k** was obtained with good 87% ee and 84% conversion (Table 7, entry 11), similarly to the isobutyl‐substituted acid (*R*)‐**6l**, which was recovered with 90% ee and 87% conversion (Table 7, entry 12). The cyclohexyl‐substituted substrate **5m** required a longer reaction time of 72 h to reach 93% conversion, and it was obtained with good 87% ee (Table 7, entry 13). Remarkably, the benzylthio‐substituted acid (*R*)‐**6n** and the phenylethylthio derivative (*R*)‐**6o** were obtained with high ee (90%, Table 7, entries 14 and 15). While (*R*)‐**6n** was obtained with excellent 97% conversion after 48 h (Table 7, entry 14), the bulkier (*R*)‐**6o** was obtained with only 36% conversion after 72 h (Table 7, entry 15). Finally, the furan‐substituted thiolactone **5p** was converted into (*R*)‐**6p** with 80% ee and an excellent 99% conversion after 24 h (Table 7, entry 16).

**Scheme 3:**
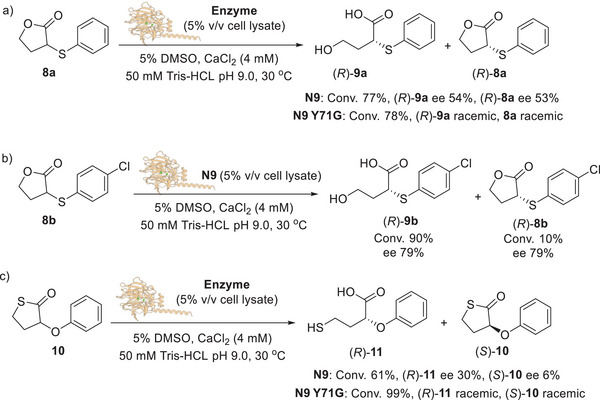
a) Biocatalytic hydrolysis of lactone **8a** with N9 and N9 Y71G lactonases. b) Biocatalytic hydrolysis of lactone **8b** with N9 lactonase. c) Biocatalytic hydrolysis of 3‐phenoxy‐thiolactone **10** with N9 and N9 Y71G lactonases.

Intrigued by the excellent data observed in the DKR of 3‐thio‐substituted thiolactones **5a**–**p**, we also decided to investigate the hydrolysis of the lactones **8a‐b** bearing an arylthiol‐substituent at C3 as well as the thiolactone **10** bearing a phenoxy moiety at C3, with both N9 and N9 Y71G biocatalysts (Scheme 3).^[^ ^84^ ^]^

**Scheme 4:**
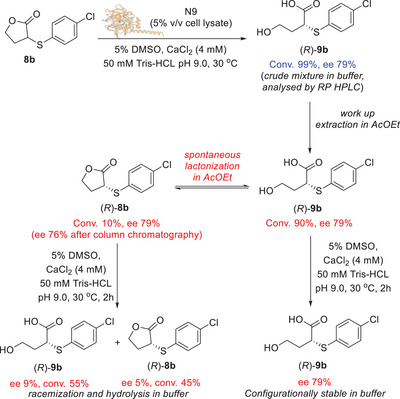
Lactonisation of (*R*)‐**9b** and configurational stability experiments.

Unlike the thiolactones, the N9 Y71G mutant showed negligible enantioselectivity towards lactone **8a**, yielding (*R*)‐**9a** as a racemate with 78% conversion (Scheme 3a). On the other hand, the wild‐type biocatalyst N9 afforded (*R*)‐**9a** with 54% ee and 77% conversion. Surprisingly, the enantioenriched lactone (*R*)‐**8a**, showing the same absolute configuration of the acid (*R*)‐**9a**, was also recovered from the reaction mixture with 53% ee. Similarly, the lactone **8b** was hydrolysed by N9 yielding the acid (*R*)‐**9b** with 79% ee and 90% conversion, together with the lactone (*R*)‐**8b** with 10% conversion and 79% ee (Scheme 3b). It was hypothesised that such results could be due to the spontaneous lactonisation of the acid (*R*)‐**9b** into the lactone (*R*)**‐8b** under the reaction/work‐up conditions. Thus, additional experiments were carried out on lactone **8b** to confirm such hypothesis (Scheme 4). The racemic **8b** was hydrolysed with N9 as biocatalyst over 48 h after which time the reaction was completed.

The crude reaction mixture was analysed through reversed phase (RP) HPLC showing the formation of the acid (*R*)‐**9b** only (>99% conversion), while no traces of the lactone (*R*)‐**8b** were detected. Interestingly, the HPLC analysis (normal phase, NP) of the reaction mixture after workup and extraction with EtOAc, revealed the presence of the lactone (*R*)‐**8b** (10% conversion) together with (*R*)‐**9b** (90% conversion). Both (*R*)‐**8b** and (*R*)‐**9b** maintained the same ee of 79%. It is evident that a spontaneous lactonisation of (*R*)‐**9b** into (*R*)‐**8b** occurred during the extraction process in EtOAc (Schemes S2 and S3). Racemisation tests conducted on the enantioenriched lactone (*R*)‐**8b** and (*R*)‐**9b** also revealed that, while the acid (*R*)‐**9b** is configurationally stable in the reaction buffer, the optical purity of the lactone (*R*)‐**8b** drops from 79% to 5% within 2 h. In addition, lactone (*R*)‐**8b** spontaneously hydrolyses in buffer conditions providing the acid (*R*)‐**9b** with 55% conversion. Such observation suggests that N9 seems able to catalyse the DKR of lactone **8b**, but the spontaneous hydrolysis of **8b** occurring in the reaction media may account for the observed moderate enantioselectivity. Finally, the N9 and N9 Y71G biocatalysts were reacted with the thiolactone **10** bearing a phenoxy substituent at C3 (Scheme 3c). Both enzymes promoted the hydrolysis of **10** with high to excellent conversions (61% and 99%, respectively). However, in both cases, poor enantioselectivities were observed.

**Figure 4:**
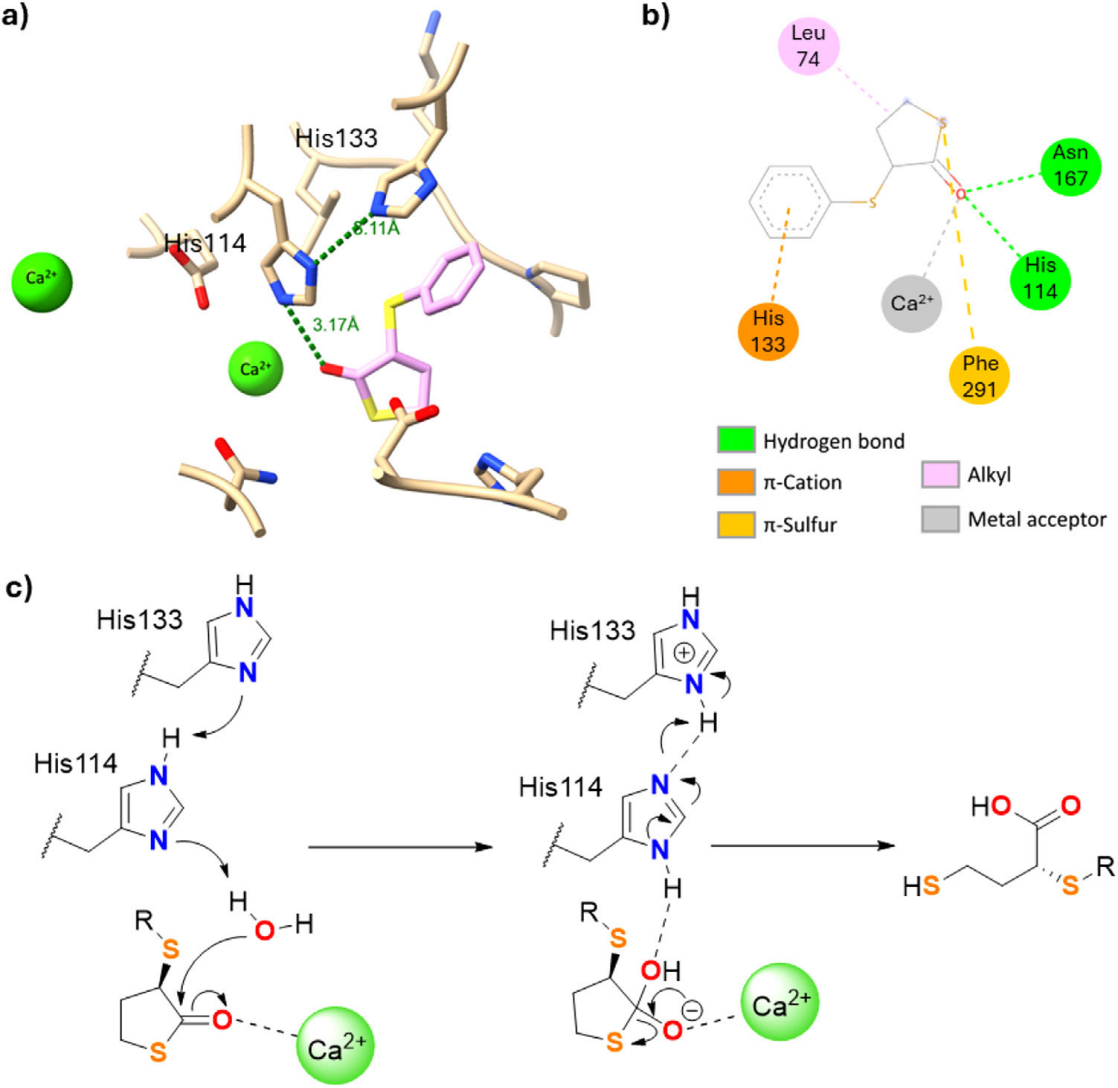
a) Coordination of the carbonyl moiety of (*R*)‐**5a** with His_144_ and coordination of His_144_ with His_133_. b) Amino acid residues of the N9 Y71G catalytic pocket interacting with (*R*)‐**5a**. c) Proposed mechanism of action of N9 Y71G.

Additional docking studies were carried out with the model substrate **5a**, with the aim to gain further insights into the mechanism of action of the biocatalyst N9 Y71G. As shown in Figure 4a,b, the carbonyl group of (*R*)‐**5a** is positioned near one of the catalytic Ca^2+^ ion in the N9 Y71G catalytic pocket and interacts with the residues His_114_, Asn_167_, and Phe_291_. The Ca^2+^ ion is further stabilised through coordination with the residues Asn_167_, Asn_223_, Asn_269_, Asp_268_, Glu_53_, and His_114_ (Figure S3). According to previous reports on the PON1 catalysed hydrolysis of lactones,^[^ ^80^, ^85^ ^]^ and on the basis of docking studies, a reaction mechanism for N9 Y71G is proposed in Figure 4c. The residue His_114_, activated through H‐bonding by His_133_, deprotonates a molecule of water which then attacks the carbonyl moiety of **5a**, in turn activated by the coordination with Ca^2+^, leading to ring opening and formation of (*R*)‐**6a**. The key role of the residues His_133_ in the activation of His_114_ and Asn_167_ in the coordination and stabilisation of the thiolactones **5** in the catalytic pocket was confirmed by further mutagenesis experiments. The mutants N9 Y71G/H133A and N9 Y71G/N167A were prepared and reacted with thiolactone **5a**.^[^ ^86^ ^]^ In both cases, no conversion of **5a** into (*R*)‐**6a** was detected and **5a** was recovered as a racemate, confirming their crucial role in the biocatalytic hydrolysis (Table S6).

**Figure 5:**
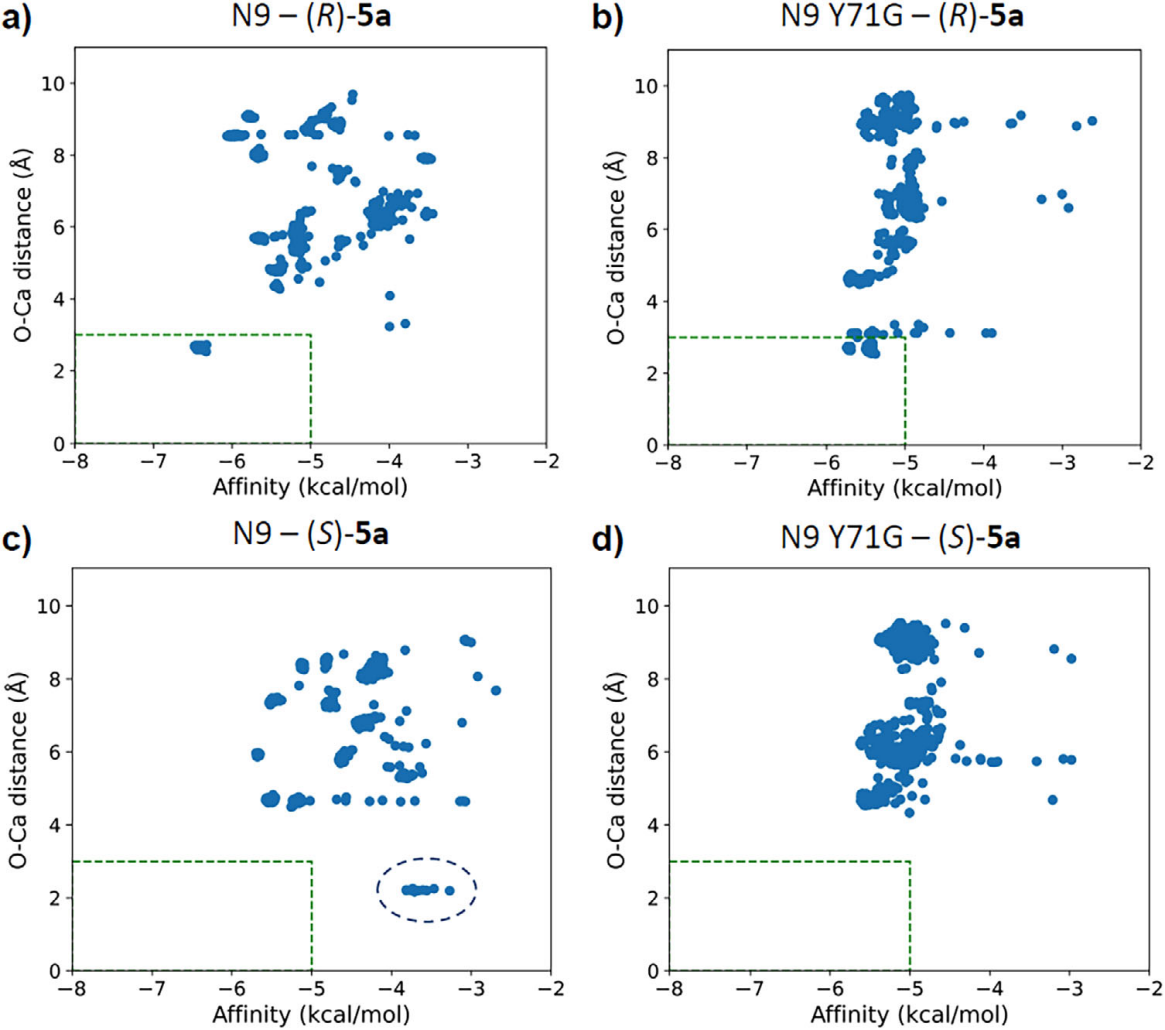
Plotted clusters of (*R*)‐**5a** and (*S*)‐**5a** docking results into N9 and N9 Y71G modelled structures. a) and b) in both N9 and N9 Y71G, there are docking pose clusters of (*R*)‐**5a** falling into the dash‐lined box that highlights the clusters that satisfy the affinity score and distance criteria, based on the catalytic orientation of the substrate in the vicinity of the catalytic Ca^2+^ ion. c) and d) no clusters of (*S*)‐**5a** docked into N9 or N9 Y71G fell into the selected box; however, some low‐scoring poses (indicated by dashed circle) with the ideal O–Ca distance and orientation were seen in N9.

Docking studies on N9 and N9 Y71G were performed to rationalise the increased selectivity of the latter towards the enantiomer (*R*)‐**5a**. For both (*R*)‐**5a** and (*S*)**‐5a**, 200 docking runs were performed on N9 and N9 Y71G. The results were clustered based on O–Ca distance, with a cutoff of 3 Å considered ideal for effective interaction and polarisation of the carbonyl bond. This distance criterion was chosen to distinguish productive substrate orientations, as efficient biocatalytic activity in this system depends on the close proximity between the Ca^2+^ ion and the carbonyl oxygen of the substrate. The results were plotted (Figure 5), reporting the enzyme‐substrate affinity on the horizontal axis and the O–Ca distance on the vertical axis, highlighting differences in selectivity between N9 and N9 Y71G. Both N9 and N9 Y71G proved to be (*R*)‐selective with observed clusters complying with the distance (<3 Å) and affinity scores (−5 kcal mol^−1^) thresholds. However, in N9 Y71G, no (*S*)‐clusters fell within a 3 Å distance, and the highest‐scoring poses adopted unproductive conformations due to the substrate's opposite orientation relative to the catalytic Ca^2+^ ion. In contrast, N9 allowed some (*S*)‐conformations to meet the distance criteria, albeit with lower affinity scores than (*R*)‐conformations. Such data are in agreement with the low (*R*)‐stereoselectivity observed in the hydrolysis of **5a** with N9 (ee 45%, Table 5, entry 1) and almost complete (*R*)‐stereoselectivity obtained with the mutant biocatalyst N9 Y71G (ee 96%, Table 5, entry 8).

## Conclusion

In conclusion, this work unveiled the biocatalytic thiolactonase activity of two lactonase enzymes, GcL, from the metallo‐β‐lactamase‐like lactonase family, and the rationally engineered N9 Y71G, a variant of the reconstructed ancestral PON enzyme N9. Even if lactonase enzymes have been widely studied from a biological perspective as quorum quenching enzymes, to date they have found limited application as biocatalysts in organic synthesis. Such limitation is partially due to the limited activity of lactonases towards non‐natural lactone substrates, which may undergo spontaneous hydrolysis under the biocatalytic reaction conditions. On the other hand, chiral γ‐thiolactones proved to be stable and to not spontaneously hydrolyse in the biocatalytic buffer solutions, thus being excellent substrates for deracemisation reactions with lactonase enzymes. Remarkably, in this study we identified two lactonase enzymes with different substrate affinities and biocatalytic properties. The lactonase GcL was found to catalyse the EKR of chiral γ‐thiolactones bearing an amide substituent at C3 position with high enantioselectivity and *E*‐values up to 136, while the rationally designed lactonase N9 Y71G was found to catalyse the DKR of γ‐thiolactones bearing an alkyl/arylthiol substituent at C3 position and to afford γ‐thio‐α‐thiocarboxylic acids with high conversions and ees up to 99%. The stability under the biocatalytic reaction conditions and the broad substrate scope explored in this work highlight the robustness of the lactonase enzymes in deracemising a wide range of γ‐thiolactones. In particular, the stability of these enzymes suggests the possibility that they could be recycled or immobilised in future preparative scale applications. Finally, insights into the mechanism of action and stereoselectivity of the lactonases were also gained through computational and mutagenesis studies. In summary, this work unlocked the potential of lactonase enzymes as biocatalysts for the sustainable and efficient deracemisation of chiral γ‐thiolactones.

## Supporting Information

Synthetic and biocatalytic procedures are reported. Copies of HPLC and NMR spectra are reported. The authors have cited additional references within the Supporting Information.^[87–112]^

## Conflict of Interests

The authors declare no conflict of interest.

## Supporting information



Supporting Information

## Data Availability

The data that support the findings of this study are available in the Supporting Information of this article.
