# Supplementary material for: Unlocking Lactonase Enzymes as Biocatalysts for the Deracemisation of Chiral γ‐Thiolactones
Source: Angew Chem Int Ed Engl. 2025 May 29;64(29):e202505032. doi: 10.1002/anie.202505032 (PMC12258673; doi:10.1002/anie.202505032)

Unlocking Lactonase Enzymes as Biocatalysts for the Deracemization of Chiral γ-Thiolactones

Jingyue Wu,[a] Michele Crotti,[a] Ivan Bassanini,[b] Mahdi Hassankalhori,[c] Erica Elisa Ferrandi,[b] Ferran Sancho,[c] Daniela Monti[b],* and Daniele Castagnolo[a],*

[a] Department of Chemistry, University College London, Marshgate Building, Manufacturing Futures Lab, 7 Sidings Street, E20 2AE, London, United Kingdom. [b] Istituto di Scienze e Tecnologie Chimiche "Giulio Natta" Consiglio Nazionale delle Ricerche, Via Mario Bianco 9, Milano, 20131, Italy. [c] Zymvol Biomodeling, C/ Pau Claris, 94, 3B, 08010, Barcelona, Spain.

Supporting information

Table of Contents

1. General S4
2. Biology S5
2.1. Sequences of the enzymes used in the study S5
2.2. DNA sequence used in the study S6
2.3. Primers used in this work to generate point mutations of N9 .... S6
2.4. Site-directed mutagenesis of N9 .... S7
2.5. Enzyme recombinant expression and purification S7
2.6. Enzyme activity assay of GcL S8
2.7. Enzyme activity assay of N9 Y71G lysate S9
3. Chemistry S11
3.1. General procedure for the synthesis of racemic thiolactones **1** S11
3.2. General procedure for the synthesis of racemic thiocarboxylic acids **2** S15
3.3. General procedure for the synthesis of lactones **3** S19
3.4. General procedure for the synthesis of racemic acids **4** S20
3.5. General procedure of the GcL biocatalysed EKR of thiolactones **1** S21
3.6. Racemisation test of (*R*)-**1a** and (*S*)-**2a** S22
3.7. ^{1}H-NMR monitoring of the EKR reaction of thiolactone **1a** catalysed by GcL S23
3.8. General procedure for the preparation of racemic lactones **8** and thiolactones **5** S24
3.9. General procedure for the preparation of racemic thiocarboxylic acids **6** S35
3.10. General procedure for the preparation of racemic thiocarboxylic acids **9** S40
3.11. Procedure for the preparation of racemic thiolactone **10** S42
3.12. Procedure for the preparation of the racemic thiocarboxylic acid **11** S42
3.13. General procedure of the N9 enzymes catalysed DKR reaction for the synthesis of the acids (*R*)-**6** or (*R*)-**9** (analytical scale) S43
3.14. General procedure for the synthesis of the acids (*R*)-**6** and (*R*)-**9** through N9 enzymes DKR reaction S44
3.15. Preparative scale synthesis of the acids (*R*)-**6a** through N9 Y71G enzyme DKR reaction S44
3.16. General procedure of N9 Y71G catalysed hydrolysis of **10** S44
3.17. Racemisation test of (*R*)-**5a** and (*R*)-**6a** S45
3.18. Deuterium labelling experiment on thiolactone (*R*)-**5a** S46
3.19. Racemisation test of (*R*)-**8b** and (*R*)-**9b** S47
3.20. Equilibrium test on lactone **8b** and acid **9b** S48
3.21. Screening of N9 mutants on lactone **8a** S49
3.22. Screening of N9 mutants on lactone **10** S49
3.23. Mutants test on thiolactone **5a** S50

3.24. Additional screening of lactonase enzymes S50
3.25. Optical rotations of compound (*R*)-**1** and (*S*)-**2** S51
3.26. Optical rotations of DKR products S52
4. Computational study S53
5. References S58
6. Conditions for HPLC analysis and copies of HPLC spectra S60
6.1. HPLC analysis for GcL catalysed EKR S60
6.2. HPLC analysis for DKR catalysed by N9 Y71G S73
6.3. HPLC analysis for enzymatic reactions catalysed by N9 .... S90
7. Copies of NMR spectra S93

1. General

Reagents and solvents were used as supplied from the vendor without further purification. Oligonucleotide primers used in this work (Table S1) were purchased from Merck Life Science Limited (Gillingham, UK). Sanger sequencing of constructs and mutants were carried out by Genewiz (Azenta Life Sciences). Thin layer chromatography plates (Merk, silica gel 60 F254, aluminium backed) were viewed under UV light. $MgSO_4$ (Sigma Aldrich, anhydrous ≥ 98.0 %) was used as the drying agent. Column chromatography was performed on silica gel for flash chromatography (Sigma Aldrich, 40-63 µm particle size, 60 Å pore size). Products were characterised by ^{1}H NMR, ^{13}C NMR and ^{19}F NMR spectra where applicable obtained from one of the following: a) Bruker (Germany) Ascend400 Spectrometer (dH 400 MHz, dC 101 MHz, dF 376 MHz) at 300 K; b) Bruker (Germany) Avance III 400 (dH 400 MHz, dC 101 MHz) at 300 K; c) Bruker (Germany) Avance Neo 500 (dH 500 MHz, dC 126 MHz) at 300 K. Chemical shifts are reported in ppm, referenced to tetramethylsilane. Coupling constants (*J*) are reported in Hertz (Hz), multiplicities are specified as singlet (s), doublet (d), triplet (t), doublet of doublet (dd), doublet of quartet (dq), doublet of doublet of doublet (ddd), triplet of doublet (td), multiplet (m). Chiral HPLC analysis was carried out using one of the following: a) Agilent series 1100 LC system coupled with UV detector; b) Agilent series 1260 UHPLC system coupled with UV detector. The columns used were Chiralpak IC® (0.5µm, 4.6mm X 250mm), Chiralpak IG® (5µm, 4.6mm X 250mm), Chiralpak ID® (0.5µm, 4.6mm X 250mm), and Chiracel OJ-H (0.5µm, 4.6mm X 250mm) supplied by Daicel. Hexane and ethanol (EtOH) were used as an isocratic mobile phase system for all columns. Reversed phase HPLC analysis was carried out using Agilent series 1260 UHPLC system coupled with UV detector. The column used was Agilent Eclipse Plus C18 column. CH_3CN and H_2O were used as mobile phase components. Mass spectra were acquired in positive or negative mode scanning over the mass range of 50 – 1500. The following ion source parameters were used: drying gas flow, 12 mL/min; nebulize pressure, 35 psi; and drying gas temperature, 350 ^{o}C. $[\alpha]_D$ measurements were taken using a Bellingham and Stanley ADP440+ Polarimeter with a cell length of 0.5 dm.

2. Biology

2.1. Sequences of the enzymes used in the study

>GcL (WP_017434252.1 N-acyl homoserine lactonase family protein [*Parageobacillus caldoxylosilyticus*])

MANVIKARPKLYVMDNGRMRMDKNWMIAMHNPATIHNPNAQTEFVEFPIYTVLIDHPEGKI
LFDTSCNPNSMGPQGRWAESTQQMFPWTATEECYLHNRLEQLKVRPEDIRYVVASHLHLDH
AGCLEMFTNATIIVHEDEFNGALQCYARNQKEGAYIWADIDAWIKNNLQWRTVKRHEDNIL
LAEGVKVLNFGSGHAWGMLGLHVELPETGGIILASDAIYTAESYGPPIKPPGIIYDSLGYMNT
VERIRRIAQETKSQVWFGHDAEQFKKFRKSTEGYYE

>N9[8]
MGKLVALTLLGIGLALVGERLLAFRNRLNASREVEPVDLPNCHLIKGIETGSEDIDILPNGLAF
ISSGLKYPGLKSFAPDKPGKIFLMDLNEEKPRALELRISRGFDVASFNPHGISTFIDKDDTVYLF
VVNHPHMKSTVEIFKFEEEENSLVHLKTIKHELLPSVNDIVAVGPEHFYATNDHYFTDFFLKF
LEMYLGLHWSNVVYYSPKEVKVVAEGFDSANGINISPDKKYIYVADILAHNIHVMEKHANW
NLTQLKVLQLDTLVDNLSVDPDTGDIWVGCHPNGMKLFFYDPDNPPGSEVLRIQNILSEKPT
VTTVYANNGSVLQGSSVASVYDGKLLIGTVFHKALYCEL

>VmutPLL (WP_202795173.1 phosphotriesterase [Vulcanisaeta moutnovskia])

GMVRISIAGGNEIDPGSMGLTLFHEHLRLITEVVRWNWPHLYNEDEELKRAIDAVNAAKKYG
VKTIIDLTVAGIGCDVRFNEKVAKATGVNIIMGTGFYTYTEIPFYFKNRGIDSLVDAFVHDITI
GIQGTNTRAAFVKAVIDSSGLTKDVEMAIRAAAKAHIKTDVPIITHSFVGNKSSLDLIRIFKEE
GVDLARTVIGHVGDTDDISFIEQILREGAFIGLDRFGLDIYLPLDKRVKTAIELIKRGWIDQLLL
SHDYCPTIDWYPPEVVRSTVPDWTMTLIFEKVIPRMRSEGITEEQINRVLIDNPRRLFTGR

2.2. DNA sequence used in the study

>N9[8]

ATGGGAAAATTAGTCGCCTTGACATTACTGGGCATCGGGCTTGCATTAGTGGGAGAGCGT
CTGCTGGCCTTTCGCAATCGTTTGAATGCTAGCCGCGAAGTAGAGCCTGTTGATCTCCCC
AATTGTCATCTGATTAAAGGTATTGAGACCGGTTCTGAAGACATCGATATCCTGCCGAAC
GGTTTAGCCTTCATCTCATCCGGCCTGAAGTATCCTGGTTTAAAATCGTTTGCGCCGGAC
AAGCCAGGTAAGATCTTTCTCATGGATTTAAATGAGGAGAAGCCTCGTGCACTGGAGCT
GCGTATTTCACGTGGGTTCGACGTAGCTTCTTTCAACCCACATGGCATTAGCACCTTTATC
GACAAGGACGATACTGTCTATCTTTTCGTTGTCAATCATCCGCACATGAAGAGCACGGTG
GAGATCTTCAAGTTCGAAGAGGAAGAGAACAGCCTCGTGCACCTGAAAACGATTAAGCA
CGAACTTCTTCCTTCAGTAAATGATATCGTAGCAGTTGGTCCAGAGCACTTTTACGCCAC
TAATGACCATTACTTTACGGATTTCTTCCTCAAGTTCCTGGAAATGTACTTGGGCTTACAC
TGGAGTAATGTGGTTTACTACAGCCCTAAGGAAGTTAAGGTCGTAGCCGAGGGTTTCGAC
AGCGCCAATGGCATCAACATTTCACCAGACAAGAAGTACATCTATGTGGCTGACATTCTG
GCGCATAATATCCATGTAATGGAGAAGCACGCGAACTGGAACTTAACACAACTGAAAGT
TCTGCAATTGGACACCCTCGTAGACAATTTGTCAGTGGACCCCGACACAGGGGACATCTG
GGTGGGGTGCCACCCGAATGGGATGAAGCTGTTCTTCTACGATCCTGACAACCCACCAG
GTAGCGAGGTACTCCGCATTCAAAACATCTTAAGCGAGAAGCCGACGGTTACTACAGTC
TATGCGAATAACGGAAGTGTGCTGCAAGGATCTAGCGTCGCCAGTGTTTACGACGGTAA
GTTGCTTATTGGAACAGTCTTTCACAAAGCGCTCTATTGTGAGTTA

2.3. Primers used in this work to generate point mutations of N9

Table S1. Primers used in this work to generate point mutations of N9.

Name		5' to 3'
N167A	F	CCTTCAGTAGCTGATATCGTAGCAGTTGGTCC
N167A	R	CGATATCAGCTACTGAAGGAAGAAGTTCGTGCT
D182A	F	GCCACTAATGCCCATTACTTTACGGATTTCT
D182A	R	GTAATGGGCATTAGTGGCGTAAAAGTG
Y71A	F	GCCTGAAGGCTCCTGGTTTAAAATCGTTTGCGCC
Y71A	R	ACCAGGAGCCTTCAGGCCGGATGAGATGAAG
Y71G	F	CCTGAAGGGTCCTGGTTTAAAATCGTTTGCGCC
Y71G	R	AACCAGGACCCTTCAGGCCGGATGAGATGA
Y71L	F	CCTGAAGCTTCCTGGTTTAAAATCGTTTGCGCC
Y71L	R	AACCAGGAAGCTTCAGGCCGGATGAGATG
Y71I	F	CTGAAGATTCCTGGTTTAAAATCGTTTGCGCC
Y71I	R	CCAGGAATCTTCAGGCCGGATGAGATGAA
Y71M	F	CCTGAAGATGCCTGGTTTAAAATCGTTTGCGCCG
Y71M	R	AACCAGGCATCTTCAGGCCGGATGAGATGAAGG
Y71W	F	CTGAAGTGGCCTGGTTTAAAATCGTTTGCGCCGG
Y71W	R	ACCAGGCCACTTCAGGCCGGATGAGATGAAGG
H133A	F	GTTGTCAATGCTCCGCACATGAAGAGCACG
H133A	R	CATGTGCGGAGCATTGACAACGAAAAGATAGAC

F = forward primer, R = reverse primer.

2.4. Site-directed mutagenesis of N9

The mutants were created by Phusion® High-Fidelity PCR Master Mix with HF Buffer (New England Biolabs, UK). Primers containing mutated codons (Table S1) were used for the PCR and each site was targeted individually using wild-type N9 plasmid as template (pET28a). Thermo Scientific® Phusion® High-Fidelity DNA Polymerase was used for the PCR. After the PCR amplification, the reaction mixture was treated with DpnI, subsequently, the DNA was used to transform to *E. coli* DH5α chemically competent cells, and transformants were selected on LB-agar plates supplemented with 50 μg mL^{-1} kanamycin. For each transformation, 2-3 colonies were randomly picked for plasmid DNA isolation and sequencing to check the incorporation of the mutation. Plasmids bearing the expected mutation were used for enzyme production and purification.

Table S2. PCR conditions used to introduce point mutations

Segment	Cycles	Temperature	Time
1	1	98 °C	30 secs
		98 °C	10 secs
2	30	55 °C	30 secs
		72 °C	3.5 min
3	1	72 °C	10 min
4	1	4 °C	Hold

2.5. Enzyme recombinant expression and purification

Recombinant expression of GcL[15] was carried out with *E. coli* BL21(DE3)-pGro7 cells transformed with the pET28a expression vector containing the synthetic gene coding for GcL and grown overnight at 37 °C and 220 rpm in 100 mL LB broth containing kanamycin (30 μg mL^{-1}) and chloramphenicol (20 μg mL^{-1}). The preculture was then used to inoculate 1 L autoinduction medium ZYM[87] containing L-arabinose (0.1% v/v), kanamycin (30 μg mL^{-1}) and chloramphenicol (20 μg mL^{-1}).[87] Cells were grown at 37 °C and 220 rpm to an OD_{600} 0.6-0.9, and then $CoCl_2$ was added (0.2 mM final concentration). After overnight at 18 °C, cells were harvested by centrifugation (5000 rpm, 30 min, 4°C) and resuspended in 20 mL of "wash buffer" (20 mM potassium phosphate, pH 7.0, containing 500 mM NaCl, 20 mM imidazole). The lysis of the bacterial cells was conducted by ultrasonication (5 cycles, 30 s each at 40% of maximum power, Omni Ruptor 250-Watt Ultrasonic Cell Disrupter), followed by centrifugation (10000 rpm 30 min, 4 °C). Protein purification was carried out by affinity chromatography on Ni-NTA Sepharose 6 Fast Flow resin (GE Healthcare, Italy), with stepwise elution with wash buffer containing 100-300 mM imidazole. Fractions were analyzed by determination of protein concentration (Bradford assay) and separately dialyzed against 100 mM Tris-HCl buffer, pH 8.0, 150 mM NaCl, 0.2 mM $CoCl_2$. Protein purity was confirmed by SDS-PAGE (10% T, 2.6% C) using as the reference marker a molecular weight protein standard mixture from BioRad (Karlsruhe, Germany).

Recombinant expression of VmutPLL[75] was carried out with *E. coli* Rosetta™ (DE3) cells transformed with the pET24a expression vector containing the gene coding for VmutPLL (kindly gifted by Prof. Bettina Siebers, University of Duisburg-Essen, Germany). Cells were grown overnight at 37 °C and 220 rpm in 100 mL LB broth containing kanamycin (30 µg mL^{-1}), chloramphenicol (20 µg mL^{-1}), and 1 mM $MnCl_2$, then used to inoculate 1 L of the same medium. Cultures were maintained at 37 °C and 220 rpm to OD_{600} 0.4-0.6, then the expression was induced by the addition of 0.5 M IPTG to a final concentration of 1 mM, and the bacterial culture was incubated overnight at 220 rpm and 30 °C. Subsequently, cells were harvested by centrifugation (5000 rpm, 30 min, 4°C), resuspended in 20 mL of wash buffer and lysed by ultrasonication as previously described. Partial purification of VmutPLL was carried out by heat precipitation of the cell extract performed at 80 °C for 20 min followed by centrifugation (10000 rpm 30 min, 4 °C). Recombinant expression of VmutPLL was checked by SDS-PAGE analysis (10% T, 2.6% C) as previously described and enzyme solution was stored at – 80 °C until use.

Recombinant expression of N9 and N9 mutants[8] was carried out with *E. coli* Rosetta™ (DE3) cells transformed with the pET28a expression vector containing the synthetic gene coding for wild-type N9 or N9 mutants and grown overnight at 37°C in LB broth containing kanamycin (30 µg mL^{-1}) and chloramphenicol (20 µg mL^{-1}). The preculture was then used to inoculate fresh TB media (1% v/v) containing 1 mM $CaCl_2$, kanamycin (30 µg mL^{-1}) and chloramphenicol (20 µg mL^{-1}). Cells were grown at 37 °C to an OD_{600} 0.6-0.9, and then IPTG was added (1 mM final concentration) for inducing protein expression. Cells were harvested by centrifugation (5000 rpm, 30 min, 4°C) after overnight at 18°C. Pellets were resuspended in 50 mM Tris-HCl buffer, pH 8.0, 1 mM $CaCl_2$, 150 mM NaCl, and lysed by sonication. Cell debris was removed by centrifugation (12,000 rpm, 15 min, 4 °C) and supernatant was collected as cell lysate and used in the reactions.

Table S3. Lactonases recombinant expression.

Enzyme	Expression vector	Expression host	Inducer
GcL	pET28a	E. coli BL21(DE3)-pGro7	autoinduction medium ZYM containing L-arabinose (0.1% v/v)
VmutPLL	pET24a	E. coli Rosetta™ (DE3)	IPTG (final concentration: 1 mM)
N9 and N9 mutants	pET28a	E. coli Rosetta™ (DE3)	IPTG (final concentration: 1 mM)

2.6. Enzyme activity assay of GcL

GcL mediated hydrolysis of **1a** yielding the deprotonated acid form of the acid and a proton was measured according to a colorimetric assay[88] at 577 nm by monitoring the decrease in pH using *m*-cresol-purple as a pH indicator. As reported, the assay required an *in situ* standard acid titration curve with 0.1 mM acetic acid, giving an absorption coefficient of 2.9 mM^{-1} cm^{-1}.[75] The reaction mixture

contained 2.5 mM Tris buffer pH 8.3, 100 mM NaCl, 5% (v/v) DMSO, and 0.15 mM cresol purple as well as 0.5% (v/v) lactonase and 30 mM thiolactone substrate **1a**. The enzyme unit is defined as the amount of enzyme that catalyses the conversion of 1 µmol of substrate in one minute under defined assay conditions.

The activity of GcL used in this study was measured as 0.54 U/mL (average value of duplicate experiments) using the equation below.

$$U/mL = \frac{\Delta A/\min \times DF \times Vol_{\mathrm{tot}}}{V_{\mathrm{camp}} \times \varepsilon}$$

• ΔA/min: absorbance change at 580 nm per minute; DF: dilution factor; Vol_{tot}: total reaction volume; Vcamp: sample volume; ε: molar extinction coefficient.

2.7. Enzyme activity assay of N9 Y71G lysate

To 950 µL 50 mM Tris-HCl buffer pH 9.0 containing $CaCl_2$ (4 mM) and N9 Y71G cell lysate (10% v/v, 100 µL), 50 µL of a 200 mM stock solution in DMSO of the thiolactone **5a** (10 mM final concentration) was added. The resulting reaction was shaken at 37 ºC. At the set time points, 100 µL reaction mixture was aliquoted and mixed with 900 µL CH_3CN, then filtered for HPLC analysis. The consumption of the substrate **5a** was quantified by comparing the recorded peak area with the standard calibration curve. The enzyme unit is defined as the amount of enzyme that catalyses the conversion of 1 µmol of substrate in one minute under defined assay conditions.

The activity of N9 Y71G lysate used in this study was measured as 2.0 U/mL (average value of duplicate experiments) using the equation below.

$$U/mL = \frac{\Delta c/\min \times DF \times Vol_{\mathrm{tot}}}{V_{\mathrm{enz}}}$$

• Δc/min: change in substrate concentration (µmol/mL) per minute (determined using HPLC + calibration curve); DF: dilution factor; Vol_{tot}: total reaction volume; V_{enz}: volume of enzyme solution used.

HPLC analysis for the activity assay for N9 Y71G

HPLC conditions: Agilent Eclipse Plus C18 column (4.6 mm × 150 mm, 5 µm), 240 nm, 1.0 mL/min, water and acetonitrile were used as mobile phase and the gradient is shown below.

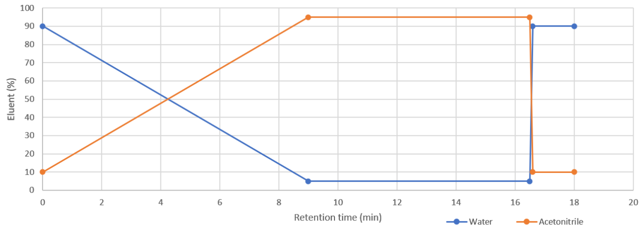

Standard calibration curve of **5a**

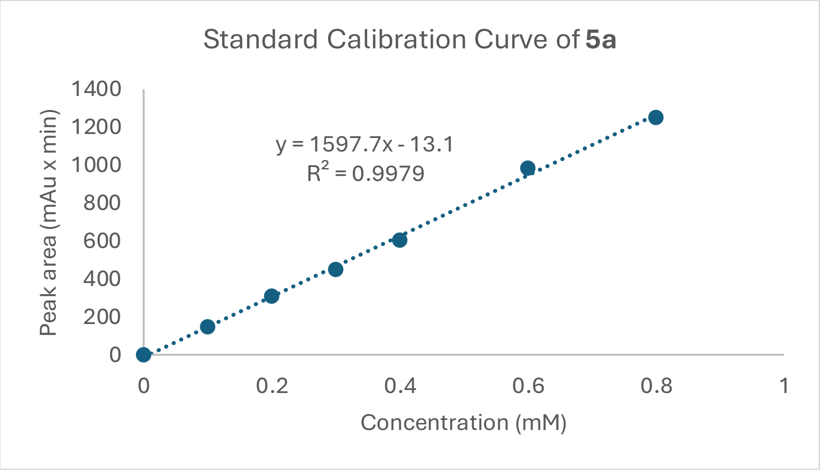

Time Course of Substrate **5a** Consumption

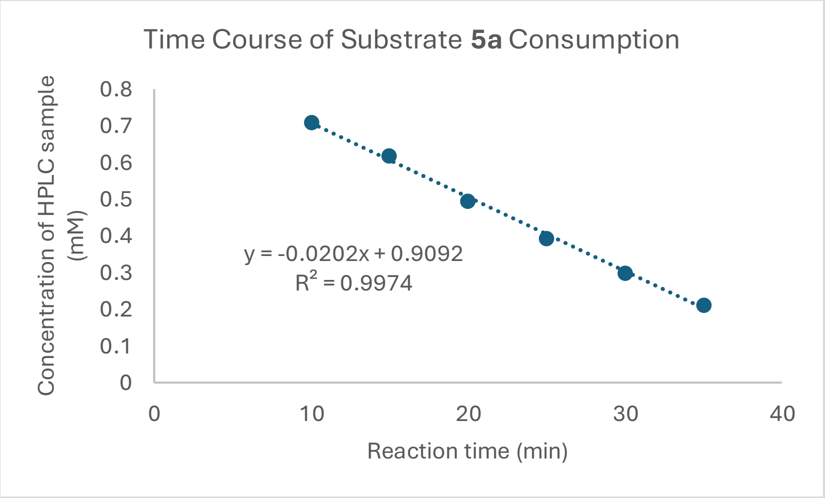

3. Chemistry

3.1. General procedure for the synthesis of racemic thiolactones 1

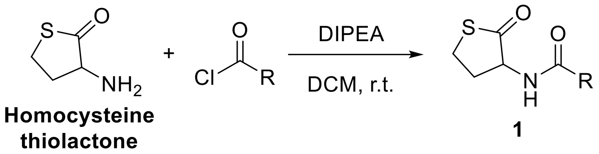

To a solution of homocysteine thiolactone hydrochloride (3.0 mmol, 461 mg) and DIPEA (6.0 mmol, 776 mg) in DCM at 0 °C under anhydrous conditions, appropriate acyl chloride (3.3 mmol) was added dropwise. The mixture was allowed to warm up to room temperature and was stirred for another 3 hours. Then, the reaction mixture was washed with water and extracted by DCM. The organic phase was dried over $MgSO_4$, filtered, and concentrated under reduced pressure. The crude products were purified by flash column chromatography (Hexane/AcOEt 9:1 to Hexane/AcOEt 6:4) to afford corresponding thiolactones **1**.

***N*-(2-Oxotetrahydrothiophen-3-yl)hexanamide 1a**[76]

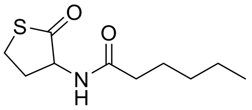

Hexanoyl chloride (3.3 mmol, 444 mg) was used in the reaction. The product was afforded as white amorphous solid (575 mg, 89% yield). ^{1}H NMR (500 MHz, $CDCl_3$) δ 6.29 (d, *J* = 6.6 Hz, 1H), 4.57 – 4.48 (m, 1H), 3.37 – 3.28 (m, 1H), 3.24 – 3.16 (m, 1H), 2.87 – 2.78 (m, 1H), 2.30 – 2.12 (m, 2H), 1.98 – 1.85 (m, 1H), 1.65 – 1.53 (m, 2H), 1.34 – 1.19 (m, 4H), 0.85 (t, *J* = 6.8 Hz, 3H) ppm. ^{13}C{^{1}H} NMR (126 MHz, $CDCl_3$) δ 205.7, 173.89, 59.3, 36.4, 31.9, 31.4, 27.6, 25.3, 22.4, 13.9 ppm.

***N*-(2-Oxotetrahydrothiophen-3-yl)benzamide 1b**[89]

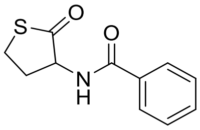

Benzoyl chloride (3.3 mmol, 464 mg) was used in the reaction. The product was afforded as yellow amorphous solid (238 mg, 36% yield). ^{1}H NMR (500 MHz, $CDCl_3$) δ 7.84 – 7.78 (m, 2H), 7.56 – 7.49 (m, 1H), 7.48 – 7.41 (m, 2H), 6.61 (s, 1H), 4.68 (ddd, *J* = 12.6, 6.8, 5.4 Hz, 1H), 3.48 – 3.39 (m, 1H), 3.35 – 3.28 (m, 1H), 3.16 – 3.08 (m, 1H), 2.09 – 1.96 (m, 1H) ppm. ^{13}C{^{1}H} NMR (126 MHz, $CDCl_3$) δ 205.8, 167.9, 133.5, 132.2, 128.8, 127.3, 60.2, 32.4, 27.9 ppm.

***N*-(2-Oxotetrahydrothiophen-3-yl)isobutyramide 1c**

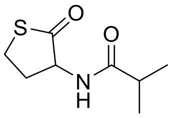

Isobutyryl chloride (3.3 mmol, 352 mg) was used in the reaction. The product was afforded as white amorphous solid (482 mg, 86% yield). ^{1}H NMR (500 MHz, $CDCl_3$) δ 5.88 (s, 1H), 4.52 – 4.43 (m, 1H), 3.36 (ddd, *J* = 12.2, 11.4, 5.2 Hz, 1H), 3.26 (ddd, *J* = 11.4, 6.9, 1.3 Hz, 1H), 3.03 – 2.94 (m, 1H), 2.49 – 2.37 (m, 1H), 1.95 – 1.83 (m, 1H), 1.18 (dd, *J* = 6.9, 3.5 Hz, 6H) ppm. ^{13}C{^{1}H} NMR (126 MHz, $CDCl_3$) δ 205.9, 177.6, 59.6, 35.6, 32.4, 27.8, 19.7, 19.6 ppm. HRMS(ESI) m/z calculated for $C_8H_{14}NO_2S^+$ [M + H]$^+$ 188.0667, found: 188.0733.

***N*-(2-Oxotetrahydrothiophen-3-yl)pivalamide 1d**[90]

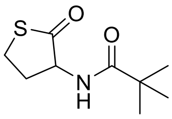

Pivaloyl chloride (3.3 mmol, 398 mg) was used in the reaction. The product was afforded as white amorphous solid (510 mg, 81% yield). ^{1}H NMR (500 MHz, $CDCl_3$) δ 6.07 (s, 1H), 4.43 (ddd, *J* = 12.6, 6.8, 5.5 Hz, 1H), 3.36 (ddd, *J* = 12.3, 11.4, 5.1 Hz, 1H), 3.25 (ddd, *J* = 11.3, 6.9, 1.3 Hz, 1H), 3.03 – 2.94 (m, 1H), 1.94 – 1.82 (m, 1H), 1.59 (s, 1H), 1.22 (s, 9H) ppm. ^{13}C{^{1}H} NMR (126 MHz, $CDCl_3$) δ 206.0, 179.2, 59.8, 39.0, 32.3, 27.8, 27.6 ppm.

***N*-(2-Oxotetrahydrothiophen-3-yl)cyclopropanecarboxamide 1e**[91]

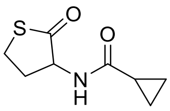

Cyclopropanecarbonyl chloride (3.3 mmol, 345 mg) was used in the reaction. The product was afforded as white amorphous solid (361 mg, 65% yield). ^{1}H NMR (500 MHz, $CDCl_3$) δ 6.20 (s, 1H), 4.57 – 4.49 (m, 1H), 3.39 – 3.30 (m, 1H), 3.24 (ddd, *J* = 11.3, 7.0, 1.3 Hz, 1H), 2.97 – 2.88 (m, 1H), 2.00 – 1.87 (m, 1H), 1.48 – 1.39 (m, 1H), 1.02 – 0.92 (m, 2H), 0.84 – 0.72 (m, 2H) ppm. ^{13}C{^{1}H} NMR (126 MHz, $CDCl_3$) δ 206.0, 174.3, 59.8, 32.3, 27.7, 14.7, 7.9, 7.9 ppm.

***N*-(2-Oxotetrahydrothiophen-3-yl)cyclobutanecarboxamide 1f**

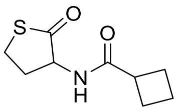

Cyclobutanecarbonyl chloride (3.3 mmol, 391 mg) was used in the reaction. The product was afforded as brown amorphous solid (196 mg, 33% yield). ^{1}H NMR (500 MHz, $CDCl_3$) δ 5.76 (s, 1H), 4.48 (dt, J = 12.7, 6.3 Hz, 1H), 3.36 (ddd, J = 12.1, 11.4, 5.1 Hz, 1H), 3.25 (ddd, J = 11.3, 6.9, 1.3 Hz, 1H), 3.11 – 2.94 (m, 2H), 2.35 – 2.23 (m, 2H), 2.22 – 2.12 (m, 2H), 2.03 – 1.93 (m, 1H), 1.93 – 1.84 (m, 2H) ppm. ^{13}C{^{1}H} NMR (126 MHz, $CDCl_3$) δ 205.9, 175.7, 59.6, 39.7, 32.4, 27.8, 25.5, 18.3 ppm. HRMS(ESI) m/z calculated for $C_9H_{14}NO_2S^+$ $[M + H]^+$ 200.0667, found: 200.0730.

***N*-(2-Oxotetrahydrothiophen-3-yl)cyclohexanecarboxamide 1g**

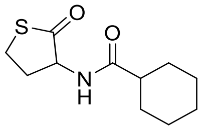

Cyclohexanecarbonyl chloride (3.3 mmol, 484 mg) was used in the reaction. The product was afforded as white amorphous solid (499 mg, 73% yield). ^{1}H NMR (500 MHz, $CDCl_3$) δ 5.89 (s, 1H), 4.52 – 4.43 (m, 1H), 3.40 – 3.31 (m, 1H), 3.28 – 3.21 (m, 1H), 3.01 – 2.93 (m, 1H), 2.20 – 2.10 (m, 1H), 1.97 – 1.81 (m, 3H), 1.82 – 1.74 (m, 2H), 1.71 – 1.64 (m, 1H), 1.49 – 1.37 (m, 2H), 1.35 – 1.15 (m, 3H) ppm. ^{13}C{^{1}H} NMR (126 MHz, $CDCl_3$) δ 205.9, 176.8, 59.6, 45.3, 32.4, 29.8, 29.7, 27.8, 25.8 ppm. HRMS(ESI) m/z calculated for $C_{11}H_{18}NO_2S^+$ $[M + H]^+$ 228.0980, found: 227.1047.

***N*-(2-Oxotetrahydrothiophen-3-yl)-2-phenylacetamide 1h**[76]

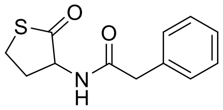

2-Phenylacetyl chloride (3.3 mmol, 510 mg) was used in the reaction. The product was afforded as pale yellow amorphous solid (240 mg, 34% yield). ^{1}H NMR (500 MHz, $CDCl_3$) δ 7.33 – 7.28 (m, 2H), 7.27 – 7.20 (m, 3H), 5.80 (s, 1H), 4.48 – 4.40 (m, 1H), 3.57 (s, 2H), 3.32 – 3.23 (m, 1H), 3.20 – 3.13 (m, 1H), 2.89 – 2.81 (m, 1H), 1.86 – 1.74 (m, 1H) ppm. ^{13}C{^{1}H} NMR (126 MHz, $CDCl_3$) δ 205.3, 171.6, 134.3, 129.6, 129.2, 127.7, 59.7, 43.6, 32.0, 27.7 ppm.

4-Fluoro-N-(2-oxotetrahydrothiophen-3-yl)benzamide 1i

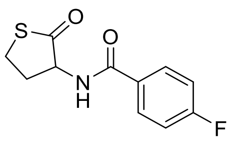

4-Fluorobenzoyl chloride (3.3 mmol, 523 mg) was used in the reaction. The product was afforded as white amorphous solid (497 mg, 69% yield). ^{1}H NMR (500 MHz, $CDCl_3$) δ 7.83 – 7.75 (m, 2H), 7.10

– 7.02 (m, 2H), 6.85 (d, *J* = 6.2 Hz, 1H), 4.72 (dt, *J* = 13.0, 6.6 Hz, 1H), 3.40 (td, *J* = 11.8, 5.1 Hz, 1H), 3.28 (ddd, *J* = 11.4, 6.9, 1.3 Hz, 1H), 3.02 – 2.93 (m, 1H), 2.12 – 1.99 (m, 1H) ppm. $^{13}C\{^1H\}$ NMR (126 MHz, $CDCl_3$) δ 206.2, 166.8, 165.1 (d, *J* = 252.8 Hz), 129.7 (d, *J* = 9.1 Hz), 129.6 (d, *J* = 3.1 Hz), 115.8 (d, *J* = 21.9 Hz), 60.0, 32.0, 27.8 ppm. ^{19}F NMR (376 MHz, $CDCl_3$) δ -107.2 (tt, *J* = 9.0, 5.3 Hz) ppm. HRMS(ESI) m/z calculated for $C_{11}H_{11}FNO_2S^+$ $[M + H]^+$ 240.0416, found: 240.0487.

4-Chloro-*N*-(2-oxotetrahydrothiophen-3-yl)benzamide 1j

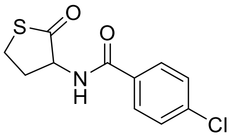

4-Chlorobenzoyl chloride (3.3 mmol, 578 mg) was used in the reaction. The product was afforded as white amorphous solid (606 mg, 79% yield). ^1H NMR (500 MHz, $CDCl_3$) δ 7.77 – 7.71 (m, 2H), 7.45 – 7.39 (m, 2H), 6.58 (s, 1H), 4.66 (ddd, *J* = 12.7, 6.9, 5.5 Hz, 1H), 3.43 (ddd, *J* = 12.1, 11.4, 5.1 Hz, 1H), 3.32 (ddd, *J* = 11.4, 7.0, 1.3 Hz, 1H), 3.15 – 3.06 (m, 1H), 2.11 – 1.96 (m, 1H) ppm. $^{13}C\{^1H\}$ NMR (126 MHz, $CDCl_3$) δ 205.8, 166.9, 138.5, 131.9, 129.1, 128.7, 60.2, 32.3, 27.9 ppm. HRMS(ESI) m/z calculated for $C_{11}H_{11}ClNO_2S^+$ $[M + H]^+$ 256.0121, found: 256.0190.

2-Chloro-*N*-(2-oxotetrahydrothiophen-3-yl)benzamide 1k

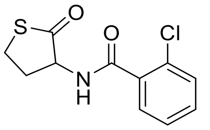

2-Chlorobenzoyl chloride (3.3 mmol, 578 mg) was used in the reaction. The product was afforded as white amorphous solid (476 mg, 62% yield). ^1H NMR (500 MHz, $CDCl_3$) δ 7.70 (dd, *J* = 7.6, 1.7 Hz, 1H), 7.44 – 7.37 (m, 2H), 7.34 (td, *J* = 7.3, 1.6 Hz, 1H), 6.71 (s, 1H), 4.71 (dt, *J* = 12.7, 6.3 Hz, 1H), 3.44 (td, *J* = 11.8, 5.1 Hz, 1H), 3.36 – 3.29 (m, 1H), 3.18 – 3.10 (m, 1H), 2.13 – 2.01 (m, 1H) ppm. $^{13}C\{^1H\}$ NMR (126 MHz, $CDCl_3$) δ 205.1, 166.9, 134.0, 132.0, 131.1, 130.6, 130.5, 127.3, 60.3, 32.1, 27.9 ppm. HRMS(ESI) m/z calculated for $C_{11}H_{11}ClNO_2S^+$ $[M + H]^+$ 256.0121, found: 256.0192.

4-Methyl-*N*-(2-oxotetrahydrothiophen-3-yl)benzamide 1l[91]

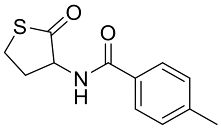

4-Methylbenzoyl chloride (3.3 mmol, 510 mg) was used in the reaction. The product was afforded as white amorphous solid (365 mg, 52% yield). ^1H NMR (500 MHz, $CDCl_3$) δ 7.69 (d, *J* = 8.3 Hz, 2H), 7.23 (d, *J* = 7.9 Hz, 2H), 4.67 (ddd, *J* = 12.6, 6.8, 5.6 Hz, 1H), 3.42 (ddd, *J* = 12.1, 11.4, 5.2 Hz, 1H),

3.30 (ddd, J = 11.3, 6.9, 1.3 Hz, 1H), 3.13 – 3.04 (m, 1H), 2.39 (s, 3H), 2.08 – 1.96 (m, 1H) ppm. $^{13}C\{^{1}H\}$ NMR (126 MHz, $CDCl_3$) δ 206.0, 167.9, 142.7, 130.6, 129.4, 127.3, 60.1, 32.4, 27.9, 21.6 ppm.

4-Methoxy-*N*-(2-oxotetrahydrothiophen-3-yl)benzamide 1m

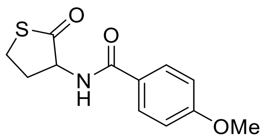

4-Methoxybenzoyl chloride (3.3 mmol, 563 mg) was used in the reaction. The product was afforded as pale yellow amorphous solid (419 mg, 56% yield). ^{1}H NMR (500 MHz, $CDCl_3$) δ 7.77 (d, J = 8.8 Hz, 2H), 6.93 (d, J = 8.8 Hz, 2H), 6.51 (s, 1H), 4.66 (dt, J = 12.5, 6.0 Hz, 1H), 3.85 (s, 3H), 3.42 (td, J = 11.8, 5.0 Hz, 1H), 3.34 – 3.27 (m, 1H), 3.11 (dt, J = 12.2, 6.0 Hz, 1H), 2.07 – 1.95 (m, 1H) ppm. $^{13}C\{^{1}H\}$ NMR (126 MHz, $CDCl_3$) δ 206.1, 167.4, 162.8, 129.2, 125.8, 114.0, 60.2, 55.6, 32.5, 27.9 ppm. HRMS(ESI) m/z calculated for $C_{12}H_{14}NO_3S^+$ $[M + H]^+$ 252.0616, found: 252.0689.

3.2. General procedure for the synthesis of racemic thiocarboxylic acids 2

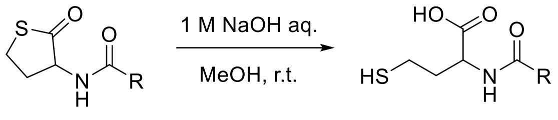

To a solution of appropriate thiolactone **1** (0.15 mmol) in MeOH (1 mL), 1M NaOH aq. (1 mL) was added. The reaction mixture was stirred vigorously at room temperature for 1h. Then, MeOH was removed under reduced pressure. The reaction mixture was extracted by AcOEt. The aqueous phase was acidified by 2M HCl aq. to pH 3 and extracted by AcOEt. The organic phase was dried over $MgSO_4$, filtered, and concentrated under reduced pressure. Then the mixture was loaded onto silica gel and elution with DCM:MeOH (95:5) afford the resulting pure corresponding γ-thio-α-amino acid **2**.

Hexanoyl homocysteine 2a

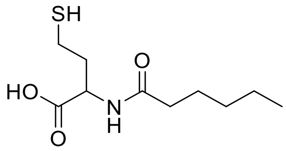

32 mg of **1a** (0.15 mmol) were hydrolysed. The corresponding acid was afforded as colourless oil (31 mg, 89% yield). ^{1}H NMR (500 MHz, MeOD) δ 4.62 – 4.50 (m, 1H), 2.86 – 2.68 (m, 1H), 2.64 – 2.48 (m, 1H), 2.33 – 2.21 (m, 2H), 2.15 – 1.93 (m, 2H), 1.67 – 1.56 (m, 2H), 1.41 – 1.27 (m, 4H), 0.92 (t, J

= 6.8 Hz, 3H) ppm. $^{13}C\{^{1}H\}$ NMR (126 MHz, MeOD) δ 176.5, 175.2, 52.2, 36.8, 36.0, 32.4, 26.6, 23.4, 21.6, 14.3 ppm. HRMS(ESI) m/z calculated for $C_{10}H_{20}NO_3S^+$ $[M + H]^+$ 234.1086, found: 234.1158.

Benzoyl homocysteine 2b[89]

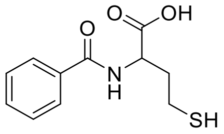

33 mg of **1b** (0.15 mmol) were hydrolysed. The corresponding acid was afforded as yellow oil (34 mg, 93% yield). ^{1}H NMR (500 MHz, MeOD) δ 7.88 – 7.82 (m, 2H), 7.56 – 7.49 (m, 1H), 7.49 – 7.41 (m, 2H), 4.85 – 4.72 (m, 1H), 2.95 – 2.52 (m, 2H), 2.47 – 2.36 (m, 1H), 2.27 – 2.10 (m, 1H) ppm. $^{13}C\{^{1}H\}$ NMR (126 MHz, MeOD) δ 175.1, 170.5, 135.3, 132.8, 129.5, 128.5, 128.5, 53.2, 32.2, 21.8 ppm.

Isobutyrylhomocysteine 2c

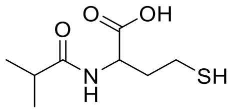

28 mg of **1c** (0.15 mmol) were hydrolysed. The corresponding acid was afforded as yellow oil (28 mg, 90% yield). ^{1}H NMR (400 MHz, MeOD) δ 4.61 – 4.43 (m, 1H), 2.61 – 2.40 (m, 3H), 2.13 – 1.87 (m, 2H), 1.08 (d, J = 6.8 Hz, 6H) ppm. $^{13}C\{^{1}H\}$ NMR (101 MHz, MeOD) δ 180.4, 175.1, 52.0, 37.0, 36.0, 21.6, 19.9, 19.7 ppm. HRMS(ESI) m/z calculated for $C_8H_{16}NO_3S^+$ $[M + H]^+$ 206.0773, found: 206.0837.

Pivaloylhomocysteine 2d

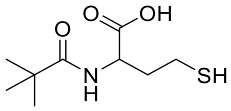

30 mg of **1d** (0.15 mmol) were hydrolysed. The corresponding acid was afforded as pale yellow oil (30 mg, 90% yield). ^{1}H NMR (500 MHz, MeOD) δ 4.63 – 4.56 (m, 1H), 2.63 – 2.46 (m, 2H), 2.18 – 2.00 (m, 2H), 1.21 (s, 9H) ppm. $^{13}C\{^{1}H\}$ NMR (126 MHz, MeOD) δ 181.6, 175.3, 52.3, 39.7, 36.6, 27.7, 21.8 ppm. HRMS(ESI) m/z calculated for $C_9H_{18}NO_3S^+$ $[M + H]^+$ 220.0929, found: 220.1001.

(Cyclopropanecarbonyl)homocysteine 2e

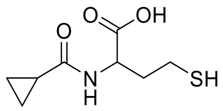

28 mg of **1e** (0.15 mmol) were hydrolysed. The corresponding acid was afforded as yellow oil (27 mg, 87% yield). ^{1}H NMR (400 MHz, MeOD) δ 4.68 – 4.49 (m, 1H), 2.65 – 2.46 (m, 2H), 2.18 – 1.91 (m, 2H), 1.72 – 1.55 (m, 1H), 0.93 – 0.71 (m, 4H) ppm. ^{13}C{^{1}H} NMR (101 MHz, MeOD) δ 176.8, 175.2, 52.4, 37.3, 21.6, 14.6, 7.5 ppm. HRMS(ESI) m/z calculated for $C_8H_{14}NO_3S^+$ [M + H]$^+$ 204.0616, found: 204.0680.

(Cyclobutanecarbonyl)homocysteine 2f

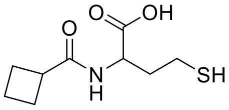

30 mg of **1f** (0.15 mmol) were hydrolysed. The corresponding acid was afforded as brown oil (29 mg, 88% yield). ^{1}H NMR (400 MHz, MeOD) δ 4.60 – 4.54 (m, 1H), 3.21 – 3.10 (m, 1H), 2.63 – 2.41 (m, 2H), 2.30 – 2.21 (m, 2H), 2.19 – 2.03 (m, 3H), 2.03 – 1.92 (m, 2H), 1.90 – 1.79 (m, 1H) ppm. ^{13}C{^{1}H} NMR (101 MHz, MeOD) δ 178.0, 175.2, 52.1, 40.5, 37.0, 26.0, 21.6, 19.1 ppm. HRMS(ESI) m/z calculated for $C_9H_{16}NO_3S^+$ [M + H]$^+$ 218.0773, found: 218.0837.

(Cyclohexanecarbonyl)homocysteine 2g

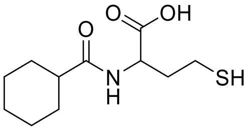

34 mg of **1g** (0.15 mmol) were hydrolysed. The corresponding acid was afforded as pale yellow oil (30 mg, 80% yield). ^{1}H NMR (500 MHz, MeOD) δ 4.60 – 4.48 (m, 1H), 2.84 – 2.67 (m, 1H), 2.63 – 2.47 (m, 1H), 2.33 – 2.22 (m, 1H), 2.15 – 1.86 (m, 2H), 1.87 – 1.74 (m, 4H), 1.75 – 1.62 (m, 1H), 1.52 – 1.37 (m, 2H), 1.39 – 1.17 (m, 4H) ppm. ^{13}C{^{1}H} NMR (126 MHz, MeOD) δ 179.5, 175.2, 51.9, 46.1, 37.0, 35.9, 32.4, 30.8, 30.5, 26.8, 21.6 ppm. HRMS(ESI) m/z calculated for $C_{11}H_{20}NO_3S^+$ [M + H]$^+$ 246.1086, found: 246.1154.

(2-Phenylacetyl)homocysteine 2h

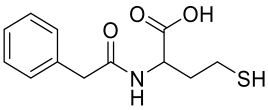

35 mg of **1h** (0.15 mmol) were hydrolysed. The corresponding acid was afforded as yellow oil (35 mg, 91% yield). ^{1}H NMR (500 MHz, MeOD) δ 7.34 – 7.26 (m, 4H), 7.25 – 7.18 (m, 1H), 4.59 (dd, *J* = 9.6, 4.6 Hz, 1H), 3.57 (s, 2H), 2.76 – 2.41 (m, 2H), 2.15 – 1.94 (m, 2H) ppm. ^{13}C{^{1}H} NMR (126 MHz, MeOD) δ 175.0, 174.2, 136.8, 130.1, 129.5, 127.9, 52.3, 43.5, 37.0, 21.5 ppm. HRMS(ESI) m/z calculated for $C_{12}H_{16}NO_3S^+$ [M + H]$^+$ 254.0773, found: 254.1115.

(4-Fluorobenzoyl)homocysteine 2i

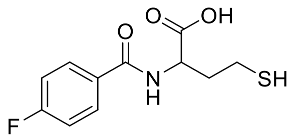

36 mg of **1i** (0.15 mmol) were hydrolysed. The corresponding acid was afforded as colourless oil (35 mg, 89% yield). ^{1}H NMR (400 MHz, MeOD) δ 7.93 – 7.81 (m, 2H), 7.21 – 7.09 (m, 2H), 4.76 (dd, *J* = 9.5, 4.9 Hz, 1H), 2.69 – 2.49 (m, 2H), 2.23 – 2.05 (m, 2H) ppm. ^{13}C{^{1}H} NMR (101 MHz, MeOD) δ 175.1, 169.4, 166.3 (d, *J* = 250.5 Hz), 131.6 (d, *J* = 3.2 Hz), 131.2 (d, *J* = 9.1 Hz), 116.4 (d, *J* = 22.2 Hz), 52.9, 36.8, 21.8 ppm. ^{19}F NMR (377 MHz, MeOD) δ -110.3 (tt, *J* = 8.7, 5.2 Hz) ppm. HRMS(ESI) m/z calculated for $C_{11}H_{13}FNO_3S^+$ $[M + H]^+$ 258.0522, found: 258.0587.

(4-Chlorobenzoyl)homocysteine 2j

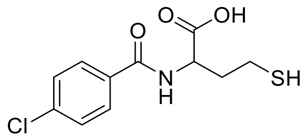

38 mg of **1j** (0.15 mmol) were hydrolysed. The corresponding acid was afforded as colourless oil (36 mg, 86% yield). ^{1}H NMR (400 MHz, MeOD) δ 7.83 (d, *J* = 8.5 Hz, 2H), 7.46 (d, *J* = 8.5 Hz, 2H), 4.81 – 4.75 (m, 1H), 2.70 – 2.52 (m, 2H), 2.30 – 2.05 (m, 2H) ppm. ^{13}C{^{1}H} NMR (101 MHz, MeOD) δ 175.1, 169.4, 138.9, 133.9, 130.2, 129.7, 52.9, 36.7, 21.8 ppm. HRMS(ESI) m/z calculated for $C_{11}H_{13}ClNO_3S^+$ $[M + H]^+$ 274.0226, found: 274.0298.

(2-Chlorobenzoyl)homocysteine 2k

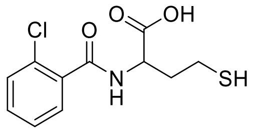

38 mg of **1k** (0.15 mmol) were hydrolysed. The corresponding acid was afforded as colourless oil (33 mg, 79% yield). ^{1}H NMR (400 MHz, MeOD) δ 7.48 – 7.30 (m, 4H), 4.77 (dd, *J* = 9.8, 4.6 Hz, 1H), 2.66 – 2.55 (m, 2H), 2.20 – 1.99 (m, 2H) ppm. ^{13}C{^{1}H} NMR (101 MHz, MeOD) δ 174.7, 170.1, 137.3, 132.2, 131.9, 131.0, 130.0, 128.1, 52.5, 36.8, 21.7 ppm. HRMS(ESI) m/z calculated for $C_{11}H_{13}ClNO_3S^+$ $[M + H]^+$ 274.0226, found: 274.0295.

(4-Methylbenzoyl)homocysteine 2l

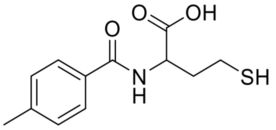

35 mg of **1l** (0.15 mmol) were hydrolysed. The corresponding acid was afforded as yellow oil (27 mg, 70% yield). ^{1}H NMR (400 MHz, MeOD) δ 7.73 (d, *J* = 8.1 Hz, 2H), 7.25 (d, *J* = 7.9 Hz, 2H), 4.82 – 4.74 (m, 1H), 2.68 – 2.50 (m, 2H), 2.36 (s, 3H), 2.25 – 2.05 (m, 2H) ppm. ^{13}C{^{1}H} NMR (101 MHz, MeOD) δ 175.2, 170.5, 143.6, 132.3, 130.1, 128.5, 52.8, 36.8, 21.8, 21.5 ppm. HRMS(ESI) m/z calculated for $C_{12}H_{16}NO_3S^+$ $[M + H]^+$ 254.0773, found: 254.0841.

(4-Methoxybenzoyl)homocysteine 2m

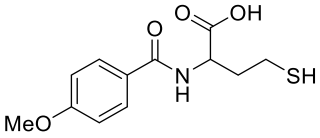

38 mg of **1m** (0.15 mmol) were hydrolysed. The corresponding acid was afforded as yellow oil (25 mg, 61% yield). ^{1}H NMR (400 MHz, MeOD) δ 7.87 – 7.77 (m, 2H), 7.01 – 6.90 (m, 2H), 4.81 – 4.73 (m, 1H), 3.83 (s, 3H), 2.70 – 2.52 (m, 2H), 2.26 – 2.06 (m, 2H) ppm. ^{13}C{^{1}H} NMR (101 MHz, MeOD) δ 175.3, 170.1, 164.1, 130.4, 130.4, 130.4, 127.2, 114.7, 55.9, 52.8, 36.8, 21.8 ppm. HRMS(ESI) m/z calculated for $C_{12}H_{16}NO_4S^+$ $[M + H]^+$ 270.0722, found: 270.0795.

3.3. General procedure for the synthesis of lactones 3

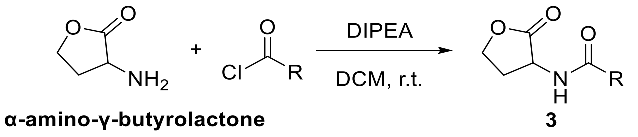

A mixture of α-amino-γ-butyrolactone hydrobromide (3 mmol, 546 mg) and DIPEA (6 mmol, 775 mg) in DCM was stirred at 0 °C under N_2. Appropriate acyl chloride (3.3 mmol) was added dropwise. The reaction mixture was allowed to warm up to room temperature and was stirred for another 3 hours. The reaction mixture was washed with sat. $NaHCO_3$ solution, water and brine were extracted by DCM (x3). The organic phase was dried over $MgSO_4$, filtered, and concentrated under vacuum. The residual was washed by Hexane/AcOEt 9:1, filtered and the residue was collected to afford compound **3**.

***N*-(2-Oxotetrahydrofuran-3-yl)benzamide 3a**[88]

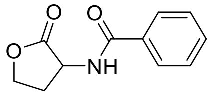

Benzoyl chloride (3.3 mmol, 464 mg) was used in the reaction. The product was afforded as white amorphous solid (362 mg, 59% yield). ^{1}H NMR (400 MHz, Chloroform-*d*) δ 7.87-7.75 (m, 2H), 7.58-7.50 (m, 1H), 7.48-7.41 (m, 2H), 6.74 (s, 1H), 4.74 (ddd, *J* = 11.6, 8.5, 5.5 Hz, 1H), 4.53 (td, *J* = 9.0, 1.2 Hz, 1H), 4.35 (ddd, *J* = 11.3, 9.3, 5.8 Hz, 1H), 3.03-2.93 (m, 1H), 2.35-2.19 (m, 1H) ppm. ^{13}C{^{1}H} NMR (101 MHz, $CDCl_3$) δ 175.7, 167.9, 133.1, 132.3, 128.9, 127.3, 66.4, 49.9, 30.9 ppm.

***N*-(2-Oxotetrahydrofuran-3-yl)hexanamide 3b**[92]

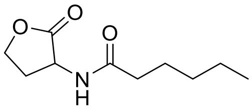

Hexanoyl chloride (3.3 mmol, 444 mg) was used in the reaction. The product was afforded as white amorphous solid (406 mg, 68% yield). ^{1}H NMR (400 MHz, D_2O) δ 4.70 – 4.61 (m, 1H), 4.56 (td, *J* = 9.3, 2.0 Hz, 1H), 4.40 (td, *J* = 9.8, 6.7 Hz, 1H), 2.69 – 2.57 (m, 1H), 2.41 – 2.25 (m, 3H), 1.61 (t, *J* = 7.4 Hz, 2H), 1.37 – 1.24 (m, 4H), 0.96 – 0.84 (m, 3H) ppm. ^{13}C{^{1}H} NMR (101 MHz, D_2O) δ 178.6, 177.4, 67.4, 49.1, 35.3, 30.4, 27.7, 24.8, 21.6, 13.1 ppm.

3.4. General procedure for the synthesis of racemic acids 4

The synthesis of acids **4** was initially attempted through hydrolysis of the lactones **3** with 1M NaOH. However, acids **4** spontaneously cyclise into lactones **3** during the work up of the reaction (extraction and drying processes). Thus, acids **4** were synthesised in D_2O and analysed by ^{1}H-NMR as crude products.

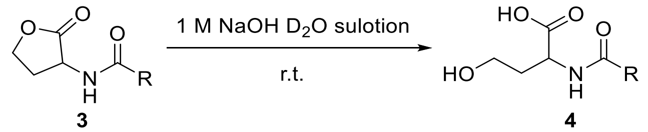

3 **4**

To a 1 M NaOH solution in D_2O, lactones **3** (0.15 mmol) were added. The reaction mixture was stirred vigorously at room temperature for 1h. Then, the reaction mixture was filtered into an NMR tube and directly used for ^{1}H NMR and HRMS characterization.

Benzoylhomoserine 4a

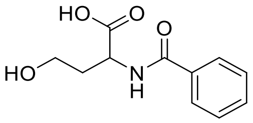

Product obtained in NaOH D_2O solution. ^1H NMR (400 MHz, D_2O) δ 3.16 – 3.02 (m, 2H), 2.88 – 2.79 (m, 1H) (proton signal integrated as 0.21 due to the proton exchange), 2.16 – 2.00 (m, 2H), 1.46 (dt, *J* = 13.8, 6.9 Hz, 1H), 1.32 (dt, *J* = 13.8, 7.1 Hz, 1H), 1.20 – 1.02 (m, 2H), 0.47 (t, *J* = 7.4 Hz, 3H) ppm. $^{13}C\{^1H\}$ NMR (101 MHz, D_2O) δ 179.3, 170.1, 133.3, 131.9, 128.6, 127.0, 58.4, 53.8, 34.4 ppm. HRMS(ESI) m/z calculated for $C_{11}H_{14}NO_4^+$ [M + H]$^+$ 224.0845, found: 224.0918.

Hexanoylhomoserine 4b

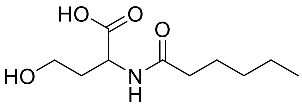

Product obtained in NaOH D_2O solution. ^1H NMR (400 MHz, D_2O) δ 3.64 (dd, *J* = 9.6, 4.3 Hz, 1H), 3.14 – 2.98 (m, 2H), 1.85 – 1.69 (m, 2H), 1.53 – 1.42 (m, 1H), 1.38 – 1.24 (m, 1H), 1.14 – 1.02 (m, 2H), 0.85 – 0.74 (m, 4H), 0.37 (t, *J* = 6.8 Hz, 3H) ppm. $^{13}C\{^1H\}$ NMR (101 MHz, D_2O) δ 179.3, 176.5, 58.2, 52.5, 35.4, 34.9, 30.2, 24.7, 21.4, 13.2 ppm. HRMS(ESI) m/z calculated for $C_{10}H_{20}NO_4^+$ [M + H]$^+$ 218.1314, found: 218.1388.

3.5. General procedure of the GcL biocatalysed EKR of thiolactones 1

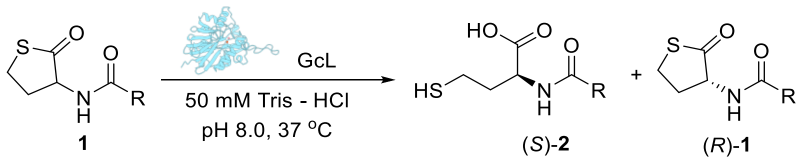

General procedure for GcL biocatalysed EKR of thiolactone **1**

50 µL of a 600 mM stock solution in DMSO of the relevant thiolactone **1** (30 mM final concentration), was added to 950 µL 50 mM Tris-HCl buffer pH 8.0 containing NaCl (200 mM) and GcL enzyme (40 µL pure enzyme) to initiate the reaction. The reaction was in shaken at 37 ºC.

Determination of conversions and ee

For substrates **1h, 1g** and **1k**

Upon completion, a 200 μL aliquot of the reaction mixture was spun down, acidified by HCl to pH 2-3 and then extracted 3 times with AcOEt (100 μL). AcOEt layer was analysed by normal phase HPLC using an appropriate chiral column to determine the ee of both thiolactone (*R*)-**1** and acid (*S*)-**2**.

For other substrates **1**

Upon completion, a 200 μL aliquot of the reaction mixture was spun down and extracted 5 times with AcOEt (100 μL). The combined AcOEt layer was analysed by normal phase HPLC using the appropriate chiral column to determine the enantiomeric excess of thiolactones (*R*)-**1**. DTBA (0.024 mmol) was added to the aqueous phase and incubated at 37 °C for 30 min to cleave potential disulfide side products. The aqueous phase was then acidified by HCl to pH 2-3 and extracted 3 times with AcOEt (100 μL), which was dried under vacuum and 100 μL $BF_3 \cdot EtO_2$ was added to catalyse the lactonization of the acid (*S*)-**2**. The mixture was shaken at room temperature for 5 minutes. Then 800 μL H_2O was added to quench the reaction. AcOEt (100 μL) was then added to extract the resulting thiolactone. The extraction was analysed by chiral HPLC to determine the ee of acid (*S*)-**2**.

Preparation of samples for optical rotation measurement

An 800 μL aliquot of the reaction mixture was extracted 5 times by AcOEt (500 μL). The collected AcOEt layer was collected and dried over $MgSO_4$ and evaporated under vacuum. Crude products were purified by flash column chromatography using an hexane:AcOEt (60:40) to afford the resulting pure enantioenriched thiolactones (*R*)-**1**, which were used for optical rotation measurement. DTBA (0.096 mmol) was added to the aqueous phase and incubated at 37 °C for 30 min to cleave the disulfide bond. The aqueous phase was then acidified by HCl to pH 2-3 and extracted 3 times by AcOEt (500 μL). The collected AcOEt layer was collected and dried over $MgSO_4$ and evaporated under vacuum. Crude products were loaded onto silica gel and elution with DCM:MeOH (95:5) afford the resulting pure enantioenriched thiocarboxylic acids (*S*)-**2**, which were used for optical rotation measurements.

3.6. Racemisation test of (*R*)-1a and (*S*)-2a

Preparation of enantioenriched thiolactone (*R*)-1a and thiocarboxylic acid (*S*)-2a

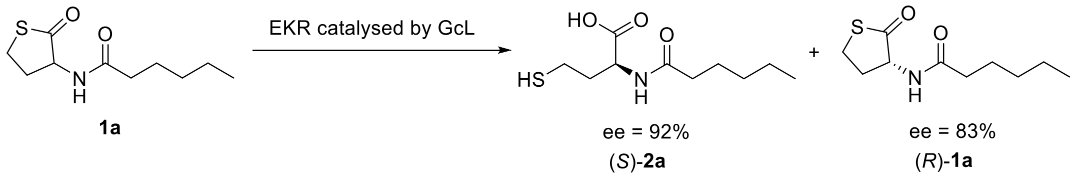

Enantioenriched thiocarboxylic acid (*S*)-**2a** (92% ee) and (*R*)-**1a** (83% ee) were prepared through EKR of **1a** catalysed by GcL as described in **3.5**.

Racemisation test

50 µL of a 600 mM stock solution of (*R*)-**1a** or (*S*)-**2a** in DMSO (30 mM final concentration), was added to 950 µL 50 mM Tris-HCl buffer pH 8.0 containing NaCl (200 mM) and shaken at 37 °C.

Both (*R*)-**1a** or (*S*)-**2a** proved to be configurationally stable over 24 h.

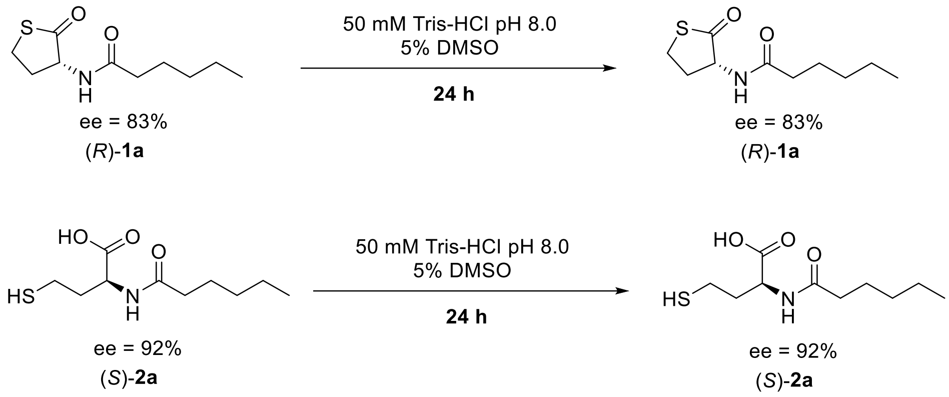

Scheme S1. Racemisation test of (*R*)-**1a** and (*S*)-**2a**

3.7. ^{1}H-NMR monitoring of the EKR reaction of thiolactone 1a catalysed by GcL

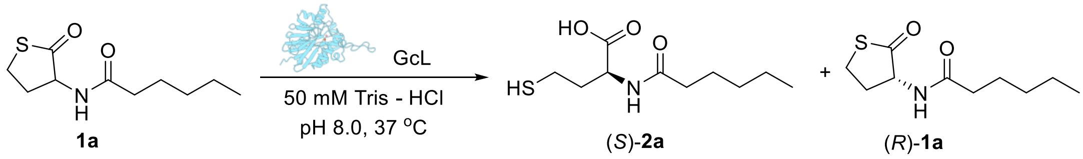

The EKR reaction of thiholactone **1a** was set up following the procedure as described in **3.5**. Upon completion, the reaction mixture was spun down and acidified to pH 2–3 using HCl, followed by extraction with AcOEt (3 × 100 µL). The organic layer was dried under vacuum, dissolved in deuterated methanol, and analyzed by ^{1}H NMR.

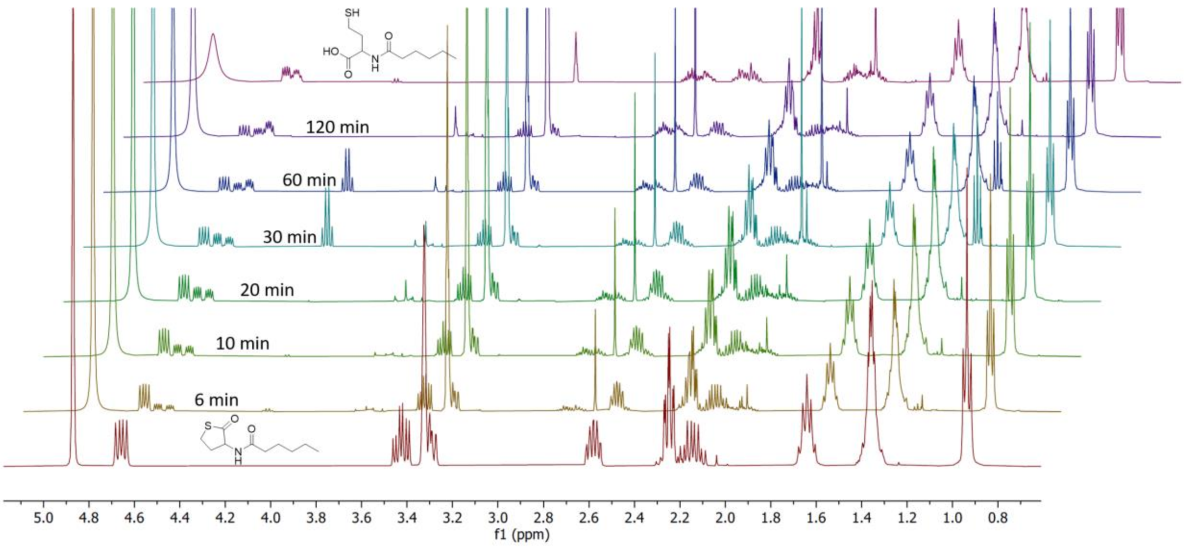

Figure S1. ^{1}H-NMR monitoring of EKR reaction on **1a** catalysed by GcL.

3.8. General procedure for the preparation of racemic lactones 8 and thiolactones 5

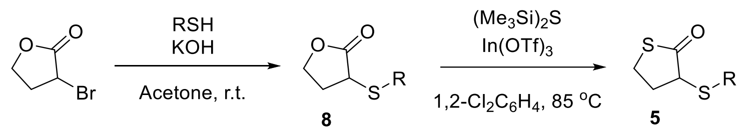

Synthesis of C3-thio-substituted-lactones **8**

The appropriate thiol (3.0 mmol) and KOH (6.0 mmol, 337 mg) were mixed and stirred in acetone. Then, α-bromo-γ-butyrolactone (3.3 mmol, 544 mg) was added to the mixture and the reaction was stirred until completion was observed from TLC. The reaction mixture was dissolved in water and extracted with AcOEt. The collected organic layer was dried over $MgSO_4$ and evaporated under vacuum. The crude products were purified by flash column chromatography (Hexane/AcOEt 9:1 to Hexane/AcOEt 6:4) to afford the resulting pure compounds **8**.

Synthesis of C3-thio-substituted-thiolactones **5**[93]

To a screw-capped tube, the appropriate lactone (1.0 mmol), $In(OTf)_3$ (0.1 mmol, 56 mg), $(Me_3Si)_2S$ (1.0 mmol, 178 mg) and 1.2-dichlorobenzene (2 mL) were added. After the tube was sealed, the reaction mixture was heated at 80 °C until the completion was observed from TLC. The reaction mixture was directly loaded onto silica gel and purified by flash column chromatography with Hexane:AcOEt (95:5) as eluent, affording the pure thiolactones **5**.

3-(Phenylthio)dihydrofuran-2(3*H*)-one 8a[83]

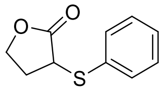

Thiophenol (3.0 mmol, 330 mg) was used in the reaction. The product was obtained as colourless oil (445 mg, 76% yield). ^{1}H NMR (500 MHz, $CDCl_3$) δ 7.60 – 7.52 (m, 2H), 7.39 – 7.31 (m, 3H), 4.30 – 4.18 (m, 2H), 3.86 (dd, *J* = 8.7, 6.2 Hz, 1H), 2.67 (dddd, *J* = 13.9, 8.6, 7.7, 6.6 Hz, 1H), 2.28 (ddt, *J* = 13.5, 7.5, 6.0 Hz, 1H) ppm. $^{13}C\{^1H\}$ NMR (126 MHz, $CDCl_3$) δ 175.1, 133.8, 131.9, 129.4, 128.9, 66.6, 44.6, 30.1 ppm.

3-(Phenylthio)dihydrothiophen-2(3*H*)-one 5a

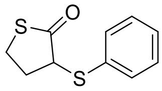

194 mg of **8a** was used in the reaction, affording the corresponding compound **5a** as yellow oil (166 mg, 79% yield). ^{1}H NMR (500 MHz, $CDCl_3$) δ 7.55 – 7.49 (m, 2H), 7.37 – 7.29 (m, 3H), 3.86 (t, *J* = 6.5 Hz, 1H), 3.48 – 3.40 (m, 1H), 3.34 – 3.25 (m, 1H), 2.65 – 2.54 (m, 1H), 2.35 – 2.25 (m, 1H) ppm. $^{13}C\{^1H\}$ NMR (126 MHz, $CDCl_3$) δ 204.6, 133.8, 132.1, 129.3, 128.7, 56.2, 32.6, 30.5 ppm. HRMS(ESI) m/z calculated for $C_{10}H_{11}OS_2^+$ $[M + H]^+$ 211.0173, found: 211.0244.

3-((4-Chlorophenyl)thio)dihydrofuran-2(3*H*)-one 8b[83]

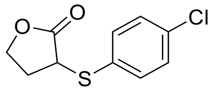

4-Chlorothiophenol (3.0 mmol, 434 mg) was used in the reaction. The product was obtained as white amorphous solid (459 mg, 67% yield). ^{1}H NMR (500 MHz, $CDCl_3$) δ 7.53 – 7.47 (m, 2H), 7.35 – 7.24 (m, 2H), 4.27 (t, *J* = 7.0 Hz, 2H), 3.82 (dd, *J* = 8.6, 6.2 Hz, 1H), 2.68 (ddt, *J* = 13.8, 8.5, 7.1 Hz, 1H), 2.30 – 2.19 (m, 1H) ppm. $^{13}C\{^1H\}$ NMR (126 MHz, $CDCl_3$) δ 174.8, 135.4, 135.2, 130.3, 129.6, 66.6, 44.6, 29.9 ppm.

3-((4-Chlorophenyl)thio)dihydrothiophen-2(3*H*)-one 5b

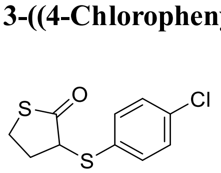

229 mg **8b** was used in the reaction, affording the corresponding compound **5b** as orange amorphous solid (152 mg, 62% yield). ^{1}H NMR (500 MHz, $CDCl_3$) δ 7.48 – 7.42 (m, 2H), 7.32 – 7.26 (m, 2H),

3.81 (t, J = 6.6 Hz, 1H), 3.48 – 3.37 (m, 1H), 3.31 (dt, J = 11.1, 6.1 Hz, 1H), 2.60 (dq, J = 13.5, 6.8 Hz, 1H), 2.28 (dq, J = 13.4, 6.1 Hz, 1H) ppm. $^{13}C\{^1H\}$ NMR (126 MHz, $CDCl_3$) δ 204.4, 135.2, 135.1, 130.5, 129.5, 56.2, 32.5, 30.5 ppm. HRMS(ESI) m/z calculated for $C_{10}H_{10}ClOS_2^+$ $[M + H]^+$ 244.9783, found: 244.9854.

3-((4-Bromophenyl)thio)dihydrofuran-2(3*H*)-one 8c[83]

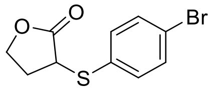

4-Bromothiophenol (3.0 mmol, 567 mg) was used in the reaction. The product was obtained as off-white amorphous solid (460 mg, 56% yield). ^1H NMR (500 MHz, $CDCl_3$) δ 7.49 – 7.45 (m, 2H), 7.45 – 7.41 (m, 2H), 4.28 (t, J = 6.9 Hz, 2H), 3.83 (dd, J = 8.6, 6.3 Hz, 1H), 2.69 (ddt, J = 13.8, 8.4, 7.1 Hz, 1H), 2.31 – 2.19 (m, 1H) ppm. $^{13}C\{^1H\}$ NMR (126 MHz, $CDCl_3$) δ 174.8, 135.3, 132.6, 131.0, 123.5, 66.6, 44.4, 30.0 ppm.

3-((4-Bromophenyl)thio)dihydrothiophen-2(3*H*)-one 5c

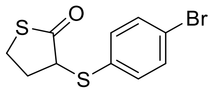

273 mg **8c** was used in the reaction, affording the corresponding compound **5c** as orange oil (197 mg, 68% yield). ^1H NMR (500 MHz, $CDCl_3$) δ 7.48 – 7.42 (m, 2H), 7.42 – 7.35 (m, 2H), 3.82 (t, J = 6.5 Hz, 1H), 3.44 (ddd, J = 11.1, 7.3, 6.1 Hz, 1H), 3.31 (dt, J = 11.1, 6.1 Hz, 1H), 2.60 (dq, J = 13.5, 6.7 Hz, 1H), 2.28 (dq, J = 13.3, 6.2 Hz, 1H) ppm. $^{13}C\{^1H\}$ NMR (126 MHz, $CDCl_3$) δ 204.3, 135.3, 132.4, 131.2, 123.2, 56.0, 32.5, 30.5 ppm. HRMS(ESI) m/z calculated for $C_{10}H_{10}BrOS_2^+$ $[M + H]^+$ 288.9278, found: 288.9346.

3-((4-Methoxyphenyl)thio)dihydrofuran-2(3*H*)-one 8d

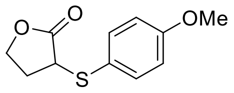

4-Methoxybenzenethiol (3.0 mmol, 421 mg) was used in the reaction. The product was obtained as yellow amorphous solid (380 mg, 56% yield). ^1H NMR (500 MHz, $CDCl_3$) δ 7.56 – 7.48 (m, 2H), 6.90 – 6.84 (m, 2H), 4.21 (ddd, J = 9.0, 7.9, 5.5 Hz, 1H), 4.13 (dt, J = 9.0, 7.2 Hz, 1H), 3.81 (s, 3H), 3.70

(dd, J = 8.7, 5.8 Hz, 1H), 2.68 – 2.57 (m, 1H), 2.30 – 2.20 (m, 1H) ppm. $^{13}C\{^1H\}$ NMR (126 MHz, $CDCl_3$) δ 175.3, 160.9, 137.1, 121.5, 115.0, 66.6, 55.5, 45.2, 29.8 ppm.

3-((4-Methoxyphenyl)thio)dihydrothiophen-2(3*H*)-one 5d

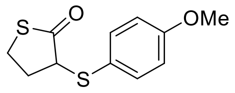

224 mg **8d** was used in the reaction, affording the corresponding compound **5d** as yellow oil (164 mg, 68% yield). ^1H NMR (500 MHz, $CDCl_3$) δ 7.51 – 7.44 (m, 2H), 6.89 – 6.82 (m, 2H), 3.80 (s, 3H), 3.70 (dd, J = 6.8, 5.8 Hz, 1H), 3.40 (ddd, J = 11.1, 7.6, 6.1 Hz, 1H), 3.26 (ddd, J = 11.1, 6.5, 5.5 Hz, 1H), 2.55 (ddt, J = 13.2, 7.6, 6.6 Hz, 1H), 2.27 (dq, J = 13.3, 5.8 Hz, 1H) ppm. $^{13}C\{^1H\}$ NMR (126 MHz, $CDCl_3$) δ 204.8, 160.6, 137.0, 121.9, 114.8, 56.8, 55.5, 32.4, 30.5 ppm. HRMS(ESI) m/z calculated for $C_{11}H_{13}O_2S_2^+$ $[M + H]^+$ 241.0279, found: 241.0345.

3-(p-Tolylthio)dihydrofuran-2(3*H*)-one 8e[93]

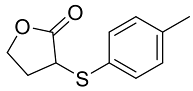

4-Methylbenzenethiol (3.0 mmol, 373 mg) was used in the reaction. The product was obtained as yellow oil (332 mg, 53% yield). ^1H NMR (500 MHz, $CDCl_3$) δ 7.49 – 7.43 (m, 2H), 7.15 (d, J = 7.9 Hz, 2H), 4.28 – 4.14 (m, 2H), 3.78 (dd, J = 8.7, 6.0 Hz, 1H), 2.70 – 2.59 (m, 1H), 2.35 (s, 3H), 2.31 – 2.21 (m, 1H) ppm. $^{13}C\{^1H\}$ NMR (126 MHz, $CDCl_3$) δ 175.2, 139.4, 134.5, 130.2, 127.9, 66.6, 44.8, 30.0, 21.4 ppm.

3-(p-Tolylthio)dihydrothiophen-2(3*H*)-one 5e

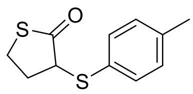

208 mg **8e** was used in the reaction, affording the corresponding compound **5e** as orange oil (121 mg, 54% yield). ^1H NMR (400 MHz, $CDCl_3$) δ 7.46 – 7.38 (m, 2H), 7.17 – 7.09 (m, 2H), 3.79 (t, J = 6.3 Hz, 1H), 3.48 – 3.37 (m, 1H), 3.32 – 3.22 (m, 1H), 2.63 – 2.50 (m, 1H), 2.34 (s, 3H), 2.32 – 2.24 (m, 1H) ppm. $^{13}C\{^1H\}$ NMR (101 MHz, $CDCl_3$) δ 204.7, 139.1, 134.5, 130.1, 128.2, 56.5, 32.5, 30.5, 21.4 ppm. HRMS(ESI) m/z calculated for $C_{11}H_{13}OS_2^+$ $[M + H]^+$ 225.0330, found: 225.0396.

3-((2-Fluorophenyl)thio)dihydrofuran-2(3*H*)-one 8f

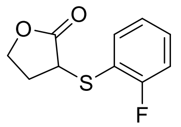

2-Fluorobenzenethiol (3.0 mmol, 386 mg) was used in the reaction. The product was obtained as pale yellow oil (524 mg, 82% yield). ^{1}H NMR (400 MHz, $CDCl_3$) δ 7.62 – 7.52 (m, 1H), 7.41 – 7.29 (m, 1H), 7.18 – 7.06 (m, 2H), 4.42 – 4.24 (m, 2H), 3.96 (dd, *J* = 8.5, 5.6 Hz, 1H), 2.73 – 2.60 (m, 1H), 2.32 – 2.19 (m, 1H) ppm. $^{13}C\{^1H\}$ NMR (101 MHz, $CDCl_3$) δ 174.5, 162.9 (d, *J* = 247.3 Hz), 136.3, 131.4 (d, *J* = 8.2 Hz), 125.0 (d, *J* = 3.7 Hz), 118.8 (d, *J* = 17.9 Hz), 116.1 (d, *J* = 22.8 Hz), 66.7, 43.1, 30.1 ppm. ^{19}F NMR (377 MHz, $CDCl_3$) δ -107.0 (ddd, *J* = 9.5, 7.2, 5.2 Hz) ppm.

3-((2-Fluorophenyl)thio)dihydrothiophen-2(3*H*)-one 5f

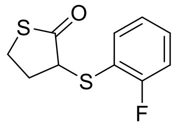

212 mg **8f** was used in the reaction, affording the corresponding compound **5f** as orange oil (83 mg, 36% yield). ^{1}H NMR (400 MHz, $CDCl_3$) δ 7.59 – 7.50 (m, 1H), 7.40 – 7.30 (m, 1H), 7.16 – 7.06 (m, 2H), 3.96 (t, *J* = 6.0 Hz, 1H), 3.54 (ddd, *J* = 11.4, 8.0, 6.0 Hz, 1H), 3.36 – 3.26 (m, 1H), 2.65 – 2.52 (m, 1H), 2.36 – 2.23 (m, 1H) ppm. $^{13}C\{^1H\}$ NMR (101 MHz, $CDCl_3$) δ 203.9, 163.0 (d, *J* = 247.4 Hz), 136.5, 131.3 (d, *J* = 8.0 Hz), 124.9 (d, *J* = 3.8 Hz), 118.9 (d, *J* = 18.0 Hz), 116.1 (d, *J* = 23.0 Hz), 54.7, 54.6, 32.5, 30.7 ppm. ^{19}F NMR (376 MHz, $CDCl_3$) δ -106.7 (td, *J* = 8.4, 5.6 Hz). HRMS(ESI) m/z calculated for $C_{10}H_{10}FOS_2^+$ $[M + H]^+$ 229.0079, found: 229.0149.

3-((2-Chlorophenyl)thio)dihydrofuran-2(3*H*)-one 8g

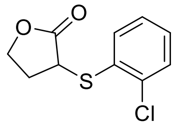

2-Chlorothiophenol (3.0 mmol, 434 mg) was used in the reaction. The product was obtained as yellow oil (401 mg, 58% yield). ^{1}H NMR (400 MHz, $CDCl_3$) δ 7.73 – 7.62 (m, 1H), 7.52 – 7.42 (m, 1H), 7.36 – 7.23 (m, 2H), 4.46 (dt, *J* = 9.1, 7.2 Hz, 1H), 4.37 (ddd, *J* = 9.1, 7.8, 5.2 Hz, 1H), 4.07 (dd, *J* = 8.4, 5.5 Hz, 1H), 2.80 – 2.67 (m, 1H), 2.37 – 2.25 (m, 1H) ppm. $^{13}C\{^1H\}$ NMR (101 MHz, $CDCl_3$) δ 174.5, 137.1, 134.6, 131.5, 130.2, 129.8, 127.7, 66.8, 42.8, 30.0 ppm.

3-((2-Chlorophenyl)thio)dihydrothiophen-2(3*H*)-one 5g

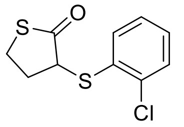

229 mg **8g** was used in the reaction, affording the corresponding compound **5g** as yellow oil (105 mg, 43% yield). ^{1}H NMR (400 MHz, $CDCl_3$) δ 7.59 – 7.49 (m, 1H), 7.41 – 7.30 (m, 1H), 7.24 – 7.12 (m, 2H), 3.97 (dd, *J* = 6.7, 5.4 Hz, 1H), 3.55 – 3.45 (m, 1H), 3.33 – 3.22 (m, 1H), 2.61 – 2.48 (m, 1H), 2.32 – 2.20 (m, 1H) ppm. ^{13}C{^{1}H} NMR (101 MHz, $CDCl_3$) δ 204.1, 137.4, 134.9, 131.5, 130.2, 129.8, 127.5, 54.3, 32.5, 30.2 ppm. HRMS(ESI) m/z calculated for $C_{10}H_{10}ClOS_2^+$ $[M + H]^+$ 244.9783, found: 244.9852.

3-((2-Bromophenyl)thio)dihydrofuran-2(3*H*)-one 8h

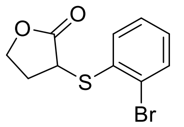

2-Bromothiophenol (3.0 mmol, 567 mg) was used in the reaction. The product was obtained as pale yellow oil (514 mg, 63% yield). ^{1}H NMR (400 MHz, $CDCl_3$) δ 7.69 – 7.57 (m, 2H), 7.35 – 7.24 (m, 1H), 7.22 – 7.12 (m, 1H), 4.47 – 4.38 (m, 1H), 4.38 – 4.30 (m, 1H), 4.05 (dd, *J* = 8.5, 5.6 Hz, 1H), 2.77 – 2.65 (m, 1H), 2.36 – 2.22 (m, 1H) ppm. ^{13}C{^{1}H} NMR (101 MHz, $CDCl_3$) δ 174.6, 133.9, 133.5, 129.7, 129.7, 128.3, 127.2, 66.8, 43.0, 30.0 ppm.

3-((2-Bromophenyl)thio)dihydrothiophen-2(3*H*)-one 5h

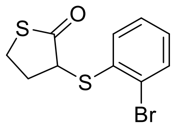

273 mg **8h** was used in the reaction, affording the corresponding compound **5h** as orange oil (200 mg, 69% yield). ^{1}H NMR (400 MHz, $CDCl_3$) δ 7.52 (dt, *J* = 7.8, 1.4 Hz, 2H), 7.20 (td, *J* = 7.6, 1.4 Hz, 1H), 7.07 (td, *J* = 7.7, 1.6 Hz, 1H), 3.97 (dd, *J* = 6.7, 5.7 Hz, 1H), 3.47 (ddd, *J* = 11.3, 7.8, 6.0 Hz, 1H), 3.26 (ddd, *J* = 11.4, 6.4, 5.4 Hz, 1H), 2.54 (ddt, *J* = 13.2, 7.8, 6.5 Hz, 1H), 2.25 (dq, *J* = 13.6, 5.7 Hz, 1H) ppm. ^{13}C{^{1}H} NMR (101 MHz, $CDCl_3$) δ 204.1, 134.2, 133.7, 133.4, 129.5, 128.1, 127.5, 54.6, 32.3, 30.7 ppm. HRMS(ESI) m/z calculated for $C_{10}H_{10}BrOS_2^+$ $[M + H]^+$ 288.9278, found: 288.9344.

3-((3,5-Dimethylphenyl)thio)dihydrofuran-2(3*H*)-one 8i

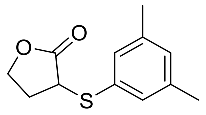

3,5-dimethylbenzenethiol (3.0 mmol, 415 mg) was used in the reaction. The product was obtained as colourless oil (453 mg, 68% yield). ^{1}H NMR (400 MHz, $CDCl_3$) δ 7.19 – 7.14 (m, 2H), 6.99 – 6.93 (m, 1H), 4.29 – 4.20 (m, 2H), 3.84 (dd, *J* = 8.6, 6.2 Hz, 1H), 2.72 – 2.59 (m, 1H), 2.34 – 2.21 (m, 8H) ppm. ^{13}C{^{1}H} NMR (101 MHz, $CDCl_3$) δ 175.2, 139.1, 131.3, 130.7, 66.6, 44.6, 30.3, 21.3 ppm.

3-((3,5-Dimethylphenyl)thio)dihydrothiophen-2(3*H*)-one 5i

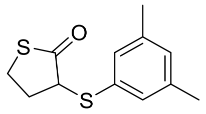

222 mg **8i** was used in the reaction, affording the corresponding compound **5i** as yellow oil (147 mg, 62% yield). ^{1}H NMR (400 MHz, $CDCl_3$) δ 7.16 – 7.11 (m, 2H), 6.96 – 6.91 (m, 1H), 3.84 (t, *J* = 6.4 Hz, 1H), 3.44 (ddd, *J* = 11.1, 7.3, 6.0 Hz, 1H), 3.29 (dt, *J* = 11.2, 6.1 Hz, 1H), 2.64 – 2.51 (m, 1H), 2.34 – 2.23 (m, 7H) ppm. ^{13}C{^{1}H} NMR (101 MHz, $CDCl_3$) δ 204.7, 138.9, 131.6, 131.3, 130.5, 56.3, 32.7, 30.5, 21.3 ppm. HRMS(ESI) m/z calculated for $C_{12}H_{15}OS_2^+$ $[M + H]^+$ 239.0486, found: 239.0555.

3-(Naphthalen-2-ylthio)dihydrofuran-2(3*H*)-one 8j

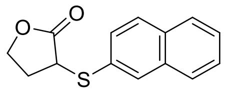

2-Naphthalenethiol (3.0 mmol, 481 mg) was used in the reaction. The product was obtained as off-white amorphous solid (443 mg, 60% yield). ^{1}H NMR (500 MHz, $CDCl_3$) δ 8.06 (d, *J* = 1.8 Hz, 1H), 7.86 – 7.78 (m, 3H), 7.60 (dd, *J* = 8.6, 1.8 Hz, 1H), 7.55 – 7.48 (m, 2H), 4.31 – 4.21 (m, 2H), 3.96 (dd, *J* = 8.6, 6.3 Hz, 1H), 2.69 (ddt, *J* = 13.8, 8.6, 6.9 Hz, 1H), 2.32 (dq, *J* = 13.4, 6.6 Hz, 1H) ppm. ^{13}C{^{1}H} NMR (126 MHz, $CDCl_3$) δ 175.1, 133.7, 133.2, 133.1, 130.3, 129.2, 129.1, 127.9, 127.9, 127.0, 126.9, 66.7, 44.6, 30.1 ppm.

3-(Naphthalen-2-ylthio)dihydrothiophen-2(3*H*)-one 5j

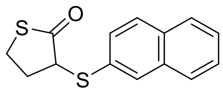

244 mg **8j** was used in the reaction, affording the corresponding compound **5j** as pale yellow amorphous solid (122 mg, 47% yield). ^{1}H NMR (500 MHz, $CDCl_3$) δ 8.03 (d, *J* = 1.8 Hz, 1H), 7.85 – 7.77 (m, 3H), 7.56 (dd, *J* = 8.5, 1.9 Hz, 1H), 7.54 – 7.46 (m, 2H), 3.97 (t, *J* = 6.5 Hz, 1H), 3.46 (ddd, *J* = 11.2, 7.3, 6.1 Hz, 1H), 3.31 (dt, *J* = 11.1, 6.1 Hz, 1H), 2.62 (dq, *J* = 13.5, 6.6 Hz, 1H), 2.34 (dq, *J* = 13.4, 6.1 Hz, 1H) ppm. ^{13}C{^{1}H} NMR (126 MHz, $CDCl_3$) δ 204.6, 133.7, 133.1, 133.0, 130.4, 129.4, 128.9, 127.9, 127.8, 126.9, 126.8, 56.3, 32.7, 30.5 ppm. HRMS(ESI) m/z calculated for $C_{14}H_{13}OS_2^+$ $[M + H]^+$ 261.0330, found: 261.0398.

3-(Propylthio)dihydrofuran-2(3*H*)-one 8k

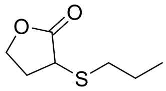

1-Propanethiol (3.0 mmol, 228 mg) was used in the reaction. The product was obtained as colourless oil (388 mg, 81% yield). ^{1}H NMR (400 MHz, $CDCl_3$) δ 4.48 – 4.38 (m, 1H), 4.32 (td, *J* = 8.5, 3.9 Hz, 1H), 3.50 (dd, *J* = 8.4, 4.1 Hz, 1H), 2.83 (ddd, *J* = 12.6, 8.1, 6.1 Hz, 1H), 2.72 – 2.58 (m, 2H), 2.17 – 2.06 (m, 1H), 1.78 – 1.56 (m, 2H), 1.00 (t, *J* = 7.3 Hz, 3H) ppm. ^{13}C{^{1}H} NMR (101 MHz, $CDCl_3$) δ 175.6, 66.9, 39.2, 33.4, 30.2, 22.5, 13.5 ppm. HRMS(ESI) m/z calculated for $C_7H_{13}O_2S^+$ $[M + H]^+$ 161.0558, found: 161.0627.

3-(Propylthio)dihydrothiophen-2(3*H*)-one 5k

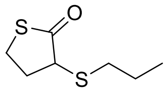

160 mg **8k** was used in the reaction, affording the corresponding compound **5k** as orange oil (108 mg, 61% yield). ^{1}H NMR (500 MHz, $CDCl_3$) δ 3.57 (ddd, *J* = 10.9, 9.5, 5.7 Hz, 1H), 3.48 (dd, *J* = 6.8, 3.8 Hz, 1H), 3.28 (ddd, *J* = 10.8, 6.6, 3.7 Hz, 1H), 2.74 (ddd, *J* = 12.4, 8.2, 6.0 Hz, 1H), 2.65 – 2.48 (m, 2H), 2.21 (ddt, *J* = 13.4, 5.7, 3.8 Hz, 1H), 1.74 – 1.56 (m, 3H), 0.99 (t, *J* = 7.3 Hz, 3H) ppm. ^{13}C{^{1}H} NMR (126 MHz, $CDCl_3$) δ 205.4, 51.1, 33.1, 32.9, 31.1, 22.7, 13.6 ppm. HRMS(ESI) m/z calculated for $C_7H_{13}OS_2^+$ $[M + H]^+$ 177.0330, found: 177.0400.

3-(Isobutylthio)dihydrofuran-2(3*H*)-one 8l

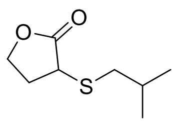

2-Methyl-1-propanethiol (3.0 mmol, 271 mg) was used in the reaction. The product was obtained as yellow oil (385 mg, 74% yield). ^{1}H NMR (500 MHz, $CDCl_3$) δ 4.48 – 4.39 (m, 1H), 4.32 (ddd, J = 8.9, 8.1, 3.9 Hz, 1H), 3.47 (dd, J = 8.4, 4.1 Hz, 1H), 2.74 (dd, J = 12.4, 6.3 Hz, 1H), 2.71 – 2.61 (m, 1H), 2.59 (dd, J = 12.4, 7.4 Hz, 1H), 2.17 – 2.06 (m, 1H), 1.94 – 1.80 (m, 1H), 1.01 (d, J = 6.6 Hz, 6H) ppm. ^{13}C{^{1}H} NMR (126 MHz, $CDCl_3$) δ 175.6, 66.9, 40.2, 39.6, 30.3, 28.4, 22.3, 21.9 ppm.

3-(Isobutylthio)dihydrothiophen-2(3*H*)-one 5l

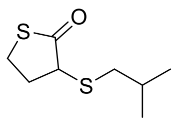

174 mg **8l** was used in the reaction, affording the corresponding compound **5l** as yellow oil (156 mg, 82% yield). ^{1}H NMR (500 MHz, $CDCl_3$) δ 3.58 (ddd, J = 11.0, 9.5, 5.7 Hz, 1H), 3.45 (dd, J = 6.8, 3.7 Hz, 1H), 3.28 (ddd, J = 11.0, 6.6, 3.7 Hz, 1H), 2.65 (dd, J = 12.5, 6.3 Hz, 1H), 2.60 – 2.52 (m, 2H), 2.26 – 2.17 (m, 1H), 1.89 – 1.76 (m, 1H), 0.99 (d, J = 6.6 Hz, 5H) ppm. ^{13}C{^{1}H} NMR (126 MHz, $CDCl_3$) δ 205.5, 51.5, 39.9, 33.0, 31.1, 28.5, 22.3, 21.9 ppm. HRMS(ESI) m/z calculated for $C_8H_{15}OS_2^+$ $[M + H]^+$ 191.0486, found: 191.0553.

3-(Cyclohexylthio)dihydrofuran-2(3*H*)-one 8m

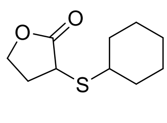

Cyclohexanethiol (3.0 mmol, 349 mg) was used in the reaction. The product was obtained as colourless oil (398 mg, 66% yield). ^{1}H NMR (500 MHz, $CDCl_3$) δ 4.43 (td, J = 8.7, 6.9 Hz, 1H), 4.32 (td, J = 8.5, 3.9 Hz, 1H), 3.59 (dd, J = 8.3, 4.1 Hz, 1H), 3.09 (tt, J = 10.4, 3.7 Hz, 1H), 2.65 (dq, J = 13.4, 8.3 Hz, 1H), 2.17 – 2.02 (m, 2H), 1.97 – 1.88 (m, 1H), 1.83 – 1.70 (m, 2H), 1.66 – 1.58 (m, 1H), 1.44 – 1.19 (m, 5H) ppm. ^{13}C{^{1}H} NMR (126 MHz, $CDCl_3$) δ 175.8, 66.9, 43.3, 37.9, 33.7, 33.0, 30.6, 26.1, 25.9 ppm.

3-(Cyclohexylthio)dihydrothiophen-2(3*H*)-one 5m

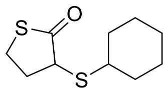

200 mg **8m** was used in the reaction, affording the corresponding compound **5m** as yellow oil (106 mg, 49% yield). ^{1}H NMR (500 MHz, $CDCl_3$) δ 3.61 – 3.53 (m, 2H), 3.31 – 3.23 (m, 1H), 2.97 (tt, *J* = 10.5, 3.8 Hz, 1H), 2.55 (ddt, *J* = 13.4, 9.6, 6.7 Hz, 1H), 2.18 (ddt, *J* = 13.3, 5.6, 3.7 Hz, 1H), 2.09 (dt, *J* = 12.4, 4.4 Hz, 1H), 1.96 – 1.85 (m, 1H), 1.82 – 1.68 (m, 2H), 1.65 – 1.56 (m, 1H), 1.40 – 1.18 (m, 5H) ppm. ^{13}C{^{1}H} NMR (126 MHz, $CDCl_3$) δ 205.8, 49.6, 42.9, 33.8, 33.2, 33.1, 31.2, 26.1, 25.9 ppm. HRMS(ESI) m/z calculated for $C_{10}H_{17}OS_2^+$ $[M + H]^+$ 217.0643, found: 217.0715.

3-(Benzylthio)dihydrofuran-2(3*H*)-one 8n[94]

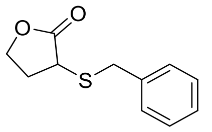

Phenylmethanethiol (3.0 mmol, 373 mg) was used in the reaction. The product was obtained as colourless oil (356 mg, 57% yield). ^{1}H NMR (500 MHz, $CDCl_3$) δ 7.41 – 7.35 (m, 2H), 7.35 – 7.29 (m, 2H), 7.32 – 7.20 (m, 1H), 4.40 – 4.29 (m, 1H), 4.27 – 4.19 (m, 1H), 4.08 (d, *J* = 13.4 Hz, 1H), 3.80 (d, *J* = 13.4 Hz, 1H), 3.32 – 3.25 (m, 1H), 2.56 – 2.42 (m, 1H), 2.05 – 1.93 (m, 1H) ppm. ^{13}C{^{1}H} NMR (126 MHz, $CDCl_3$) δ 175.4, 137.0, 129.1, 128.5, 127.3, 66.7, 37.8, 35.1, 29.5 ppm.

3-(Benzylthio)dihydrothiophen-2(3*H*)-one 5n

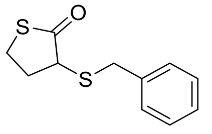

208 mg **8n** was used in the reaction, affording the corresponding compound **5n** as orange oil (133 mg, 59% yield). ^{1}H NMR (500 MHz, $CDCl_3$) δ 7.39 – 7.35 (m, 2H), 7.35 – 7.29 (m, 2H), 7.29 – 7.23 (m, 1H), 3.98 (d, *J* = 13.4 Hz, 1H), 3.80 (d, *J* = 13.4 Hz, 1H), 3.52 (ddd, *J* = 11.2, 9.3, 5.8 Hz, 1H), 3.32 (dd, *J* = 6.9, 4.0 Hz, 1H), 3.25 (ddd, *J* = 10.9, 6.6, 3.9 Hz, 1H), 2.46 (ddt, *J* = 13.6, 9.4, 6.8 Hz, 1H), 2.12 (ddt, *J* = 13.7, 5.8, 4.0 Hz, 1H) ppm. ^{13}C{^{1}H} NMR (126 MHz, $CDCl_3$) δ 205.4, 137.3, 129.4, 128.7, 127.4, 49.6, 35.0, 32.6, 31.2 ppm. HRMS(ESI) m/z calculated for $C_{11}H_{13}OS_2^+$ $[M + H]^+$ 225.0330, found: 225.0403.

3-(Phenethylthio)dihydrofuran-2(3*H*)-one 8o

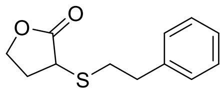

2-Phenylethanethiol (3.0 mmol, 415 mg) was used in the reaction. The product was obtained as colourless oil (402 mg, 60% yield). ^{1}H NMR (500 MHz, $CDCl_3$) δ 7.33 – 7.28 (m, 2H), 7.25 – 7.19 (m, 3H), 4.42 (td, J = 8.7, 6.9 Hz, 1H), 4.31 (td, J = 8.6, 3.9 Hz, 1H), 3.48 (dd, J = 8.4, 4.1 Hz, 1H), 3.18 – 3.07 (m, 1H), 3.04 – 2.87 (m, 3H), 2.63 (dq, J = 13.4, 8.3 Hz, 1H), 2.09 (ddt, J = 13.4, 6.8, 4.0 Hz, 1H) ppm. ^{13}C{^{1}H} NMR (126 MHz, $CDCl_3$) δ 175.4, 140.0, 128.7, 128.7, 126.7, 66.9, 39.2, 35.7, 32.8, 30.1 ppm.

3-(Phenethylthio)dihydrothiophen-2(3*H*)-one 5o

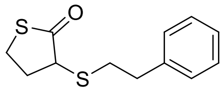

222 mg **8o** was used in the reaction, affording the corresponding compound **5o** as orange oil (126 mg, 53% yield). ^{1}H NMR (500 MHz, $CDCl_3$) δ 7.33 – 7.27 (m, 2H), 7.25 – 7.18 (m, 3H), 3.56 (ddd, J = 11.1, 9.5, 5.7 Hz, 1H), 3.49 (dd, J = 6.7, 3.8 Hz, 1H), 3.27 (ddd, J = 10.9, 6.6, 3.7 Hz, 1H), 3.05 – 2.97 (m, 1H), 2.97 – 2.86 (m, 3H), 2.54 (ddt, J = 13.5, 9.5, 6.7 Hz, 1H), 2.19 (ddt, J = 13.4, 5.7, 3.7 Hz, 1H) ppm. ^{13}C{^{1}H} NMR (126 MHz, $CDCl_3$) δ 205.3, 140.1, 128.7, 128.6, 126.6, 51.0, 35.9, 32.8, 32.4, 31.1 ppm. HRMS(ESI) m/z calculated for $C_{12}H_{15}OS_2^+$ $[M + H]^+$ 239.04861, found: 239.0553.

3-((Furan-2-ylmethyl)thio)dihydrofuran-2(3*H*)-one 8p

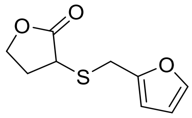

Furan-2-ylmethanethiol (3.0 mmol, 342 mg) was used in the reaction. The product was obtained as colourless oil (397 mg, 67% yield). ^{1}H NMR (400 MHz, $CDCl_3$) δ 7.41 – 7.36 (m, 1H), 6.35 – 6.27 (m, 2H), 4.44 – 4.36 (m, 1H), 4.35 – 4.27 (m, 1H), 4.22 (dd, J = 14.9, 1.9 Hz, 1H), 3.80 (d, J = 14.9 Hz, 1H), 3.45 (dd, J = 8.6, 4.8 Hz, 1H), 2.67 – 2.53 (m, 1H), 2.12 – 1.99 (m, 1H) ppm. ^{13}C{^{1}H} NMR (101 MHz, $CDCl_3$) δ 175.5, 150.1, 142.7, 110.6, 108.9, 66.9, 38.2, 29.6, 27.6 ppm.

3-((Furan-2-ylmethyl)thio)dihydrothiophen-2(3*H*)-one 5p

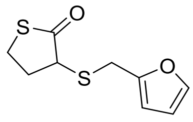

198 mg **8p** was used in the reaction, affording the corresponding compound **5p** as yellow oil (115 mg, 54% yield). ^{1}H NMR (400 MHz, $CDCl_3$) δ 7.37 (dd, *J* = 1.9, 0.9 Hz, 1H), 6.31 (dd, *J* = 3.2, 1.9 Hz, 1H), 6.27 (dd, *J* = 3.2, 0.8 Hz, 1H), 4.08 (d, *J* = 14.8 Hz, 1H), 3.78 (d, *J* = 14.8 Hz, 1H), 3.52 (ddd, *J* = 11.2, 9.1, 5.9 Hz, 1H), 3.45 (dd, *J* = 6.9, 4.3 Hz, 1H), 3.28 (ddd, *J* = 11.0, 6.6, 4.2 Hz, 1H), 2.52 (ddt, *J* = 13.6, 9.1, 6.8 Hz, 1H), 2.15 (ddt, *J* = 13.6, 5.9, 4.2 Hz, 1H) ppm. $^{13}C\{^1H\}$ NMR (101 MHz, $CDCl_3$) δ 205.3, 150.3, 142.7, 110.6, 108.8, 50.0, 32.5, 31.2, 27.3 ppm. HRMS(ESI) m/z calculated for $C_9H_{11}O_2S_2^+$ $[M + H]^+$ 215.0122, found: 215.0189.

3.9. General procedure for the preparation of racemic thiocarboxylic acids 6

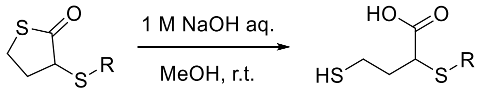

To a solution of appropriate thiolactone **5** (0.15 mmol) in MeOH (1 mL), 1M NaOH aq. (1 mL) was added. The reaction mixture was stirred vigorously at room temperature for 1h. Then, MeOH was removed under reduced pressure. The reaction mixture was extracted by AcOEt. The aqueous phase was acidified by 2M HCl aq. to pH 2-3 and extracted by AcOEt. The organic phase was dried over $MgSO_4$, filtered, and concentrated under reduced pressure. Then the mixture was loaded onto silica gel and eluted with DCM:MeOH (95:5) affording the pure thiocarboxylic acid **6**.

4-Mercapto-2-(phenylthio)butanoic acid 6a

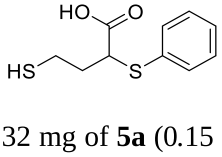

32 mg of **5a** (0.15 mmol) was hydrolysed, affording the corresponding acid as yellow oil (27 mg, 78% yield). ^{1}H NMR (500 MHz, $CDCl_3$) δ 7.55 – 7.45 (m, 2H), 7.37 – 7.29 (m, 3H), 3.89 (t, *J* = 7.5 Hz, 1H), 2.88 – 2.63 (m, 2H), 2.20 – 2.11 (m, 1H), 2.10 – 2.01 (m, 1H), 1.38 (t, *J* = 8.3 Hz, 1H) ppm. $^{13}C\{^1H\}$ NMR (126 MHz, $CDCl_3$) δ 177.4, 133.3, 132.5, 129.3, 128.6, 48.9, 35.1, 22.1 ppm. HRMS (ESI) m/z calculated for $C_{10}H_{13}O_2S_2^+$ $[M + H]^+$ 229.0279, found 229.0087.

2-((4-Chlorophenyl)thio)-4-mercaptobutanoic acid 6b

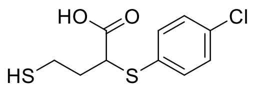

37 mg of **5b** (0.15 mmol) was hydrolysed, affording the corresponding acid as pale yellow amorphous solid (30 mg, 75% yield). ^{1}H NMR (400 MHz, MeOD) δ 7.53 – 7.43 (m, 2H), 7.38 – 7.29 (m, 2H), 3.88 (t, *J* = 7.4 Hz, 1H), 2.73 – 2.51 (m, 2H), 2.17 – 2.04 (m, 1H), 2.05 – 1.91 (m, 1H) ppm. ^{13}C{^{1}H} NMR (101 MHz, MeOD) δ 180.6, 135.2, 135.1, 133.8, 130.1, 50.8, 36.9, 22.5 ppm. HRMS(ESI) m/z calculated for $C_{10}H_{12}ClO_2S_2^+$ [M + H]$^+$ 262.9889, found: 262.9768.

2-((4-Bromophenyl)thio)-4-mercaptobutanoic acid 6c

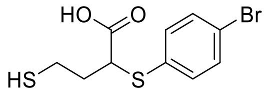

43 mg of **5c** (0.15 mmol) was hydrolysed, affording the corresponding acid as pale yellow amorphous solid (29 mg, 62% yield).^{1}H NMR (400 MHz, CDCl$_3$) δ 7.50 – 7.40 (m, 2H), 7.40 – 7.25 (m, 2H), 3.87 (t, *J* = 7.4 Hz, 1H), 2.83 – 2.63 (m, 2H), 2.22 – 1.97 (m, 2H), 1.38 (t, *J* = 8.3 Hz, 1H) ppm. ^{13}C{^{1}H} NMR (101 MHz, CDCl$_3$) δ 177.1, 134.8, 132.5, 131.7, 123.1, 48.9, 34.9, 22.0 ppm. HRMS(ESI) m/z calculated for $C_{10}H_{12}BrO_2S_2^+$ [M + H]$^+$ 306.9384, found: 306.9276.

4-Mercapto-2-((4-methoxyphenyl)thio)butanoic acid 6d

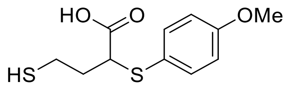

36 mg of **5d** (0.15 mmol) was hydrolysed, affording the corresponding acid as orange oil (29 mg, 74% yield). ^{1}H NMR (400 MHz, MeOD) δ 7.48 – 7.39 (m, 2H), 6.93 – 6.84 (m, 2H), 3.79 (s, 3H), 3.68 (t, *J* = 7.4 Hz, 1H), 2.73 – 2.55 (m, 2H), 2.18 – 1.85 (m, 2H) ppm. ^{13}C{^{1}H} NMR (101 MHz, MeOD) δ 175.4, 161.9, 137.4, 124.1, 115.6, 55.8, 51.1, 36.6, 22.4 ppm. HRMS(ESI) m/z calculated for $C_{11}H_{15}O_3S_2^+$ [M + H]$^+$ 259.0384, found: 259.0260.

4-Mercapto-2-(p-tolylthio)butanoic acid 6e

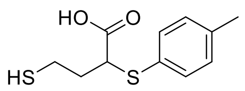

34 mg of **5e** (0.15 mmol) was hydrolysed, affording the corresponding acid as pale yellow amorphous solid (31 mg, 84% yield). ^{1}H NMR (400 MHz, CDCl$_3$) δ 7.39 (d, *J* = 8.0 Hz, 2H), 7.14 (d, *J* = 7.7 Hz, 2H), 3.81 (t, *J* = 7.4 Hz, 1H), 2.87 – 2.61 (m, 2H), 2.34 (s, 4H), 2.19 – 1.95 (m, 2H), 1.37 (t, *J* = 8.3 Hz, 1H) ppm. ^{13}C{^{1}H} NMR (101 MHz, CDCl$_3$) δ 175.3, 139.1, 134.1, 130.1, 128.5, 49.2, 35.0, 22.1, 21.4 ppm. HRMS(ESI) m/z calculated for $C_{11}H_{13}O_2S_2^-$ [M - H]$^-$ 241.0435, found: 241.0359.

2-((2-Fluorophenyl)thio)-4-mercaptobutanoic acid 6f

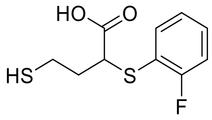

34 mg of **5f** (0.15 mmol) was hydrolysed, affording the corresponding acid as pale yellow oil (28 mg, 75% yield). ^{1}H NMR (400 MHz, MeOD) δ 7.59 – 7.50 (m, 1H), 7.43 – 7.33 (m, 1H), 7.20 – 7.09 (m, 2H), 3.89 (t, *J* = 7.4 Hz, 1H), 2.75 – 2.58 (m, 2H), 2.16 – 2.04 (m, 1H), 2.04 – 1.91 (m, 1H) ppm. ^{13}C{^{1}H} NMR (101 MHz, MeOD) δ 174.8, 164.0 (d, *J* = 245.8 Hz), 131.9 (d, *J* = 8.1 Hz), 125.8 (d, *J* = 3.9 Hz), 121.2 (d, *J* = 18.0 Hz), 116.8 (d, *J* = 23.2 Hz), 49.6, 36.8, 22.4 ppm. ^{19}F NMR (377 MHz, MeOD) δ -108.9 (ddd, *J* = 9.8, 7.3, 5.0 Hz) ppm. HRMS(ESI) m/z calculated for $C_{10}H_{12}FO_2S_2^+$ [M + H]$^+$ 247.0185, found: 247.0047.

2-((2-Chlorophenyl)thio)-4-mercaptobutanoic acid 6g

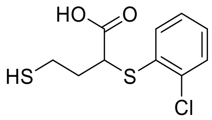

37 mg of **5g** (0.15 mmol) was hydrolysed, affording the corresponding acid as pale yellow oil (30 mg, 75% yield). ^{1}H NMR (400 MHz, $CDCl_3$) δ 7.62 – 7.50 (m, 1H), 7.48 – 7.38 (m, 1H), 7.30 – 7.19 (m, 3H), 4.01 (t, *J* = 7.3 Hz, 1H), 2.87 – 2.62 (m, 2H), 2.31 – 1.98 (m, 2H), 1.47 – 1.31 (m, 1H) ppm. ^{13}C{^{1}H} NMR (101 MHz, $CDCl_3$) δ 176.8, 137.0, 134.2, 132.1, 130.3, 129.6, 127.6, 47.9, 35.2, 22.1 ppm. HRMS(ESI) m/z calculated for $C_{10}H_{12}ClO_2S_2^+$ [M + H]$^+$ 262.9889, found: 262.9767.

2-((2-Bromophenyl)thio)-4-mercaptobutanoic acid 6h

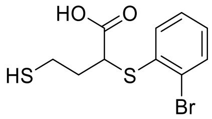

43 mg of **5h** (0.15 mmol) was hydrolysed, affording the corresponding acid as pale yellow amorphous solid (34 mg, 73% yield). ^{1}H NMR (400 MHz, $CDCl_3$) δ 7.65 – 7.53 (m, 2H), 7.33 – 7.22 (m, 1H), 7.15 (td, *J* = 7.7, 1.6 Hz, 1H), 4.02 (t, *J* = 7.4 Hz, 1H), 2.88 – 2.66 (m, 2H), 2.33 – 2.07 (m, 2H), 1.43 (t, *J* = 8.3 Hz, 1H) ppm. ^{13}C{^{1}H} NMR (101 MHz, $CDCl_3$) δ 177.1, 134.4, 133.6, 129.5, 128.3, 127.3, 48.2, 35.1, 22.2 ppm. HRMS(ESI) m/z calculated for $C_{10}H_{12}BrO_2S_2^+$ [M + H]$^+$ 306.9384, found: 306.9270.

2-((3,5-Dimethylphenyl)thio)-4-mercaptobutanoic acid 6i

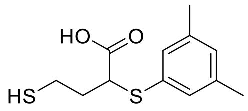

36 mg of **5i** (0.15 mmol) was hydrolysed, affording the corresponding acid as pale yellow amorphous solid (34 mg, 87% yield). ^{1}H NMR (400 MHz, $CDCl_3$) δ 7.10 (s, 2H), 6.93 (s, 1H), 3.86 (t, *J* = 7.4 Hz, 1H), 2.83 – 2.63 (m, 2H), 2.29 (s, 6H), 2.22 – 1.96 (m, 2H), 1.38 (t, *J* = 8.3 Hz, 1H) ppm. $^{13}C\{^{1}H\}$ NMR (101 MHz, $CDCl_3$) δ 177.7, 139.0, 132.1, 130.7, 130.4, 49.1, 35.2, 22.1, 21.3 ppm. HRMS(ESI) m/z calculated for $C_{12}H_{17}O_2S_2^+$ [M + H]$^+$ 257.0592, found: 257.0465.

4-Mercapto-2-(naphthalen-2-ylthio)butanoic acid 6j

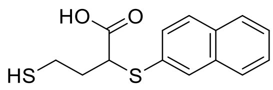

39 mg of **5j** (0.15 mmol) was hydrolysed, affording the corresponding acid as pale yellow oil (30 mg, 71% yield). ^{1}H NMR (400 MHz, MeOD) δ 8.02 – 7.97 (m, 1H), 7.88 – 7.77 (m, 3H), 7.58 – 7.42 (m, 3H), 4.01 (t, *J* = 7.4 Hz, 1H), 2.84 – 2.60 (m, 2H), 2.24 – 1.97 (m, 2H) ppm. $^{13}C\{^{1}H\}$ NMR (101 MHz, MeOD) δ 175.5, 135.1, 134.1, 132.5, 132.2, 130.7, 129.6, 128.7, 128.5, 127.7, 127.6, 50.4, 37.0, 22.5 ppm. HRMS(ESI) m/z calculated for $C_{14}H_{15}O_2S_2^+$ [M + H]$^+$ 279.0435, found: 279.1590.

4-Mercapto-2-(propylthio)butanoic acid 6k

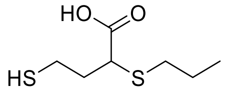

26 mg of **5k** (0.15 mmol) was hydrolysed, affording the corresponding acid as colourless oil (19 mg, 64% yield). ^{1}H NMR (500 MHz, $CDCl_3$) δ 3.50 (t, *J* = 7.5 Hz, 1H), 2.74 – 2.52 (m, 4H), 2.14 (dq, *J* = 14.4, 7.2 Hz, 1H), 1.98 (dq, *J* = 14.3, 7.1 Hz, 1H), 1.63 (tdd, *J* = 21.2, 10.7, 5.1 Hz, 2H), 1.41 (t, *J* = 8.3 Hz, 1H), 1.00 (t, *J* = 7.2 Hz, 3H) ppm. $^{13}C\{^{1}H\}$ NMR (126 MHz, $CDCl_3$) δ 178.2, 34.9, 33.8, 29.8, 22.8, 22.2, 13.6 ppm. HRMS(ESI) m/z calculated for $C_7H_{15}O_2S_2^+$ [M + H]$^+$ 195.0435, found: 195.0305.

2-(Isobutylthio)-4-mercaptobutanoic acid 6l

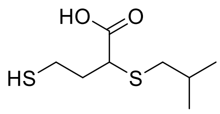

29 mg of **5l** (0.15 mmol) was hydrolysed, affording the corresponding acid as colourless oil (22 mg, 69% yield). ^{1}H NMR (500 MHz, MeOD) δ 3.46 – 3.35 (m, 1H), 2.81 (t, J = 7.2 Hz, 1H), 2.66 – 2.54 (m, 2H), 2.53 – 2.44 (m, 1H), 2.12 – 1.95 (m, 1H), 1.95 – 1.89 (m, 1H), 1.84 – 1.75 (m, 1H), 1.01 – 0.96 (m, 6H) ppm. ^{13}C{^{1}H} NMR (126 MHz, MeOD) δ 176.0, 46.3, 41.2, 36.6, 31.7, 29.7, 22.4, 22.2 ppm. HRMS(ESI) m/z calculated for $C_8H_{17}O_2S_2^+$ $[M + H]^+$ 209.0592, found: 209.0460.

2-(Cyclohexylthio)-4-mercaptobutanoic acid 6m

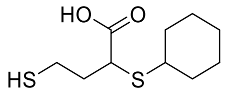

32 mg of **5m** (0.15 mmol) was hydrolysed, affording the corresponding acid as pale yellow oil (25 mg, 70% yield). ^{1}H NMR (500 MHz, MeOD) δ 3.52 (t, J = 7.5 Hz, 1H), 2.91 – 2.71 (m, 1H), 2.60 (t, J = 7.1 Hz, 2H), 2.10 – 2.00 (m, 2H), 2.02 – 1.81 (m, 2H), 1.81 – 1.71 (m, 2H), 1.64 – 1.56 (m, 1H), 1.41 – 1.19 (m, 6H) ppm. ^{13}C{^{1}H} NMR (126 MHz, MeOD) δ 176.6, 45.2, 44.9, 37.2, 35.1, 34.8, 26.8, 22.6 ppm. HRMS(ESI) m/z calculated for $C_{10}H_{19}O_2S_2^+$ $[M + H]^+$ 235.0748, found: 235.0619.

2-(Benzylthio)-4-mercaptobutanoic acid 6n

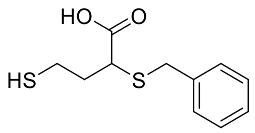

34 mg of **5n** (0.15 mmol) was hydrolysed, affording the corresponding acid as yellow oil (26 mg, 71% yield). ^{1}H NMR (400 MHz, $CDCl_3$) δ 7.41 – 7.21 (m, 5H), 3.99 – 3.78 (m, 2H), 3.36 (dd, J = 8.6, 6.4 Hz, 1H), 2.62 – 2.47 (m, 1H), 2.13 – 1.97 (m, 1H), 1.98 – 1.83 (m, 1H), 1.09 (t, J = 8.5 Hz, 1H) ppm. ^{13}C{^{1}H} NMR (101 MHz, $CDCl_3$) δ 178.8, 137.4, 129.4, 128.8, 127.6, 43.3, 36.3, 34.2, 21.8 ppm. HRMS(ESI) m/z calculated for $C_{11}H_{15}O_2S_2^+$ $[M + H]^+$ 243.0435, found: 243.0306.

4-Mercapto-2-(phenethylthio)butanoic acid 6o

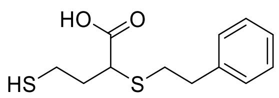

36 mg of **5o** (0.15 mmol) was hydrolysed, affording the corresponding acid as colourless oil (31 mg, 79% yield). ^{1}H NMR (400 MHz, MeOD) δ 7.32 – 7.13 (m, 5H), 3.49 (t, J = 7.5 Hz, 1H), 3.00 – 2.77 (m, 4H), 2.66 – 2.51 (m, 2H), 2.14 – 2.00 (m, 1H), 1.95 – 1.82 (m, 1H) ppm. ^{13}C{^{1}H} NMR (101 MHz, MeOD) δ 175.9, 141.7, 129.6, 129.5, 127.4, 46.0, 37.0, 36.5, 33.9, 22.5 ppm. HRMS(ESI) m/z calculated for $C_{12}H_{17}O_2S_2^+$ $[M + H]^+$ 257.0592, found: 257.0461.

2-((Furan-2-ylmethyl)thio)-4-mercaptobutanoic acid 6p

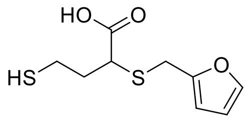

32 mg of **5p** (0.15 mmol) was hydrolysed, affording the corresponding acid as yellow oil (24 mg, 68% yield). ^{1}H NMR (400 MHz, $CDCl_3$) δ 7.41 – 7.35 (m, 1H), 6.32 (td, J = 3.6, 1.9 Hz, 1H), 6.26 (dd, J = 7.6, 3.2 Hz, 1H), 3.99 (dd, J = 14.8, 7.6 Hz, 1H), 3.83 (d, J = 14.8 Hz, 1H), 3.55 – 3.39 (m, 1H), 2.79 – 2.56 (m, 2H), 2.33 – 1.89 (m, 2H), 1.27 (t, J = 8.4 Hz, 1H) ppm. $^{13}C\{^1H\}$ NMR (101 MHz, $CDCl_3$) δ 178.3, 150.3, 142.8, 110.7, 110.7, 108.8, 108.8, 44.2, 43.9, 35.5, 34.2, 29.9, 28.6, 21.9 ppm. HRMS(ESI) m/z calculated for $C_9H_{11}O_3S_2^-$ [M - H]$^-$ 231.0228, found: 231.0140.

3.10. General procedure for the preparation of racemic thiocarboxylic acids 9

Since some acids **9** can spontaneously lactonized during the work up procedure (extraction and drying processes), in such cases the acids **9** were prepared using a NaOH D_2O solution and analysed directly by ^{1}H-NMR as crude compounds.

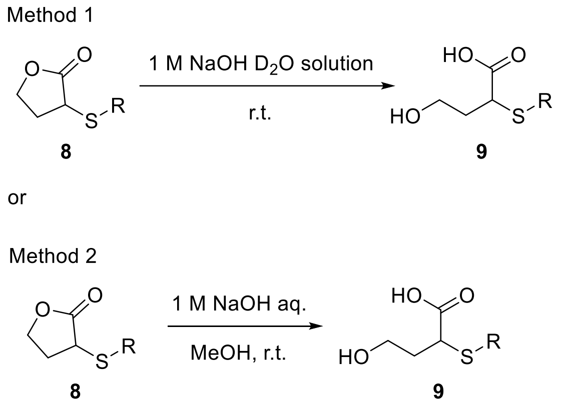

Method 1: The lactone **8** (0.15 mmol) was added to a 1 M NaOH D_2O solution. The reaction mixture was stirred vigorously at room temperature for 1h. Then, the reaction mixture was filtered into an NMR tube for ^{1}H-NMR analysis.

Method 2: To a solution of appropriate lactone **8** (0.15 mmol) in MeOH (1 mL), 1 M NaOH aq. (1 mL) was added. The reaction mixture was stirred vigorously at room temperature for 1h. Then, MeOH was removed under reduced pressure. The reaction mixture was extracted with AcOEt. The aqueous phase was acidified by 2 M HCl aq. to pH 2-3 and extracted with AcOEt. The organic phase was dried over $MgSO_4$, filtered, and concentrated under reduced pressure to afford the corresponding thiocarboxylic acid **9**.

4-Hydroxy-2-(phenylthio)butanoic acid 9a

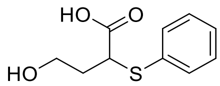

Method 2 was applied for the synthesis of **9a**. 29 mg of **8a** (0.15 mmol) were hydrolysed. The product was obtained as colourless oil (26 mg, 82% yield). ^{1}H NMR (500 MHz, MeOD) δ 7.50 – 7.42 (m, 2H), 7.38 – 7.28 (m, 2H), 3.89 (t, J = 7.4 Hz, 1H), 3.77 – 3.68 (m, 1H), 3.66 – 3.58 (m, 1H), 2.15 – 2.02 (m, 1H), 1.96 – 1.86 (m, 1H) ppm. ^{13}C{^{1}H} NMR (126 MHz, MeOD) δ 174.4, 134.4, 134.3, 130.1, 129.2, 59.9, 48.5, 35.4 ppm. HRMS(ESI) m/z calculated for $C_{10}H_{11}O_3S^-$ [M - H]$^-$ 211.0507, found: 211.0425.

2-((4-Chlorophenyl)thio)-4-hydroxybutanoic acid 9b

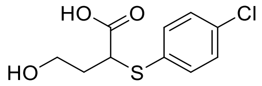

Method 1 was applied for the synthesis of **9b**. Product obtained in NaOH D_2O solution. ^{1}H NMR (400 MHz, D_2O) δ 7.06 – 6.94 (m, 4H), 3.32 – 3.23 (m, 2H), 1.69 – 1.47 (m, 2H) ppm. ^{13}C{^{1}H} NMR (101 MHz, D_2O) δ 179.1, 132.6, 132.5, 132.5, 128.9, 59.0, 34.7 ppm. HRMS(ESI) m/z calculated for $C_{10}H_{10}ClO_3S^-$ [M - H]$^-$ 245.0117, found: 245.0040.

4-Hydroxy-2-(propylthio)butanoic acid 9k

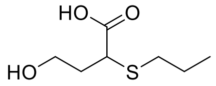

Method 1 was applied for the synthesis of **9c**. Product obtained in NaOH D_2O solution. ^{1}H NMR (400 MHz, D_2O) δ 3.16 – 3.02 (m, 2H), 2.88 – 2.79 (m, 1H) (proton signal integrated as 0.21 due to the proton exchange), 2.16 – 2.00 (m, 2H), 1.46 (dt, J = 13.8, 6.9 Hz, 1H), 1.32 (dt, J = 13.8, 7.1 Hz, 1H), 1.20 – 1.02 (m, 2H), 0.47 (t, J = 7.4 Hz, 3H) ppm. ^{13}C{^{1}H} NMR (101 MHz, D_2O) δ 180.4, 59.1, 47.2, 35.3, 32.9, 22.0, 12.8 ppm. HRMS(ESI) m/z calculated for $C_7H_{15}O_3S^+$ [M + H]$^+$ 179.0664, found: 179.0732.

2-(Benzylthio)-4-hydroxybutanoic acid 9n

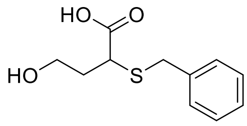

Method 2 was applied for the synthesis of **9d**. 31 mg of **8d** (0.15 mmol) were hydrolysed. The product was obtained as colourless oil; yield 90%. ^{1}H NMR (500 MHz, MeOD) δ 7.41 – 7.18 (m, 6H), 3.92 – 3.79 (m, 2H), 3.65 – 3.51 (m, 2H), 3.36 (t, J = 7.5 Hz, 1H), 2.09 – 1.96 (m, 1H), 1.83 – 1.73 (m, 1H) ppm. ^{13}C{^{1}H} NMR (126 MHz, MeOD) δ 176.1, 139.2, 130.2, 129.5, 128.1, 60.1, 43.9, 36.7, 35.1 ppm. HRMS(ESI) m/z calculated for $C_{11}H_{15}O_3S^+$ [M + H]$^+$ 227.0664, found: 227.0729.

3.11. Procedure for the preparation of racemic thiolactone 10

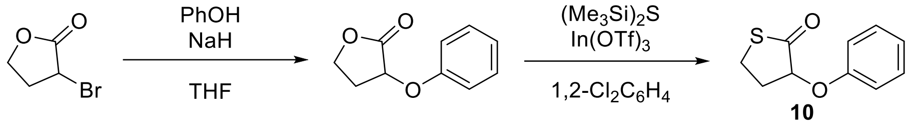

Synthesis of 3-phenoxydihydrofuran-2(3*H*)-one

Phenol (3.3 mmol, 311 mg) was dissolved in THF and NaH (60% dispersion in mineral oil) (3.3 mmol, 132 mg) was added potion-wise. The reaction mixture was stirred for 10 min. Then α-bromo-γ-butyrolactone (3.0 mmol, 495 mg) was added to the mixture and the reaction was stirred until completion was observed by TLC. The reaction was quenched with NH_4Cl sat. solution and extracted by DCM. The collected organic layer was dried over $MgSO_4$ and evaporated under vacuum. Crude products were purified by flash column chromatography (Hexane/AcOEt 9:1 to Hexane/AcOEt 6:4), affording the 3-phenoxydihydrofuran-2(3*H*)-one as white amorphous solid (198 mg, 37% yield). ^1H NMR (500 MHz, $CDCl_3$) δ 7.36 – 7.24 (m, 2H), 7.07 – 7.00 (m, 3H), 4.95 (t, *J* = 7.8 Hz, 1H), 4.56 – 4.48 (m, 1H), 4.36 (td, *J* = 8.7, 6.8 Hz, 1H), 2.77 – 2.67 (m, 1H), 2.52 – 2.40 (m, 1H) ppm. $^{13}C\{^1H\}$ NMR (126 MHz, $CDCl_3$) δ 173.6, 157.4, 129.8, 122.5, 116.0, 72.5, 65.5, 30.0 ppm.

Synthesis of 3-phenoxydihydrothiophen-2(3*H*)-one 10

To a screw-capped tube, 3-phenoxydihydrofuran-2(3*H*)-one (1.0 mmol, 178 mg), $In(OTf)_3$ (0.1 mmol, 56 mg), $(Me_3Si)_2S$ (1.0 mmol, 178 mg) and 1.2-dichlorobenzene (2 mL) were added. After the tube was sealed, the reaction mixture was heated at 80 °C until the completion of the reaction was observed by TLC. The reaction mixture was then directly loaded onto silica gel and purified by flash chromatography with Hexane:AcOEt (90:10) affording the product 3-phenoxydihydrothiophen-2(3*H*)-one **10** as pale yellow amorphous solid (128 mg, 66% yield). ^1H NMR (500 MHz, $CDCl_3$) δ 7.32 – 7.24 (m, 2H), 7.05 – 6.98 (m, 3H), 4.84 (dd, *J* = 8.3, 6.0 Hz, 1H), 3.47 (ddd, *J* = 11.3, 6.5, 4.8 Hz, 1H), 3.35 (ddd, *J* = 11.2, 8.5, 5.9 Hz, 1H), 2.65 – 2.56 (m, 1H), 2.48 – 2.37 (m, 1H) ppm. $^{13}C\{^1H\}$ NMR (126 MHz, $CDCl_3$) δ 204.1, 157.7, 129.7, 122.4, 116.2, 81.5, 31.4, 27.4 ppm. HRMS(ESI) m/z calculated for $C_{10}H_{11}O_2S^+$ $[M + H]^+$ 195.0402, found: 195.0470.

3.12. Procedure for the preparation of the racemic thiocarboxylic acid 11

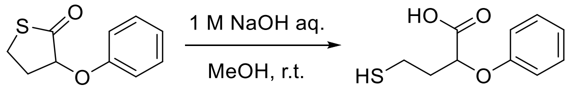

To a solution of thiolactone **10** (0.15 mmol, 29 mg) in MeOH (1 mL), 1M NaOH aq. (1 mL) was added. The reaction mixture was stirred vigorously at room temperature for 1h. Then, MeOH was removed under reduced pressure. The reaction mixture was extracted by AcOEt. The aqueous phase was acidified by 2M HCl aq. to pH 2-3 and extracted again by AcOEt. The organic phase was dried over $MgSO_4$, filtered, and concentrated under reduced pressure. Then the mixture was loaded onto silica gel and purified by flash chromatography with DCM:MeOH (95:5) affording the resulting pure 4-mercapto-2-phenoxybutanoic acid **11** as white amorphous solid (28 mg, 88% yield). ^1H NMR (400 MHz, MeOD) δ 7.29 – 7.19 (m, 2H), 6.97 – 6.84 (m, 3H), 2.79 – 2.59 (m, 2H), 2.32 – 2.07 (m, 2H) ppm. $^{13}C\{^1H\}$ NMR (101 MHz, MeOD) δ 159.3, 130.5, 122.6, 116.1, 38.2, 21.0 ppm. HRMS(ESI) m/z calculated for $C_{10}H_{11}O_3S^-$ [M - H]$^-$ 211.0507, found: 211.0425.

3.13. General procedure of the N9 enzymes catalysed DKR reaction for the synthesis of the acids (*R*)-6 or (*R*)-9 (analytical scale)

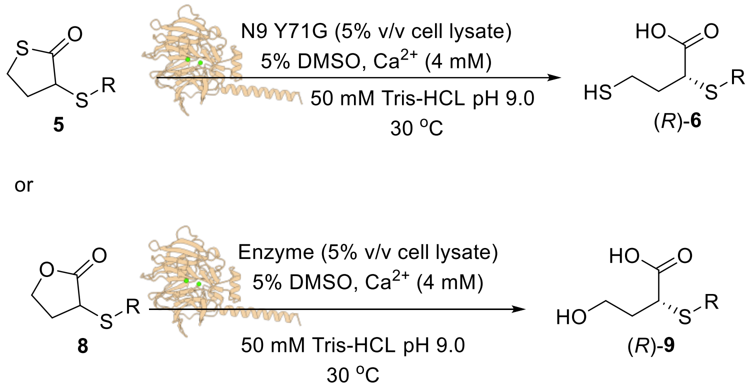

To 950 μL 50 mM Tris-HCl buffer pH 9.0 containing $CaCl_2$ (4 mM) and appropriate enzyme (10% v/v cell lysate), 50 μL of a 200 mM stock solution in DMSO of the relevant thiolactone **5** or lactone **8** (10 mM final concentration) was added. The resulting reaction was shaken at 30 °C and was monitored until completion.

For thiolactones **5**, upon completion, a 100 μL aliquot was spun down and treated with 60 μL 100 mM stock solution of DTBA (4 eq.) at 30 °C for 30 min. Then the mixture was acidified by HCl to pH 3 and extracted by AcOEt (100 μL), centrifuged and the collected organic layer was analysed by normal phase HPLC using the appropriate chiral column to determine the conversion and enantiomeric excess of (*R*)-**6** (see HPLC analysis and traces for conditions).

For lactones **8**, upon completion, a 100 μL reaction mixture was spun down and acidified by HCl to pH 3 and extracted by AcOEt (100 μL), centrifuged and the collected organic layer was analysed by normal phase HPLC using the appropriate chiral column to determine the conversion and enantiomeric excess of (*R*)-**9** (see HPLC analysis and traces for conditions).

3.14. General procedure for the synthesis of the acids (*R*)-6 and (*R*)-9 through N9 enzymes DKR reaction

To 4750 μL 50 mM Tris-HCl buffer pH 9.0 containing $CaCl_2$ (4 mM) and appropriate enzyme (10% v/v cell lysate), 250 μL of a 200 mM stock solution in DMSO of the relevant thiolactone **5** or lactone **8** (10 mM final concentration) was added. The reaction was shaken at 30 °C.

For thiolactone **5**, upon completion, the reaction mixture was spun down and treated with 3 mL 100 mM stock solution of DTBA (4 eq.) at 30 °C for 30 min. Then the mixture was acidified by HCl to pH 3 and extracted by AcOEt (5x5 mL), centrifuged and the collected organic layers were collected and dried over $MgSO_4$ and evaporated under vacuum.

For lactone **8**, upon completion, the reaction mixture was spun down and directly acidified by HCl to pH 3 and extracted by AcOEt (5x5 mL), centrifuged and the collected organic layers were collected and dried over $MgSO_4$ and evaporated under vacuum.

Crude products were purified by flash column chromatography using an appropriate eluent mixture of DCM:MeOH (98:2) to afford the resulting pure enantioenriched thiocarboxylic acids (*R*)-**6** or (*R*)-**9**, which were used for optical rotation measurements.

3.15. Preparative scale synthesis of the acids (*R*)-6a through N9 Y71G enzyme DKR reaction

To 95 mL 50 mM Tris-HCl buffer pH 9.0 containing $CaCl_2$ (4 mM) and N9 Y71G (10% v/v cell lysate), 5 mL of a 200 mM stock solution in DMSO of thiolactone **5a** (210.5 mg, 1 mmol) was added. The reaction was shaken at 30 °C for 24 h. Upon completion, the reaction mixture was spun down and treated with DTBA (609 mg, 4 eq.) at 30 °C for 30 min. Then the mixture was acidified by HCl to pH 3 and extracted by AcOEt (5x10 mL), centrifuged and the collected organic layers were collected and dried over $MgSO_4$ and evaporated under vacuum. Crude products were purified by flash column chromatography using an appropriate eluent mixture of DCM:MeOH (98:2) to afford the resulting pure enantioenriched thiocarboxylic acids (*R*)-**6a** (196 mg, 86% yield, 97% ee).

3.16. General procedure of N9 Y71G catalysed hydrolysis of 10

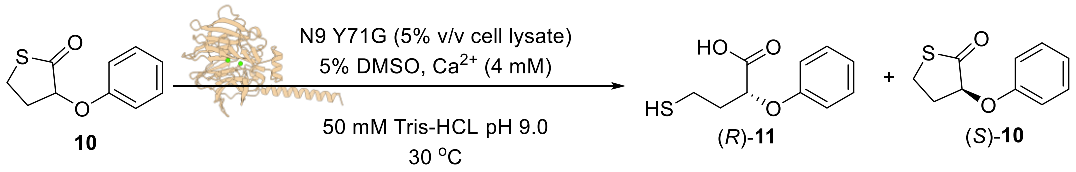

To 950 μL 50 mM Tris-HCl buffer pH 9.0 containing $CaCl_2$ (4 mM) and appropriate enzyme (10% v/v cell lysate), 50 μL of a 200 mM stock solution in DMSO of thiolactone **10** (10 mM final concentration),

was added. The reaction was shaken at 30 ºC. Upon completion, a 100 µL aliquot was spun down and treated with 60 µL 100 mM stock solution of DTBA (4 eq.) at 30 ºC for 30 min. Then the mixture was acidified by HCl to pH 3 and extracted by AcOEt (100 µL), centrifuged and the collected organic layer was analysed by normal phase HPLC using chiral columns to determine the enantiomeric excess of the products from the reactions.

3.17. Racemisation test of (*R*)-5a and (*R*)-6a

Preparation of enantioenriched thiocarboxylic acid (*R*)-6a and thiolactone (*R*)-5a

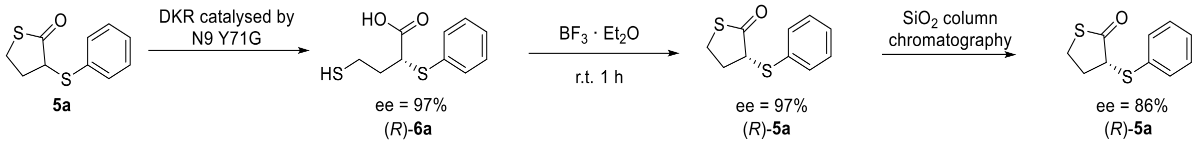

The enantioenriched thiocarboxylic acid (*R*)-**6a** (97% ee) was prepared through the DKR of **5a** catalysed by N9 Y71G as described in section 3.14. The enantioenriched thiolactone (*R*)-**5a** was prepared through lactonization of (*R*)-**6a**. In a round bottom flask containing (*R*)-**6a** (10 mg, 97% ee), 1 mL $BF_3{\cdot}EtO_2$ was added. The mixture was stirred at room temperature for 1 h. Then 10 mL H_2O was added to quench the reaction. AcOEt (5 mL x 3) was added to extract the resulting thiolactone. The organic phase was dried over $MgSO_4$, filtered, and concentrated under reduced pressure. The crude product was purified by flash column chromatography using Hexane/AcOEt 9:1 to afford (*R*)-**5a** (6.7 mg, 86% ee). Little epimerization was observed only after the purification on silica gel.

Racemisation test

To 950 µL 50 mM Tris-HCl buffer pH 9.0 containing $CaCl_2$ (4 mM), 50 µL of a 200 mM stock solution of (*R*)-**5a** or (*R*)-**6a** in DMSO (10 mM final concentration) was added and the resulting mixture was shaken at 30 ºC.

Full racemization of thiolactone (*R*)-**5a** occurred in 15 min. No racemization was observed for the enantioenriched thiocarboxylic acid (*R*)-**6a** over 72 h.

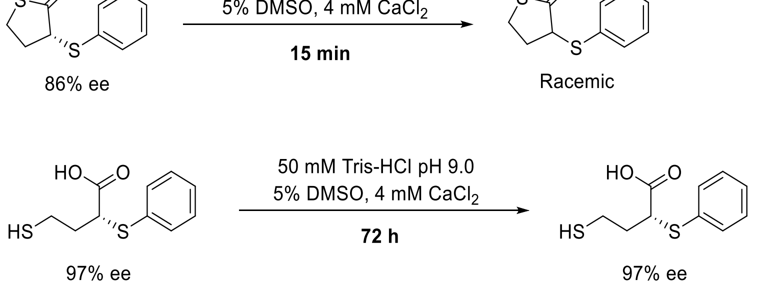

3.18. Deuterium labelling experiment on thiolactone (*R*)-5a

The thiolactone **5a** was suspended in deuterated Tris-HCl buffer (50 mM, pH 9.0, prepared using D_2O). At different times, AcOEt was then added to the mixture and used to extract **5a** from the deuterated buffer. The AcOEt layer was dried over $MgSO_4$, filtered, and concentrated under reduced pressure. The obtained product was analysed by ^{1}H-NMR.

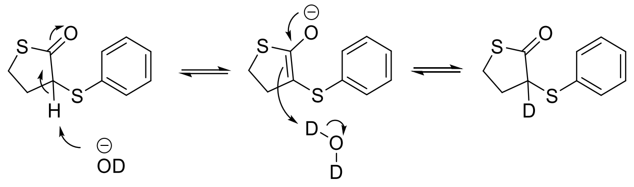

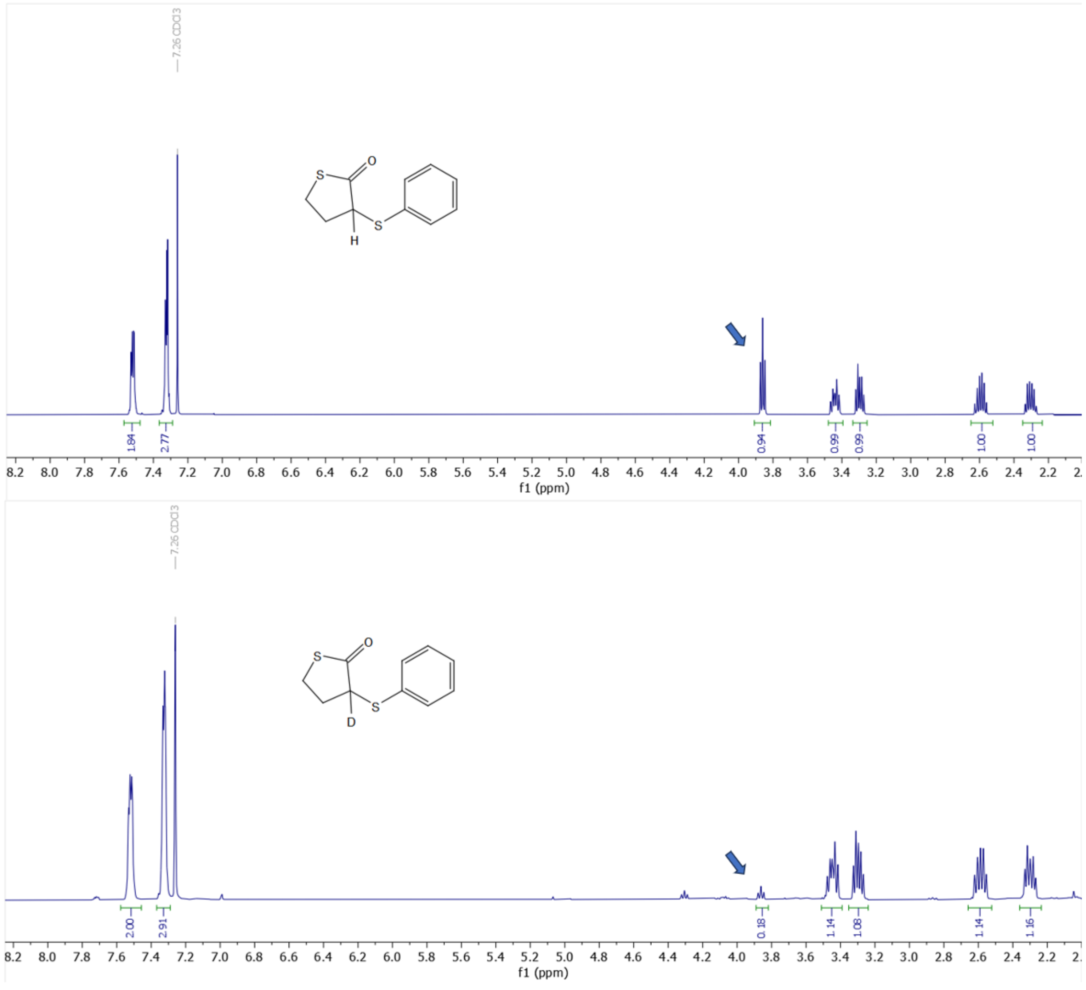

Figure S2. Deuterium labelling experiment on thiolactone (*R*)-**5a**.

3.19. Racemisation test of (*R*)-8b and (*R*)-9b

Preparation of enantioenriched lactone (*R*)-8b and acid (*R*)-9b and

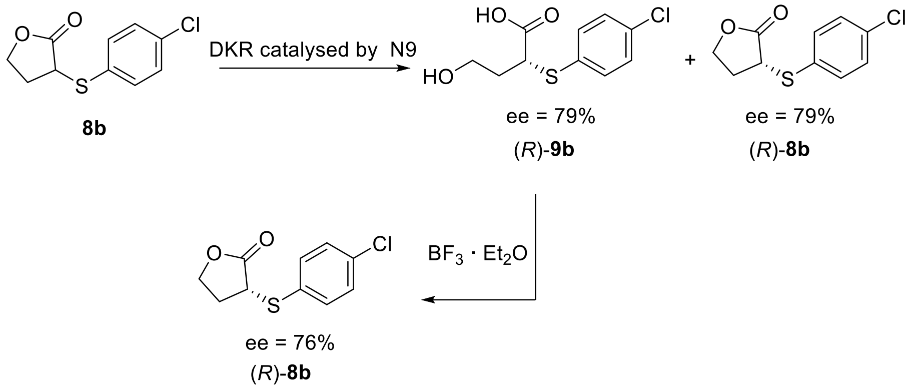

The enantioenriched acid (*R*)-**9b** (79% ee) was prepared through DKR of **8b** catalysed by N9 biocatalyst as described in 3.14. The enantioenriched lactone (*R*)-**8b** was prepared through lactonization of (*R*)-**9b**. In a round bottom flask containing the crude product obtained from the N9 catalysed DKR of **8b** (10 mg, 79% ee), 1 mL $BF_3{\cdot}EtO_2$ was added. The mixture was stirred at room temperature for 1 h. Then 10 mL H_2O was added to quench the reaction. AcOEt (5 mL x 3) was added to extract the resulting lactone. The organic phase was dried over $MgSO_4$, filtered, and concentrated under reduced pressure. The crude product was purified by flash column chromatography using Hexane/AcOEt 9:1 to afford (*R*)-**8b** (6.7 mg, 76% ee).

Racemisation test

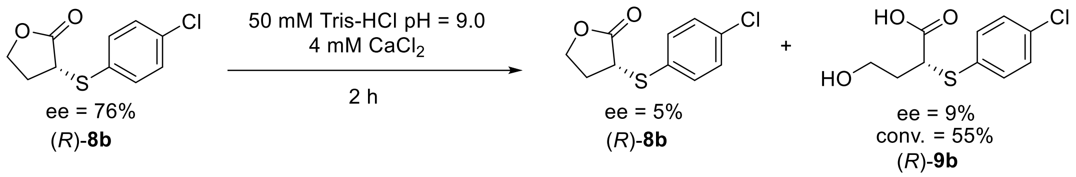

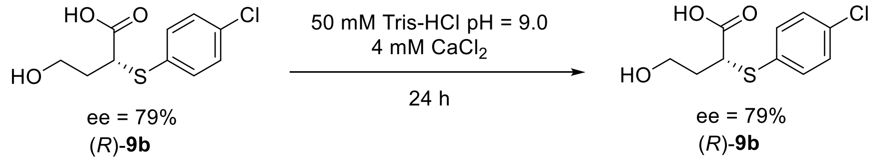

To 950 µL 50 mM Tris-HCl buffer pH 9.0 containing $CaCl_2$ (4 mM), 50 µL of a 200 mM stock solution of (*R*)-**8b** or (*R*)-**9b** in DMSO (10 mM final concentration) was added and shaken at 30 ºC. The ee of (*R*)-**8b** dropped from 76% to 5% in 2 hours. The formation of (*R*)-**9d**, arising from spontaneous hydrolysis of (*R*)-**8b** was observed at the same time. No racemization was observed from enantioenriched thiocarboxylic acid (*R*)-**9d** over 24 h.

3.20. Equilibrium test on lactone 8b and acid 9b

A test reaction to evaluate the spontaneous hydrolysis of the lactone **8b** was carried out. To 950 µL 50 mM Tris-HCl buffer pH 9.0 containing $CaCl_2$ (4 mM), 50 µL of a 200 mM stock solution of **9b** or **8b** in DMSO (10 mM final concentration) was added and shaken at 30 °C. The reaction mixture was monitored using Agilent Eclipse Plus C18 column (H_2O and CH_3CN with TFA additive were used as mobile phase components).

Lactone **8b** was fully converted into the corresponding acid **9b** after 48 h (>99% conversion). The mixture was then acidified by 5 M HCl to pH 3 and extracted by AcOEt (100 µL). The AcOEt phase was analysed using Chiralpak IG column (hexane and EtOH were used as mobile phase components). Lactone **8b** was observed with 10% conversion from the extraction step.

In absence of N9 biocatalyst, the lactone **8b** gives slow spontaneous hydrolysis, which, together with spontaneous epimerization at C3, explain the 79% ee observed in the N9 biocatalysed reaction (Scheme 4 in main text).

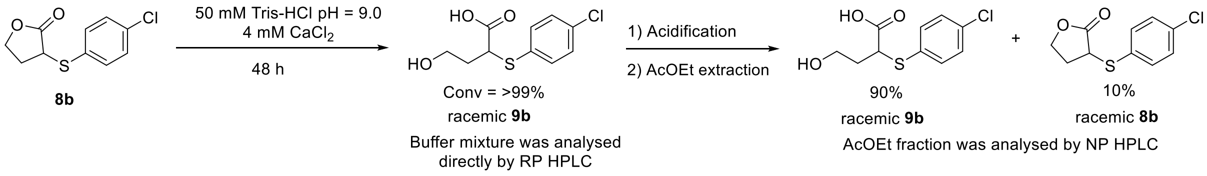

Scheme S2. Equilibrium test on lactone **8b**

Acid **9b** under buffer condition (50 mM Tris-HCl pH 9.0) was observed to have no spontaneous lactonization over 72 h. The mixture was then acidified with 5 M HCl to pH 3 and extracted by AcOEt (100 µL). The AcOEt extraction was monitored using Chiralpak IG column (hexane and EtOH were used as mobile phase components). Lactone **8b** was observed with 10% conversion from the extraction step.

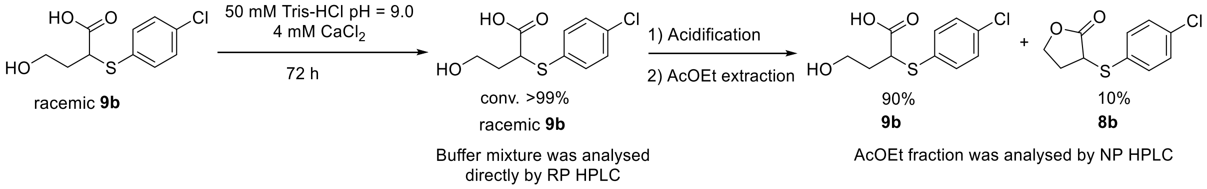

Scheme S3. Equilibrium test on acid **9b**

3.21. Screening of N9 mutants on lactone 8a

Table S4. Screening of N9 mutants on lactone **8a**

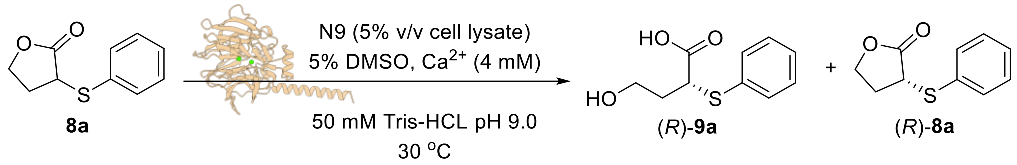

Entry	Variant	Reaction time (h)	Lactone ee (%)[a]	Acid ee (%)[a]	Conversion (%)[a]
1	WT	24	53	54	77
2	Y71A	24	<1	<1	78
3	Y71G	24	<1	<1	78
4	Y71I	24	38	38	79
5	Y71W	24	40	40	76
6	Y71M	24	9	9	76
7	Y71L	24	44	44	71
8	D182A	24	36	36	76
9	N167A	24	37	36	77
10	Empty vector	24	<1	<1	74

All the reactions were conducted at 1 mL scale with 10 mM substrate, 5% v/v cell lysate of N9 mutant, 5% DMSO and 4 mM $CaCl_2$ in 50 Mm pH 9.0 Tris-HCl buffer at 30 ºC. [a] Determined using chiral HPLC using Chiralpak IG column, monitored at 240 nm.

3.22. Screening of N9 mutants on lactone 10

Table S5. Screening of N9 mutants on lactone **10**

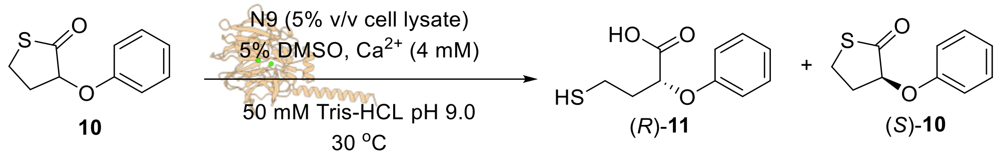

Entry	Variant	Reaction time (h)	Lactone ee (%)[a]	Acid ee (%)[a]	Conversion (%)[a]
1	WT	24	6	30	61
2	Y71A	24	8	10	79
3	Y71G	24	3	Racemic	>99
4	Y71I	24	-20	Racemic	42
5	Y71W	24	14	30	57
6	Y71M	24	6	Racemic	72
7	Y71L	24	3	4	64
8	D182A	24	12	6	71
9	N167A	24	Racemic	Racemic	<1
10	Empty vector	24	Racemic	Racemic	<1

All the reactions were conducted at 1 mL scale with 10 mM substrate, 5% v/v cell lysate of N9 mutant, 5% DMSO and 4 mM $CaCl_2$ in 50 Mm pH 9.0 Tris-HCl buffer at 30 ºC. [a] Determined using chiral HPLC using Chiralcel OJ-H column, monitored at 240 nm.

3.23. Mutants test on thiolactone 5a

Table S6. Mutant test on thiolactone **5a**

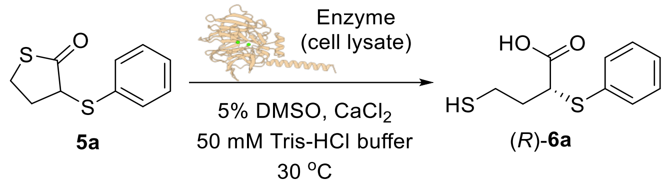

Entry	Variant	Reaction time (h)	Conversion (%)[a]	Acid ee (%)
1	Y71G/H133A	24	<1	n.d.[b]
2	Y71G/N167A	24	<1	n.d.[b]

[a] The conversions were calculated by chiral HPLC using Chiralpak IG column, monitored at 240 nm. [b] Not determined.

3.24. Additional screening of lactonase enzymes

Table S7. Initial screening of lactonases

Entry	Substrate		Enzyme	Time	Lactone ee (%)[a]	Acid ee (%)[a]	Conv. (%)[a]
1	**3b**	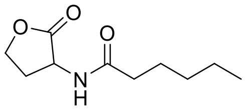	GcL	1 h	72	n.d.[b]	45[c]
2			No enzyme	1 h	< 1	n.d.[b]	11[c]
3	**8k**	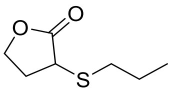	VmutPLL	4h	<1	n.d.[b]	n.d.[b]
4			AaL	15 min	<1	<1	>99
5			GcL	15 min	<1	<1	>99
6			N9	18 h	22	80	23
7			No enzyme	18 h	<1	<1	20
8	**8n**	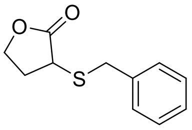	AaL	1 h	<1	<1	>99
9			GcL	1 h	<1	<1	>99
10			N9	1 h	n.d.[b]	52	30
11			No enzyme	1 h	<1	<1	10

All the reactions were conducted at 1 mL scale with 30 mM substrate, 1 mM metal cofactor ($CoCl_2$ for GcL, $MnCl_2$ for VmutPLL, $CaCl_2$ for N9) in 50 mM Tris-HCl buffer, pH 8.0, at 37 °C. After addition of purified lactonase (GcL or VmutPPL, 0.2 mg) or cell extract (N9, 10% v/v), conversions and enantiomeric excesses were monitored at scheduled times. [a] Determined by chiral HPLC using Chiralpak IC or IG column, monitored at 240 nm. [b] Not determined. [c] Determined by ^{1}H-NMR.

3.25. Optical rotations of compound (*R*)-1 and (*S*)-2

Table S8. $[\alpha]_D^{25}$ values of thiolactones **1** and thiocarboxylic acids **2**[a].

Compound	ee (%)	$[\alpha]_D^{25}$	Compound	ee (%)	$[\alpha]_D^{25}$
(*R*)-**1a**[b]	83	-46	(*R*)-**1m**	19	-25
(*R*)-**1b**	94	-55	(*S*)-**2a**	92	+14
(*R*)-**1c**	94	-82	(*S*)-**2b**	79	+6
(*R*)-**1d**	97	-69	(*S*)-**2c**	90	+19
(*R*)-**1g**	78	-52	(*S*)-**2g**	96	+7
(*R*)-**1h**[c]	92	-52	(*S*)-**2h**	90	+14
(*R*)-**1i**	90	-40	(*S*)-**2j**	95	+7
(*R*)-**1j**	71	-65	(*S*)-**2k**	94	+6
(*R*)-**1k**	88	-29	(*S*)-**1j**	95	+42
(*R*)-**1l**	64	-25	(*S*)-**1k**	94	+18

[a] All $[\alpha]_D^{25}$ values were recorded in $CHCl_3$, c = 5 mg/mL, 25 ºC. [b] (*S*)-**1a** $[\alpha]_D$ ($CHCl_3$, c = 7.5 mg/mL): +45.2° was previously reported in literature[76]. [c] (*S*)-**1h** $[\alpha]_D$ ($CHCl_3$, c = 7.7 mg/mL): +20.6° was previously reported in literature[76].

3.26. Optical rotations of DKR products

Determination of the absolute configuration of compounds 8, 9, 6 and 5

The absolute configuration of compounds (*R*)-**8b** was established through comparison with the $[\alpha]_D^{25}$ value reported in literature.[83] The absolute configuration of the acid product **9b** was then determined as (*R*). Since the compounds **9b** and **6b** are structurally similar and the $[\alpha]_D^{25}$ of both optical active samples are measured as negative, the absolute configuration of **6b** arising from N9 catalysed DKR was also assigned as (*R*). The computational studies carried out in this work also showed that the N9 enzymes are (*R*) selective on substrates **5**, which matches and is in perfect agreement with the conclusions arising from the optical rotation data.

Table S9. $[\alpha]_D^{25}$ values of thiocarboxylic acids (*R*)-**6**, (*R*)-**9b** and lactone (*R*)-**8b**

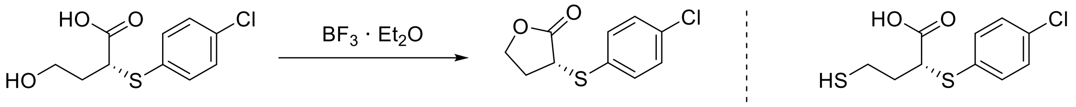

ee = 79%, $[\alpha]_D^{25}$ = -49 (*R*)-**9b**; ee = 79%, $[\alpha]_D^{25}$ = -32 (*R*)-**8b**; ee = 95%, $[\alpha]_D^{25}$ = -49 (*R*)-**6b**

Compound	ee (%)	$[\alpha]_D^{25}$
(*R*)-**6a**	97	-81
(*R*)-**6b**	95	-50
(*R*)-**6c**	97	-62
(*R*)-**6d**	97	-71
(*R*)-**6e**	97	-60
(*R*)-**6f**	>99	-82
(*R*)-**6g**	95	-55
(*R*)-**6h**	92	-56
(*R*)-**6i**	97	-39
(*R*)-**6j**	93	-69
(*R*)-**6k**	87	-90
(*R*)-**6l**	90	-60
(*R*)-**6m**	87	-63
(*R*)-**6n**	90	-82
(*R*)-**6o**	90	-80
(*R*)-**6p**	80	-60
(*R*)-**9b**	79	-49
(*R*)-**8b**	79	-32

All $[\alpha]_D^{25}$ values were recorded in AcOEt, c = 5 mg/mL, 25 °C.

4. Computational study

The crystal structure of GcL was downloaded from PDB database (6N9Q).[16] Structural models of N9 and N9 mutants were built using AlphaFold2[95] and two calcium ions and water molecules in the active site were modelled and incorporated in the structures using AlphaFill[96].

Catalytic Ca^{2+} stability and its coordinating binding site residues in N9

Molecular dynamics (MD) simulation was employed to validate the stability of the modelled catalytic calcium coordination and its binding site coordinating residues in N9. The *AmberTools22*[97] *tleap* program was used to set up the solvated system in a cubic box of *TIP3P*[98] water molecules, ensuring at least a 10 Å buffer between the protein and the nearest box edge. The protein was parameterized with the ff19SB force field[99]. Using Amber 22 *pmemd.CUDA*[100] the MD simulation protocol involved a two-stage energy minimization—first, 1,000 cycles without SHAKE (switching to conjugate gradient after 50 cycles) and then 2,500 cycles with SHAKE applied to bonds with hydrogen—both at constant volume with a 9 Å nonbonded interactions cutoff. This was followed by an equilibration phase at constant volume for 100,000 steps (1 fs timestep) where the temperature was ramped from 0 K to 300 K using Langevin dynamics (1 ps^{-1} collision frequency) with weak atom restraints on the enzyme (10 kcal/(mol·$Å^2$)). The production run was conducted at constant pressure (1.0 bar with isotropic scaling and a 2.0 ps pressure relaxation time) at 300 K, employing a 2 fs timestep over 130 ns simulation time with SHAKE maintained. The Particle Mesh Ewald (PME) method[101] was used to calculate long-range electrostatics, with periodic boundary conditions (PBC) applied. MD analysis was done via scripts using numpy[102] and matplotlib[103] python packages. Ultimately the MD simulation confirmed that the Ca^{2+} ion remained stably coordinated with its binding site residues depicted in the Figure S3.

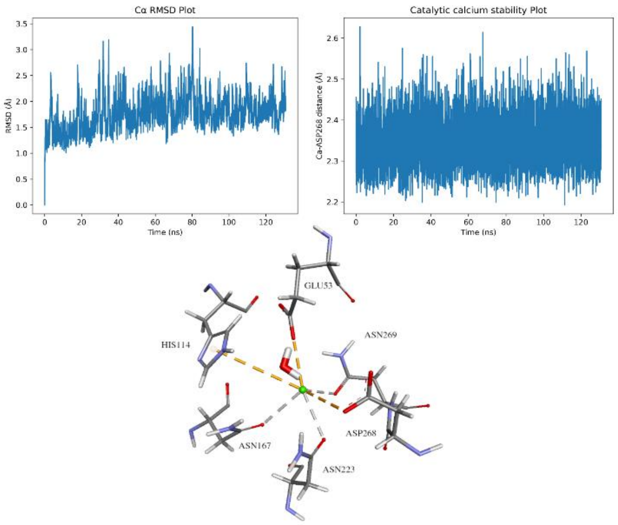

Figure S3. Top: on the left, The Root Mean Square Deviation (RMSD) plot of the N9 carbon alpha (Cα) indicates that the system is equilibrated throughout the 130 ns trajectory. On the right, the plot shows the stable position of Ca^{2+} throughout 130 ns of simulation by monitoring the distance between the catalytic Ca^{2+} and Asp 268. Bottom: Ca^{2+} coordinating residues in the binding site are depicted.

Molecular docking of lactonase GcL

Table S10. Docking analysis for lactonase GcL.

Ligand (Substrate)	ΔG (kcal/mol)	Ranking[a]	In catalytic pot	Orientation state[b]
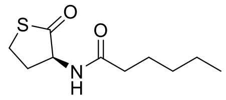 (*S*)-**1a**	-5.2	1	Yes	Yes
	-5.0	2	Yes	No
	-4.8	3	Yes	Yes
	-4.6	4	Yes	No
	-4.5	5	Yes	No
	-4.4	6	No	-
	-4.3	7	No	-
	-4.2	8	Yes	No
	-4.1	9	No	-
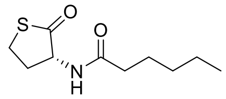 (*R*)-**1a**	-4.9	1	Yes	No
	-4.1	2	Yes	No
	-4.1	3	No	-
	-4.1	4	No	-
	-4.0	5	No	-
	-4.0	6	No	-
	-3.9	7	No	-
	-3.9	8	No	-
	-3.8	9	No	-

[a] The ranking order followed the affinity energy ΔG.
[b] Orientation state: If yes, the hydrolytic site of the ligand is facing towards the bimetallic site of GcL WT. Otherwise, the ligand is not in a suitable orientation state. (see below)

If yes

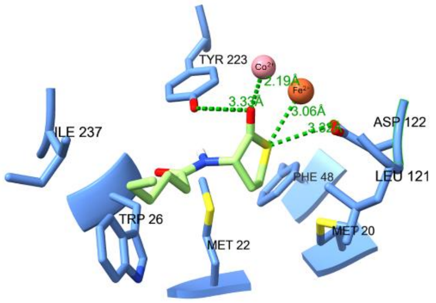

If no

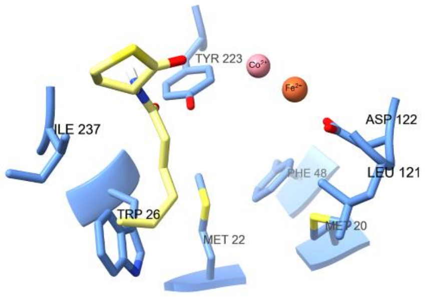

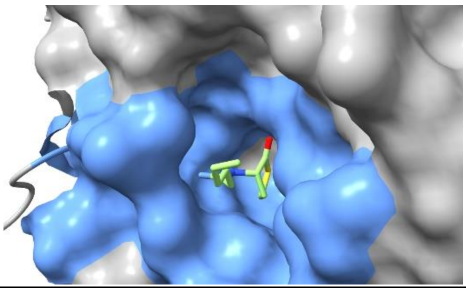

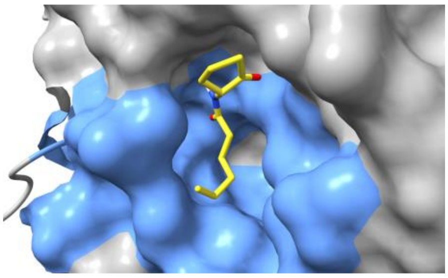

The water molecules and other subunits were deleted and saved as a pdb file using UCSF ChimeraX[104,105]. Hydrogens were then added to the protein using AutoDock Tools (version 1.5.6)[106]. In GcL docking study of substrate **1a**, the docking box was set to the size that covers the whole protein (CENTERX = 54, CENTERY = 3, CENTERZ = 36, docking box dimension of 50*50*50 Å) and then

saved as a pdbqt file. Chemdraw3D was used to generate and minimise the energy of the ligand. Then, the ligand was formatted as pdbqt file via AutoDock Tools. Docking was performed via Autodock Vina (v.1.2.0)[107,108] through a terminal window.

Molecular docking of lactonase N9 and N9 Y71G

For the docking studies on N9 and N9 Y71G to rationalise the increased selectivity of the latter towards the enantiomer (*R*)-**5a**, the ligand structures were prepared using Open Babel software.[109] For each docking system mentioned in the manuscript, 200 docking runs via Autodock Vina[107,108] with exhaustiveness of 32, docking box dimension of 16*16*16 Å centred on the catalytic Ca^{2+} in the active site (CENTERX = 2.057, CENTERY = 2.051, CENTERZ = −0.188), were performed. The results were clustered based on the distance between the oxygen of the carbonyl moiety of the ligands resulted poses and the catalytic Ca^{2+} ion and plotted against the docking affinity score using python packages of pandas[109], numpy[102] and matplotlib[110]. The structures and docking results were analysed and visualized using UCSF ChimeraX[104,105] and Biovia Discovery Studio Visualizer[111,112].

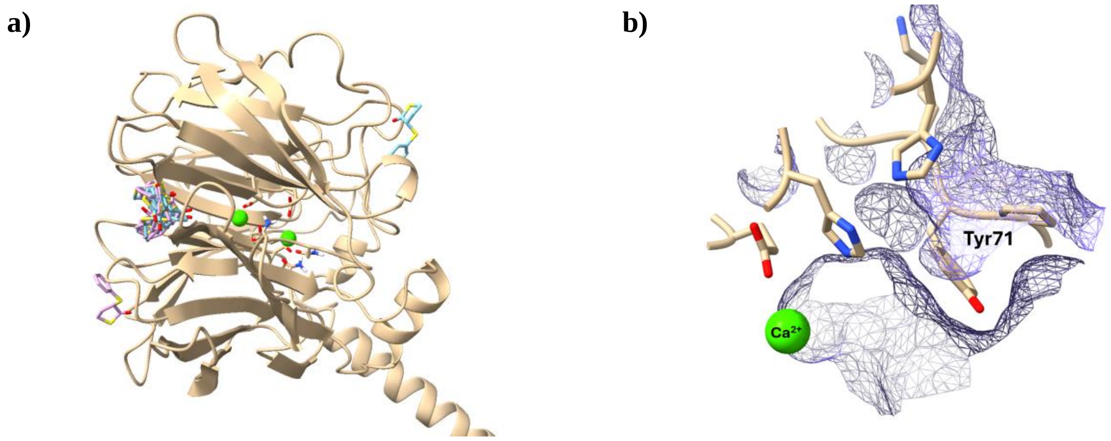

Figure S4. a) Docking of thiolactones (*S*)-**5a** and (*R*)-**5a** into biocatalyst N9. b) Active site of the biocatalyst N9.

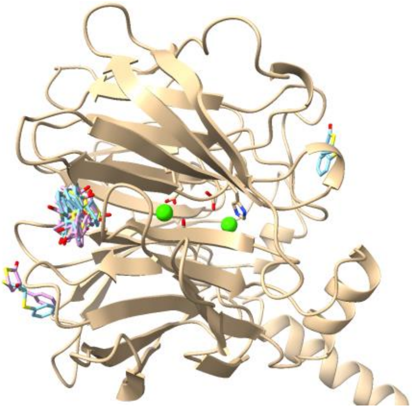

Figure S5. Docking of thiolactones (*S*)-**5a** (blue) and (*R*)-**5a** (purple) into N9 Y71W.

a)

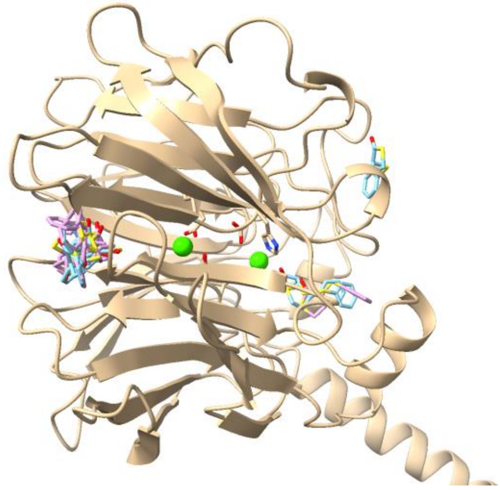

b)

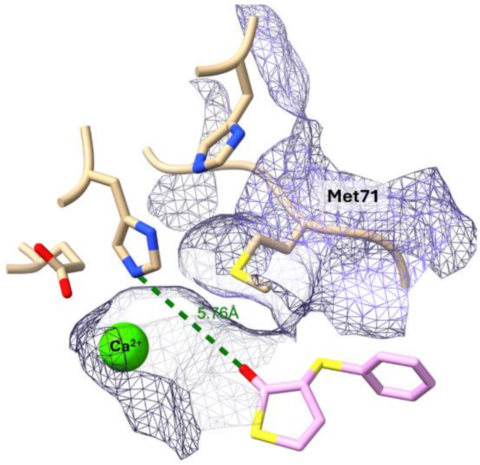

Figure S6. a) Docking of thiolactone (*S*)-**5a** and (*R*)-**5a** into N9 Y71M. b) Best docking pose of (*R*)-**5a** in the active site of N9 Y71M.

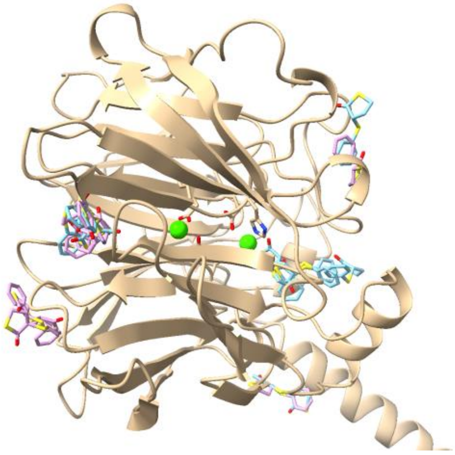

Figure S7. Docking of thiolactone (*S*)-**5a** (blue) and (*R*)-**5a** (purple) into N9 Y71I.

a)

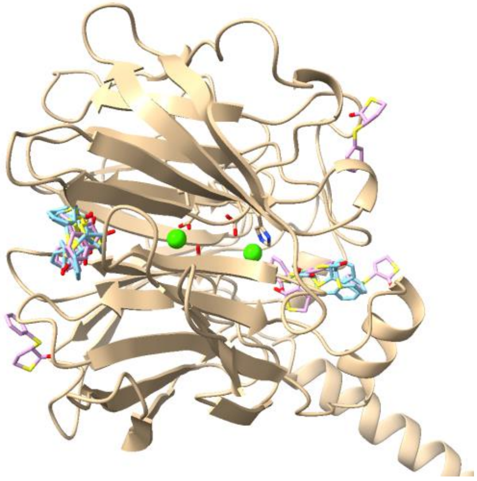

b)

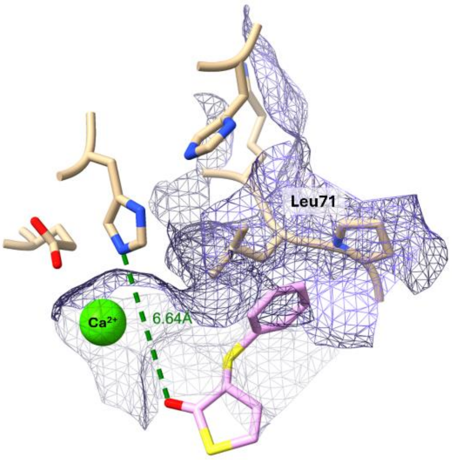

Figure S8. a) Docking of thiolactones (*S*)-**5a** and (*R*)-**5a** into N9 Y71L. b) Best docking pose of (*R*)-**5a** in the active site of N9 Y71L.

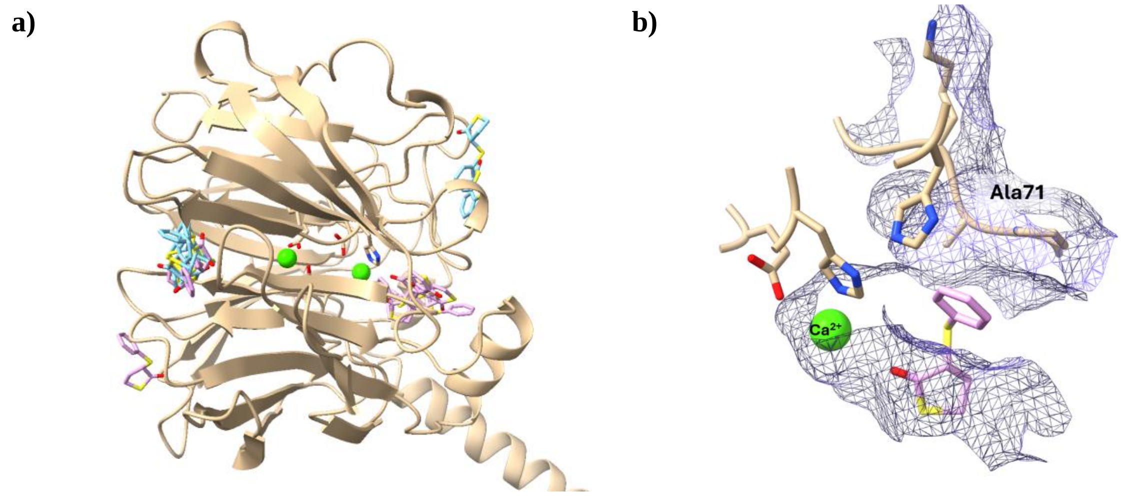

Figure S9. Docking of thiolactone (*S*)-**5a** (blue) and (*R*)-**5a** (purple) into N9 Y71A. b) Best docking pose of (*R*)-**5a** in the active site of N9 Y71A.

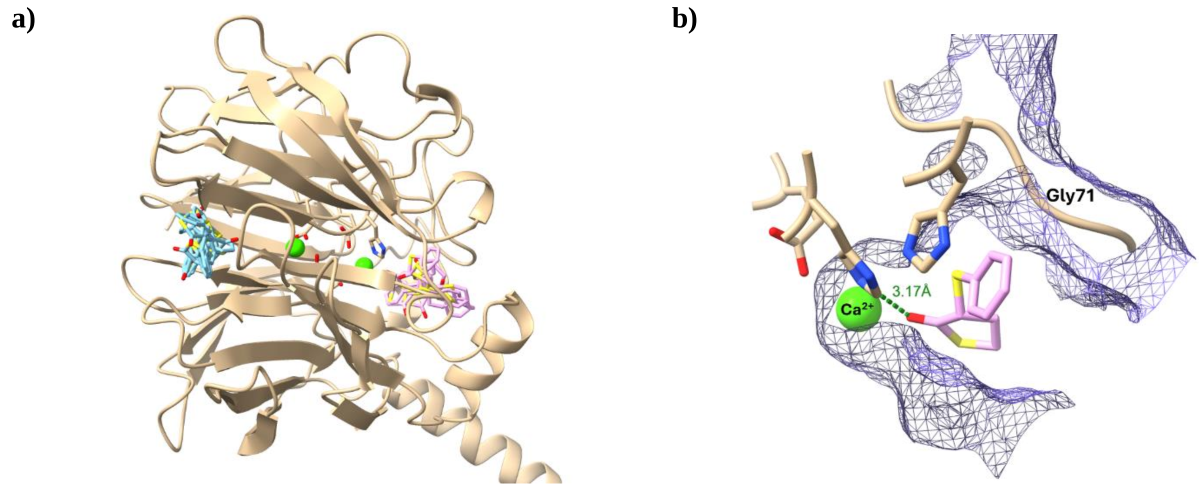

Figure S10. a) Docking of thiolactone (*S*)-**5a** and (*R*)-**5a** into N9 Y71G. b) Best docking pose of (*R*)-**5a** in the active site of N9 Y71G.

5. References

[87] F. W. Studier, *Protein Expression Purif.* **2005**, *41*, 207–234.

[88] R. Ramanujam, S. Ganjihal, N. Kalyanam, M. Majeed, *Tetrahedron Asymmetry* **2013**, *24*, 663–668.

[89] S. Hofer, A. Ronacher, J. Horak, H. Graalfs, W. Lindner, *J. Chromatogr. A* **2011**, *1218*, 8925–8936.

[90] J. Yan, X. Wang, J. Xiong, L. Wang, D. Pan, Y. Xu, M. Yang, *Chem. Eng. J.* **2022**, *428*, 132142.

[91] L. K. Shekhawat, T. Markle, K. Esfandiarfard, E. K. Theel, J.-L. Maloisel, G. Malmquist, *J. Chromatogr. A* **2023**, *1699*, 464018.

[92] J. T. Hodgkinson, W. R. J. D. Galloway, M. Casoli, H. Keane, X. Su, G. P. C. Salmond, M. Welch, D. R. Spring, *Tetrahedron Lett.* **2011**, *52*, 3291–3294.

[93] Y. Tamura, H. Annoura, M. Fuji, M. Okura, H. Ishibasi, *Chem. Pharm. Bull. (Tokyo)* **1986**, *34*, 540–549.

[94] Y. Ogiwara, K. Takano, S. Horikawa, N. Sakai, *Molecules* **2018**, *23*, 1339.

[95] J. Jumper, R. Evans, A. Pritzel, T. Green, M. Figurnov, O. Ronneberger, K. Tunyasuvunakool, R. Bates, A. Žídek, A. Potapenko, A. Bridgland, C. Meyer, S. A. A. Kohl, A. J. Ballard, A. Cowie, B. Romera-Paredes, S. Nikolov, R. Jain, J. Adler, T. Back, S. Petersen, D. Reiman, E. Clancy, M. Zielinski, M. Steinegger, M. Pacholska, T. Berghammer, S. Bodenstein, D. Silver, O. Vinyals, A. W. Senior, K. Kavukcuoglu, P. Kohli, D. Hassabis, *Nat.* **2021**, *596*, 583–589.

[96] M. L. Hekkelman, I. de Vries, R. P. Joosten, A. Perrakis, *Nat. Methods* **2023**, *20*, 205–213.

[97] D. A. Case, H. M. Aktulga, K. Belfon, D. S. Cerutti, G. A. Cisneros, V. W. D. Cruzeiro, N. Forouzesh, T. J. Giese, A. W. Götz, H. Gohlke, S. Izadi, K. Kasavajhala, M. C. Kaymak, E. King, T. Kurtzman, T.-S. Lee, P. Li, J. Liu, T. Luchko, R. Luo, M. Manathunga, M. R. Machado, H. M. Nguyen, K. A. O'Hearn, A. V Onufriev, F. Pan, S. Pantano, R. Qi, A. Rahnamoun, A. Risheh, S. Schott-Verdugo, A. Shajan, J. Swails, J. Wang, H. Wei, X. Wu, Y. Wu, S. Zhang, S. Zhao, Q. Zhu, T. E. I. I. I. Cheatham, D. R. Roe, A. Roitberg, C. Simmerling, D. M. York, M. C. Nagan, K. M. Jr. Merz, *J. Chem. Inf. Model.* **2023**, *63*, 6183–6191.

[98] I. S. Joung, T. E. I. I. I. Cheatham, *J. Phys. Chem. B* **2009**, *113*, 13279–13290.

[99] C. Tian, K. Kasavajhala, K. A. A. Belfon, L. Raguette, H. Huang, A. N. Migues, J. Bickel, Y. Wang, J. Pincay, Q. Wu, C. Simmerling, *J. Chem. Theory Comput.* **2020**, *16*, 528–552.

[100] D. A. Case, R. E. Duke, R. C. Walker, N. R. Skrynnikov, T. E. Cheatham III, O. Mikhailovskii, C. Simmerling, Y. Xue, A. Roitberg, S. A. Izmailov, K. M. Merz, *AMBER 22 Reference Manual*, **2022**.

[101] T. Darden, D. York, L. Pedersen, *J. Chem. Phys.* 1993, *98*, 10089–10092.

[102] C. R. Harris, K. J. Millman, S. J. van der Walt, R. Gommers, P. Virtanen, D. Cournapeau, E. Wieser, J. Taylor, S. Berg, N. J. Smith, R. Kern, M. Picus, S. Hoyer, M. H. van Kerkwijk, M. Brett, A. Haldane, J. F. del Río, M. Wiebe, P. Peterson, P. Gérard-Marchant, K. Sheppard, T. Reddy, W. Weckesser, H. Abbasi, C. Gohlke, T. E. Oliphant, *Nat.* **2020**, *585*, 357–362.

[103] P. Barrett, J. Hunter, J. T. Miller, J.-C. Hsu, P. Greenfield, in *Astronomical Data Analysis Software and Systems XIV*, 2005, p. 91.

[104] E. C. Meng, T. D. Goddard, E. F. Pettersen, G. S. Couch, Z. J. Pearson, J. H. Morris, T. E. Ferrin, *Protein Sci.* **2023**, *32*, e4792.

[105] E. F. Pettersen, T. D. Goddard, C. C. Huang, E. C. Meng, G. S. Couch, T. I. Croll, J. H. Morris, T. E. Ferrin, *Protein Sci.* **2021**, *30*, 70–82.

[106] G. M. Morris, R. Huey, W. Lindstrom, M. F. Sanner, R. K. Belew, D. S. Goodsell, A. J. Olson, *J. Comput. Chem.* **2009**, *30*, 2785–2791.

[107] O. Trott, A. Olson, *Effic. Optim. Multithreading* **2009**, *31*, 455–461.

[108] J. Eberhardt, D. Santos-Martins, A. F. Tillack, S. Forli, *J. Chem. Inf. Model.* **2021**, *61*, 3891–3898.

[109] N. M. O’Boyle, M. Banck, C. A. James, C. Morley, T. Vandermeersch, G. R. Hutchison, *J. cheminf.* **2011**, *3*, 1-14.

[110] W. McKinney, *Python for high performance and scientific computing* **2011**, *14*, 1–9.

[111] P. Barrett, J. Hunter, J. T. Miller, J.-C. Hsu, P. Greenfield, *Astronomical Data Analysis Software and Systems XIV*, **2005**, p. 91.

[112] D. S. Biovia, H. M. Berman, J. Westbrook, Z. Feng, G. Gilliland, T. N. Bhat, T. J. Richmond, *J. Chem. Phys.* **2000**, *10*, 21–9991.

6. Conditions for HPLC analysis and copies of HPLC spectra

6.1. HPLC analysis for GcL catalysed EKR

HPLC conditions: Chiralpak® IC column (4.6 mm × 250 mm, 5 μm), 240 nm, 20% EtOH/hexane, 1.0 mL/min.

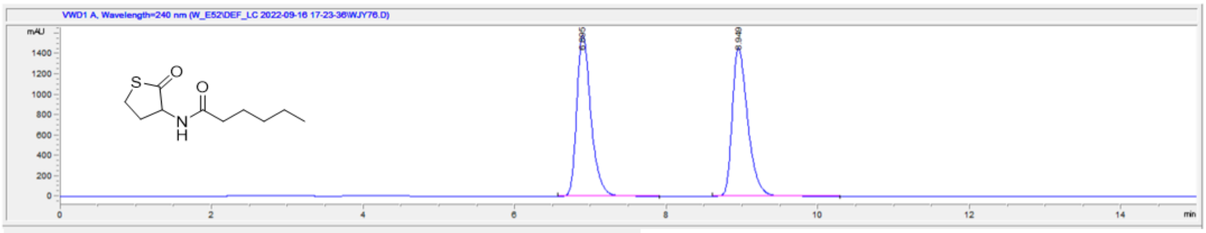

#	Time	Area	Height	Width	Area%	Symmetry
1	6.895	20534.8	1584	0.1993	49.942	0.697
2	8.949	20582.4	1457	0.2187	50.058	0.626

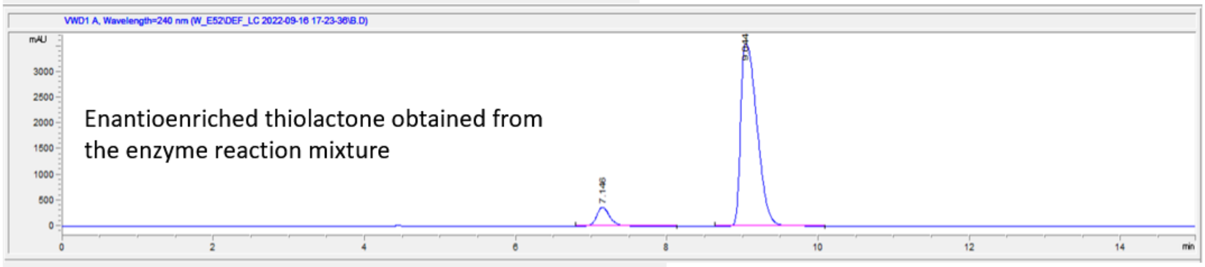

#	Time	Area	Height	Width	Area%	Symmetry
1	7.146	4377	362.5	0.186	7.614	0.752
2	9.044	53110.1	3551.1	0.2313	92.386	0.458

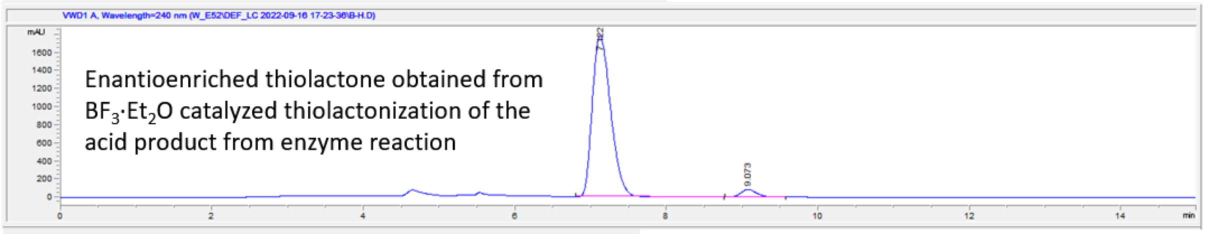

#	Time	Area	Height	Width	Area%	Symmetry
1	7.122	29993	1782.4	0.2647	96.161	0.684
2	9.073	1197.5	83.1	0.2263	3.839	0.756

HPLC conditions: Chiralpak® IG column (4.6 mm × 250 mm, 5 μm), 240 nm, 10% EtOH/hexane, 1.0 mL/min.

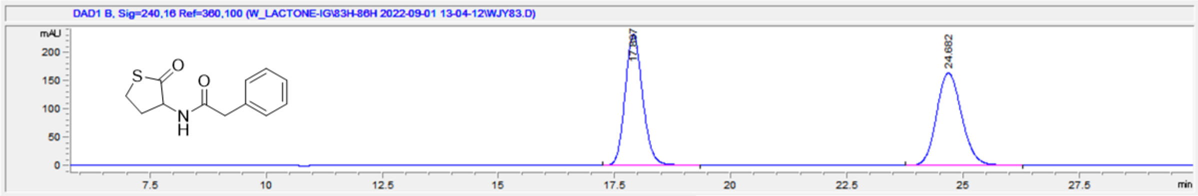

#	Time	Area	Height	Width	Area%	Symmetry
1	17.897	6074.4	230.8	0.4011	49.799	0.839
2	24.682	6123.4	163.2	0.5837	50.201	0.854

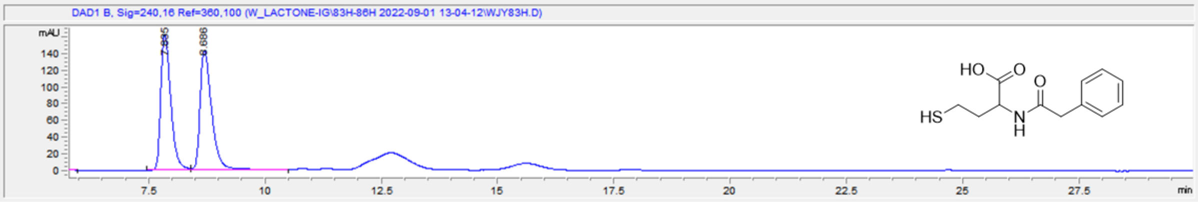

#	Time	Area	Height	Width	Area%	Symmetry
1	7.835	2429.8	163	0.2319	49.270	0.617
2	8.686	2501.8	143.2	0.2638	50.730	0.543

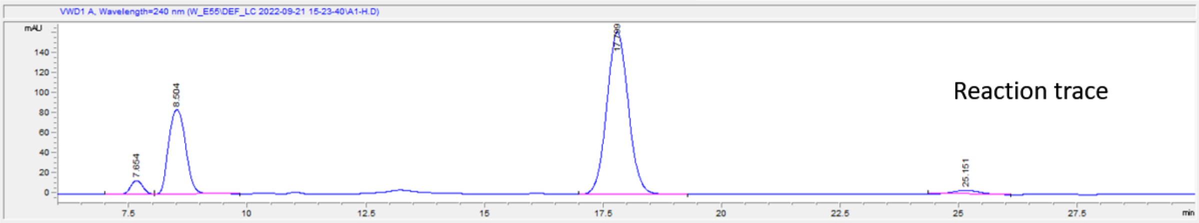

#	Time	Area	Height	Width	Area%	Symmetry
1	7.654	247.4	13.5	0.2887	3.237	0.943
2	8.504	2119.3	84.3	0.4083	27.731	0.854
3	17.799	5116.3	162.3	0.4987	66.947	0.927
4	25.151	159.4	4	0.5636	2.085	0.939

HPLC conditions: Chiralpak® IC column (4.6 mm × 250 mm, 5 µm), 240 nm, 20% EtOH/hexane, 1.0 mL/min.

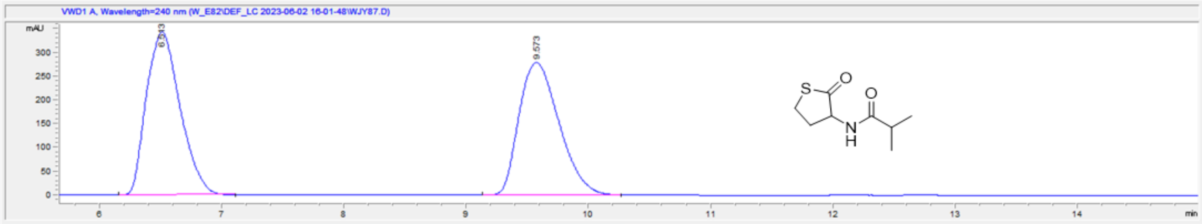

#	Time	Area	Height	Width	Area%	Symmetry
1	6.513	6574.5	343.3	0.3064	50.728	0.813
2	9.573	6385.9	279.5	0.3641	49.272	0.718

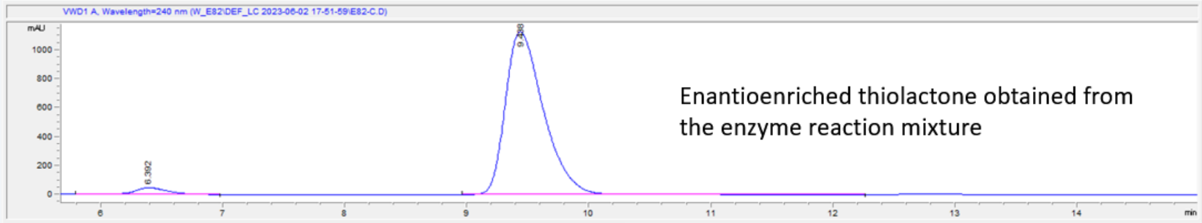

#	Time	Area	Height	Width	Area%	Symmetry
1	6.392	949.4	46.6	0.3074	3.737	0.815
2	9.438	24458.9	1127.7	0.3317	96.263	0.604

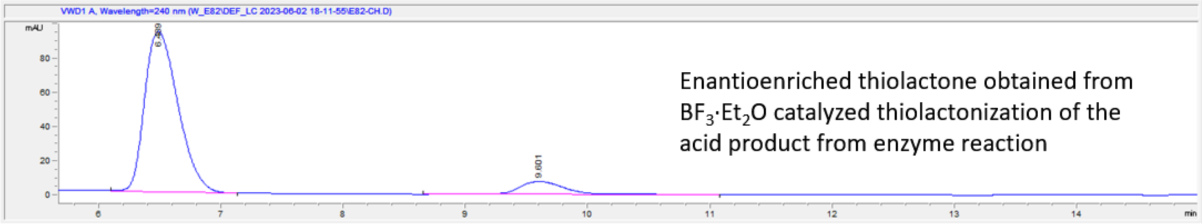

#	Time	Area	Height	Width	Area%	Symmetry
1	6.489	1898.9	95.3	0.3082	89.704	0.663
2	9.601	218	7.6	0.433	10.296	0.719

HPLC conditions: Chiralpak® IC column (4.6 mm × 250 mm, 5 µm), 240 nm, 20% EtOH/hexane, 1.0 mL/min.

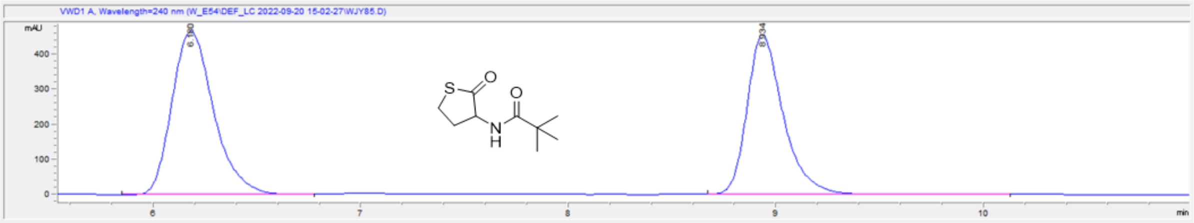

#	Time	Area	Height	Width	Area%	Symmetry
1	6.18	6500.9	464.6	0.2157	54.053	0.777
2	8.934	5526	454.9	0.1839	45.947	0.69

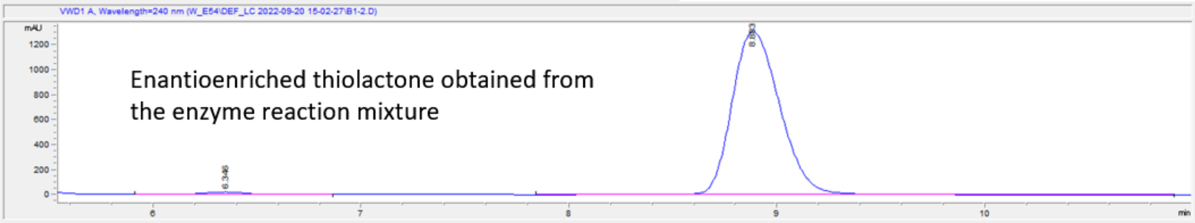

#	Time	Area	Height	Width	Area%	Symmetry
1	6.346	279.1	20.1	0.2158	1.320	1.152
2	8.883	20871.2	1307.8	0.2546	98.680	0.738

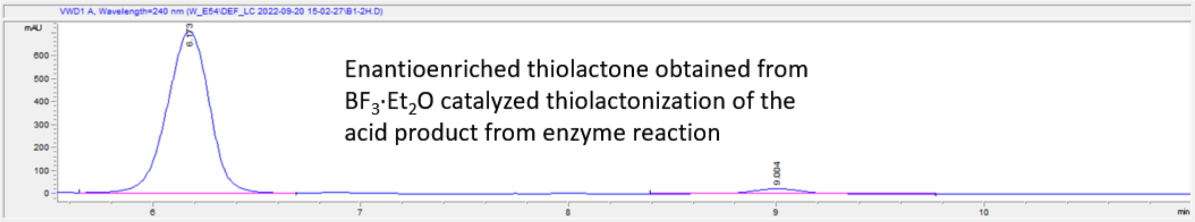

#	Time	Area	Height	Width	Area%	Symmetry
1	6.173	10080.1	705.9	0.2219	96.826	1.111
2	9.004	330.4	20.9	0.2468	3.174	1.032

HPLC conditions: Chiralpak® IG column (4.6 mm × 250 mm, 5 μm), 240 nm, 40% EtOH/hexane, 1.0 mL/min.

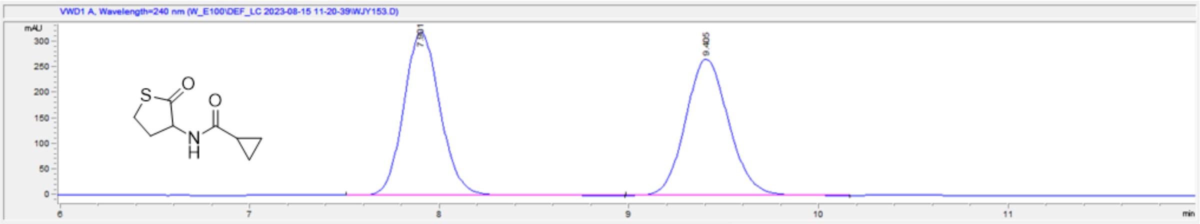

#	Time	Area	Height	Width	Area%	Symmetry
1	7.901	4242	319.8	0.2057	50.191	0.872
2	9.405	4209.8	266.1	0.2443	49.809	0.876

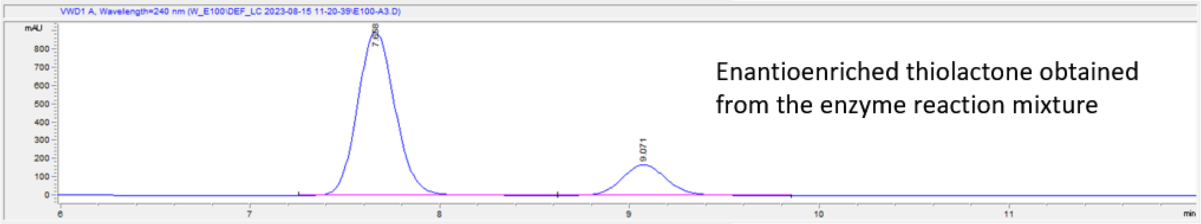

#	Time	Area	Height	Width	Area%	Symmetry
1	7.658	12274.9	897.1	0.2135	81.944	0.877
2	9.071	2704.8	167.4	0.2511	18.056	0.905

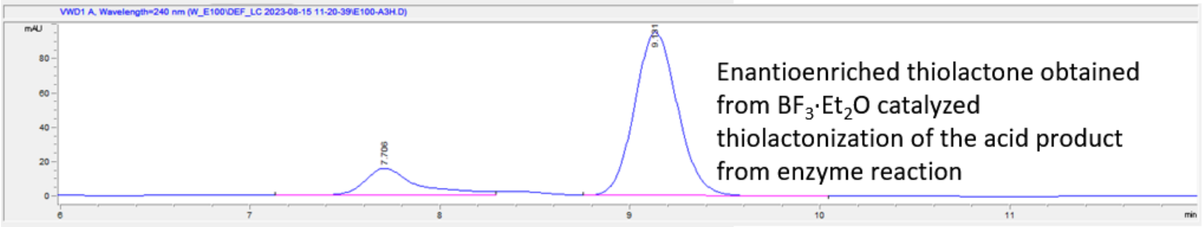

#	Time	Area	Height	Width	Area%	Symmetry
1	7.706	320.7	15.9	0.2874	17.505	0.533
2	9.131	1511.5	95.5	0.2458	82.495	0.895

HPLC conditions: Chiralpak® IG column (4.6 mm × 250 mm, 5 µm), 240 nm, 40% EtOH/hexane, 1.0 mL/min.

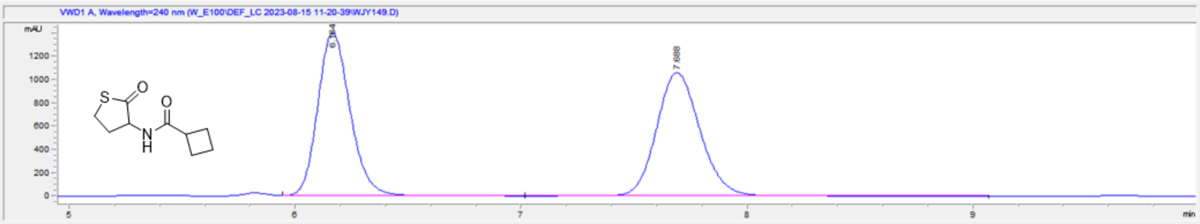

#	Time	Area	Height	Width	Symmetry
1	6.164	13957.4	1404.4	0.1538	0.797
2	7.688	14127.5	1059.5	0.2065	0.847

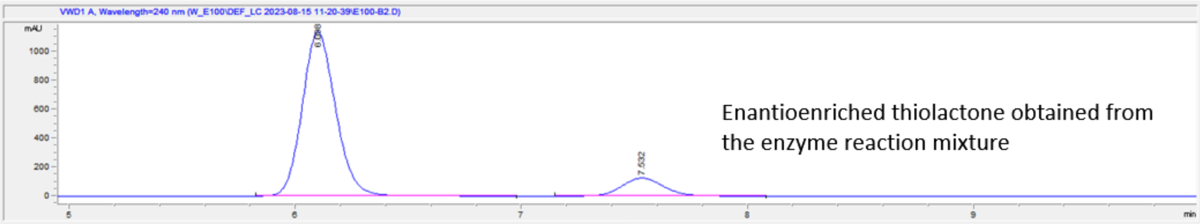

#	Time	Area	Height	Width	Symmetry
1	6.098	11636.3	1133.3	0.1576	0.833
2	7.532	1565.3	122.7	0.1983	0.89

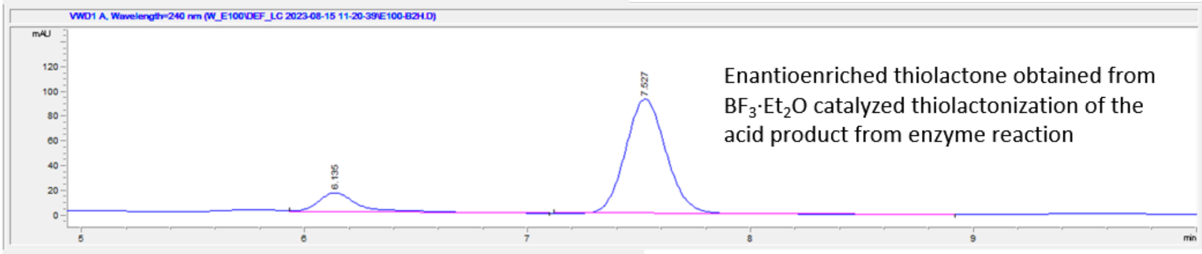

#	Time	Area	Height	Width	Area%	Symmetry
1	6.135	225.3	16	0.2051	16.006	0.549
2	7.527	1182.5	92.5	0.2016	83.994	0.883

HPLC conditions: Chiralpak® IC column (4.6 mm × 250 mm, 5 µm), 240 nm, 20% EtOH/hexane, 1.0 mL/min.

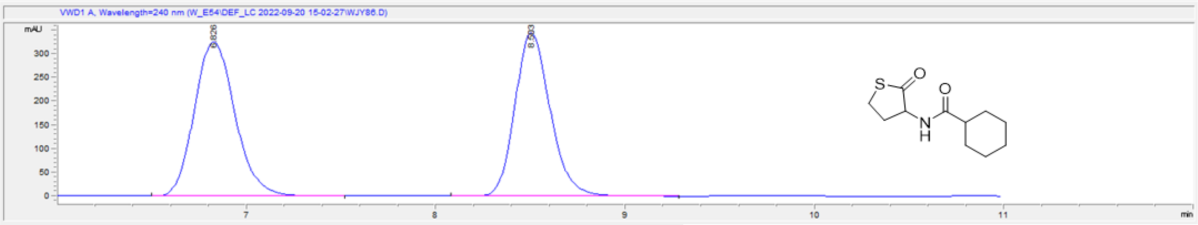

#	Time	Area	Height	Width	Area%	Symmetry
1	6.826	4859.9	325.9	0.2322	52.120	0.85
2	8.503	4464.5	345.1	0.2005	47.880	0.806

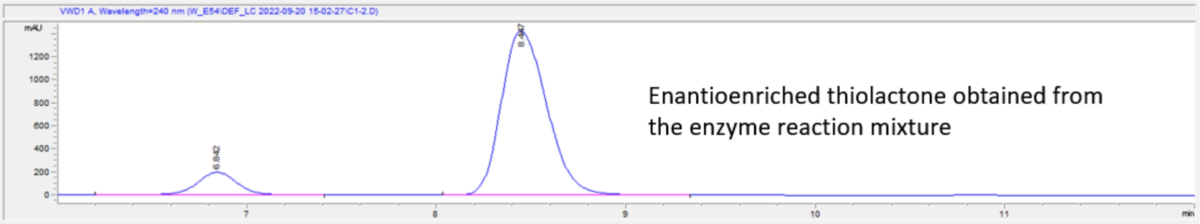

#	Time	Area	Height	Width	Area%	Symmetry
1	6.842	2932	198.2	0.2279	10.963	0.97
2	8.447	23811.6	1422.2	0.2638	89.037	0.733

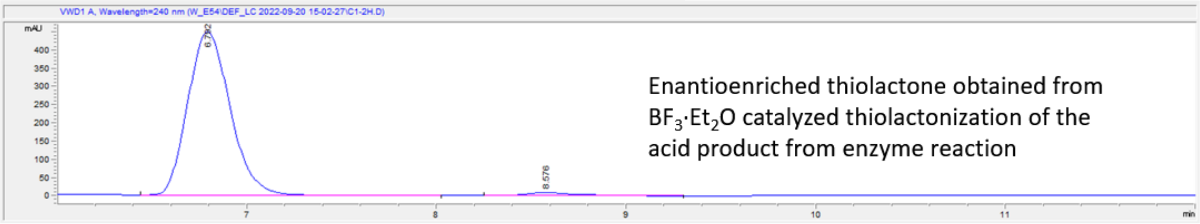

#	Time	Area	Height	Width	Area%	Symmetry
1	6.792	7038.9	449.5	0.2453	97.793	0.831
2	8.576	158.9	8.5	0.2732	2.207	0.538

HPLC conditions: Chiralpak® IC column (4.6 mm × 250 mm, 5 µm), 240 nm, 10% EtOH/hexane, 1.0 mL/min.

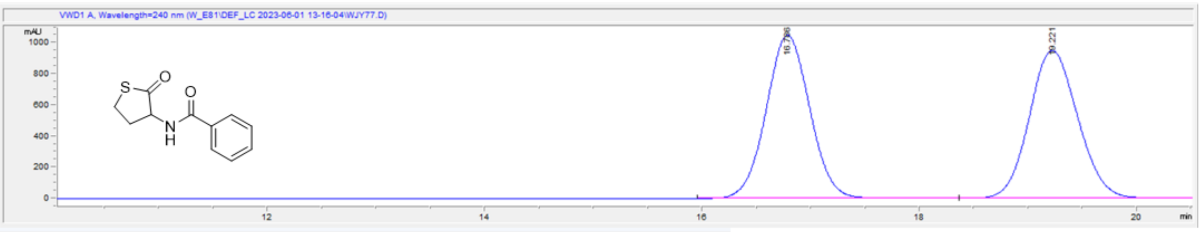

#	Time	Area	Height	Width	Area%	Symmetry
1	16.786	29180.2	1047.5	0.4298	49.797	0.946
2	19.221	29418.2	950.6	0.4761	50.203	0.864

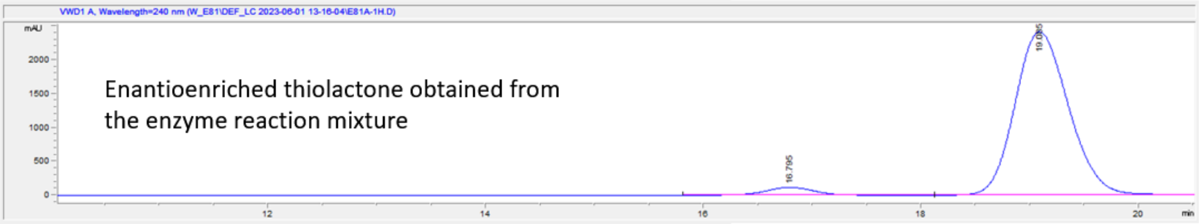

#	Time	Area	Height	Width	Area%	Symmetry
1	16.795	3492.2	114.1	0.4705	4.034	0.991
2	19.086	83080	2413.3	0.5414	95.966	0.793

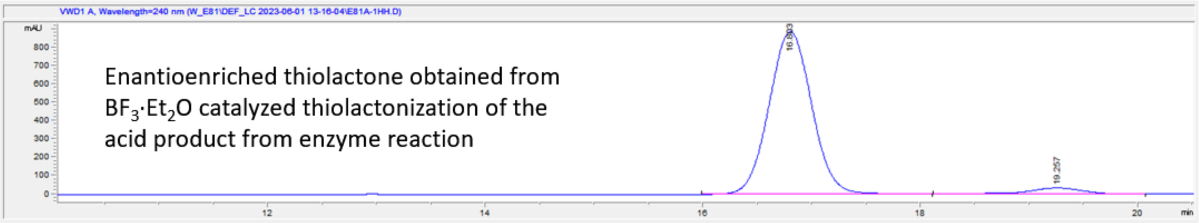

#	Time	Area	Height	Width	Area%	Symmetry
1	16.803	24297.1	888.8	0.4238	94.847	0.949
2	19.257	1320	34.5	0.572	5.153	1.303

HPLC conditions: Chiralpak® IG column (4.6 mm × 250 mm, 5 μm), 240 nm, 40% EtOH/hexane, 1.0 mL/min.

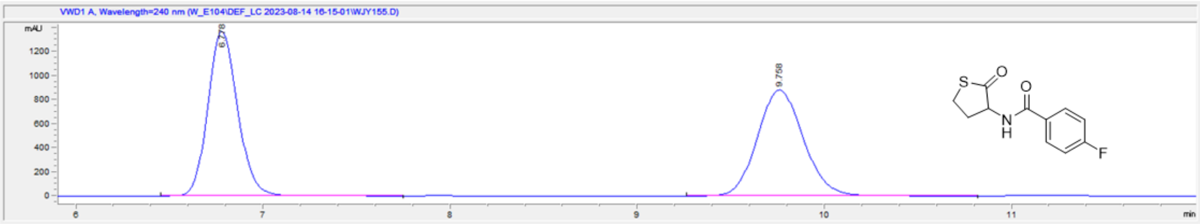

#	Time	Area	Height	Width	Area%	Symmetry
1	6.778	15087.2	1362.8	0.1714	50.042	0.839
2	9.758	15062.1	881.7	0.2663	49.958	0.88

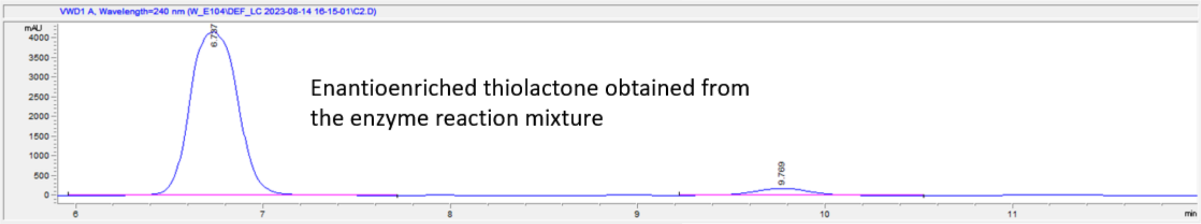

#	Time	Area	Height	Width	Area%	Symmetry
1	6.737	73013.3	4165.8	0.2176	95.180	0.98
2	9.769	3697.1	179.3	0.3207	4.820	1.005

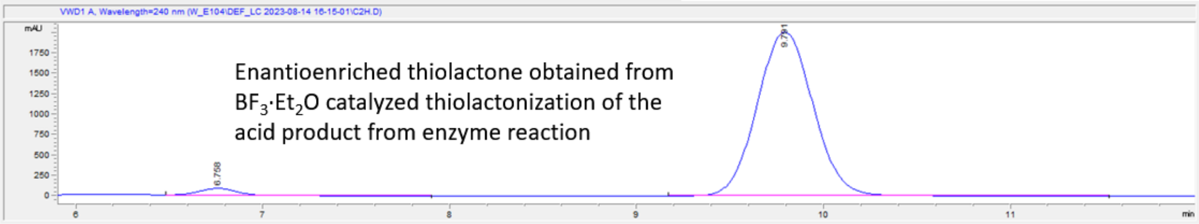

#	Time	Area	Height	Width	Area%	Symmetry
1	6.758	1428.9	91.1	0.2384	3.307	0.958
2	9.791	41774.8	2007.6	0.3272	96.693	0.936

HPLC conditions: Chiralpak® IG column (4.6 mm × 250 mm, 5 μm), 240 nm, 30% EtOH/hexane, 1.0 mL/min.

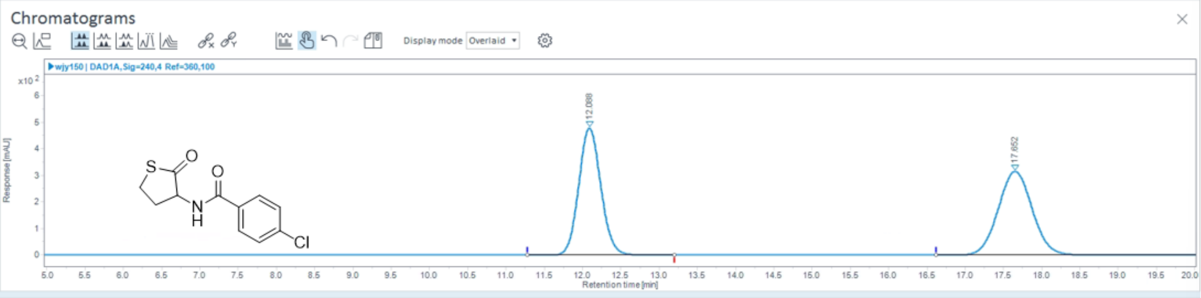

Injection Results

#	Name	Signal description	RT (min)	Area (mAU·s)	Area%	Height (mAU)	Height%	Amount	Concentration	Start time (min)	End time (min)
1		DAD1A,Sig=240,4 Ref=360,100	12.088	9607.254	49.579	478.884	60.32			11.281	13.200
2		DAD1A,Sig=240,4 Ref=360,100	17.652	9770.403	50.421	315.001	39.68			16.621	20.281

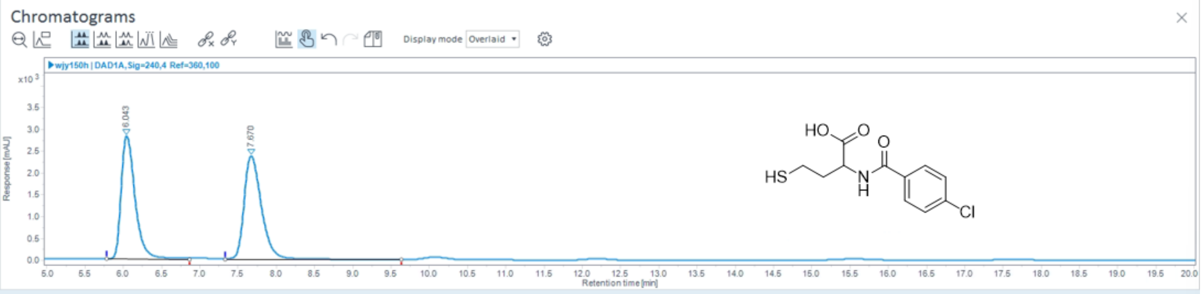

Injection Results

#	Name	Signal description	RT (min)	Area (mAU·s)	Area%	Height (mAU)	Height%	Amount	Concentration	Start time (min)	End time (min)
1		DAD1A,Sig=240,4 Ref=360,100	6.043	36124.217	48.945	2820.900	54.33			5.776	6.868
2		DAD1A,Sig=240,4 Ref=360,100	7.670	37681.261	51.055	2371.014	45.67			7.330	9.633

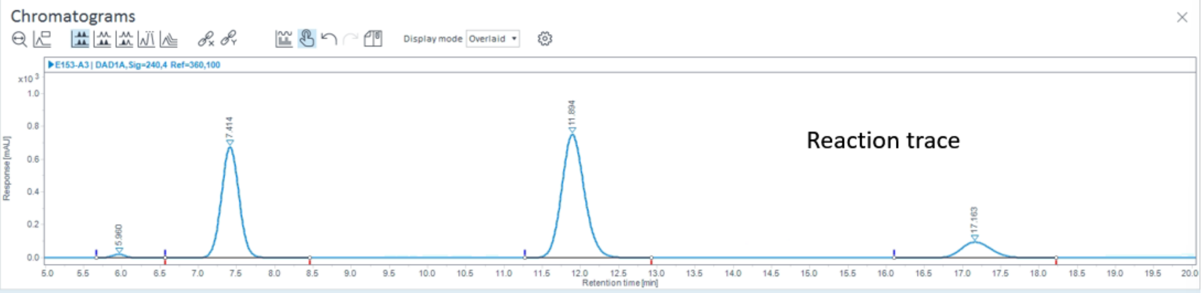

Injection Results

#	Name	Signal description	RT (min)	Area (mAU·s)	Area%	Height (mAU)	Height%	Amount	Concentration	Start time (min)	End time (min)
1		DAD1A,Sig=240,4 Ref=360,100	5.960	264.475	0.894	20.902	1.35			5.669	6.568
2		DAD1A,Sig=240,4 Ref=360,100	7.414	10931.343	36.951	676.318	43.74			6.569	8.459
3		DAD1A,Sig=240,4 Ref=360,100	11.894	15717.507	53.129	753.215	48.71			11.276	12.926
4		DAD1A,Sig=240,4 Ref=360,100	17.163	2670.335	9.026	95.812	6.20			16.109	18.229

HPLC conditions: Chiralpak® IG column (4.6 mm × 250 mm, 5 μm), 240 nm, 30% EtOH/hexane, 1.0 mL/min.

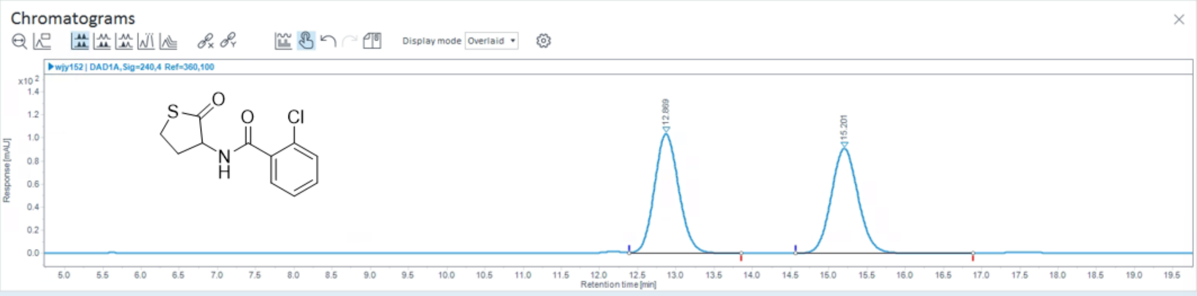

Injection Results

Peaks | Summary

#	Name	Signal description	RT (min)	Area (mAU·s)	Area%	Height (mAU)	Height%	Amount	Concentration	Start time (min)	End time (min)
1		DAD1A,Sig=240,4 Ref=360,100	12.869	2291.781	50.874	103.412	53.26			12.385	13.855
2		DAD1A,Sig=240,4 Ref=360,100	15.201	2213.058	49.126	90.751	46.74			14.561	16.881

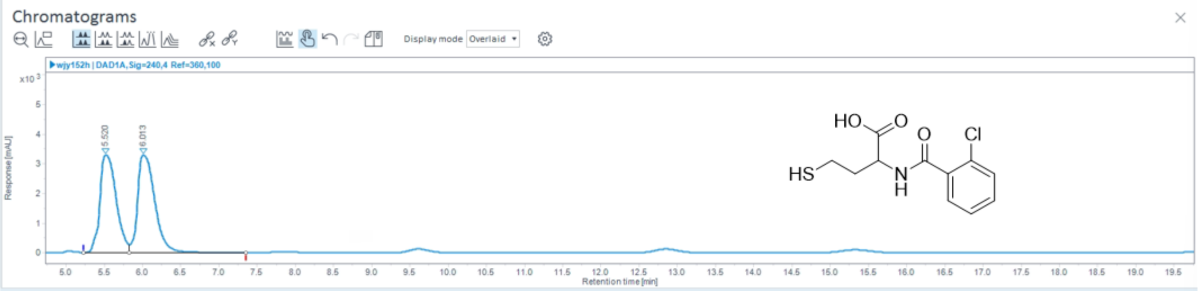

Injection Results

Peaks | Summary

#	Name	Signal description	RT (min)	Area (mAU·s)	Area%	Height (mAU)	Height%	Amount	Concentration	Start time (min)	End time (min)
1		DAD1A,Sig=240,4 Ref=360,100	5.520	49010.761	48.640	3298.685	50.10			5.227	5.828
2		DAD1A,Sig=240,4 Ref=360,100	6.013	51752.464	51.360	3285.820	49.90			5.828	7.349

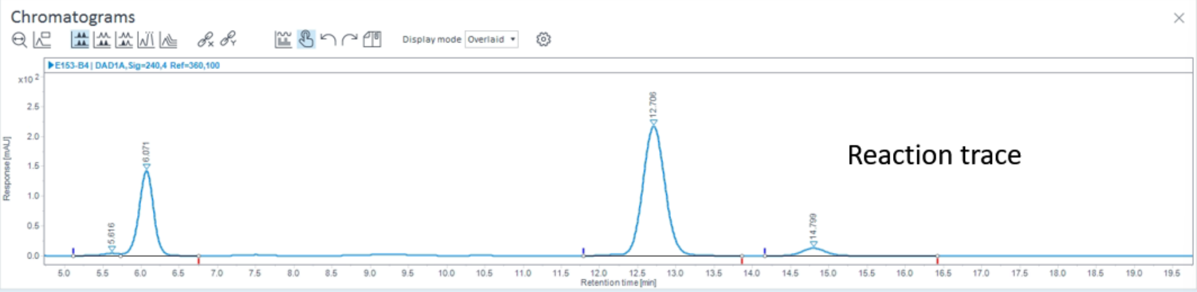

Injection Results

Peaks | Summary

#	Name	Signal description	RT (min)	Area (mAU·s)	Area%	Height (mAU)	Height%	Amount	Concentration	Start time (min)	End time (min)
1		DAD1A,Sig=240,4 Ref=360,100	5.616	61.032	0.915	4.215	1.12			5.118	5.735
2		DAD1A,Sig=240,4 Ref=360,100	6.071	1914.591	28.700	142.958	37.92			5.735	6.758
3		DAD1A,Sig=240,4 Ref=360,100	12.706	4418.543	66.235	217.206	57.61			11.784	13.864
4		DAD1A,Sig=240,4 Ref=360,100	14.799	276.868	4.150	12.660	3.36			14.164	16.417

HPLC conditions: Chiralpak® ID column (4.6 mm × 250 mm, 5 µm), 240 nm, 40% EtOH/hexane, 1.0 mL/min.

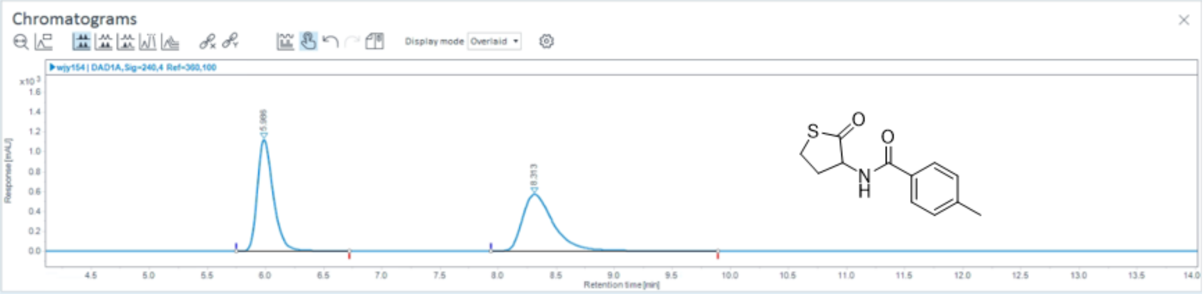

Injection Results

Peaks | Summary

#	Name	Signal description	RT (min)	Area (mAU·s)	Area%	Height (mAU)	Height%	Amount	Concentration	Start time (min)	End time (min)
1		DAD1A,Sig=240,4 Ref=360,100	5.986	10499.434	49.710	1122.627	66.28			5.746	6.721
2		DAD1A,Sig=240,4 Ref=360,100	8.313	10621.949	50.290	571.033	33.72			7.939	9.889

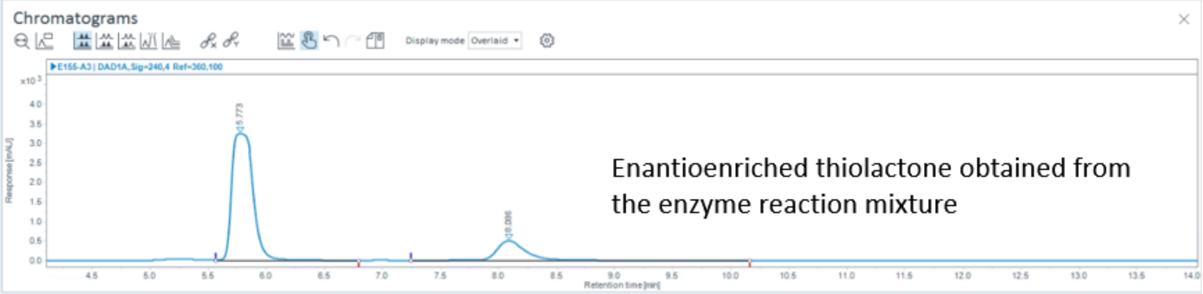

Injection Results

Peaks | Summary

#	Name	Signal description	RT (min)	Area (mAU·s)	Area%	Height (mAU)	Height%	Amount	Concentration	Start time (min)	End time (min)
1		DAD1A,Sig=240,4 Ref=360,100	5.773	41491.119	82.210	3243.218	86.46			5.566	6.796
2		DAD1A,Sig=240,4 Ref=360,100	8.086	8978.591	17.790	508.050	13.54			7.246	10.166

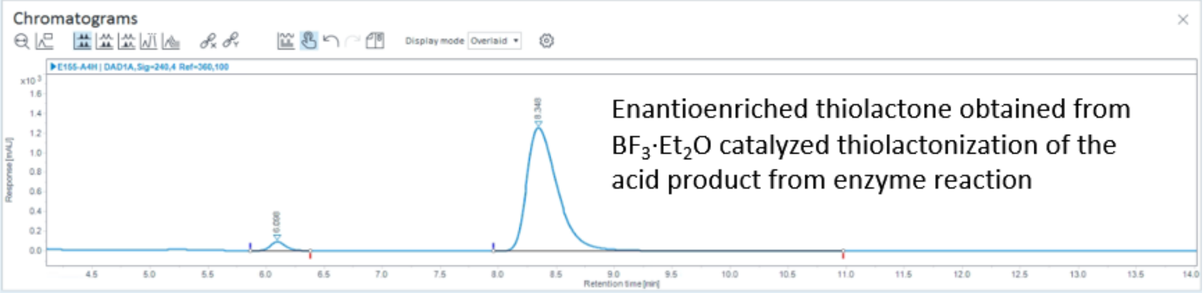

Injection Results

Peaks | Summary

#	Name	Signal description	RT (min)	Area (mAU·s)	Area%	Height (mAU)	Height%	Amount	Concentration	Start time (min)	End time (min)
1		DAD1A,Sig=240,4 Ref=360,100	6.098	814.013	3.402	91.291	6.79			5.864	6.383
2		DAD1A,Sig=240,4 Ref=360,100	8.348	23116.263	96.598	1253.346	93.21			7.961	10.975

HPLC conditions: Chiralpak® ID column (4.6 mm × 250 mm, 5 μm), 240 nm, 40% EtOH/hexane, 1.0 mL/min.

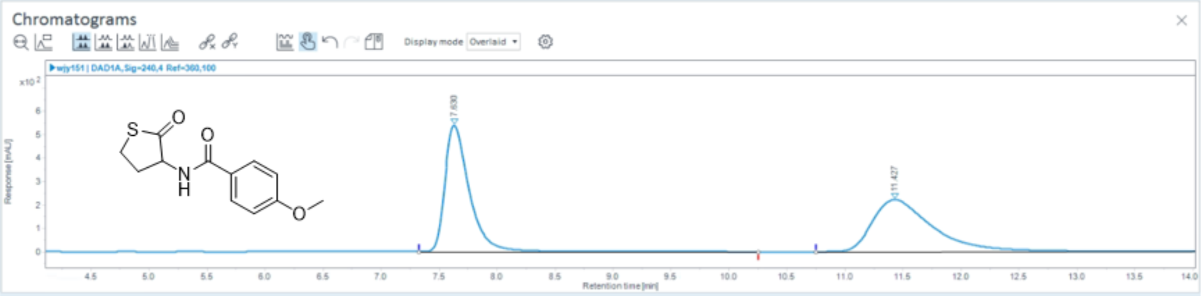

Injection Results

#	Name	Signal description	RT (min)	Area (mAU·s)	Area%	Height (mAU)	Height%	Amount	Concentration	Start time (min)	End time (min)
1		DAD1A,Sig=240,4 Ref=360,100	7.630	7952.061	49.354	541.074	70.89			7.324	10.254
2		DAD1A,Sig=240,4 Ref=360,100	11.427	8160.168	50.646	222.135	29.11			10.748	15.077

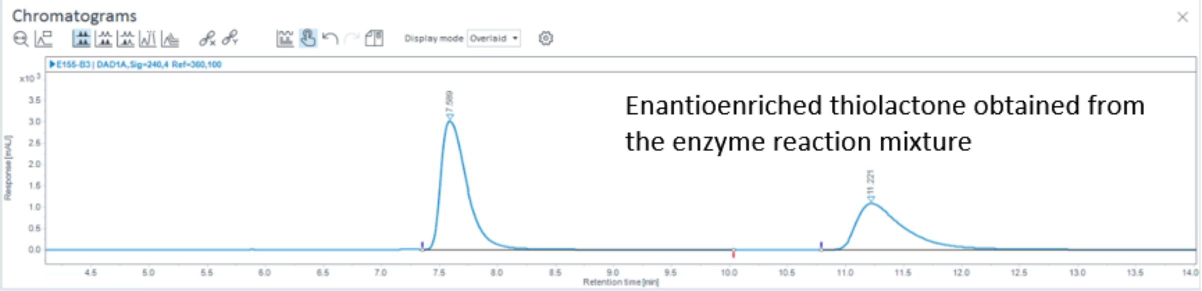

Injection Results

#	Name	Signal description	RT (min)	Area (mAU·s)	Area%	Height (mAU)	Height%	Amount	Concentration	Start time (min)	End time (min)
1		DAD1A,Sig=240,4 Ref=360,100	7.589	46835.590	59.669	3018.122	73.54			7.355	10.031
2		DAD1A,Sig=240,4 Ref=360,100	11.221	31657.351	40.331	1085.950	26.46			10.791	14.998

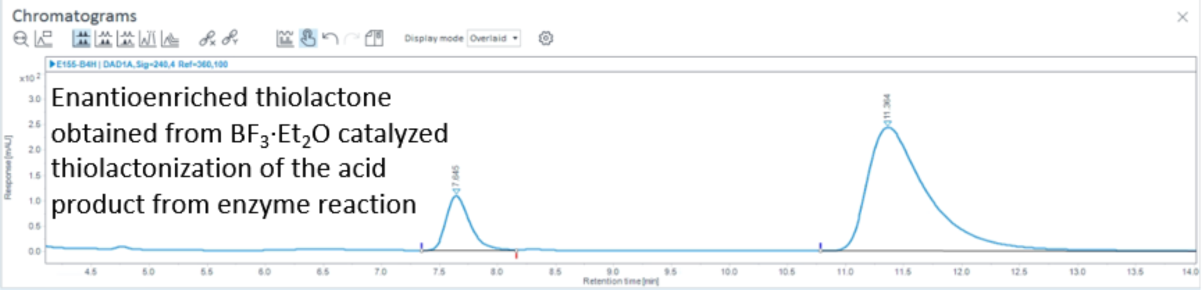

Injection Results

#	Name	Signal description	RT (min)	Area (mAU·s)	Area%	Height (mAU)	Height%	Amount	Concentration	Start time (min)	End time (min)
1		DAD1A,Sig=240,4 Ref=360,100	7.645	1555.125	14.891	108.175	30.66			7.346	8.161
2		DAD1A,Sig=240,4 Ref=360,100	11.364	8887.991	85.109	244.610	69.34			10.780	15.000

6.2. HPLC analysis for DKR catalysed by N9 Y71G

HPLC conditions: Chiralpak® IG column (4.6 mm × 250 mm, 5 µm), 240 nm, 10% EtOH/hexane, 1.0 mL/min.

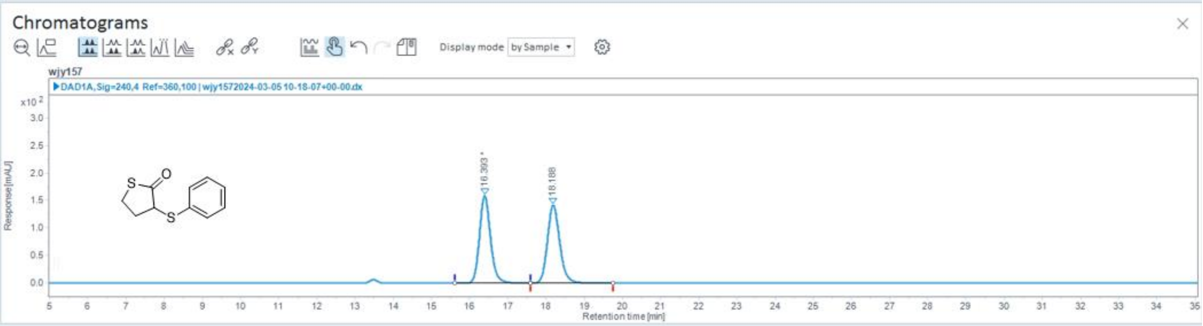

Injection Results

#	Name	Signal description	RT (min)	Area (mAU·s)	Area%	Height (mAU)	Height%	Amount	Concentration	Start time (min)	End time (min)
1		DAD1A,Sig=240,4 Ref=360,100	16.393	3223.379	49.985	159.678	52.76			15.603	17.598
2		DAD1A,Sig=240,4 Ref=360,100	18.188	3225.306	50.015	142.949	47.24			17.598	19.745

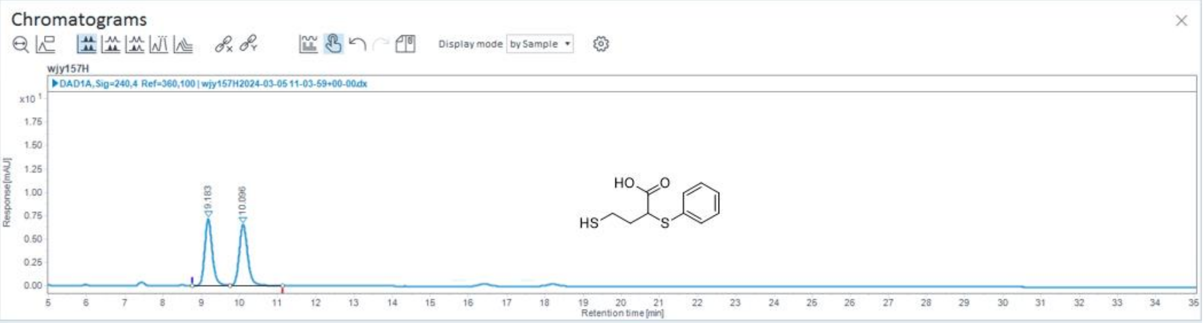

Injection Results

#	Name	Signal description	RT (min)	Area (mAU·s)	Area%	Height (mAU)	Height%	Amount	Concentration	Start time (min)	End time (min)
1		DAD1A,Sig=240,4 Ref=360,100	9.183	100.188	49.614	7.194	52.19			8.772	9.762
2		DAD1A,Sig=240,4 Ref=360,100	10.096	101.747	50.386	6.591	47.81			9.762	11.125

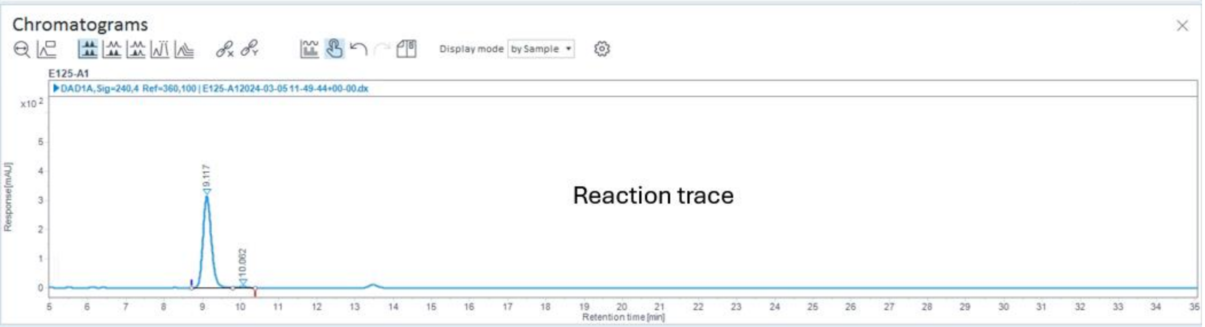

Injection Results

#	Name	Signal description	RT (min)	Area (mAU·s)	Area%	Height (mAU)	Height%	Amount	Concentration	Start time (min)	End time (min)
1		DAD1A,Sig=240,4 Ref=360,100	9.117	4781.413	98.345	313.809	98.52			8.709	9.790
2		DAD1A,Sig=240,4 Ref=360,100	10.062	80.458	1.655	4.727	1.48			9.790	10.392

HPLC conditions: Chiralpak® IG column (4.6 mm × 250 mm, 5 μm), 240 nm, 10% EtOH/hexane, 1.0 mL/min.

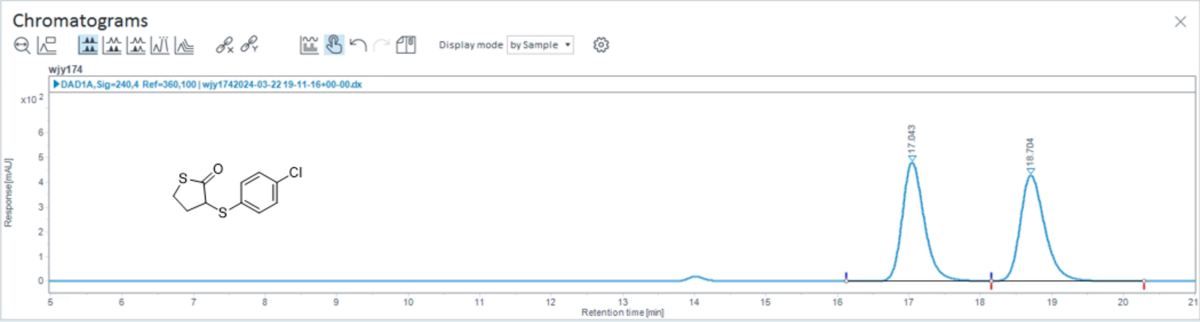

Injection Results

#	Name	Signal description	RT (min)	Area (mAU·s)	Area%	Height (mAU)	Height%	Amount	Concentration	Start time (min)	End time (min)
1		DAD1A,Sig=240,4 Ref=360,100	17.043	10239.667	50.099	480.311	52.89			16.123	18.148
2		DAD1A,Sig=240,4 Ref=360,100	18.704	10199.016	49.901	427.873	47.11			18.148	20.283

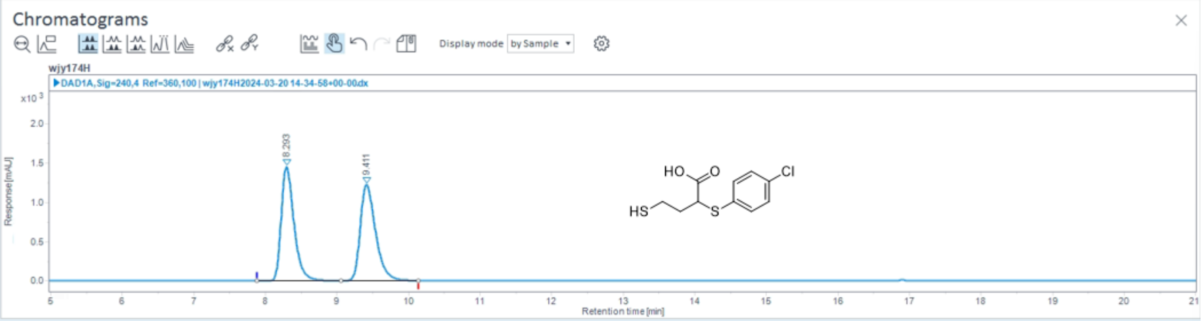

Injection Results

#	Name	Signal description	RT (min)	Area (mAU·s)	Area%	Height (mAU)	Height%	Amount	Concentration	Start time (min)	End time (min)
1		DAD1A,Sig=240,4 Ref=360,100	8.293	18219.005	49.676	1449.230	54.19			7.878	9.055
2		DAD1A,Sig=240,4 Ref=360,100	9.411	18456.919	50.324	1225.294	45.81			9.055	10.131

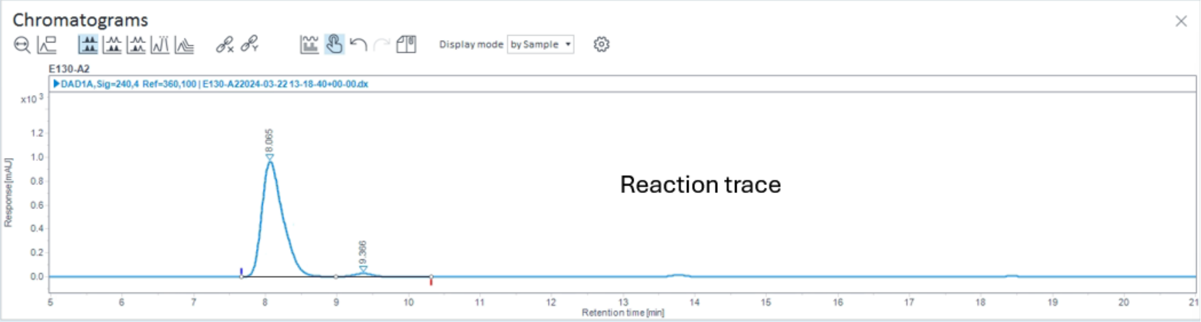

Injection Results

#	Name	Signal description	RT (min)	Area (mAU·s)	Area%	Height (mAU)	Height%	Amount	Concentration	Start time (min)	End time (min)
1		DAD1A,Sig=240,4 Ref=360,100	8.065	19896.610	97.348	963.780	97.15			7.658	8.985
2		DAD1A,Sig=240,4 Ref=360,100	9.366	541.947	2.652	28.295	2.85			8.985	10.310

HPLC conditions: Chiralpak® IG column (4.6 mm × 250 mm, 5 µm), 240 nm, 10% EtOH/hexane, 1.0 mL/min.

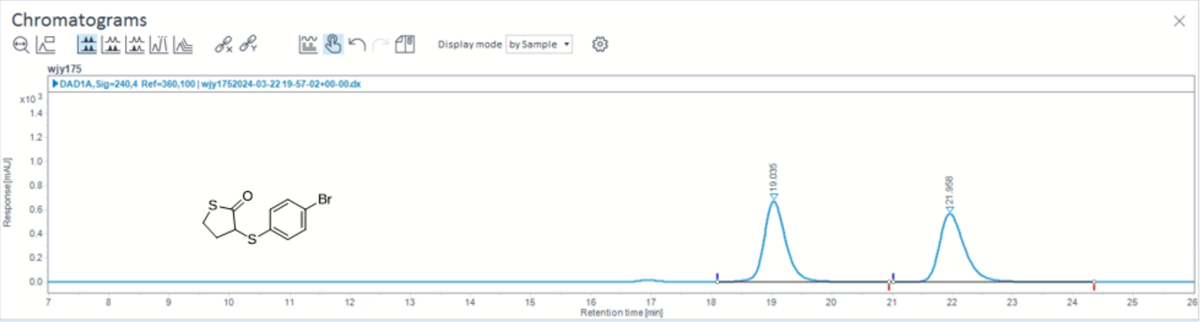

Injection Results

#	Name	Signal description	RT (min)	Area (mAU·s)	Area%	Height (mAU)	Height%	Amount	Concentration	Start time (min)	End time (min)
1		DAD1A,Sig=240,4 Ref=360,100	19.035	16732.102	50.594	670.506	54.19			18.095	20.945
2		DAD1A,Sig=240,4 Ref=360,100	21.958	16339.534	49.406	566.877	45.81			21.018	24.355

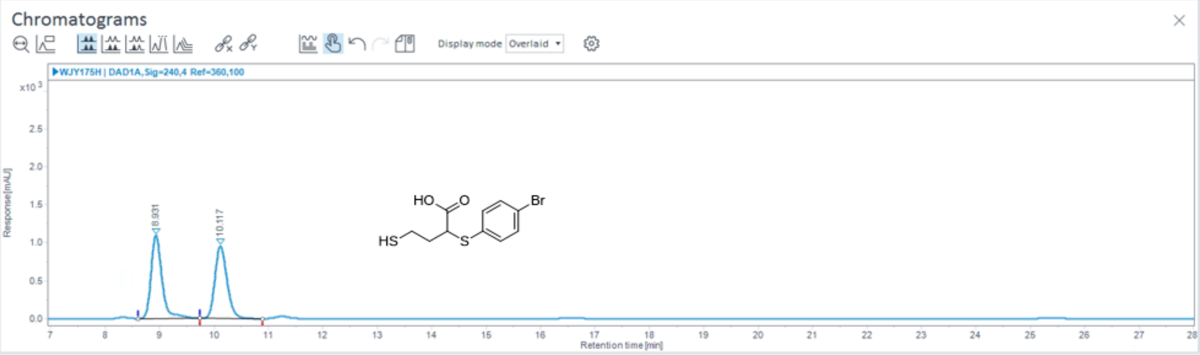

Injection Results

#	Name	Signal description	RT (min)	Area (mAU·s)	Area%	Height (mAU)	Height%	Amount	Concentration	Start time (min)	End time (min)
1		DAD1A,Sig=240,4 Ref=360,100	8.931	15433.024	51.166	1099.153	53.47			8.598	9.735
2		DAD1A,Sig=240,4 Ref=360,100	10.117	14729.600	48.834	956.585	46.53			9.736	10.896

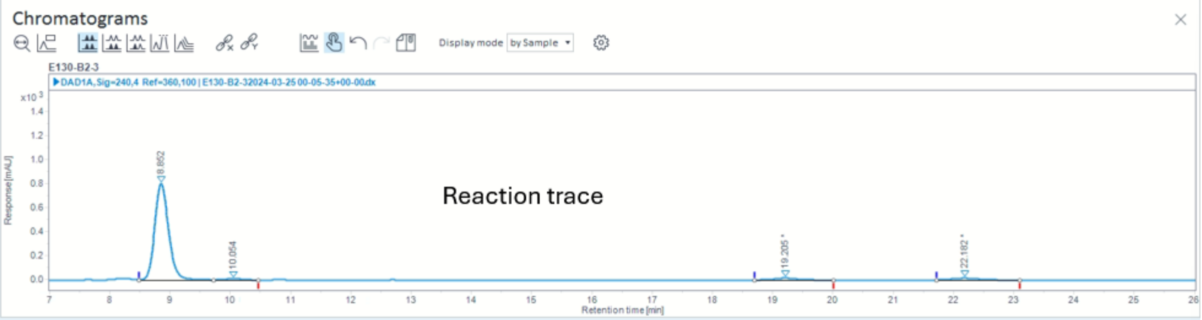

Injection Results

#	Name	Signal description	RT (min)	Area (mAU·s)	Area%	Height (mAU)	Height%	Amount	Concentration	Start time (min)	End time (min)
1		DAD1A,Sig=240,4 Ref=360,100	8.852	12815.274	92.468	805.582	94.82			8.490	9.724
2		DAD1A,Sig=240,4 Ref=360,100	10.054	211.484	1.526	13.180	1.55			9.724	10.464
3		DAD1A,Sig=240,4 Ref=360,100	19.205	391.849	2.827	15.830	1.86			18.692	20.005
4		DAD1A,Sig=240,4 Ref=360,100	22.182	440.596	3.179	14.981	1.76			21.722	23.103

HPLC conditions: Chiralcel® OJ-H column (4.6 mm × 250 mm, 5 μm), 240 nm, 20% EtOH/hexane, 1.0 mL/min.

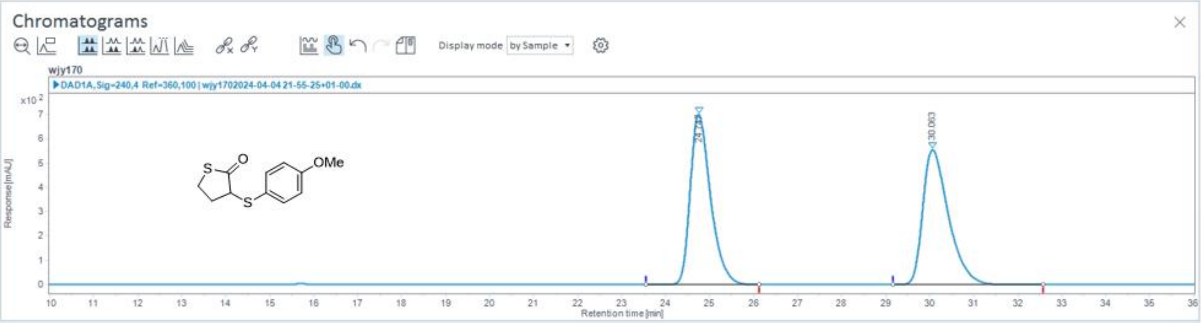

Injection Results

Peaks Summary

#	Name	Signal description	RT (min)	Area (mAU·s)	Area%	Height (mAU)	Height%	Amount	Concentration	Start time (min)	End time (min)
1		DAD1A,Sig=240,4 Ref=360,100	24.747	22463.840	50.028	701.231	55.85			23.545	26.128
2		DAD1A,Sig=240,4 Ref=360,100	30.063	22438.704	49.972	554.368	44.15			29.171	32.571

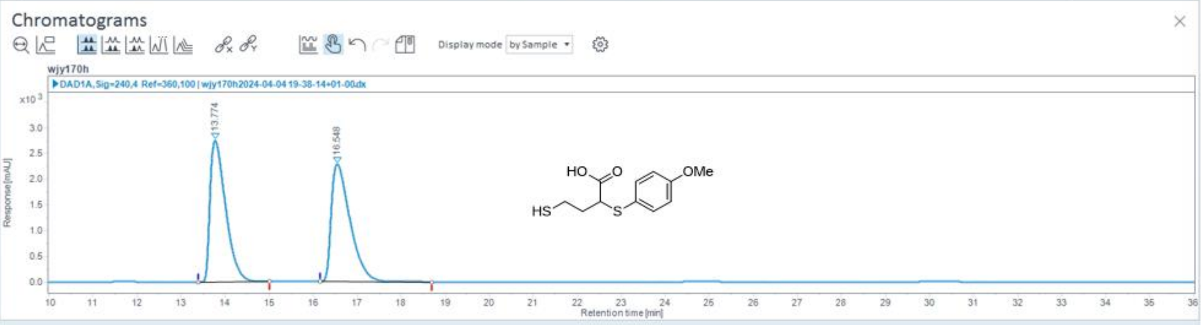

Injection Results

Peaks Summary

#	Name	Signal description	RT (min)	Area (mAU·s)	Area%	Height (mAU)	Height%	Amount	Concentration	Start time (min)	End time (min)
1		DAD1A,Sig=240,4 Ref=360,100	13.774	67642.097	49.879	2751.646	54.58			13.391	15.009
2		DAD1A,Sig=240,4 Ref=360,100	16.548	67970.450	50.121	2289.916	45.42			16.158	18.691

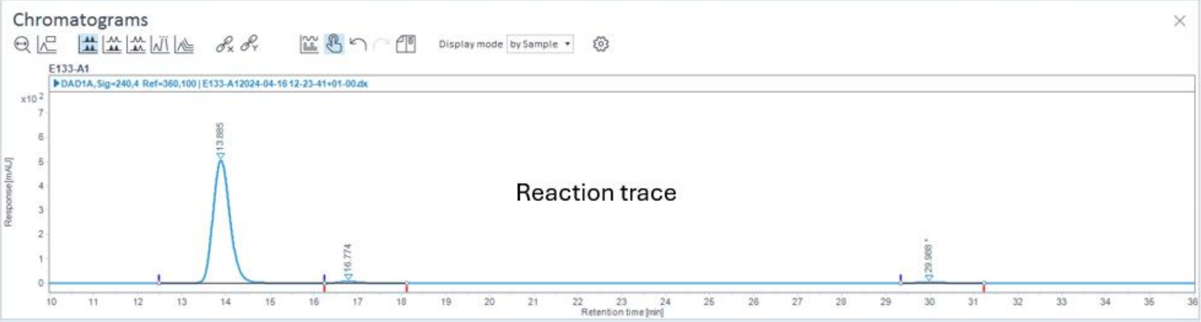

Injection Results

Peaks Summary

#	Name	Signal description	RT (min)	Area (mAU·s)	Area%	Height (mAU)	Height%	Amount	Concentration	Start time (min)	End time (min)
1		DAD1A,Sig=240,4 Ref=360,100	13.885	13608.003	97.493	505.122	97.94			12.472	16.239
2		DAD1A,Sig=240,4 Ref=360,100	16.774	201.715	1.445	6.919	1.34			16.246	18.106
3		DAD1A,Sig=240,4 Ref=360,100	29.988	148.255	1.062	3.719	0.72			29.339	31.225

HPLC conditions: Chiralpak® IG column (4.6 mm × 250 mm, 5 μm), 240 nm, 10% EtOH/hexane, 1.0 mL/min.

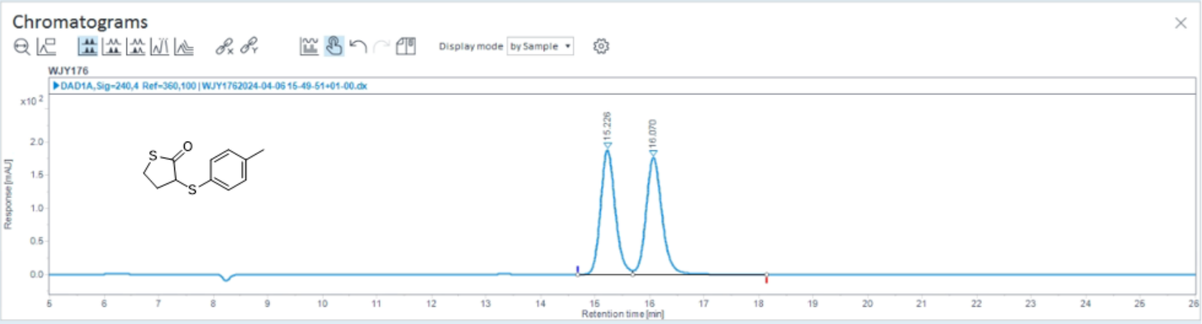

Injection Results

#	Name	Signal description	RT (min)	Area (mAU·s)	Area%	Height (mAU)	Height%	Amount	Concentration	Start time (min)	End time (min)
1		DAD1A,Sig=240,4 Ref=360,100	15.226	3507.977	49.500	187.580	51.53			14.679	15.695
2		DAD1A,Sig=240,4 Ref=360,100	16.070	3578.868	50.500	176.468	48.47			15.695	18.149

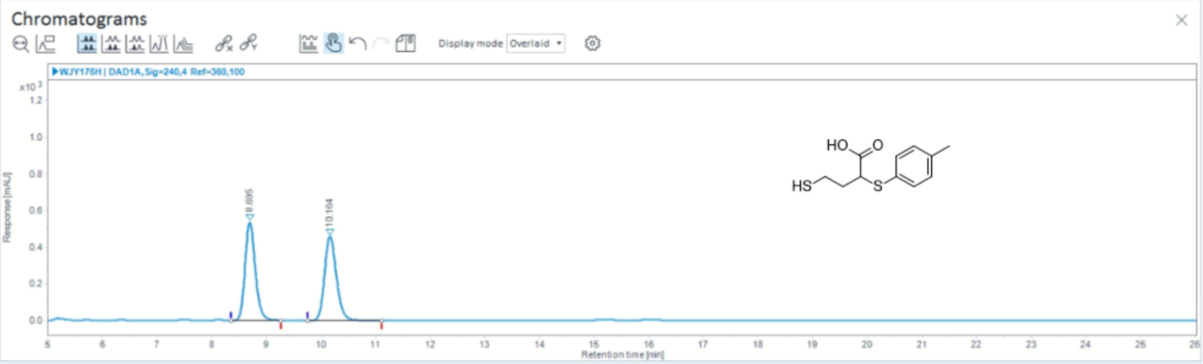

Injection Results

#	Name	Signal description	RT (min)	Area (mAU·s)	Area%	Height (mAU)	Height%	Amount	Concentration	Start time (min)	End time (min)

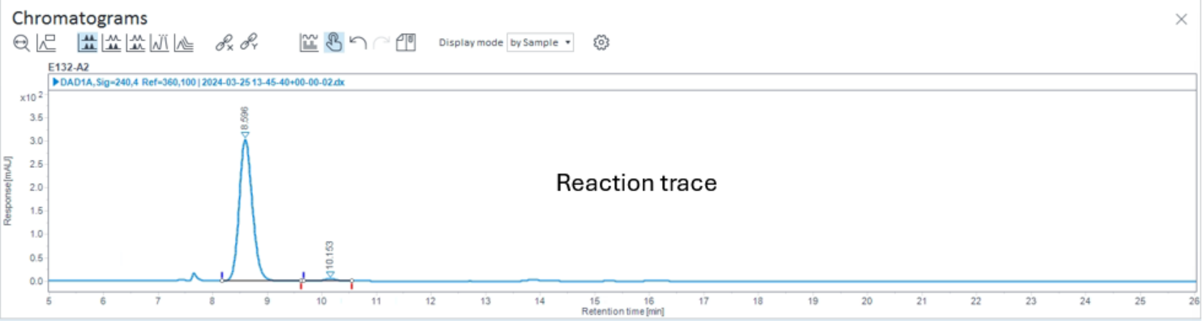

Injection Results

#	Name	Signal description	RT (min)	Area (mAU·s)	Area%	Height (mAU)	Height%	Amount	Concentration	Start time (min)	End time (min)
1		DAD1A,Sig=240,4 Ref=360,100	8.596	5111.047	98.546	302.679	98.41			8.168	9.615
2		DAD1A,Sig=240,4 Ref=360,100	10.153	75.415	1.454	4.899	1.59			9.662	10.552

HPLC conditions: Chiralpak® IG column (4.6 mm × 250 mm, 5 µm), 240 nm, 10% EtOH/hexane, 1.0 mL/min.

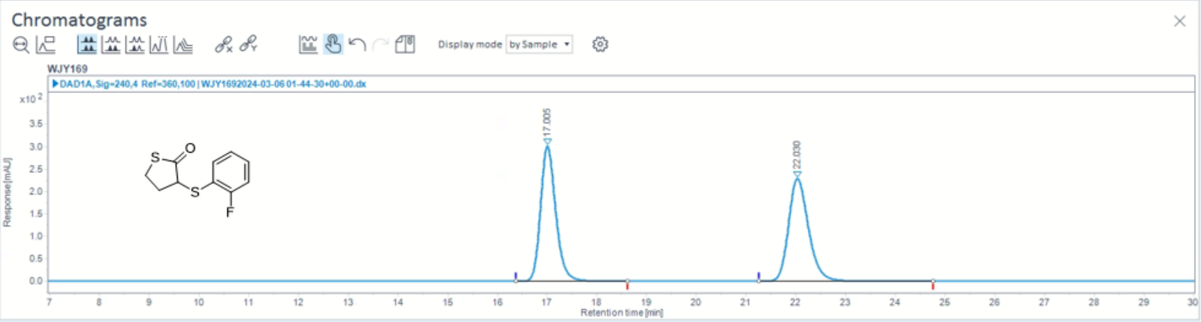

Injection Results

#	Name	Signal description	RT (min)	Area (mAU·s)	Area%	Height (mAU)	Height%	Amount	Concentration	Start time (min)	End time (min)
1		DAD1A,Sig=240,4 Ref=360,100	17.005	6412.444	50.015	300.616	56.77			16.378	18.618
2		DAD1A,Sig=240,4 Ref=360,100	22.030	6408.642	49.985	228.923	43.23			21.258	24.752

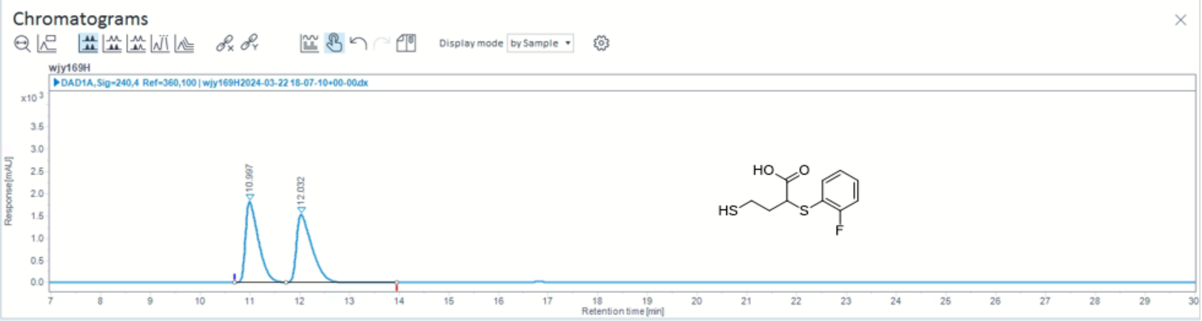

Injection Results

#	Name	Signal description	RT (min)	Area (mAU·s)	Area%	Height (mAU)	Height%	Amount	Concentration	Start time (min)	End time (min)
1		DAD1A,Sig=240,4 Ref=360,100	10.997	33018.628	49.867	1814.078	54.31			10.689	11.736
2		DAD1A,Sig=240,4 Ref=360,100	12.032	33194.244	50.133	1526.198	45.69			11.736	13.948

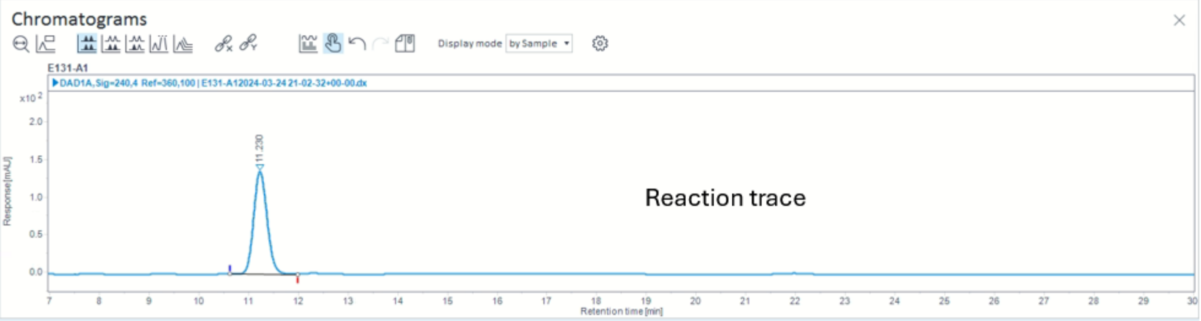

Injection Results

#	Name	Signal description	RT (min)	Area (mAU·s)	Area%	Height (mAU)	Height%	Amount	Concentration	Start time (min)	End time (min)
1		DAD1A,Sig=240,4 Ref=360,100	11.230	2557.031	100.000	135.998	100.00			10.620	11.993

HPLC conditions: Chiralpak® IG column (4.6 mm × 250 mm, 5 µm), 240 nm, 10% EtOH/hexane, 1.0 mL/min.

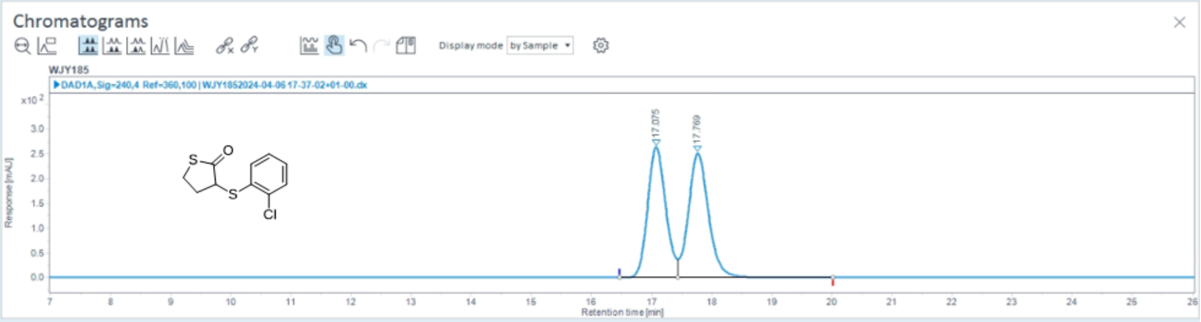

Injection Results

Peaks | Summary

#	Name	Signal description	RT (min)	Area (mAU·s)	Area%	Height (mAU)	Height%	Amount	Concentration	Start time (min)	End time (min)
1		DAD1A,Sig=240,4 Ref=360,100	17.075	5513.311	48.533	263.383	51.21			16.469	17.438
2		DAD1A,Sig=240,4 Ref=360,100	17.769	5846.498	51.467	250.950	48.79			17.438	20.022

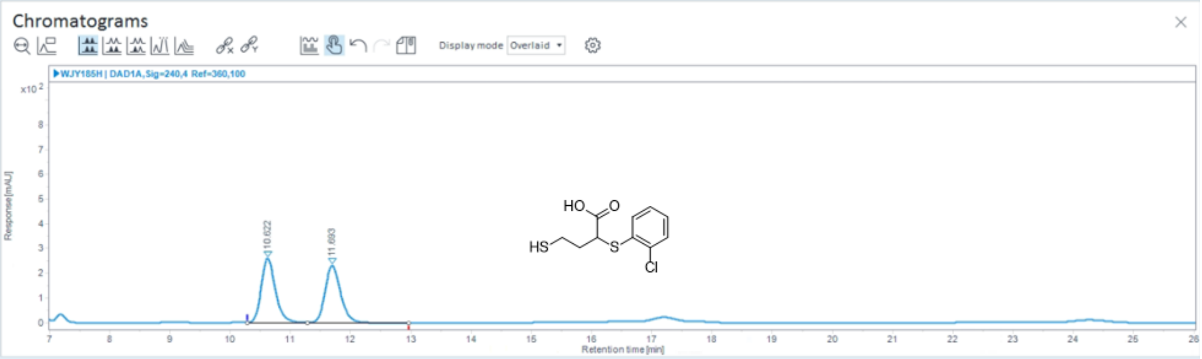

Injection Results

Peaks | Summary

#	Name	Signal description	RT (min)	Area (mAU·s)	Area%	Height (mAU)	Height%	Amount	Concentration	Start time (min)	End time (min)
1		DAD1A,Sig=240,4 Ref=360,100	10.622	4323.236	50.681	261.508	53.02			10.287	11.281
2		DAD1A,Sig=240,4 Ref=360,100	11.693	4207.038	49.319	231.695	46.98			11.281	12.958

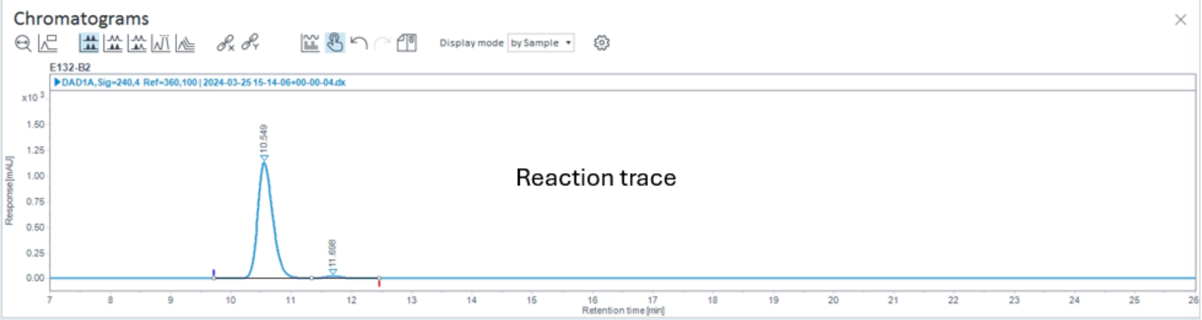

Injection Results

Peaks | Summary

#	Name	Signal description	RT (min)	Area (mAU·s)	Area%	Height (mAU)	Height%	Amount	Concentration	Start time (min)	End time (min)
1		DAD1A,Sig=240,4 Ref=360,100	10.549	19725.083	97.707	1132.072	97.95			9.712	11.334
2		DAD1A,Sig=240,4 Ref=360,100	11.698	462.809	2.293	23.729	2.05			11.334	12.458

HPLC conditions: Chiralpak® IG column (4.6 mm × 250 mm, 5 μm), 240 nm, 10% EtOH/hexane, 1.0 mL/min.

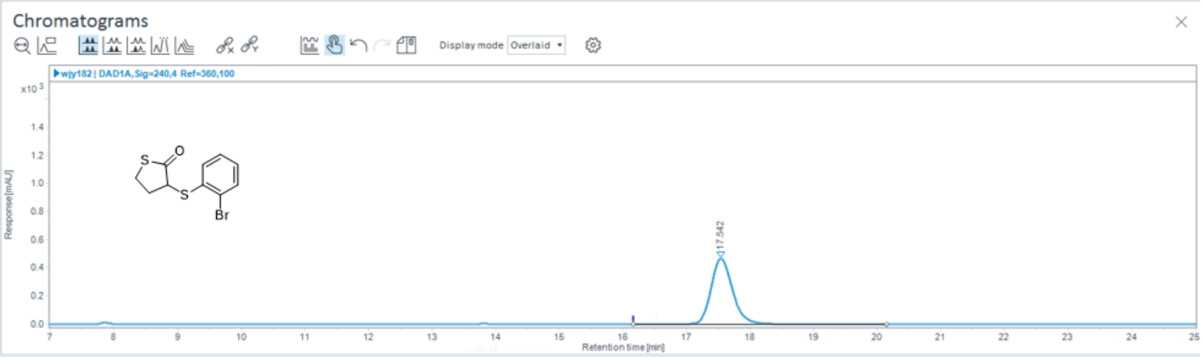

Injection Results

Peaks Summary

#	Name	Signal description	RT (min)	Area (mAU·s)	Area%	Height (mAU)	Height%	Amount	Concentration	Start time (min)	End time (min)
1		DAD1A,Sig=240,4 Ref=360,100	17.542	11064.110	100.000	468.697	100.00			16.162	20.149

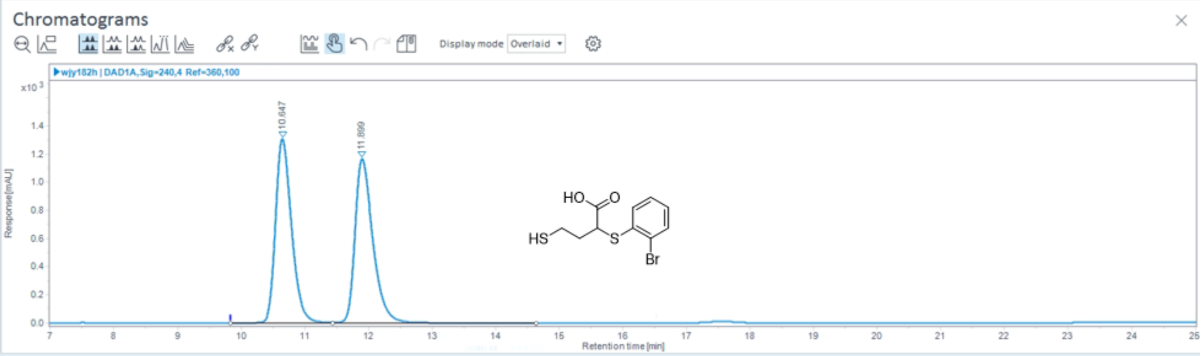

Injection Results

Peaks Summary

#	Name	Signal description	RT (min)	Area (mAU·s)	Area%	Height (mAU)	Height%	Amount	Concentration	Start time (min)	End time (min)
1		DAD1A,Sig=240,4 Ref=360,100	10.647	22761.126	49.937	1310.112	52.89			9.831	11.442
2		DAD1A,Sig=240,4 Ref=360,100	11.899	22818.353	50.063	1167.125	47.11			11.442	14.637

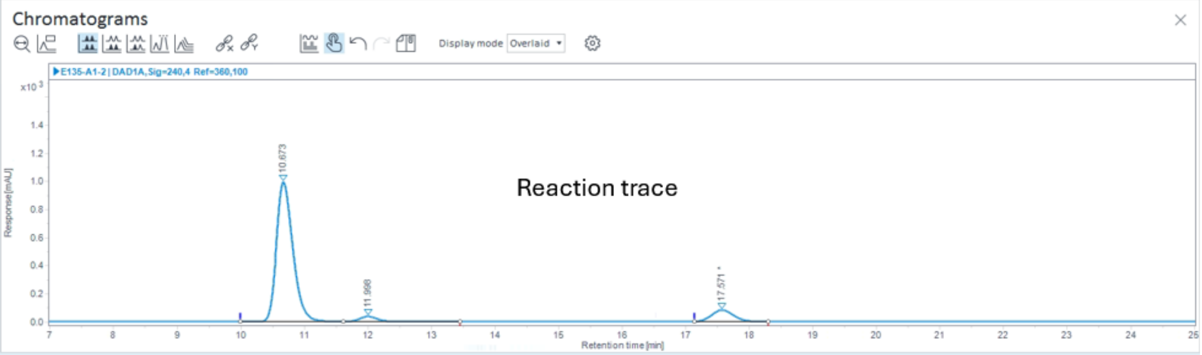

Injection Results

Peaks Summary

#	Name	Signal description	RT (min)	Area (mAU·s)	Area%	Height (mAU)	Height%	Amount	Concentration	Start time (min)	End time (min)
1		DAD1A,Sig=240,4 Ref=360,100	10.673	17978.839	86.396	994.861	89.10			9.986	11.606
2		DAD1A,Sig=240,4 Ref=360,100	11.998	799.840	3.844	38.133	3.42			11.606	13.445
3		DAD1A,Sig=240,4 Ref=360,100	17.571	2031.021	9.760	83.518	7.48			17.131	18.300

HPLC conditions: Chiralpak® IG column (4.6 mm × 250 mm, 5 µm), 240 nm, 5% EtOH/hexane, 1.0 mL/min.

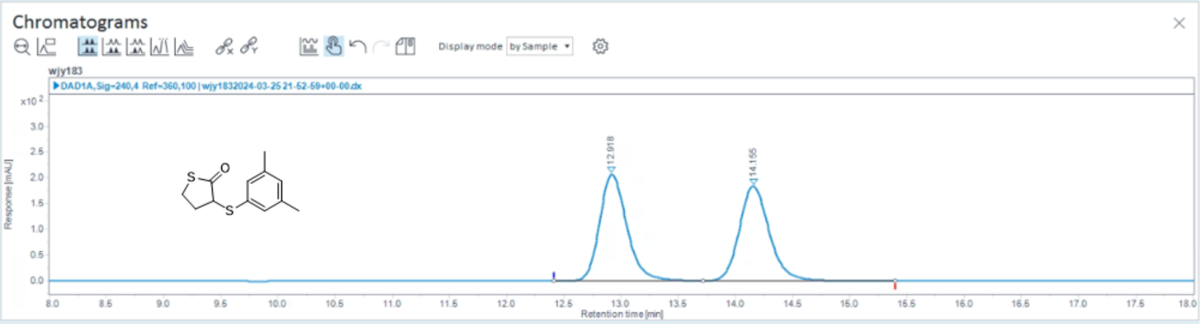

Injection Results

#	Name	Signal description	RT (min)	Area (mAU·s)	Area%	Height (mAU)	Height%	Amount	Concentration	Start time (min)	End time (min)
1		DAD1A,Sig=240,4 Ref=360,100	12.918	3436.876	50.455	206.869	52.88			12.410	13.715
2		DAD1A,Sig=240,4 Ref=360,100	14.155	3374.904	49.545	184.348	47.12			13.715	15.396

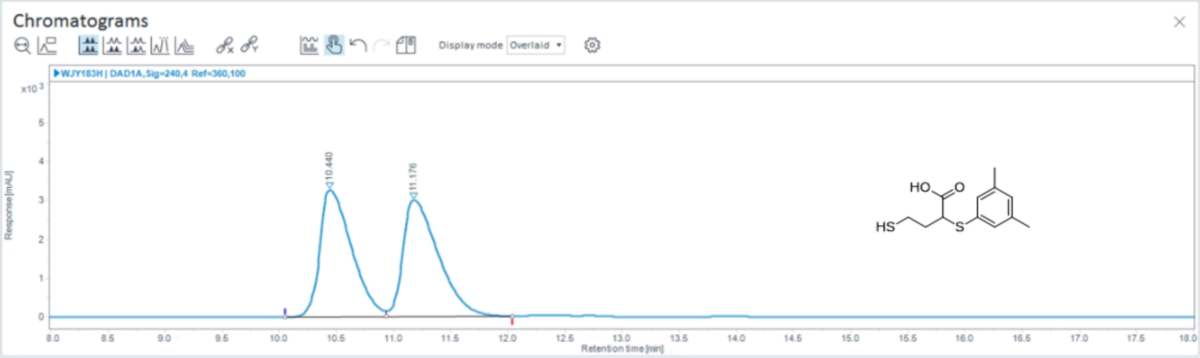

Injection Results

#	Name	Signal description	RT (min)	Area (mAU·s)	Area%	Height (mAU)	Height%	Amount	Concentration	Start time (min)	End time (min)
1		DAD1A,Sig=240,4 Ref=360,100	10.440	63177.737	49.374	3260.703	52.03			10.051	10.936
2		DAD1A,Sig=240,4 Ref=360,100	11.176	64778.925	50.626	3006.234	47.97			10.936	12.034

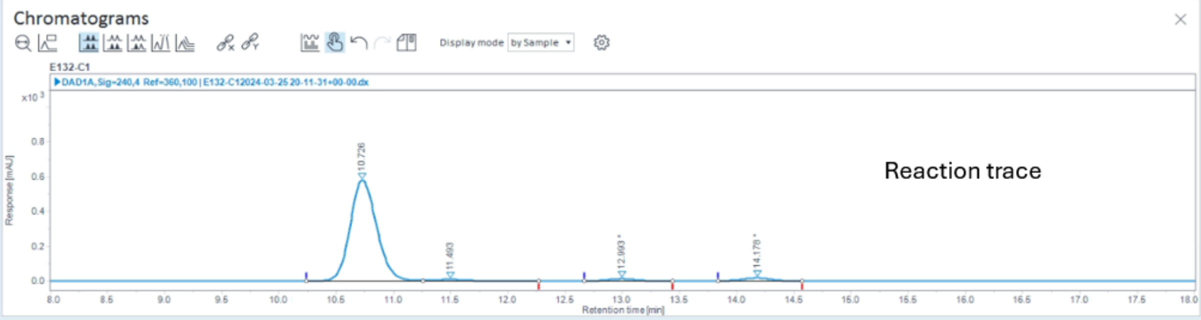

Injection Results

#	Name	Signal description	RT (min)	Area (mAU·s)	Area%	Height (mAU)	Height%	Amount	Concentration	Start time (min)	End time (min)
1		DAD1A,Sig=240,4 Ref=360,100	10.726	9368.343	93.863	580.962	93.98			10.236	11.258
2		DAD1A,Sig=240,4 Ref=360,100	11.493	154.563	1.549	8.146	1.32			11.258	12.271
3		DAD1A,Sig=240,4 Ref=360,100	12.993	186.656	1.870	12.013	1.94			12.664	13.440
4		DAD1A,Sig=240,4 Ref=360,100	14.178	271.313	2.718	17.040	2.76			13.835	14.567

HPLC conditions: Chiralcel® OJ-H column (4.6 mm × 250 mm, 5 μm), 240 nm, 20% EtOH/hexane, 1.0 mL/min.

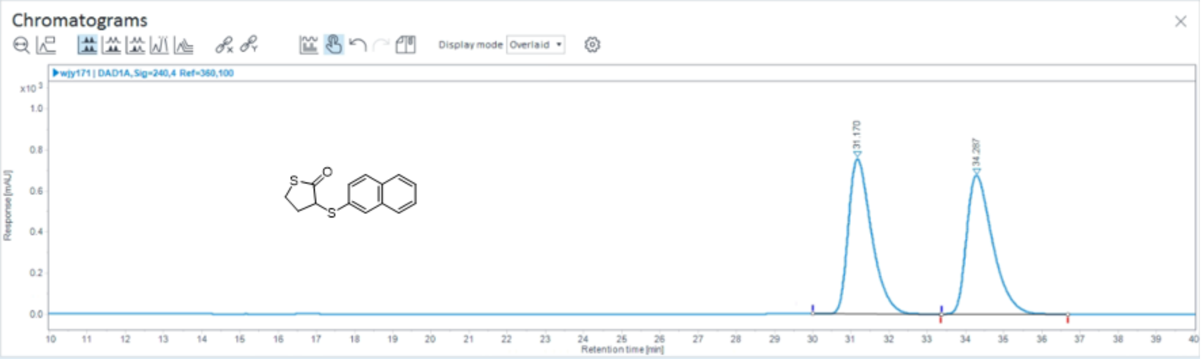

Injection Results

#	Name	Signal description	RT (min)	Area (mAU·s)	Area%	Height (mAU)	Height%	Amount	Concentration	Start time (min)	End time (min)
1		DAD1A,Sig=240,4 Ref=360,100	31.170	32054.209	49.989	755.682	52.80			29.991	33.351
2		DAD1A,Sig=240,4 Ref=360,100	34.287	32068.022	50.011	675.601	47.20			33.363	36.671

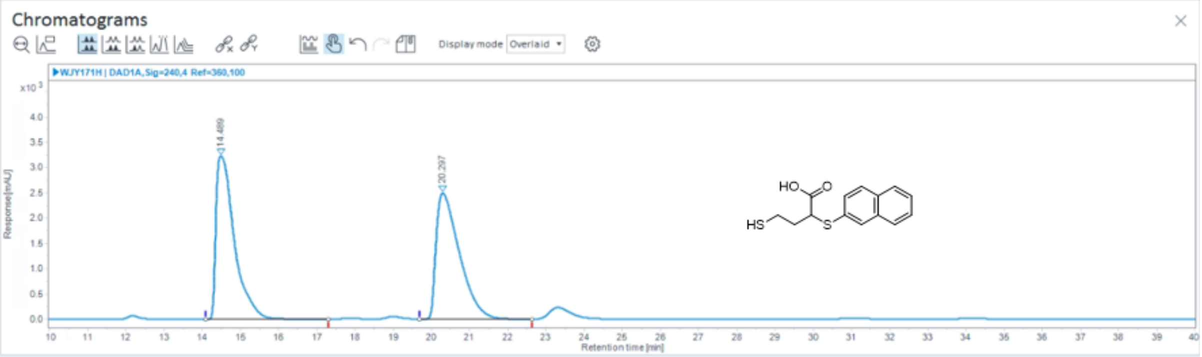

Injection Results

#	Name	Signal description	RT (min)	Area (mAU·s)	Area%	Height (mAU)	Height%	Amount	Concentration	Start time (min)	End time (min)
1		DAD1A,Sig=240,4 Ref=360,100	14.489	107678.402	50.543	3224.794	56.41			14.092	17.300
2		DAD1A,Sig=240,4 Ref=360,100	20.297	105366.741	49.457	2491.835	43.59			19.682	22.625

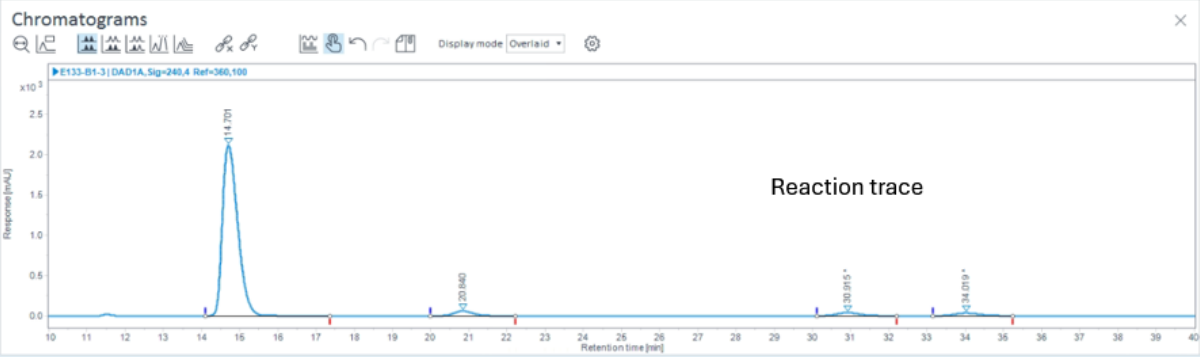

Injection Results

#	Name	Signal description	RT (min)	Area (mAU·s)	Area%	Height (mAU)	Height%	Amount	Concentration	Start time (min)	End time (min)
1		DAD1A,Sig=240,4 Ref=360,100	14.701	58774.173	91.704	2119.478	94.03			14.105	17.365
2		DAD1A,Sig=240,4 Ref=360,100	20.840	2049.575	3.198	59.295	2.63			19.991	22.208
3		DAD1A,Sig=240,4 Ref=360,100	30.915	1668.069	2.603	40.034	1.78			30.112	32.200
4		DAD1A,Sig=240,4 Ref=360,100	34.019	1599.186	2.495	35.195	1.56			33.143	35.231

HPLC conditions: Chiralpak® IG column (4.6 mm × 250 mm, 5 μm), 240 nm, 5% EtOH/hexane, 1.0 mL/min.

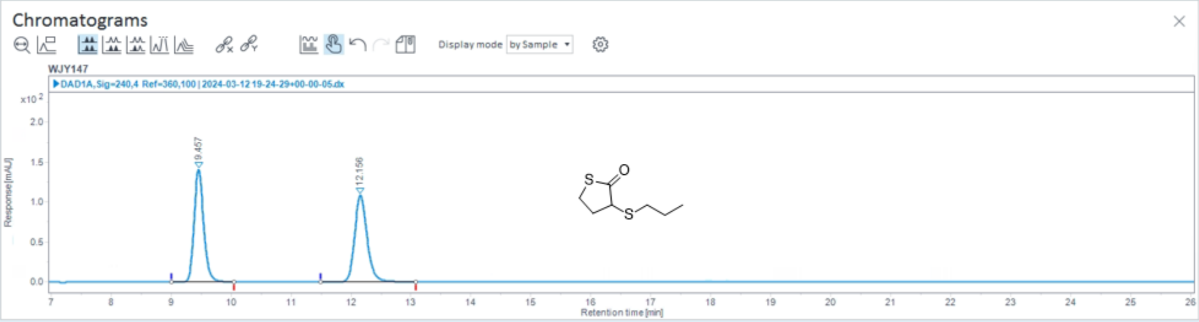

Injection Results

#	Name	Signal description	RT (min)	Area (mAU·s)	Area%	Height (mAU)	Height%	Amount	Concentration	Start time (min)	End time (min)
1		DAD1A,Sig=240,4 Ref=360,100	9.457	1555.332	49.865	140.843	56.43			8.998	10.048
2		DAD1A,Sig=240,4 Ref=360,100	12.156	1563.768	50.135	108.725	43.57			11.492	13.075

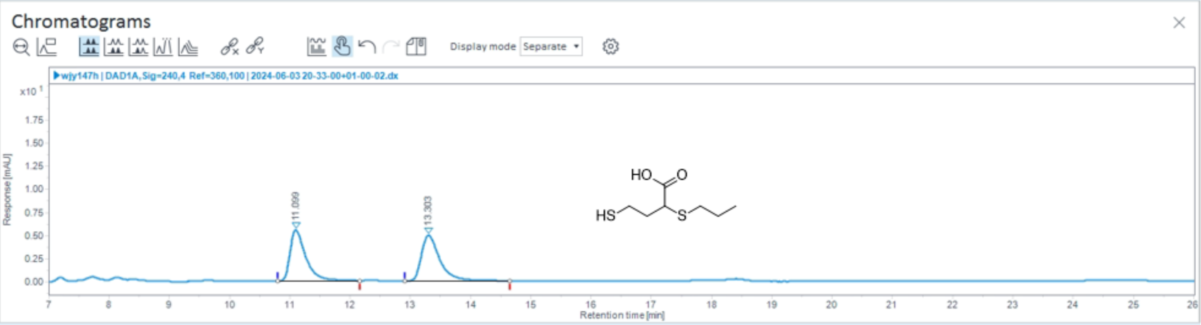

Injection Results

#	Name	Signal description	RT (min)	Area (mAU·s)	Area%	Height (mAU)	Height%	Amount	Concentration	Start time (min)	End time (min)
1		DAD1A,Sig=240,4 Ref=360,100	11.099	100.643	48.606	5.515	52.72			10.792	12.155
2		DAD1A,Sig=240,4 Ref=360,100	13.303	106.415	51.394	4.947	47.28			12.912	14.652

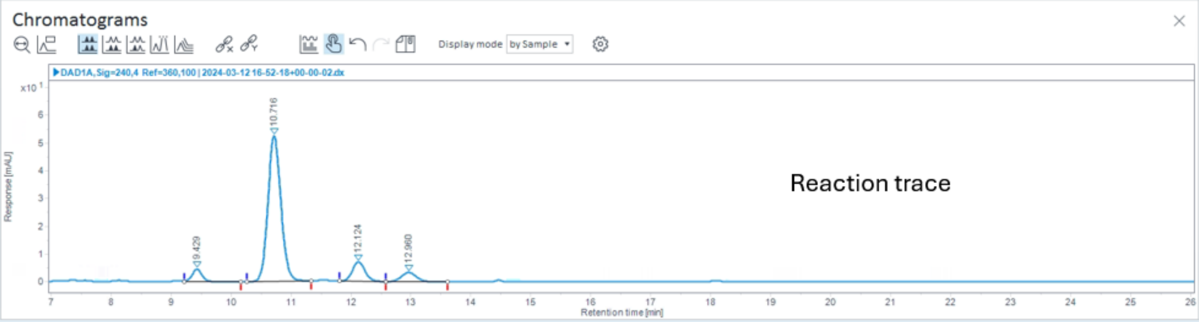

Injection Results

#	Name	Signal description	RT (min)	Area (mAU·s)	Area%	Height (mAU)	Height%	Amount	Concentration	Start time (min)	End time (min)
1		DAD1A,Sig=240,4 Ref=360,100	9.429	52.590	5.266	4.494	6.69			9.220	10.155
2		DAD1A,Sig=240,4 Ref=360,100	10.716	786.125	78.711	52.367	77.92			10.268	11.335
3		DAD1A,Sig=240,4 Ref=360,100	12.124	103.531	10.366	7.076	10.53			11.803	12.582
4		DAD1A,Sig=240,4 Ref=360,100	12.960	56.508	5.658	3.270	4.87			12.582	13.608

HPLC conditions: Chiralpak® IG column (4.6 mm × 250 mm, 5 µm), 240 nm, 10% EtOH/hexane, 1.0 mL/min.

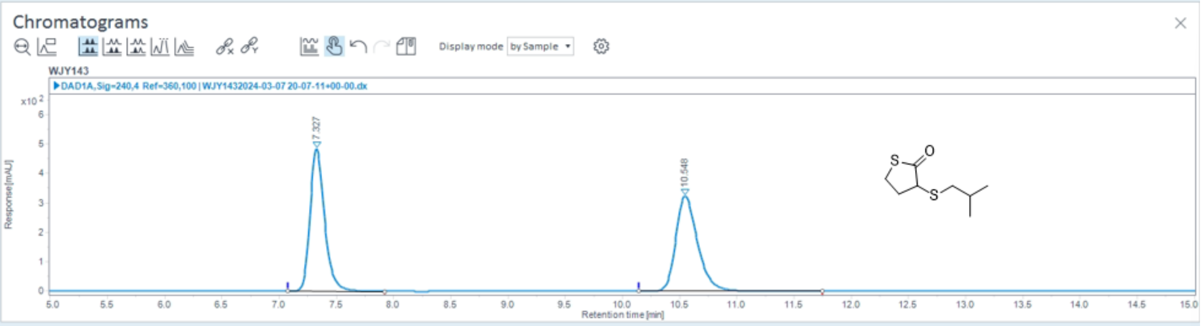

Injection Results

Peaks | Summary

#	Name	Signal description	RT (min)	Area (mAU·s)	Area%	Height (mAU)	Height%	Amount	Concentration	Start time (min)	End time (min)
1		DAD1A,Sig=240,4 Ref=360,100	7.327	4293.891	50.122	483.926	59.97			7.075	7.924
2		DAD1A,Sig=240,4 Ref=360,100	10.548	4273.026	49.878	323.072	40.03			10.142	11.749

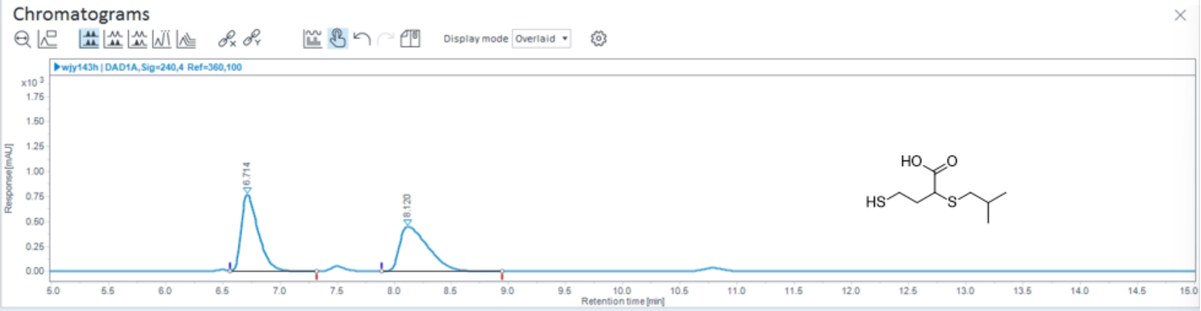

Injection Results

Peaks | Summary

#	Name	Signal description	RT (min)	Area (mAU·s)	Area%	Height (mAU)	Height%	Amount	Concentration	Start time (min)	End time (min)
1		DAD1A,Sig=240,4 Ref=360,100	6.714	7976.294	50.620	768.960	63.15			6.566	7.318
2		DAD1A,Sig=240,4 Ref=360,100	8.120	7780.879	49.379	448.649	36.85			7.889	8.941

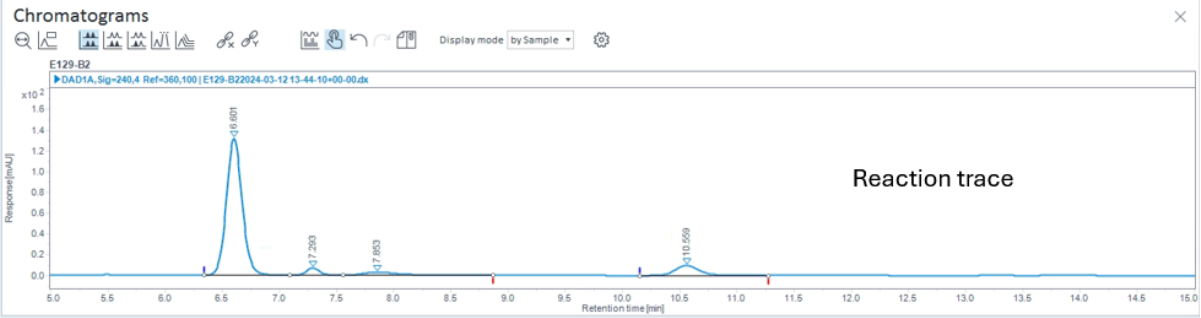

Injection Results

Peaks | Summary

#	Name	Signal description	RT (min)	Area (mAU·s)	Area%	Height (mAU)	Height%	Amount	Concentration	Start time (min)	End time (min)
1		DAD1A,Sig=240,4 Ref=360,100	6.601	1277.939	82.464	131.735	86.83			6.337	7.090
2		DAD1A,Sig=240,4 Ref=360,100	7.293	60.702	3.917	7.213	4.75			7.090	7.558
3		DAD1A,Sig=240,4 Ref=360,100	7.853	69.453	4.482	3.162	2.08			7.558	8.870
4		DAD1A,Sig=240,4 Ref=360,100	10.559	141.599	9.137	9.606	6.33			10.150	11.274

HPLC conditions: Chiralpak® IG column (4.6 mm × 250 mm, 5 μm), 240 nm, 10% EtOH/hexane, 1.0 mL/min.

Chromatograms

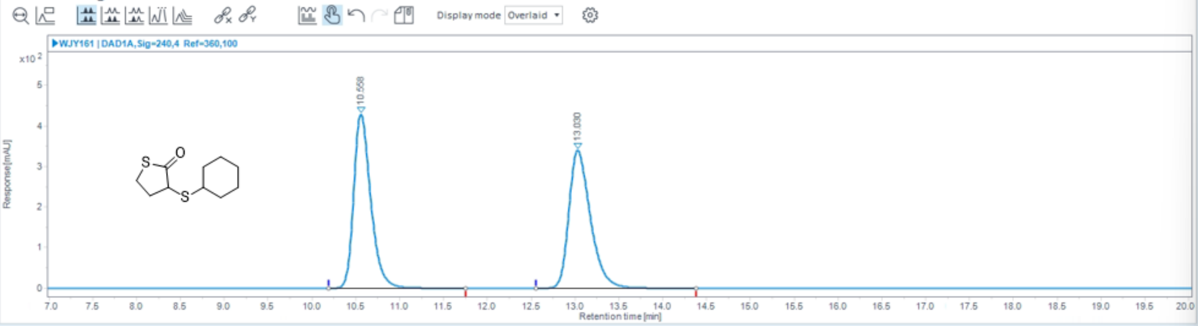

Injection Results

#	Name	Signal description	RT (min)	Area (mAU·s)	Area%	Height (mAU)	Height%	Amount	Concentration	Start time (min)	End time (min)
1		DAD1A,Sig=240,4 Ref=360,100	10.558	5794.973	49.985	428.895	55.78			10.189	11.749
2		DAD1A,Sig=240,4 Ref=360,100	13.030	5798.562	50.015	339.947	44.22			12.549	14.376

Chromatograms

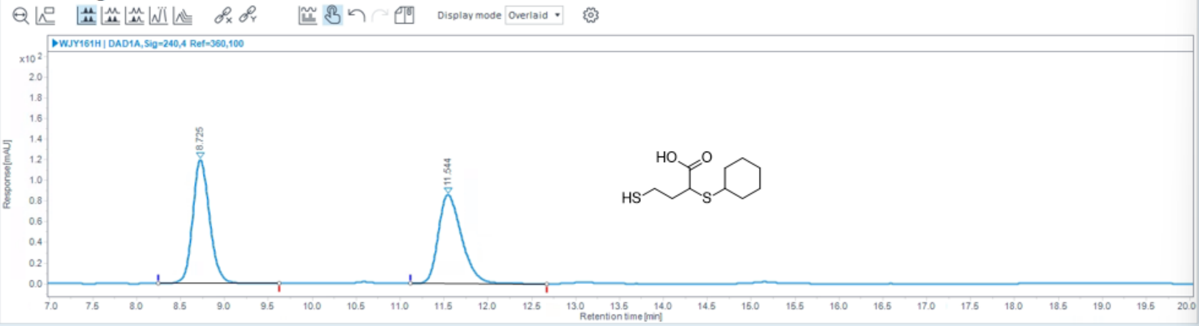

Injection Results

#	Name	Signal description	RT (min)	Area (mAU·s)	Area%	Height (mAU)	Height%	Amount	Concentration	Start time (min)	End time (min)
1		DAD1A,Sig=240,4 Ref=360,100	8.725	1619.381	50.408	119.956	58.16			8.248	9.626
2		DAD1A,Sig=240,4 Ref=360,100	11.544	1593.157	49.592	86.301	41.84			11.117	12.667

Chromatograms

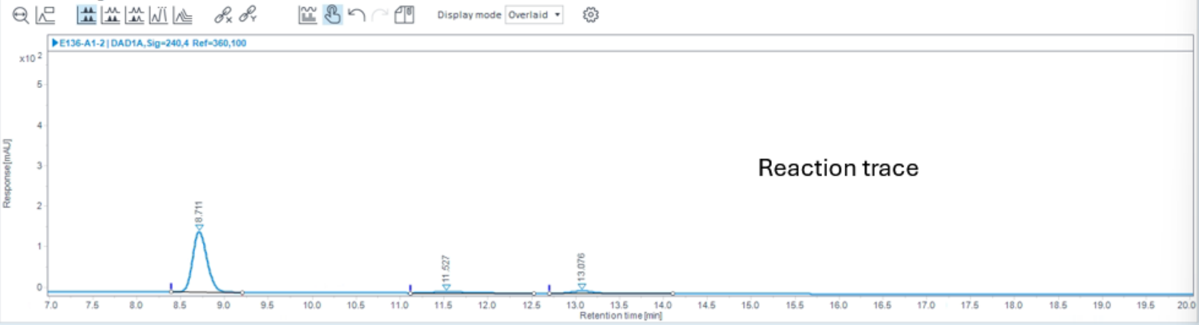

Injection Results

#	Name	Signal description	RT (min)	Area (mAU·s)	Area%	Height (mAU)	Height%	Amount	Concentration	Start time (min)	End time (min)
1		DAD1A,Sig=240,4 Ref=360,100	8.711	1723.199	88.506	149.387	93.45			8.394	9.199
2		DAD1A,Sig=240,4 Ref=360,100	11.527	115.268	5.920	4.163	2.60			11.116	12.522
3		DAD1A,Sig=240,4 Ref=360,100	13.076	108.512	5.573	6.316	3.95			12.696	14.109

HPLC conditions: Chiralpak® IG column (4.6 mm × 250 mm, 5 μm), 240 nm, 10% EtOH/hexane, 1.0 mL/min.

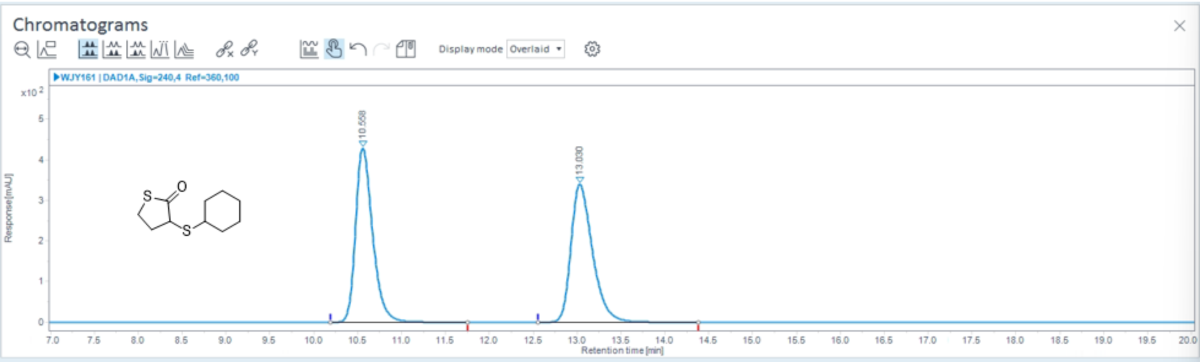

Injection Results

#	Name	Signal description	RT (min)	Area (mAU·s)	Area%	Height (mAU)	Height%	Amount	Concentration	Start time (min)	End time (min)
1		DAD1A,Sig=240,4 Ref=360,100	10.558	5794.973	49.985	428.895	55.78			10.189	11.749
2		DAD1A,Sig=240,4 Ref=360,100	13.030	5798.562	50.015	339.947	44.22			12.549	14.376

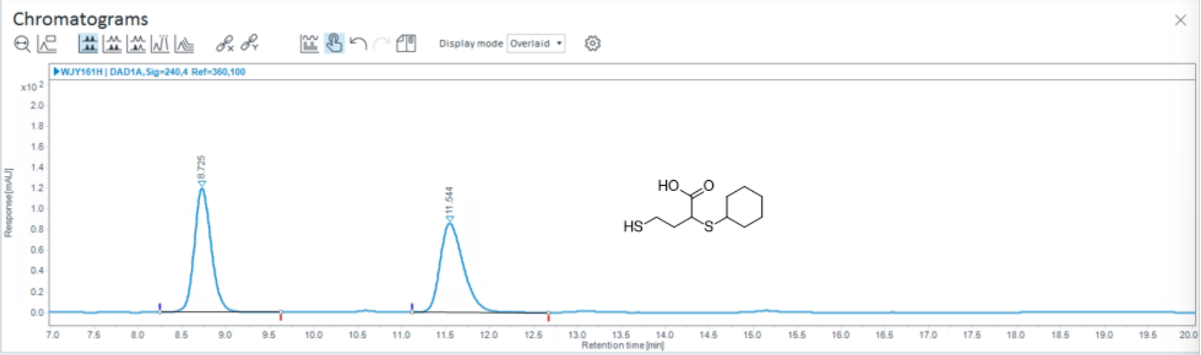

Injection Results

#	Name	Signal description	RT (min)	Area (mAU·s)	Area%	Height (mAU)	Height%	Amount	Concentration	Start time (min)	End time (min)
1		DAD1A,Sig=240,4 Ref=360,100	8.725	1619.381	50.408	119.956	58.16			8.248	9.626
2		DAD1A,Sig=240,4 Ref=360,100	11.544	1593.157	49.592	86.301	41.84			11.117	12.667

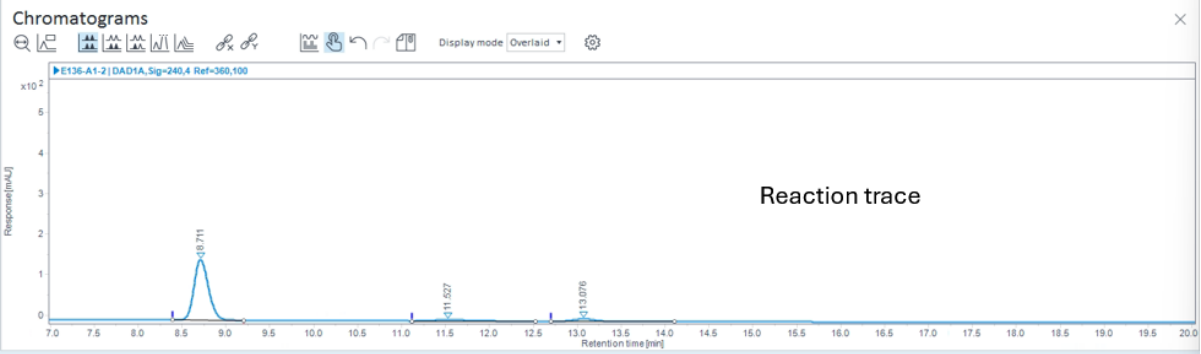

Injection Results

#	Name	Signal description	RT (min)	Area (mAU·s)	Area%	Height (mAU)	Height%	Amount	Concentration	Start time (min)	End time (min)
1		DAD1A,Sig=240,4 Ref=360,100	8.711	1723.199	88.506	149.387	93.45			8.394	9.199
2		DAD1A,Sig=240,4 Ref=360,100	11.527	115.268	5.920	4.163	2.60			11.116	12.522
3		DAD1A,Sig=240,4 Ref=360,100	13.076	108.512	5.573	6.316	3.95			12.696	14.109

HPLC conditions: Chiralpak® IG column (4.6 mm × 250 mm, 5 µm), 240 nm, 10% EtOH/hexane, 1.0 mL/min.

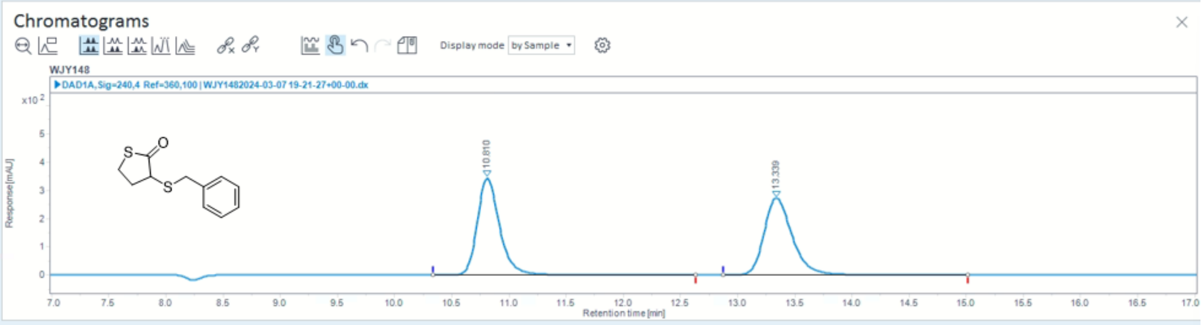

Injection Results

#	Name	Signal description	RT (min)	Area (mAU·s)	Area%	Height (mAU)	Height%	Amount	Concentration	Start time (min)	End time (min)
1		DAD1A,Sig=240,4 Ref=360,100	10.810	4671.405	49.966	343.164	55.71			10.339	12.632
2		DAD1A,Sig=240,4 Ref=360,100	13.339	4677.690	50.034	272.862	44.29			12.872	15.012

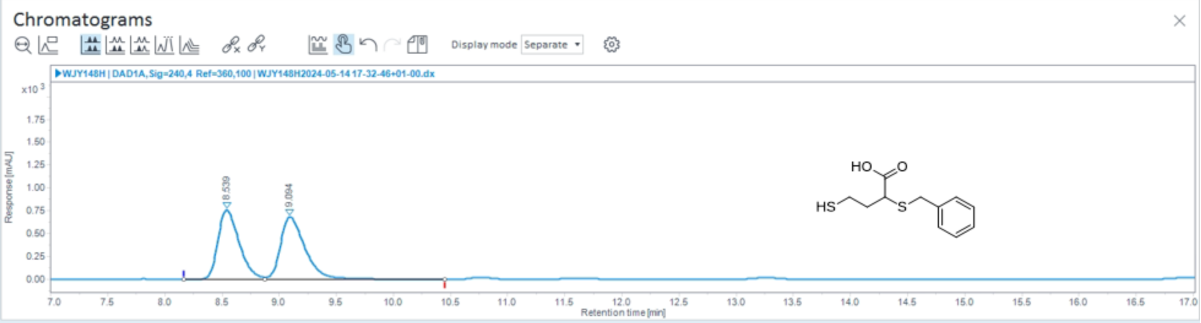

Injection Results

#	Name	Signal description	RT (min)	Area (mAU·s)	Area%	Height (mAU)	Height%	Amount	Concentration	Start time (min)	End time (min)
1		DAD1A,Sig=240,4 Ref=360,100	8.539	9806.854	48.775	759.659	52.66			8.166	8.876
2		DAD1A,Sig=240,4 Ref=360,100	9.094	10299.580	51.225	682.811	47.34			8.876	10.445

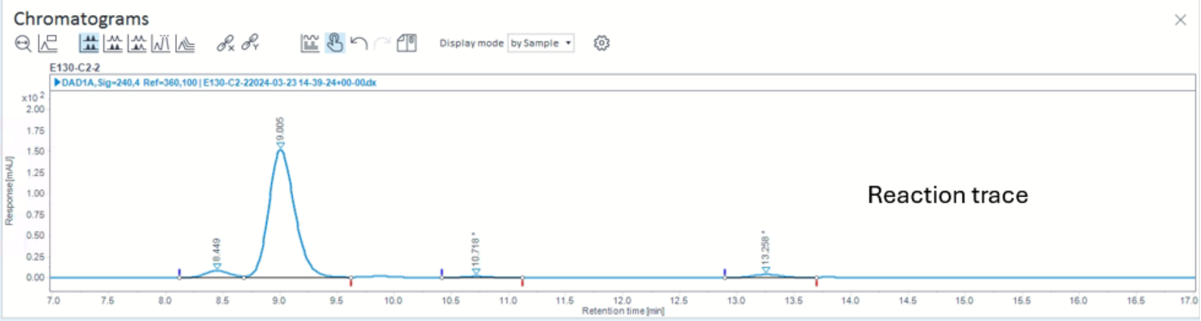

Injection Results

#	Name	Signal description	RT (min)	Area (mAU·s)	Area%	Height (mAU)	Height%	Amount	Concentration	Start time (min)	End time (min)
1		DAD1A,Sig=240,4 Ref=360,100	8.449	127.761	4.859	8.393	5.06			8.123	8.685
2		DAD1A,Sig=240,4 Ref=360,100	9.005	2415.772	91.875	151.988	91.69			8.685	9.619
3		DAD1A,Sig=240,4 Ref=360,100	10.718	25.783	0.981	1.706	1.03			10.420	11.124
4		DAD1A,Sig=240,4 Ref=360,100	13.258	60.099	2.286	3.669	2.21			12.893	13.702

HPLC conditions: Chiralpak® IG column (4.6 mm × 250 mm, 5 μm), 240 nm, 8% EtOH/hexane, 1.0 mL/min.

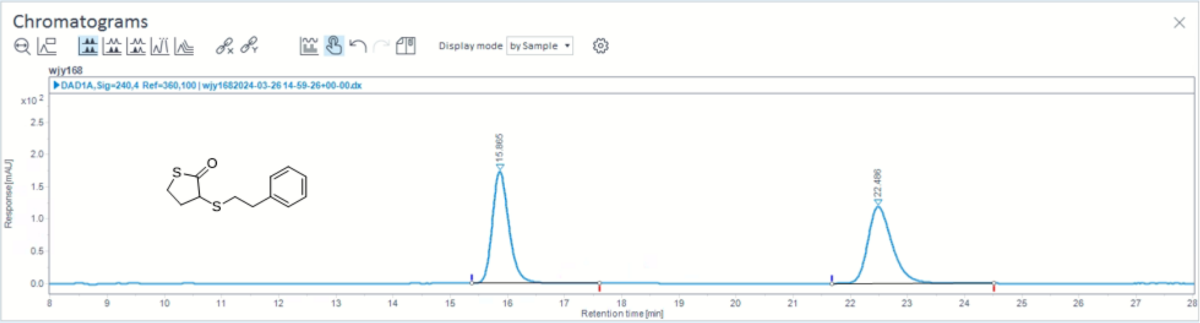

Injection Results

#	Name	Signal description	RT (min)	Area (mAU·s)	Area%	Height (mAU)	Height%	Amount	Concentration	Start time (min)	End time (min)
1		DAD1A,Sig=240,4 Ref=360,100	15.865	3531.581	50.081	174.264	59.38			15.383	17.607
2		DAD1A,Sig=240,4 Ref=360,100	22.486	3520.128	49.919	119.228	40.62			21.677	24.510

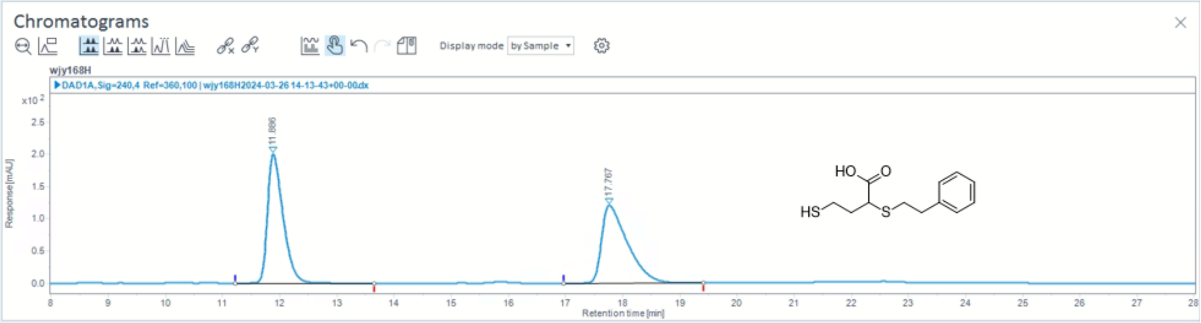

Injection Results

#	Name	Signal description	RT (min)	Area (mAU·s)	Area%	Height (mAU)	Height%	Amount	Concentration	Start time (min)	End time (min)
1		DAD1A,Sig=240,4 Ref=360,100	11.886	3785.717	49.936	200.371	62.41			11.225	13.652
2		DAD1A,Sig=240,4 Ref=360,100	17.767	3795.386	50.064	120.660	37.59			16.965	19.405

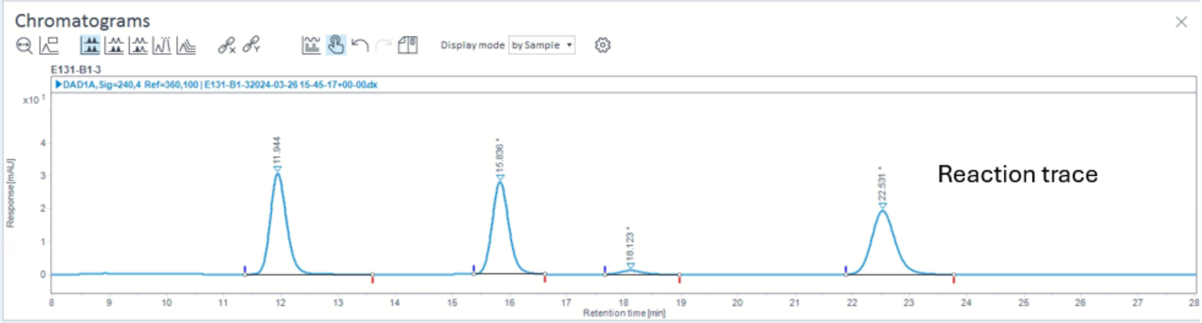

Injection Results

#	Name	Signal description	RT (min)	Area (mAU·s)	Area%	Height (mAU)	Height%	Amount	Concentration	Start time (min)	End time (min)
1		DAD1A,Sig=240,4 Ref=360,100	11.944	613.435	34.489	30.685	38.65			11.368	13.608
2		DAD1A,Sig=240,4 Ref=360,100	15.836	572.834	32.207	28.117	35.41			15.371	16.616
3		DAD1A,Sig=240,4 Ref=360,100	18.123	31.900	1.794	1.166	1.47			17.677	18.979
4		DAD1A,Sig=240,4 Ref=360,100	22.531	560.455	31.511	19.429	24.47			21.881	23.778

HPLC conditions: Chiralpak® IG column (4.6 mm × 250 mm, 5 µm), 240 nm, 10% EtOH/hexane, 1.0 mL/min.

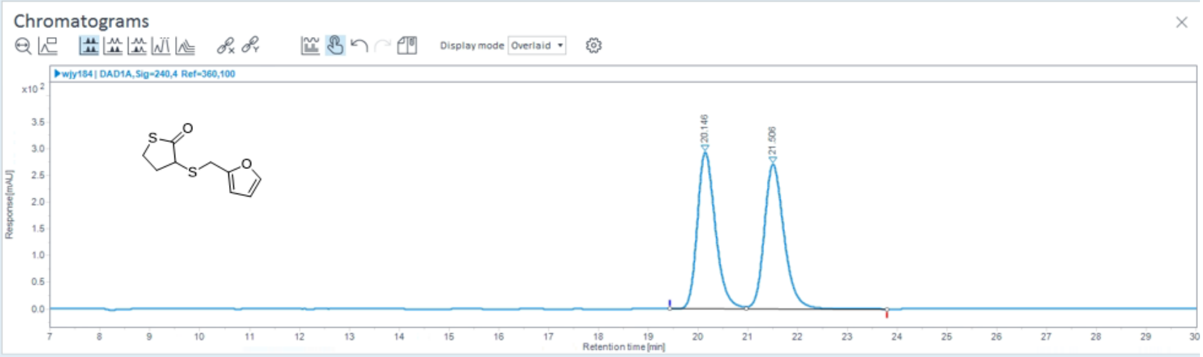

Injection Results

#	Name	Signal description	RT (min)	Area (mAU·s)	Area%	Height (mAU)	Height%	Amount	Concentration	Start time (min)	End time (min)
1		DAD1A,Sig=240,4 Ref=360,100	20.146	7432.088	49.775	293.761	52.02			19.435	20.961
2		DAD1A,Sig=240,4 Ref=360,100	21.506	7499.255	50.225	270.973	47.98			20.961	23.802

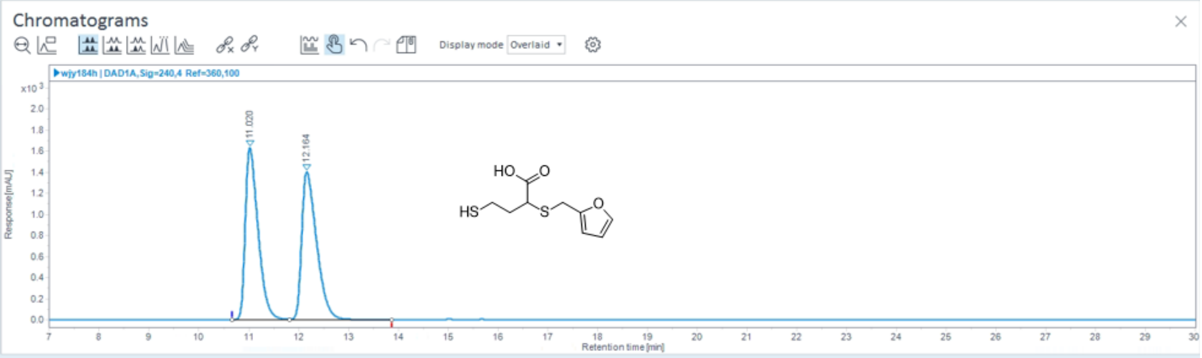

Injection Results

#	Name	Signal description	RT (min)	Area (mAU·s)	Area%	Height (mAU)	Height%	Amount	Concentration	Start time (min)	End time (min)
1		DAD1A,Sig=240,4 Ref=360,100	11.020	29127.965	49.820	1629.130	53.74			10.670	11.812
2		DAD1A,Sig=240,4 Ref=360,100	12.164	29338.083	50.180	1402.153	46.26			11.812	13.876

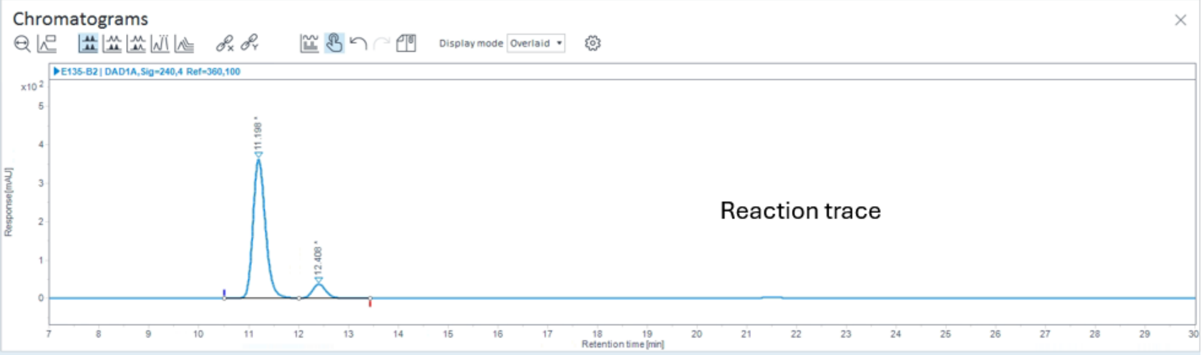

Injection Results

#	Name	Signal description	RT (min)	Area (mAU·s)	Area%	Height (mAU)	Height%	Amount	Concentration	Start time (min)	End time (min)
1		DAD1A,Sig=240,4 Ref=360,100	11.198	6294.083	89.885	361.936	90.71			10.503	12.008
2		DAD1A,Sig=240,4 Ref=360,100	12.408	708.295	10.115	37.051	9.29			12.008	13.441

6.3. HPLC analysis for enzymatic reactions catalysed by N9

HPLC conditions: Chiralpak® IG column (4.6 mm × 250 mm, 5 µm), 240 nm, 10% EtOH/hexane, 1.0 mL/min.

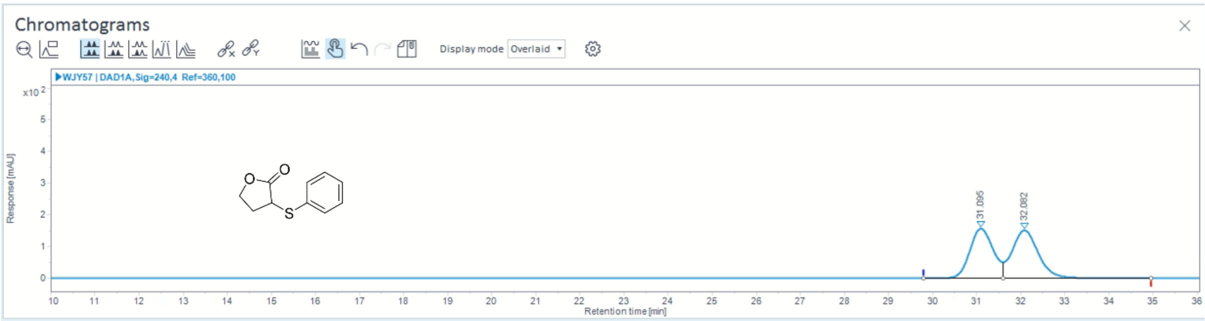

Injection Results

Peaks Summary

#	Name	Signal description	RT (min)	Area (mAU·s)	Area%	Height (mAU)	Height%	Amount	Concentration	Start time (min)	End time (min)
1		DAD1A,Sig=240,4 Ref=360,100	31.095	5782.200	47.932	156.706	50.87			29.785	31.600
2		DAD1A,Sig=240,4 Ref=360,100	32.082	6281.260	52.068	151.350	49.13			31.600	34.962

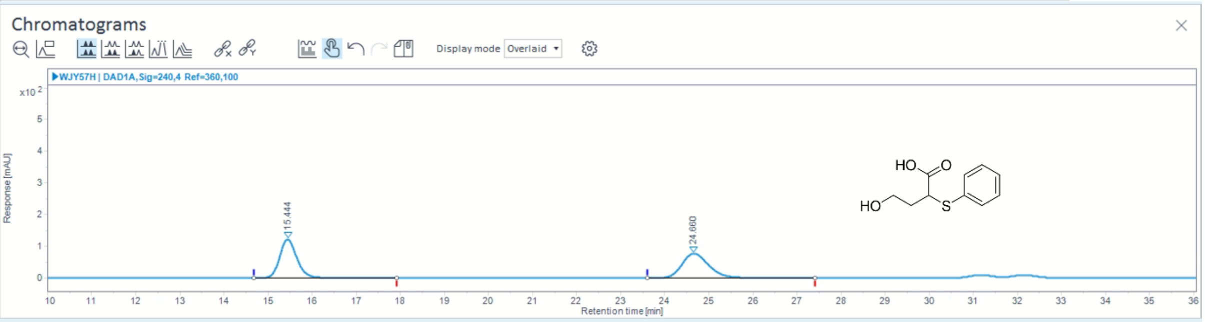

Injection Results

Peaks Summary

#	Name	Signal description	RT (min)	Area (mAU·s)	Area%	Height (mAU)	Height%	Amount	Concentration	Start time (min)	End time (min)
1		DAD1A,Sig=240,4 Ref=360,100	15.444	3318.743	50.181	121.635	61.19			14.685	17.912
2		DAD1A,Sig=240,4 Ref=360,100	24.660	3294.750	49.819	77.161	38.81			23.605	27.404

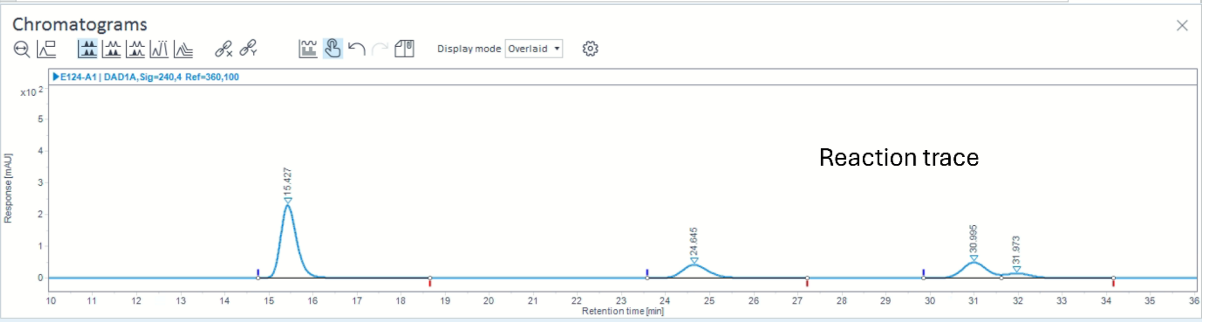

Injection Results

Peaks Summary

#	Name	Signal description	RT (min)	Area (mAU·s)	Area%	Height (mAU)	Height%	Amount	Concentration	Start time (min)	End time (min)
1		DAD1A,Sig=240,4 Ref=360,100	15.427	5896.168	57.835	229.587	68.36			14.749	18.656
2		DAD1A,Sig=240,4 Ref=360,100	24.645	1741.503	17.082	41.618	12.39			23.576	27.216
3		DAD1A,Sig=240,4 Ref=360,100	30.995	1948.094	19.109	49.534	14.75			29.849	31.614
4		DAD1A,Sig=240,4 Ref=360,100	31.973	609.119	5.975	15.118	4.50			31.614	34.156

HPLC conditions: Chiralpak® IG column (4.6 mm × 250 mm, 5 μm), 240 nm, 10% EtOH/hexane, 1.0 mL/min.

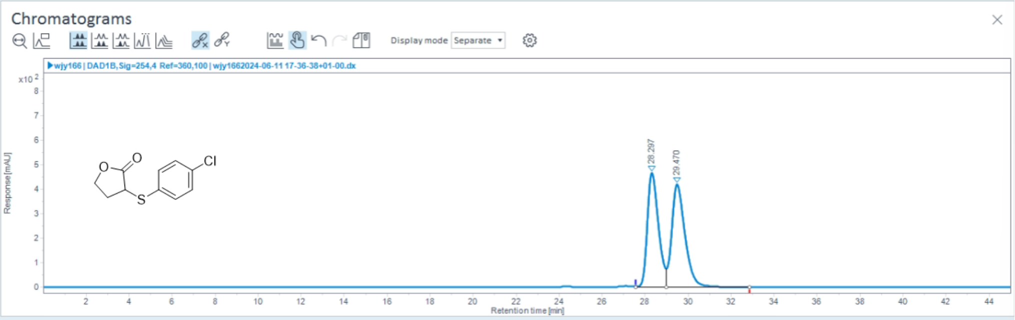

Injection Results

#	Name	Signal description	RT (min)	Area (mAU·s)	Area%	Height (mAU)	Height%	Amount	Concentration	Start time (min)	End time (min)
1		DAD1B,Sig=254,4 Ref=360,100	28.297	16716.030	48.240	467.598	52.67			27.545	28.969
2		DAD1B,Sig=254,4 Ref=360,100	29.470	17936.046	51.760	420.186	47.33			28.969	32.874

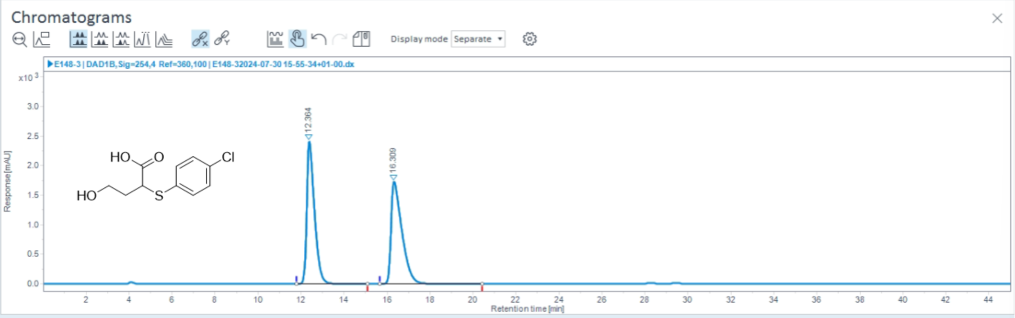

Injection Results

#	Name	Signal description	RT (min)	Area (mAU·s)	Area%	Height (mAU)	Height%	Amount	Concentration	Start time (min)	End time (min)
1		DAD1B,Sig=254,4 Ref=360,100	12.364	59722.159	49.622	2416.418	58.16			11.793	15.082
2		DAD1B,Sig=254,4 Ref=360,100	16.309	60632.471	50.378	1738.678	41.84			15.646	20.413

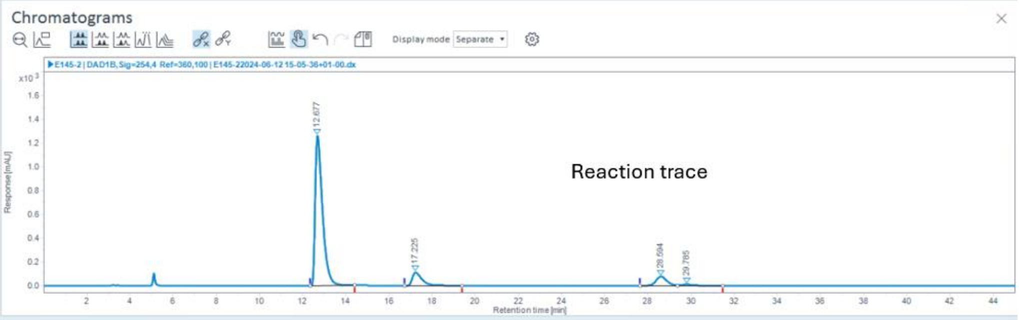

Injection Results

#	Name	Signal description	RT (min)	Area (mAU·s)	Area%	Height (mAU)	Height%	Amount	Concentration	Start time (min)	End time (min)
1		DAD1B,Sig=254,4 Ref=360,100	12.677	29667.792	81.945	1266.167	86.52			12.336	14.413
2		DAD1B,Sig=254,4 Ref=360,100	17.225	3440.890	9.504	111.291	7.61			16.723	19.400
3		DAD1B,Sig=254,4 Ref=360,100	28.594	2738.879	7.565	76.762	5.25			27.650	29.380
4		DAD1B,Sig=254,4 Ref=360,100	29.785	356.785	0.985	9.146	0.63			29.380	31.476

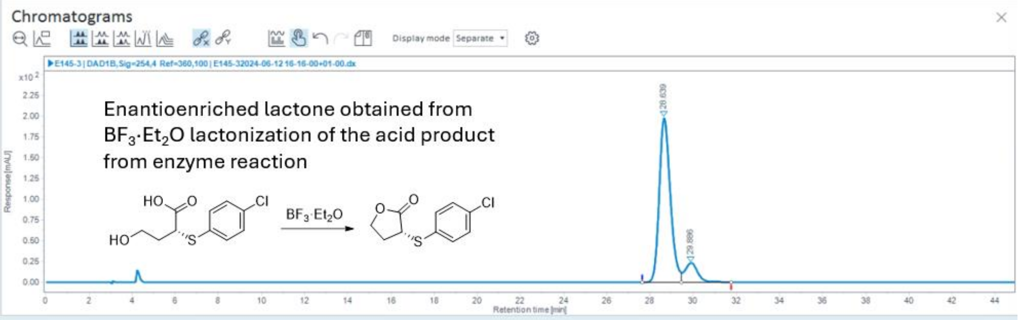

Injection Results

#	Name	Signal description	RT (min)	Area (mAU·s)	Area%	Height (mAU)	Height%	Amount	Concentration	Start time (min)	End time (min)
1		DAD1B,Sig=254,4 Ref=360,100	28.639	7077.797	88.334	197.944	89.47			27.646	29.464
2		DAD1B,Sig=254,4 Ref=360,100	29.886	934.752	11.666	23.304	10.53			29.464	31.760

Lactone 8b equilibrium monitoring monitored by reversed-phase HPLC

HPLC conditions: Agilent Eclipse Plus C18 column (4.6 mm × 150 mm, 5 µm), 240 nm, 1.0 mL/min, water and acetonitrile were used as mobile phase and the gradient is shown below.

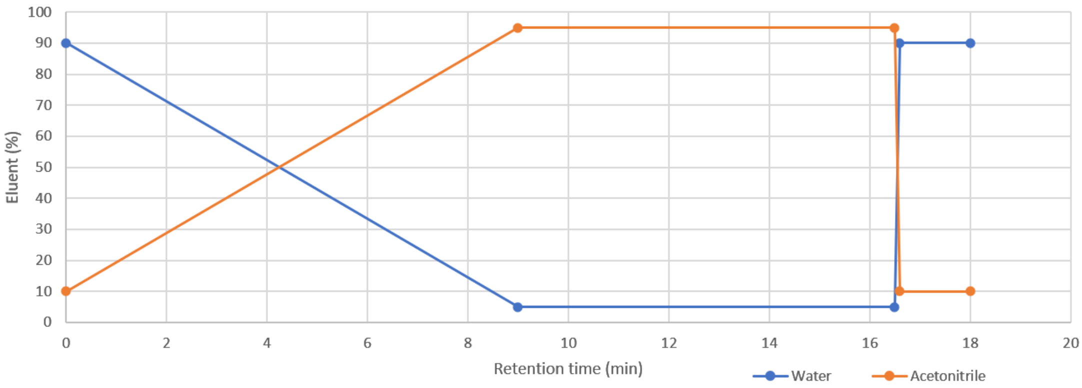

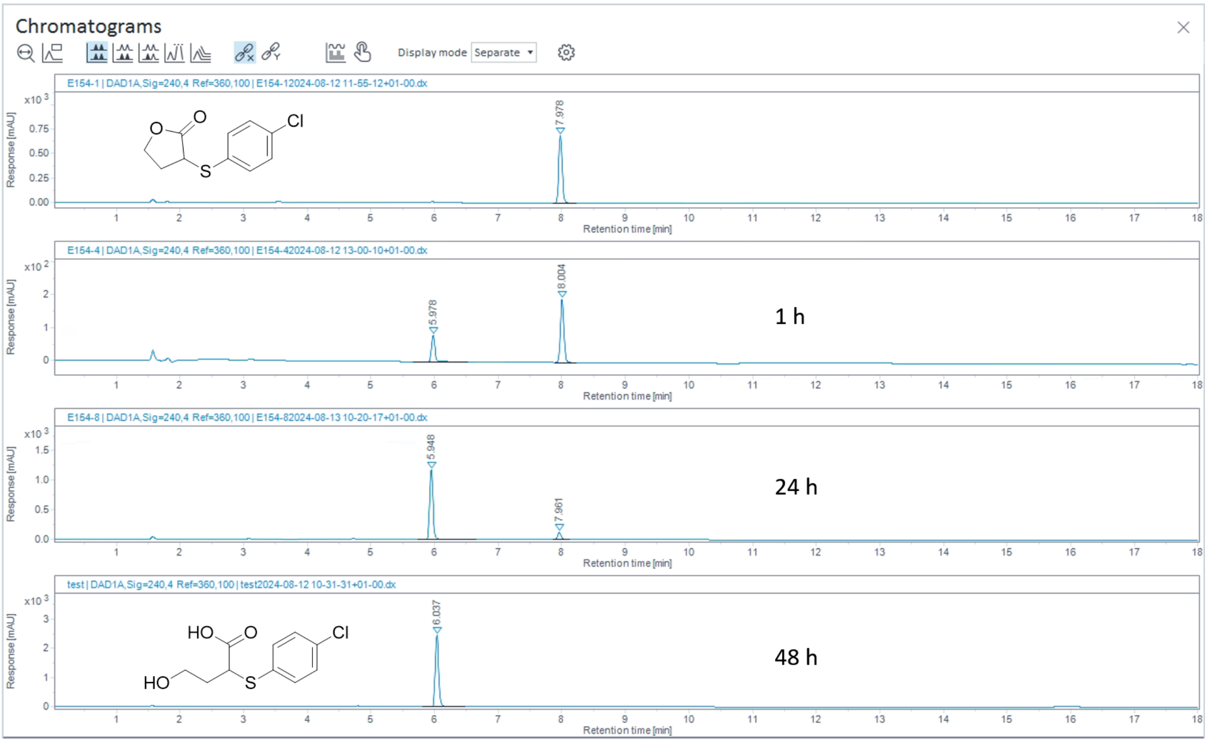

7. Copies of NMR spectra

N-(2-Oxotetrahydrothiophen-3-yl)hexanamide 1a

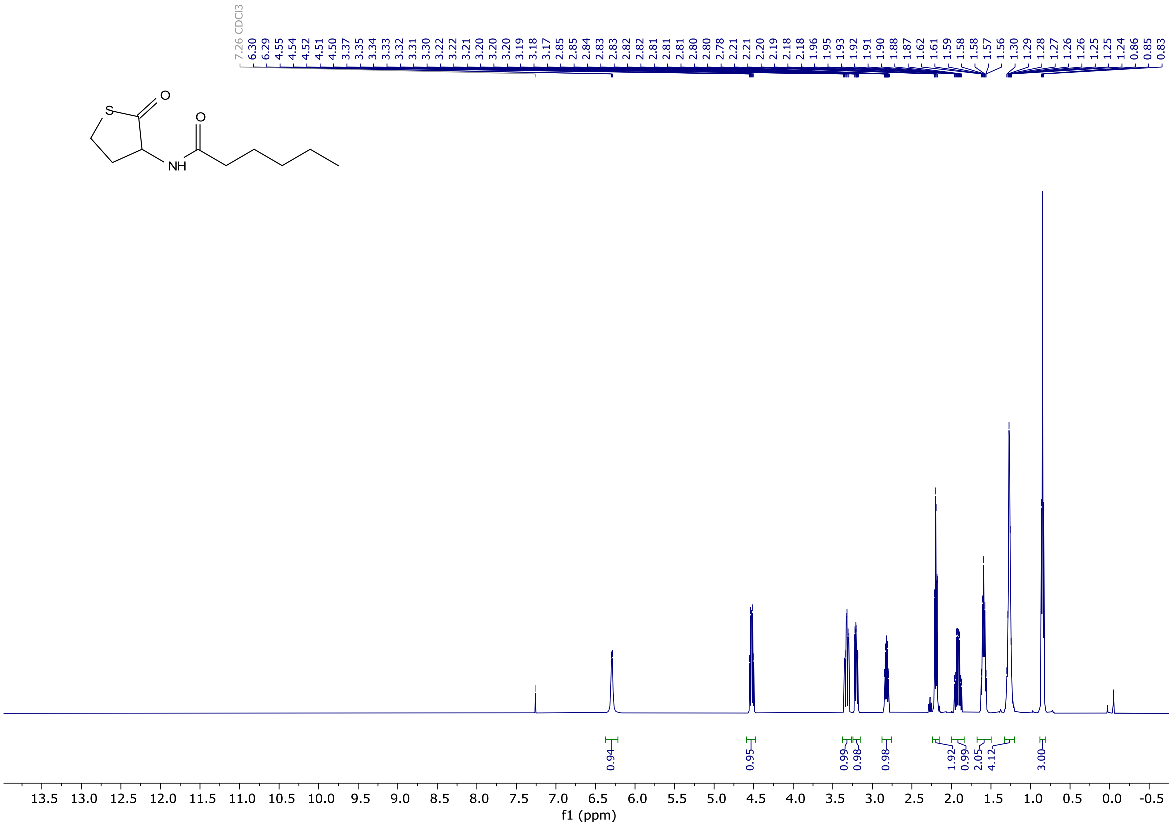

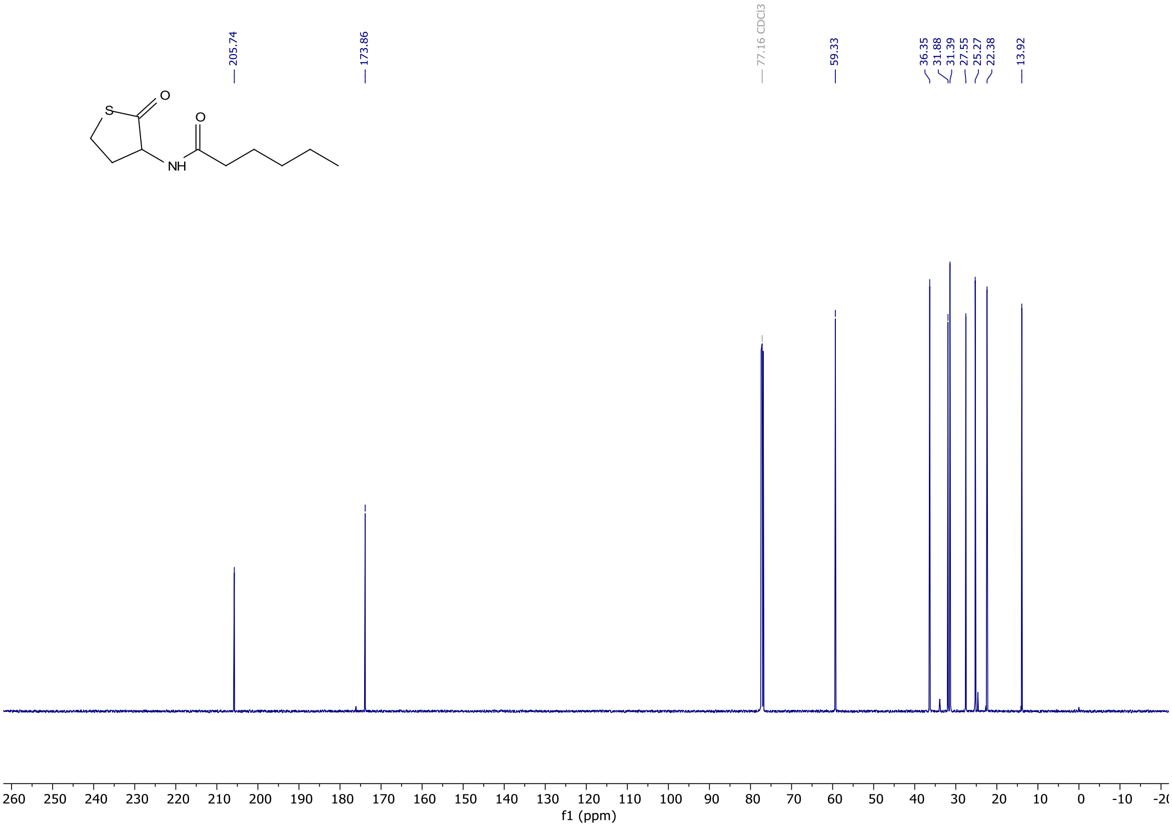

N-(2-Oxotetrahydrothiophen-3-yl)benzamide 1b

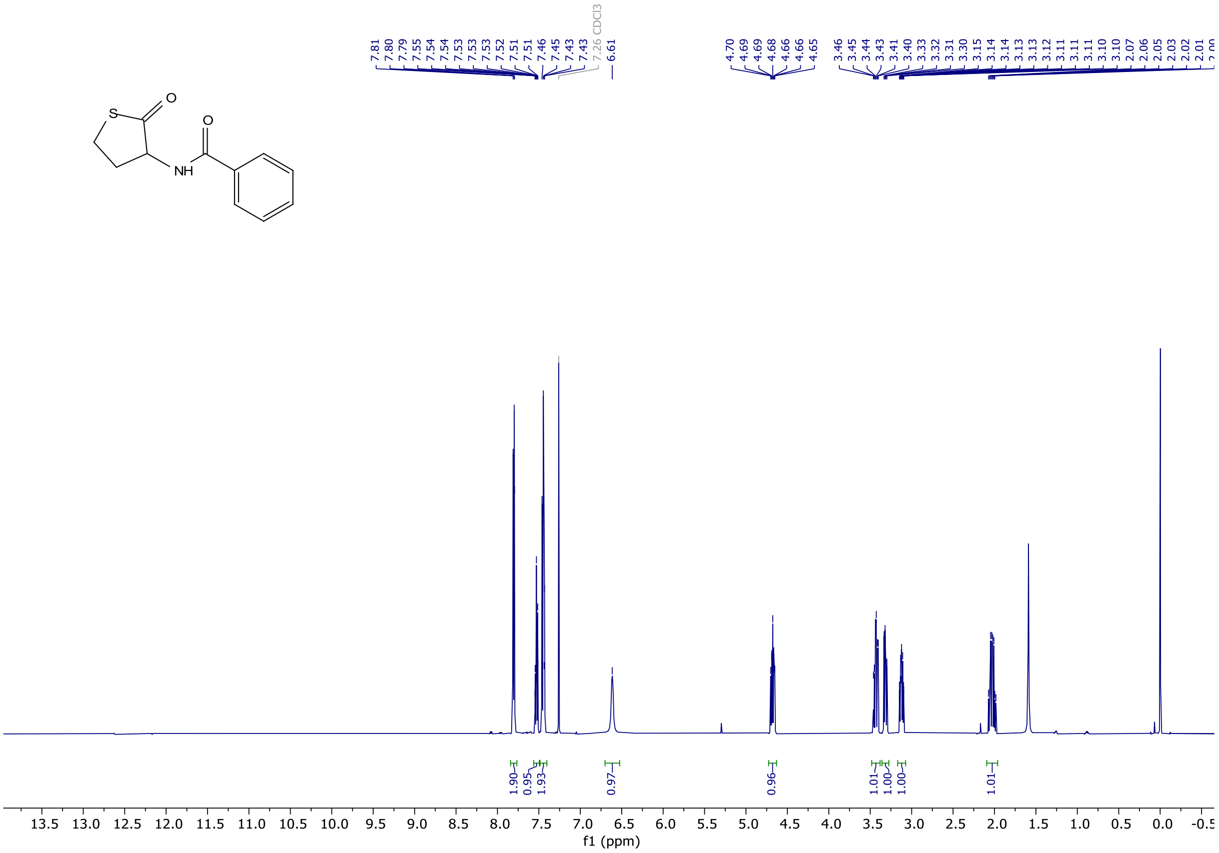

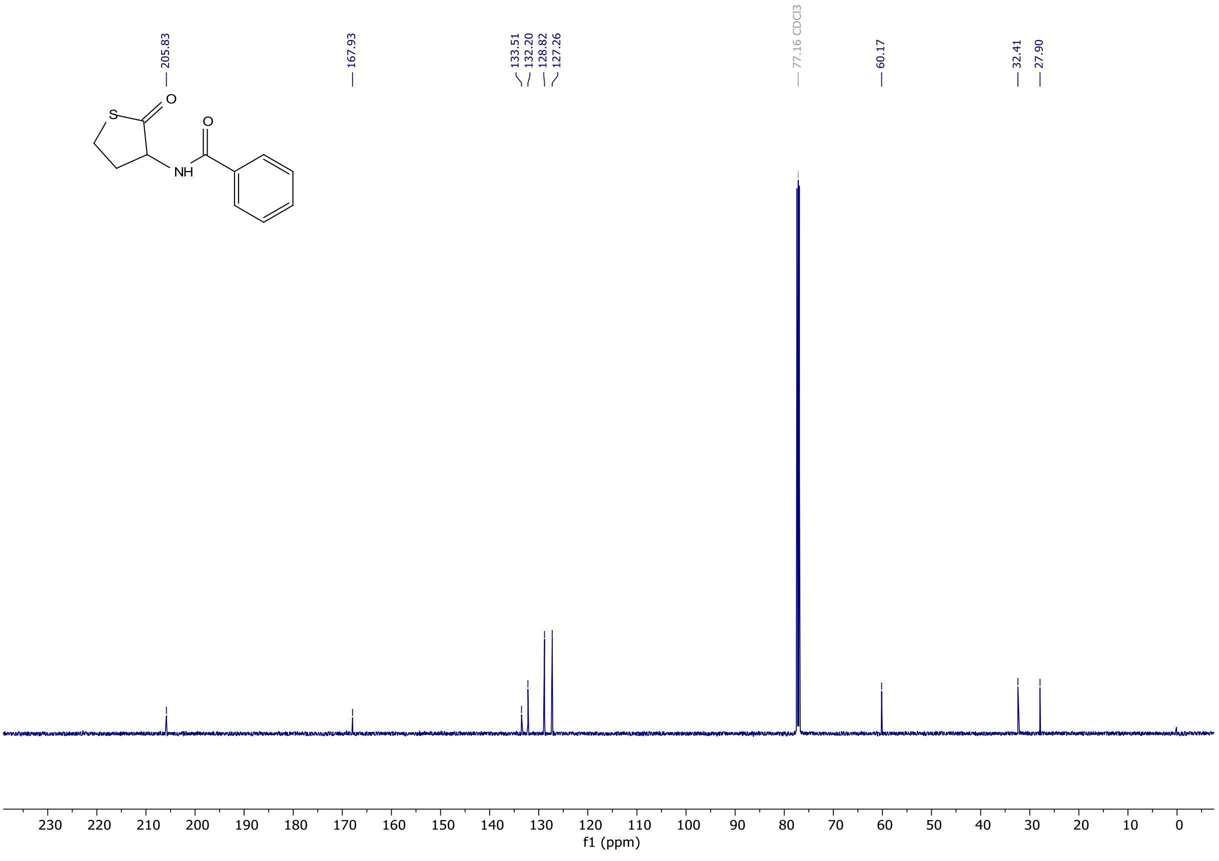

N-(2-Oxotetrahydrothiophen-3-yl)-2-phenylacetamide 1c

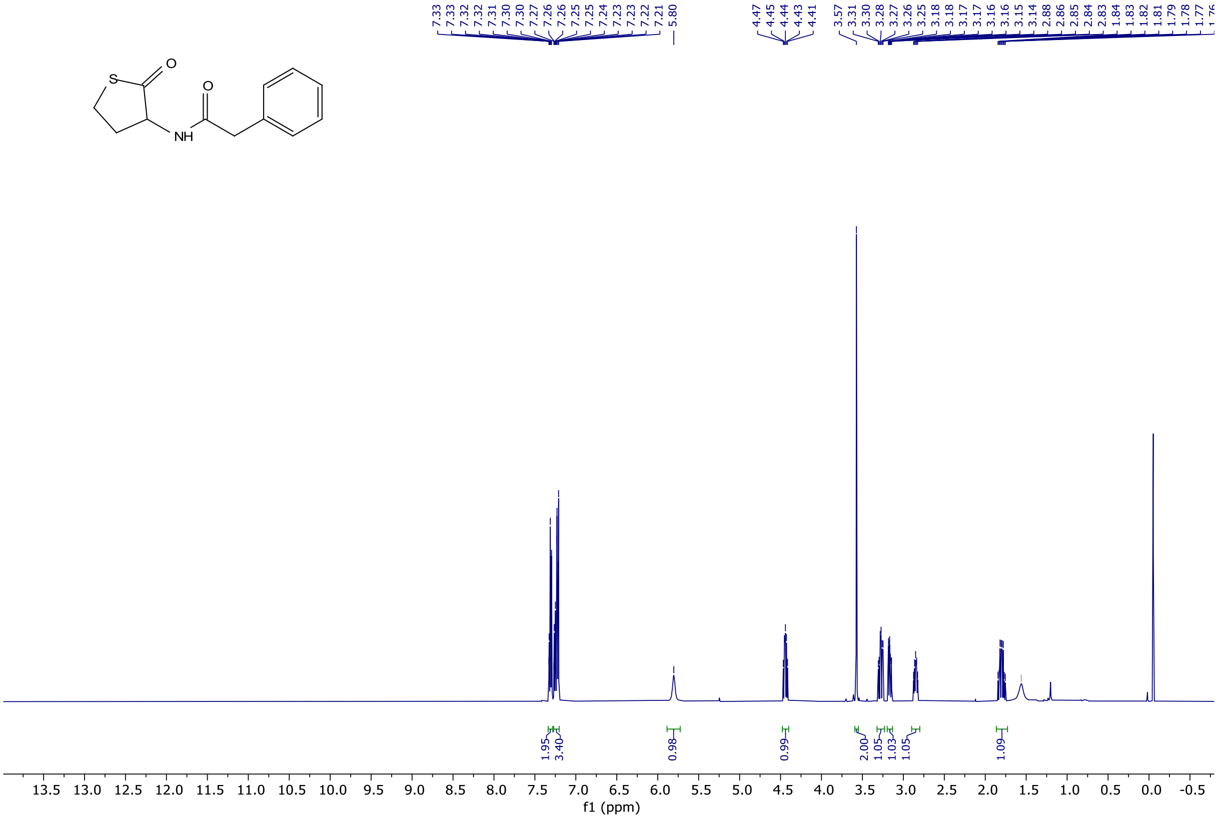

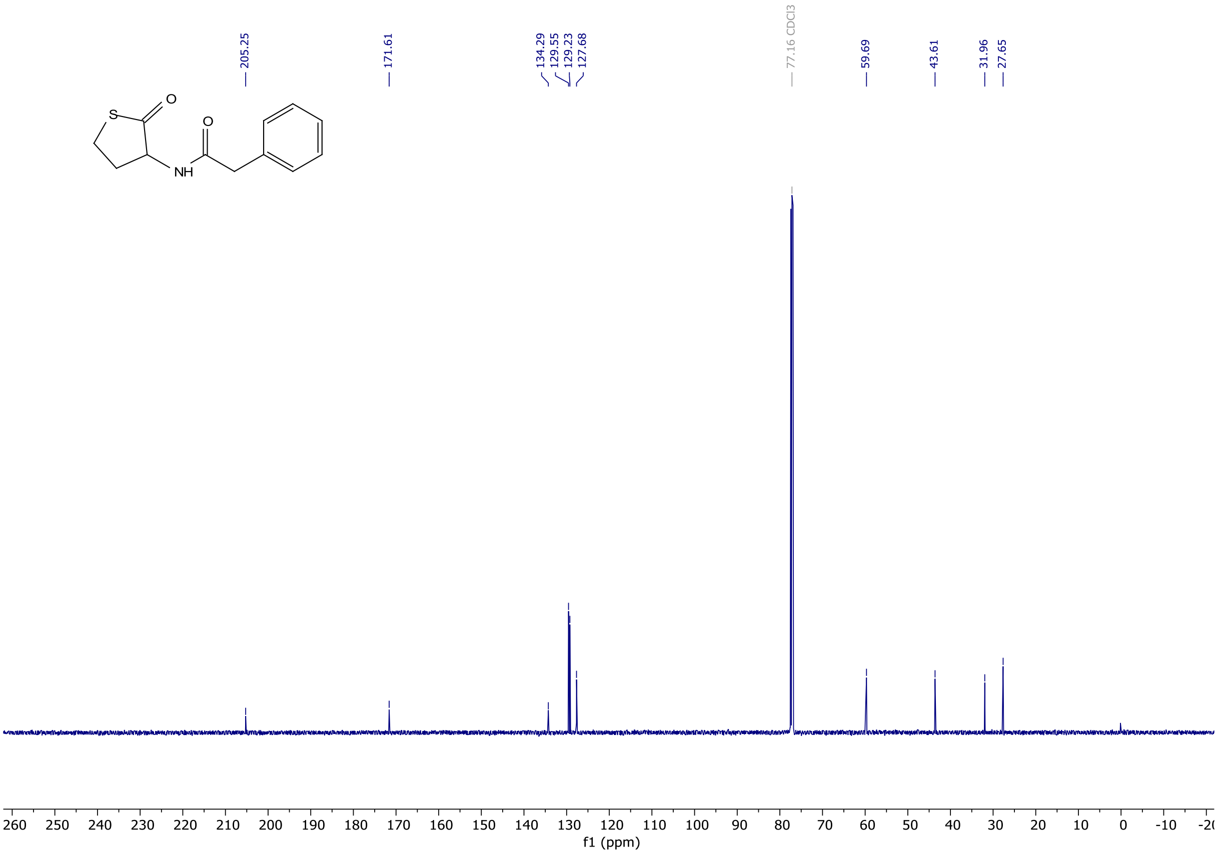

N-(2-Oxotetrahydrothiophen-3-yl)isobutyramide 1d

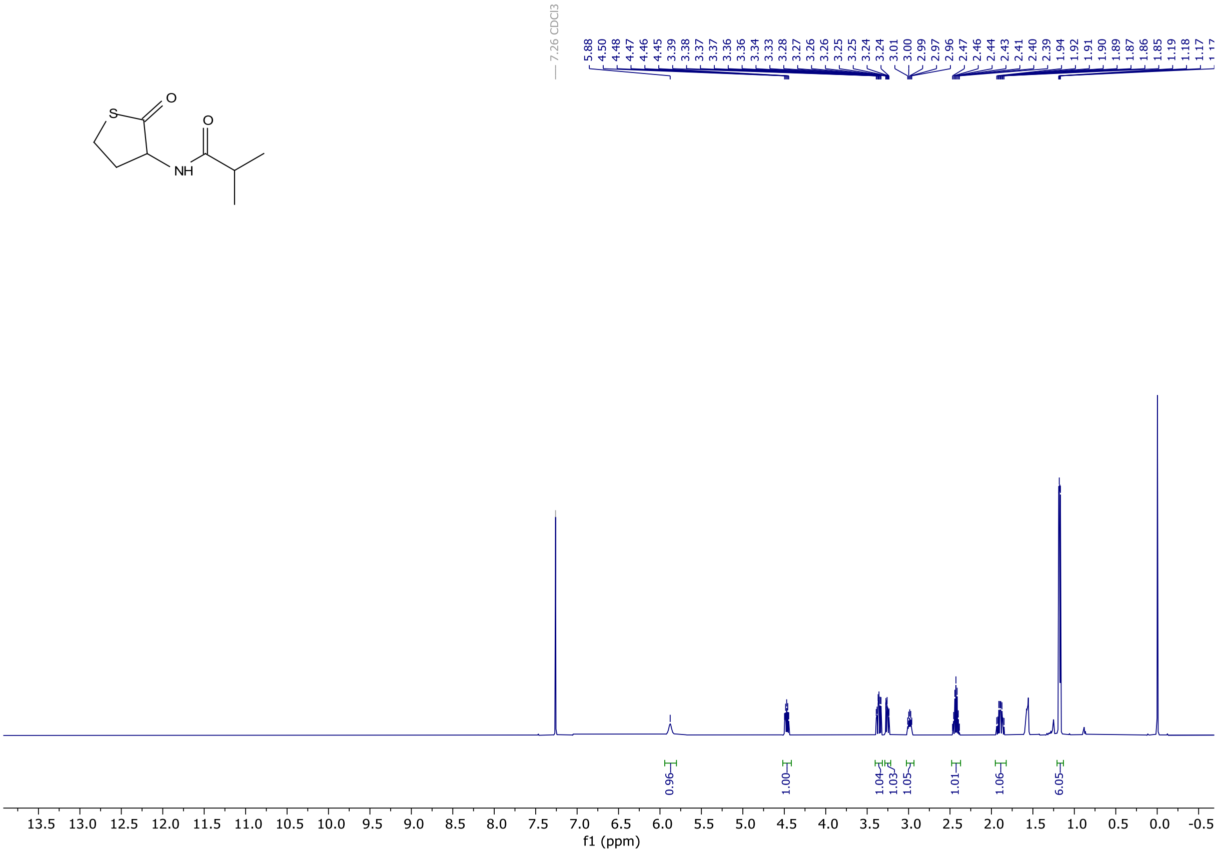

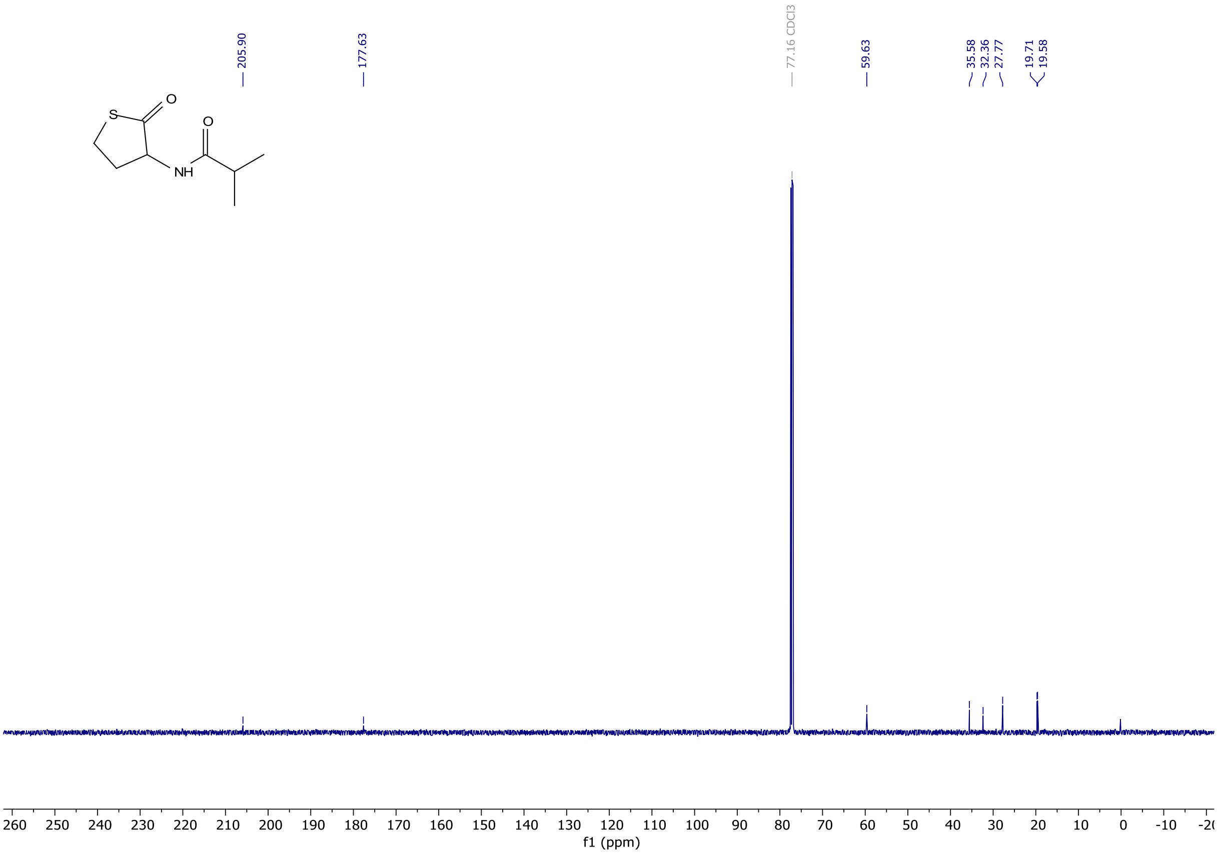

N-(2-Oxotetrahydrothiophen-3-yl)pivalamide 1e

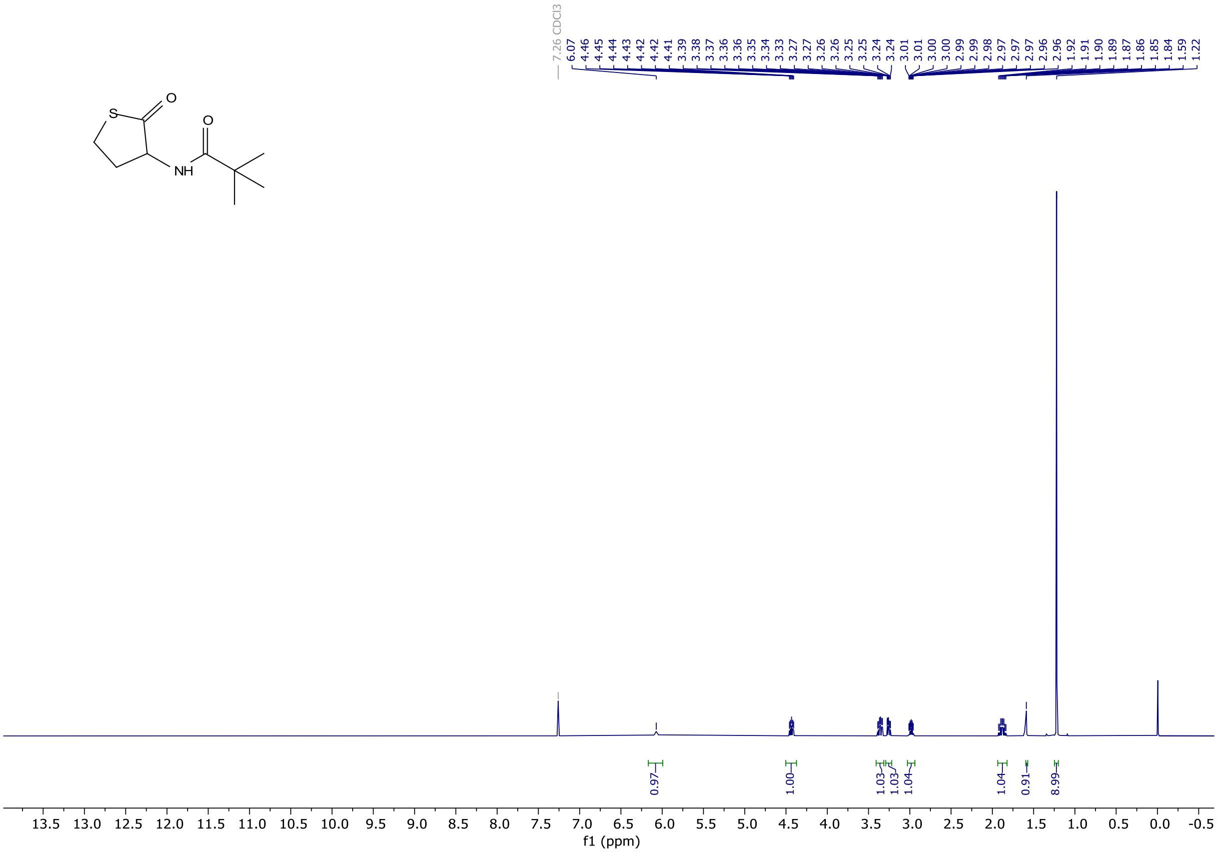

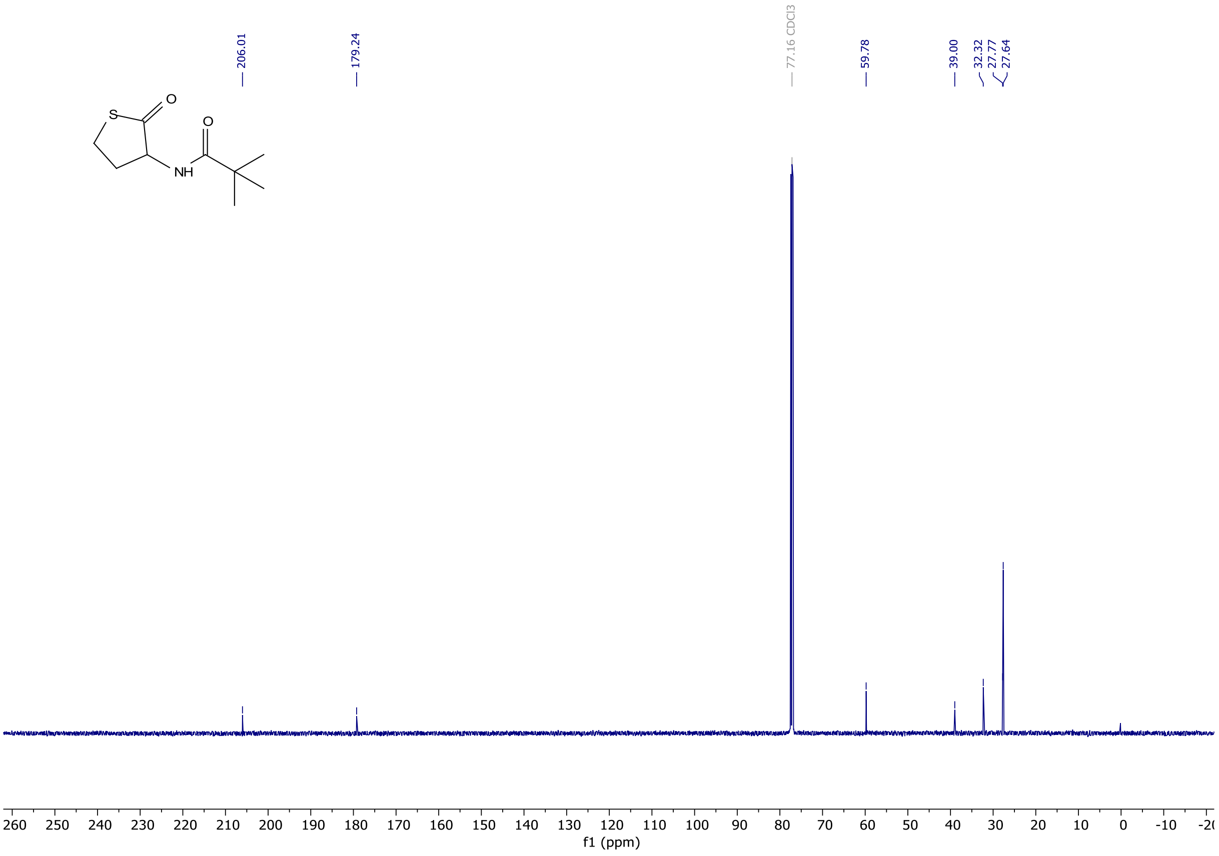

N-(2-Oxotetrahydrothiophen-3-yl)cyclopropanecarboxamide 1f

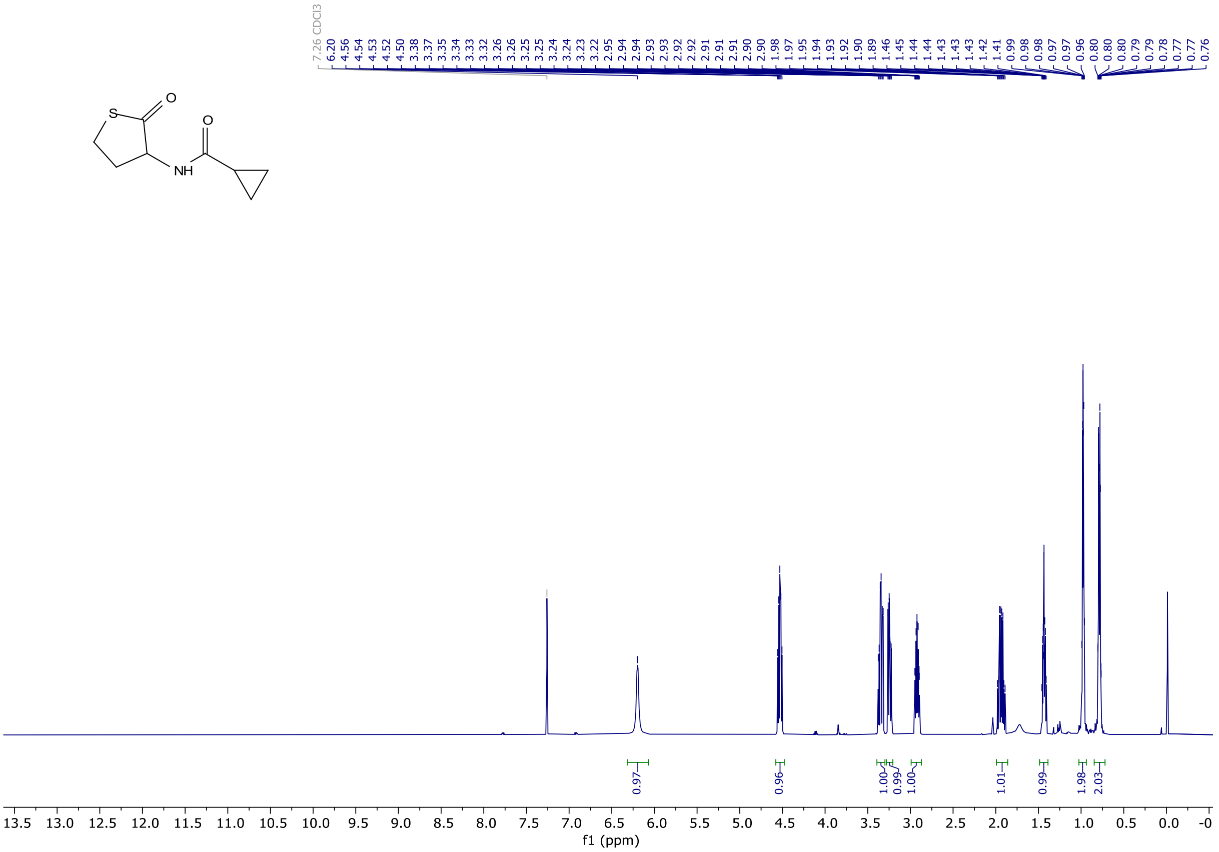

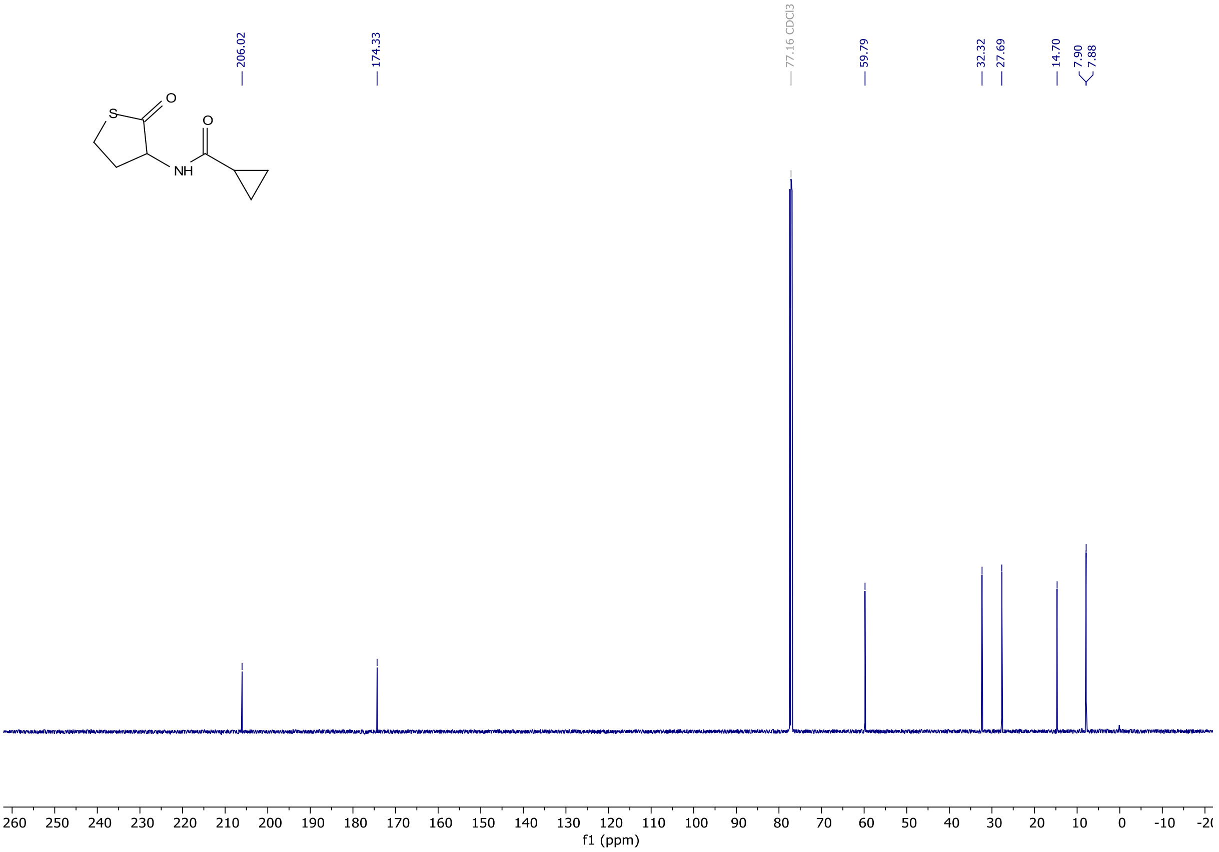

N-(2-Oxotetrahydrothiophen-3-yl)cyclobutanecarboxamide 1g

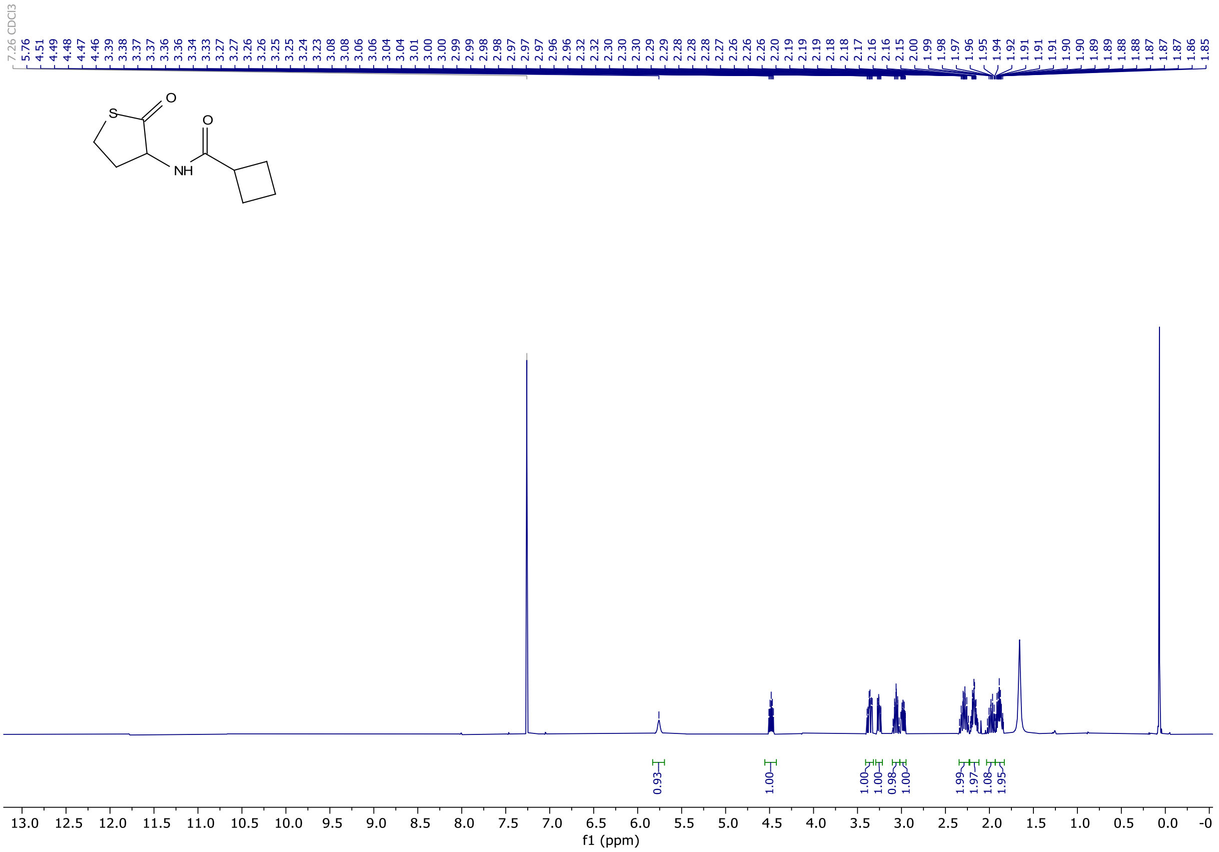

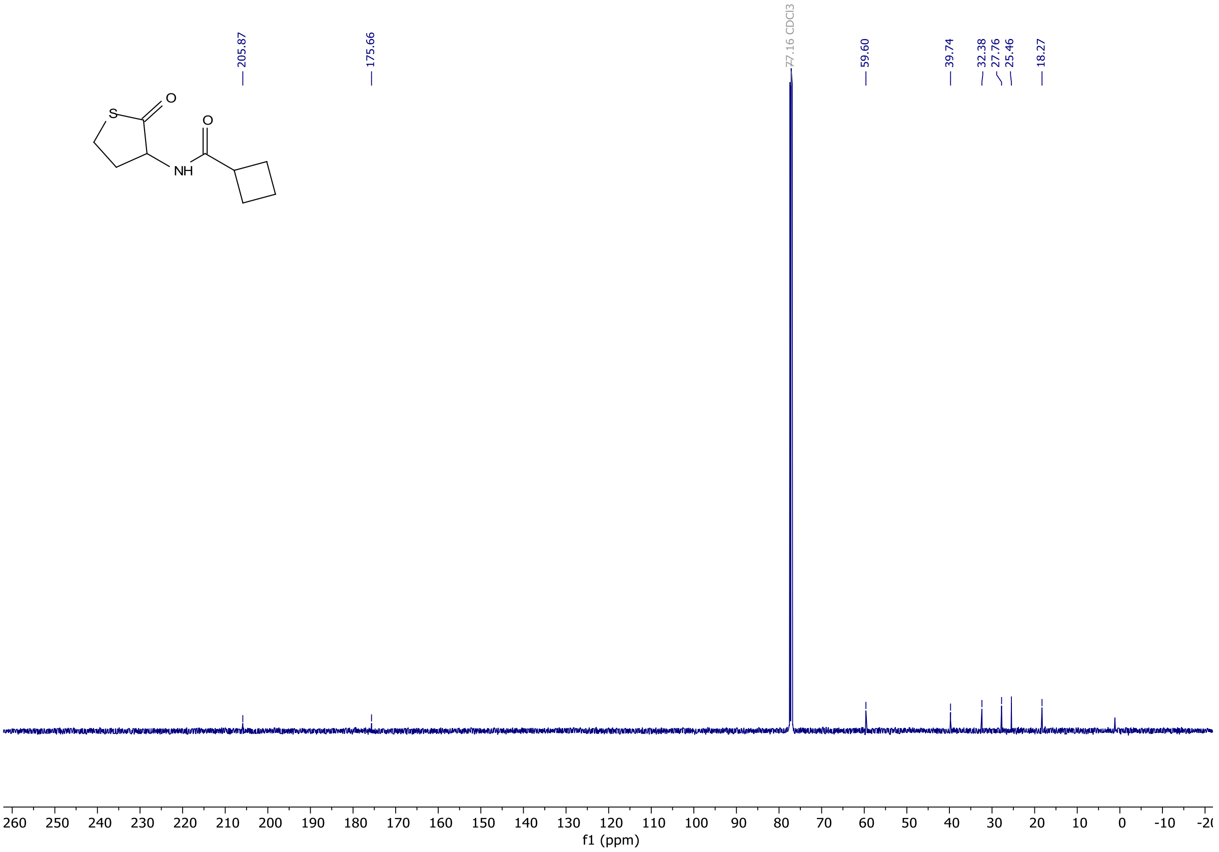

N-(2-Oxotetrahydrothiophen-3-yl)cyclohexanecarboxamide 1h

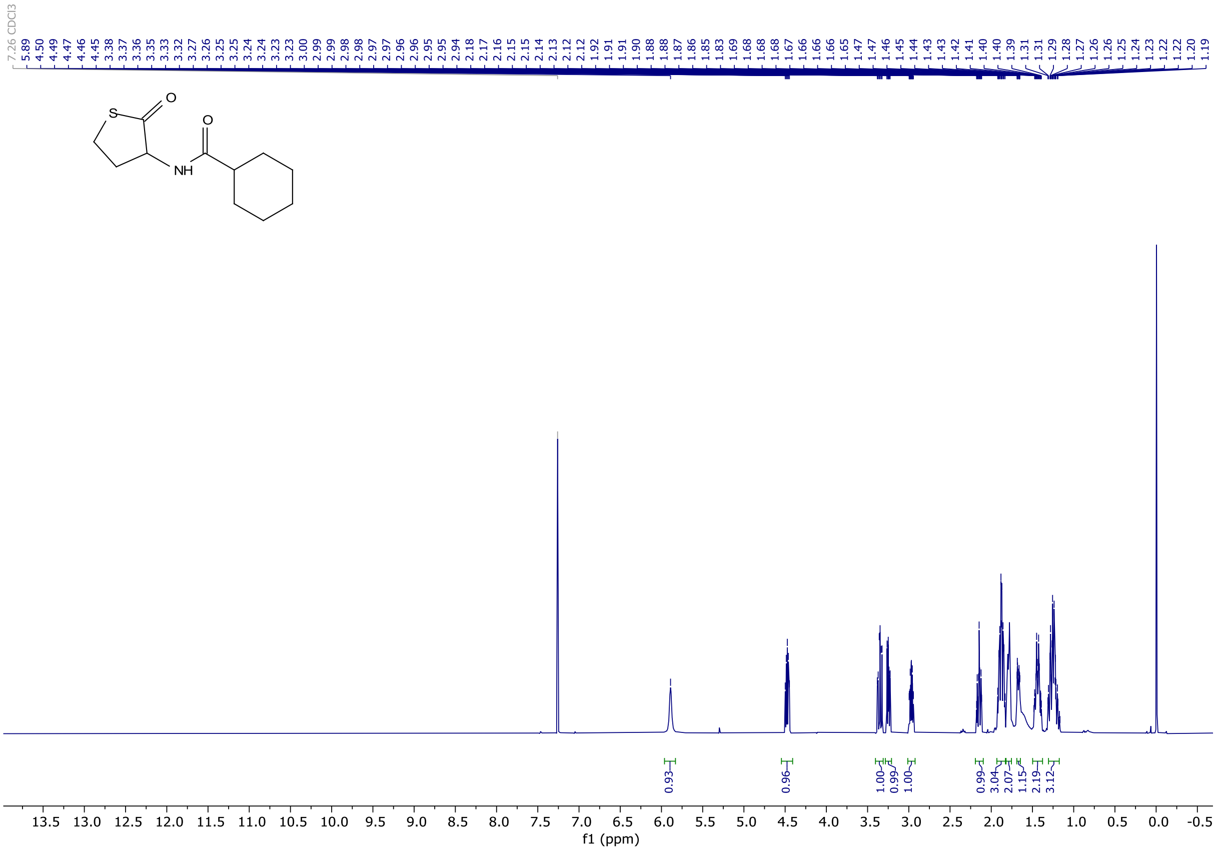

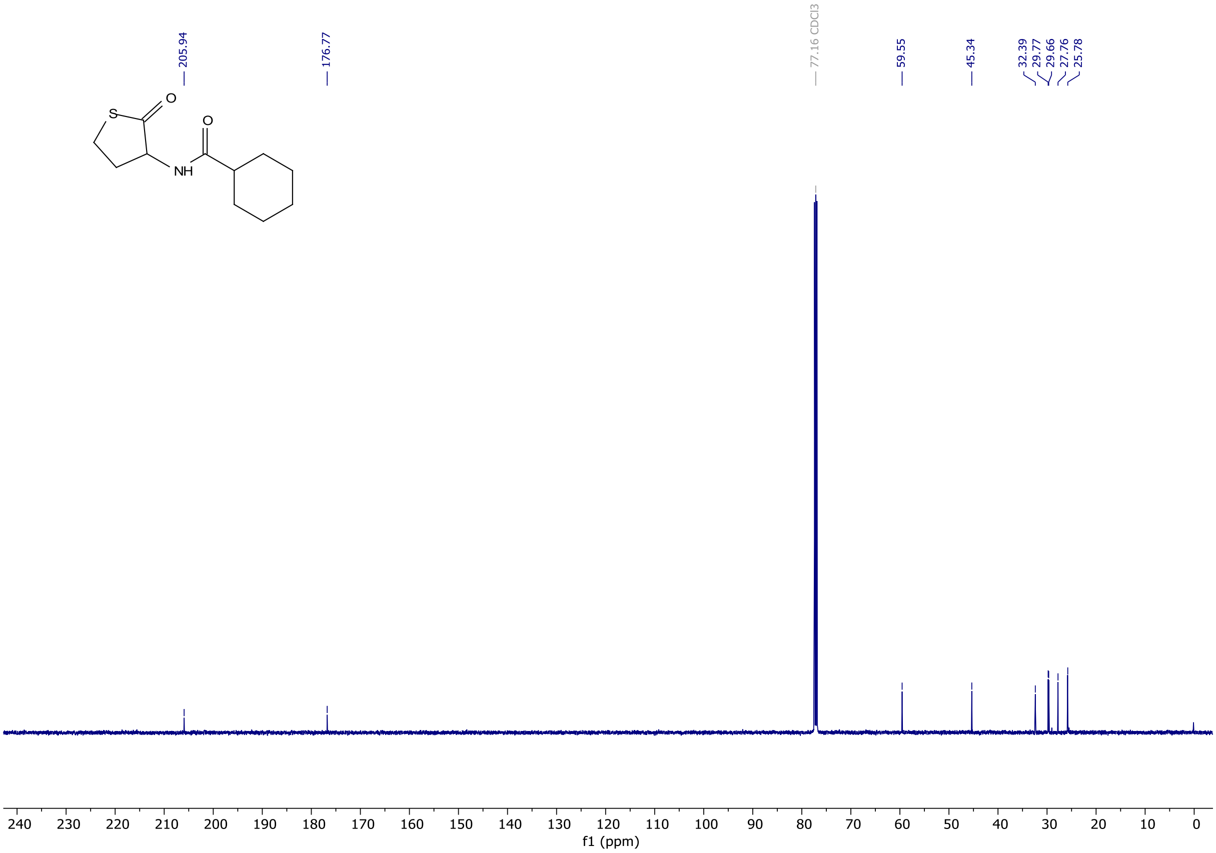

4-Fluoro-*N*-(2-oxotetrahydrothiophen-3-yl)benzamide 1i

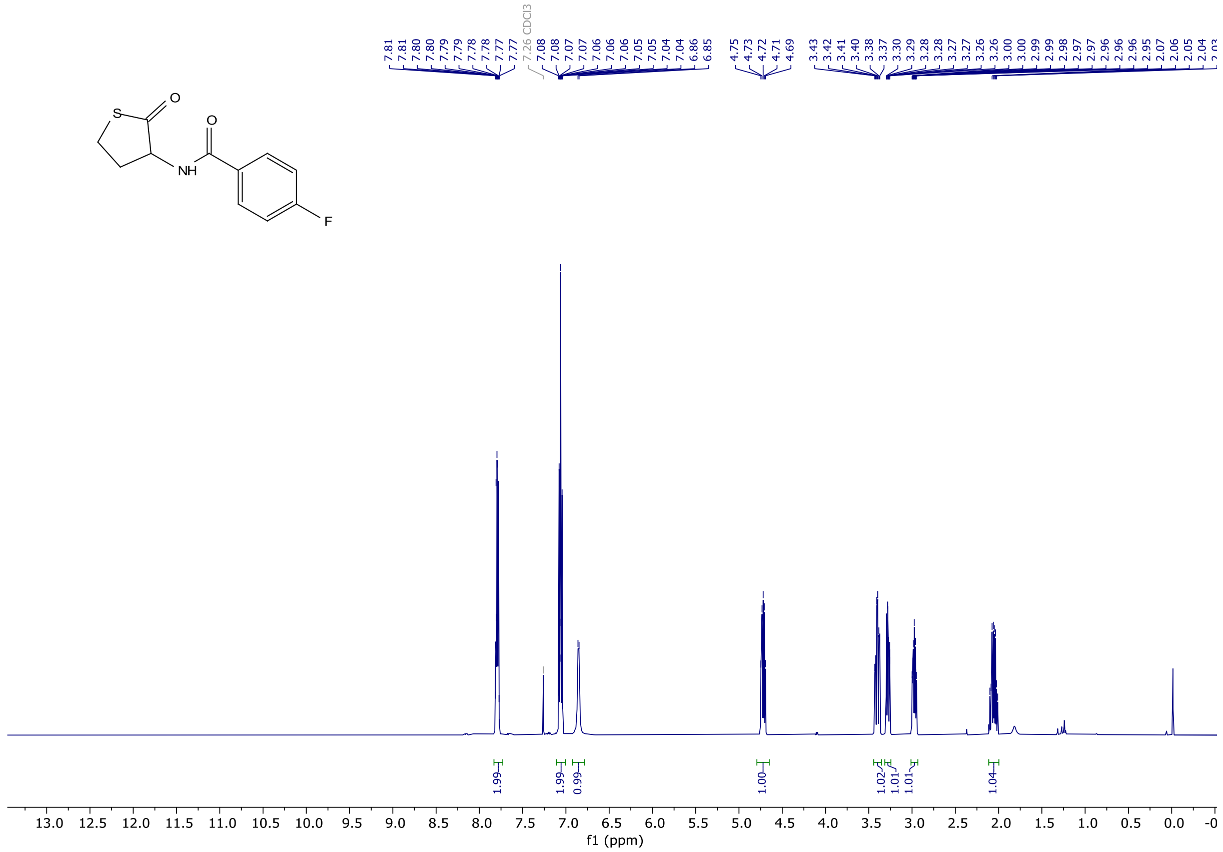

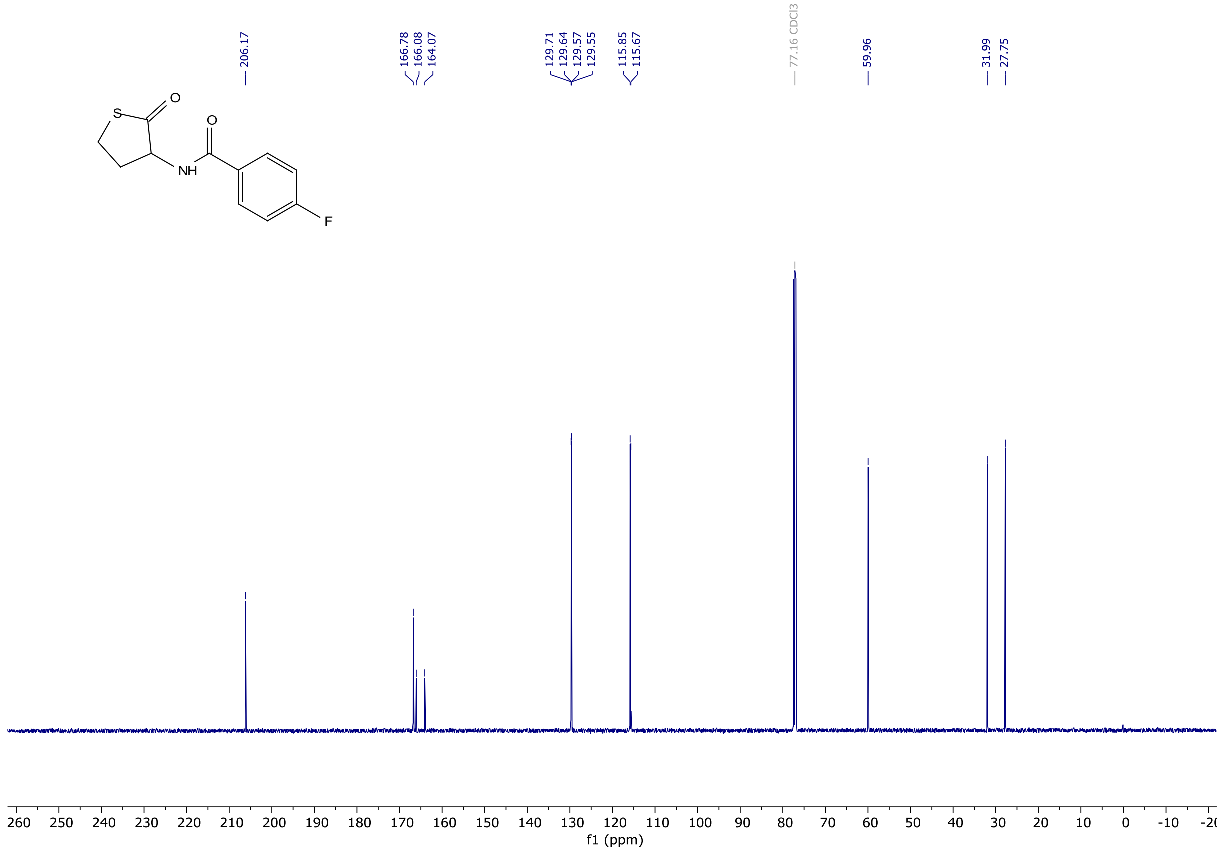

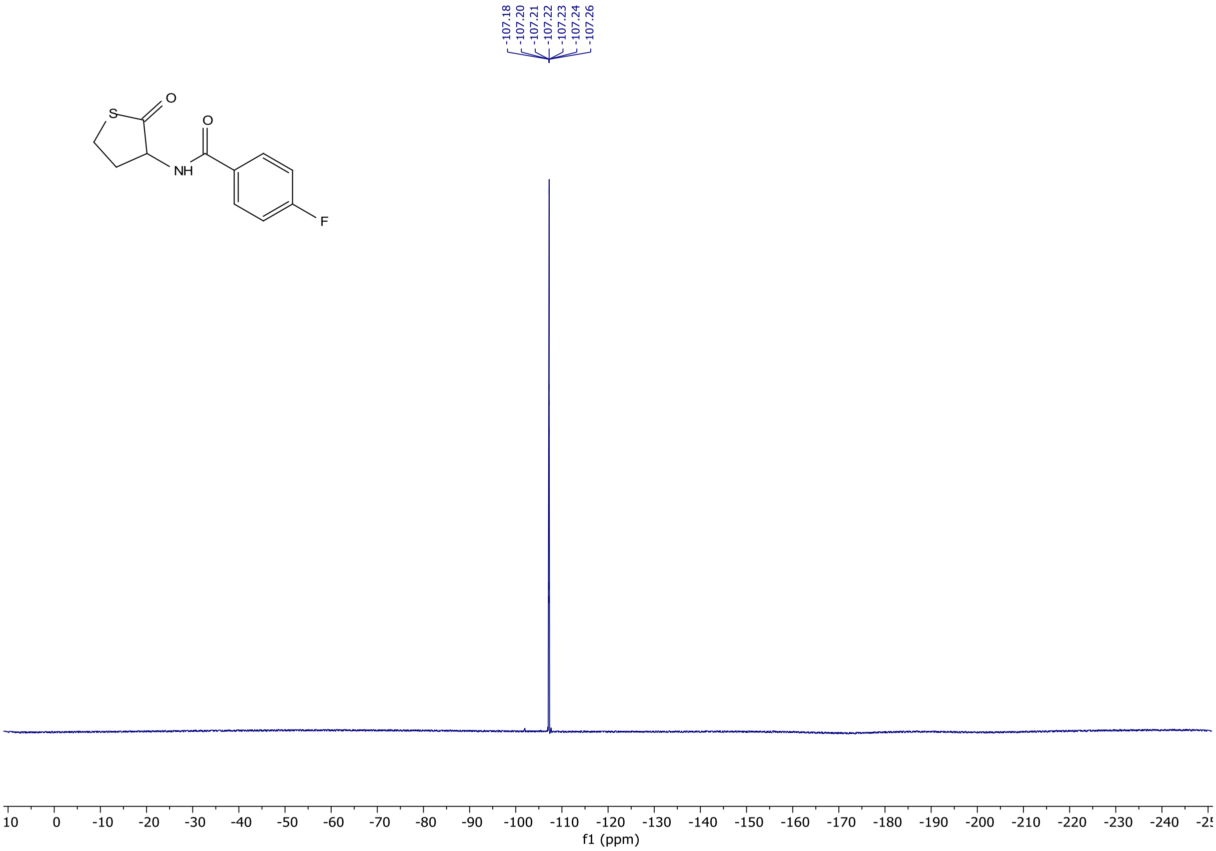

-107.18
-107.20
-107.21
-107.22
-107.23
-107.24
-107.26
S
O
O
NH
F
10 0 -10 -20 -30 -40 -50 -60 -70 -80 -90 -100 -110 -120 -130 -140 -150 -160 -170 -180 -190 -200 -210 -220 -230 -240
f1 (ppm)

4-Chloro-*N*-(2-oxotetrahydrothiophen-3-yl)benzamide 1j

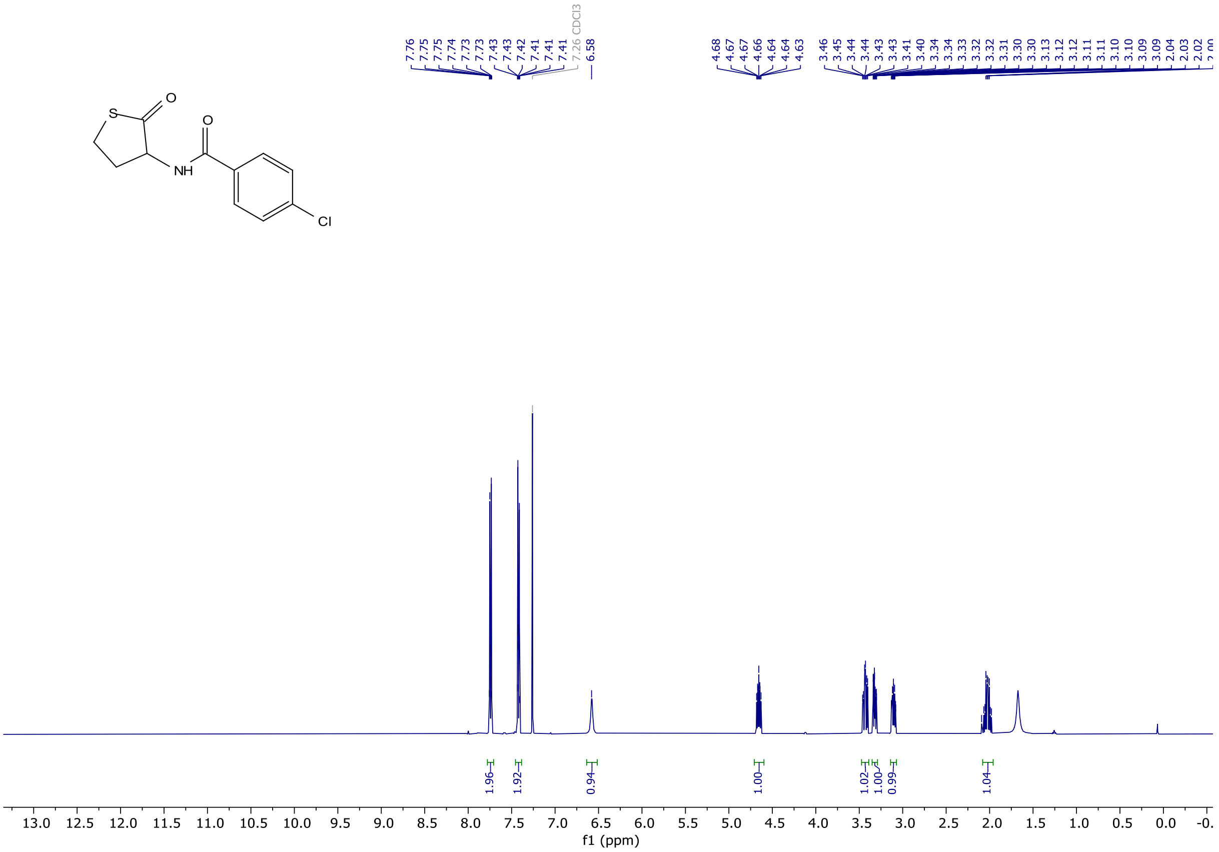

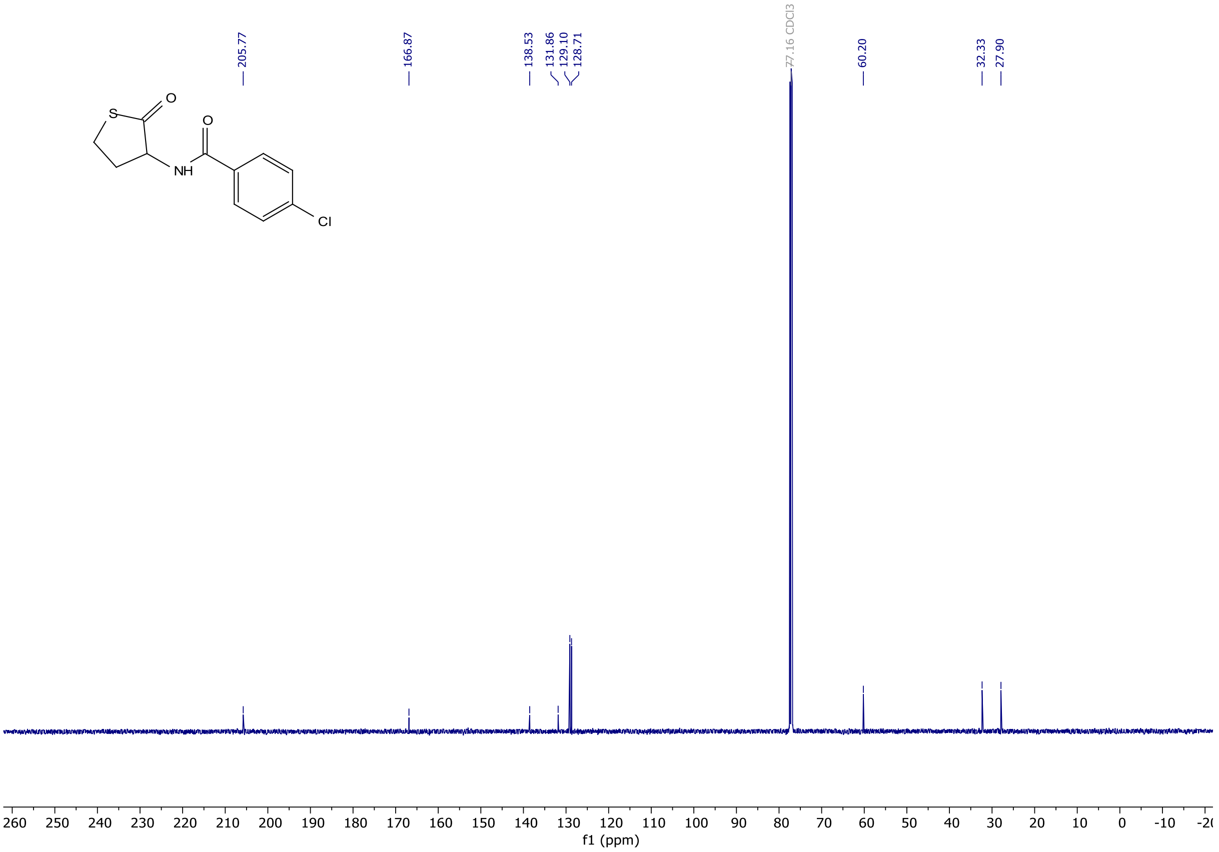

2-Chloro-*N*-(2-oxotetrahydrothiophen-3-yl)benzamide 1k

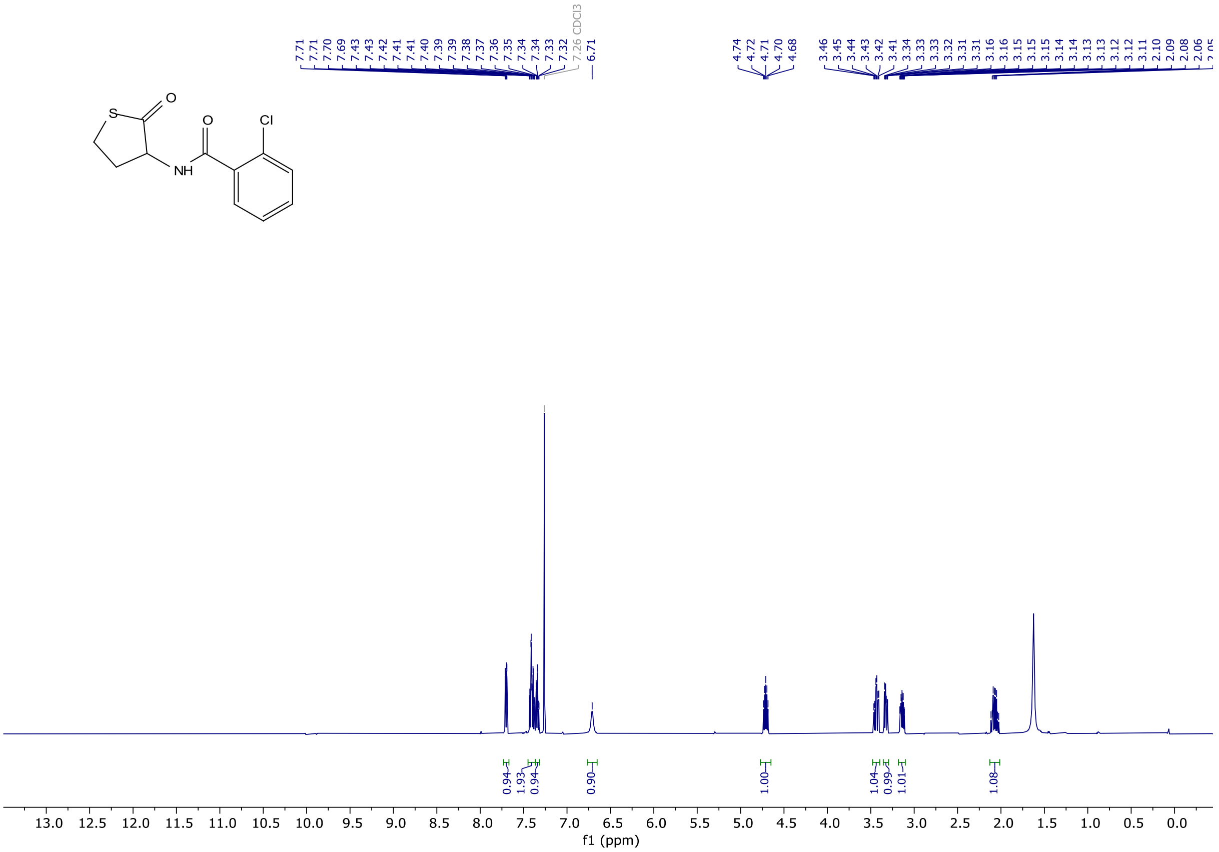

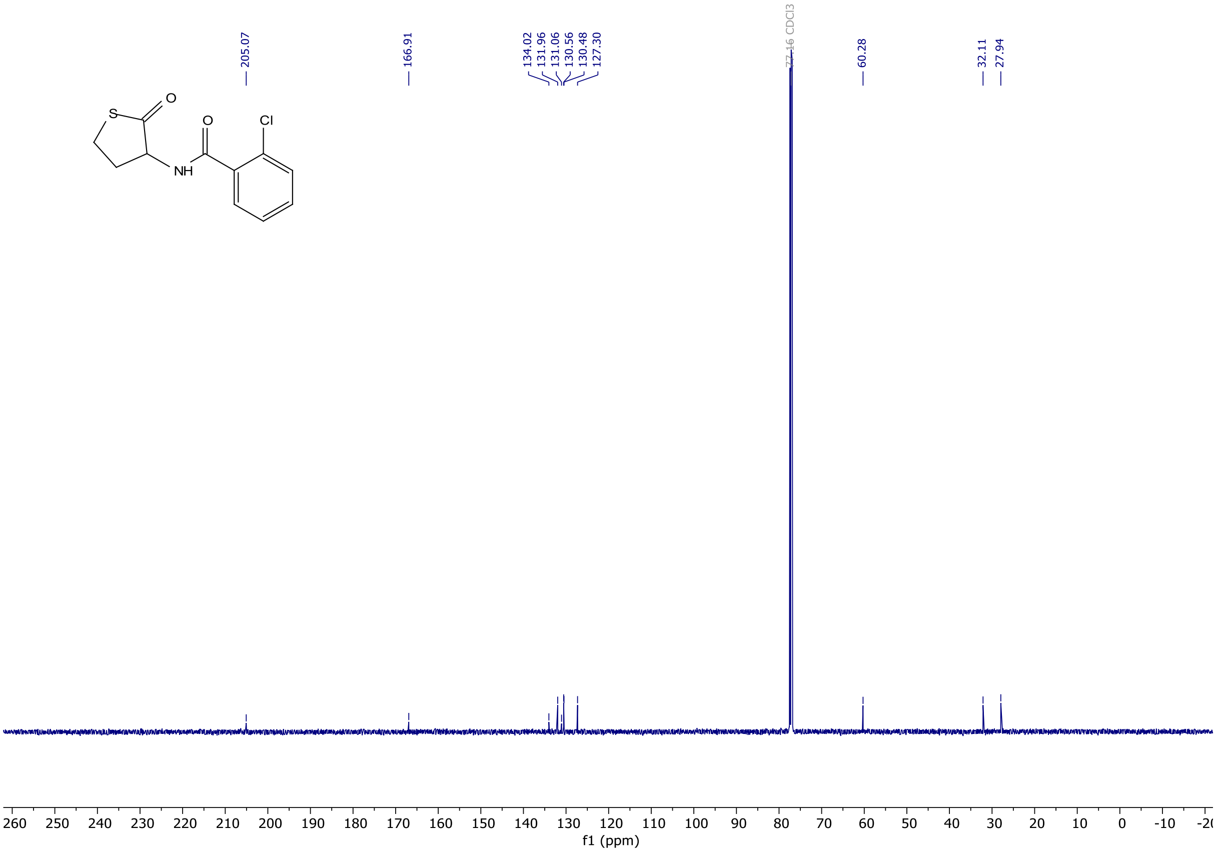

4-Methyl-*N*-(2-oxotetrahydrothiophen-3-yl)benzamide 1l

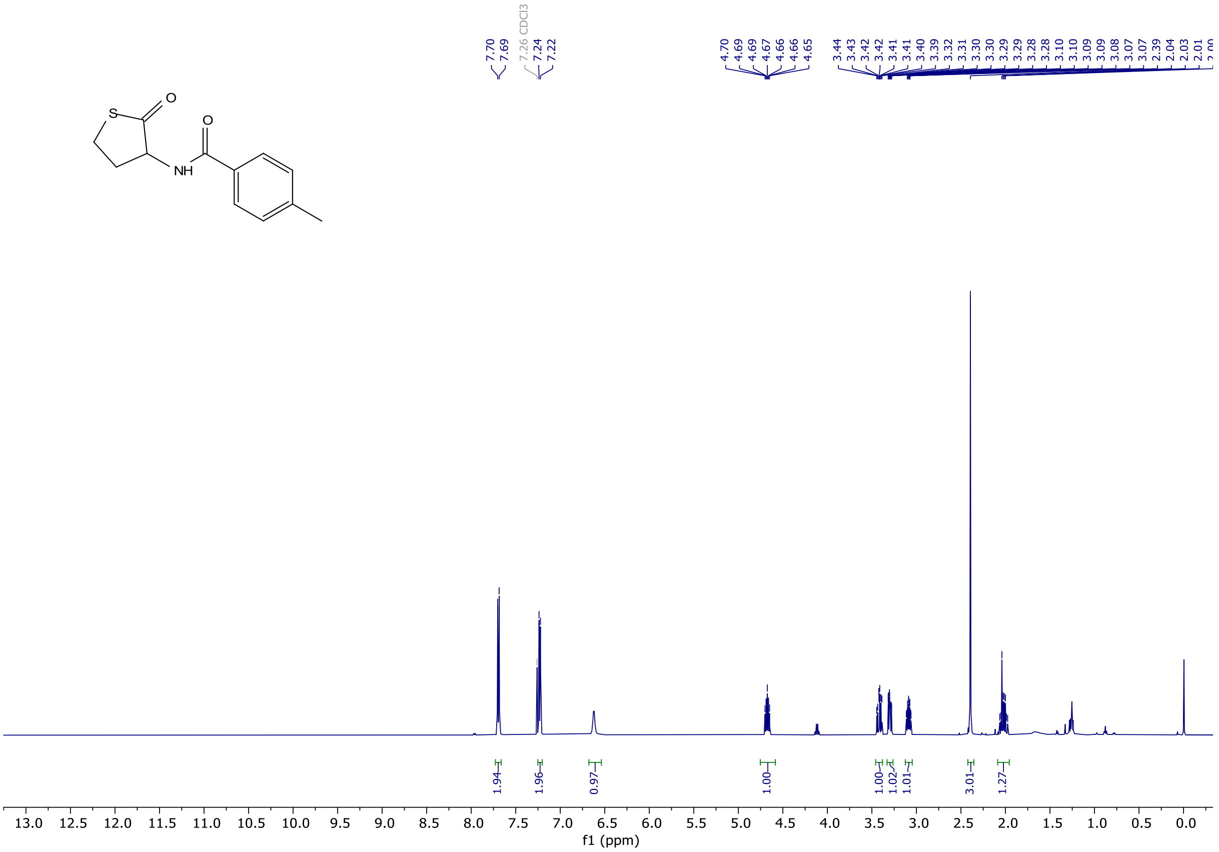

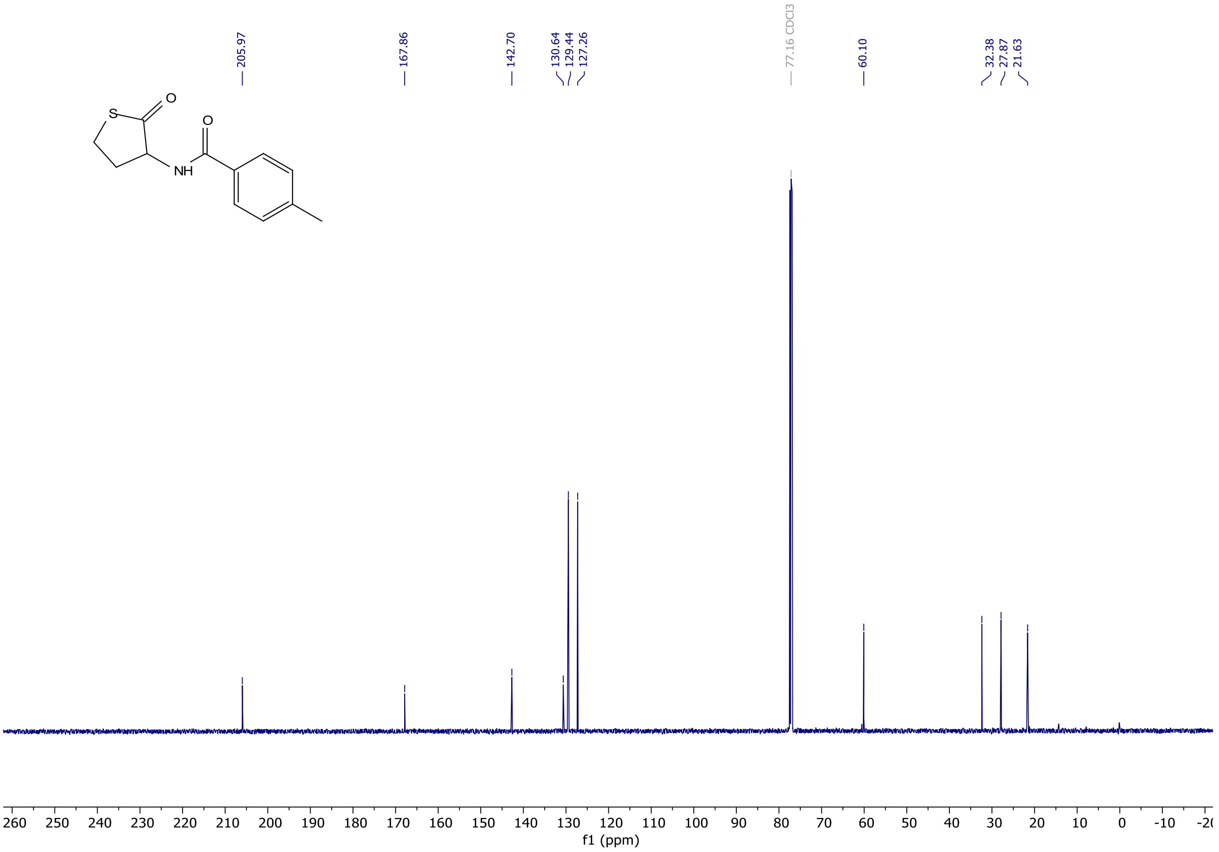

4-Methoxy-*N*-(2-oxotetrahydrothiophen-3-yl)benzamide 1m

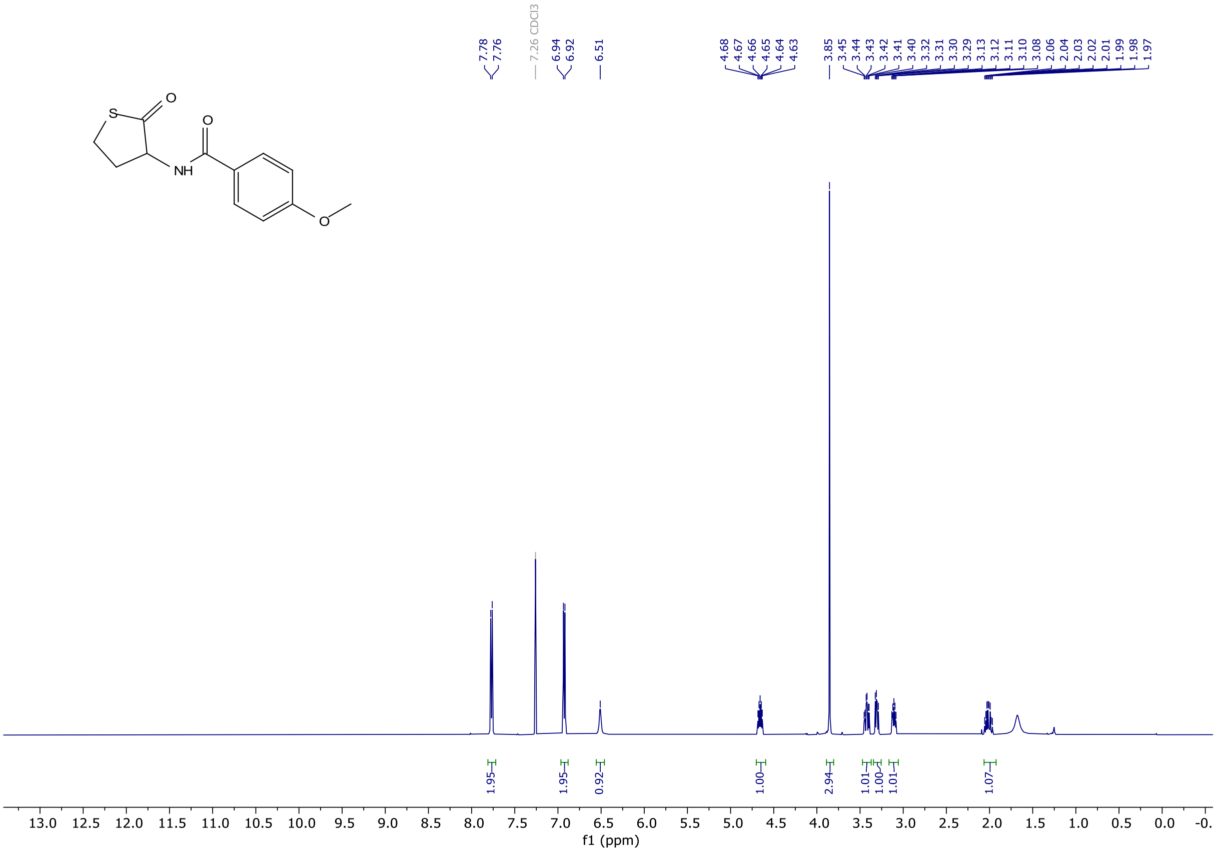

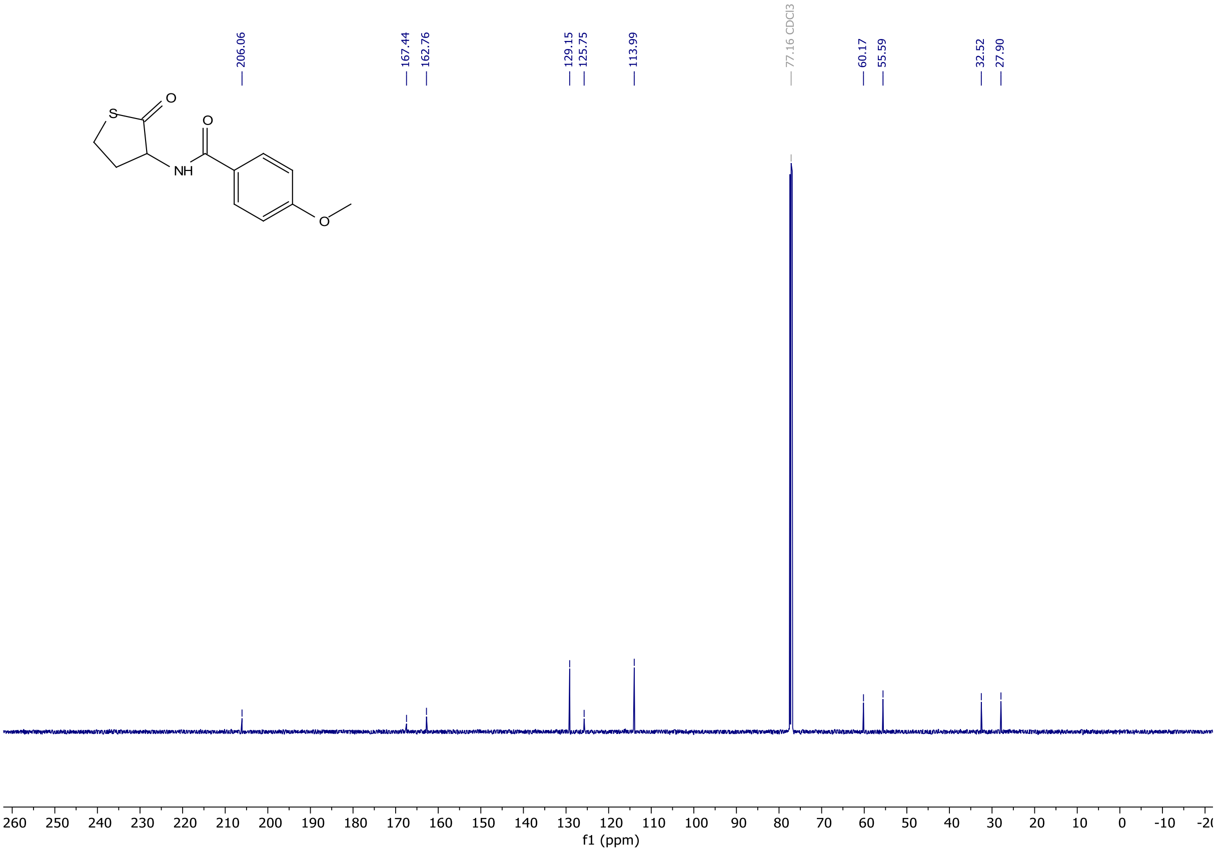

Hexanoyl homocysteine 2a

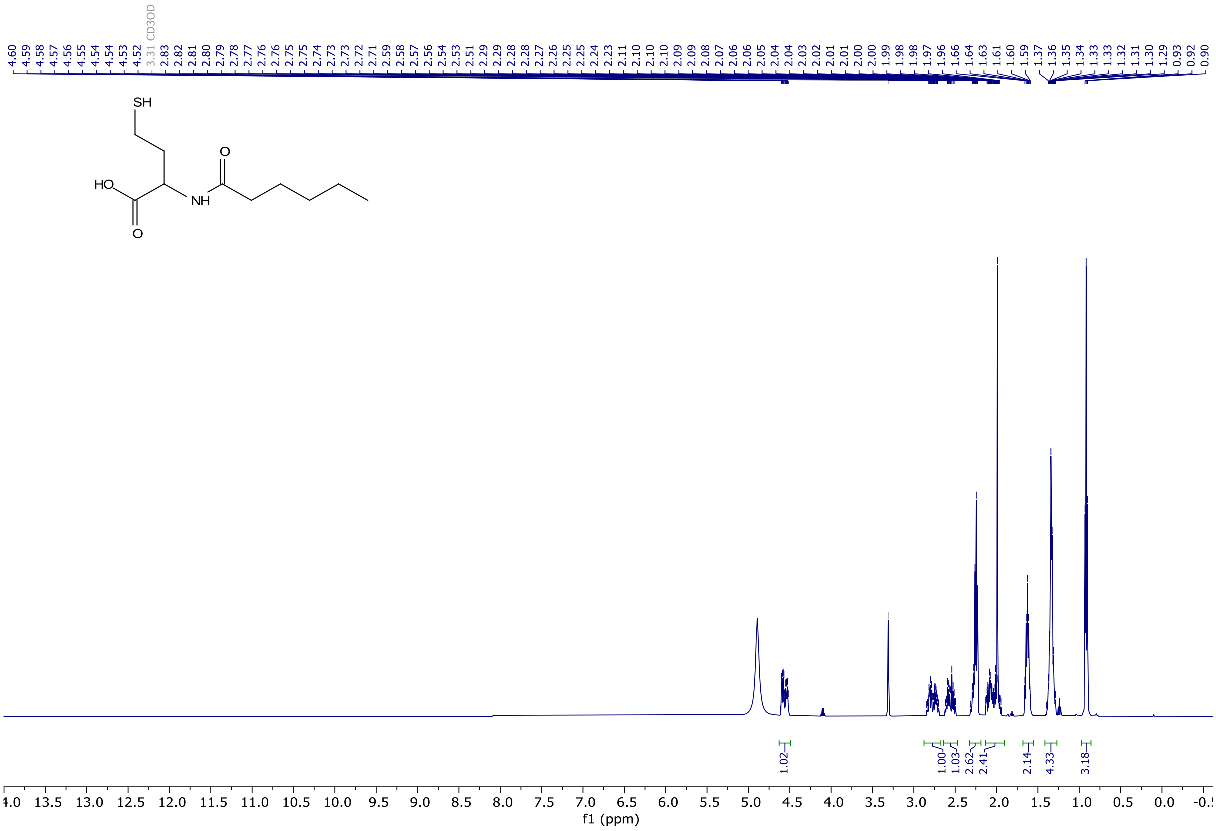

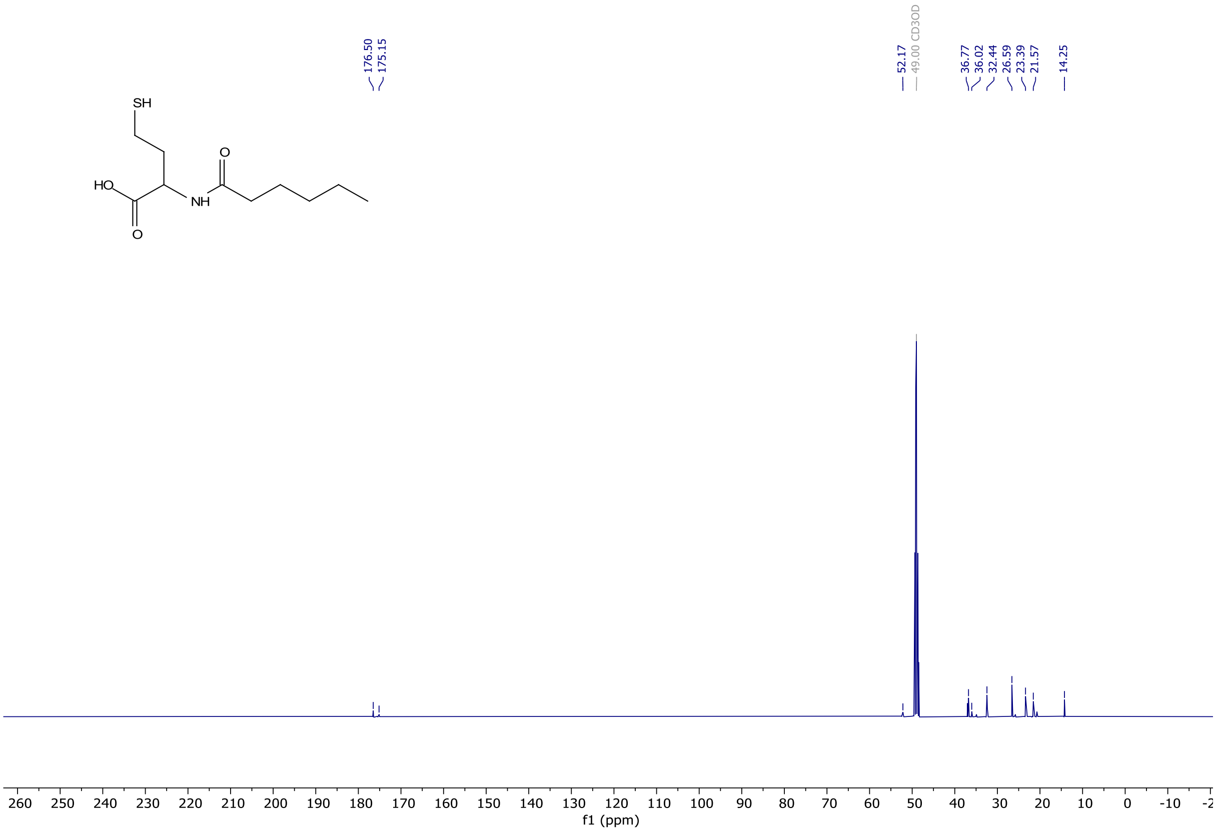

Benzoyl homocysteine 2b

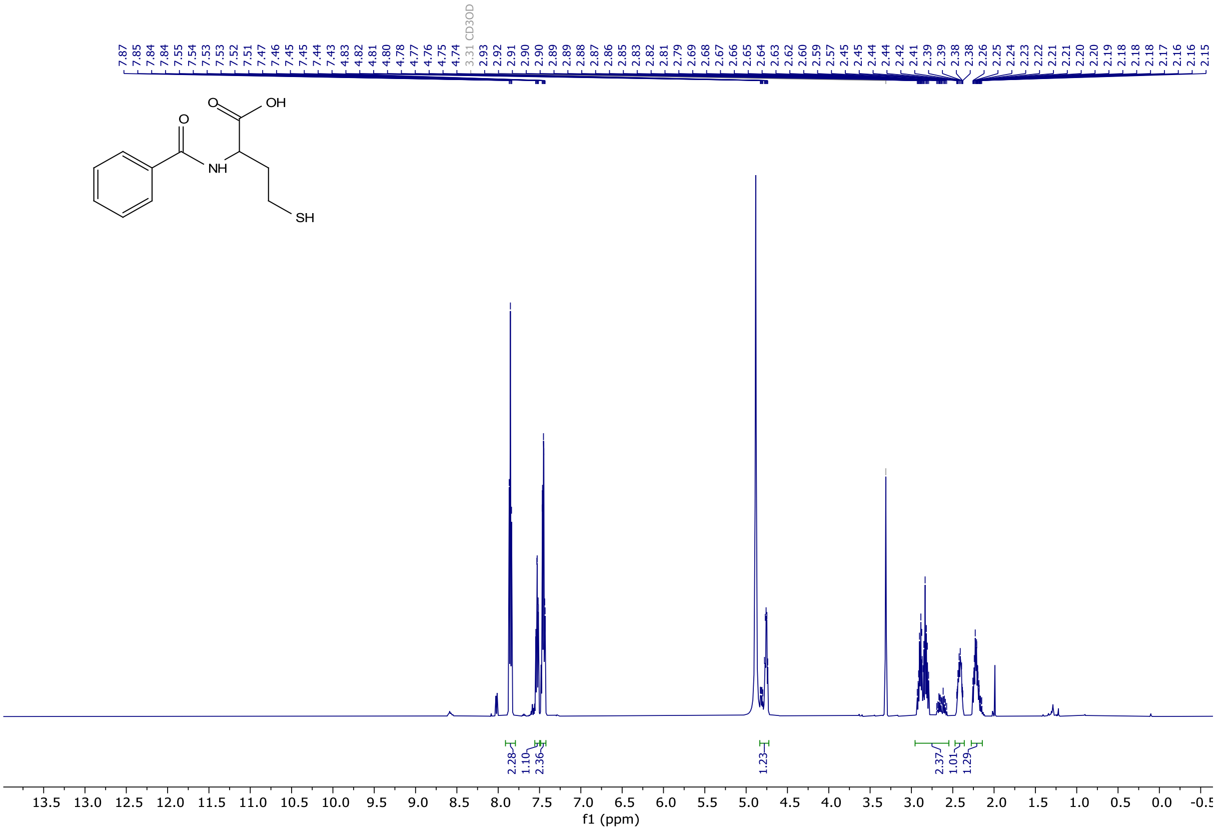

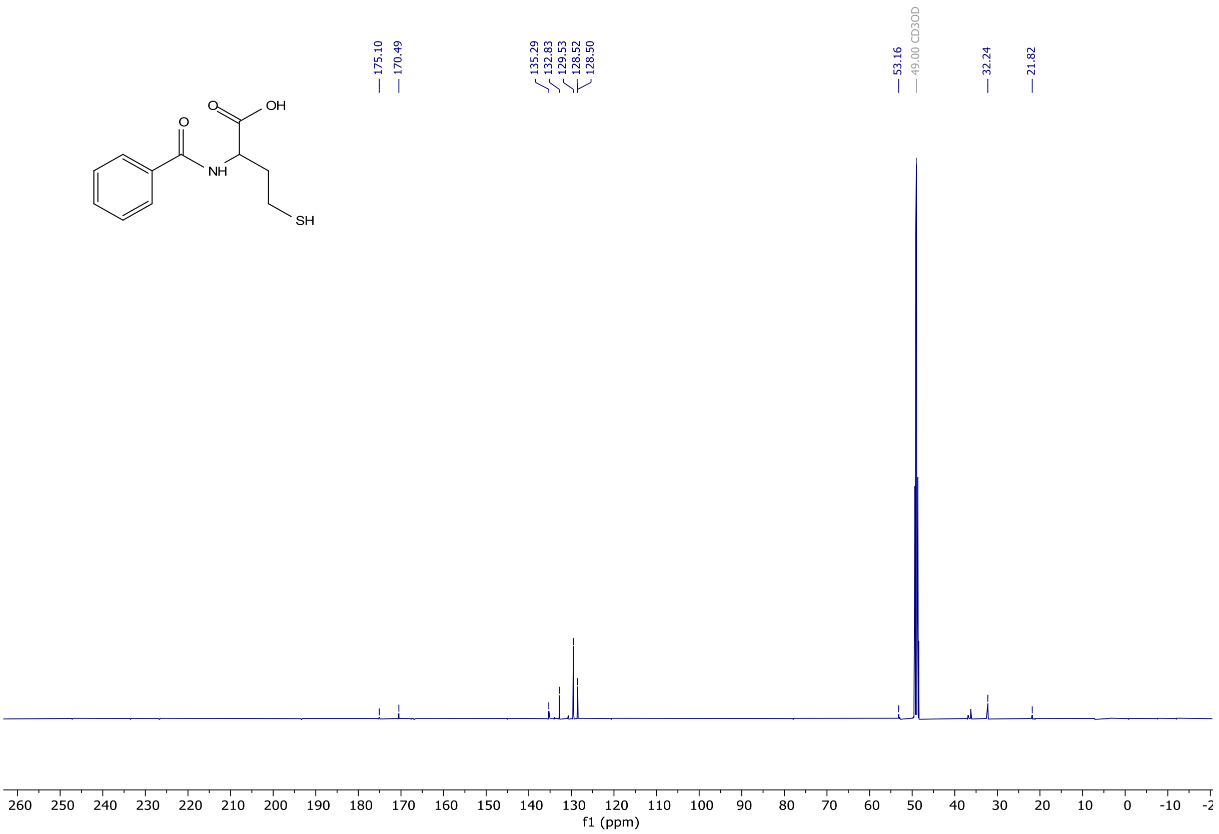

(2-Phenylacetyl)homocysteine 2c

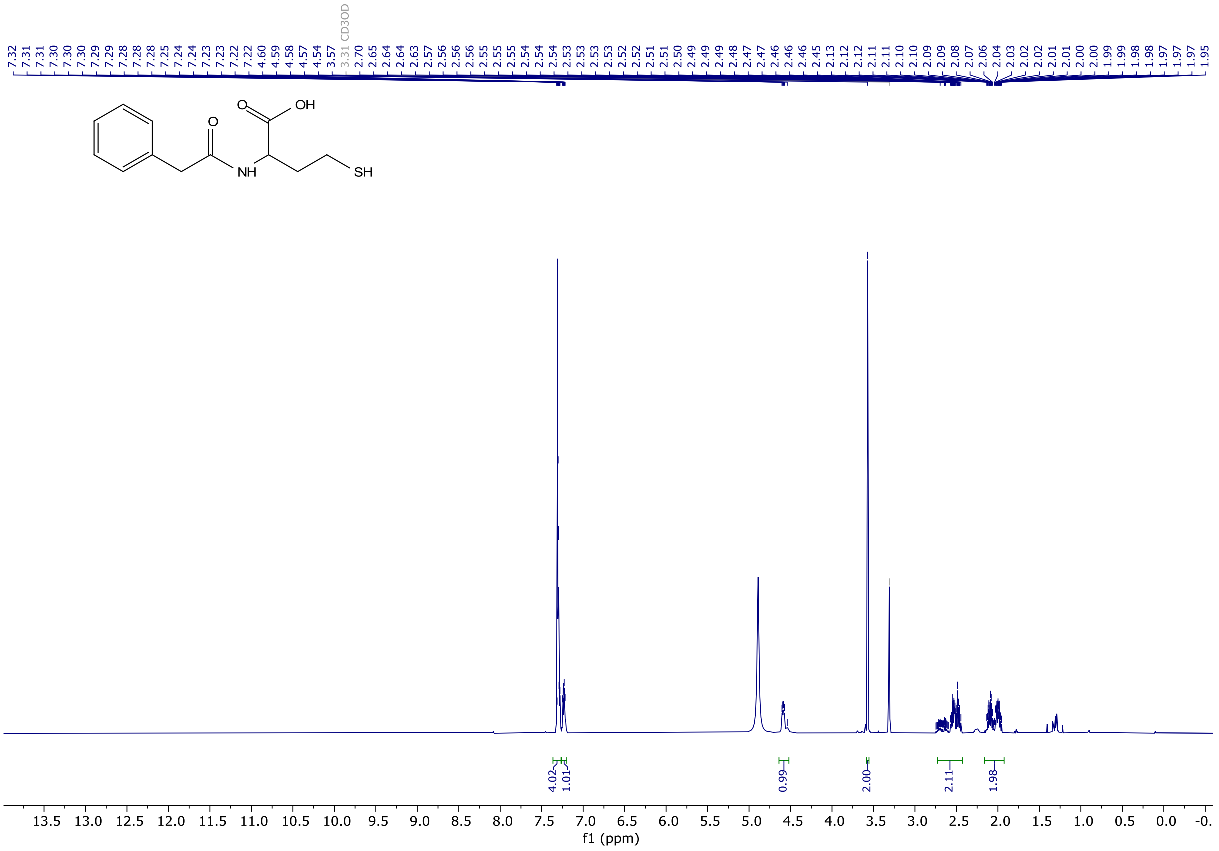

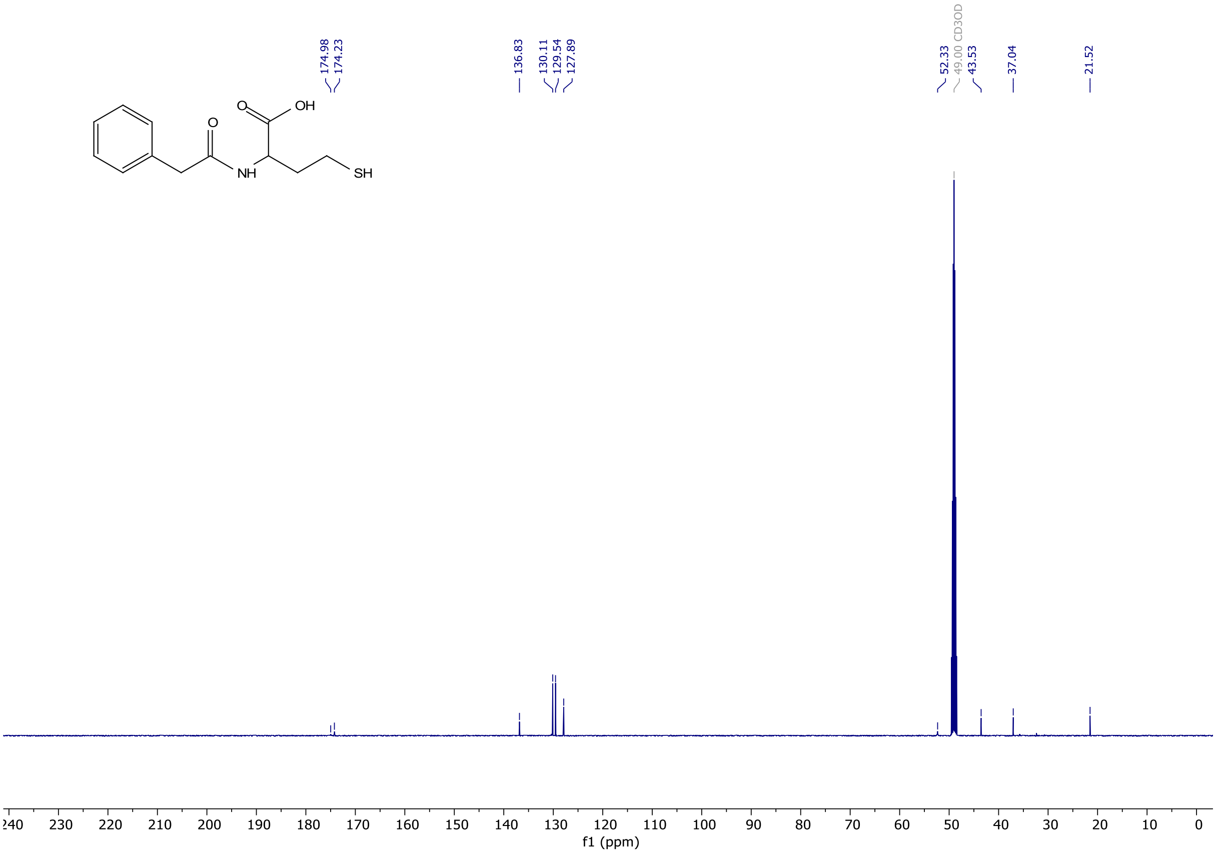

Isobutyrylhomocysteine 2d

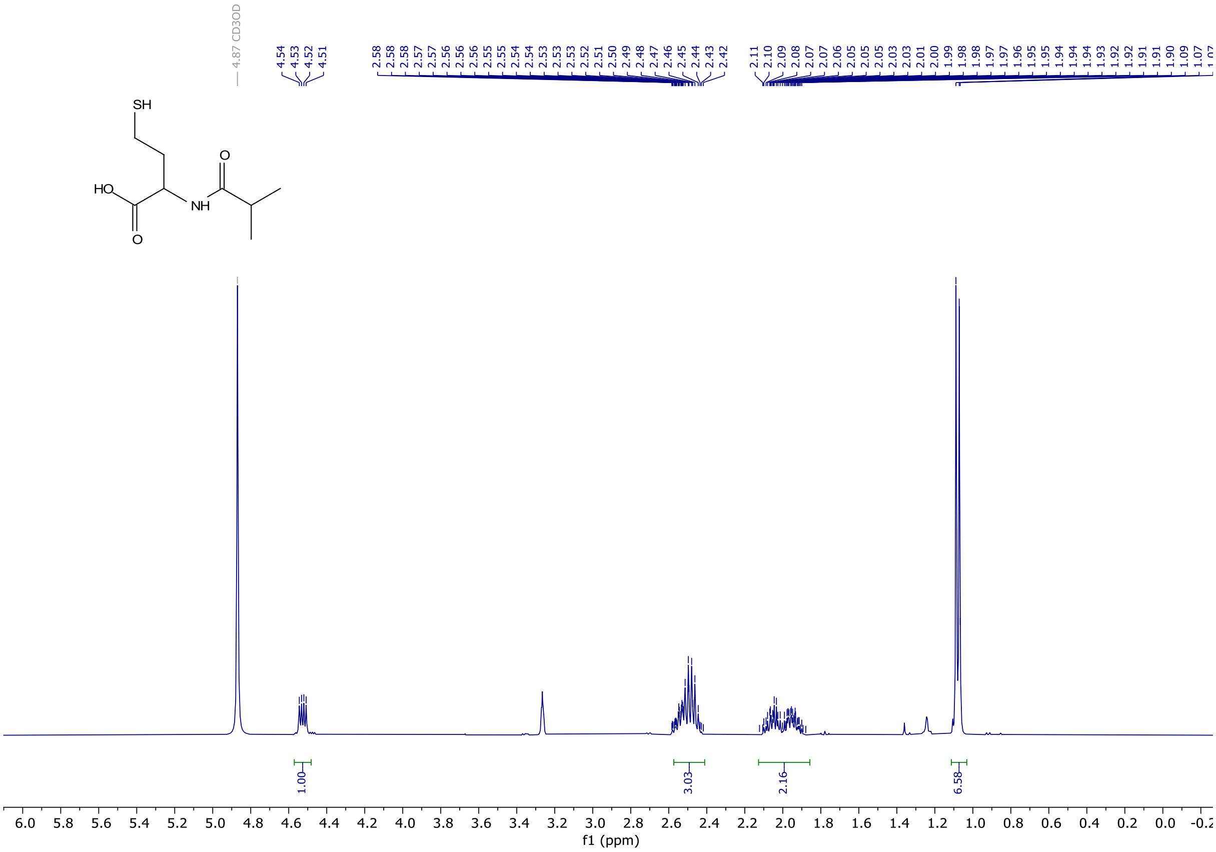

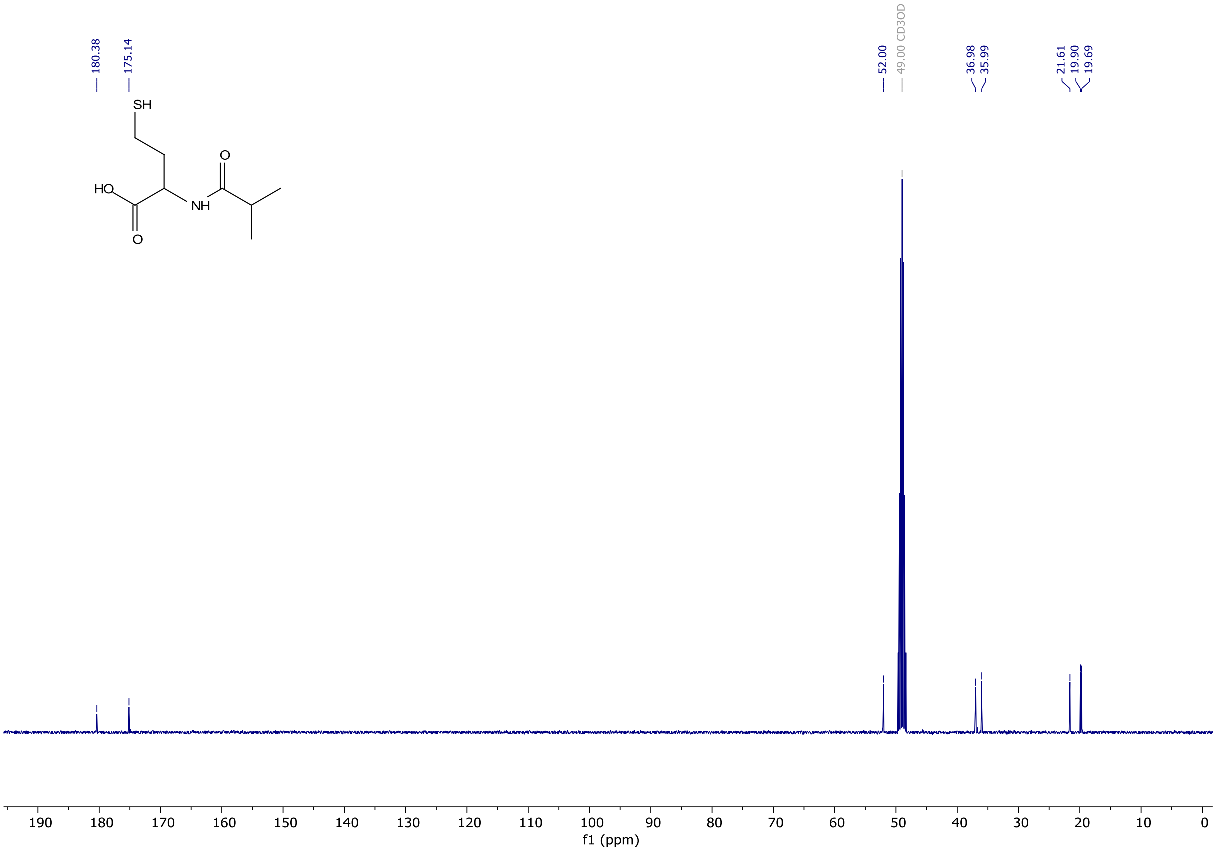

Pivaloylhomocysteine 2e

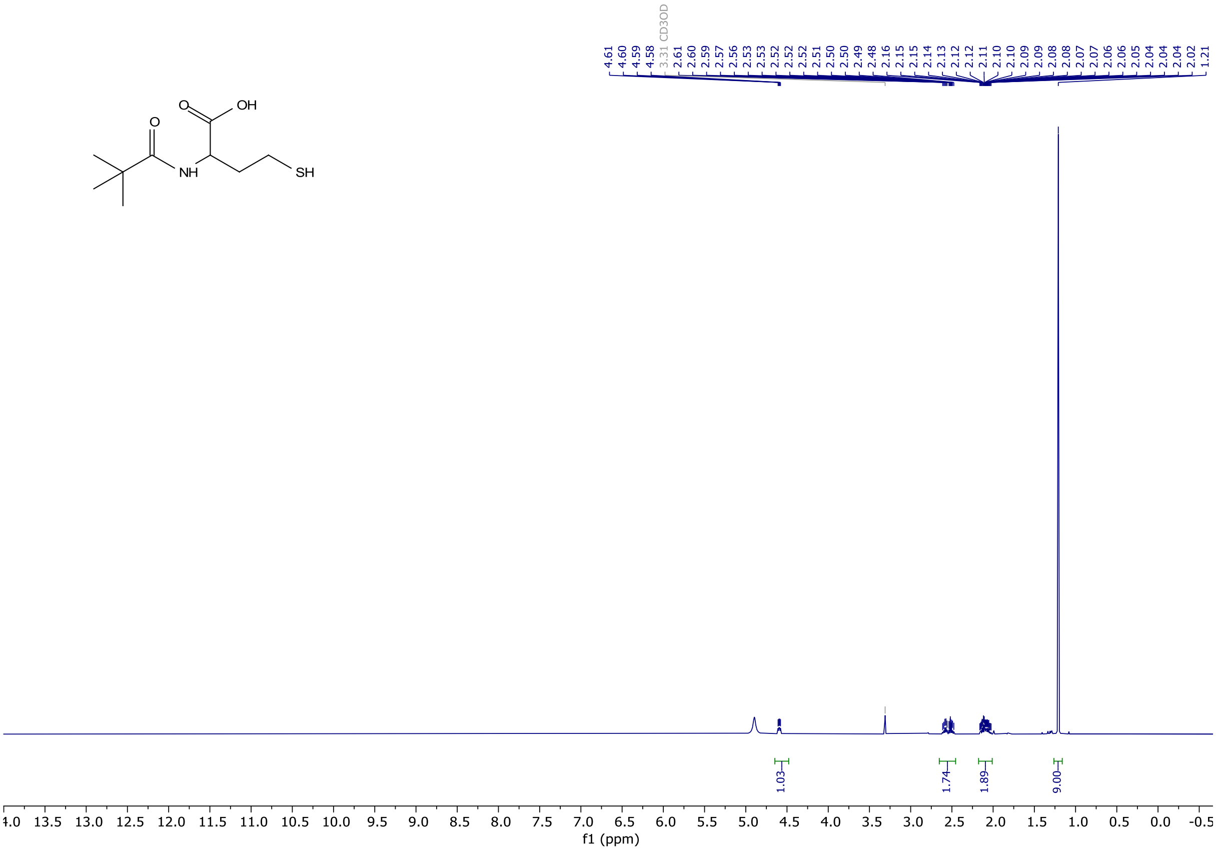

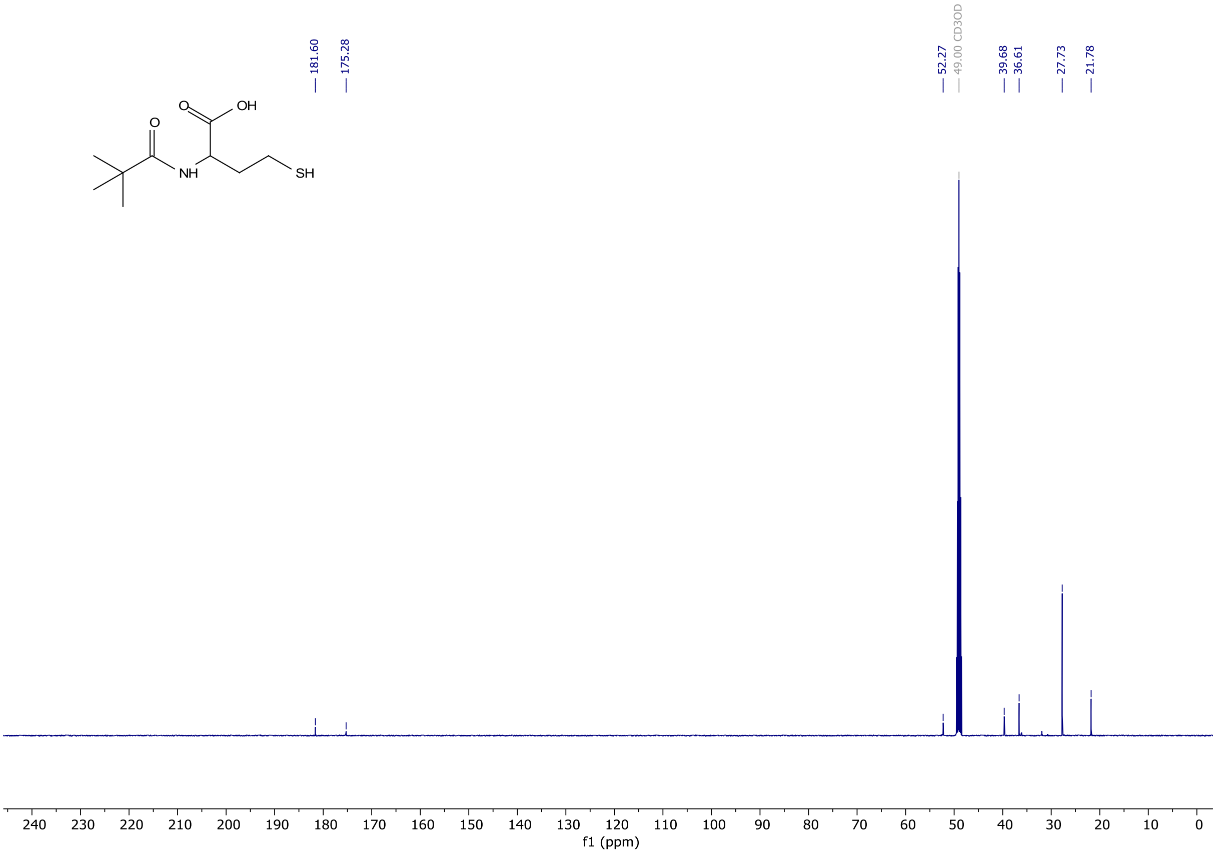

(Cyclopropanecarbonyl)homocysteine 2f

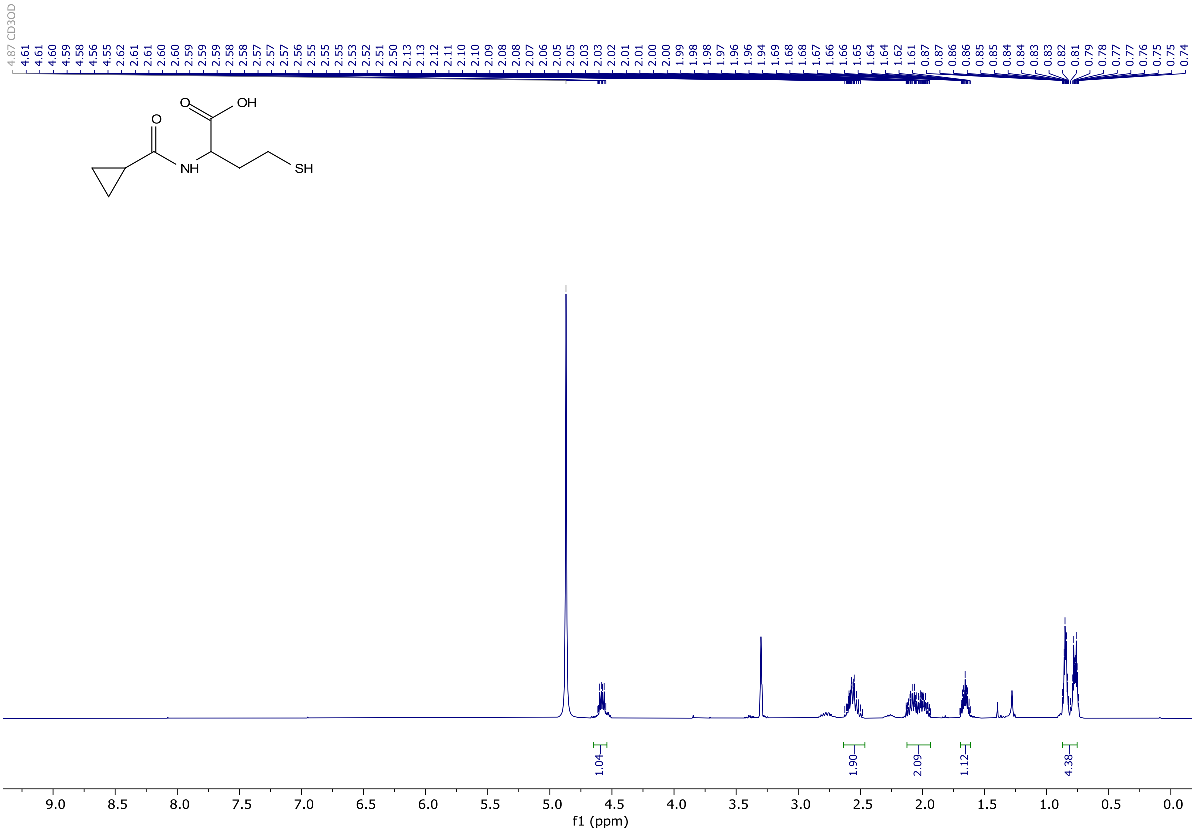

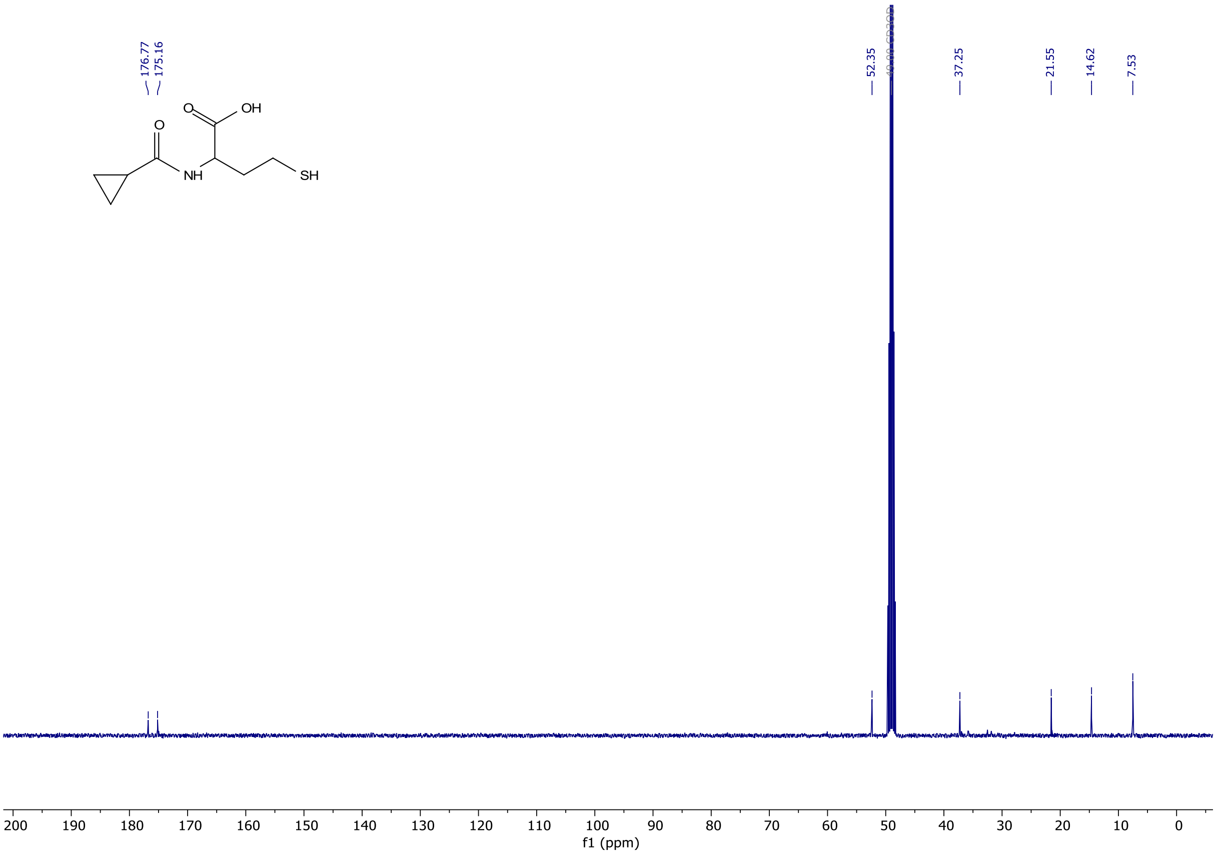

(Cyclobutanecarbonyl)homocysteine 2g

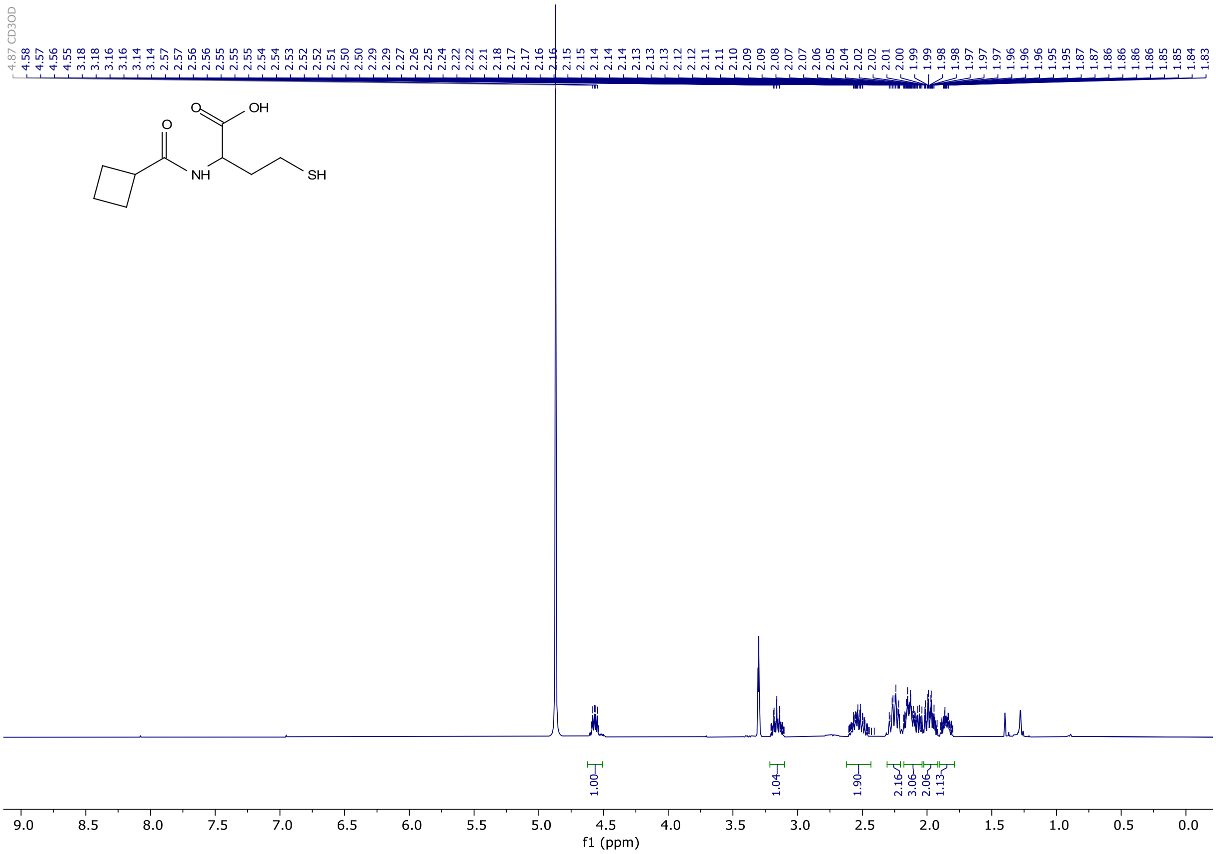

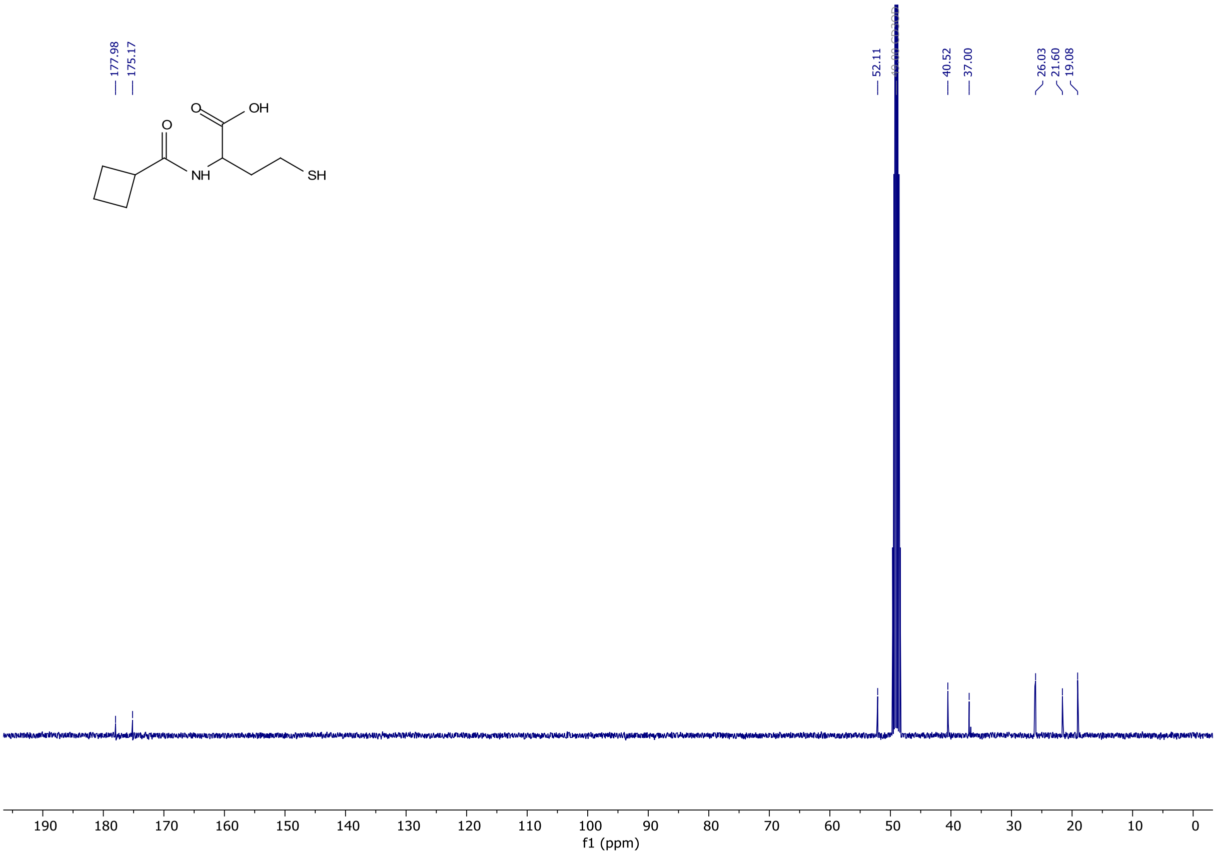

(Cyclohexanecarbonyl)homocysteine 2h

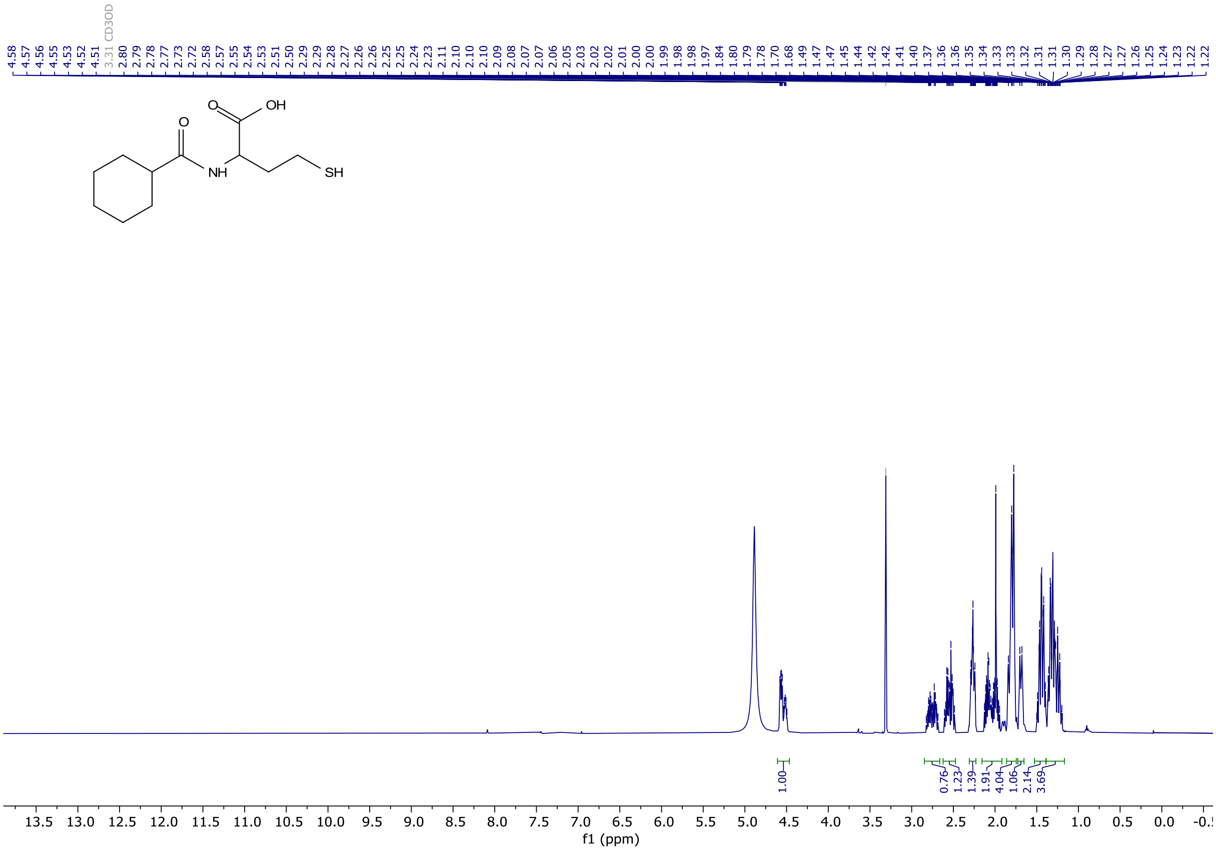

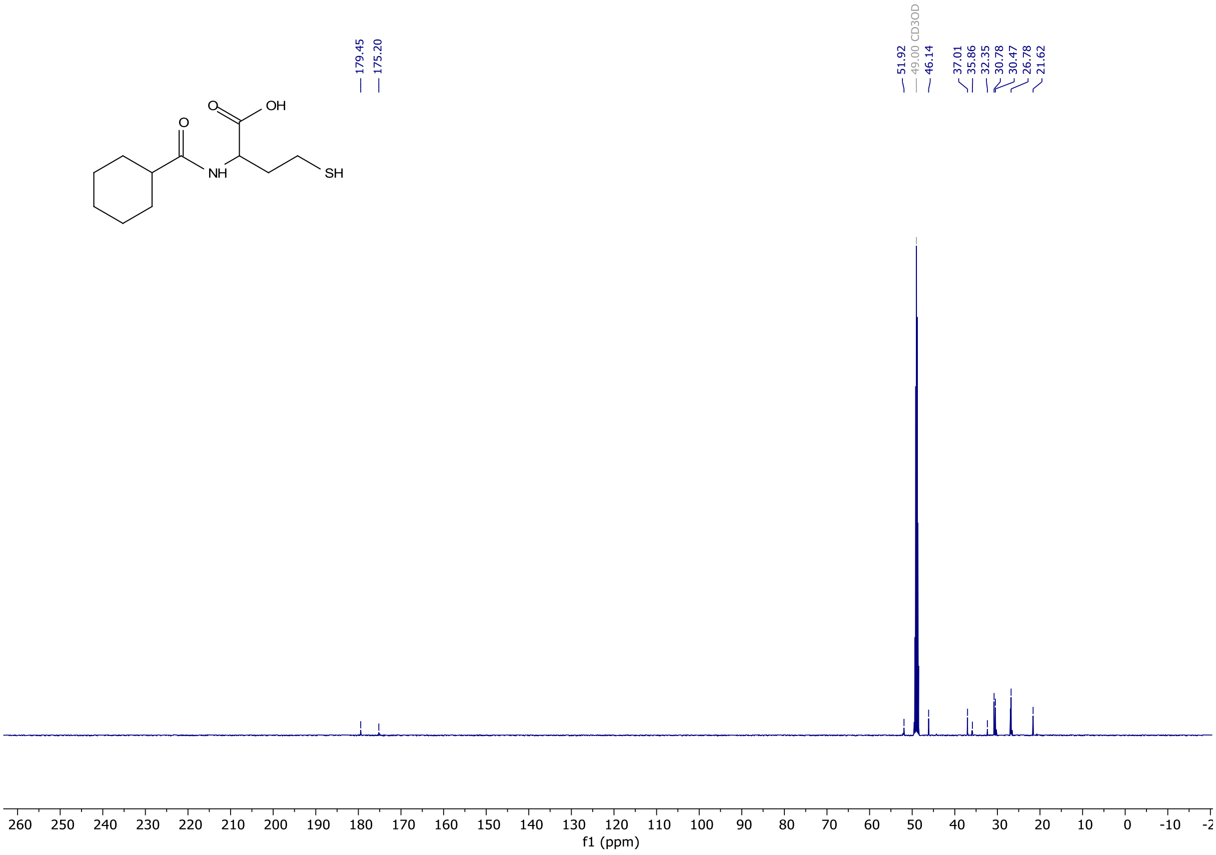

(4-Fluorobenzoyl)homocysteine 2i

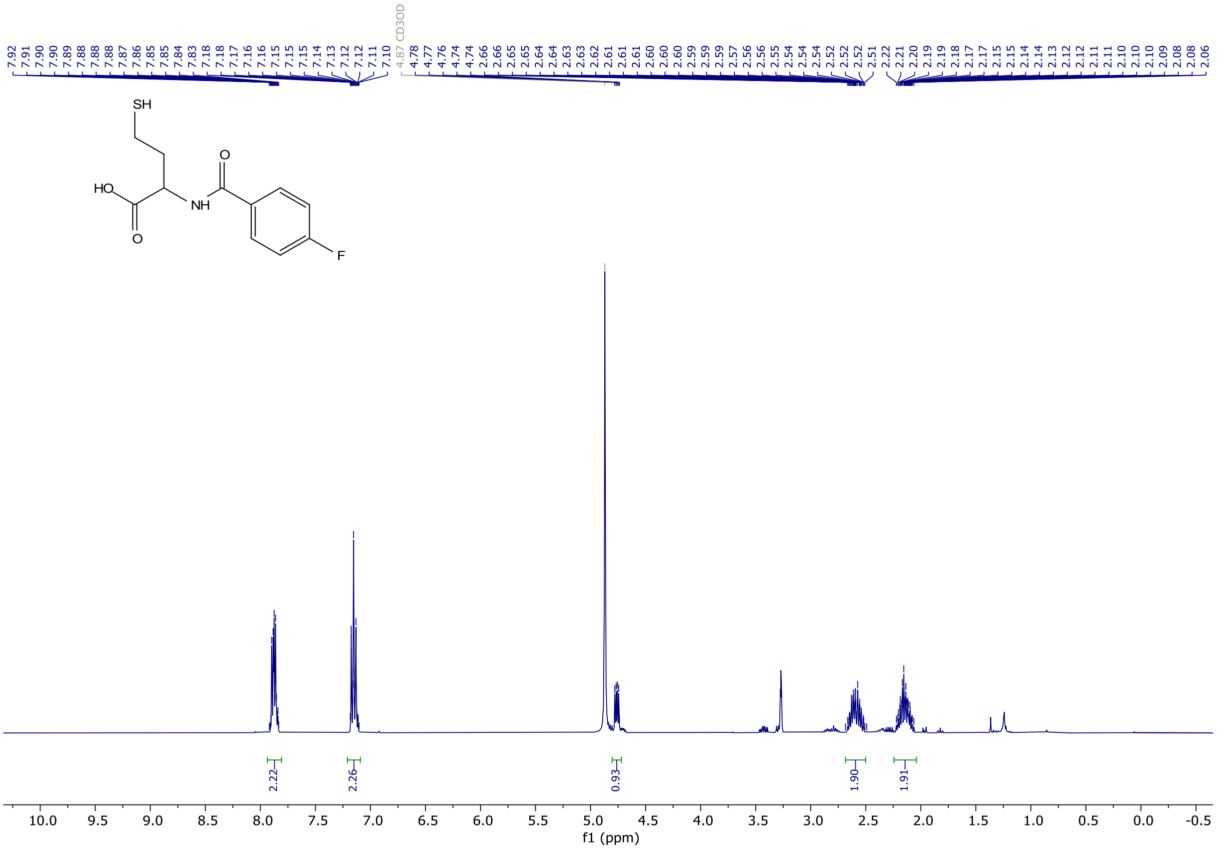

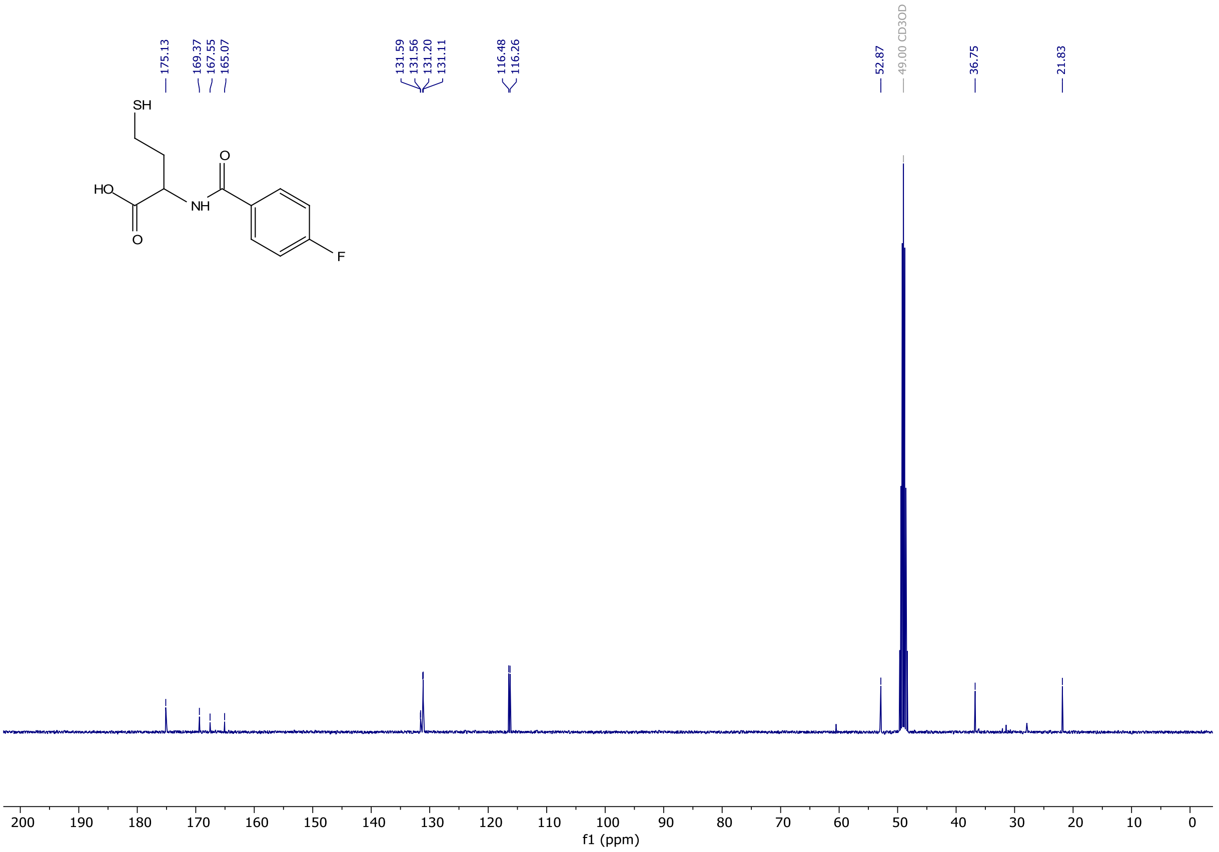

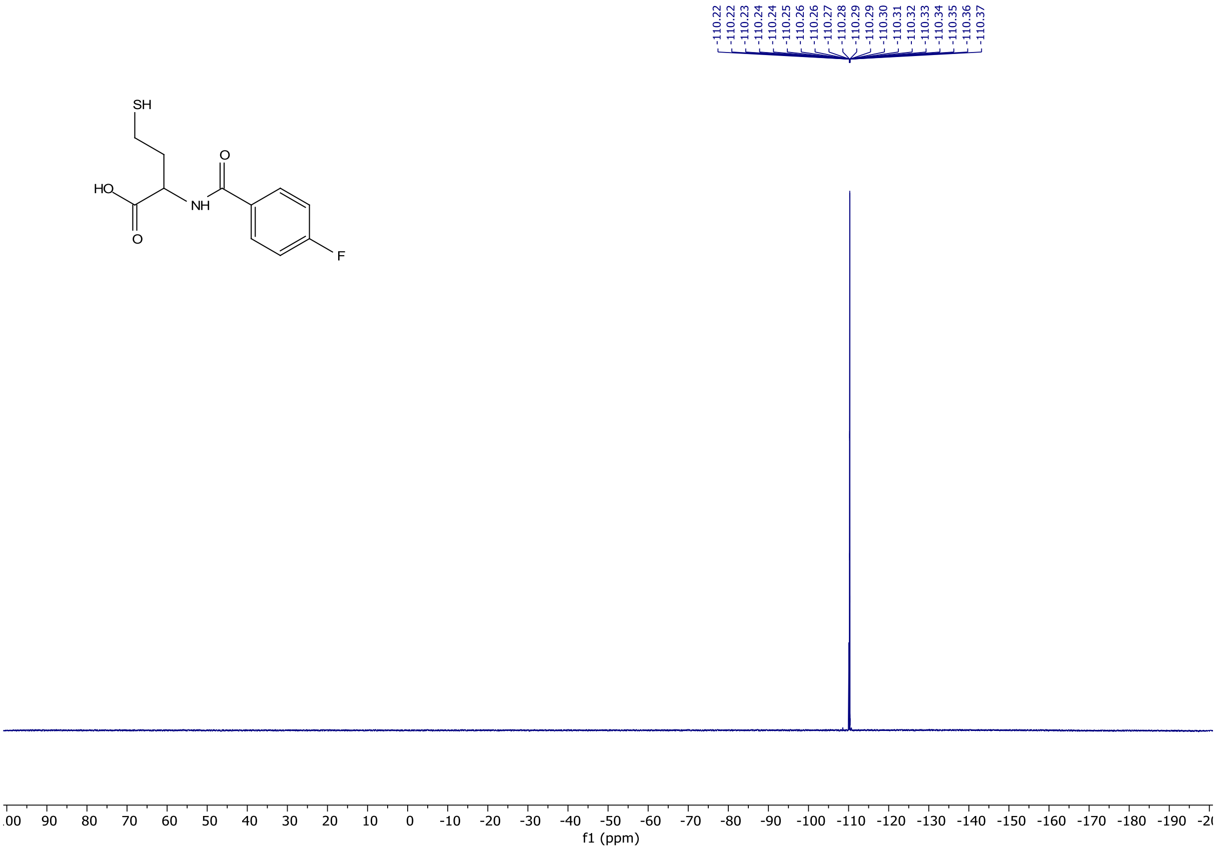
SH
O
HO
NH
O
F
-110.22
-110.22
-110.23
-110.24
-110.24
-110.25
-110.26
-110.26
-110.27
-110.28
-110.29
-110.29
-110.30
-110.31
-110.32
-110.33
-110.34
-110.35
-110.36
-110.37
f1 (ppm)

(4-Chlorobenzoyl)homocysteine 2j

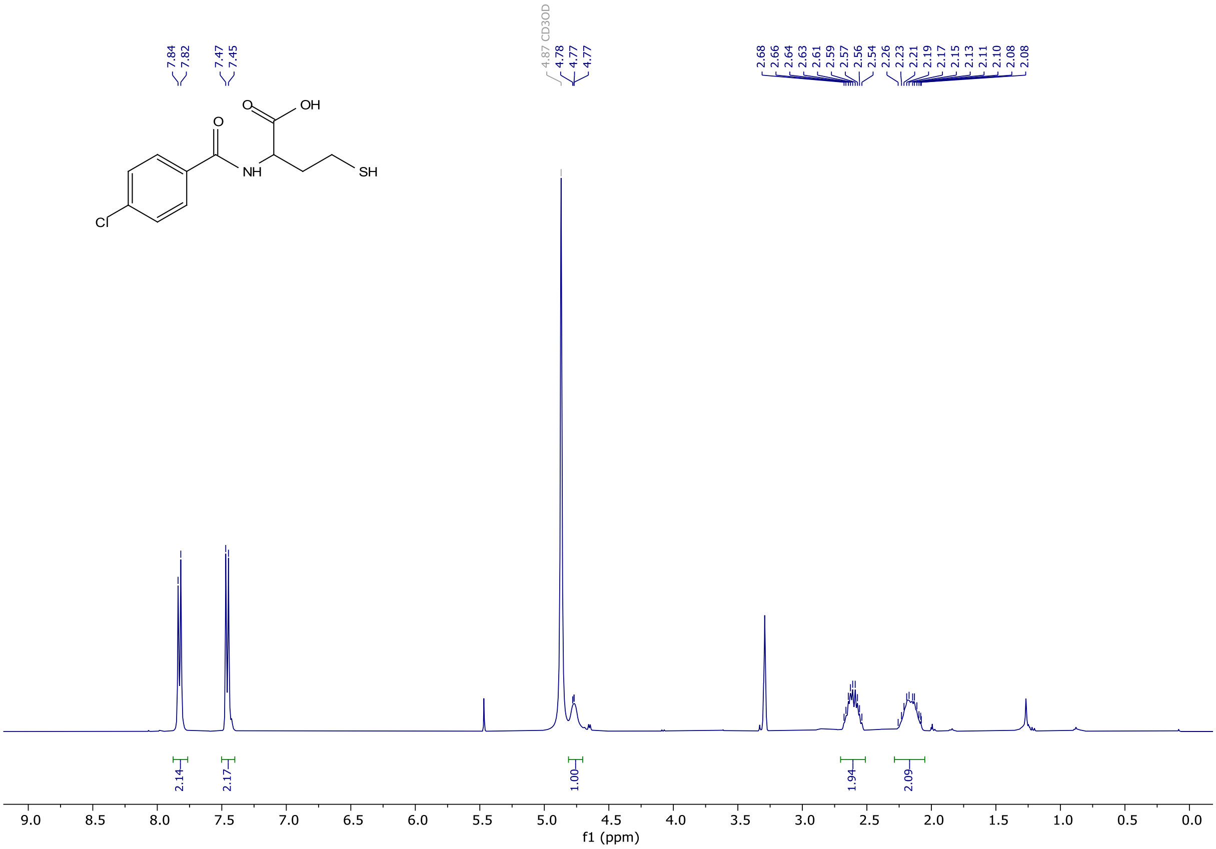

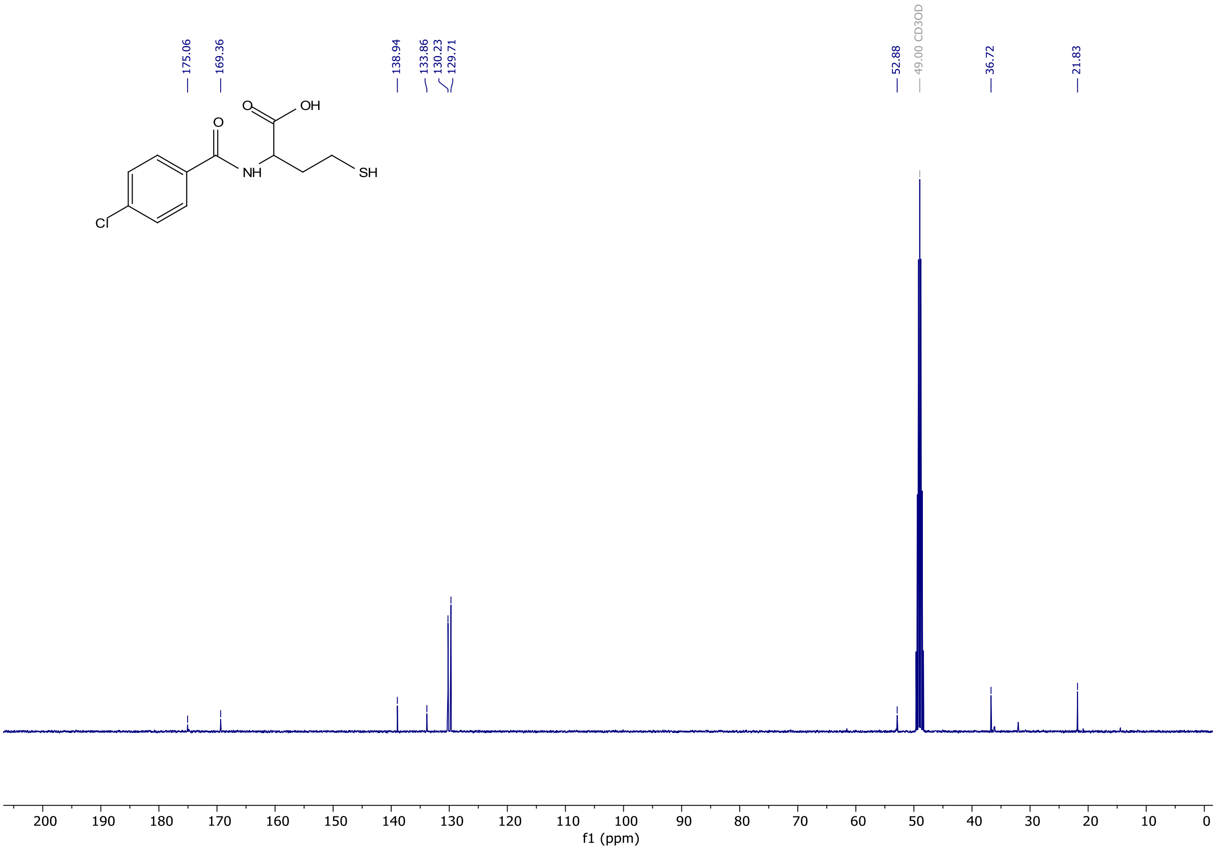

(2-Chlorobenzoyl)homocysteine 2k

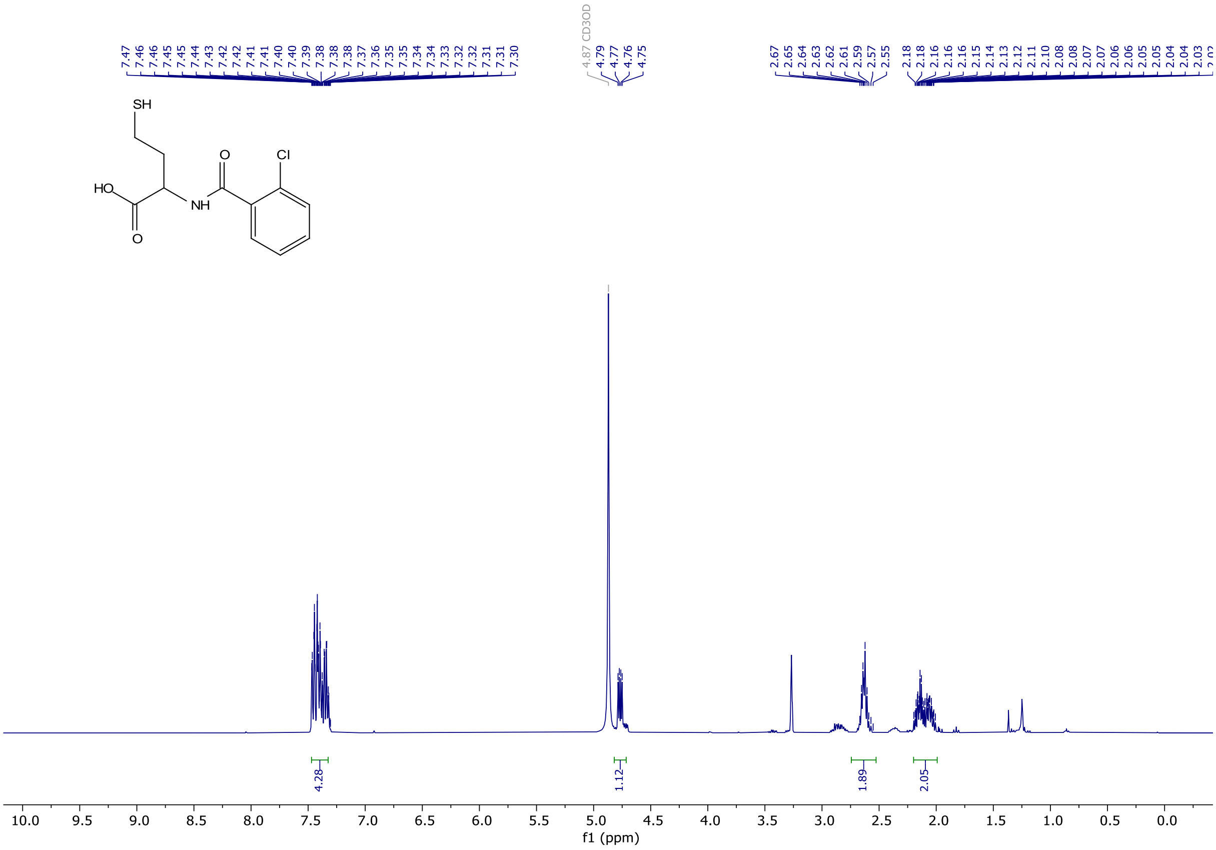

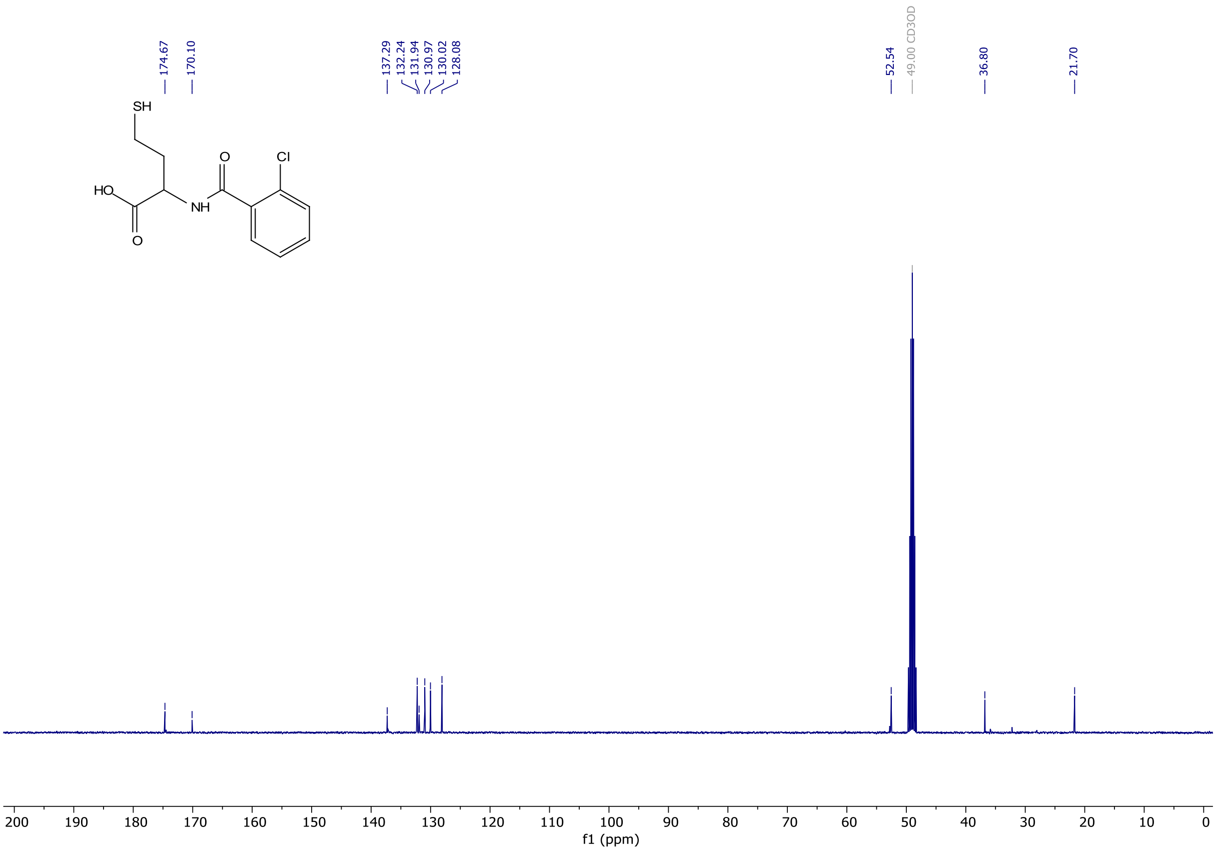

(4-Methylbenzoyl)homocysteine 2l

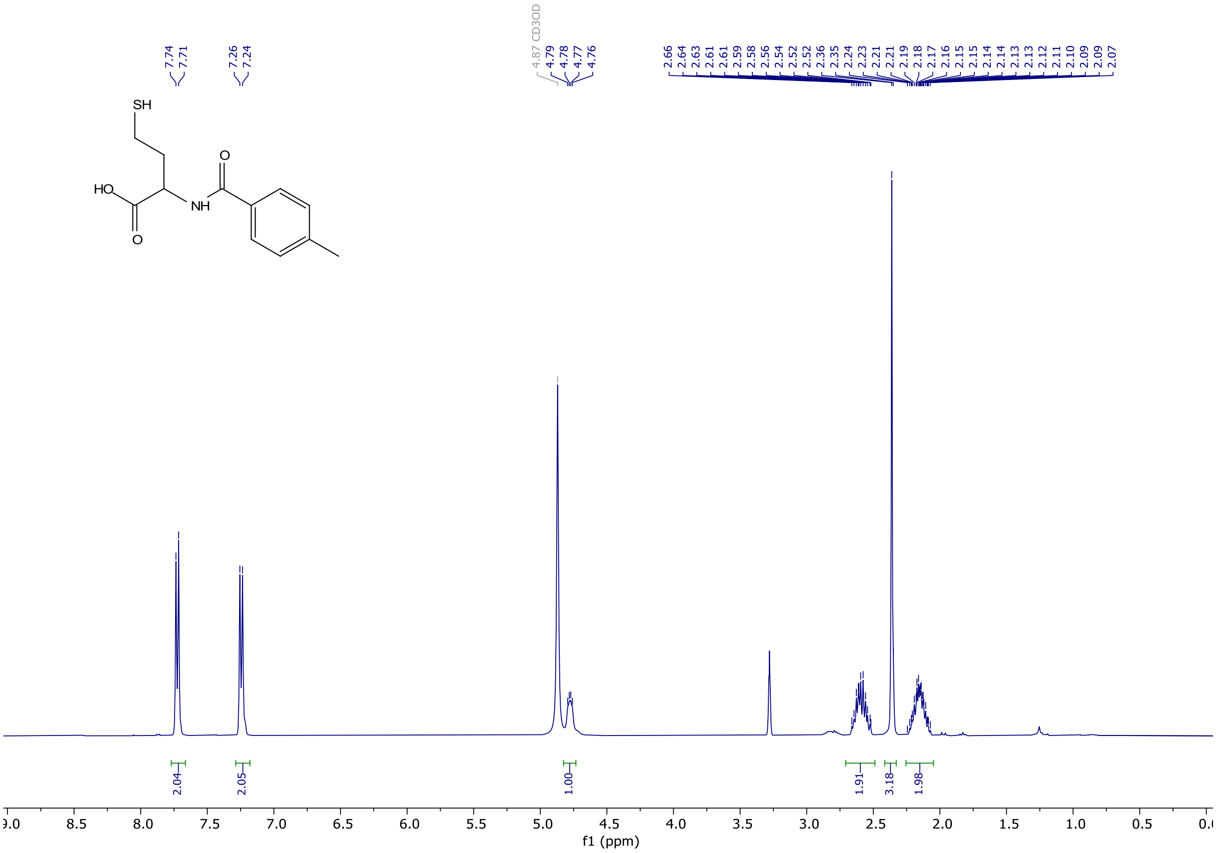

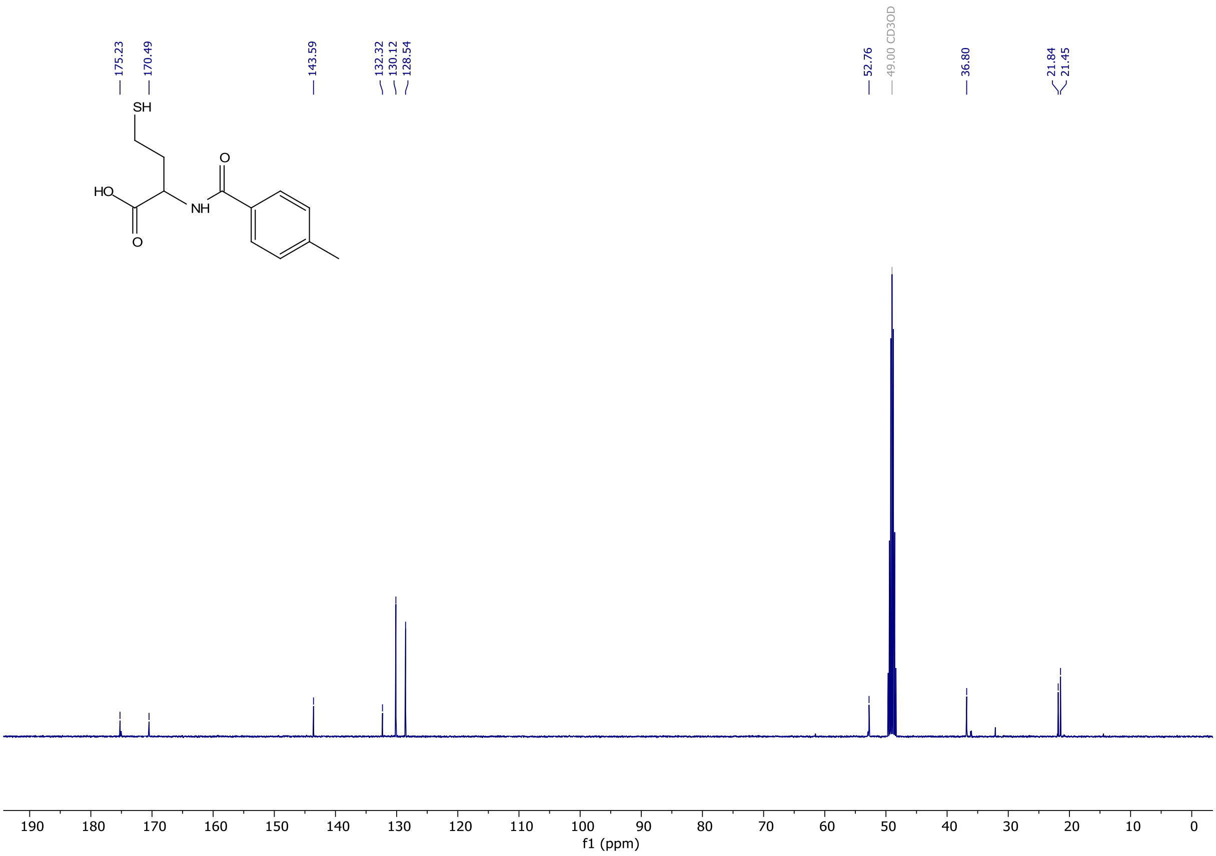

(4-Methoxybenzoyl)homocysteine 2m

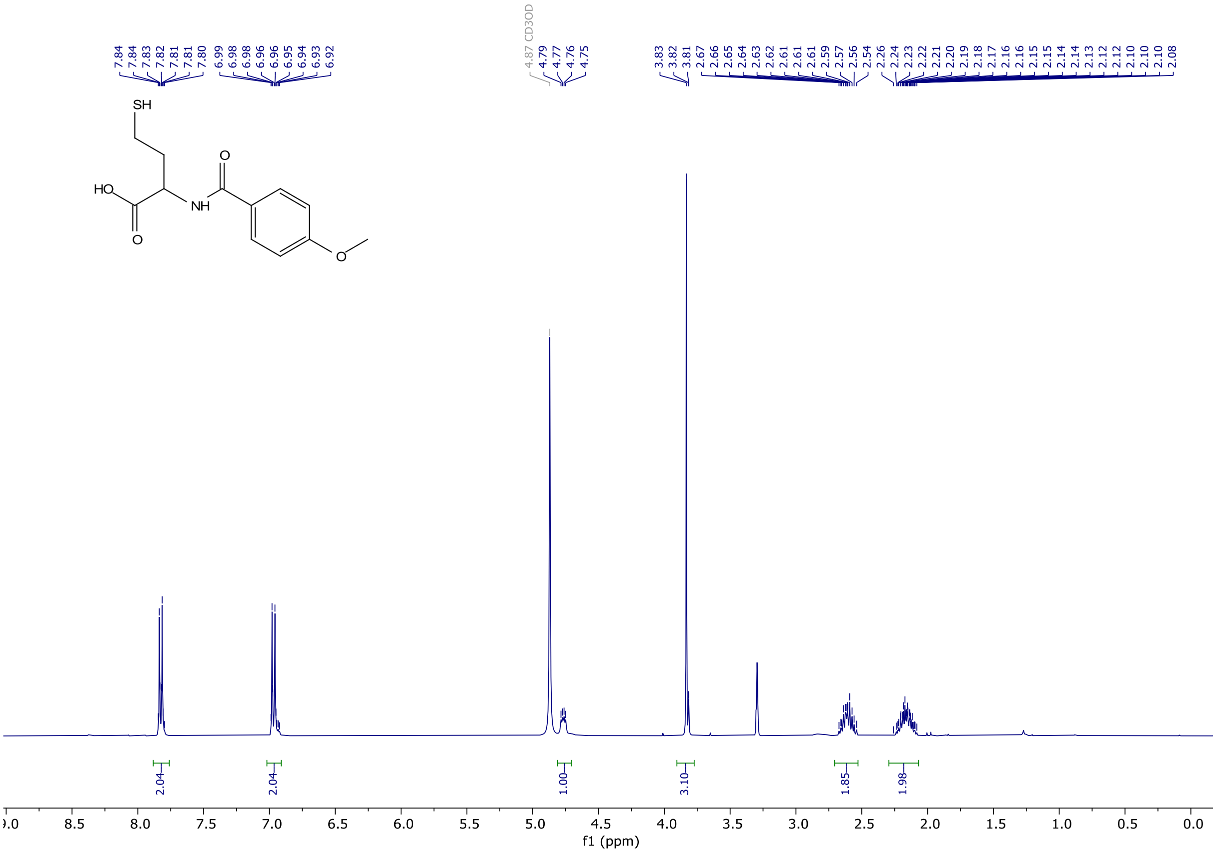

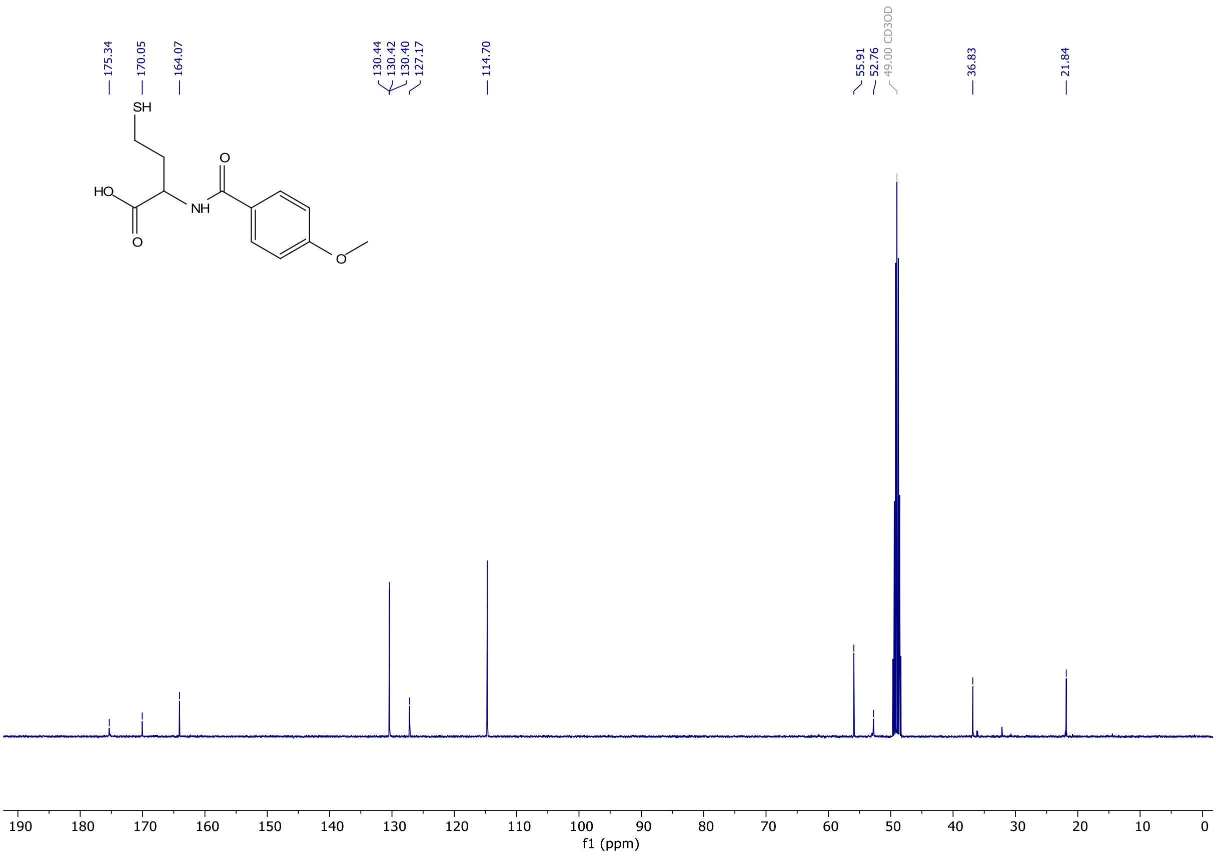

N-(2-Oxotetrahydrofuran-3-yl)benzamide 3a

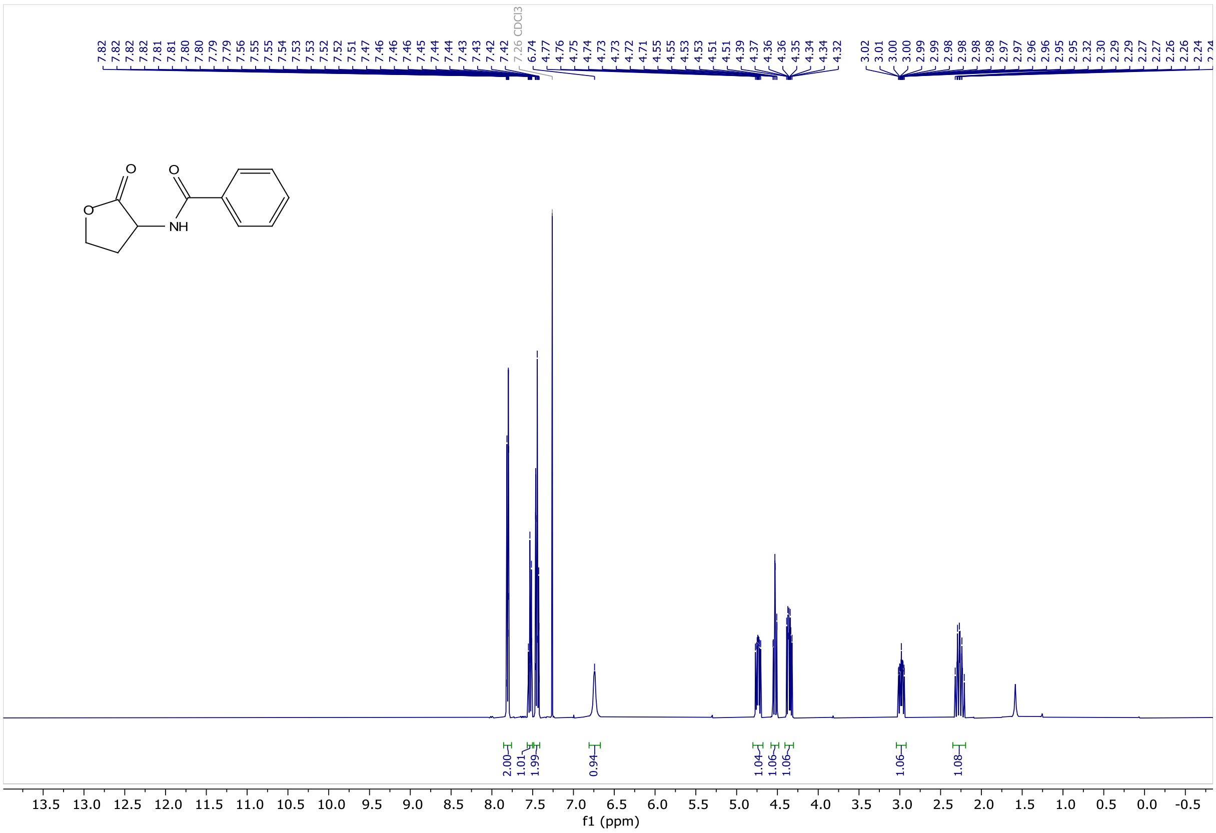

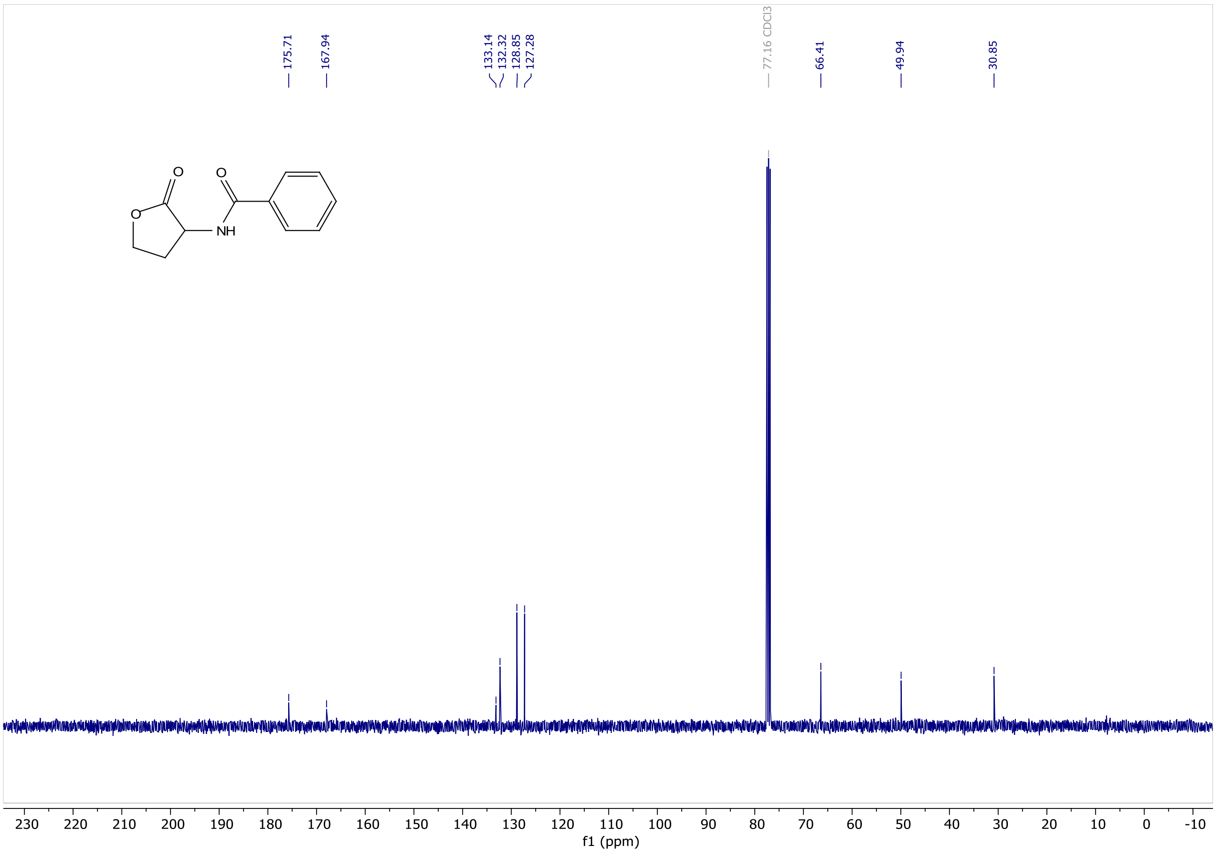

N-(2-Oxotetrahydrofuran-3-yl)hexanamide 3b

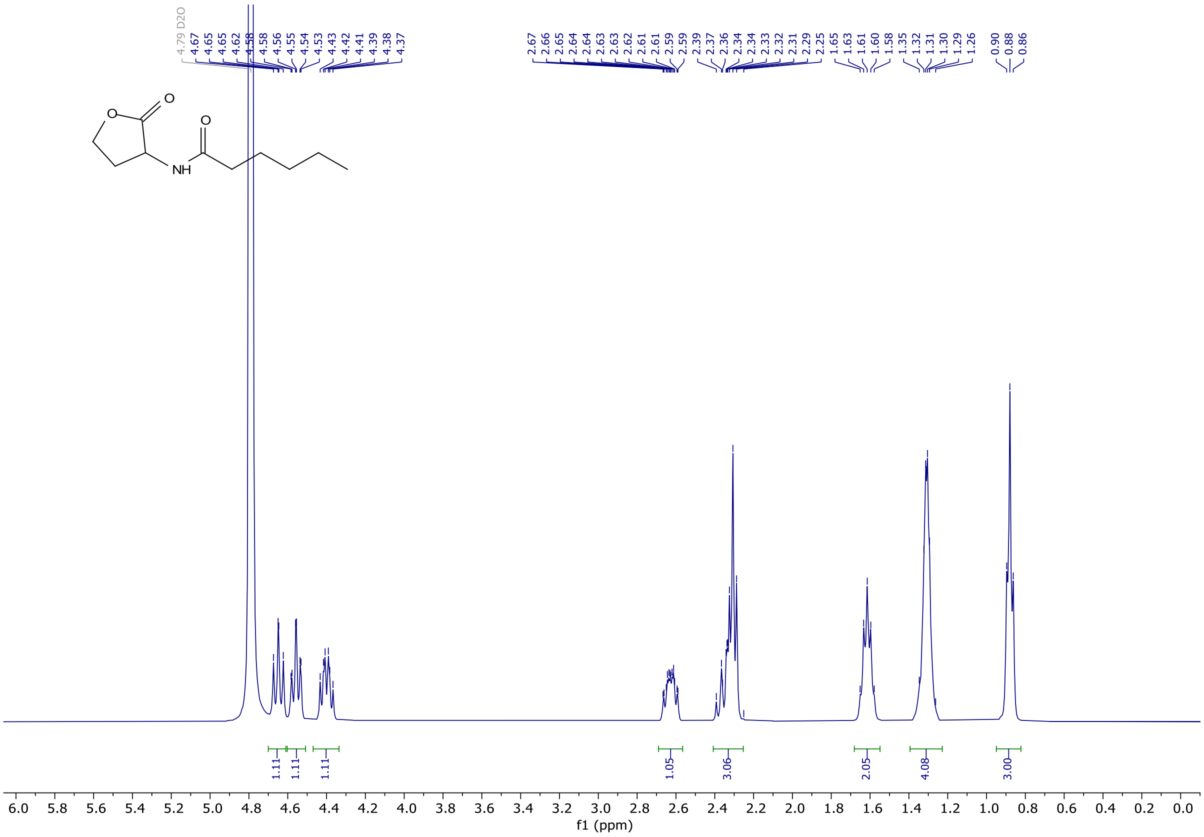

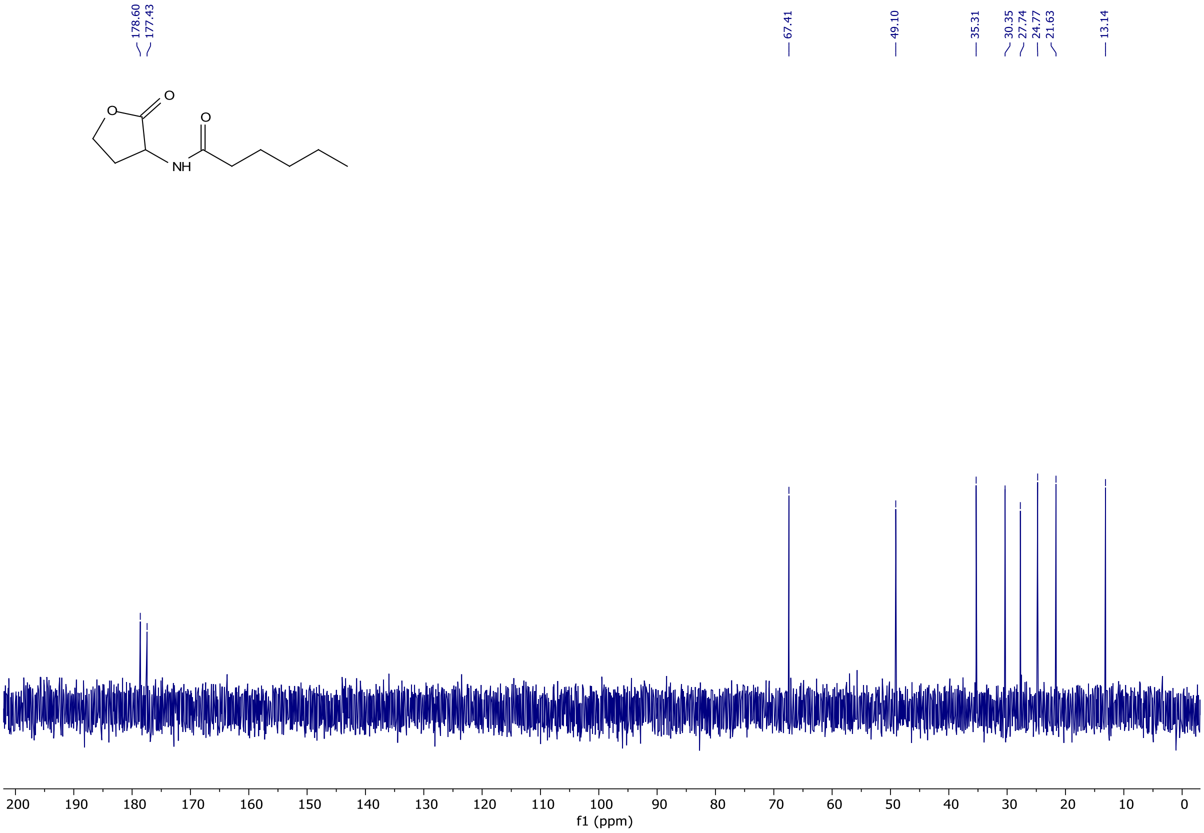

Benzoylhomoserine 4a

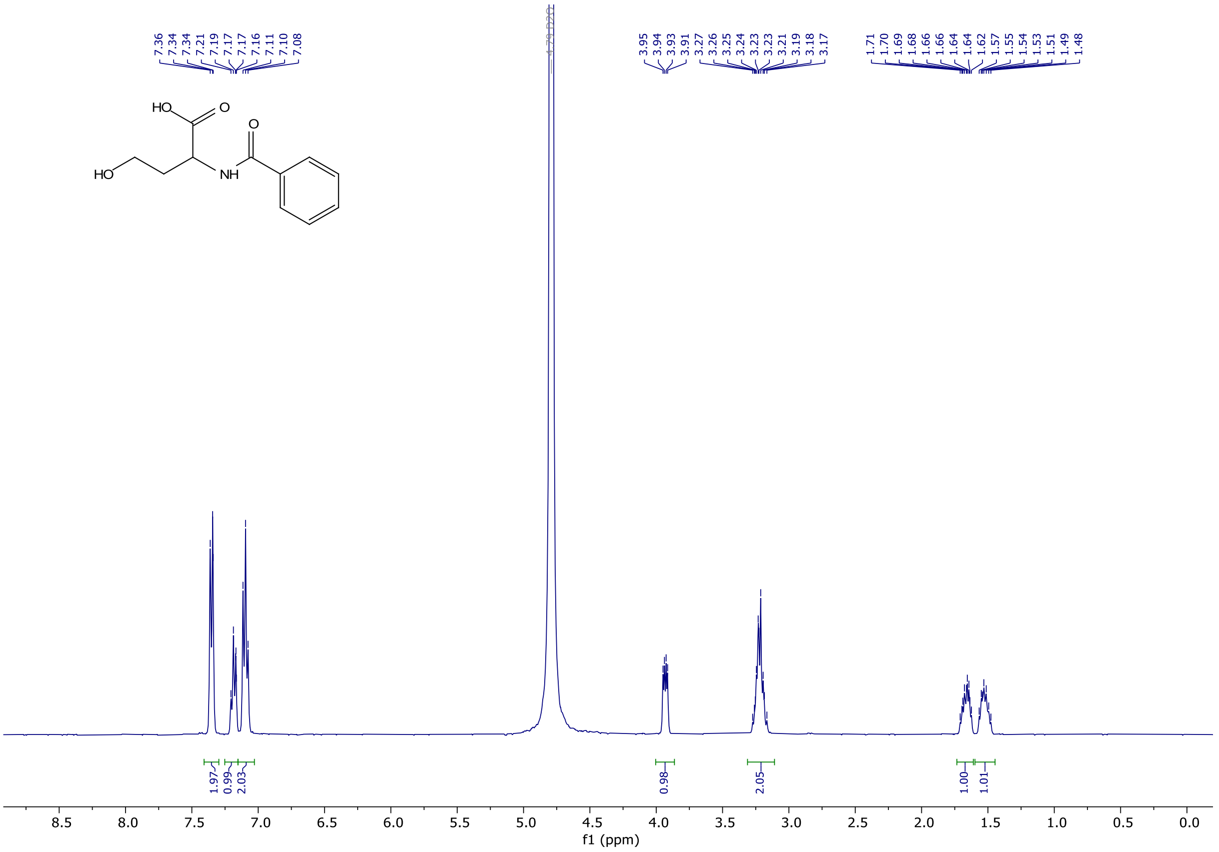

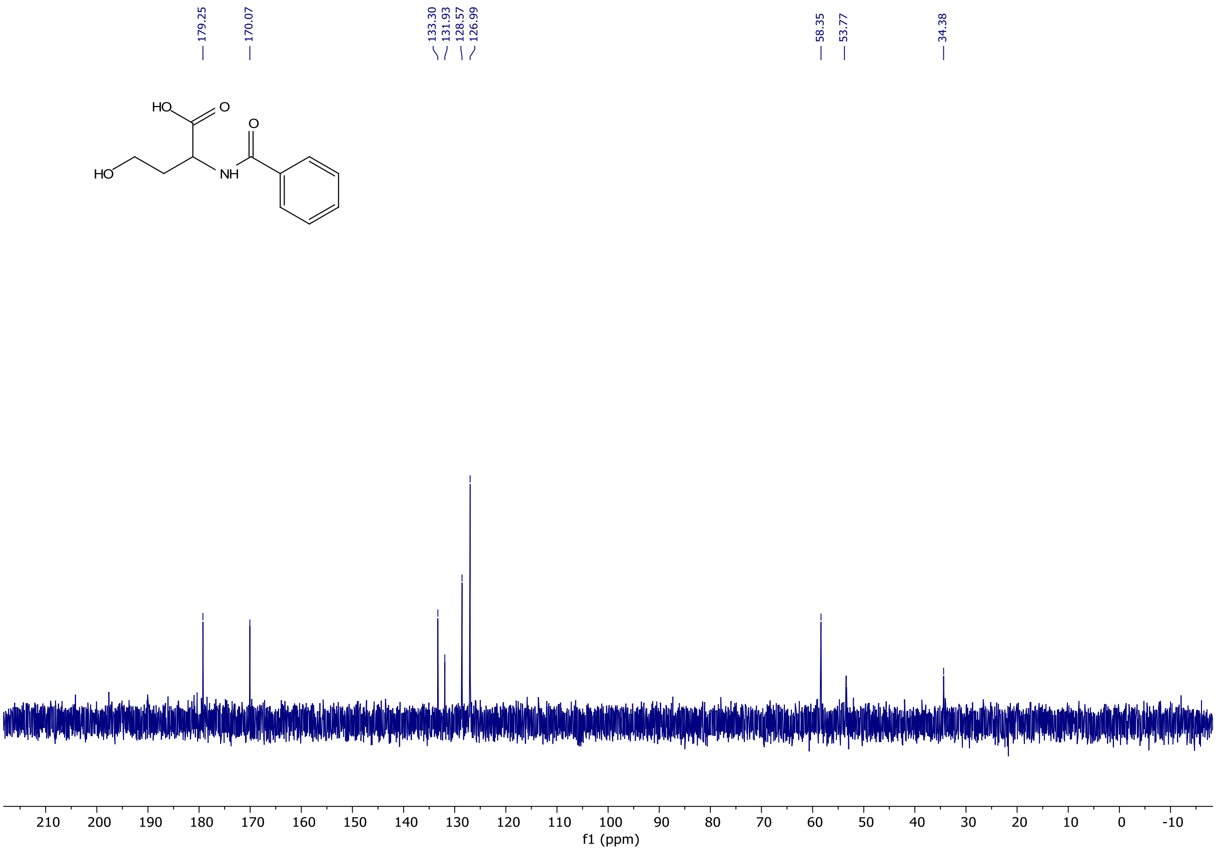

Hexanoylhomoserine 4b

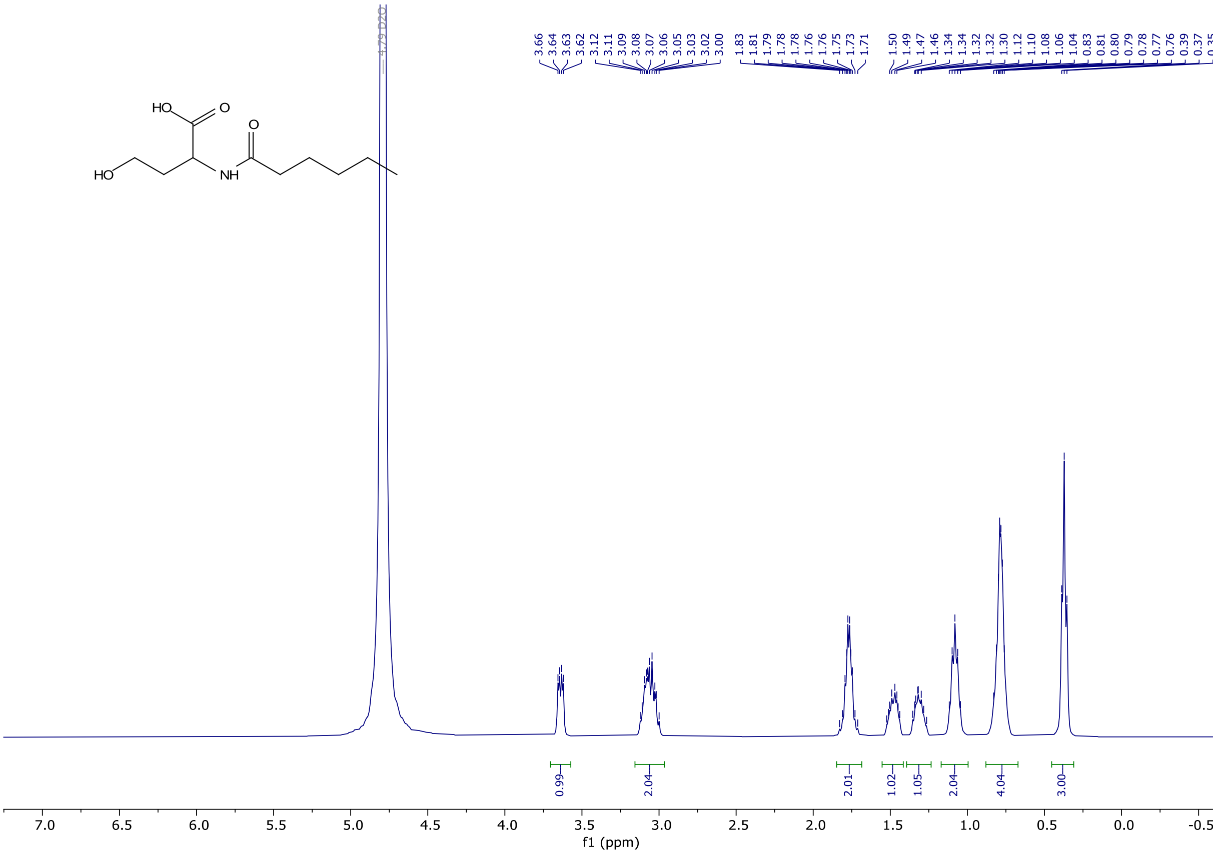

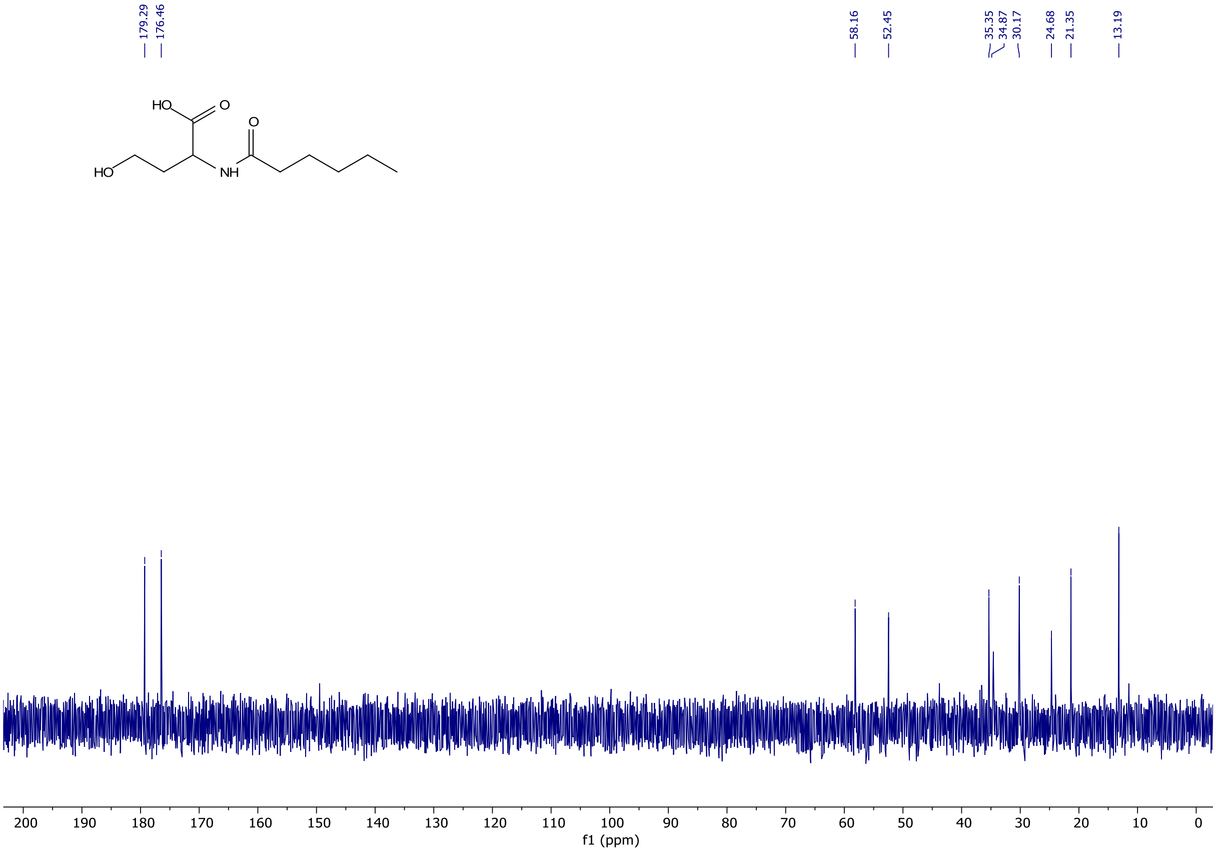

3-(Phenylthio)dihydrothiophen-2(3H)-one 5a

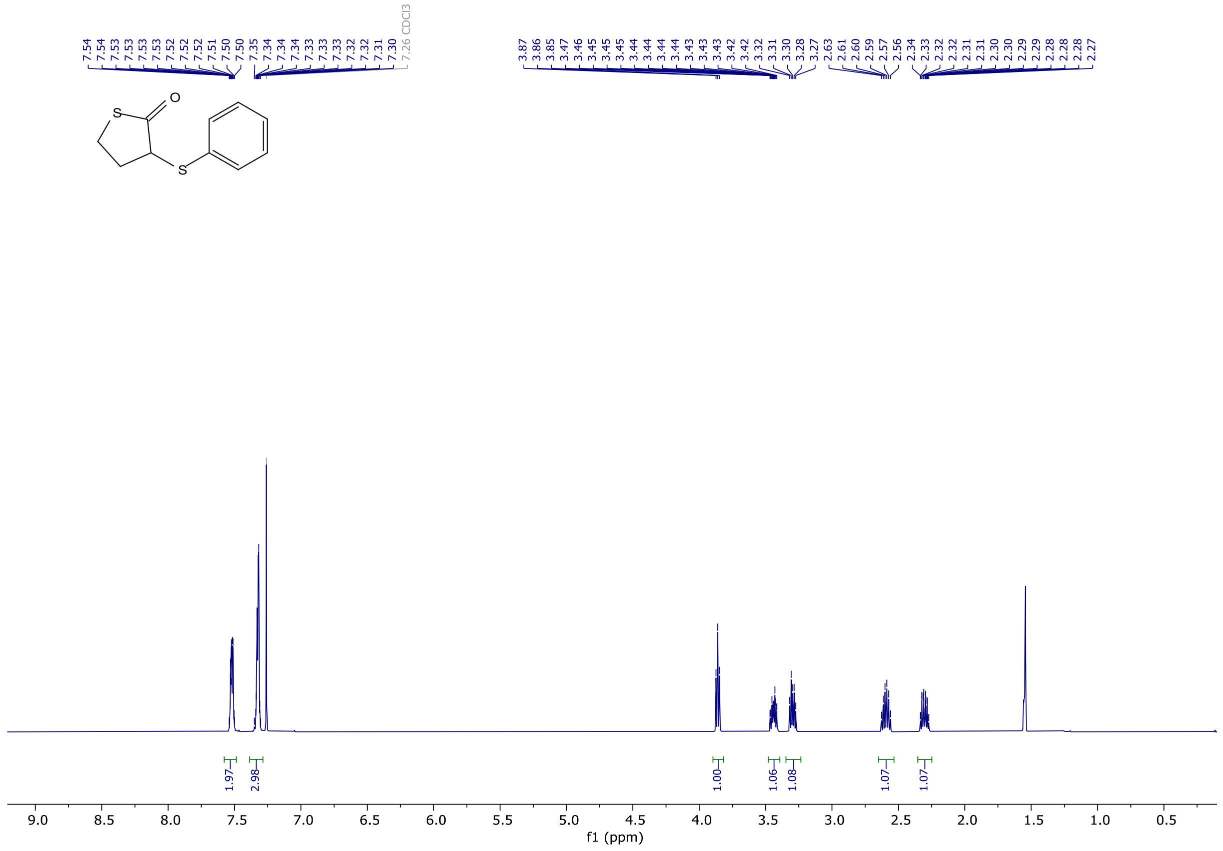

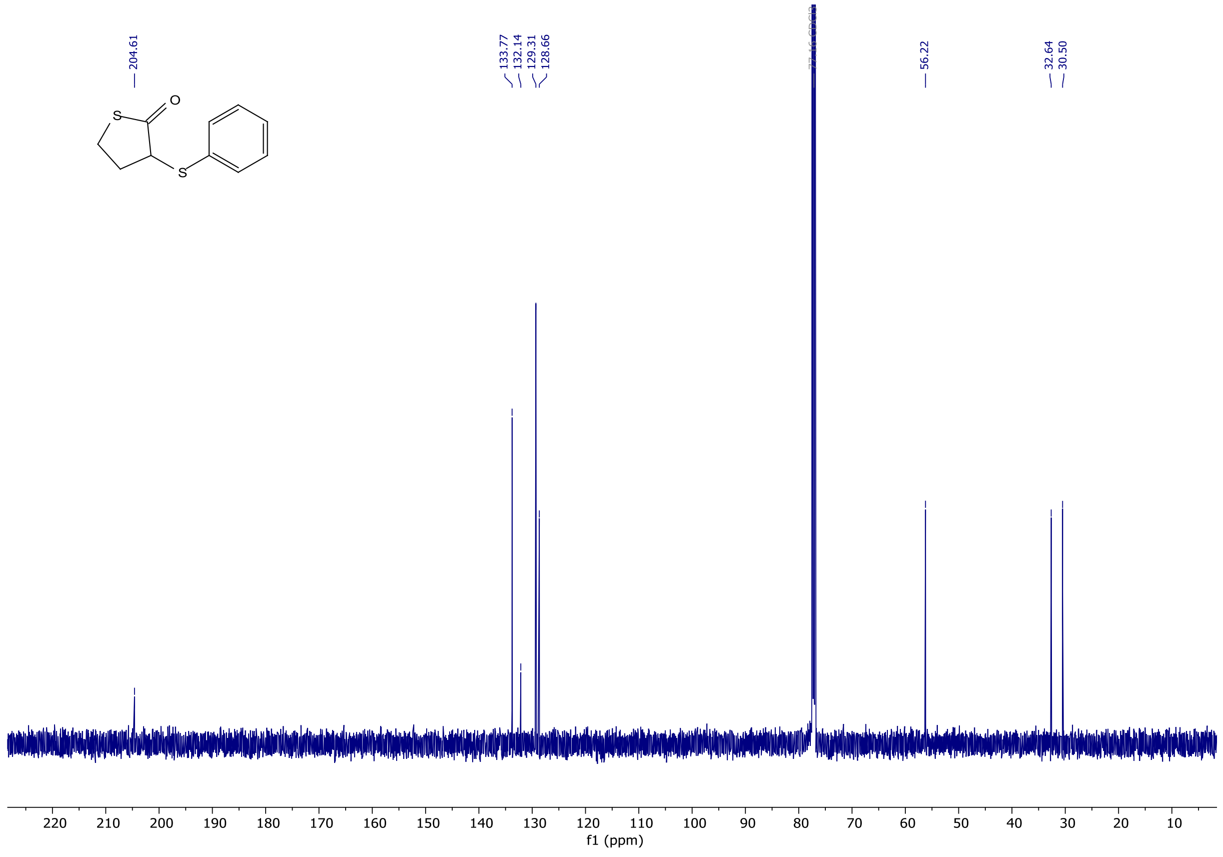

3-((4-Chlorophenyl)thio)dihydrothiophen-2(3H)-one 5b

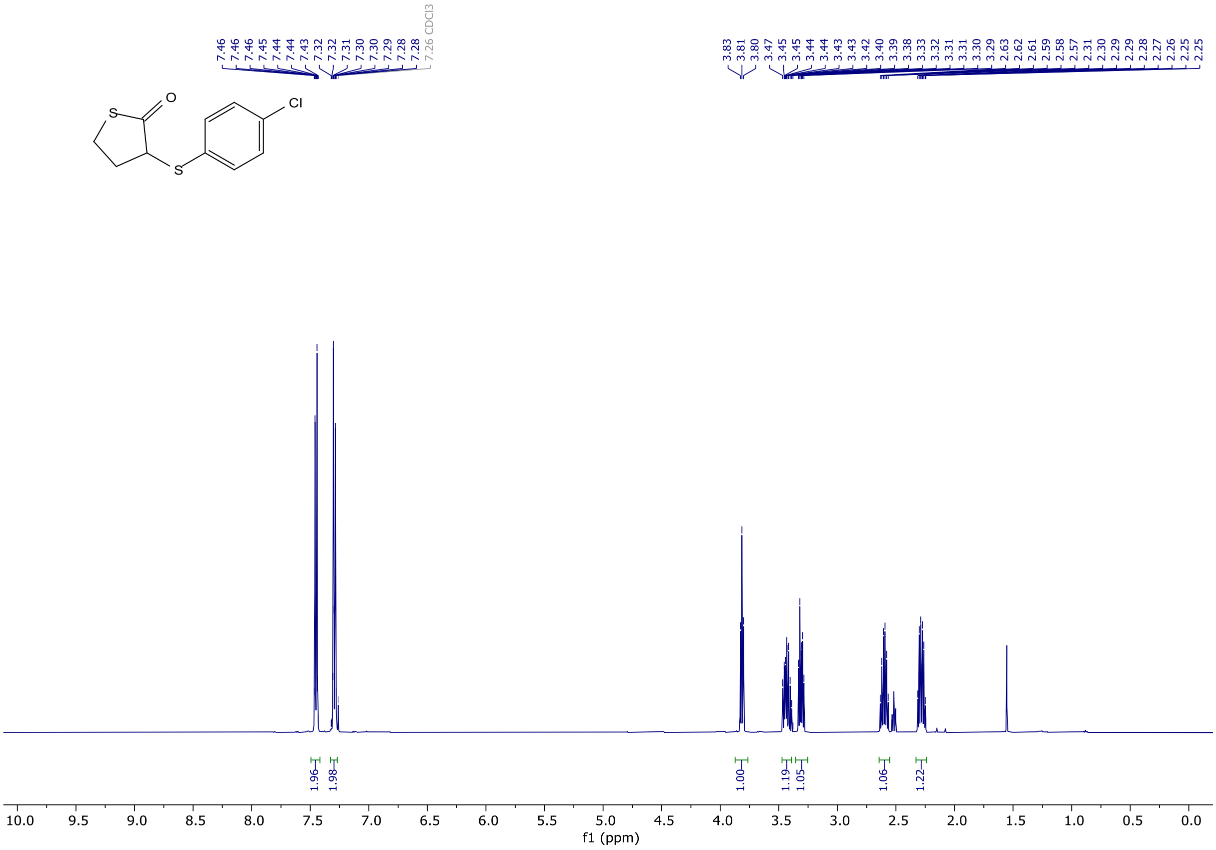

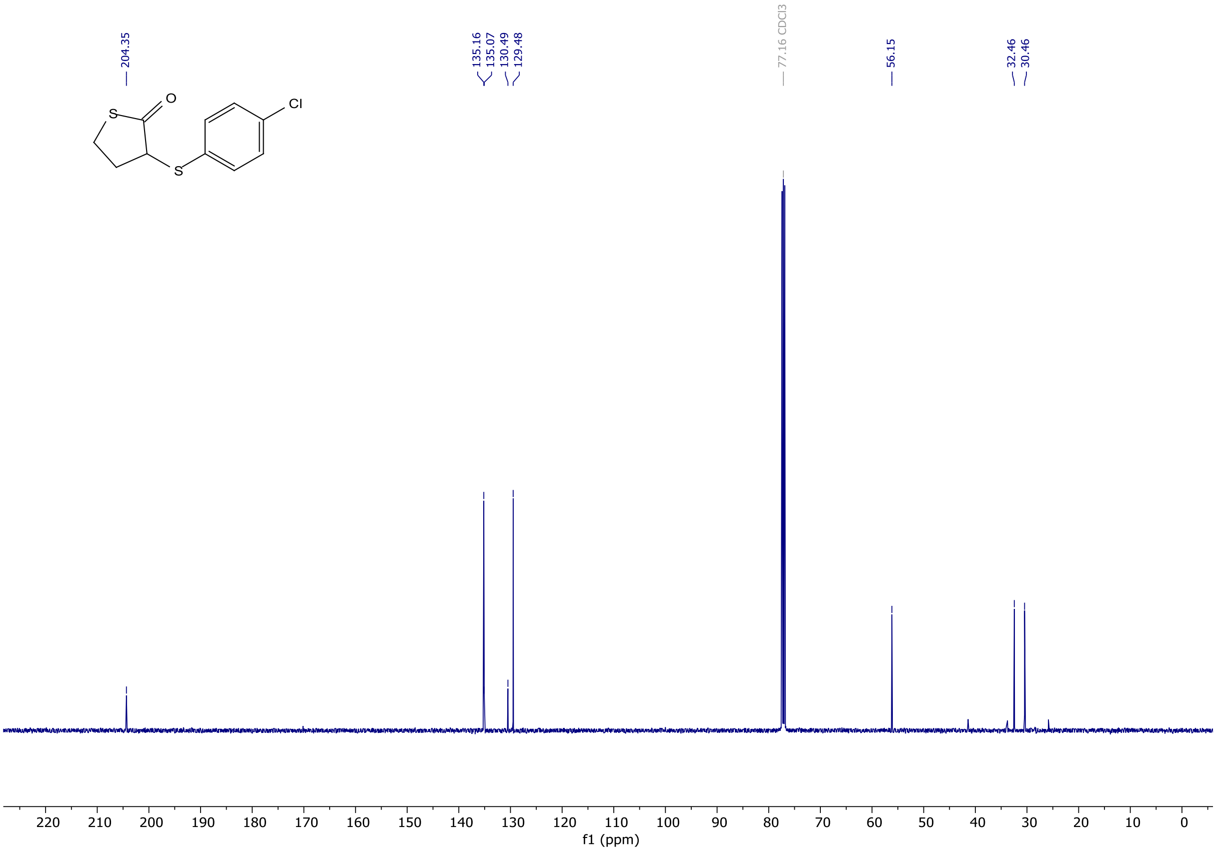

3-((4-Bromophenyl)thio)dihydrothiophen-2(3H)-one 5c

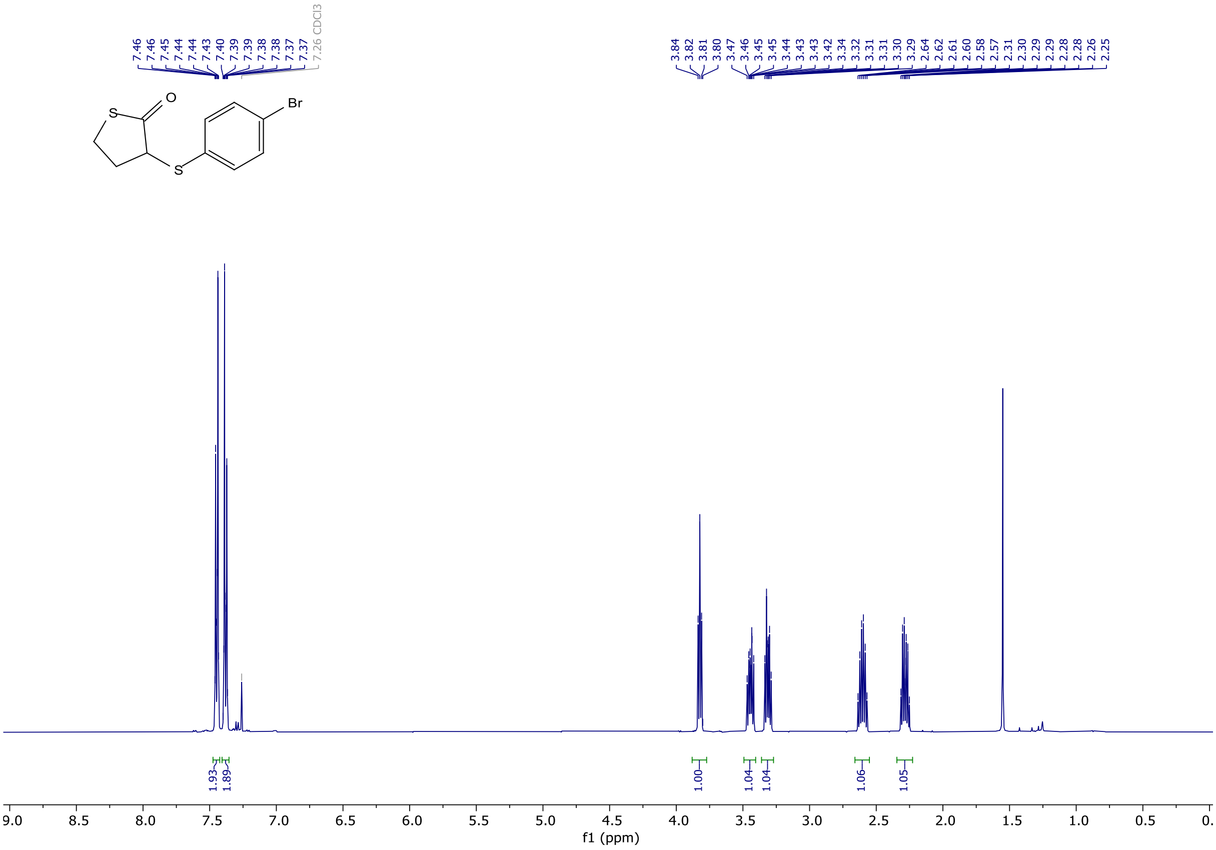

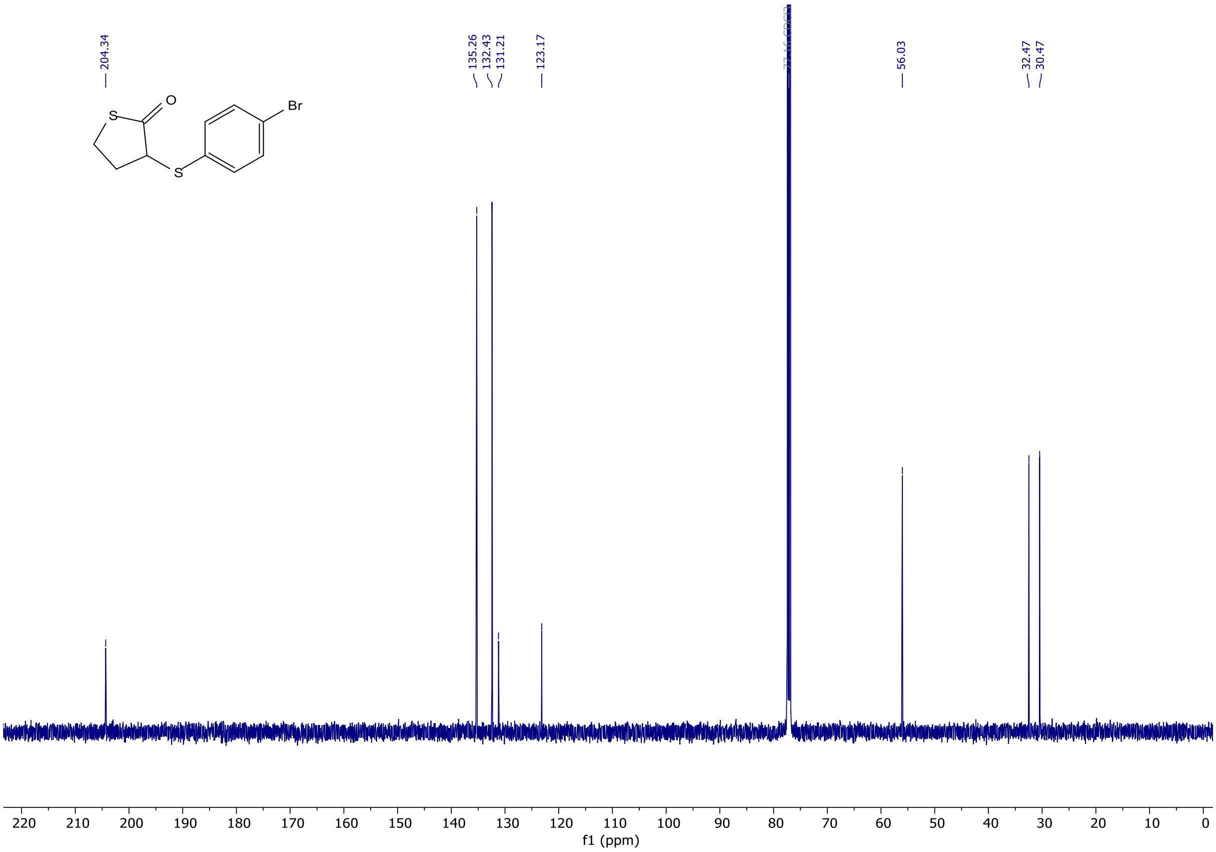

3-((4-Methoxyphenyl)thio)dihydrothiophen-2(3H)-one 5d

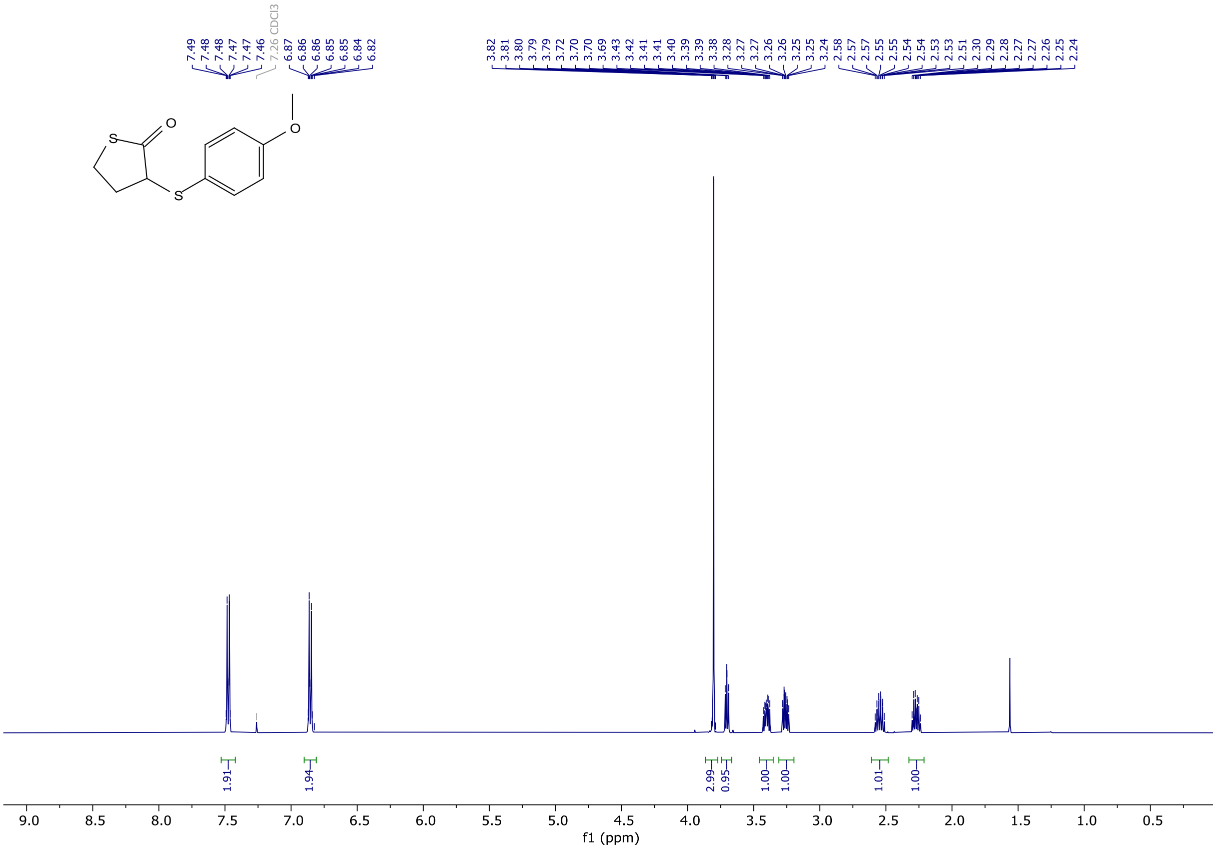

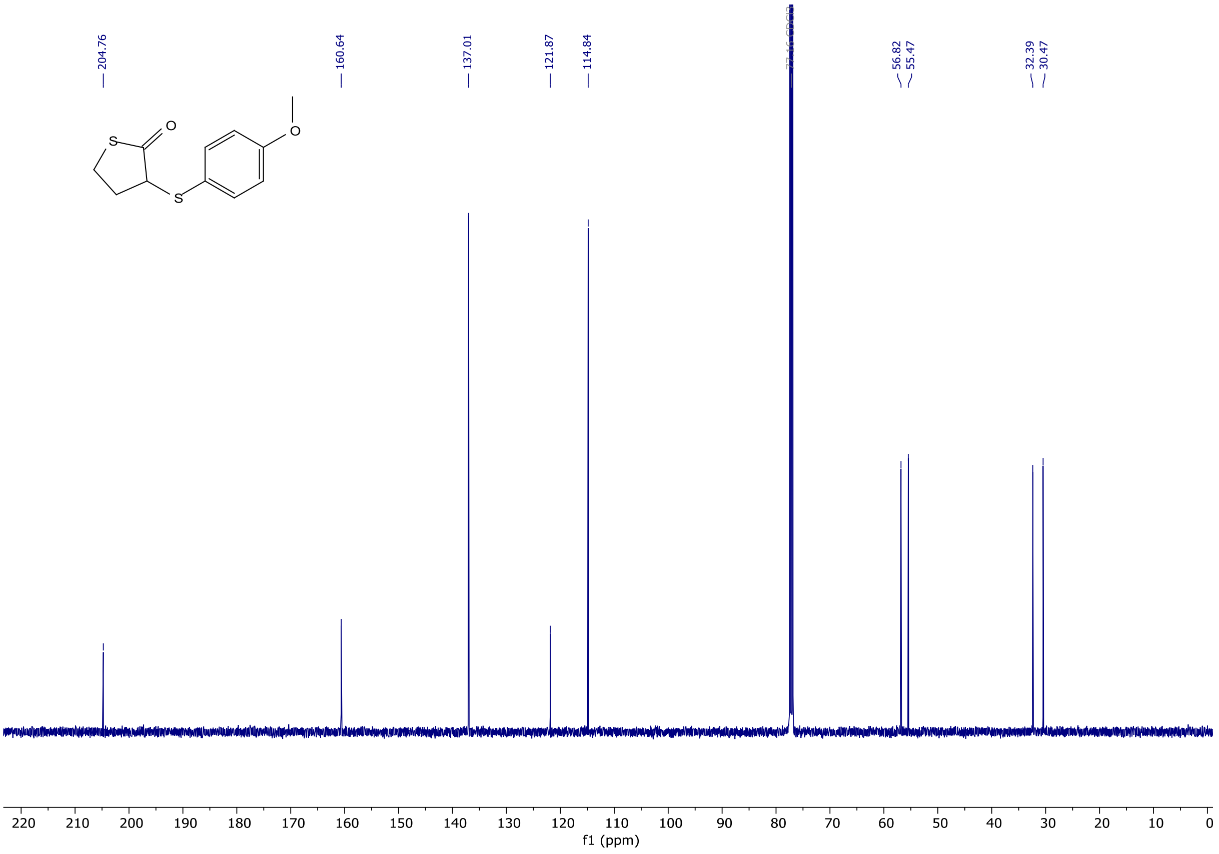

3-(*p*-Tolylthio)dihydrothiophen-2(3H)-one 5e

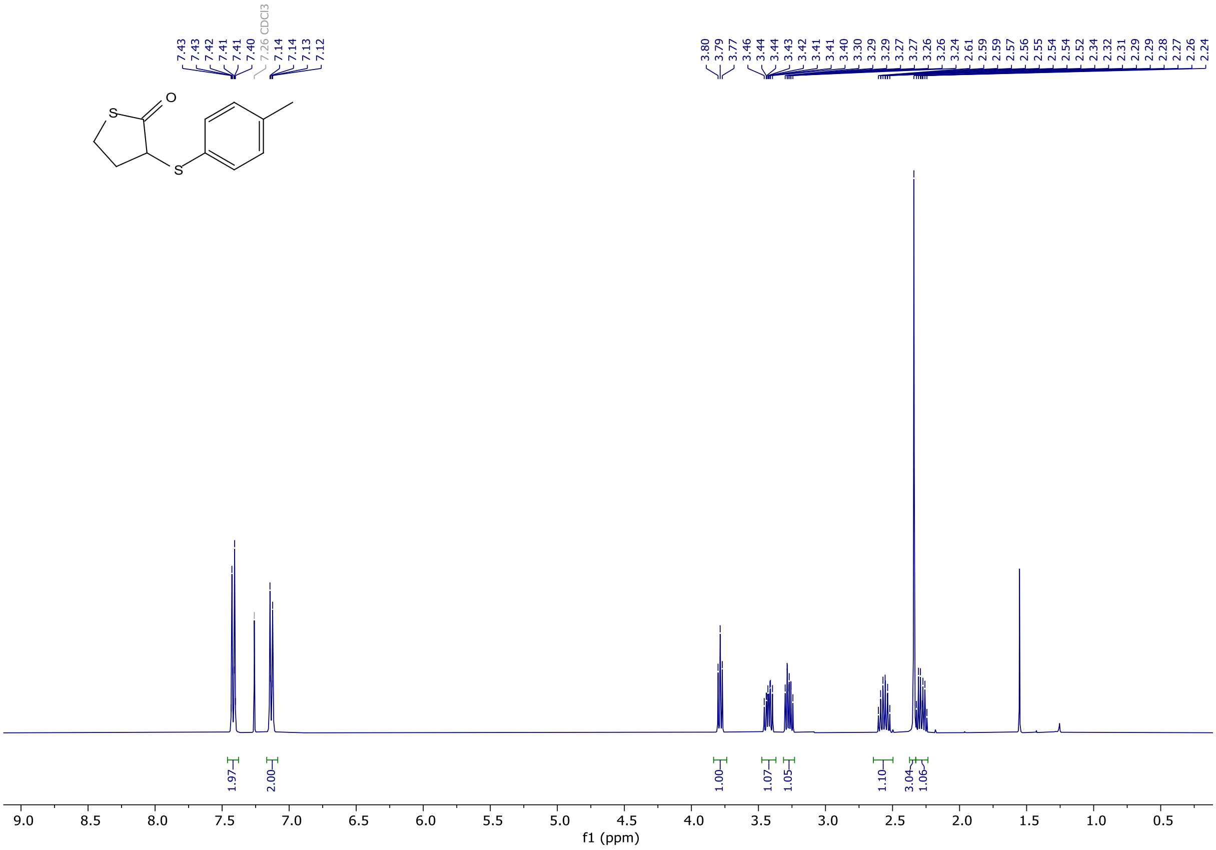

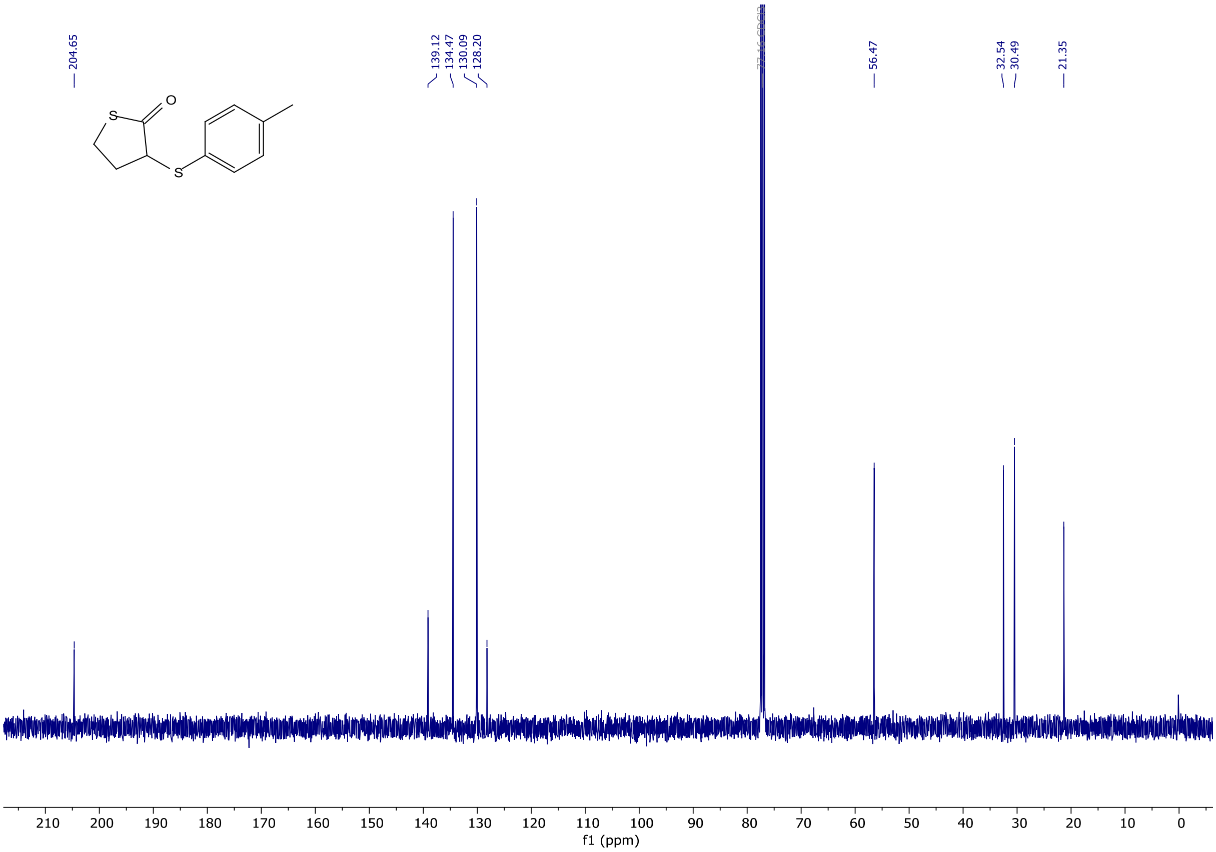

3-((2-Fluorophenyl)thio)dihydrothiophen-2(3H)-one 5f

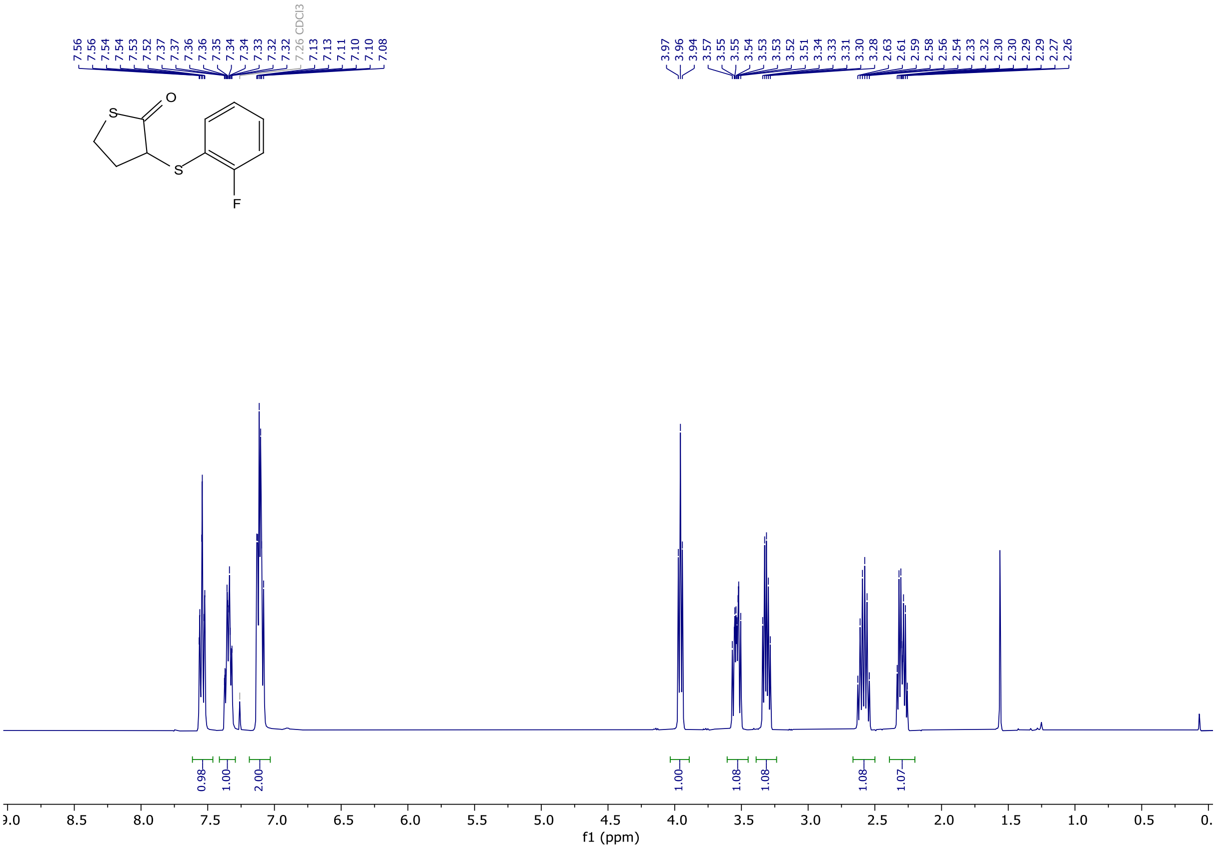

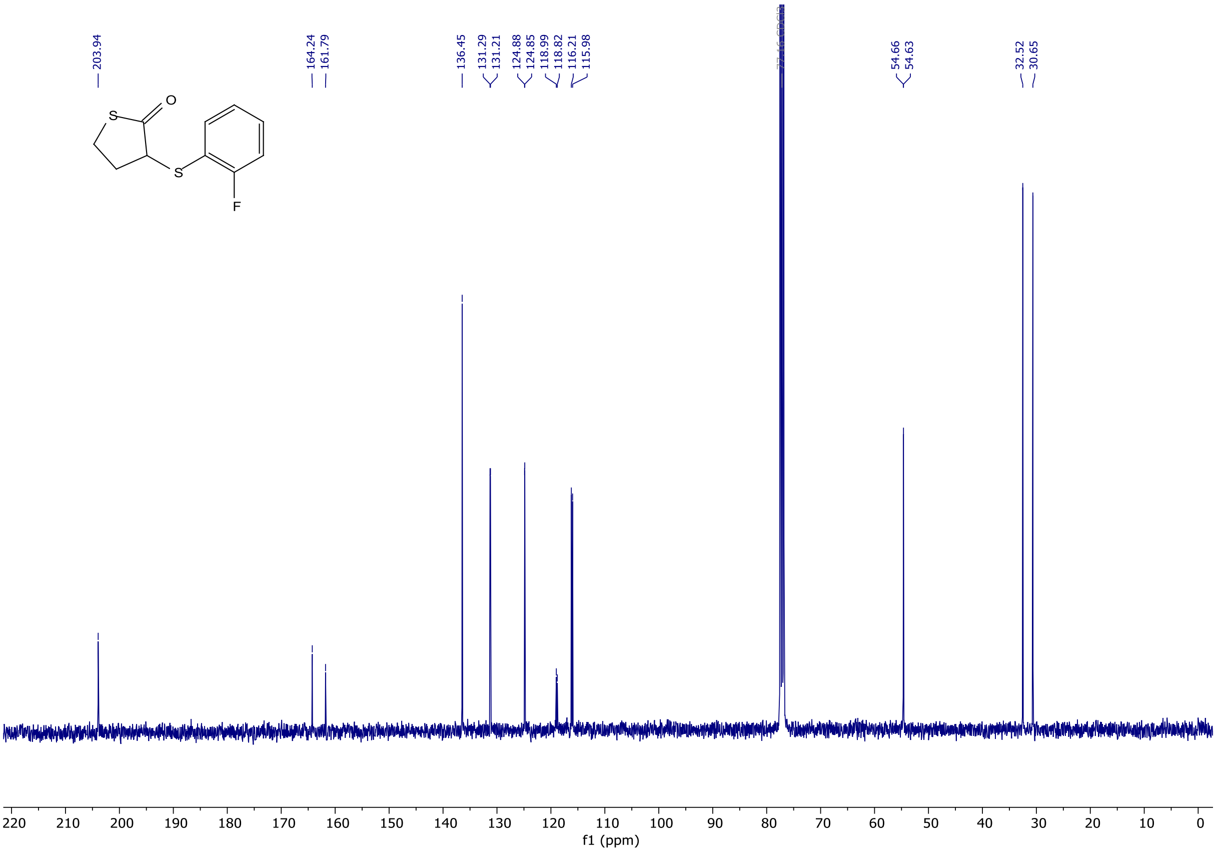

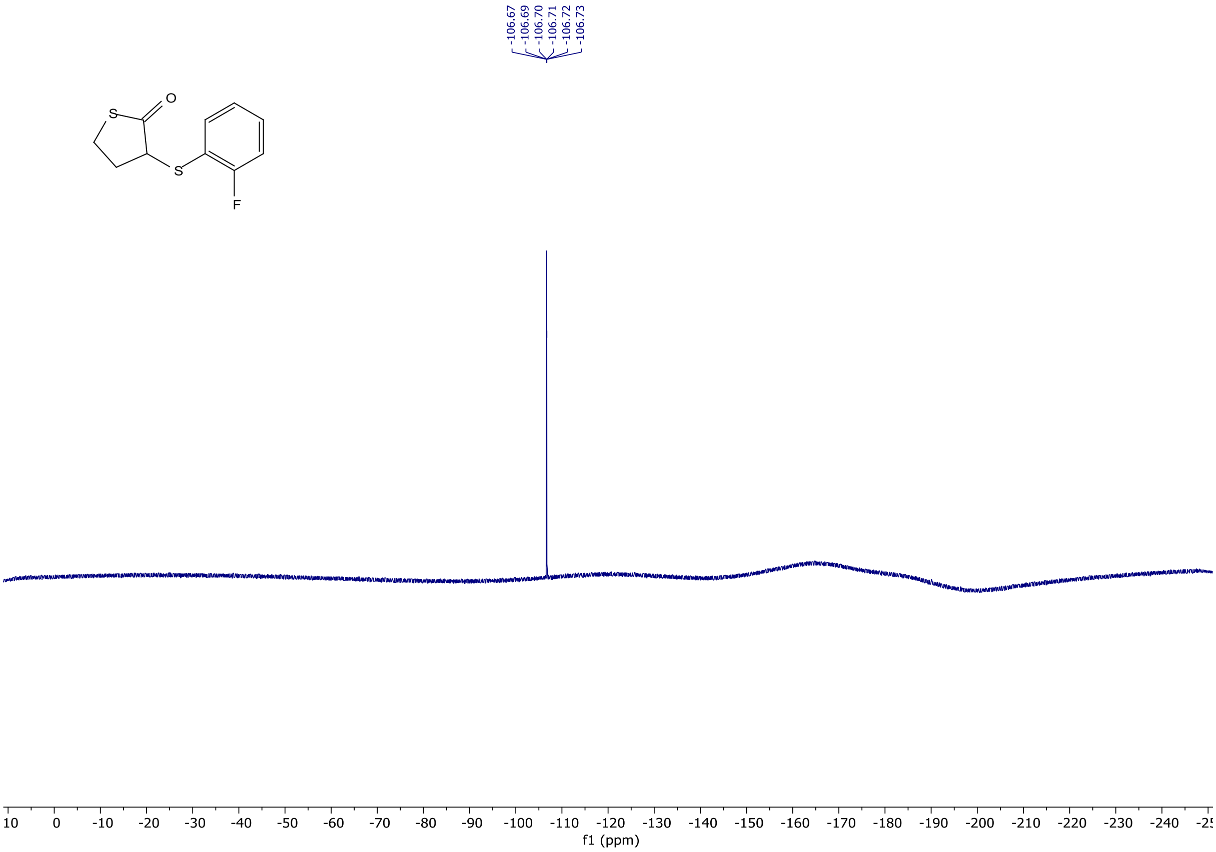

-106.67
-106.69
-106.70
-106.71
-106.72
-106.73
S
O
S
F
10
0
-10
-20
-30
-40
-50
-60
-70
-80
-90
-100
-110
-120
-130
-140
-150
-160
-170
-180
-190
-200
-210
-220
-230
-240
f1 (ppm)

3-((2-Chlorophenyl)thio)dihydrothiophen-2(3H)-one 5g

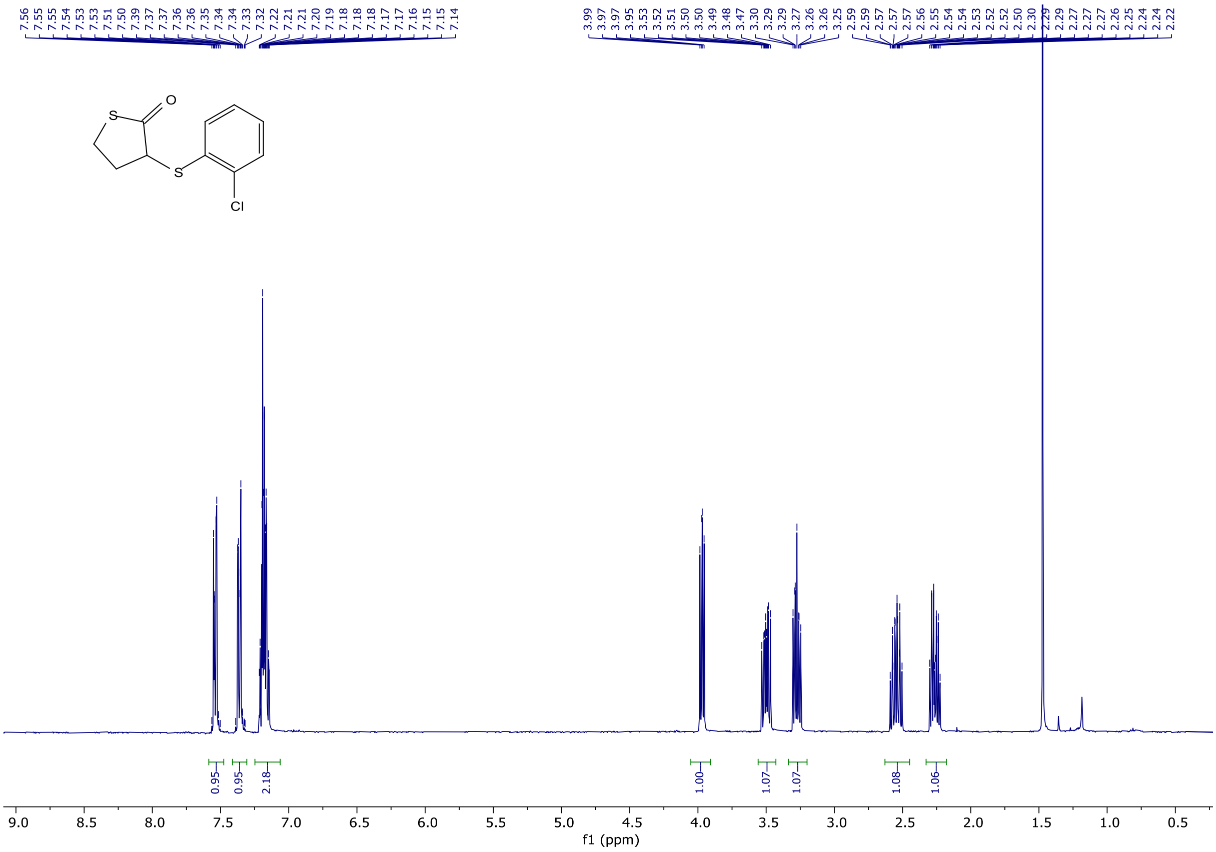

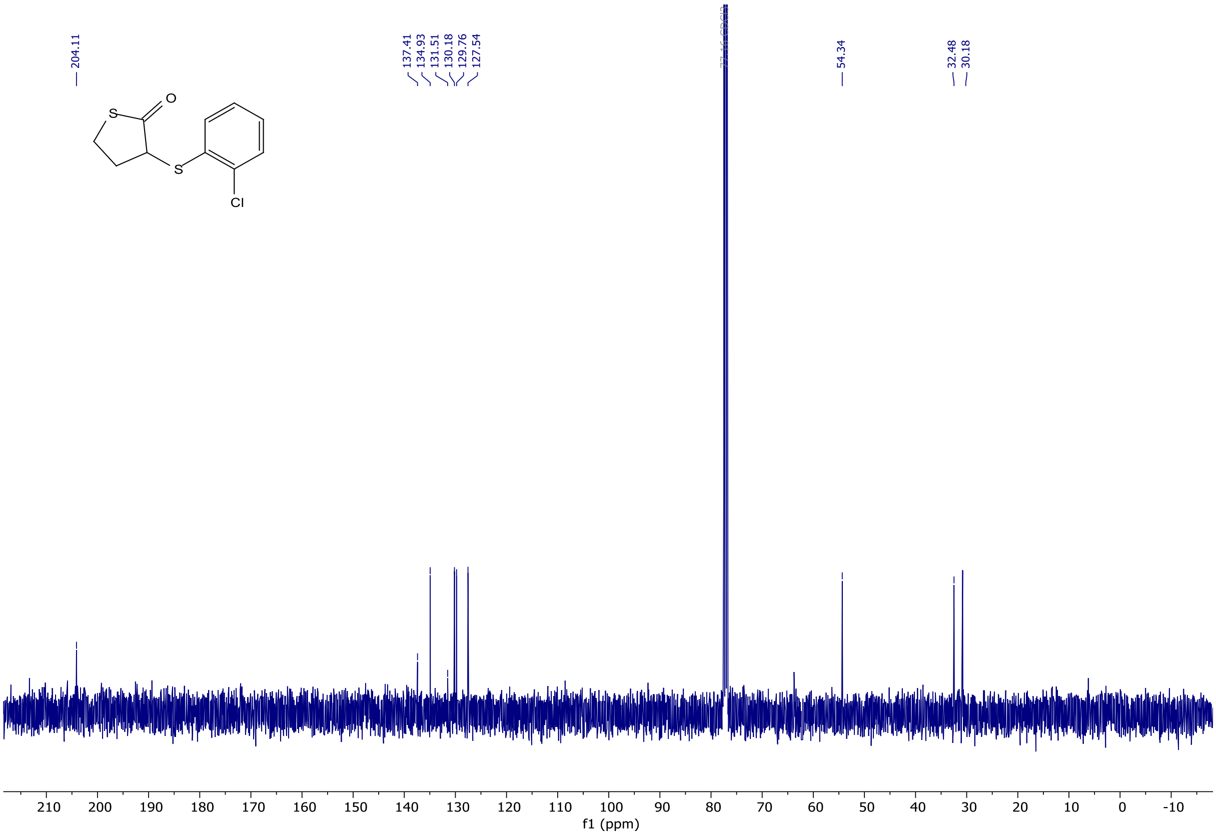

3-((2-Bromophenyl)thio)dihydrothiophen-2(3H)-one 5h

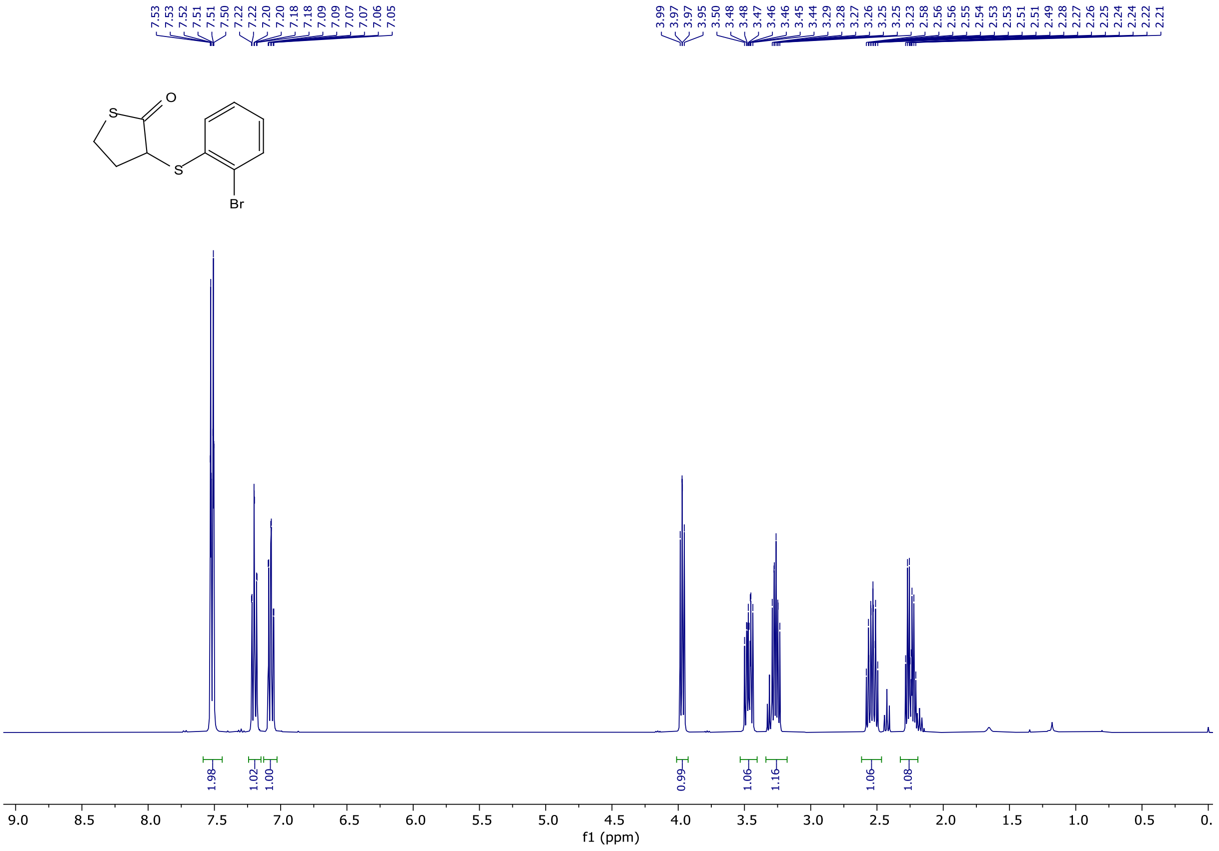

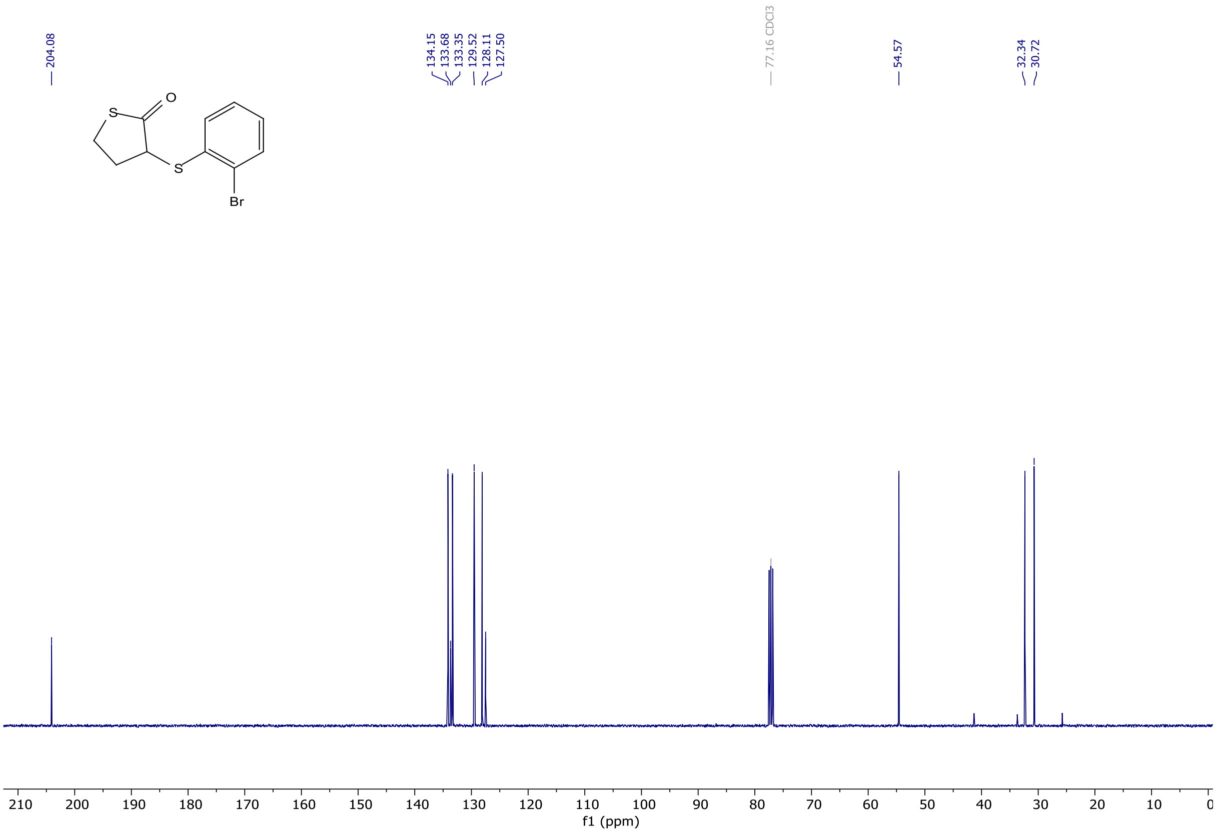

3-((3,5-Dimethylphenyl)thio)dihydrothiophen-2(3H)-one 5i

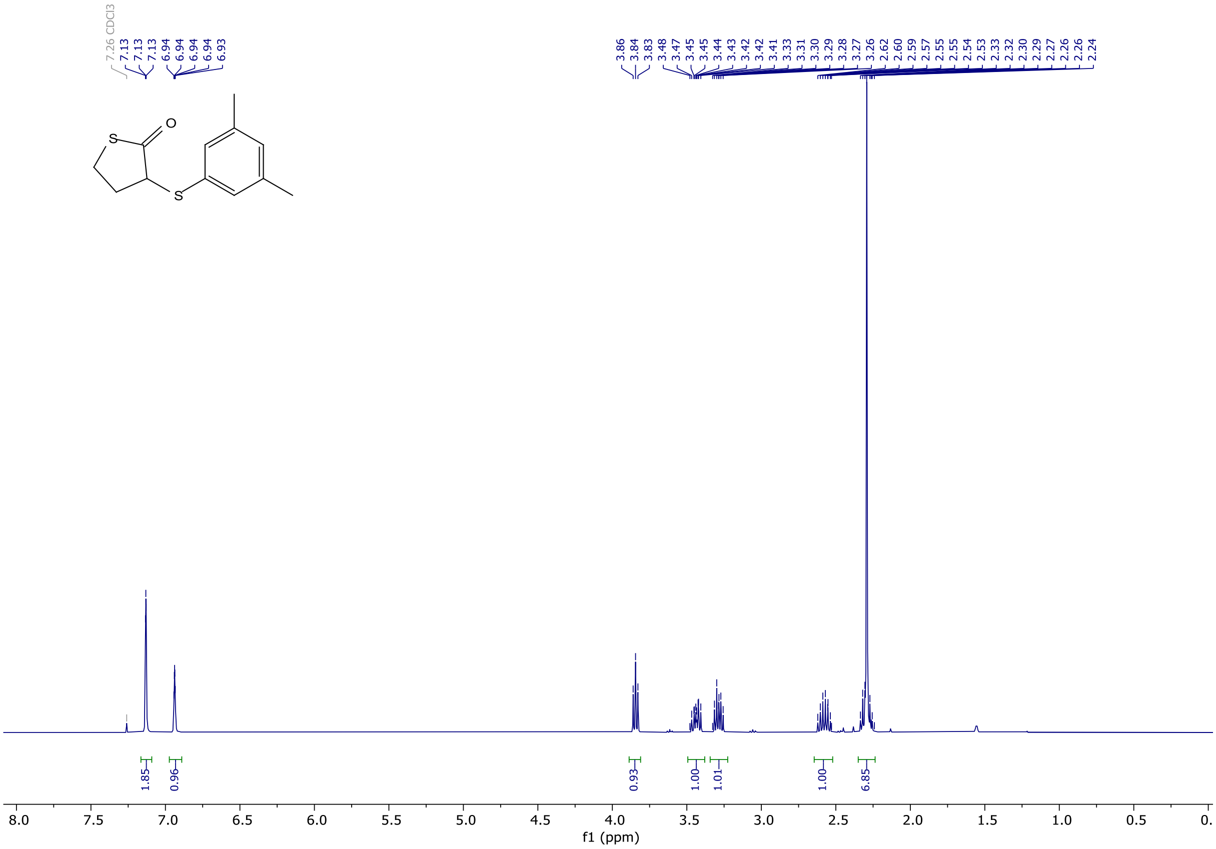

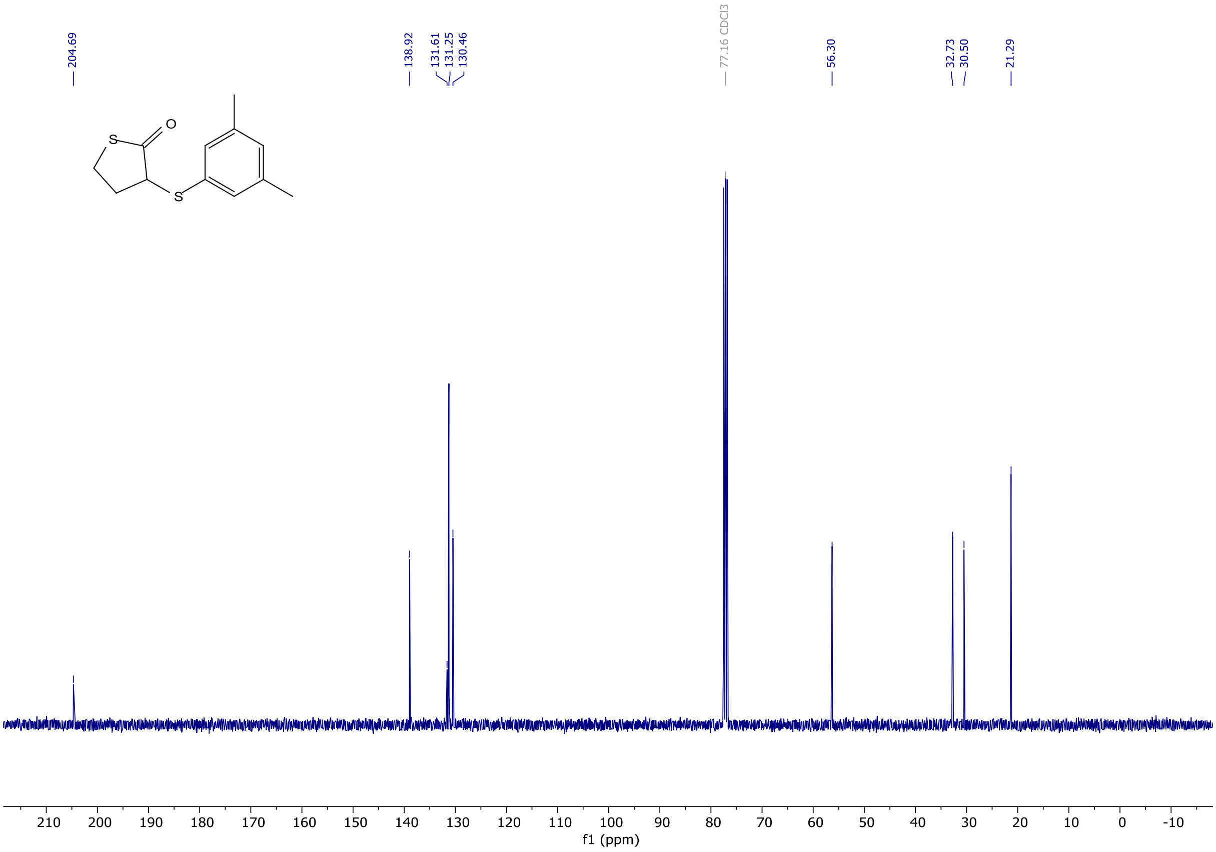

3-(Naphthalen-2-ylthio)dihydrothiophen-2(3H)-one 5j

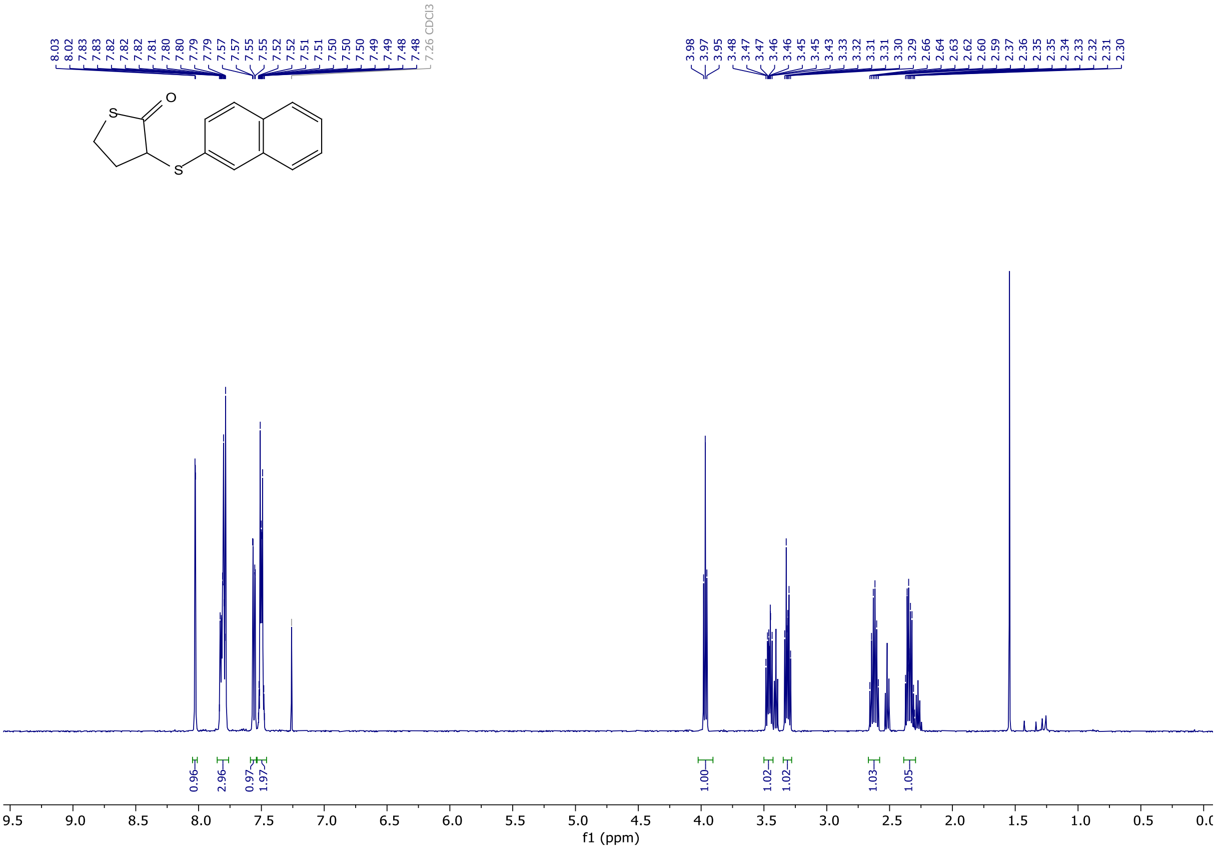

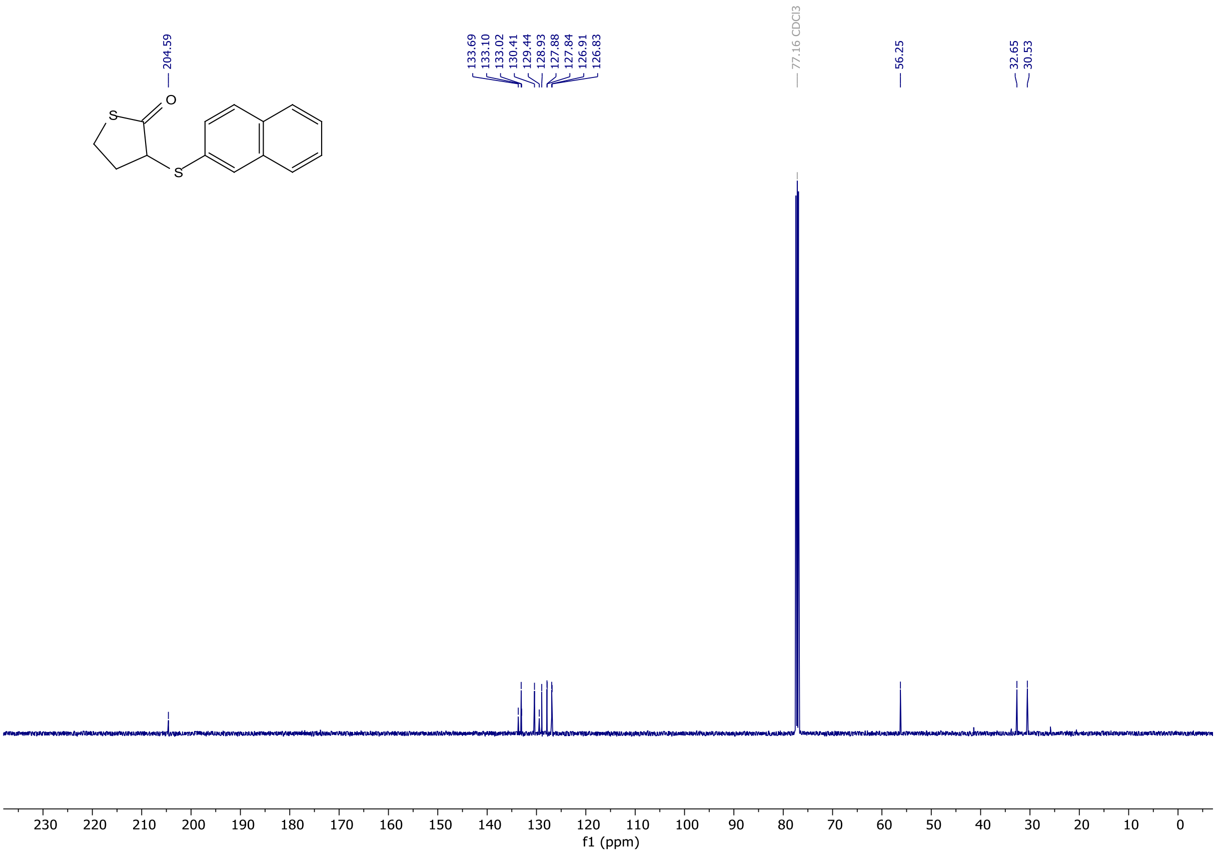

3-(Propylthio)dihydrothiophen-2(3H)-one 5k

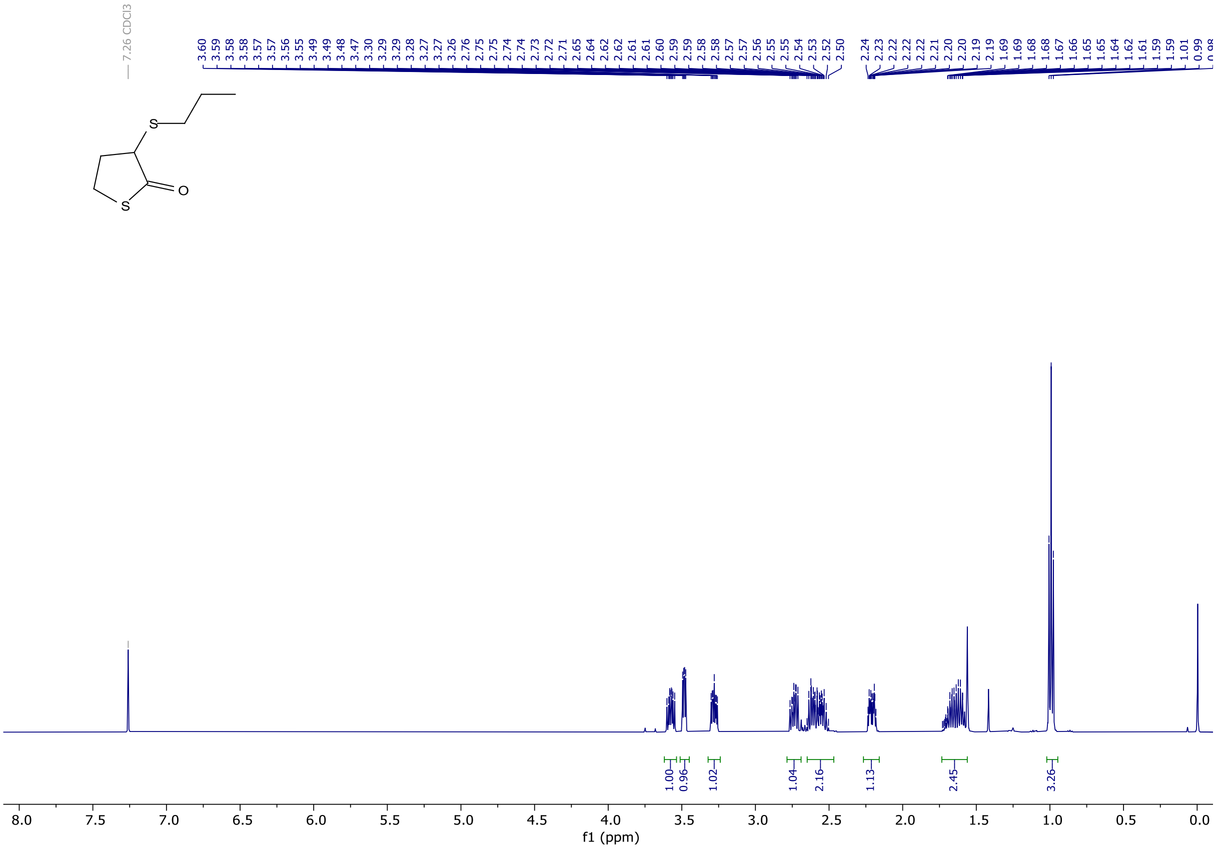

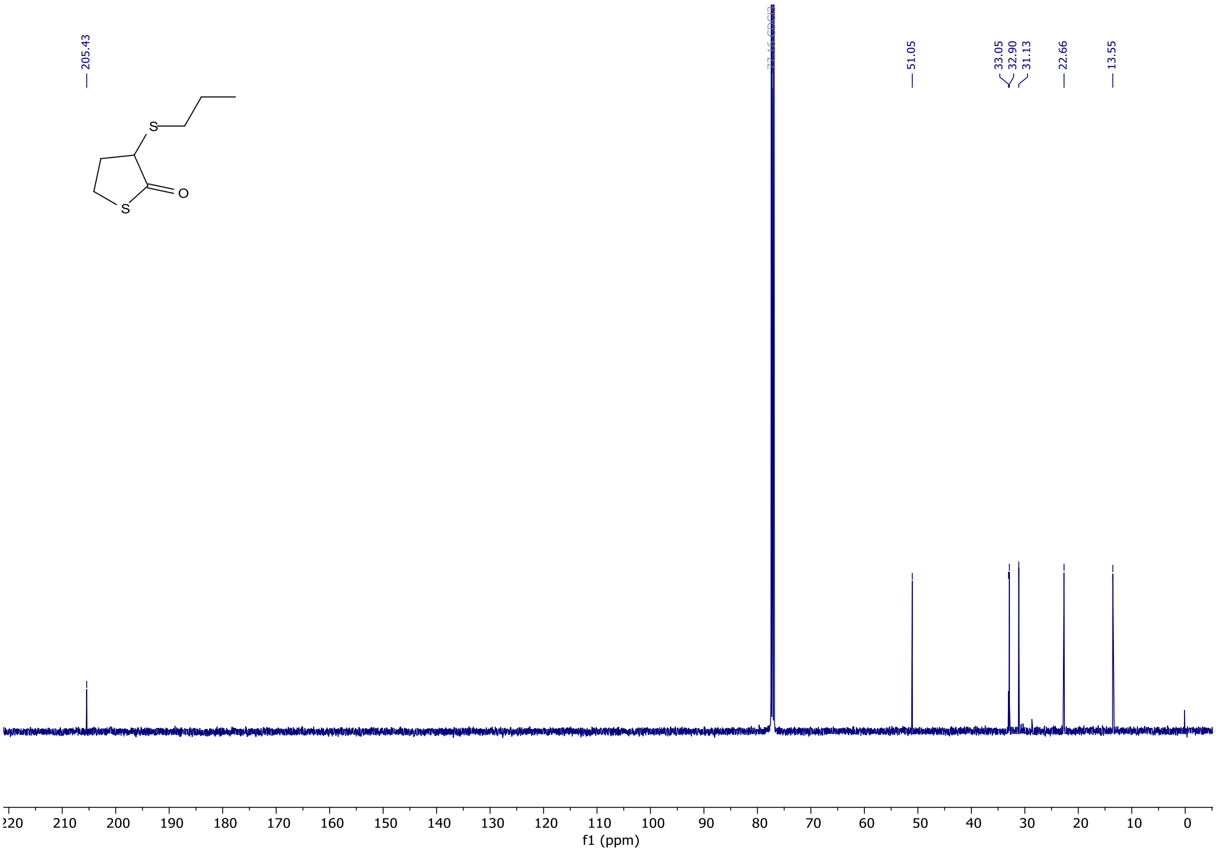

3-(Isobutylthio)dihydrothiophen-2(3H)-one 5l

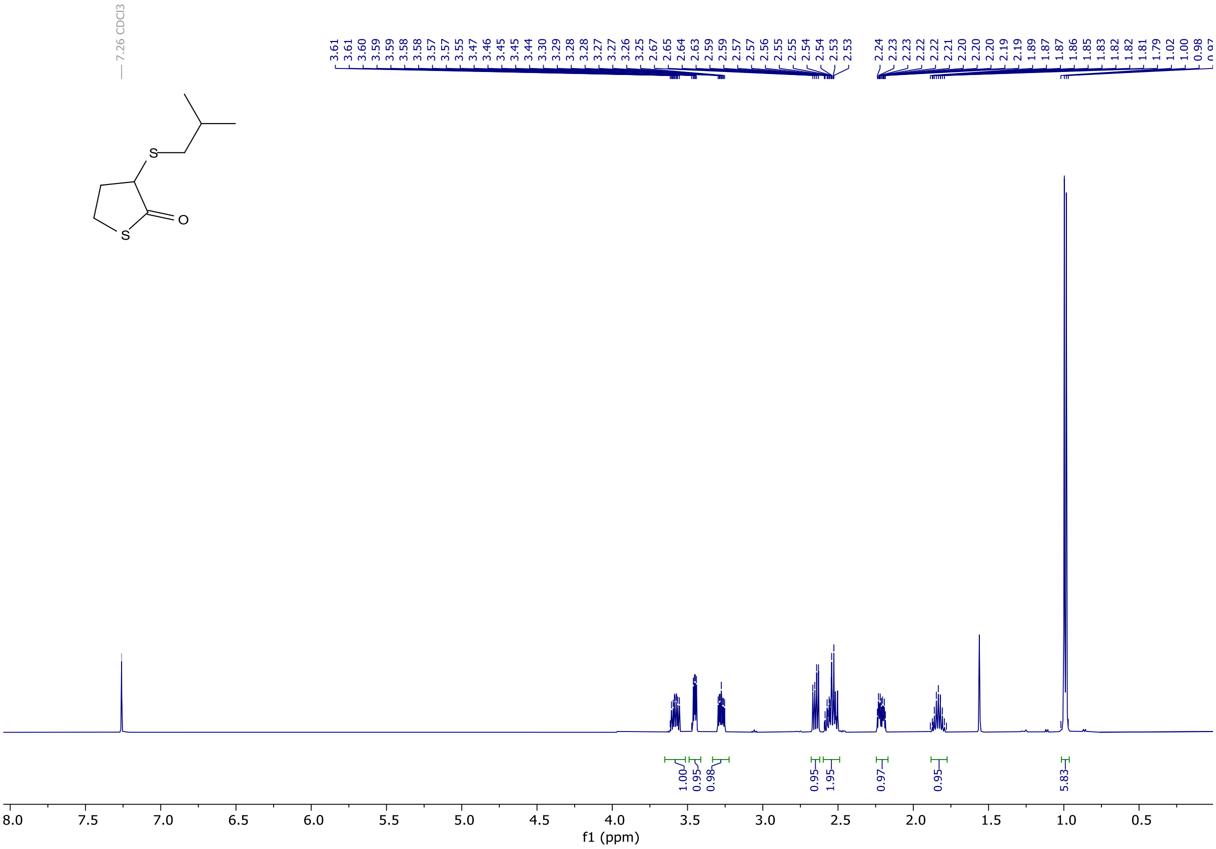

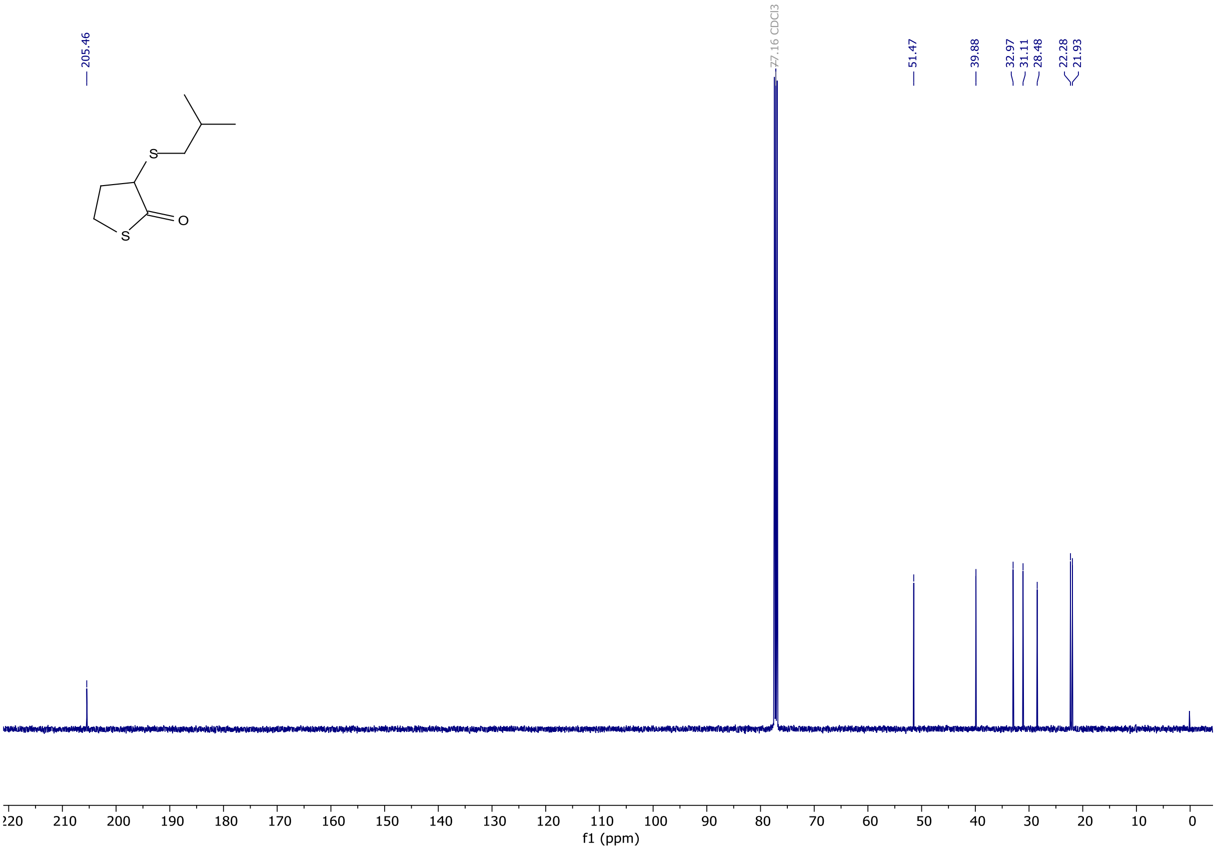

3-(Cyclohexylthio)dihydrothiophen-2(3H)-one 5m

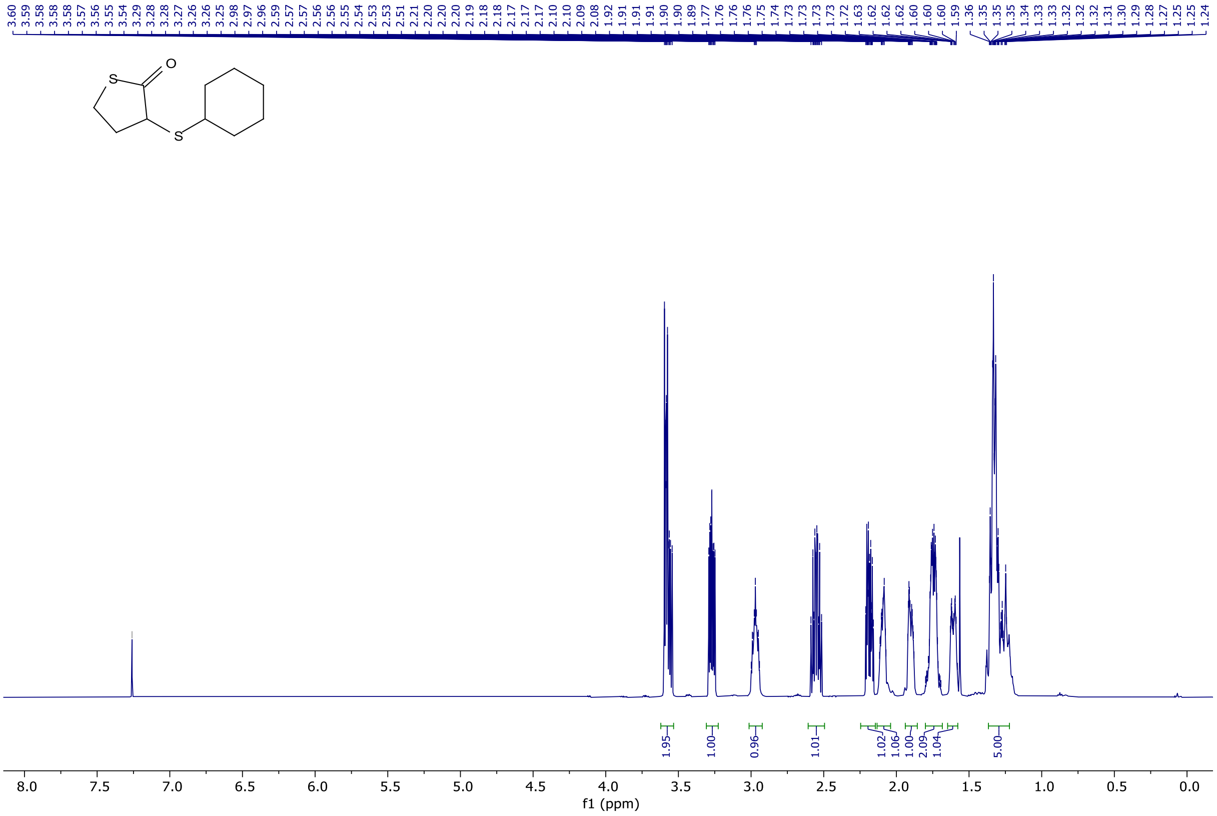

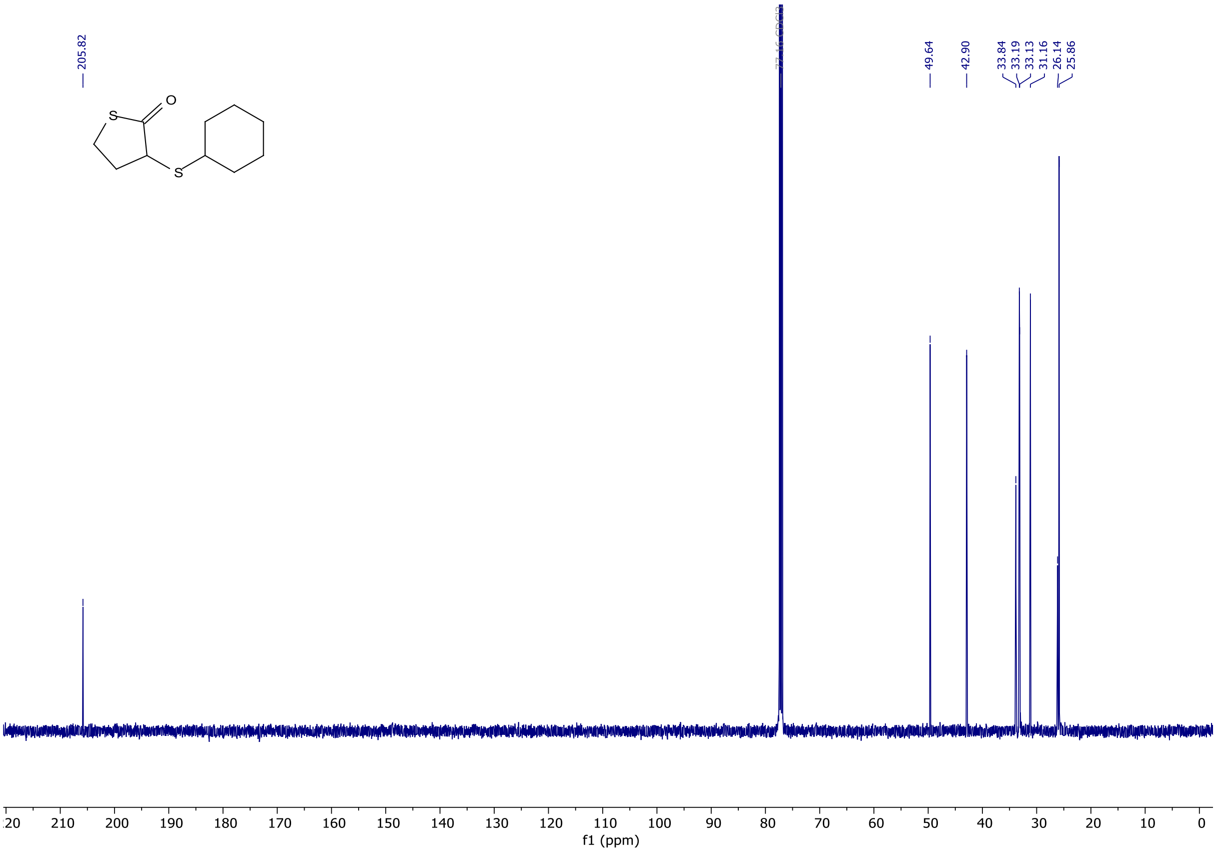

3-(Benzylthio)dihydrothiophen-2(3H)-one 5n

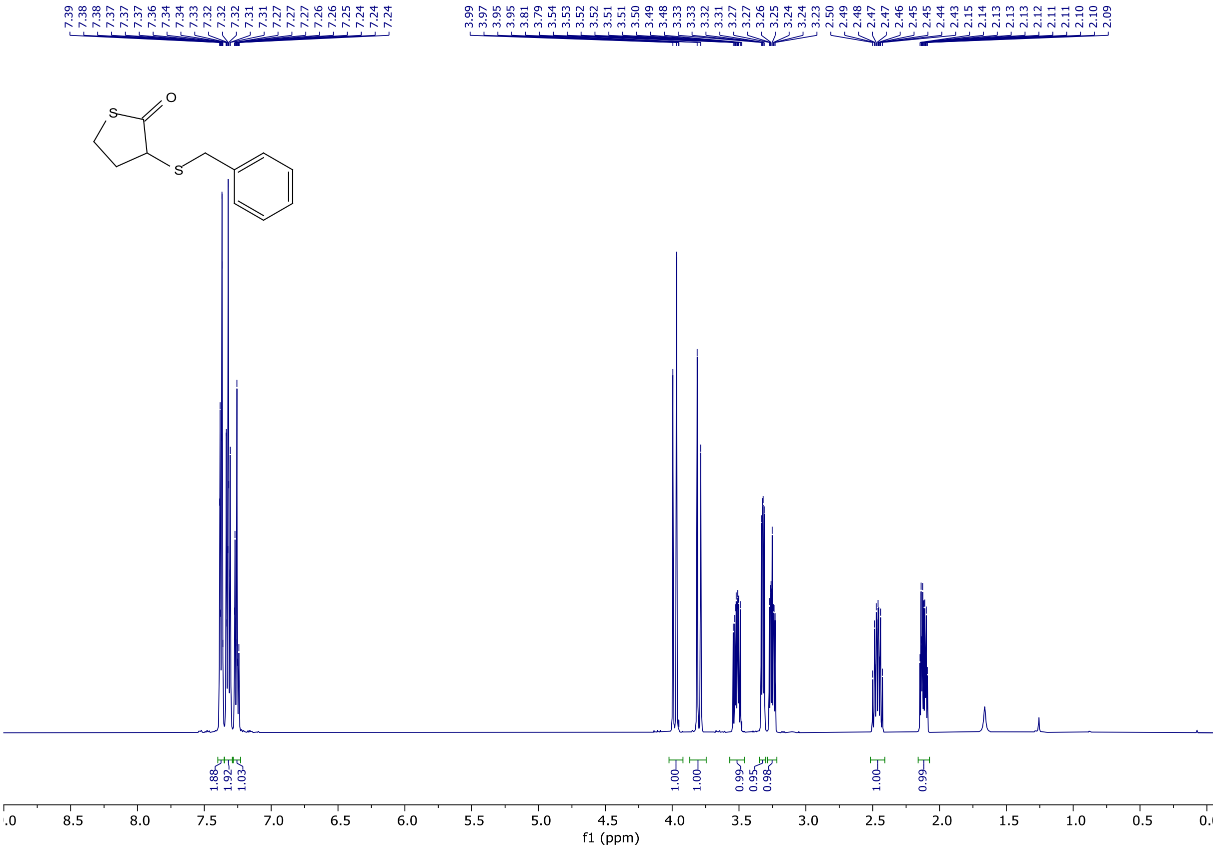

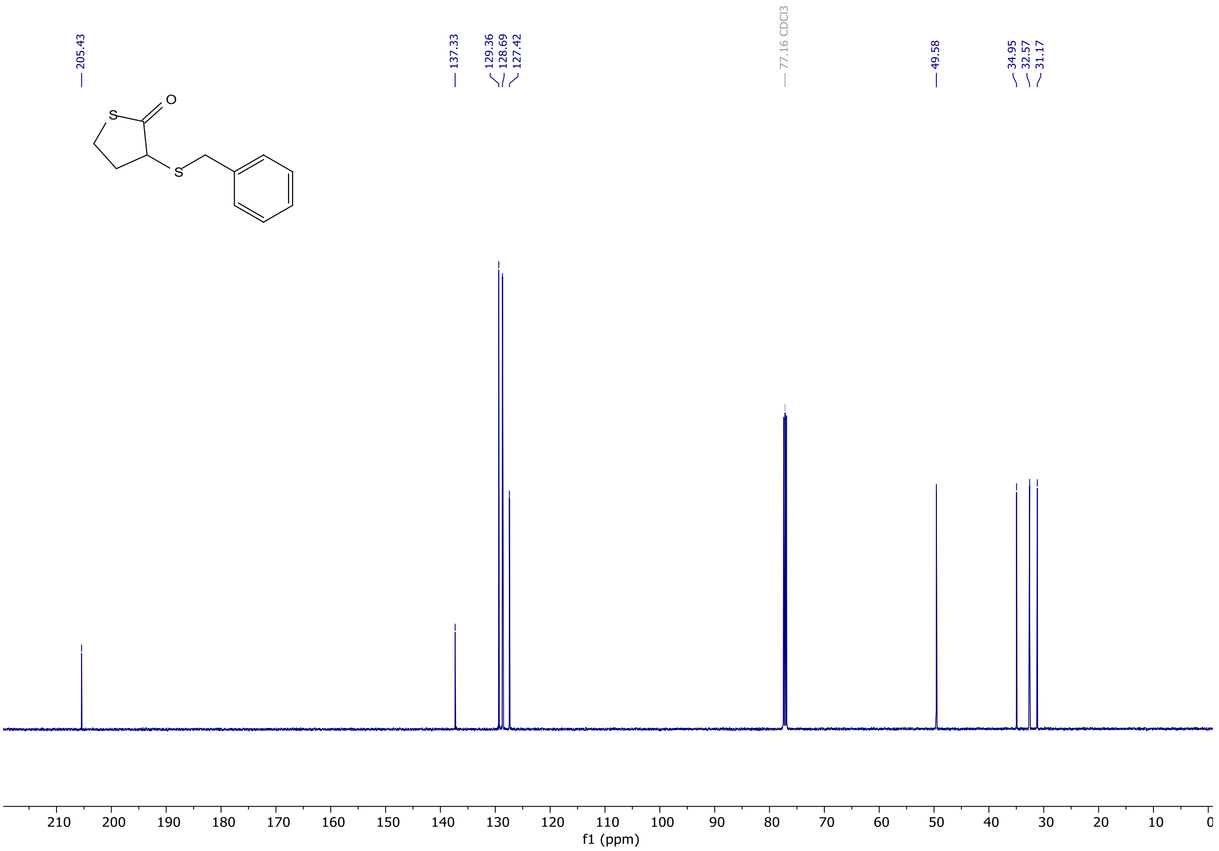

3-(Phenethylthio)dihydrothiophen-2(3H)-one 5o

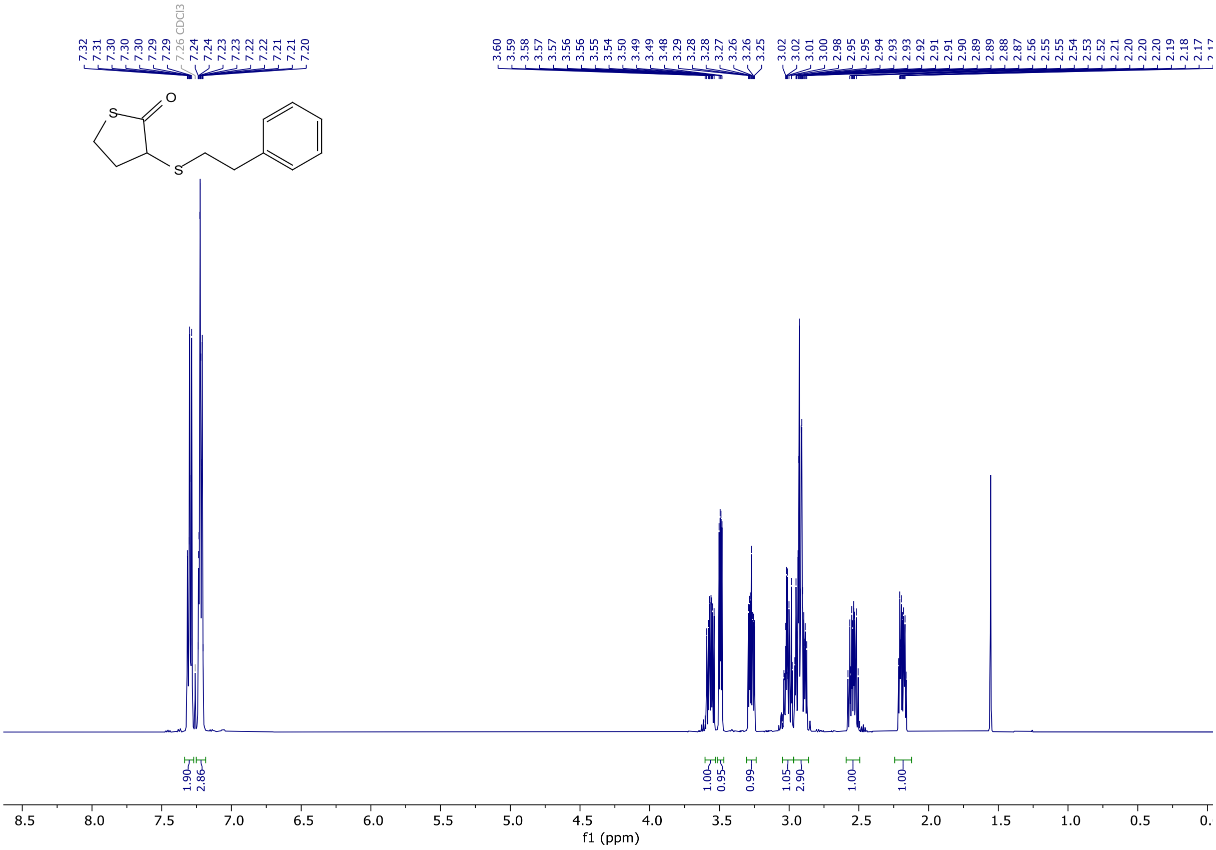

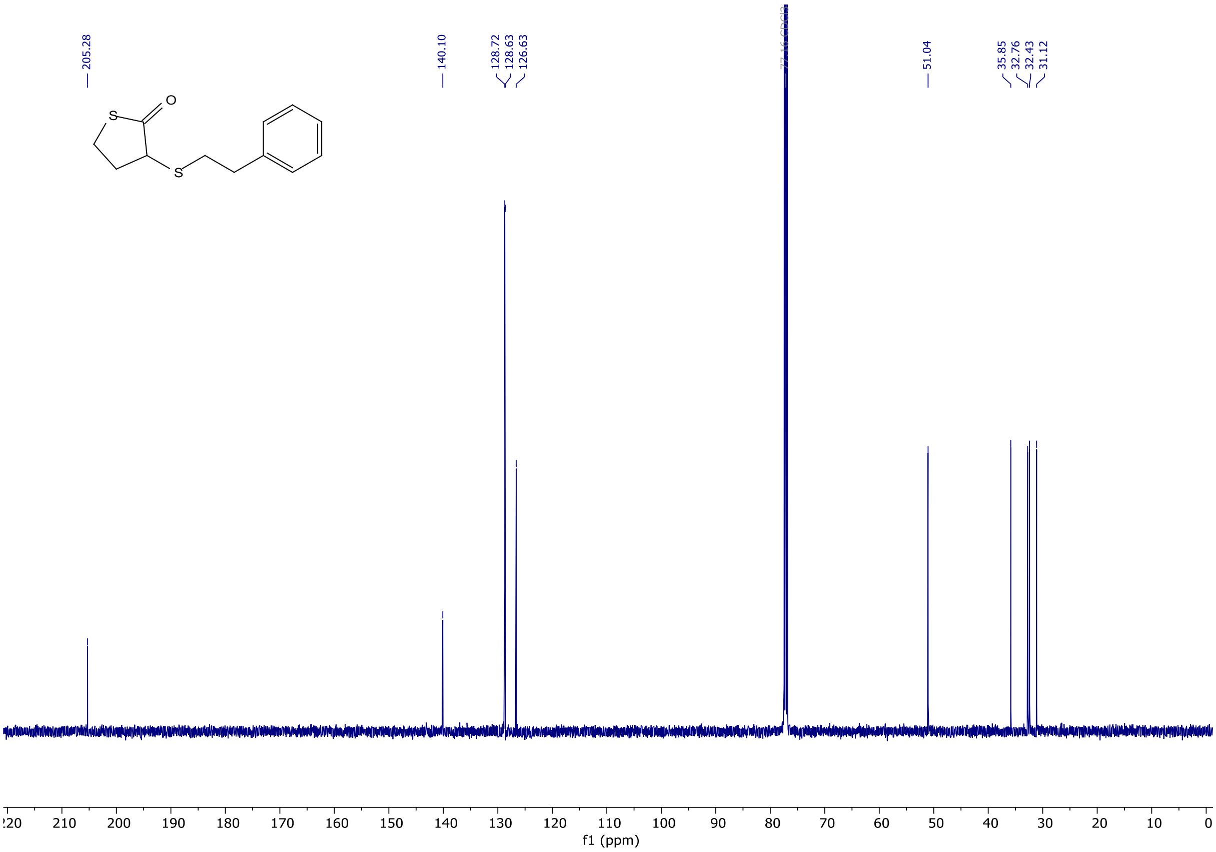

3-((Furan-2-ylmethyl)thio)dihydrothiophen-2(3H)-one 5p

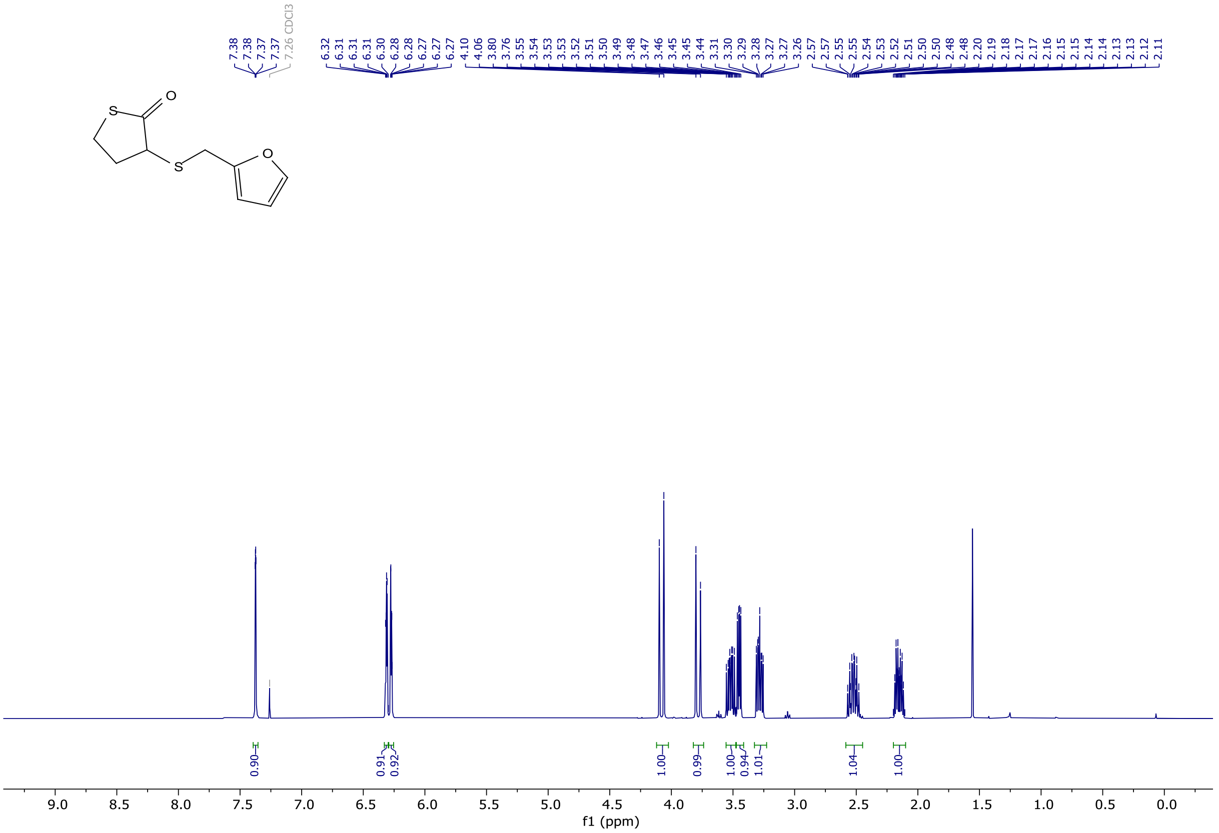

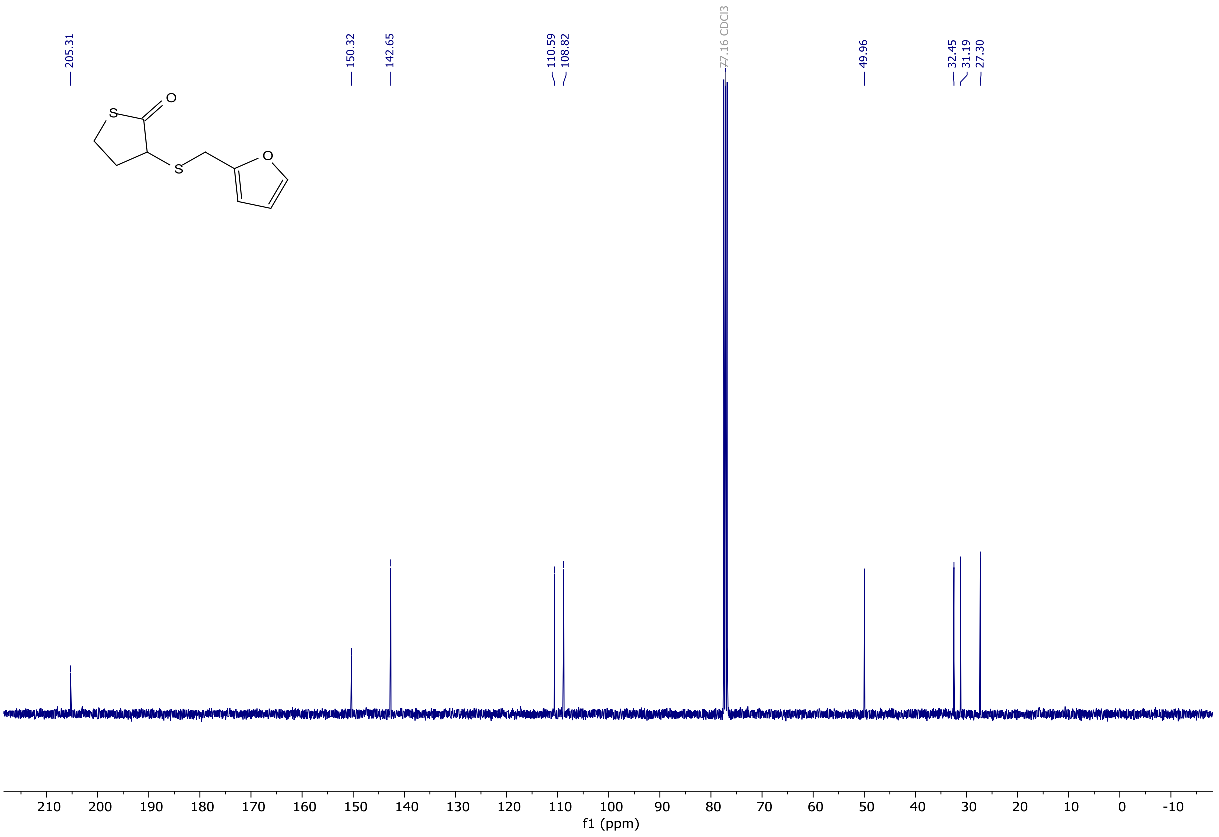

4-Mercapto-2-(phenylthio)butanoic acid 6a

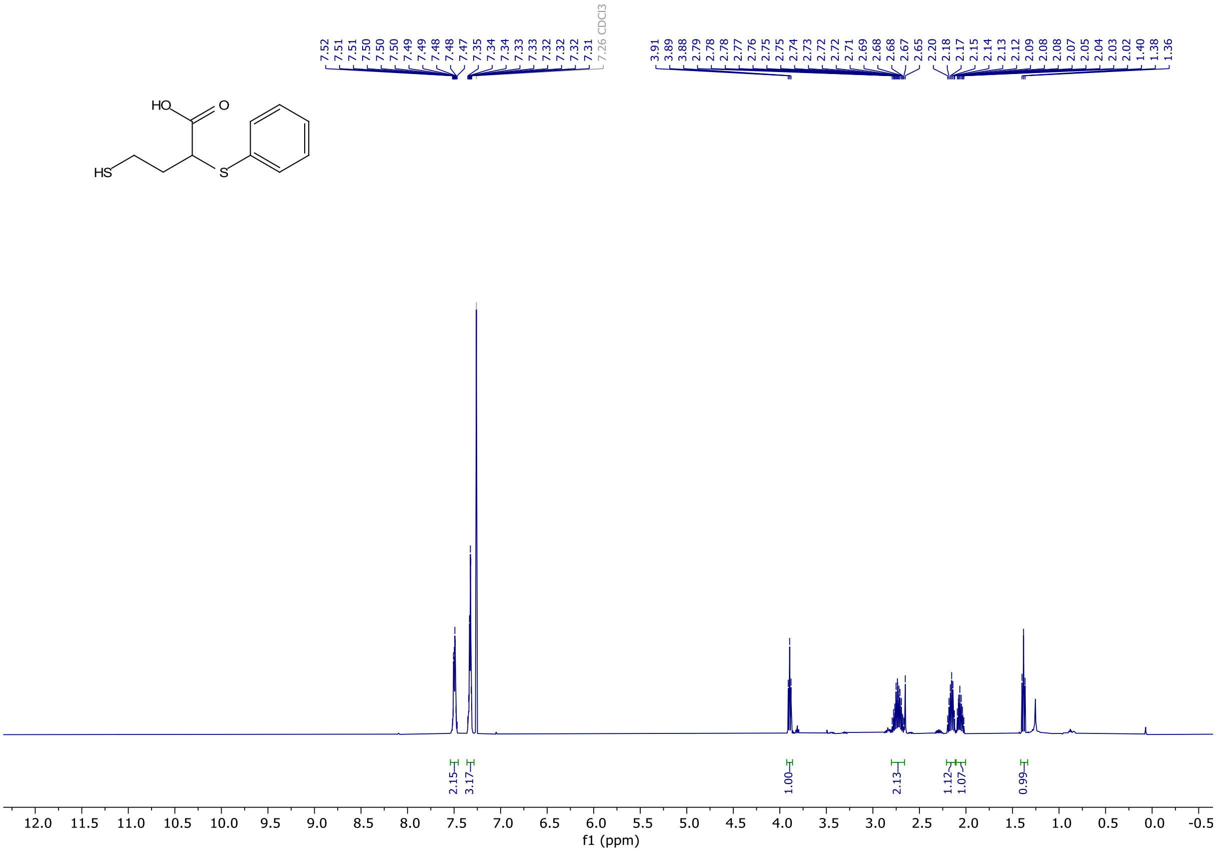

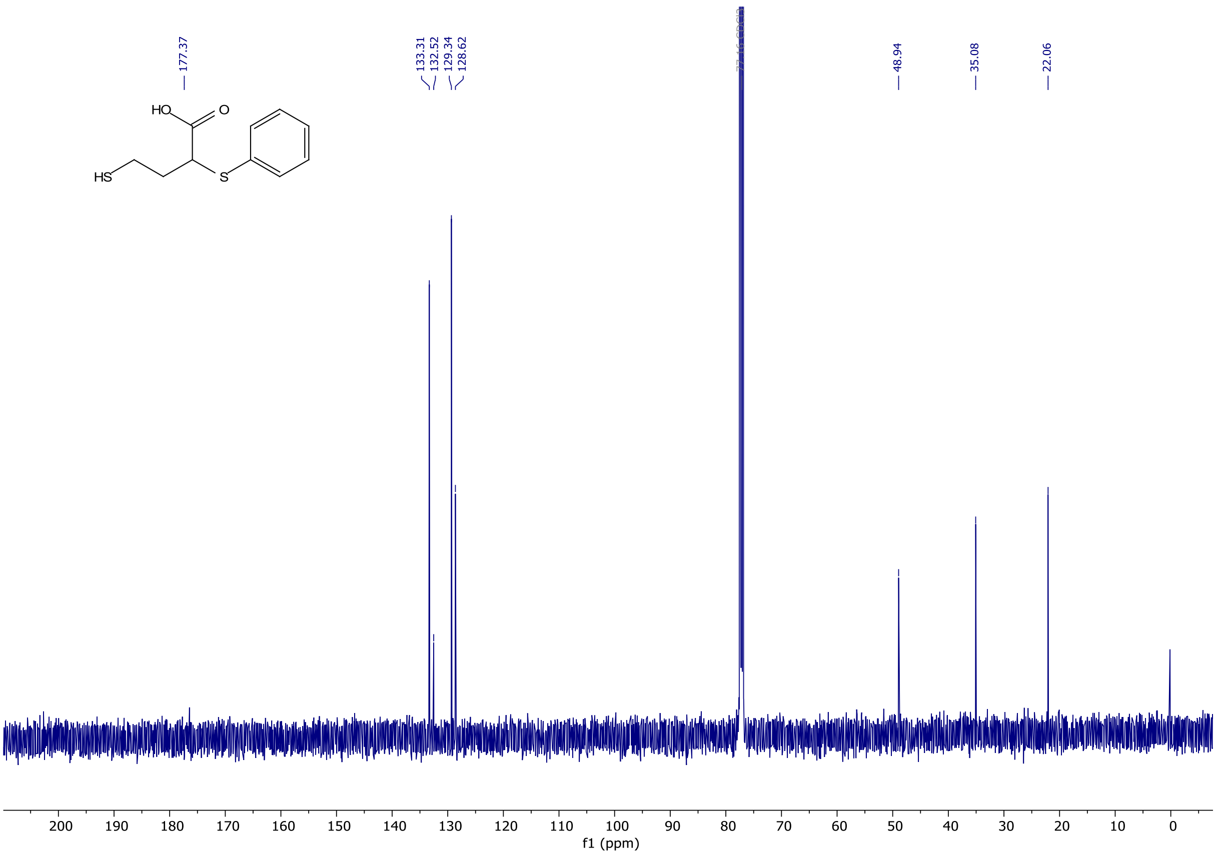

2-((4-Chlorophenyl)thio)-4-mercaptobutanoic acid 6b

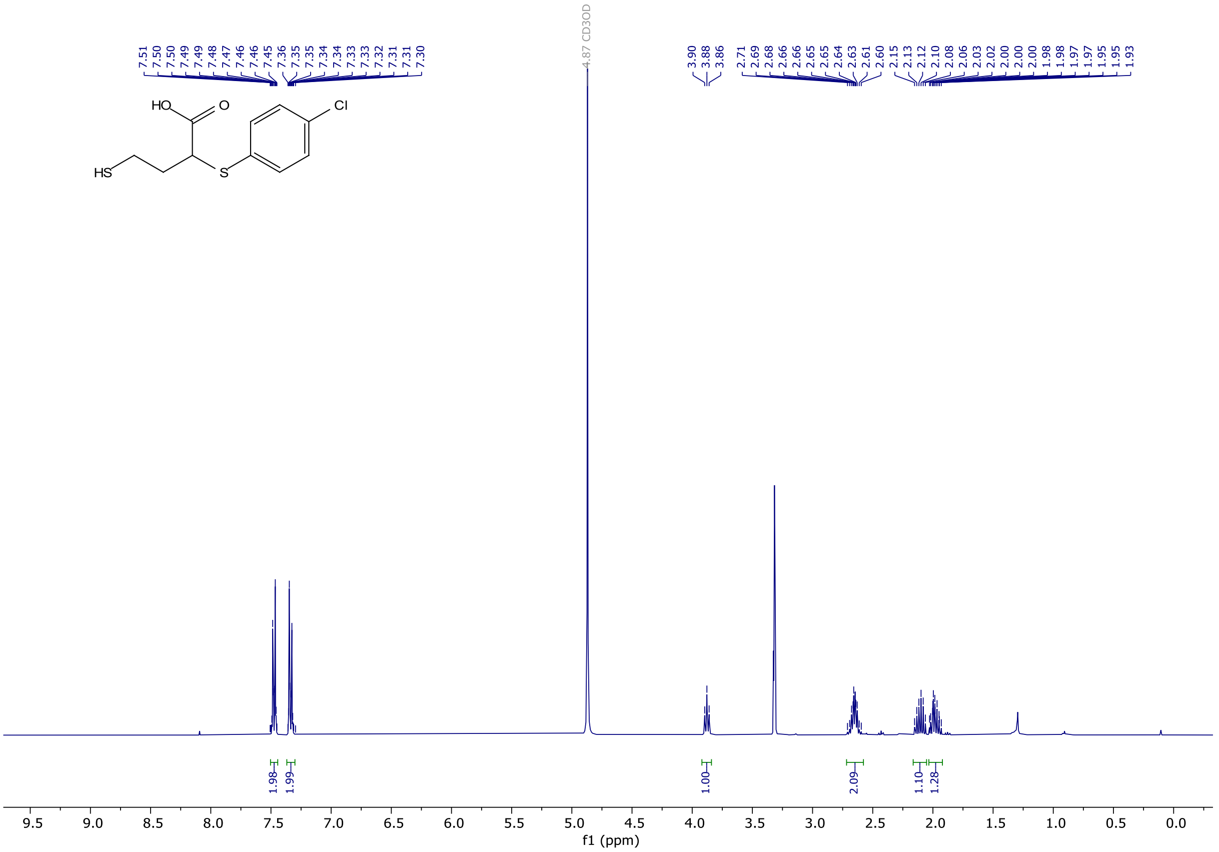

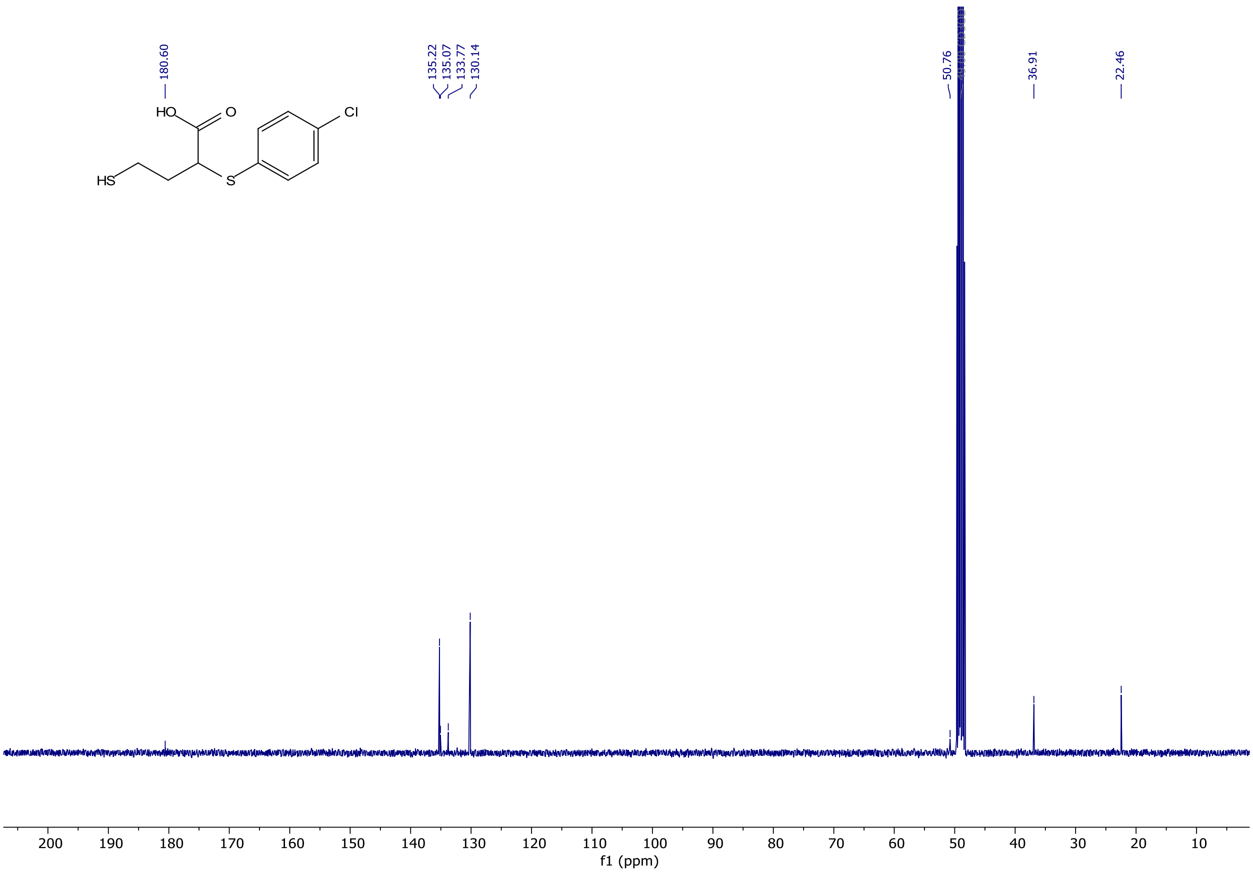

2-((4-Bromophenyl)thio)-4-mercaptobutanoic acid 6c

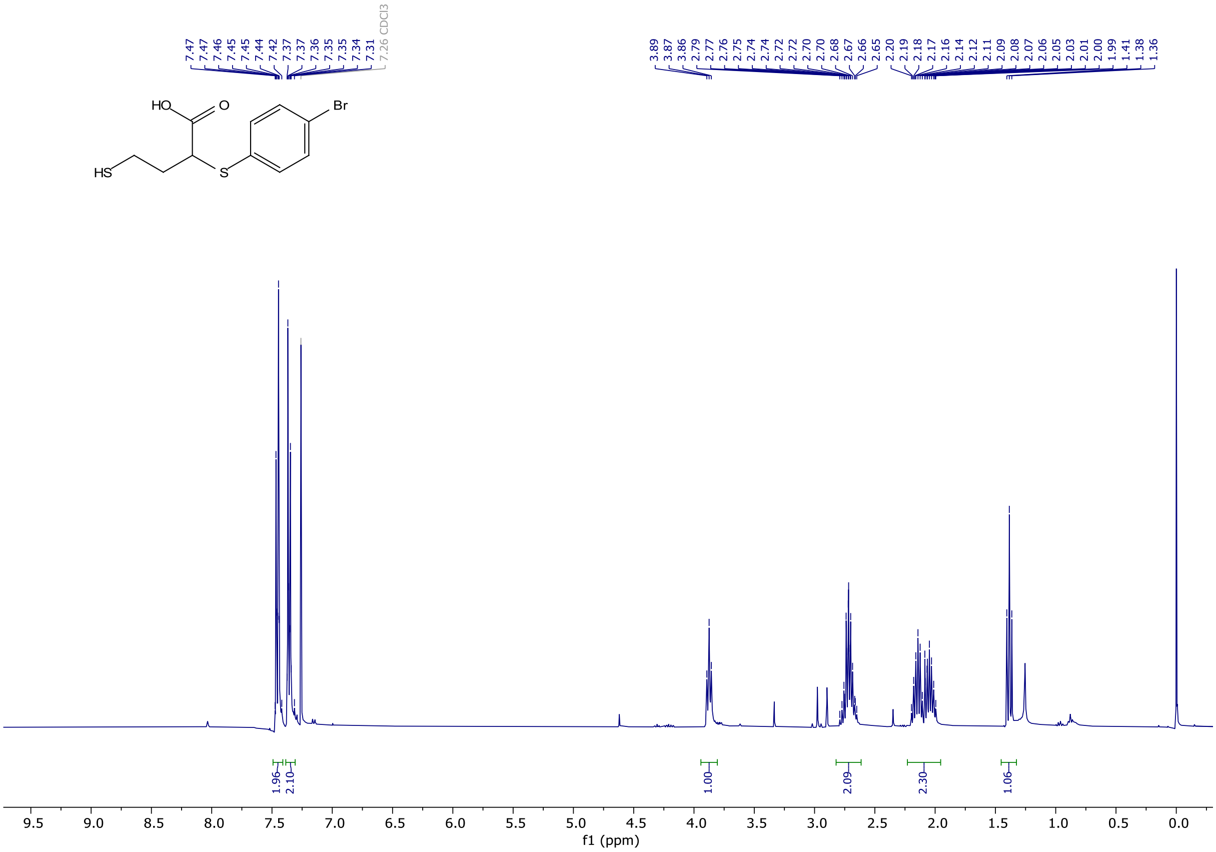

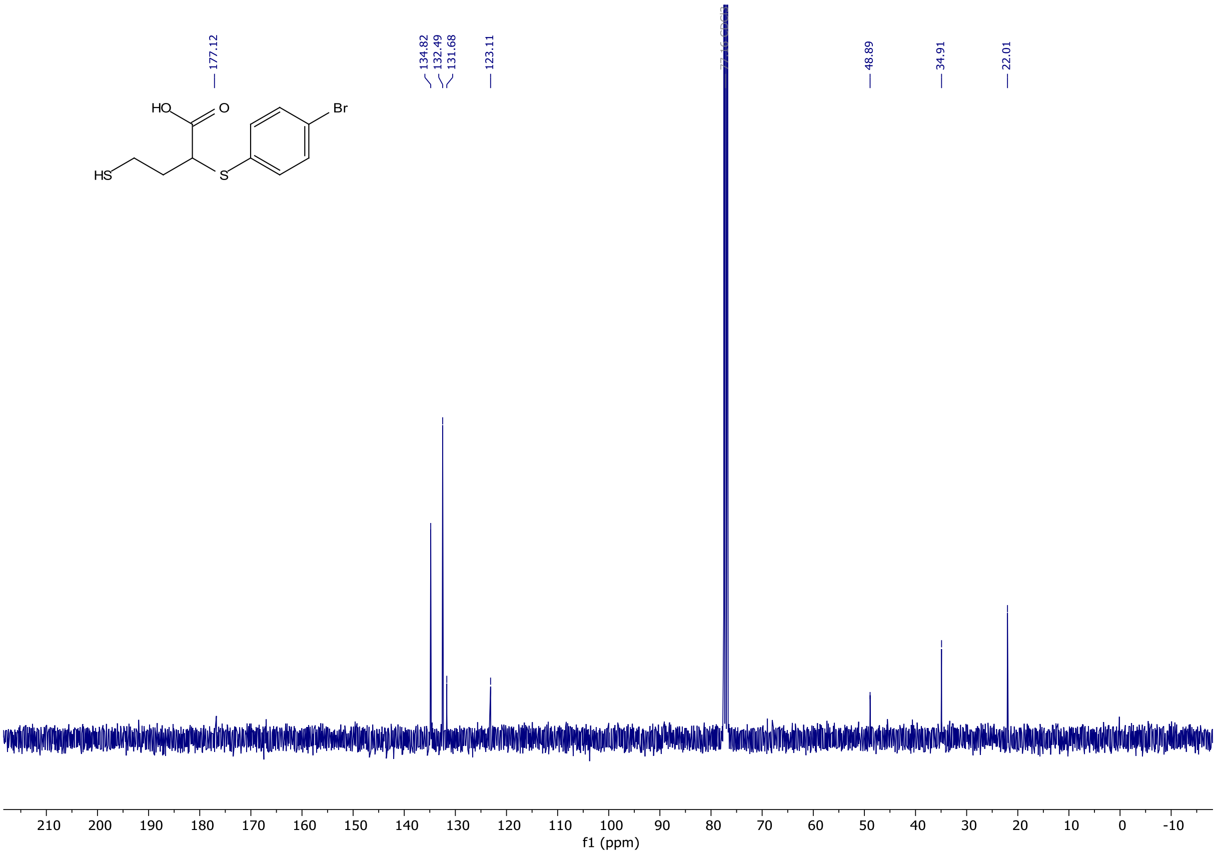

4-Mercapto-2-((4-methoxyphenyl)thio)butanoic acid 6d

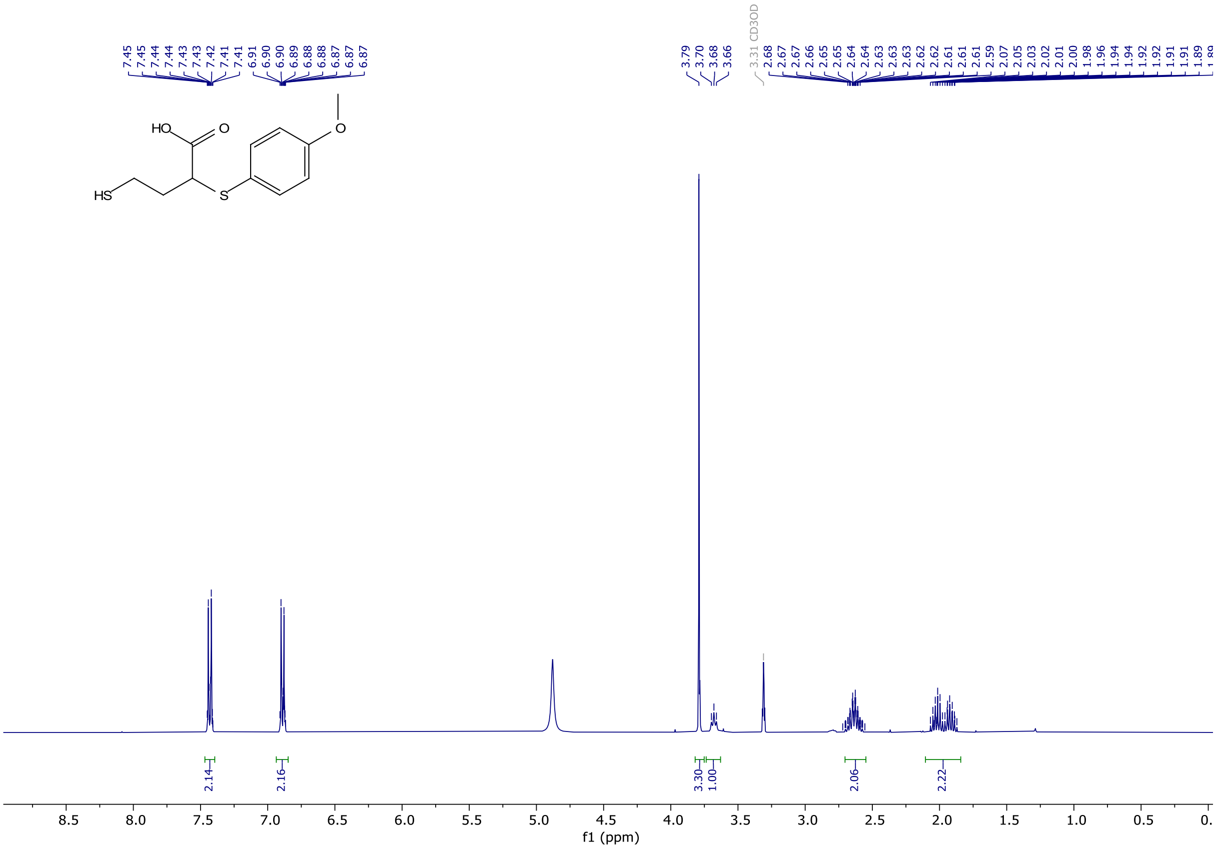

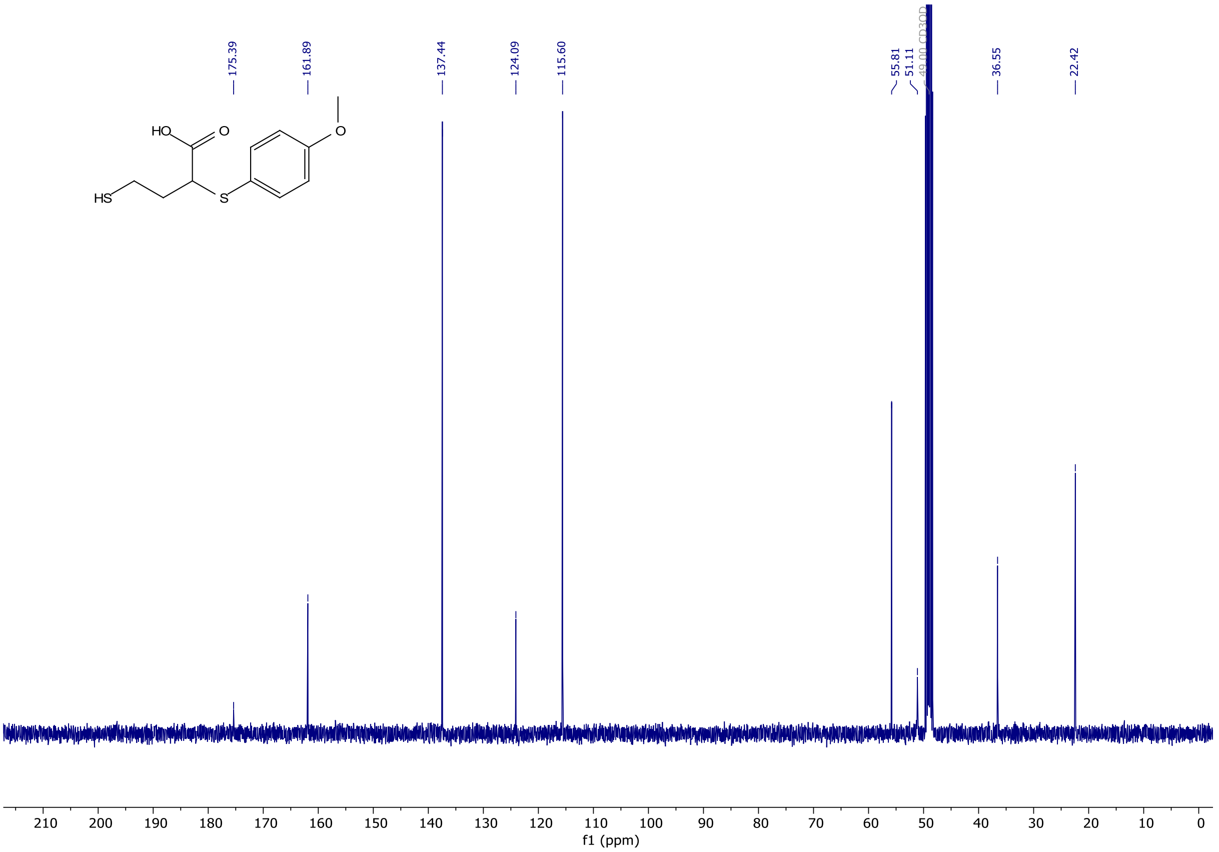

4-Mercapto-2-(*p*-tolylthio)butanoic acid 6e

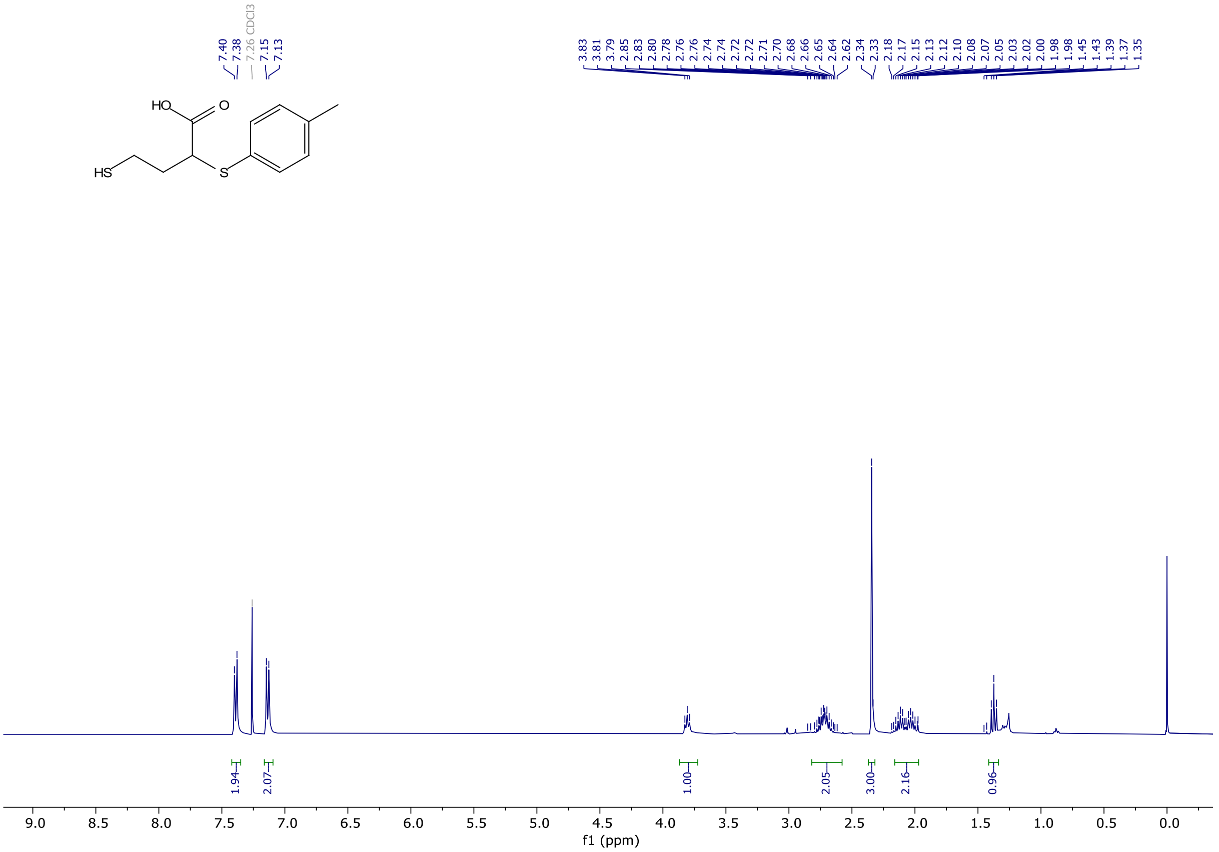

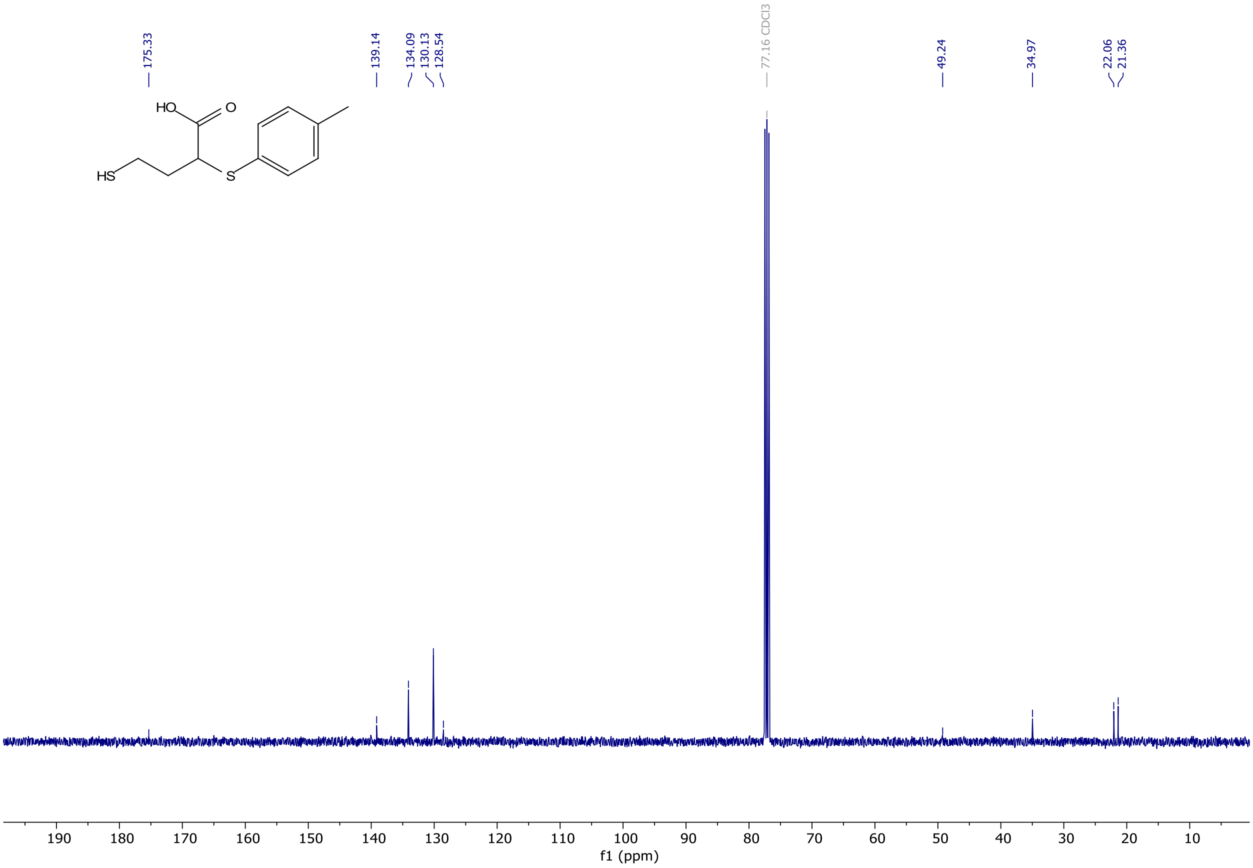

2-((2-Fluorophenyl)thio)-4-mercaptobutanoic acid 6f

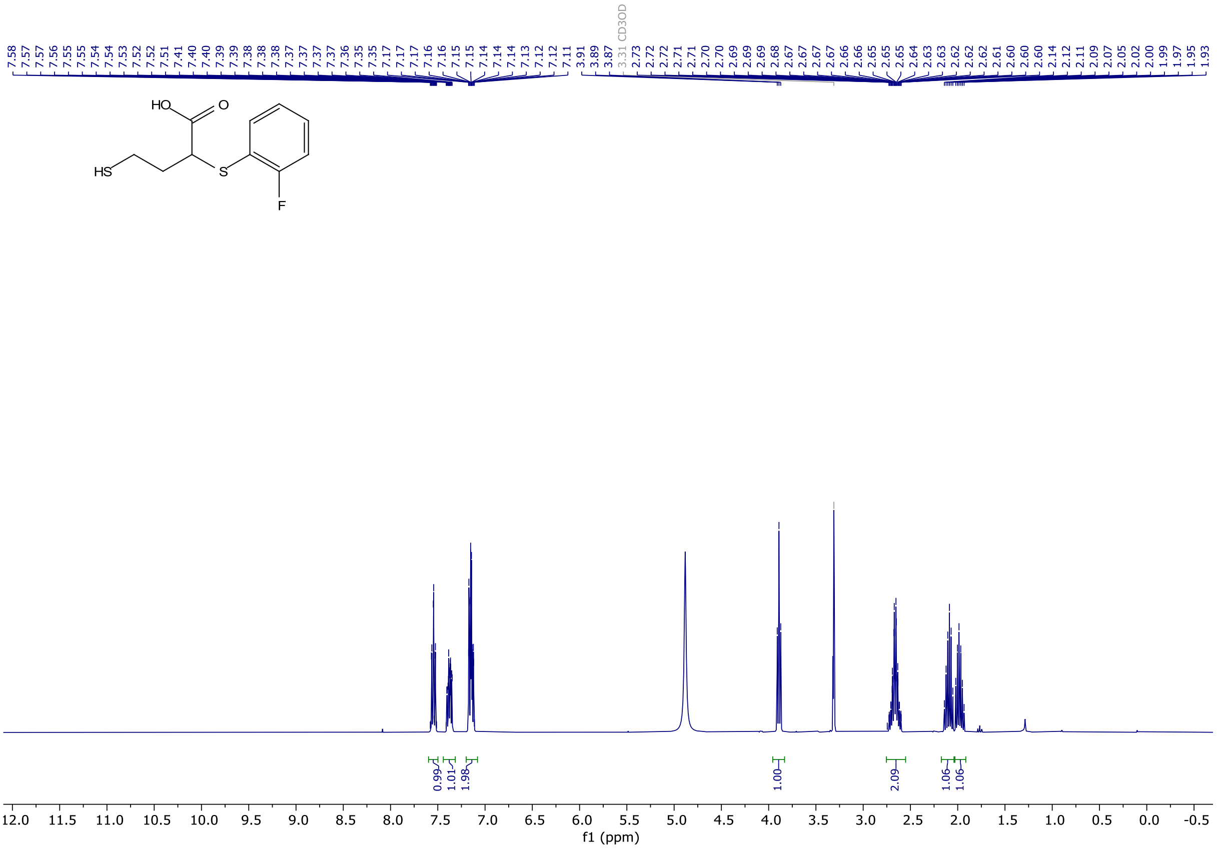

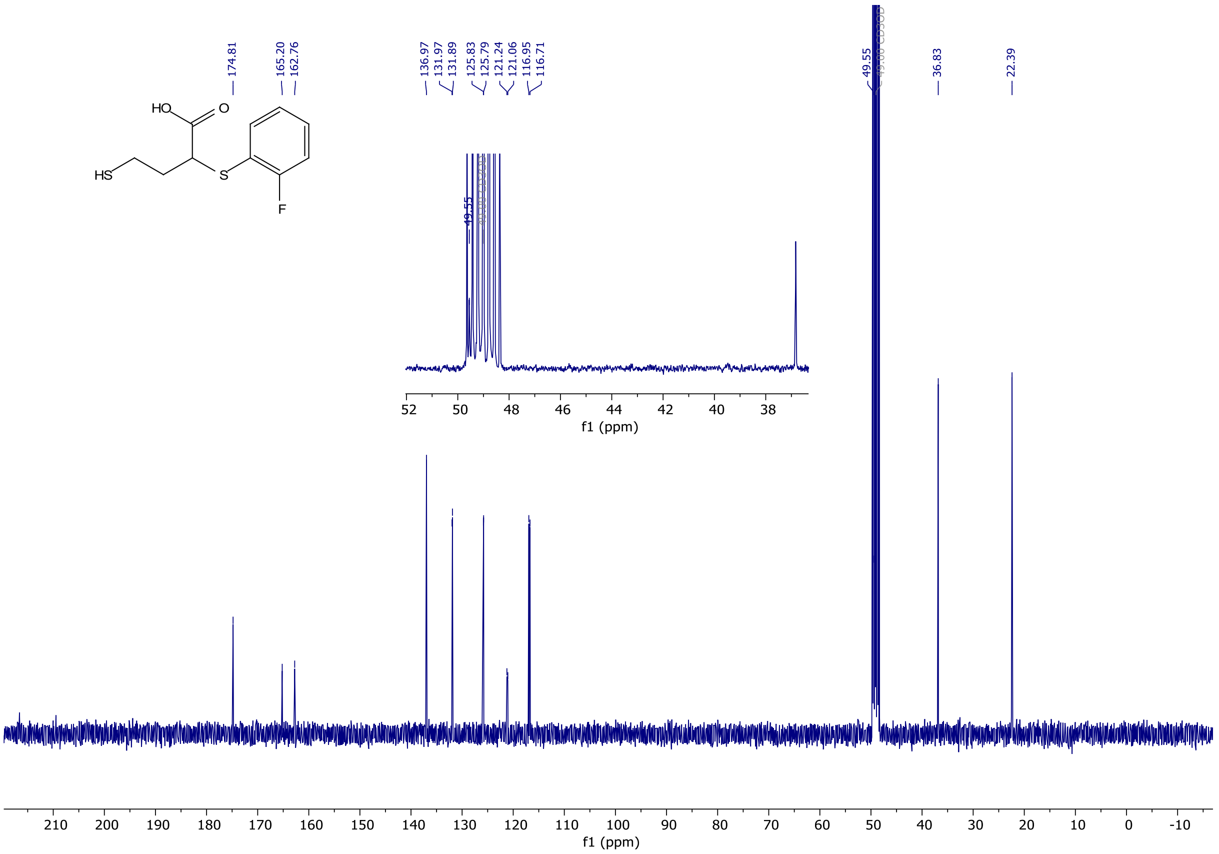

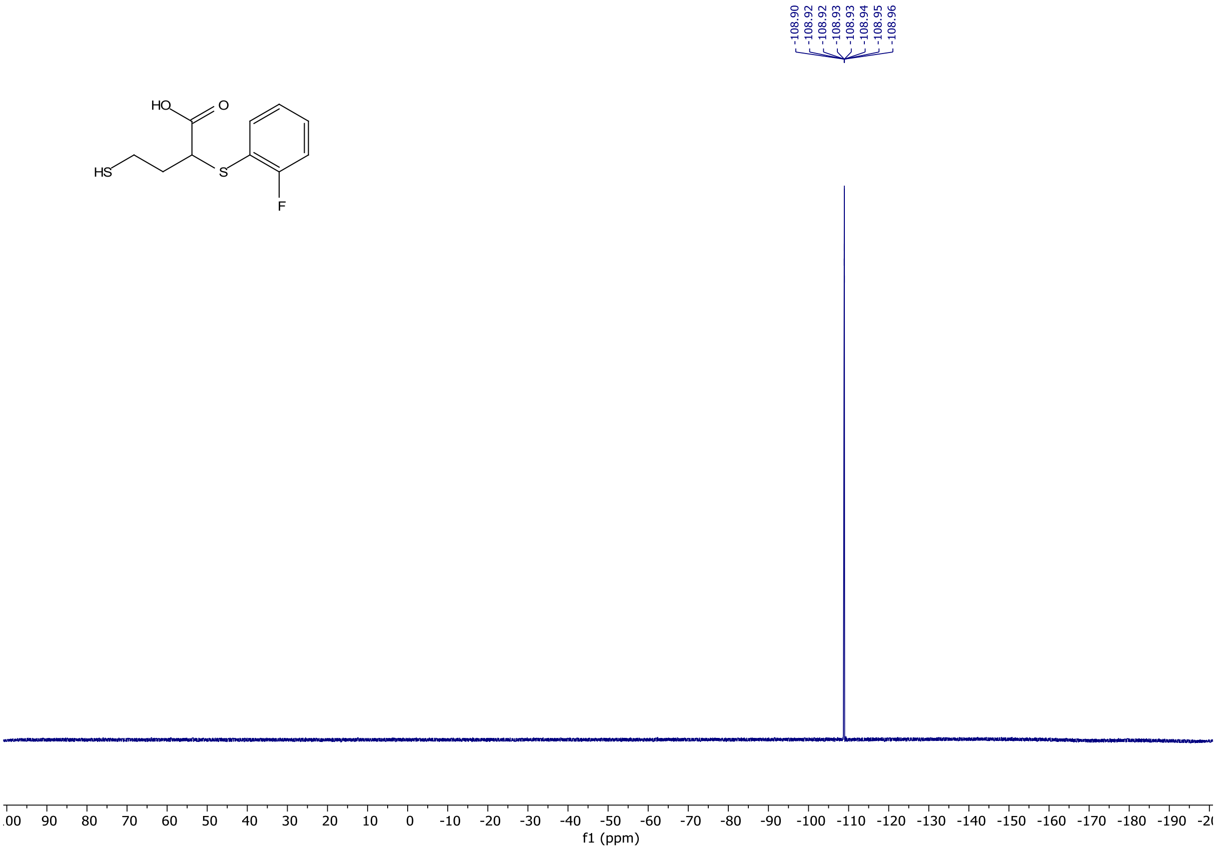
HO
O
HS
S
F
-108.90
-108.92
-108.92
-108.93
-108.93
-108.94
-108.95
-108.96
90 80 70 60 50 40 30 20 10 0 -10 -20 -30 -40 -50 -60 -70 -80 -90 -100 -110 -120 -130 -140 -150 -160 -170 -180 -190
f1 (ppm)

2-((2-Chlorophenyl)thio)-4-mercaptobutanoic acid 6g

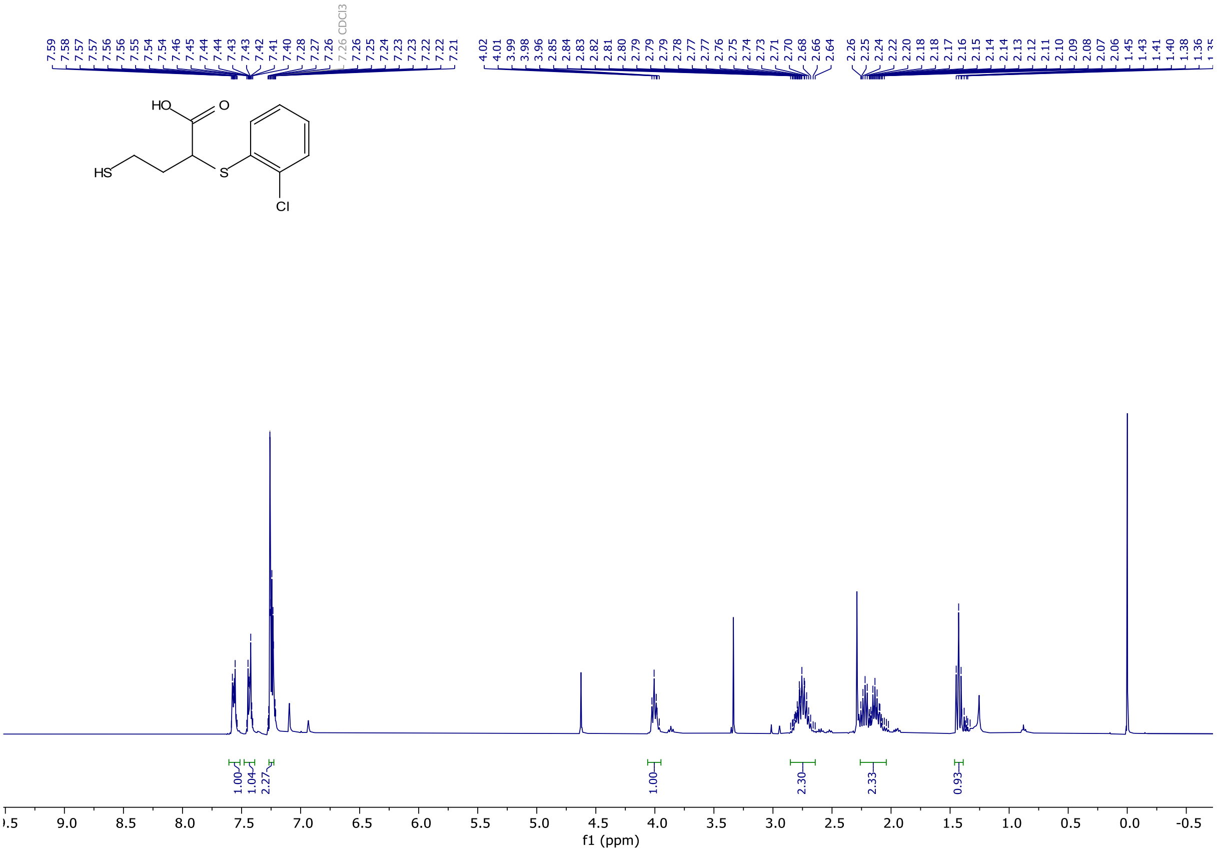

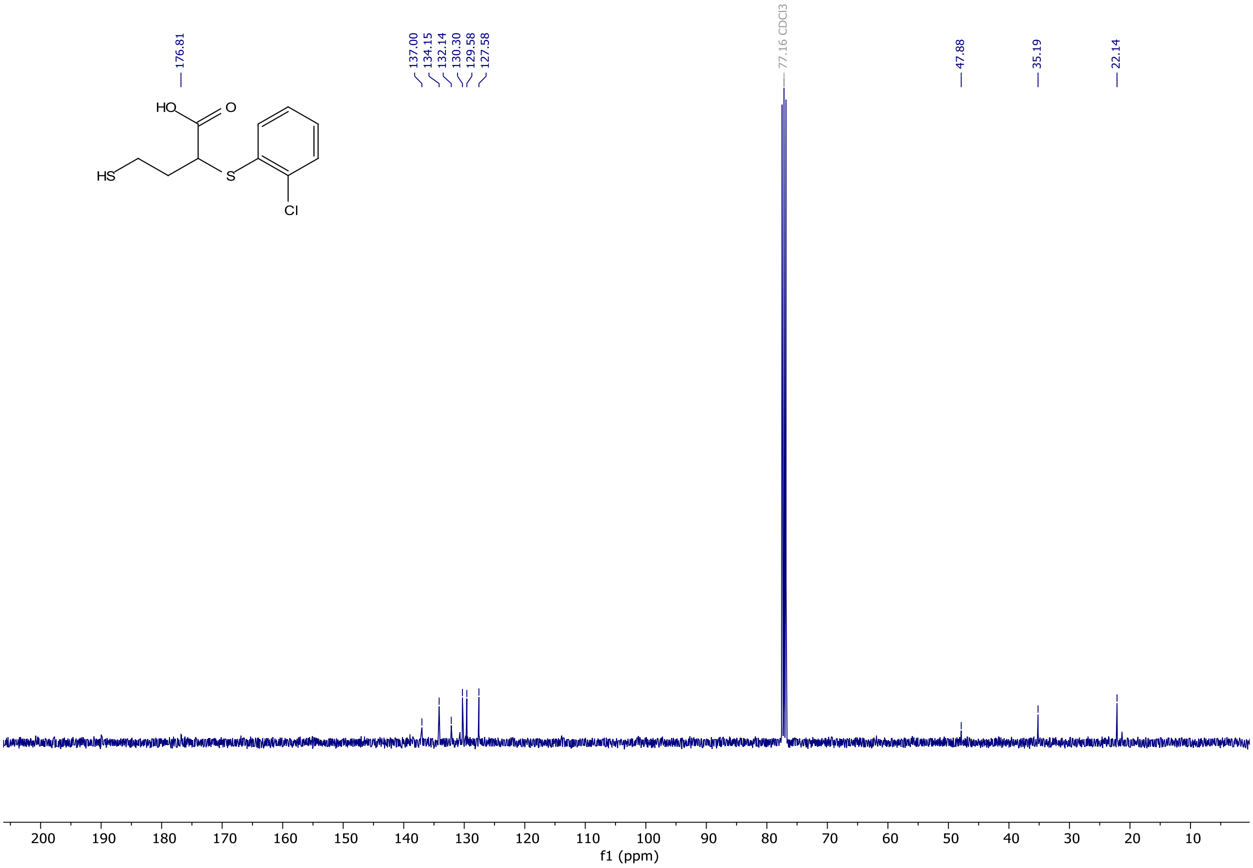

2-((2-Bromophenyl)thio)-4-mercaptobutanoic acid 6h

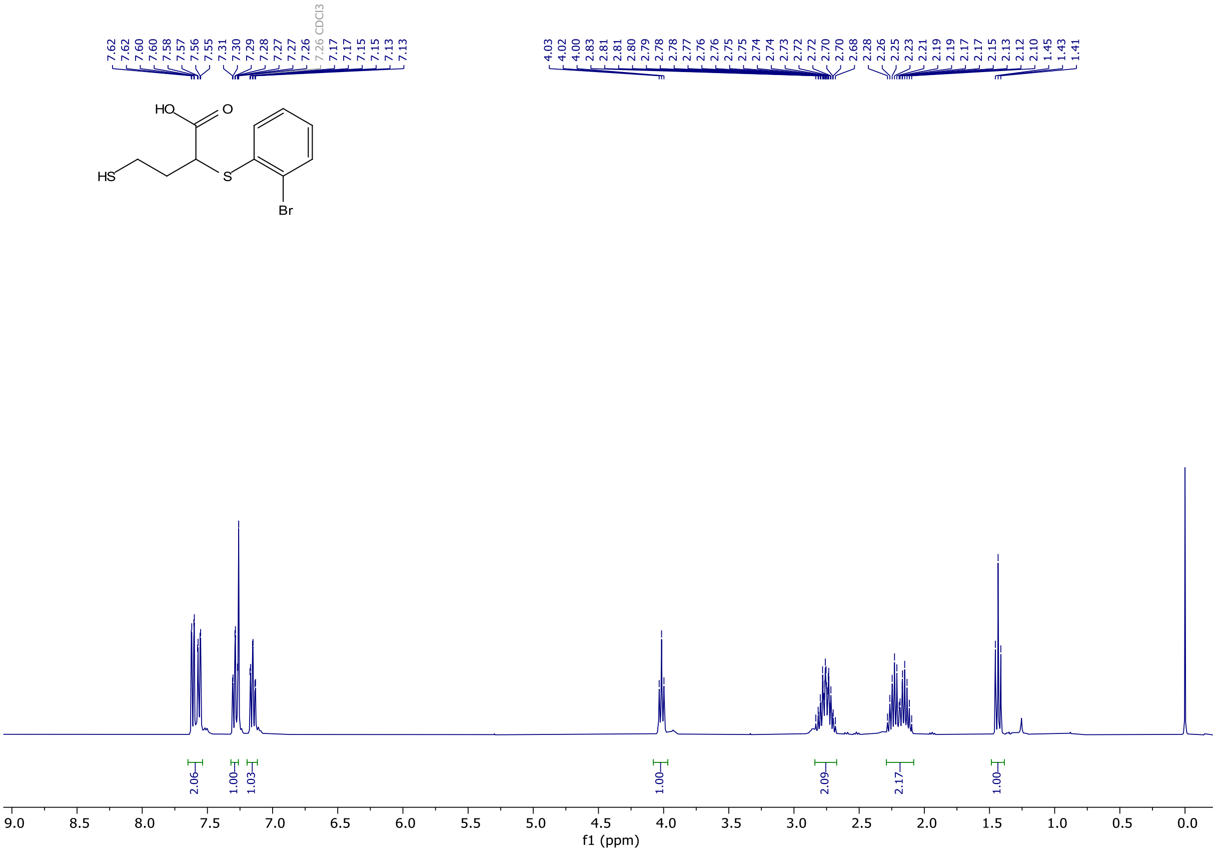

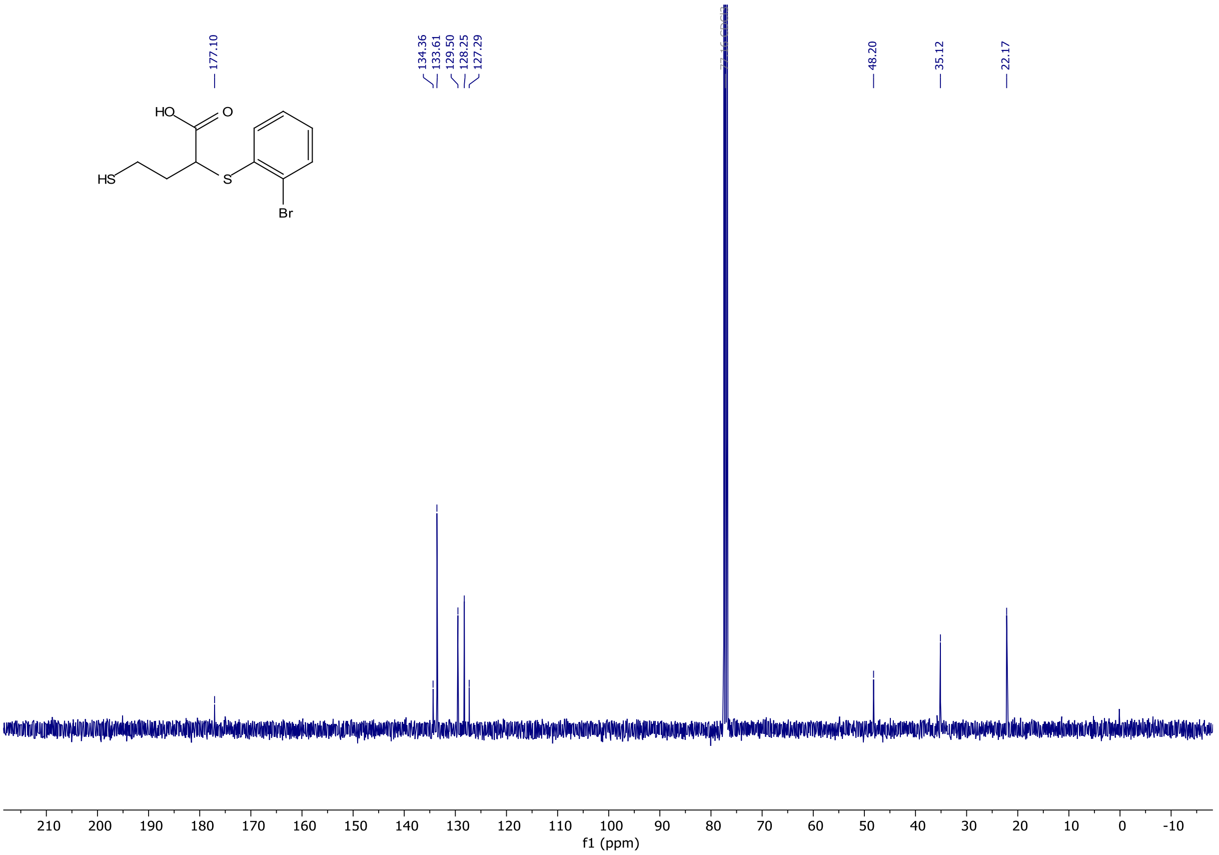

2-((3,5-Dimethylphenyl)thio)-4-mercaptobutanoic acid 6i

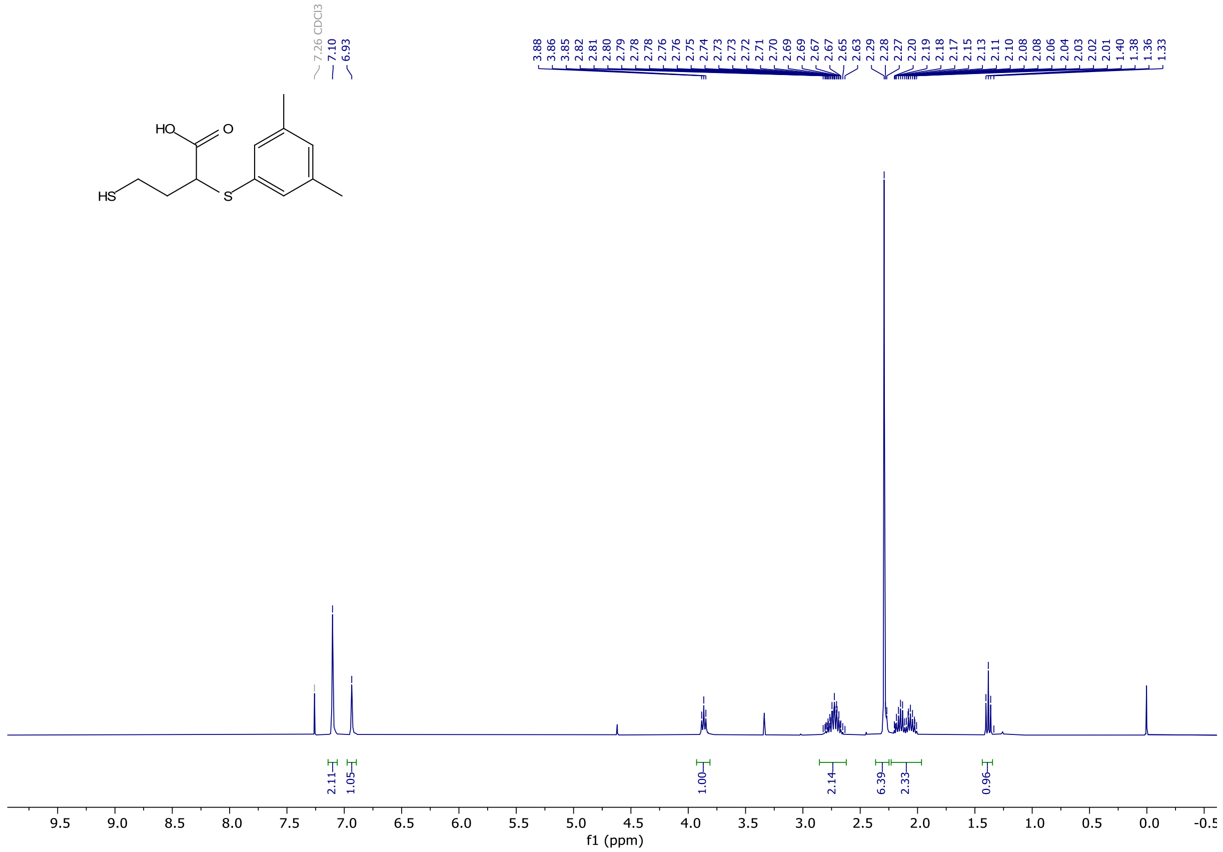

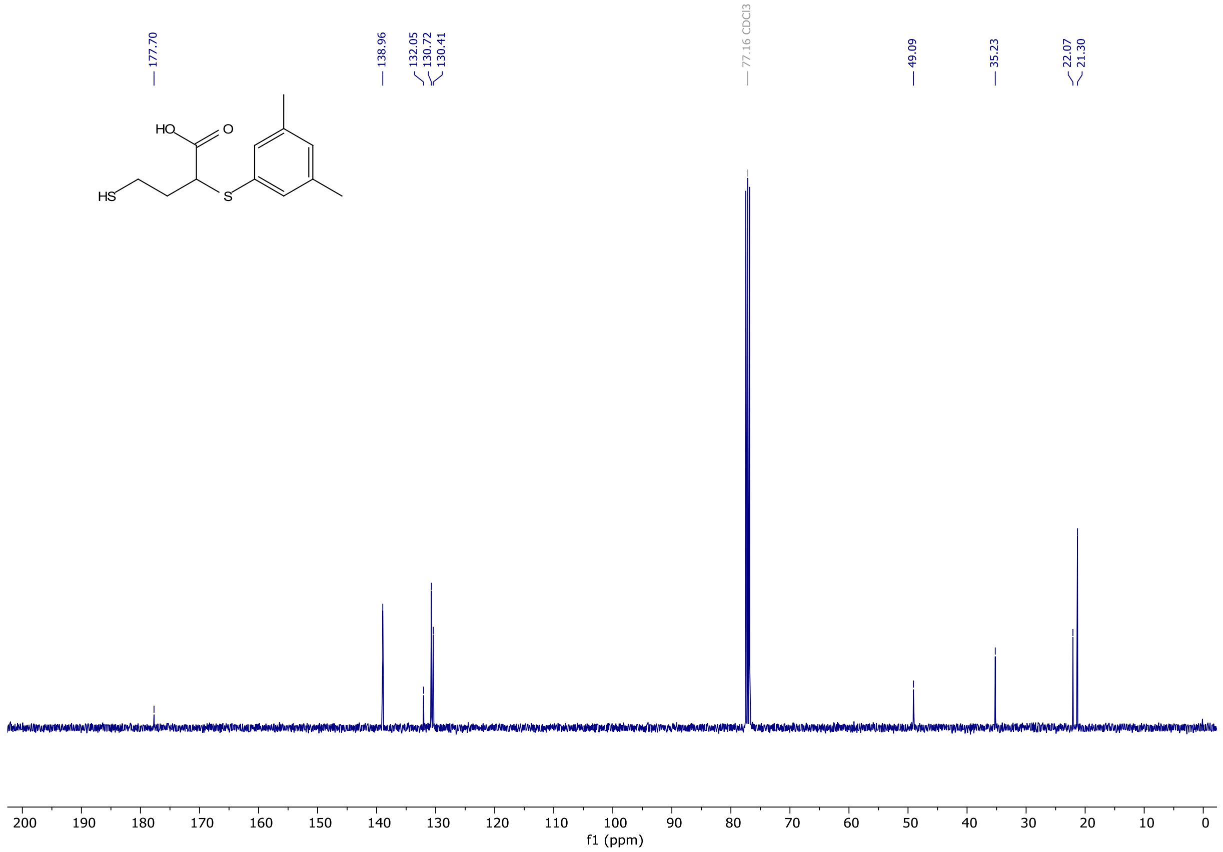

4-Mercapto-2-(naphthalen-2-ylthio)butanoic acid 6j

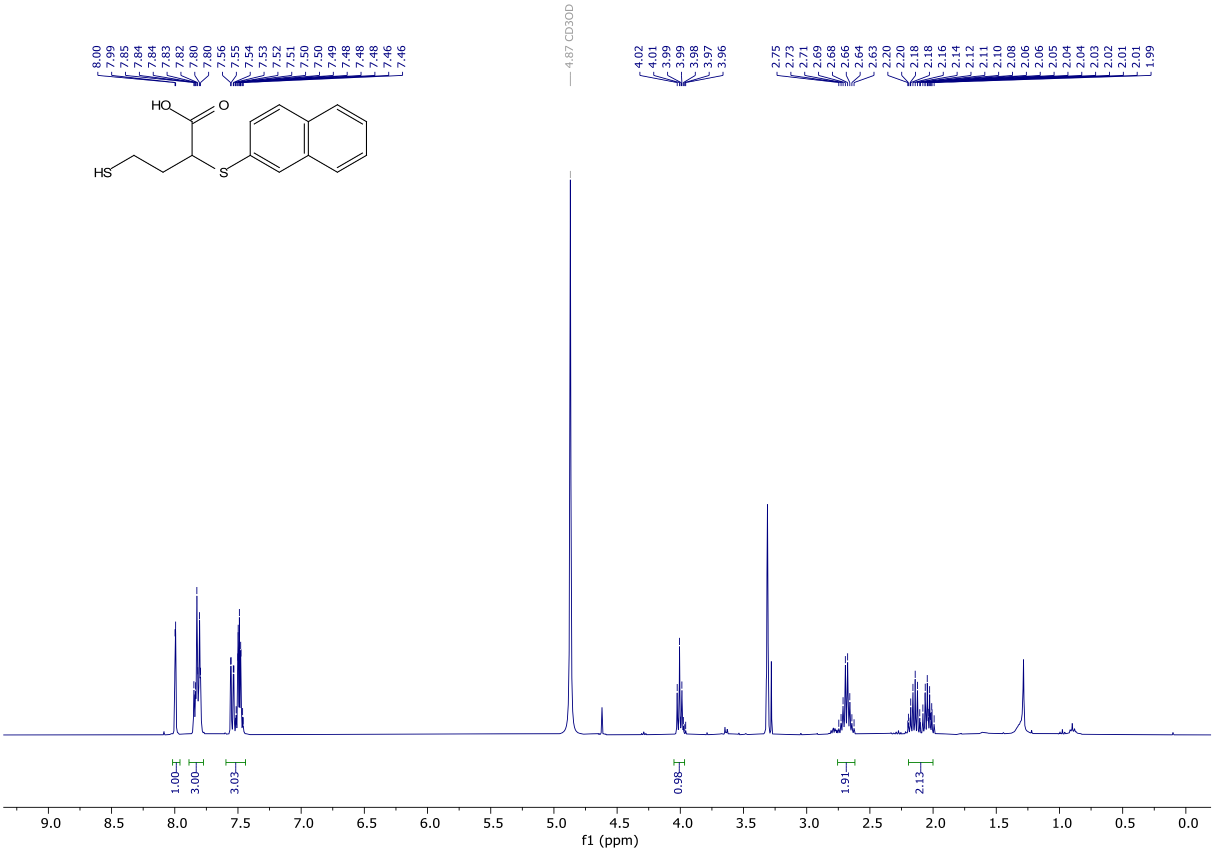

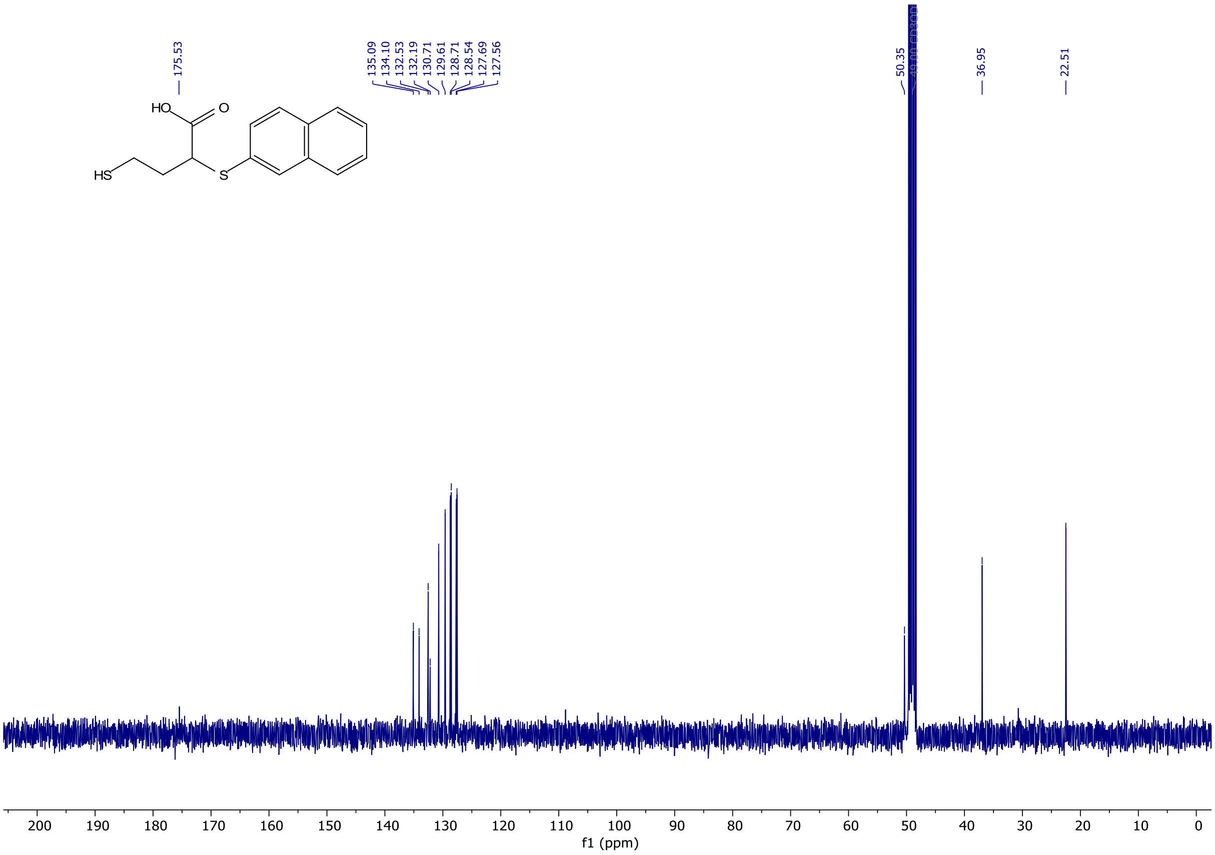

4-Mercapto-2-(propylthio)butanoic acid 6k

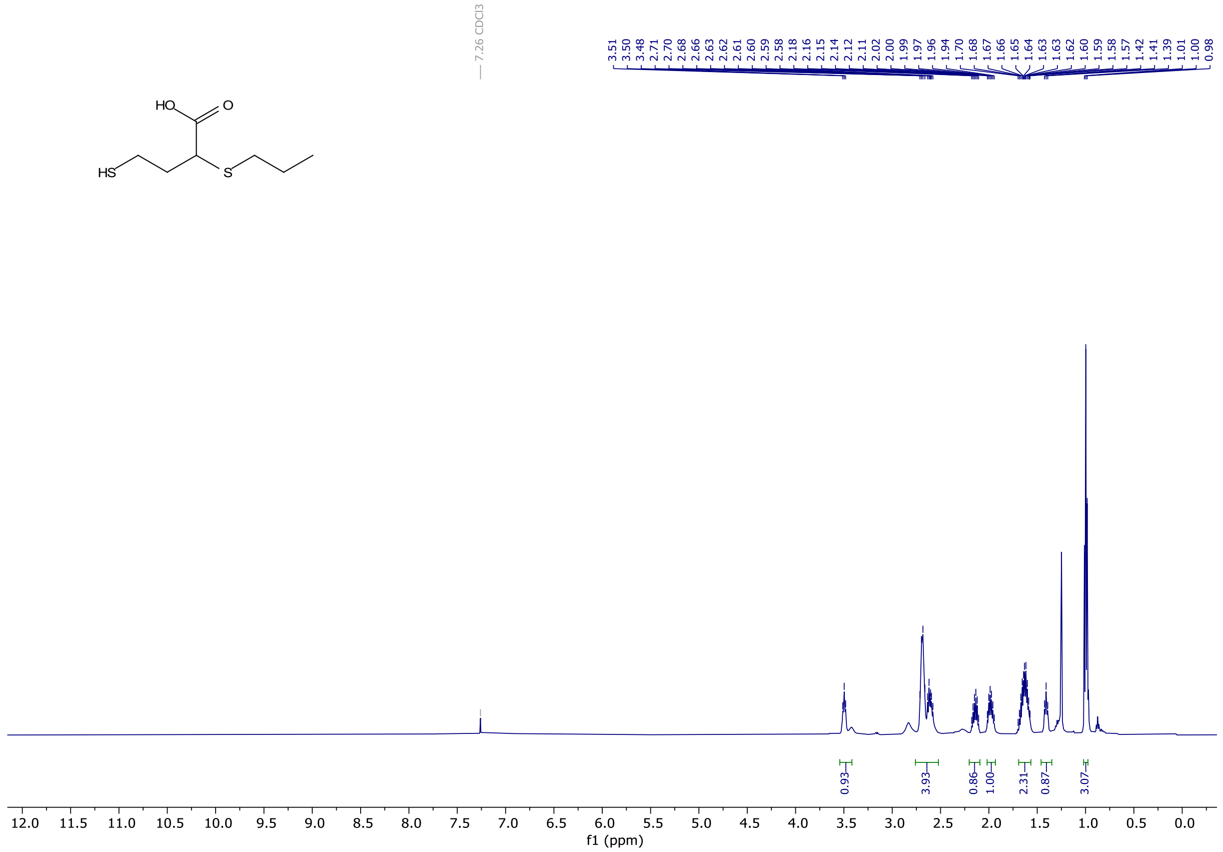

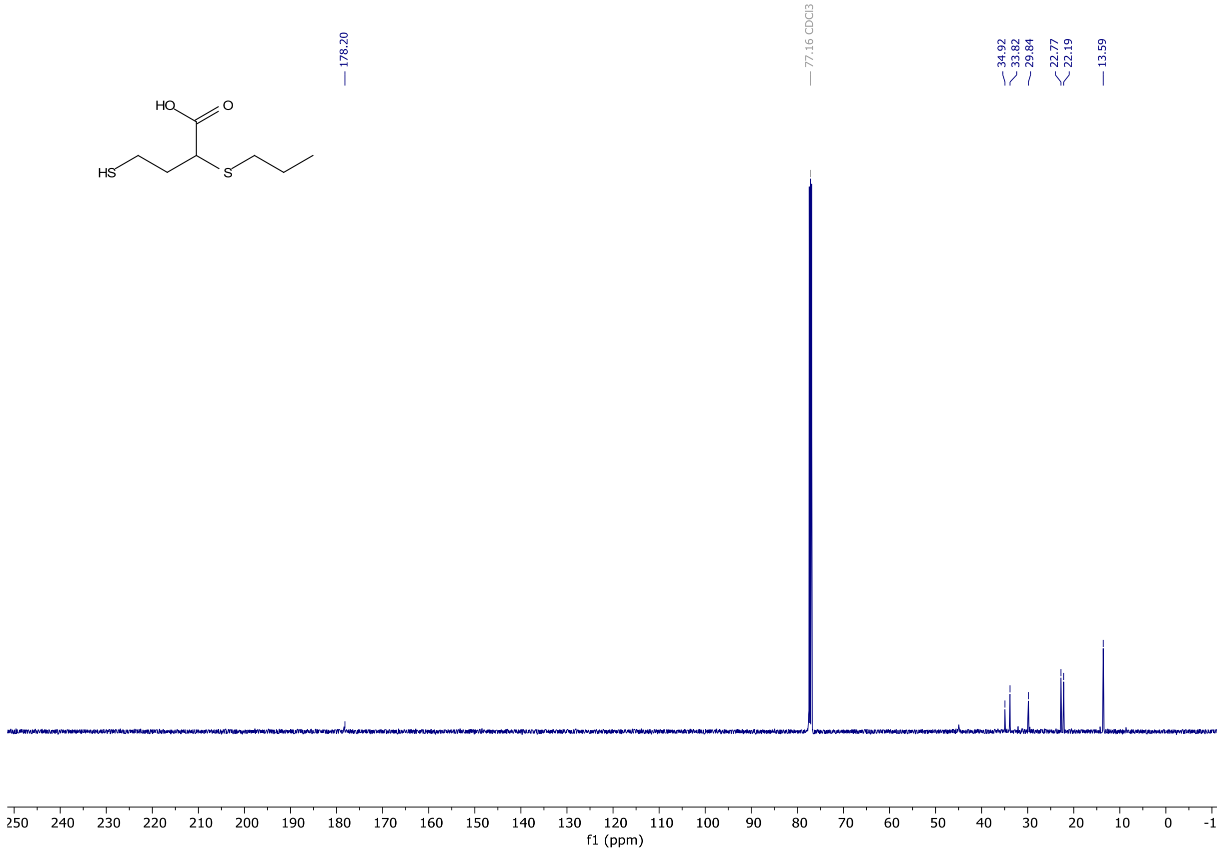

2-(Isobutylthio)-4-mercaptobutanoic acid 6l

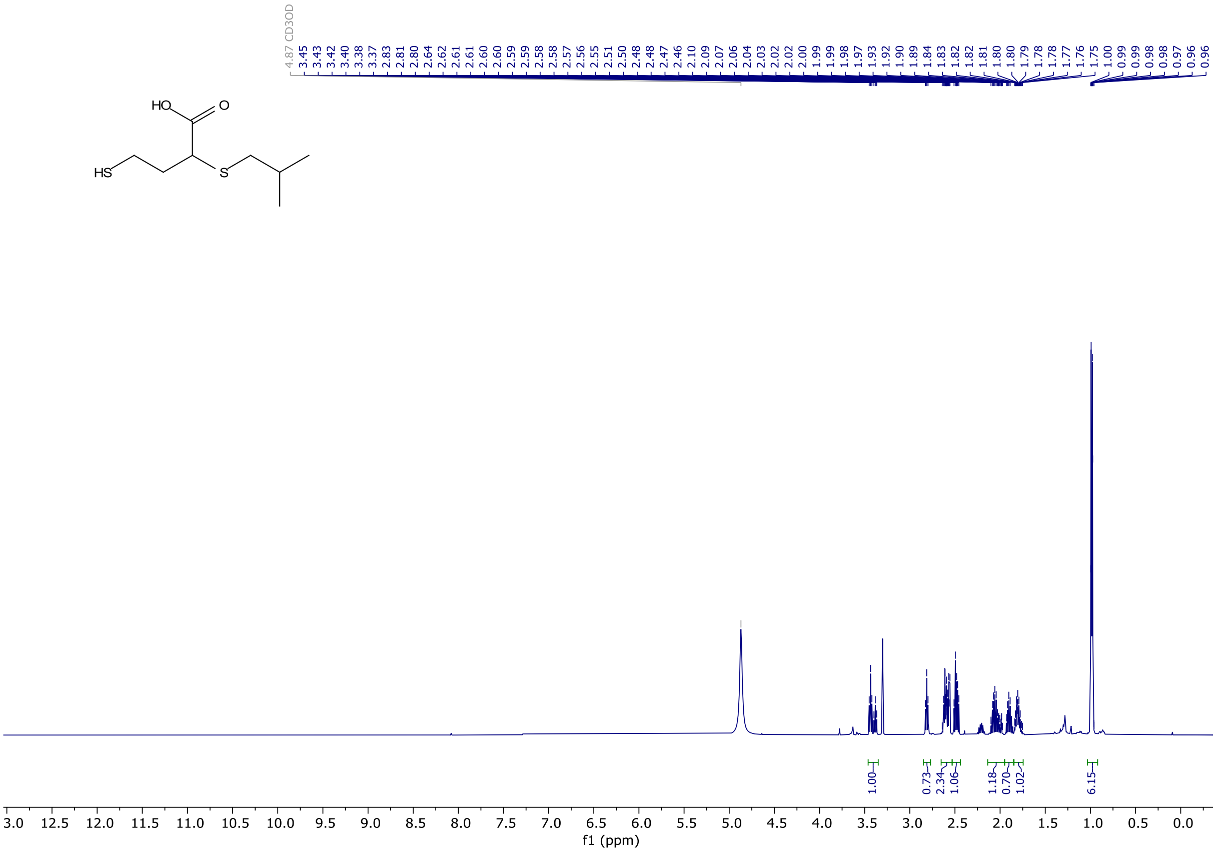

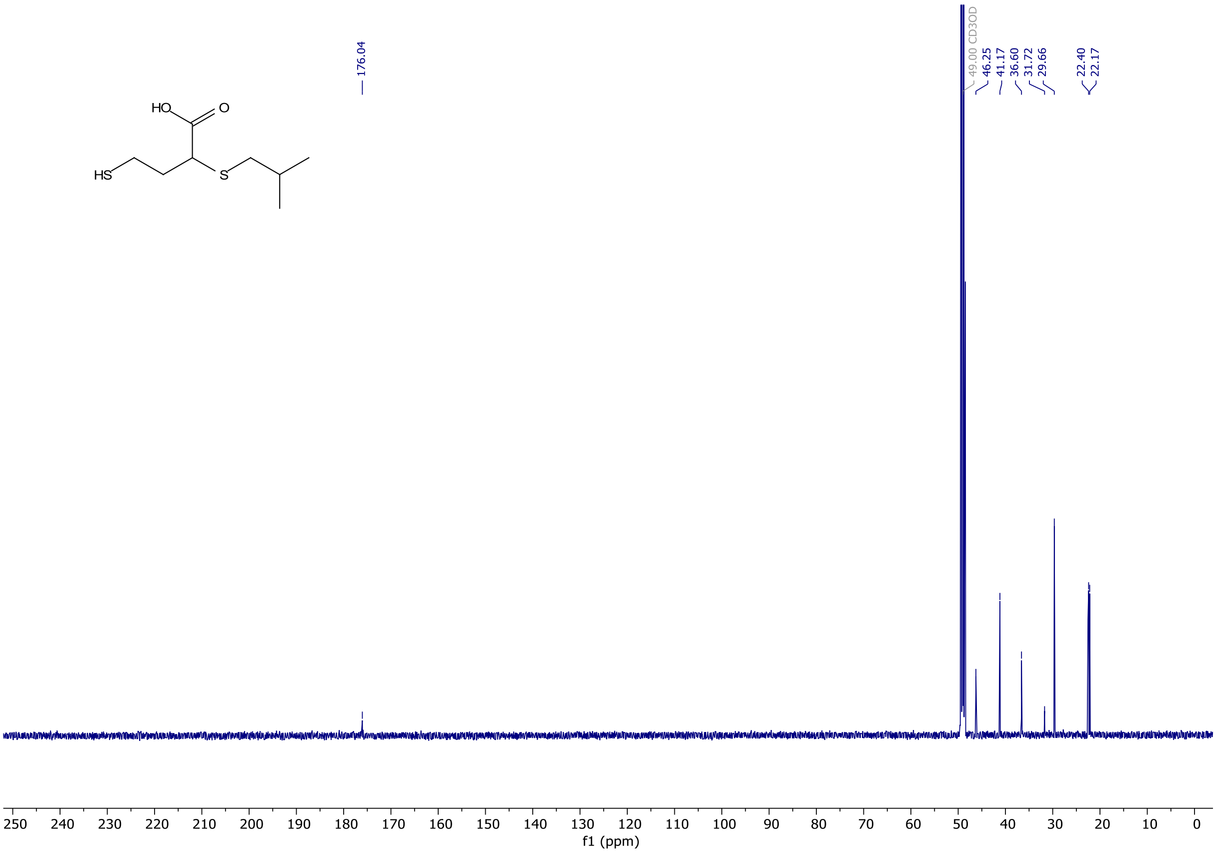

2-(Cyclohexylthio)-4-mercaptobutanoic acid 6m

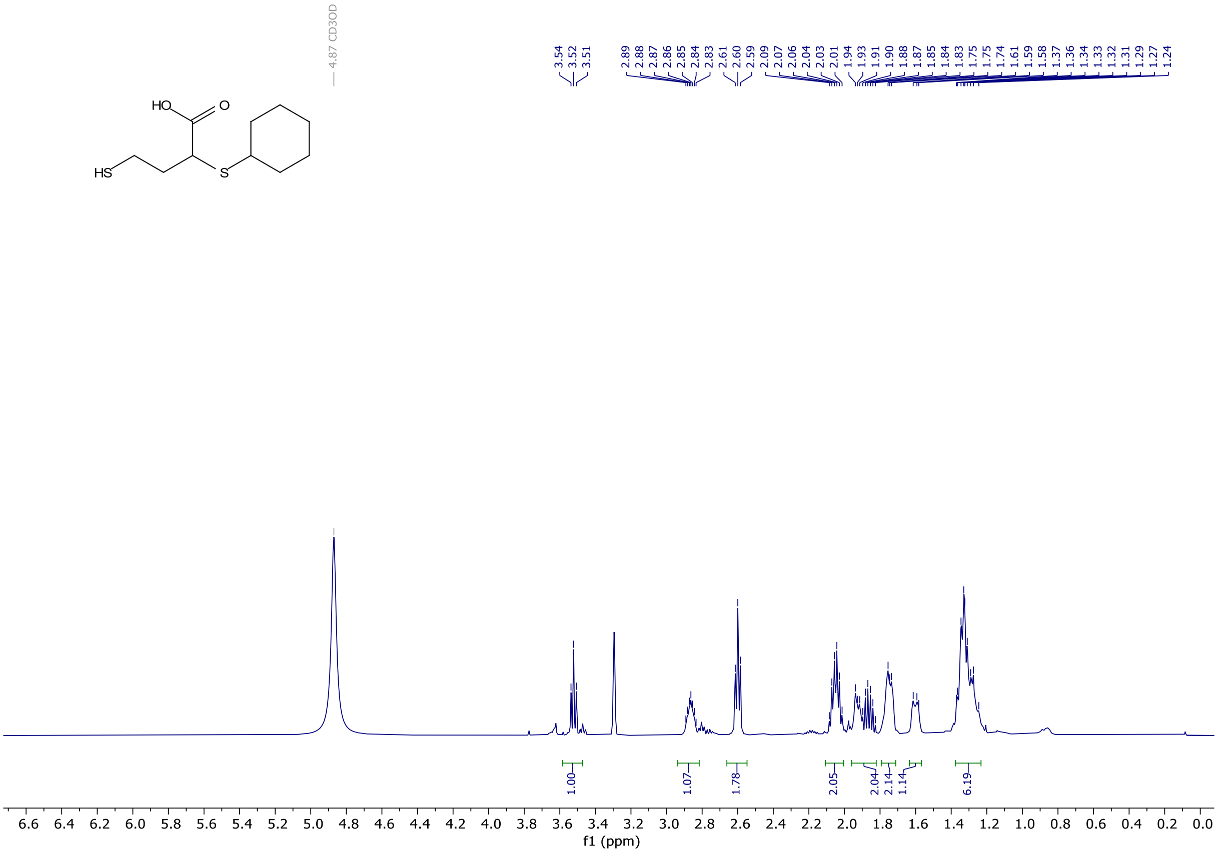

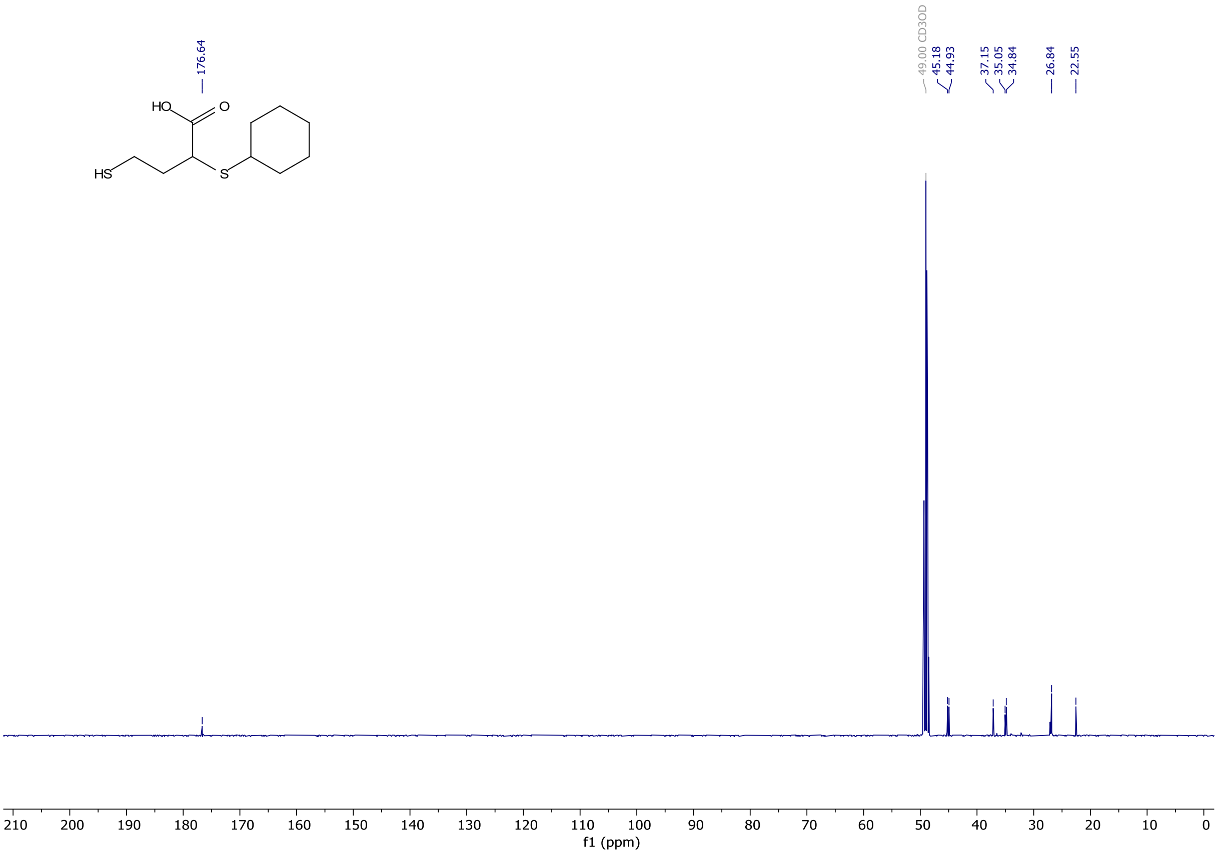

2-(Benzylthio)-4-mercaptobutanoic acid 6n

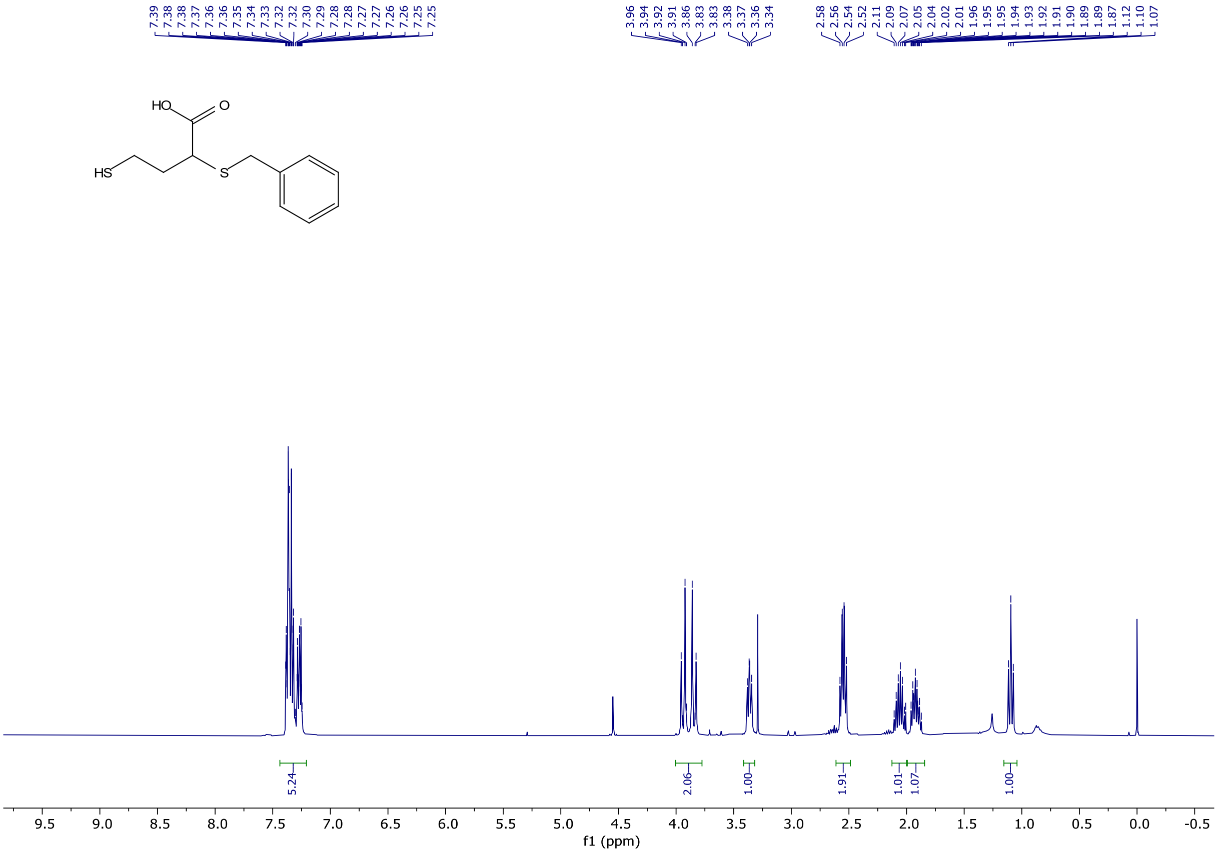

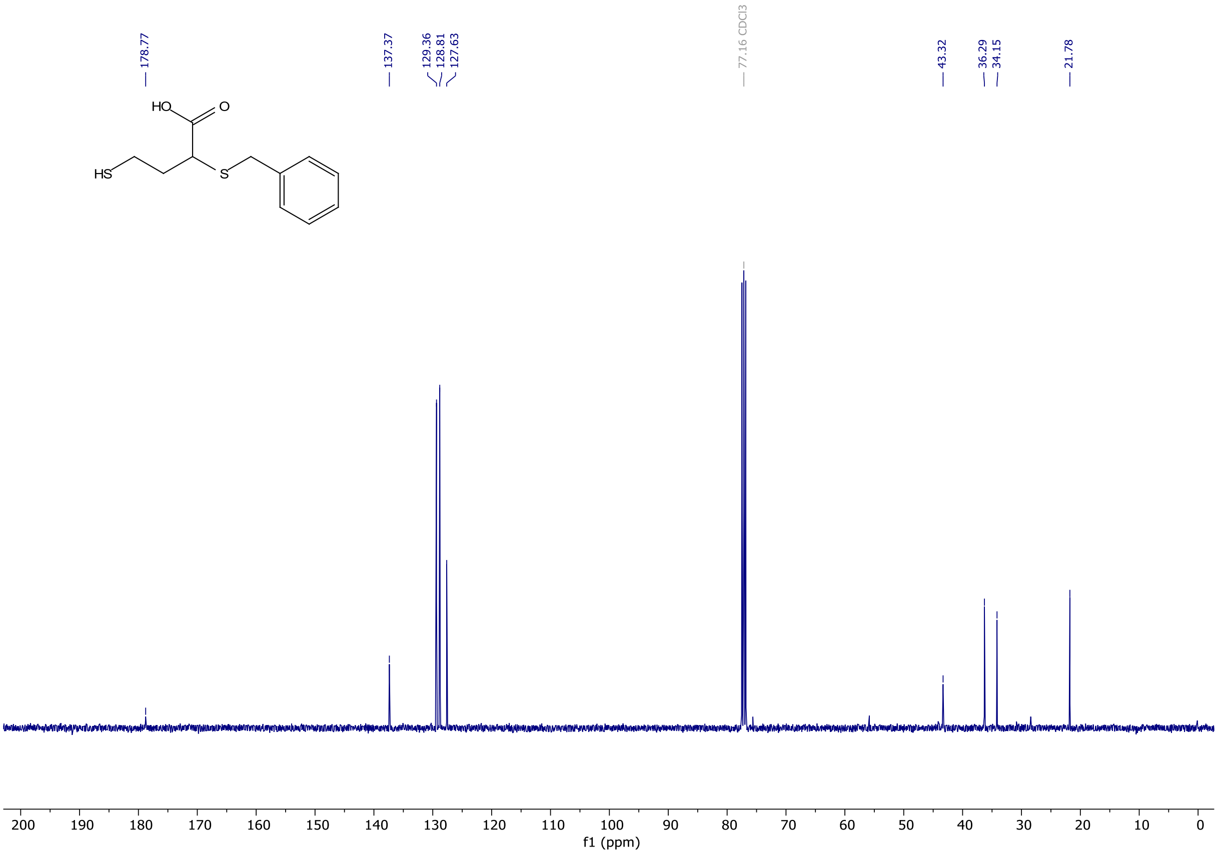

4-Mercapto-2-(phenethylthio)butanoic acid 6o

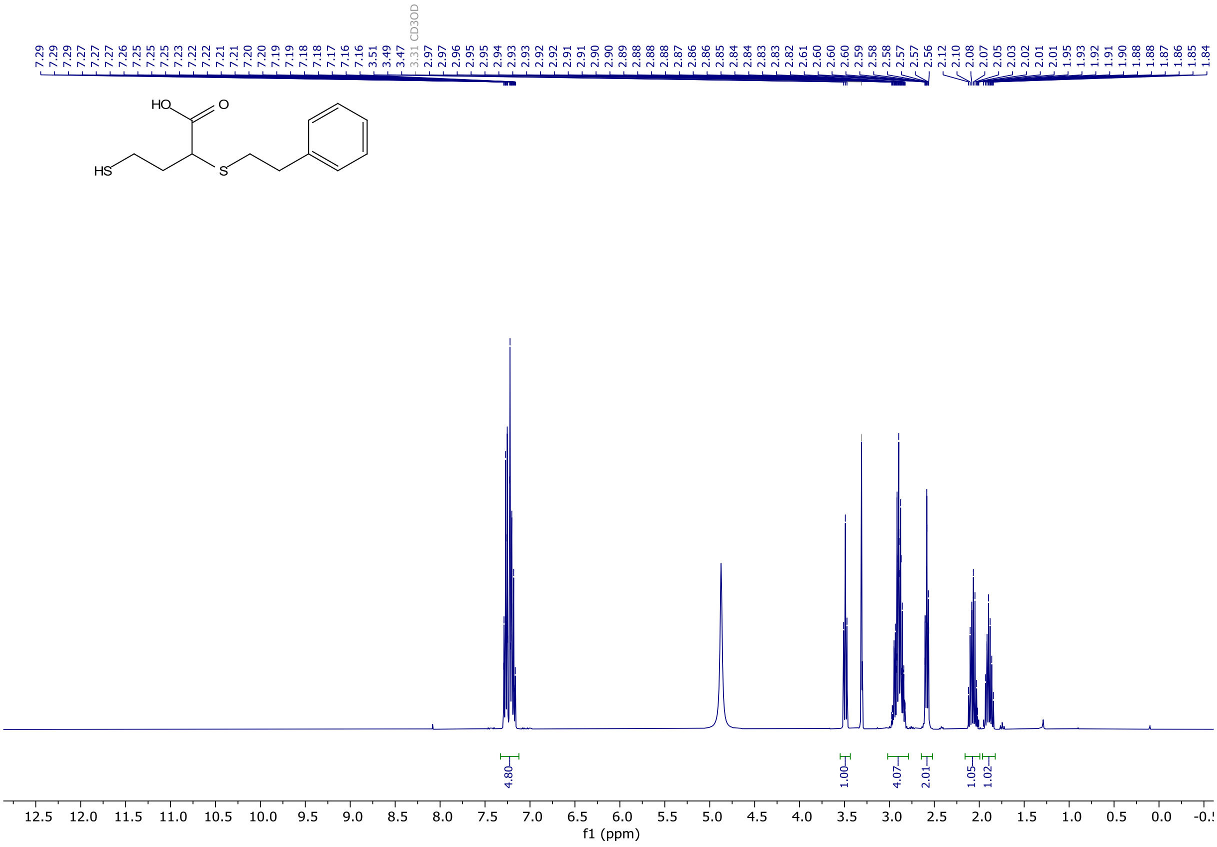

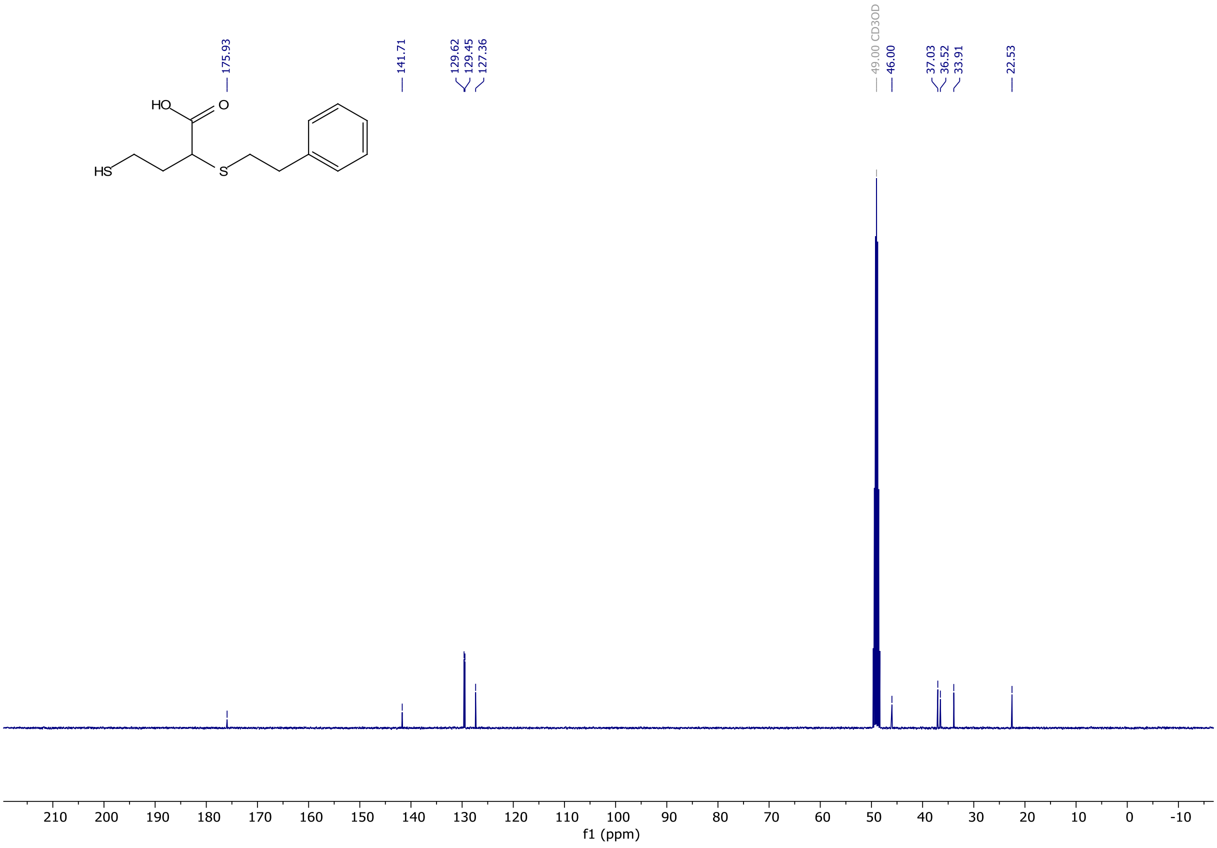

2-((Furan-2-ylmethyl)thio)-4-mercaptobutanoic acid 6p

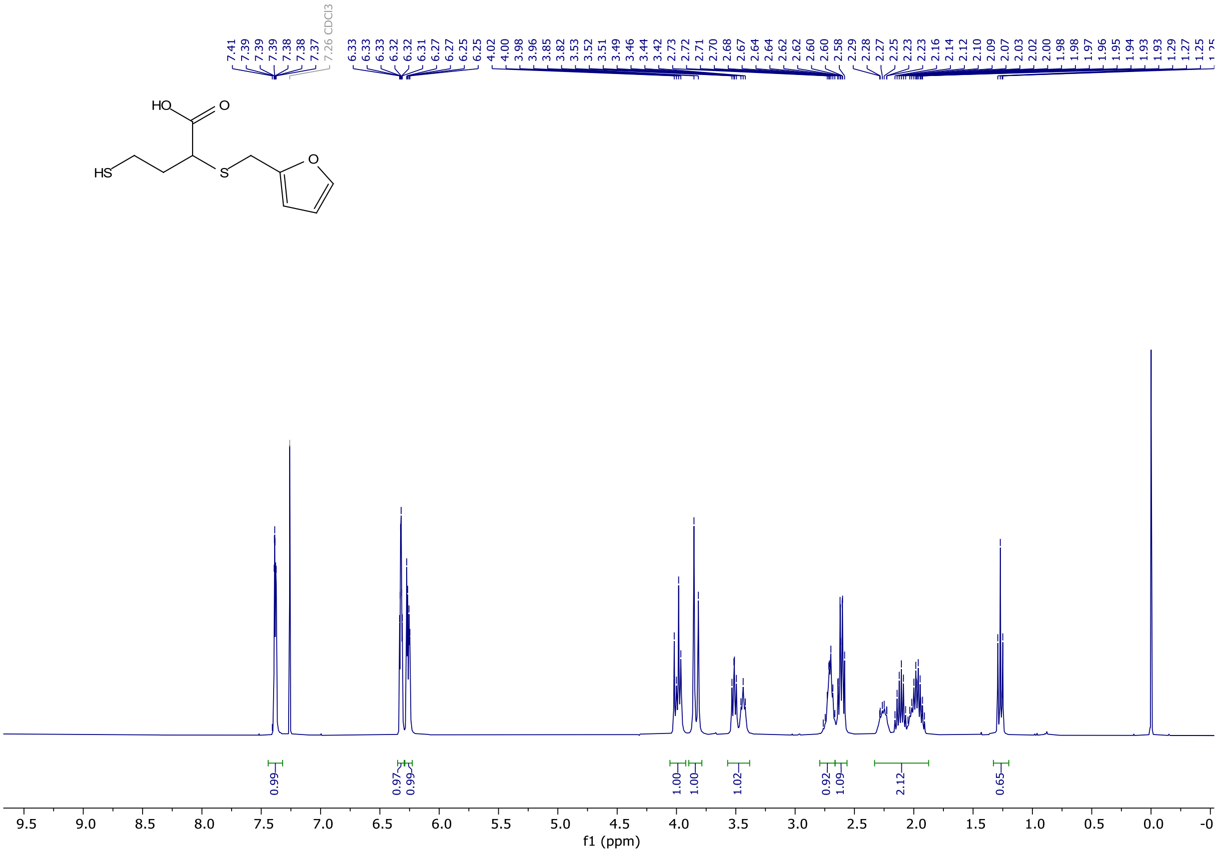

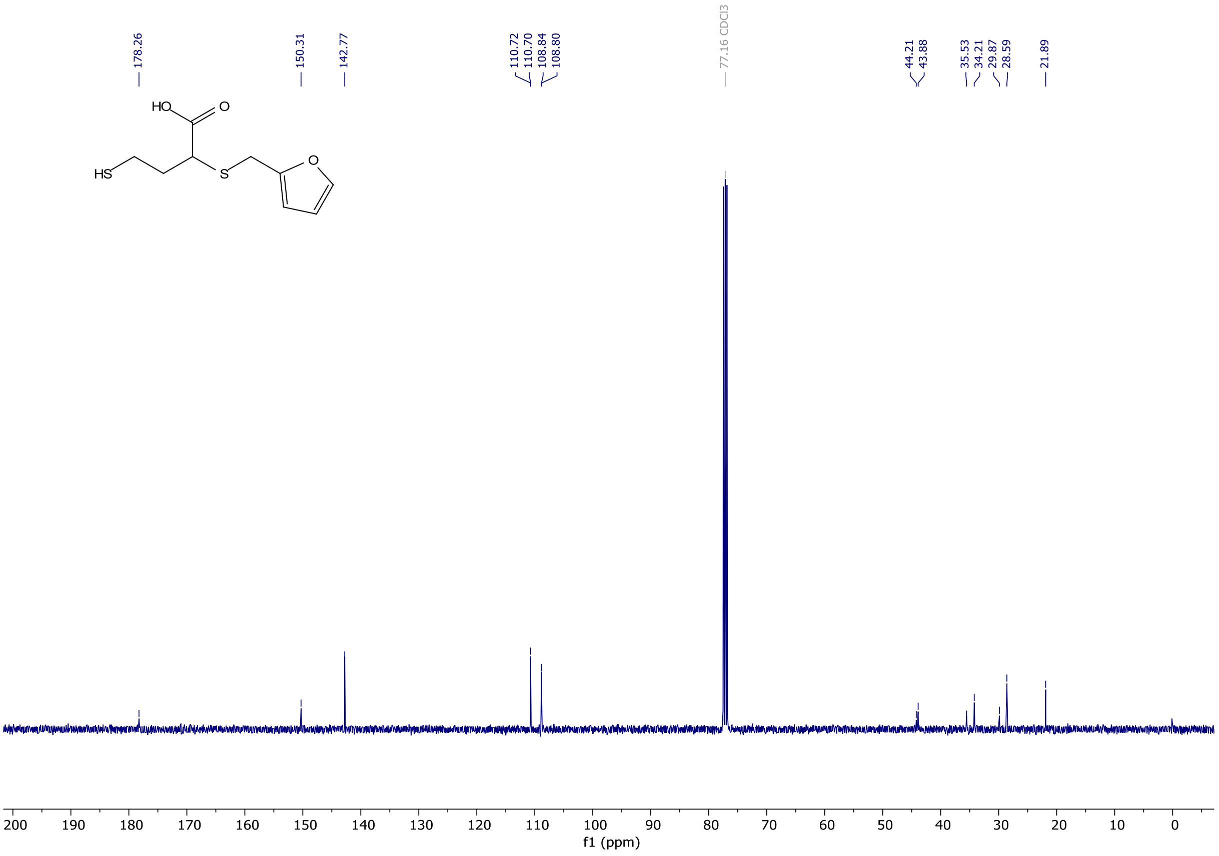

3-(Phenylthio)dihydrofuran-2(3H)-one 8a

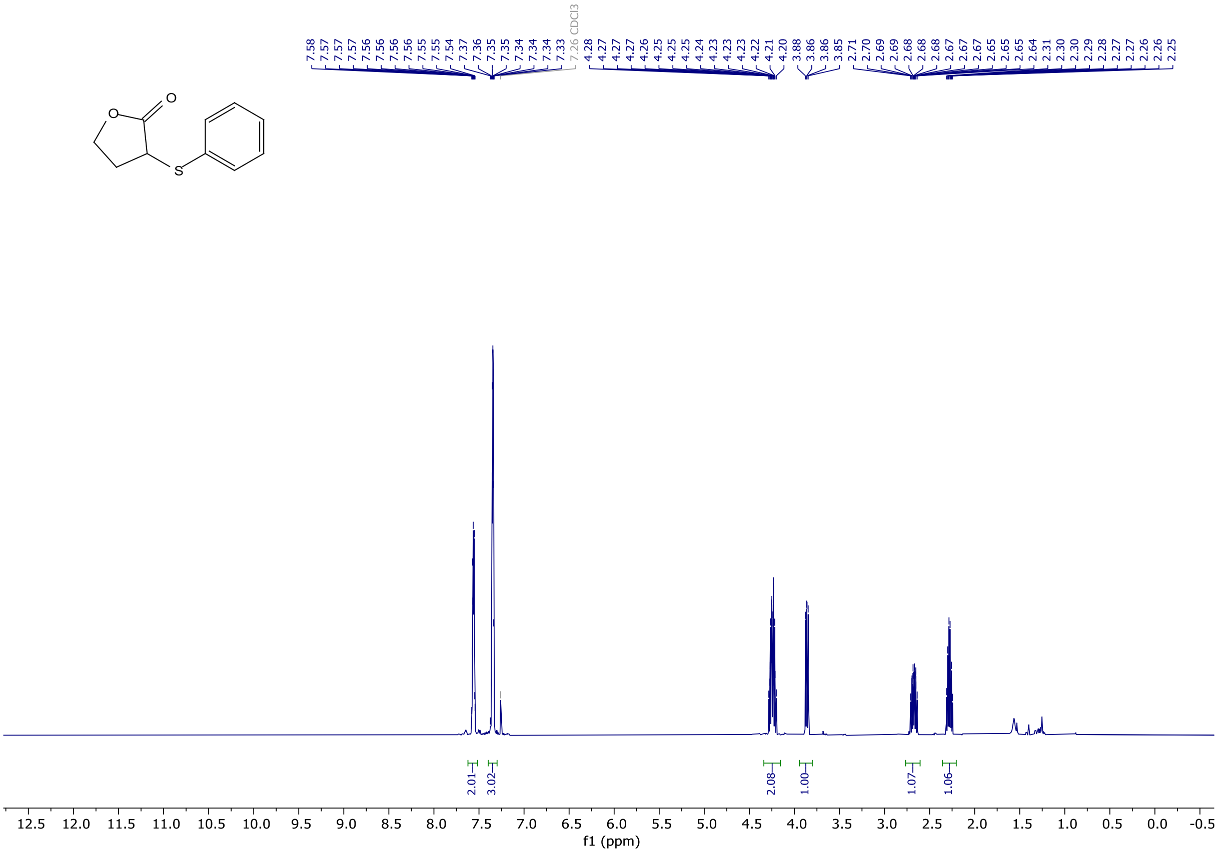

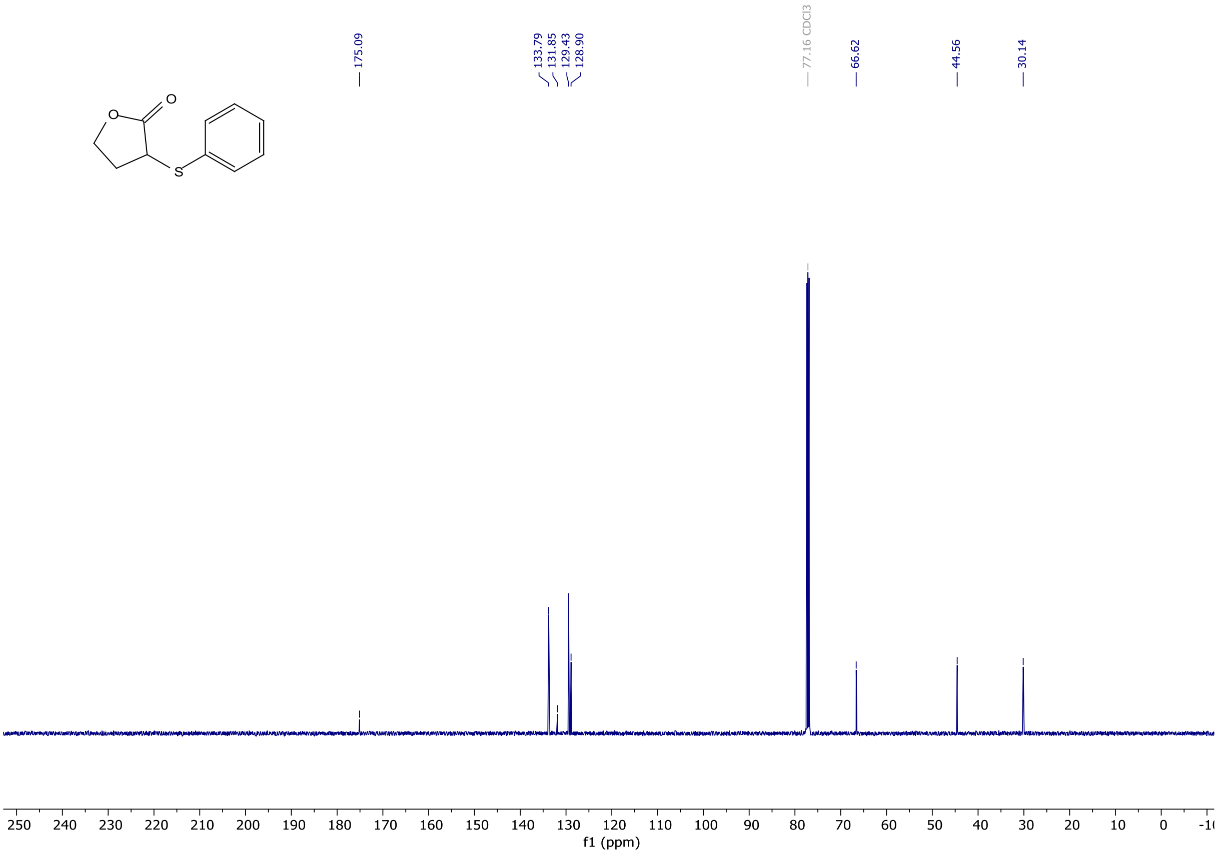

3-((4-Chlorophenyl)thio)dihydrofuran-2(3H)-one 8b

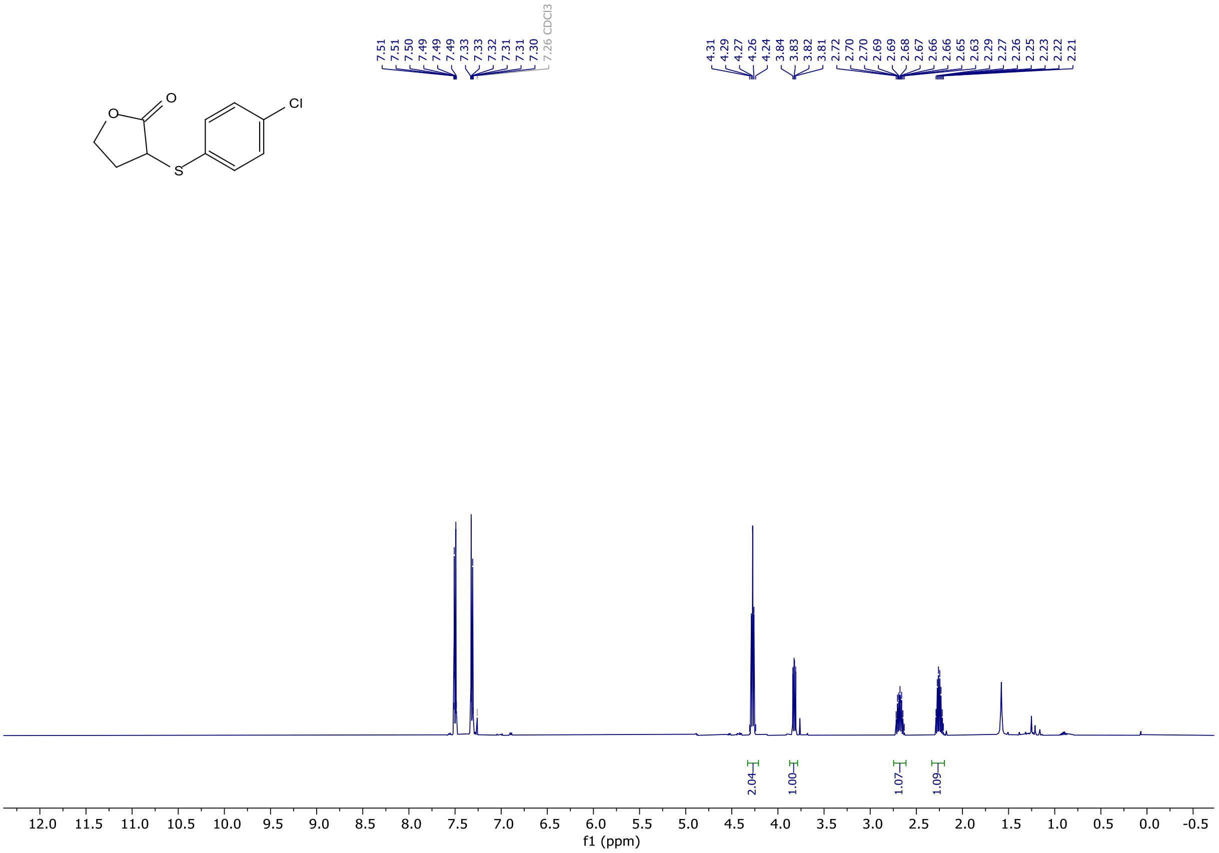

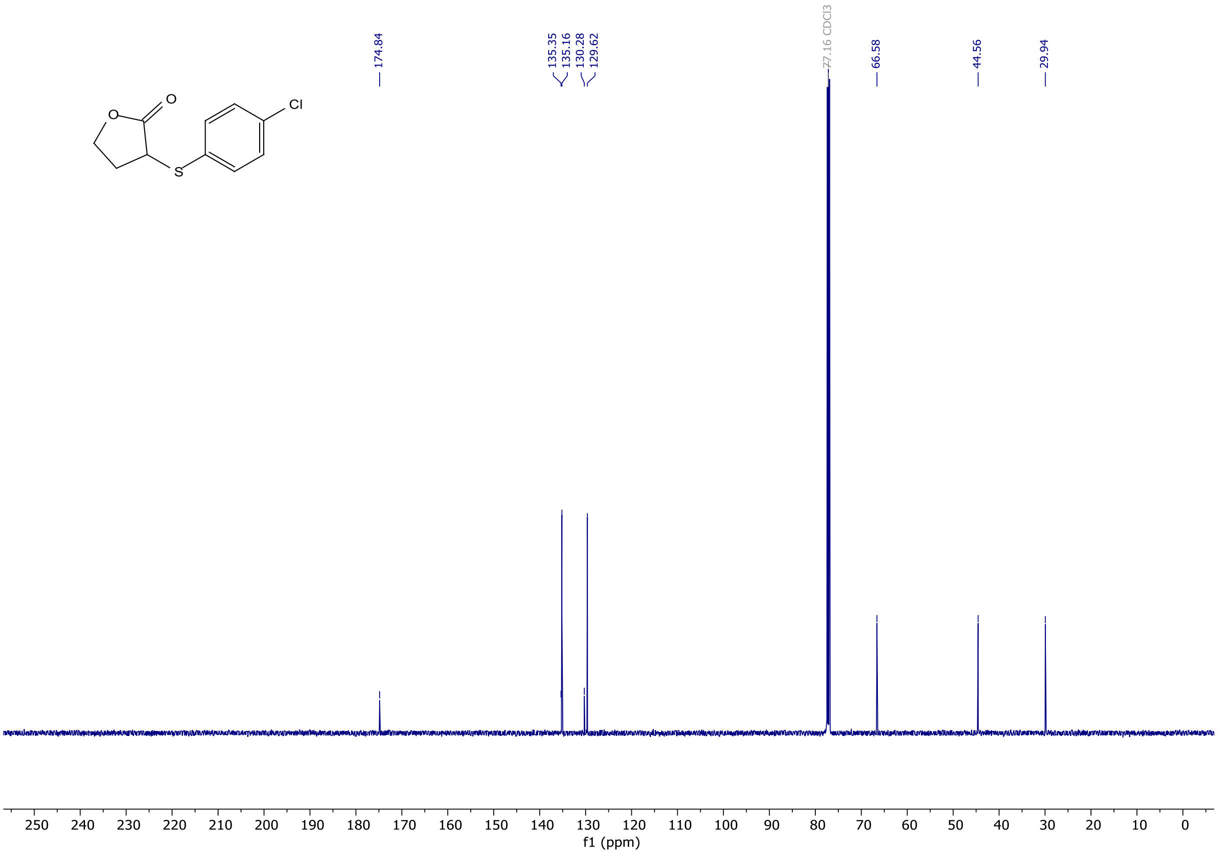

3-(Propylthio)dihydrofuran-2(3H)-one 8k

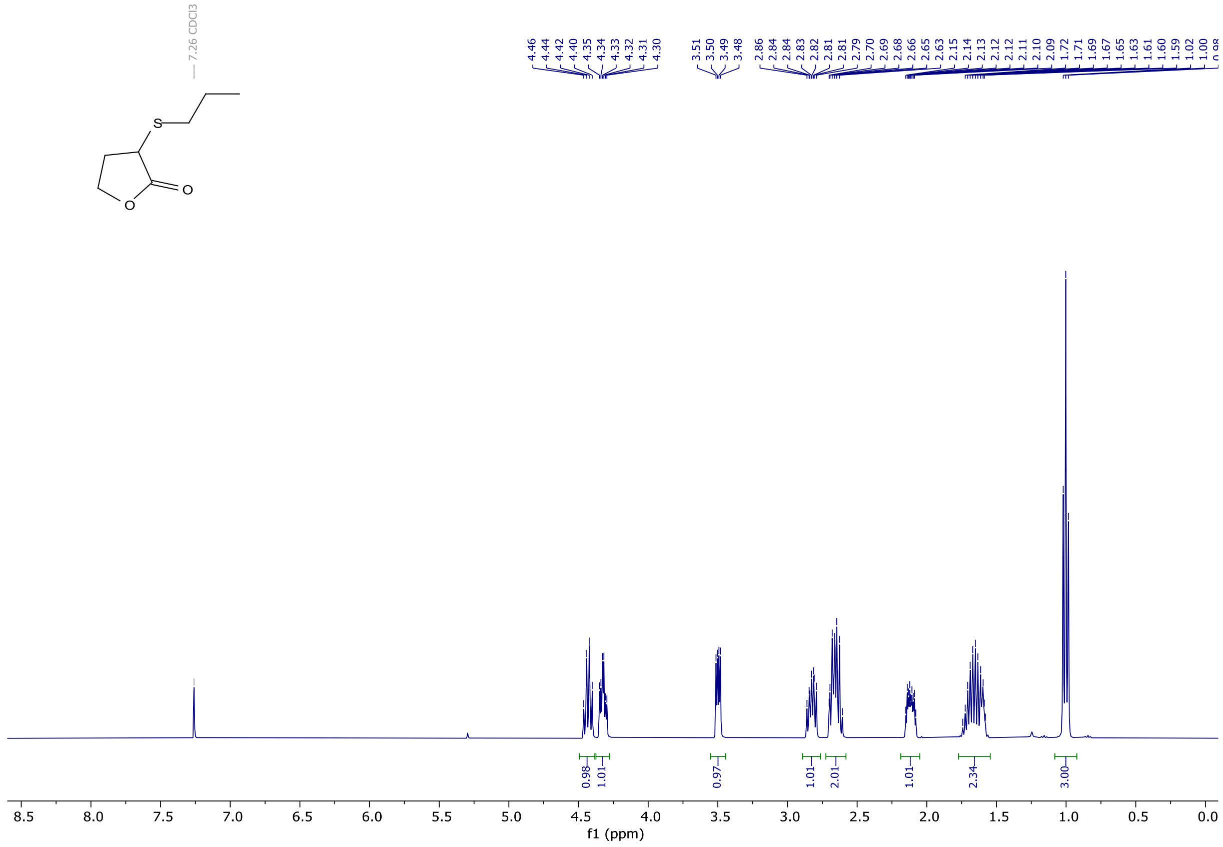

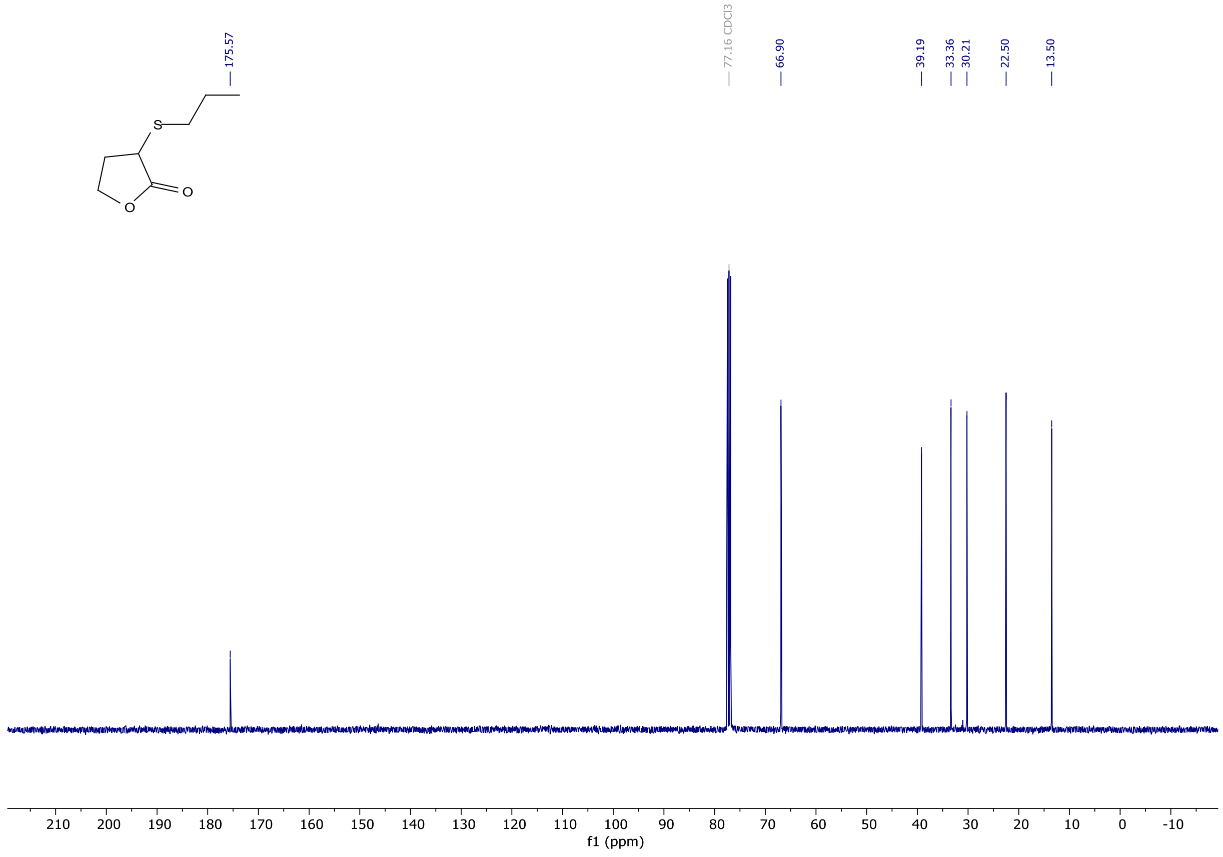

3-(Benzylthio)dihydrofuran-2(3H)-one 8n

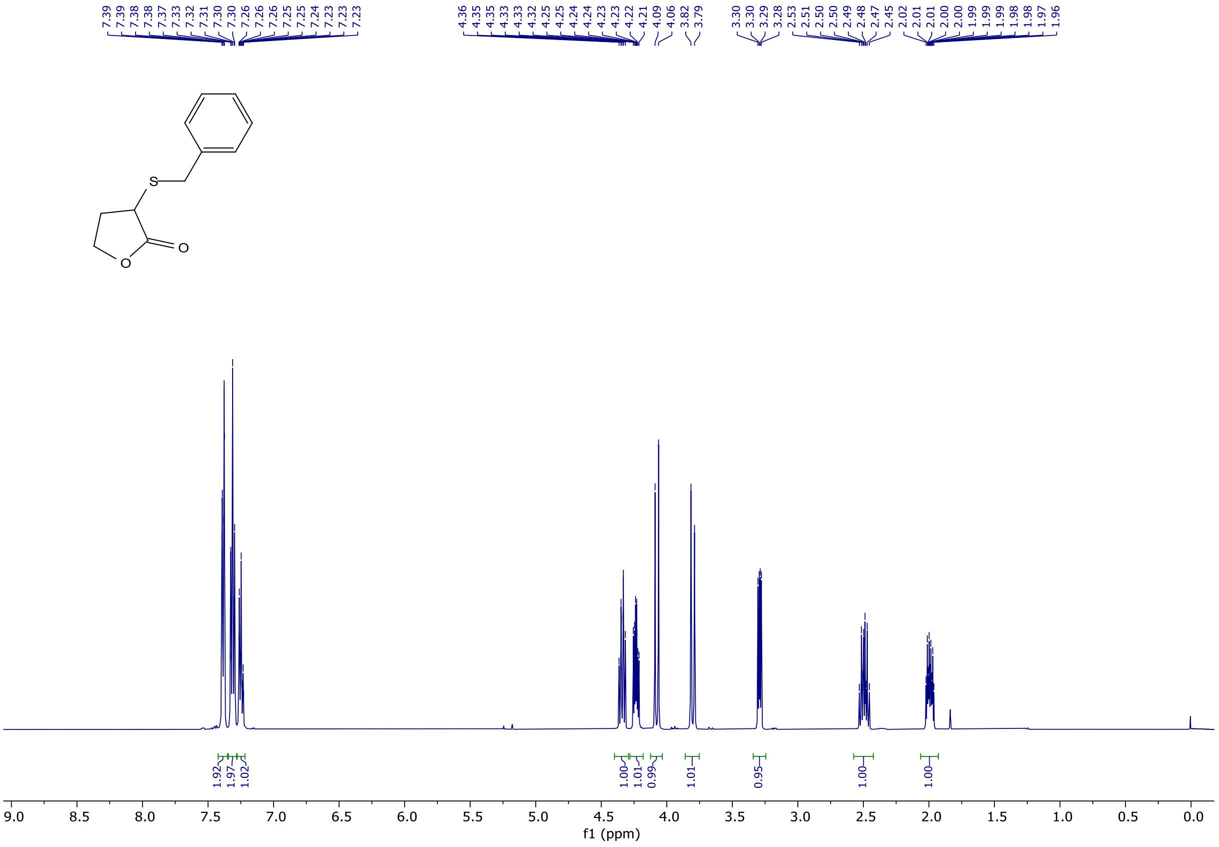

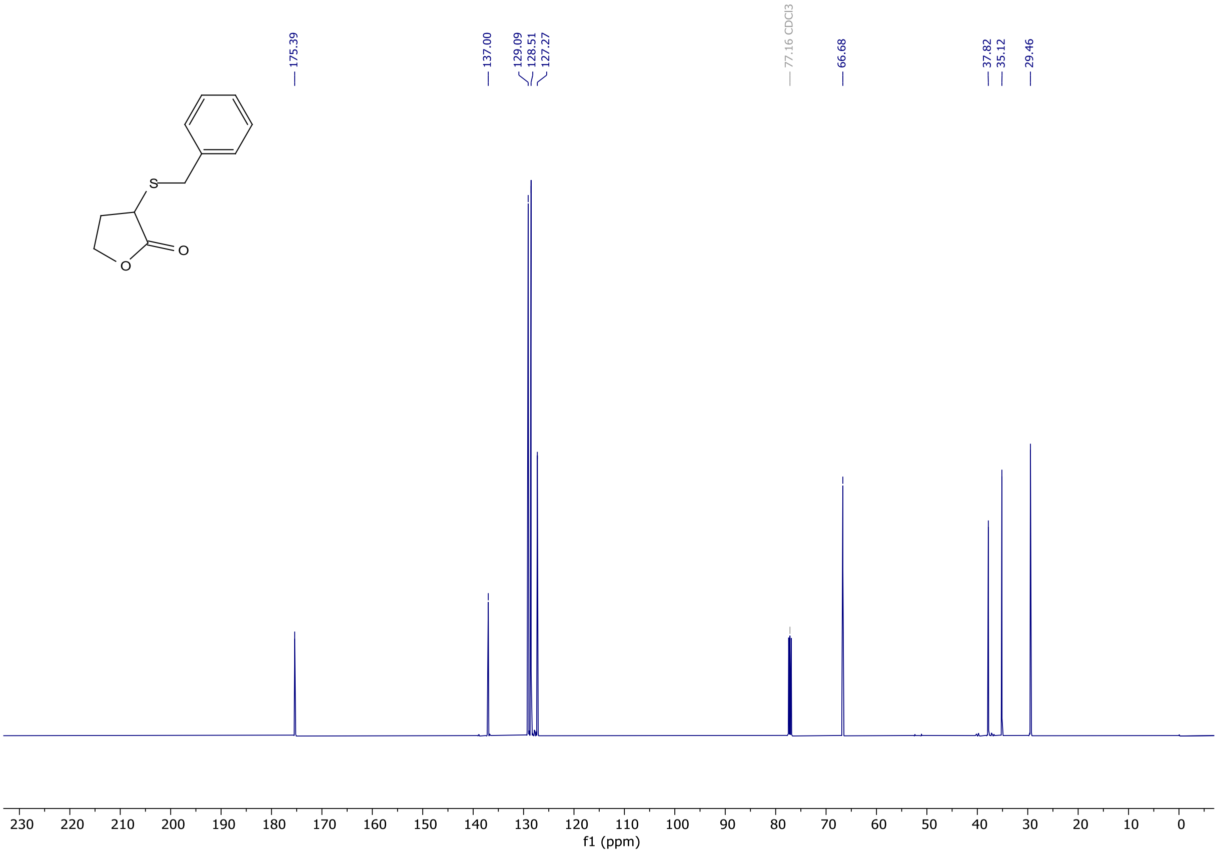

4-Hydroxy-2-(phenylthio)butanoic acid 9a

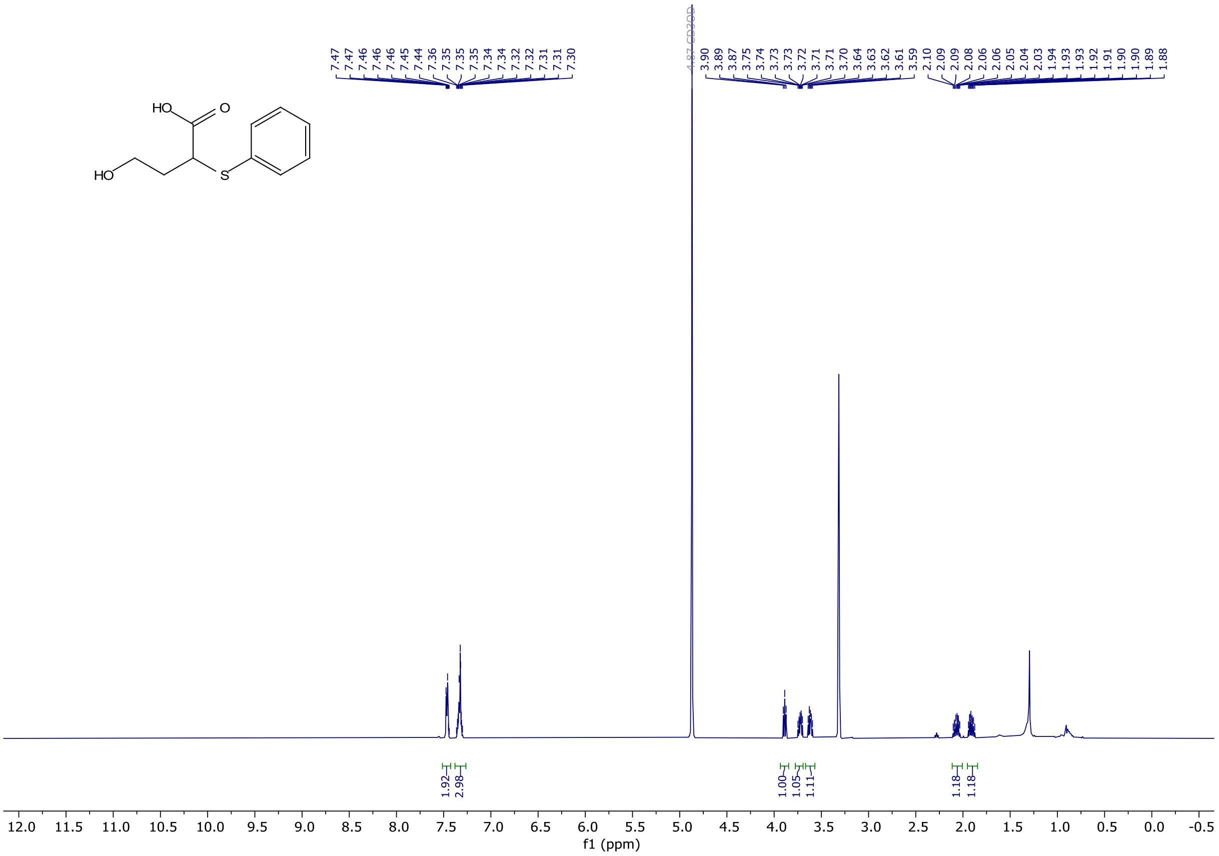

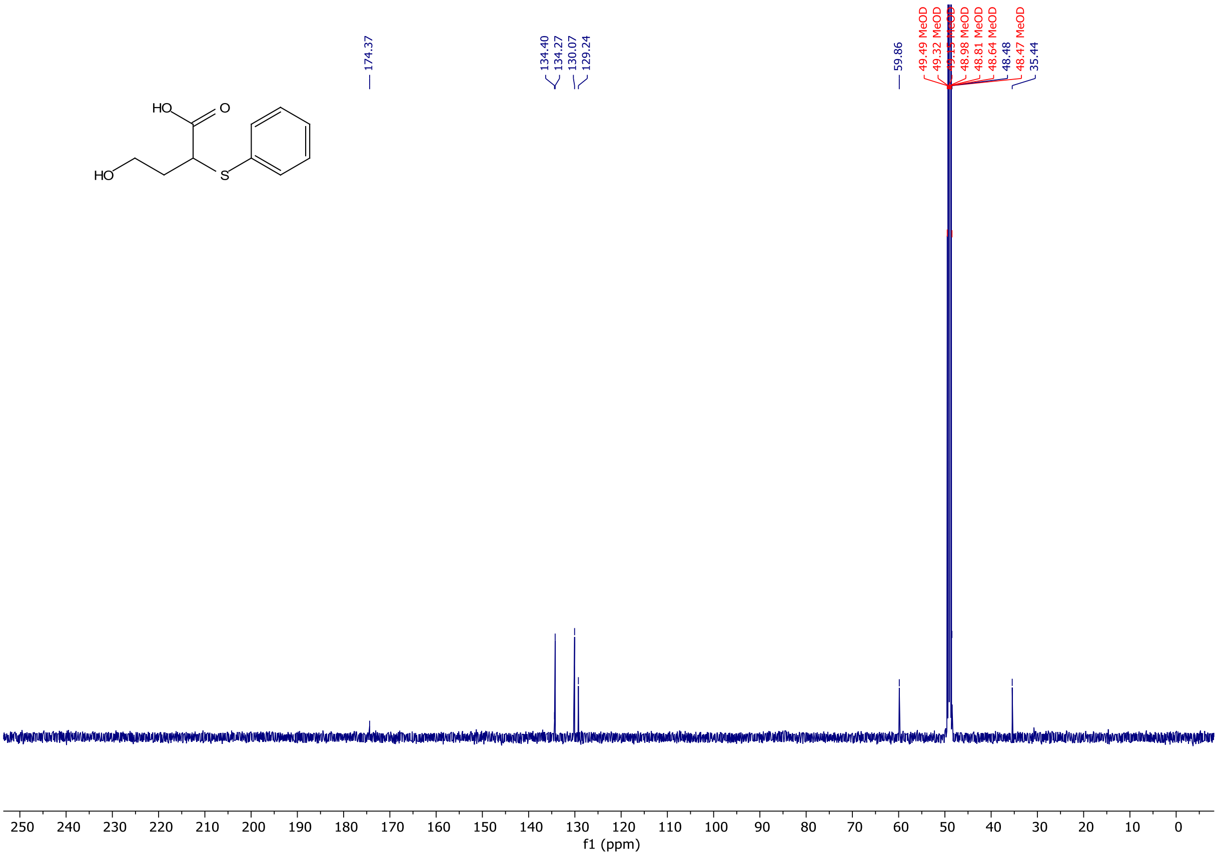

2-((4-Chlorophenyl)thio)-4-hydroxybutanoic acid 9b

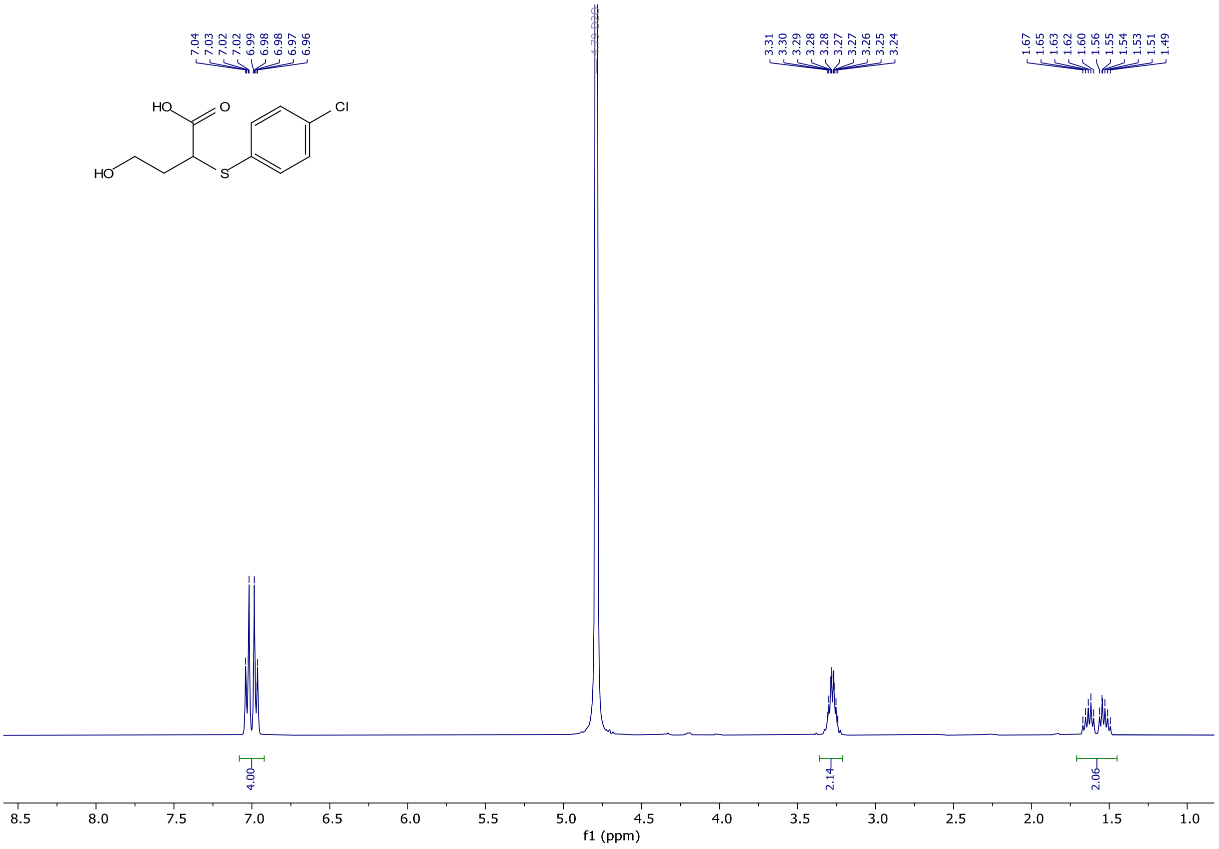

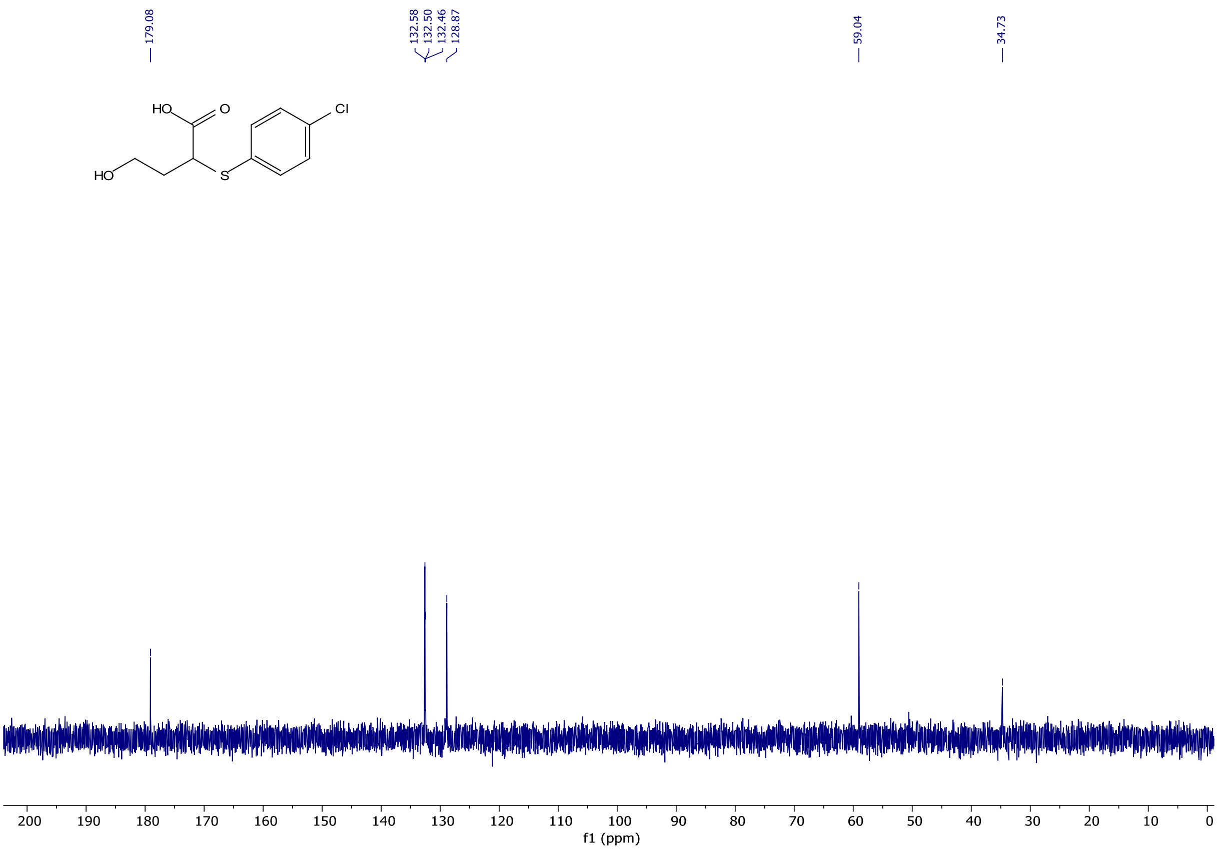

4-Hydroxy-2-(propylthio)butanoic acid 9k

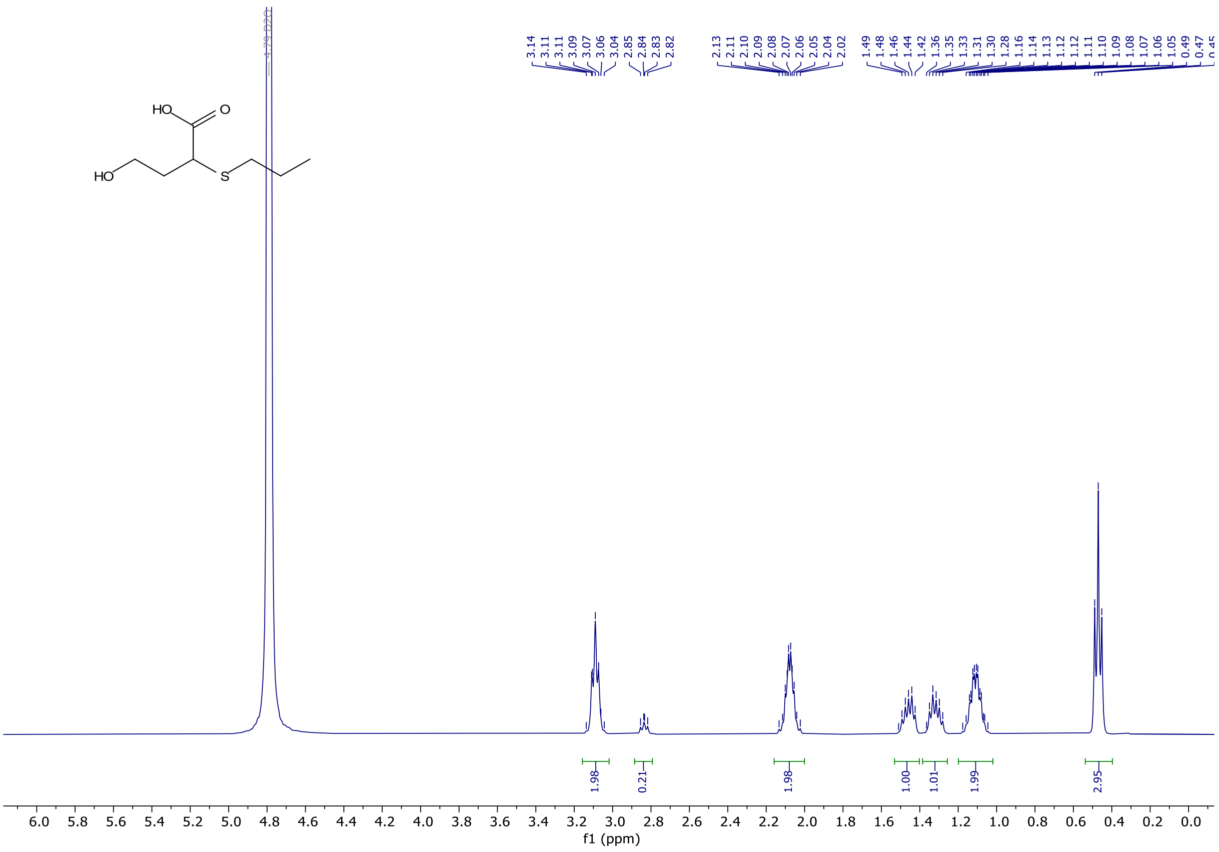

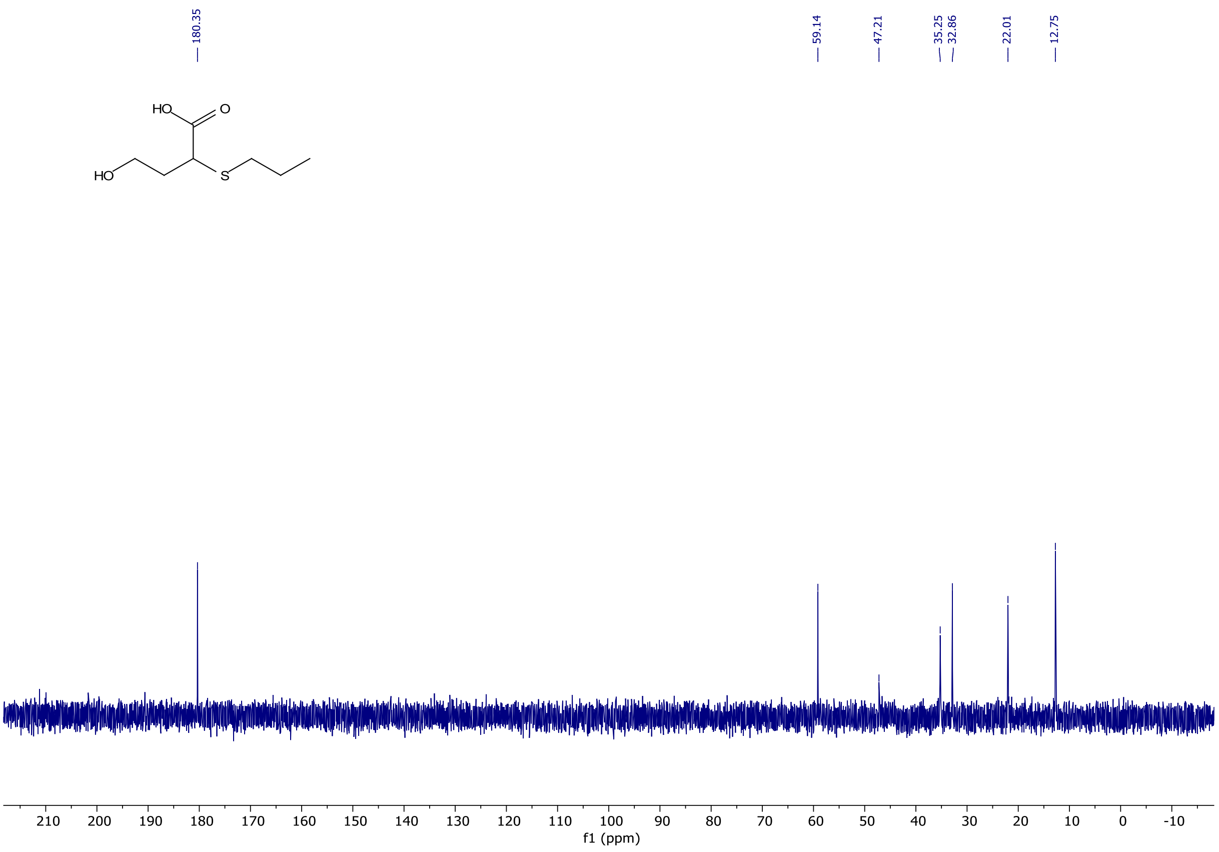

2-(Benzylthio)-4-hydroxybutanoic acid 9n

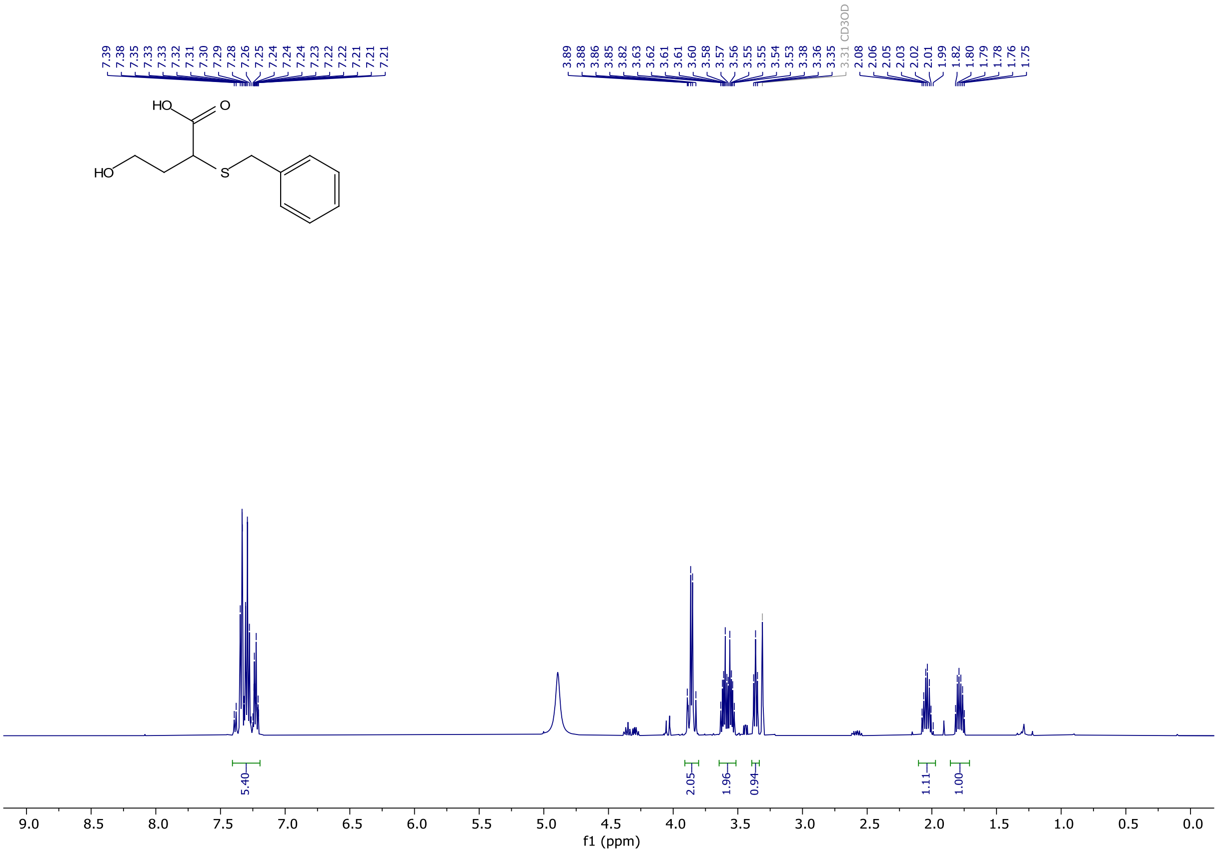

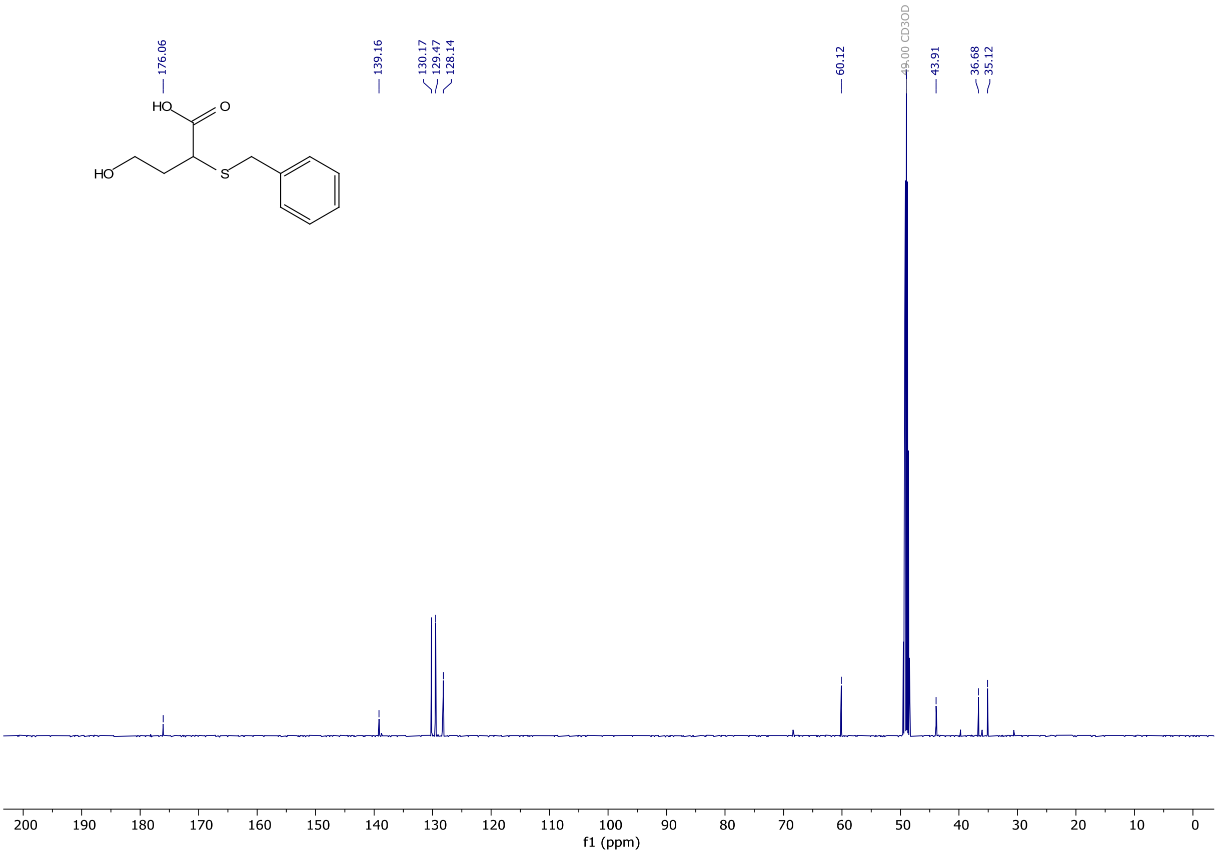

3-Phenoxydihydrothiophen-2(3H)-one 10

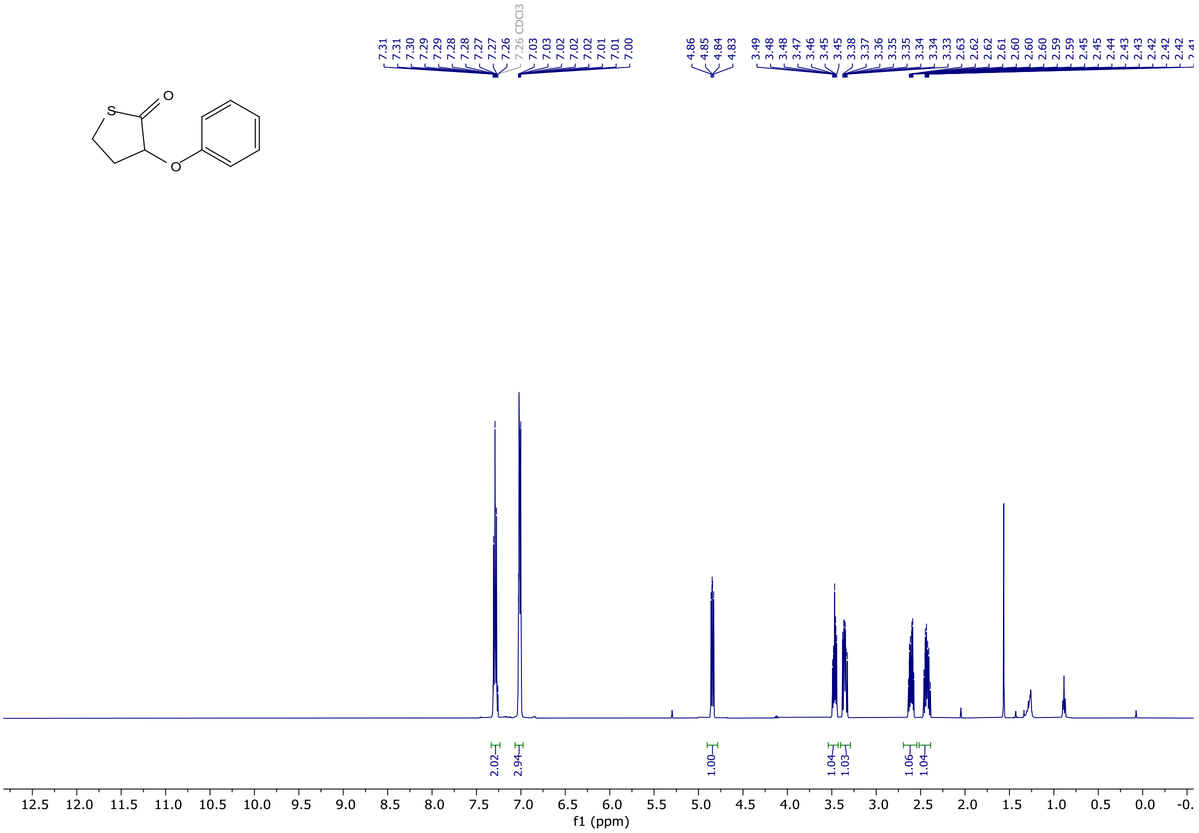

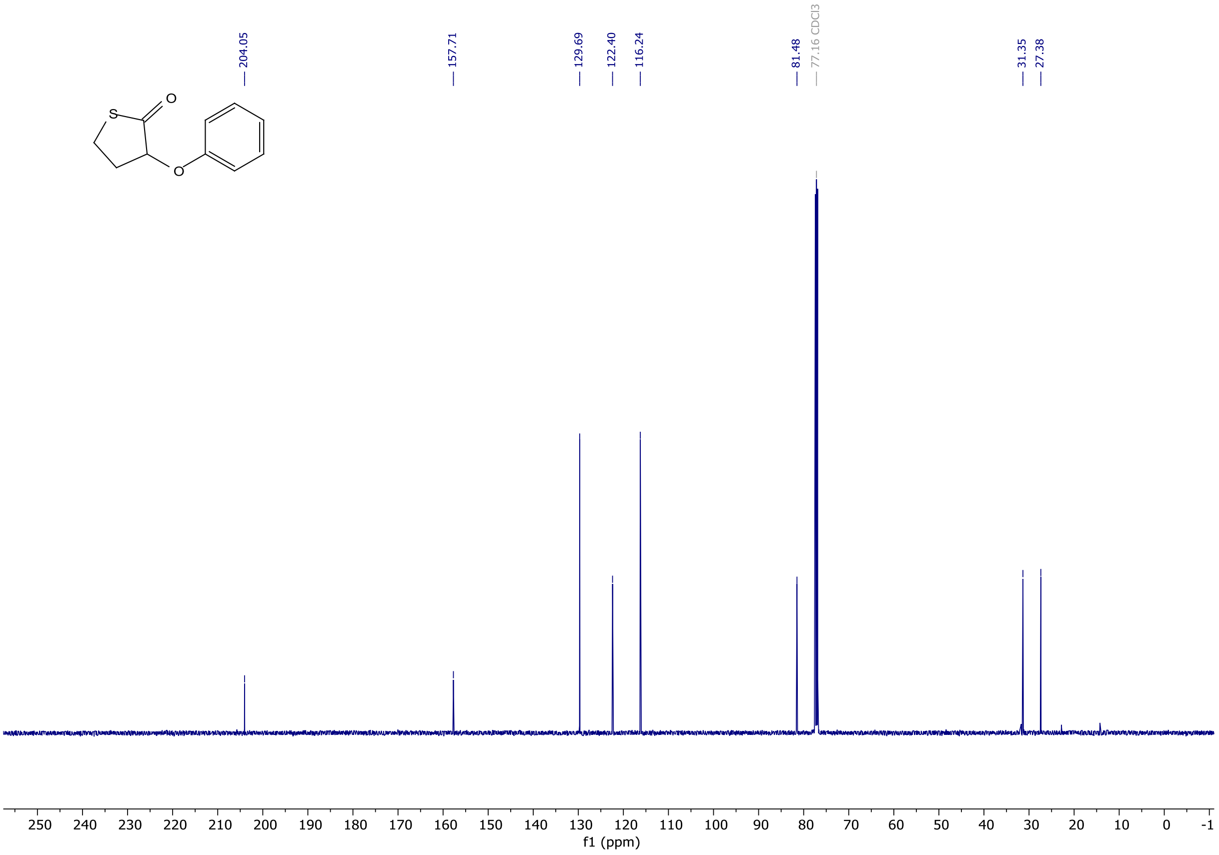

4-Mercapto-2-phenoxybutanoic acid 11

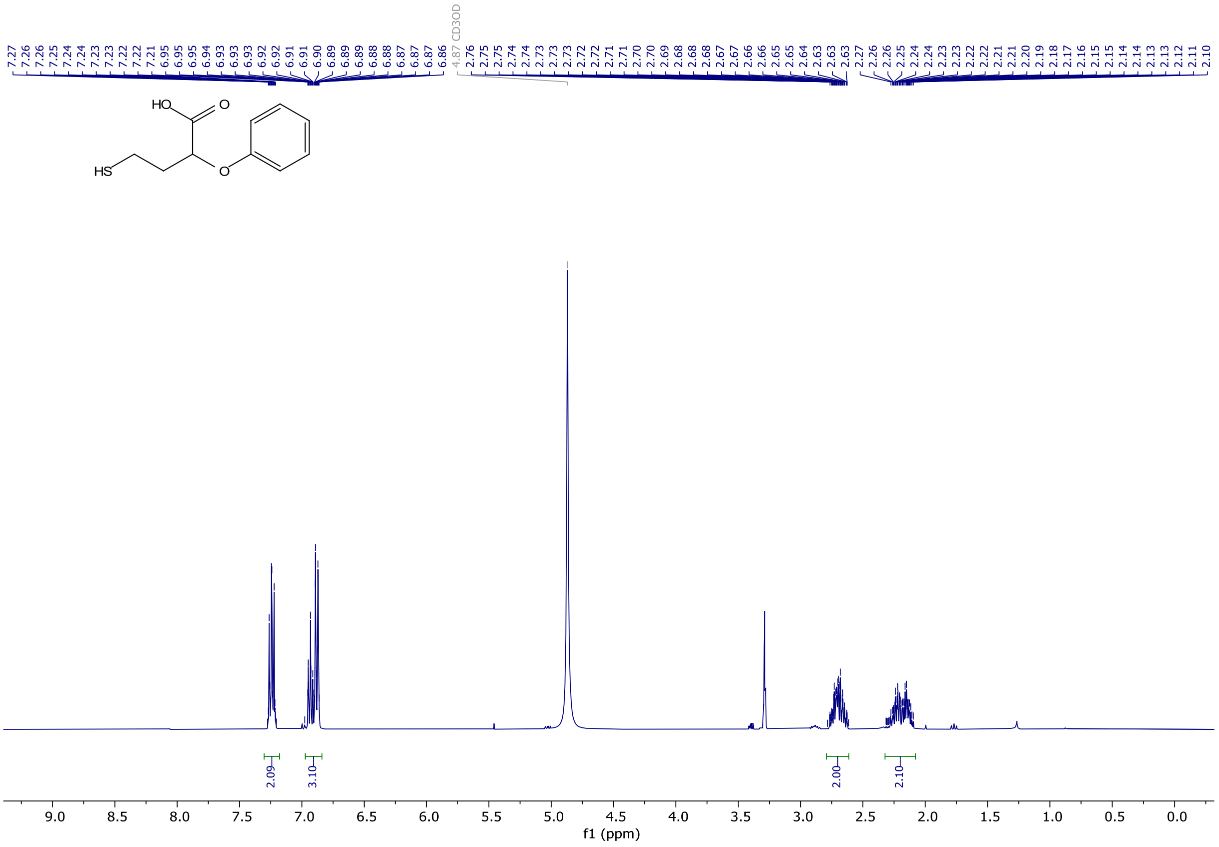

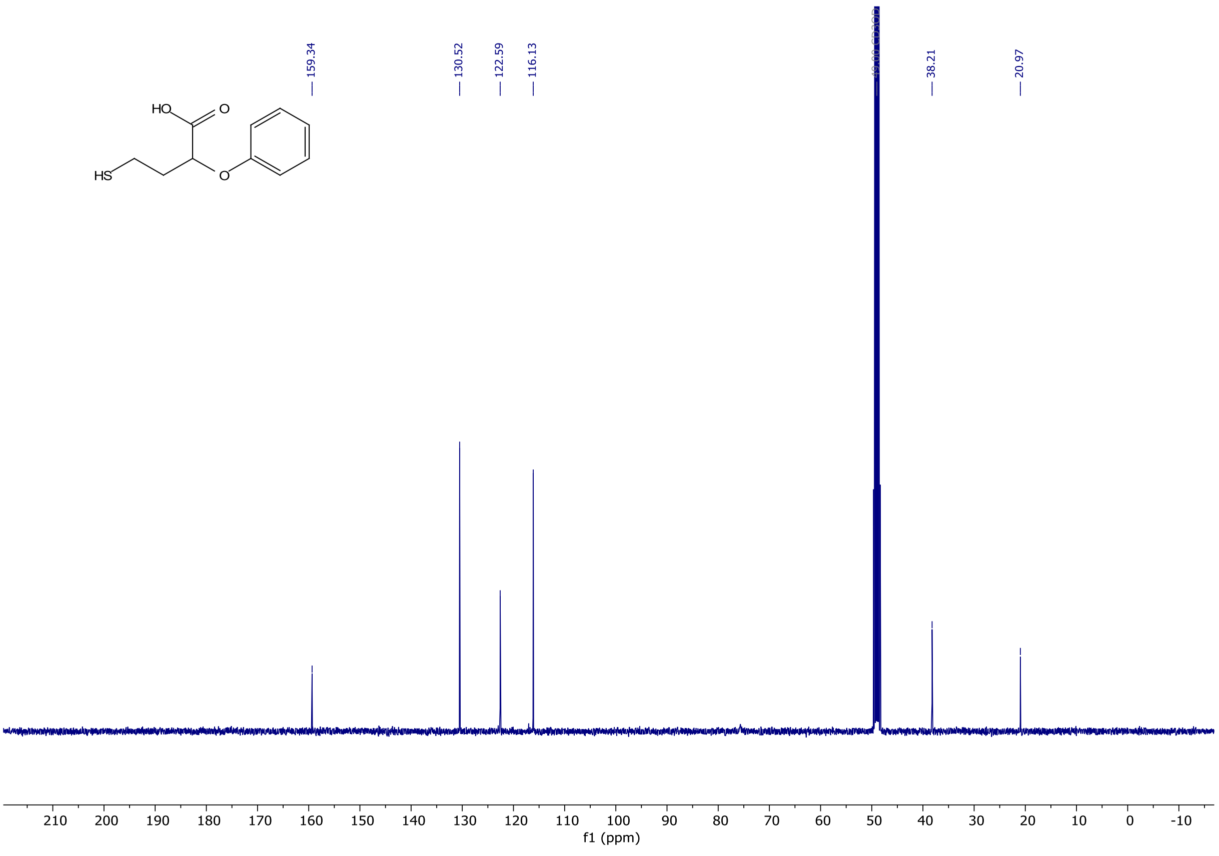

Supplement: Supplementary file 1 — Supporting Information [file ANIE-64-e202505032-s001.pdf]
